# Stereodivergent synthesis of chiral succinimides via Rh-catalyzed asymmetric transfer hydrogenation

**DOI:** 10.1038/s41467-022-35124-5

**Published:** 2022-12-17

**Authors:** Fangyuan Wang, Zongpeng Zhang, Yu Chen, Virginie Ratovelomanana-Vidal, Peiyuan Yu, Gen-Qiang Chen, Xumu Zhang

**Affiliations:** 1 Department of Chemistry, Shenzhen Grubbs Institute and Guangdong Provincial Key Laboratory of Catalysis, Southern University of Science and Technology, Shenzhen, 518000 China; 2 PSL University, Chimie ParisTech, CNRS, Institute1 of Chemistry for Life and Health Sciences, CSB2D team, 75005 Paris, France; 3 Academy for Advanced Interdisciplinary Studies, Southern University of Science and Technology, Shenzhen, 518000 China

**Keywords:** Synthetic chemistry methodology, Asymmetric catalysis

## Abstract

Chiral succinimide moieties are ubiquitous in biologically active natural products and pharmaceuticals. Until today, despite the great interest, little success has been made for stereodivergent synthesis of chiral succinimides. Here, we report a general and efficient method for accessing 3,4-disubstituted succinimides through a dynamic kinetic resolution strategy based on asymmetric transfer hydrogenation. The Rh catalyst system exhibit high activities, enantioselectivities, and diastereoselectivities (up to 2000 TON, up to >99% ee, and up to >99:1 dr). Products with *syn*- and *anti*-configuration are obtained separately by control of the reaction conditions. For the *N*-unprotected substrates, both the enol and the imide group can be reduced by control of reaction time and catalyst loading. In addition, the detailed reaction pathway and origin of stereoselectivity are elucidated by control experiments and theoretical calculations. This study offers a straightforward and stereodivergent approach to the valuable enantioenriched succinimides (all 4 stereoisomers) from cheap chemical feedstocks in a single reaction step.

## Abstract (web summary)

Chiral succinimides are ubiquitous in bioactive compounds. Here, authors report a stereodivergent method for accessing 3,4-disubstituted succinimides, and the origin of stereoselectivity is elucidated by experimental and theoretical investigations.

## Introduction

**Fig. 1:**
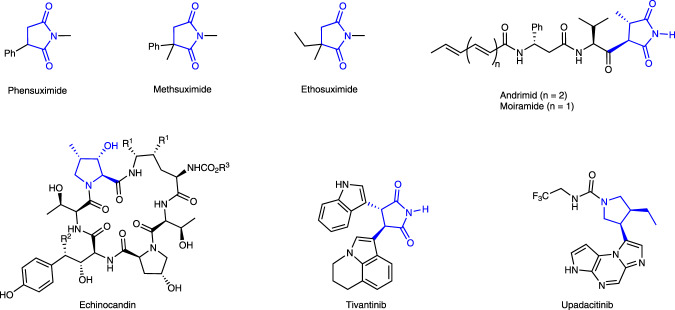
Biologically active compounds and drugs derived from succinimides. Phensuximide, methsuximide and ethosuximide are well-known antiepileptic drugs. Upadacitinib is a drug for the treatment of immune disorders, Echinocandins have antifungal activities, and Tivantinib is used for the treatment of advanced hepatocellular carcinoma.

**Fig. 2:**
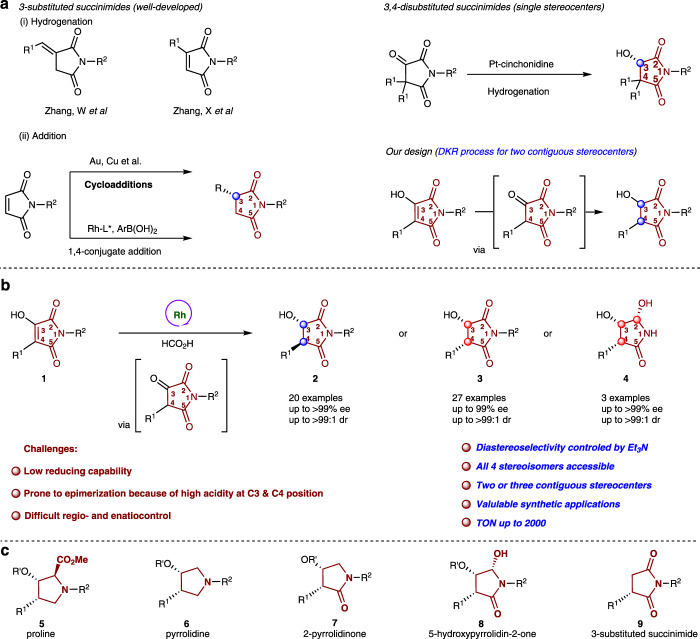
Synthetic methods for the construction of chiral succinimide derivatives. **a** Catalytic asymmetric synthesis of chiral succinimides (previous work)**. b** Stereodivergent synthesis of chiral succinimide derivatives via ATH (this work). **c** Obtained building blocks.

Chiral 3-substituted and 3,4-disubstituted succinimide substructures are widely found in natural products and pharmaceuticals (Fig. 1)^1–6^. Succinimide derivatives such as phensuximide (PTS), methsuximide (MTS) and ethosuximide (ETS), are well-known antiepileptic drugs (AEDs). In recent years, chiral succinimides have received great attention from synthetic chemists due to their wide range of biological activities such as antibacterial^7^, antifungal^8^, analgesic^9^, anticonvulsant^10^, and antitumor effects^11,12^. The most well-developed methods include enantioselective cycloaddition reactions^13–15^ and hydrogenation reaction^16–18^ using maleimides as the substrates. Asymmetric catalytic addition ^19,20^of nucleophilic reagents to maleimides has also been reported (Fig. 2a). However, compared with well-developed synthetic methods of 3-substituted succinimides, few methodologies exist concerning the synthesis of 3,4-disubstituted succinimides^19,21^. The synthon, 3-hydroxy-4-substituted-succinimides can be easily converted to valuable chiral skeletons, such as chiral pyrrolidones and chiral lactams (Fig. 2c). Consequently, developing an efficient synthetic methodology for the construction of 3-hydroxy-4-substituted-succinimides from readily available starting materials is highly desirable.

Since the first asymmetric transfer hydrogenation (ATH) of ketones reported by Noyori and Ikariya using (*S*,*S*)-**cat.1**^22^, a series of catalysts containing *N*-monotosylated 1,2-diphenylethylenediamine (TsDPEN) have been documented by Noyori^22^, Ikariya^23^, Wills^24–26^ and others^27–42^. In 1999, Baiker et al. reported an enantioselective hydrogenation of pyrrolidine-2,3,5-triones using Pt–cinchonidine systems, obtaining 3,4-disubstituted succinimides with a single stereocenter^16^. Inspired by this work, we envisioned that 3-hydroxy-4-substituted maleimides could be reduced through a DKR (dynamic kinetic resolution)-ATH process. Over the past decades, DKR-ATH is an extremely attractive approach for the reduction of *α*-functional ketones due to the assured enantiomeric purity^43–51^. In particular, base is necessary to facilitate the ATH process^52,53^. Compared with well-studied *α*-functional ketones, 3-hydroxy-4-substituted maleimide (1) exists mainly in its enol form^45^, and has low reducing activity under alkaline conditions. It can be reduced through the Pd/C-H_2_ system but cannot be reduced by NaBH_4_/MeOH system (for details, see Supplementary Fig. 9). Moreover, the corresponding reduction products are prone to epimerization under alkaline conditions. This also made it difficult to obtain the reduction products using traditional alkaline reduction systems. Therefore, the development of appropriate asymmetric reduction methods to solve such problems is of great significance.

Herein, we report the stereodivergent enantio- and diastereoselective ATH of maleimide derivatives catalyzed by a tethered rhodium catalyst. Anti-3-hydroxy-4-substituted-succinimides 2 (up to >99% ee, up to >99:1 dr) and *syn*-3-hydroxy-4-substituted-succinimides **3** can be obtained by adjusting the amount of base (up to >99% ee, up to >99:1 dr). In addition, both the enol and the adjacent imide can be reduced to obtain 4 with high chemoselectivity by control the loading of the catalyst (up to >99% ee, up to >99:1 dr) (Fig. 2b).

## Results

### Condition optimization

To investigate the proposed ATH process, model substrate **1a** was subjected to reduction using the commercially available TsDPEN-derived Ru, Rh and Ir complexes (2 mol%) in an azeotropic mixture of formic acid and triethylamine at 25 °C in MeOH. (Table 1) The Noyori’s catalyst (*R*,*R*)-**cat.1** and (*S*,*S*)-**cat.2** cannot achieve any catalytic conversion (entries 1–2). Ir complexes, (*S*,*S*)-**cat.3** can only achieve 23% conversion with 84% ee and 84:16 dr (entry 3). In contrast, the tethered-catalysts, **cat.4**~**cat.5** showed more excellent conversion, enantioselectivity and diastereoselectivity. 96% ee and 90:10 dr were achieved with (*R*,*R*)-**cat.4** while 95% ee and 92:8 dr were achieved with catalyst (*R*,*R*)-**cat.5** (entries 4–5). To our great delight, the catalytic selectivity of Rh catalyst (*R*,*R*)-**cat.6** and (*S*,*S*)-**cat.6** outperformed that of the catalysts previously evaluated, providing corresponding product **2a** with 95:5 dr and 96% ee in an *anti*-selective manner (entries 6–7). (*S*,*S*)-**cat.6** proved to be potential catalysts for the DKR-ATH of **1a**.

**Table 1:** Optimization for Rh(III)-catalyzed DKR-ATH of 1a^a^


entry	catalyst	Hydrogen donor	Solvent	conv. (%)	ee_anti_ (%)	ee_syn_ (%)	dr (*anti*/*syn*)
1	(*R*,*R*)-**cat.1**	HCO_2_H:Et_3_N (5:2)	MeOH	<5	–	–	–
2	(*S*,*S*)-**cat.2**	HCO_2_H:Et_3_N (5:2)	MeOH	<5	–	–	–
3	(*S*,*S*)-**cat.3**	HCO_2_H:Et_3_N (5:2)	MeOH	23	84	–	84:16
4	(*R*,*R*)-**cat.4**	HCO_2_H:Et_3_N (5:2)	MeOH	99	−96	–	90:10
5	(*R*,*R*)-**cat.5**	HCO_2_H:Et_3_N (5:2)	MeOH	90	−95	–	92:8
6	(*R*,*R*)-**cat.6**	HCO_2_H:Et_3_N (5:2)	MeOH	84	−96	–	95:5
7	(*S*,*S*)-**cat.6**	HCO_2_H:Et_3_N (5:2)	MeOH	85	96	–	95:5
8	(*S*,*S*)-**cat.6**	HCO_2_H:Et_3_N (5:2)	hexane	<5	–	–	–
9	(*S*,*S*)-**cat.6**	HCO_2_H:Et_3_N (5:2)	EtOH	71	93	–	97:3
10	(*S*,*S*)-**cat.6**	HCO_2_H:Et_3_N (5:2)	DCM	66	95	–	93:7
11	(*S*,*S*)-**cat.6**	HCO_2_H:Et_3_N (5:2)	THF	81	99	–	96:4
12	(*S*,*S*)-**cat.6**	HCO_2_H:Et_3_N (5:2)	dioxane	97	99	–	93:7
13	(*S*,*S*)-**cat.6**	HCO_2_H:Et_3_N (5:2)	toluene	>99	95	–	98:2
14	(*S*,*S*)-**cat.6**	HCO_2_H:Et_3_N (5:2)	EtOAc	>99	99	–	98:2
15^b^	(*S*,*S*)-**cat.6**	HCO_2_H:Et_3_N (2:0.02)	EtOAc	>99	–	96	2:98
16^c^	(*S*,*S*)-**cat.6**	HCO_2_H:Et_3_N (2:0)	EtOAc	>99	–	93	<1:99
17^d^	(*S*,*S*)-**cat.6**	^*i*^PrOH	^*i*^PrOH	<5	–	–	–
18^e^	(*S*,*S*)-**cat.6**	HCO_2_Na	^*i*^PrOH	<5	–	–	–

^a^Conditions: Catalyst**/1a** (0.1 mmol) ratio of 1:50 in 1 mL of solvent, HCO_2_H/Et_3_N azeotropic mixture (20 μL) at 25 °C for 12 h. Conversions (conv.) were determined by ^1^H NMR analysis. Enantiomeric excesses (ee) and diastereomeric ratios (dr) were determined by HPLC analysis using a chiral stationary phase.

^b^HCO_2_H (2.0 equiv.) was used.

^c^HCO_2_H (2.0 equiv.) was used for 48 h.

^d^KO^*t*^Bu (3.0 equiv.) was used in 1.0 mL of ^*i*^PrOH at 60 °C for 12 h.

^e^HCO_2_Na (5.0 equiv.) was used in 2.0 mL of ^*i*^PrOH /H_2_O (1.0 mL/1.0 mL) at 60 °C for 12 h.

With (*S*,*S*)-**cat.6** as a catalyst, we further explored the effects of solvents and the ratio of formic acid and triethylamine (Table 1). Several solvents, such as EtOH, DCM, THF, dioxane, toluene, hexane and EtOAc were examined with HCO_2_H/Et_3_N (5:2) for 12 h (entries 8–14). Except for a low conversion in hexane (<5% conv.), moderate to high conversion and high stereoselectivity were observed in other solvents. Pleasingly, when EtOAc was used, excellent enantioselectivity and diastereoselectivity were obtained in full conversion (entry 14, 99% ee, 98:2 dr). On the contrary, *syn*-product **3a** was obtained by reducing the amount of trimethylamine (entry 15). When only formic acid was used, the enantioselectivity of the product decreased slightly, and extended reaction time was necessary to achieve full conversion (entry 16). In addition, different ratios of triethylamine and formic acid were investigated in EtOAc (Supplementary Table 1 presents details), indicating that the decrease in the proportion of Et_3_N resulted in a decrease in the proportion of *anti*-product. Finally, isopropanol and sodium formate were also investigated as hydrogen donors, and a very low reactivity was observed under the alkaline environment (entries 17–18, <5% conv.). So far, HCO_2_H/Et_3_N (5:2) was used as a hydrogen donor for *anti*-**2a** (entry 14) and HCO_2_H/Et_3_N (2.0 equiv./0.02 equiv.) for *syn*-**3a** (entry 15).

### Substrate scope

**Fig. 3:**
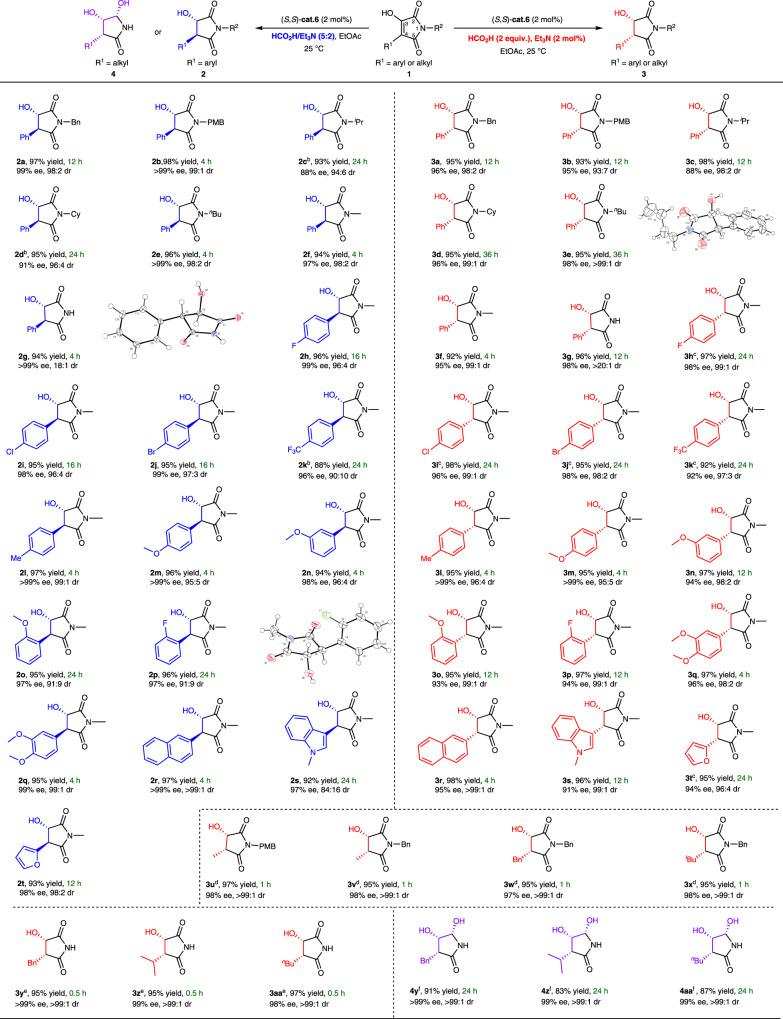
Substrate scope. ^a^Conducted with catalyst/substrate (0.2 mmol) ratio of 1: 50 in 1 mL of solvent, HCO_2_H (2.0 equiv.)/Et_3_N (2 mol%) for *syn*–isomer **3** and HCO_2_H/Et_3_N azeotropic mixture (20 μL) obtained *anti*–isomer **2**. Isolated yield including the minor enantiomer, the ee and dr value of were determined by HPLC analysis using a chiral stationary phase. ^b^(*S*,*S*)-**cat.6** (5 mol%) and HCO_2_H/Et_3_N azeotropic mixture (50 μL) were used. ^c^(*S*,*S*)-**cat.6** (5 mol%) and HCO_2_H (2.0 equiv.) were used for 24 h. ^d^(*S*,*S*)-**cat.6** (1 mol%) and HCO_2_H/Et_3_N azeotropic mixture (20 μL) were used for 1 h. ^e^(*S*,*S*)-**cat.6** (1 mol%) and HCO_2_H/Et_3_N azeotropic mixture (20 μL) were used for 0.5 h. ^f^(*S*,*S*)-**cat.6** (5 mol%) and HCO_2_H/Et_3_N azeotropic mixture (40 μL) were used for 24 h.

Under the optimal conditions, the methodology using of HCO_2_H/Et_3_N (5:2) as a hydrogen source was successfully extended to a series of substrates, and the results were illustrated in Fig. 3. Substrates with the different protective groups on the nitrogen atom were subjected to the standard reaction conditions, and the reaction proceeded smoothly to provide the *anti*-product with high yields, excellent enantioselectivities and diastereoselectivities (**2a**–**2g**, 94–98% yield, 94:6–>99:1 dr, 88%–>99% ee). Next, we evaluated the effect of the substituents on the C4 position. Functional groups, such as halides (**2h**–**2j**), trifluoromethyl (**2k**), methyl (**2l**), methoxy (**2m**) at the *para* position of the phenyl group were compatible with this transformation (88–97% yield, 90:10–99:1 dr, 96%–>99% ee). Substrates with *meta*-substitution on the phenyl group were also tolerated, and 98% ee and 96:4 dr were obtained (**2n**). In addition, the *ortho*-methoxy and *ortho*-fluoro substrate were evaluated with lower dr values (**2o**–**2p**, 95–96% yield, 91:9 dr and 97% ee). Moreover, the product **2r** with 3,4-methoxy groups on the phenyl ring was obtained with 99% ee and 99:1 dr. The current reaction also tolerates substrates bearing 2-naphthyl, 2-furanyl, and 3-indolyl groups (**2r**–**2t**, 92%–95% yield, 84:16–99:1 dr, 97–99% ee).

Subsequently, a sharp contrast was exhibited using HCO_2_H (2.0 equiv.)/Et_3_N (2 mol%) as a hydrogen source, and the *syn-*products could be obtained selectively. (Fig. 3) High enantioselectivities and diastereoselectivities were obtained with a wide range of *N*-protected substrates (**3a**–**3g**, 93–98% yield, 93:7–>99:1 dr, 88–98% ee). The effect of substitution at the C4 position of the substrates was also evaluated, and products with halides, trifluoromethyl, methyl, and methoxy group at the ortho, meta, or para position of the phenyl group were smoothly produced in 92–98% yields with 95:5–99:1 dr and up to >99% ee (**3h**–**3q**). 2-Naphthyl-containing substrates could obtain excellent enantioselectivity and diastereoselectivity as well (**3r**, 98% yield, >99:1 dr, 98% ee). Heterocycle-containing substrates, such as indole (**3s**, 91% ee, 99:1 dr) and furan (**3t**, 94% ee, 96:4 dr) were compatible with the conditions. For substrates with alkyl substitution at the C4 position, using an azeotropic mixture of HCO_2_H/Et_3_N (5:2) as a hydrogen source, this transformation proceeded smoothly to provide the *syn*-product in excellent yields (up to 97%) and with high levels of diastereo- and enantioselectivities (**3u**–**3x**, 97–98% ee, >99:1 dr). What’s more, *N*-unprotected substrates were well tolerated (**3y**–**3aa**, 98%–>99% ee, >99:1 dr). Interestingly, for *N*-unprotected substrates, both the enol and imide groups can be reduced. The products 4,5-dihydroxy-3-alkyl-pyrrolidin-2-one, containing three contiguous stereocenters, can be obtained by increasing the loading of catalyst (**4y**-**4aa**, 98%–>99% ee, >99:1 dr).

### Synthetic applications

**Fig. 4:**
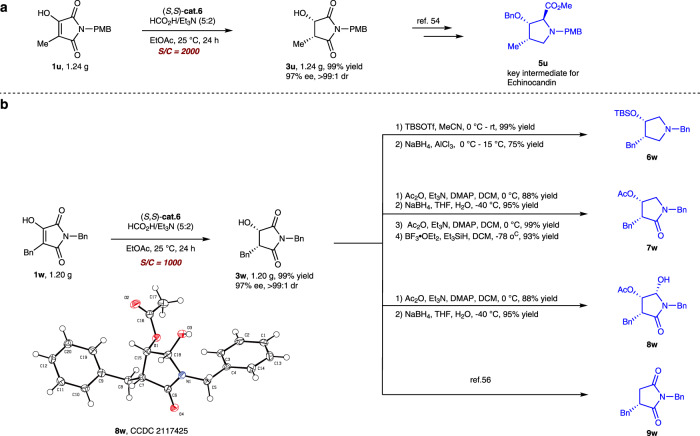
Gram scale synthetic utilities of the DKR-ATH process. **a** Gram-scale experiment of **1u**. **b** Gram-scale experiment of **1w** and transformations of product **3w**.

To demonstrate the synthetic utilities of this methodology, two gram-scale transformations were conducted, and the results were summarized in Fig. 4. To our delight, when 0.05 mol% (S/C = 2000) catalyst loading was used, the gram-scale experiment proceeded smoothly to provide **3u** with excellent results. According to the procedure in the literature, **3u** can be further transformed into the important synthon **5u** (3-hydroxy-4-methylproline), which is a key intermediate for Echinocandin (Fig. 4a)^54^. Similarly, the ATH of **1w** could be performed with a catalyst loading of 0.1 mol% to obtain **3w** with excellent results. Then it can be further transformed into pyrrolidine derivative **6w**, lactam derivatives **7w**^55^**, 8w**, and succinimide derivative **9w**^56^ in high yield and excellent stereoselectivity (Fig. 4b).

### Mechanism study

**Fig. 5:**
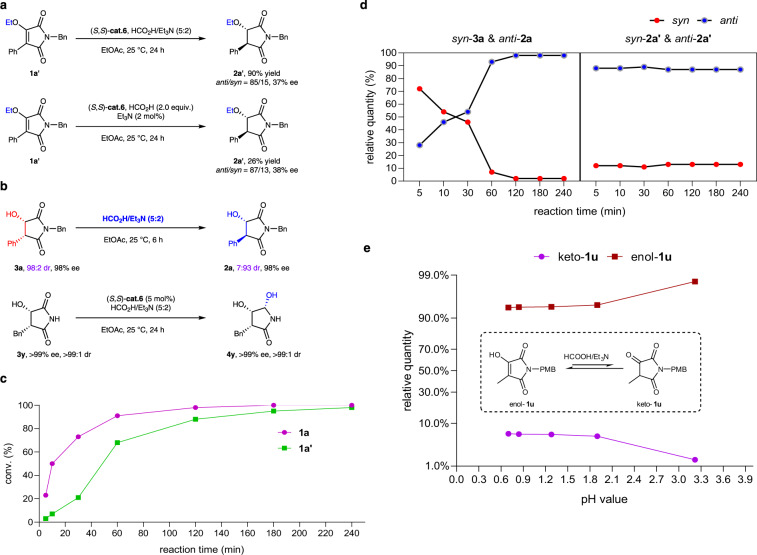
Mechanistic investigations. **a** Reactions of ethyl-protected substrate under standard conditions. **b** Observed effect of HCO_2_H/Et_3_N (5:2) on the diastereoselectivities. **c** Kinetic experiments of **1a** and **1a’**. **d** Monitoring of the reaction process of **1a** and **1a’** under standard conditions. **e** Effect of pH on the relative quantity of enol and keto form of **1u**.

To elucidate the detailed mechanism of this ATH process, a series of mechanistic investigations were conducted, and the results were summarized in Fig. 5. First, the reaction of hydroxyl-protected substrate **1a’** was conducted under the standard conditions, and **2a’** was obtained with only 37–38% ee and 85/15-87/13 dr, which means that the presence of the –OH group at the C3-position is necessary to ensure high enantioselectivity, and the C=C reduction pathway is also possible. (Fig. 5a). Second, the *syn*-product **3a** can be smoothly transformed into the *anti*-product **2a** in the presence of an azeotropic mixture of HCO_2_H/Et_3_N (5:2). This suggested that the *anti*-product was probably produced by epimerization of the *syn*-product. Interestingly, **3y** can be transformed into the **4y** with three contiguous stereocenters (Fig. 5b). Moreover, the reaction kinetic experiment showed that **1a** can be completely reduced in preference to **1a’** under standard conditions (Fig. 5c). During the process, the reduction of **1a** first produced the *syn*-product which isomerized to the *anti*-product along with the process of the reaction, whereas, the reduction of **1a’** obtained the *anti*-product mainly, and the *anti/syn* ratio changed slightly during the reaction process (Fig. 5d). In addition, the keto-enol equilibrium experiment revealed that the ratio of enol-**1u** increased with the increase of pH value in acidic environment (Fig. 5e). Our mechanistic investigations revealed that the current reaction probably proceeded via reduction of the keto form.

**Fig. 6:**
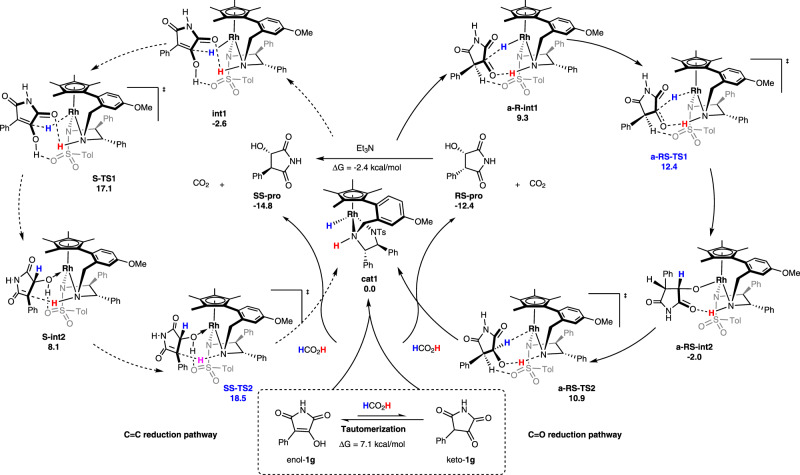
Proposal catalytic cycle for ATH of 1g with HCOOH. The C=C reduction pathway was depicted in the left catalytic cycle and the C=O reduction pathway was described in the catalytic cycle on the right. The Gibbs free energy of the compounds, intermediates and transition states are provided below the corresponding chemical structures and the energies are given in kcal/mol.

The mechanistic investigations and theoretical data^57,58^ accumulated for the ATH of **1** point to two plausible catalytic cycles. Key results from DFT calculations, including the most favorable reaction pathways for C=O and C=C reduction, respectively, are shown in Fig. 6. Active catalyst **cat1** was generated from decarboxylation of the formate complex of **cat.6** (Supplementary Fig. 11 and Supplementary Data 1). In the catalytic cycle for C=O reduction, **cat1** interacts with substrate keto**-1g** to generate the intermediate **a-R-int1**, followed by a direct hydride (H^−^) transfer via transition state **a-RS-TS1**, with an energy barrier of 3.1 kcal/mol relative to **a-R-int1**. This step generates an intermediate **a-RS-int2**, which is more stable than **a-R-int1** by 11.3 kcal/mol. Subsequently, the proton transfer step is completed via **a-RS-TS2** by overcoming the energy barrier of 12.9 kcal/mol relative to **a-RS-int2**. The active catalyst **cat1** is regenerated after releasing of the *syn*-product **RS-pro** followed by proton transfer from formic acid. Finally, considering that active catalyst **cat1** first more easily capture enol-**1g** than keto-**1g** to form a more stable intermediate **int1** in an exergonic process of 2.6 kcal/mol, the free energy barrier of the C=O reduction pathway from **cat1** to product should be 15.0 kcal/mol from **int1** to **a-RS-TS1**. On the other hand, for the catalytic cycle of the C=C reduction, the hydride transfer step suffered from an energy barrier of 19.7 kcal/mol via transition state **S-TS1** relative to **int1**. The following proton transfer step has an energy barrier of 21.1 kcal/mol relative to **int1**. Subsequent release of the *anti*-product **SS-pro** and interaction with formic acid regenerates **cat1** and produces CO_2_. The results show that from **cat1**, the free energy barrier of the most favorable pathway for C=O and C=C reduction are respectively 15.0 kcal/mol (from **int1** to **a-RS-TS1**) and 21.1 kcal/mol (from **int1** to **SS-TS2**), and are consistent with the experimental observation that the formation of the *anti*-product is favored over the *syn*-product. The CO_2_ generated in the asymmetric reduction with formic acid can be effectively removed from the catalytic system. In addition, the theoretical study of the C=C reduction pathway for the hydroxyl-protected substrate was also conducted under standard conditions (Supplementary Fig. 12 and Supplementary Data 1 present the details).

**Fig. 7:**
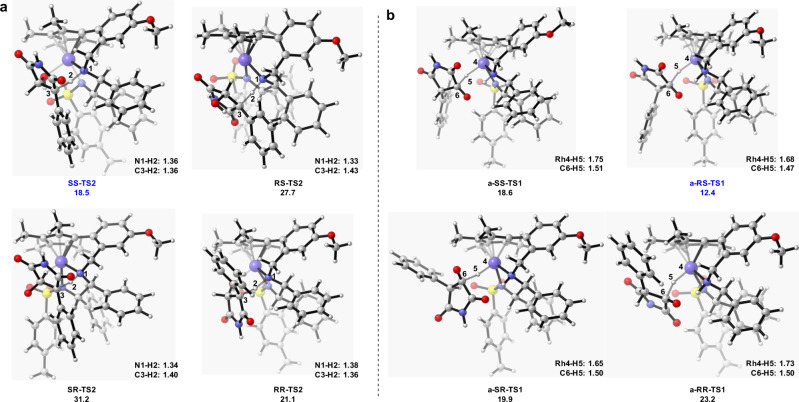
The free energies of the transition states of the enantio-determining steps. **a** Calculated transition states for the C=O bond reduction pathway. **b** Calculated transition states for the C=C bond reduction pathway. The energies are given in kcal/mol. The lengths are given in angstrom.

The free energies and key lengths of the transition states leading to different stereoselective products were also shown in Fig. 7. For the C=O reduction pathway, it is found that the transition state **a-RS-TS1** has a significant advantage relative to other three transition states **a-SS-TS1**, **a-SR-TS1**, and **a-RR-TS1** by 6.2, 7.5, and 10.8 kcal/mol, respectively. The proton transfer step in the C=C reduction pathway is the enantio/rate-determining step, the transition state **SS-TS2** is more stable than the other three transition states **RS-TS2**, **SR-TS2**, and **RR-TS2** by 9.2, 12.7, and 2.6 kcal/mol, respectively. Comparing to the reduction of the C=C, **a-RS-TS1** is more favorable than the corresponding transition states **SS-TS2** by 6.1 kcal/mol. The result showed that reduction of the C=O bond is the more reasonable pathway, resulting in the formation of *syn-*product, which can transform into the *anti*-product in the presence of Et_3_N. The reduction of the C=C path need to overcome a higher energy barrier (21.1 kcal/mol vs 15.0 kcal/mol) and form the *anti*-product in the catalytic cycle, which is also inconsistent with the experimental results of the formation of *syn-*product.

## Discussion

In conclusion, a highly asymmetric transfer hydrogenation of 3-hydroxy-4-substituted-maleimide derivatives was successfully developed using a tethered Rh catalyst under mild reaction conditions. Through strategic modulation of the amount of Et_3_N, a variety of 3-hydroxy-4-substituted-maleimide were transformed into the corresponding *syn-* and *anti*- chiral succinimides with excellent enantio- and diastereoselectivities. This method successfully breaks the inherent impression of single product in previously reported ATH methodologies. Comprehensive mechanistic studies revealed that the -OH group at the C3-position of **1** is crucial for driving the reaction to the high enantioselectivity, and the configuration of the product will undergo epimerization during the ATH process. This also leads to two possible reduction processes (C=O or C=C). Computational analysis revealed that the C=C reduction pathway may suffer a higher energy barrier than the C=O reduction pathway, and it cannot generate the *syn*-product. Thus, the C=O reduction pathway via dynamic kinetics resolution process is reasonable. In addition, gram-scale experiments and varieties of transformation at high TONs provides an efficient way to synthesis chiral pyrrolidine derivatives. The present findings demonstrate successful mechanistic control to realize the ATH of a challenging substrate, which can provide further insight into the development of ATH.

## Methods

### Representative procedure of asymmetric transfer hydrogenation of 1a

To a 10 mL Schleck tube charged with a magnetic stirring bar were added successively substrate **1a** (0.2 mmol, 56 mg), formic acid/trimethylamine azeotropic mixture (5/2) (40 μL), **cat.6** (3 mg, 0.004 mmol) and the solvent (2 mL). The mixture was then stirred at room temperature for the indicated reaction time. After completion, the reaction solution was concentrated and the residue was passed through a short column of silica gel (eluent: EtOAc:PE = 2:1) to produce **2a** as a white solid (55 mg, 97% yield, 99% ee, 98:2 dr). The ee or dr values of compound **2a** were determined by HPLC analysis on a chiral stationary phase (Chiralpak IE column, hexane/isopropanol = 80/20; flow rate = 1.0 mL/min; UV detection at 210 nm; *t*_1_ = 8.9 min, *t*_2_ = 9.9 min, *t*_3_ = 10.6 min, *t*_4_ = 13.4 min (major).

## Supplementary information

Supplementary Information

Description of Additional Supplementary Files

Supplementary Data 1

## Data Availability

The authors declare that the data supporting the findings of this study are available within the paper and its supplementary information files. Crystallographic data for compounds 2g, 2p, 3e, 8w have been deposited in the Cambridge Structural Database with the deposition numbers 2040537, 2074951, 2074952, and 2117425 respectively. Copies of the crystallographic data can be obtained free of charge *via* https://www.ccdc.cam.ac.uk/structures/.
