# Supplementary material for: Stereodivergent synthesis of chiral succinimides via Rh-catalyzed asymmetric transfer hydrogenation
Source: Nat Commun. 2022 Dec 17;13:7794. doi: 10.1038/s41467-022-35124-5 (PMC9759521; doi:10.1038/s41467-022-35124-5)
Supplement: Supplementary file 1 — Supplementary Information [file 41467_2022_35124_MOESM1_ESM.pdf]

Supplementary Information

Stereodivergent Synthesis of Chiral Succinimides via Rh-Catalyzed Asymmetric Transfer Hydrogenation

Fangyuan Wang[#], Zongpeng Zhang[#], Yu Chen[#], Virginie Ratovelomanana-Vidal, Peiyuan Yu*, Gen-Qiang Chen* and Xumu Zhang*

Table of Contents

1. Supplementary Notes 3
2. Supplementary Methods 4
Synthesis of **cat.6** 4
General Procedure for the Synthesis of Substrate 5
Characterization Data of **1** 10
General Procedure of Asymmetric Transfer Hydrogenation of **1** 20
General Procedure 20
Characterization Data of **2**, **3** and **4** 21
Detailed Optimization of Reaction Conditions and Gram-Scale DKR-ATH Procedure 47
3. Supplementary Discussion 49
Mechanistic Study 49
Computational methods 53
Synthetic Applications 55
4. Supplementary Figures 59
Crystallographic Information 59
NMR Spectra of **1** 68
NMR Spectra of the **2**, **3**, **4** 99
HPLC Traces of the Products 158
5. Supplementary References 192

1. Supplementary Notes

Unless otherwise mentioned, all experiments were carried out under an atmosphere of argon or using standard Schlenk techniques. Solvents and reagents were purchased from commercial suppliers and used without further purification. Column Chromatography was performed with silica gel Merck 60 (300-400 mesh). NMR spectra were recorded on a Bruker DPX 400 spectrometer at 400 MHz for ^{1}H NMR, 101 MHz for ^{13}C NMR, 376 MHz for ^{19}F NMR and a Bruker DPX 600 spectrometer at 600 MHz for ^{1}H NMR, 151 MHz for ^{13}C NMR, 565 MHz for ^{19}F NMR. $CDCl_3$, CD_3OD and d^6-DMSO were the solvents used for the NMR analysis, with tetramethylsilane (TMS) as the internal standard. Chemical shifts were reported in ppm and coupling constants were given in Hz. Chemical shifts were reported relative to TMS (0.00 ppm) for ^{1}H NMR and relative to $CDCl_3$ (77.0 ppm) for ^{13}C NMR, CD_3OD (49.0 ppm) for ^{13}C NMR, d^6-DMSO (39.5 ppm) for ^{13}C NMR. HPLC analysis was carried out on Agilent 1260 Series instrument using a chiral stationary phase. PE refers to petroleum ether, and EA refers to ethyl acetate, HOBT refers to 1-Hydroxybenzotriazole, EDCI refers to 1-(3-Dimethylaminopropyl)-3-ethylcarbodiimide hydrochloride, CDI refers to N,N'-Carbonyldiimidazole. Catalyst **cat.1~cat.5** were purchased from LaaJoo.

2. Supplementary Methods

Synthesis of cat.6

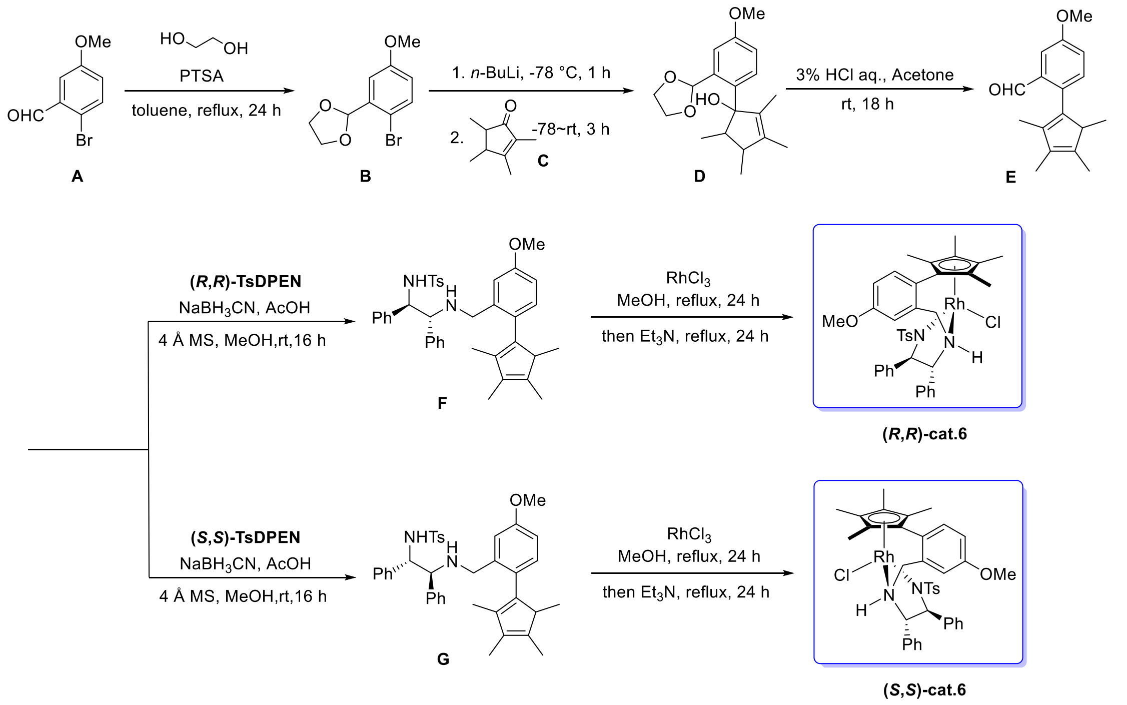

Supplementary Figure 1. Synthetic route of compound **cat.6**.

cat.6 were synthesized according to the literature.[1]

step 1. A mixture of **A** (10.0 g, 46.4 mmol), ethylene glycol (6.2 mL, 11.3 mmol) and *p*-TsOH (112 mg, 0.64 mmol) in toluene (60 mL) was refluxed under a Dean-Stark water separator for 24 h. The cooled mixture was washed with H_2O and brine. The organic layer was dried over Na_2SO_4 and concentrated to afforded **B** (12.0 g, quant.).

step 2. To a solution of **B** (12.0 g, 46.4 mmol) in Et_2O (70 mL) was cooled to -78 °C and *n*BuLi was added dropwise (20 mL, 2.5 M in hexane, 50 mmol). The mixture was stirred at -78 °C for 1.5 h. 2,3,4,5-tetramethylcyclopentenone (7.5 mL, 49 mmol) was added dropwise to the mixture at -78 °C and the reaction was allowed to warm to rt and stirred for 3 h. Toluene and water (50 mL/50 mL) were added and the aqueous layer was extracted with toluene. The combined organic layers were washed with brine, dried over $MgSO_4$ and concentrated to afford crude **D.**

step 3. To a solution of **D** in THF (200 mL) and acetone (30 mL) was added 3% aqueous

HCl solution (120 mL). The mixture was stirred 10 h at rt. When the start material D completely consumed, toluene was added to the mixture and the organic layer was washed with H_2O then brine, dried over Na_2SO_4 The combined organic layer was concentrated under vacuum. The crude residue was purified by flash chromatography (SiO_2, petroleum ether/EtOAc: 98/2) to give **E**

step 4. To a solution of compound **E** (1.08 g, 4.2 mmol) in dry MeOH (50 mL) was added (*R*,*R*)-TsDPEN (1.8 g, 5 mmol) followed by the addition of 1.4 g of molecular sieves (4 Å) and 2 drops of AcOH. The mixture was stirred at rt for 5 h then sodium cyanoborohydride (340 mg, 5.4 mmol) was added and the reaction was stirred overnight at rt. After removal of the molecular sieves and evaporation of MeOH, the residue was redissolved in EtOAc (80 mL). The organic layer was washed with saturated $NaHCO_3$ then brine, dried over Na_2SO_4 and concentrated. Purification of the residue by flash chromatography (SiO_2, pentane/ EtOAc: 9/1 to 8/2) afforded **F** (1.6 g, 61%) as a white solid. (**G** was obtained using the (*S*,*S*)-TsDPEN)

step 5. To a solution of **F** (1.5 g, 2.44 mmol) in MeOH (50 mL) was added rhodium(III) chloride hydrate (500 mg, 2.44 mmol) and the reaction mixture was heated under reflux for 23 h. Triethylamine (0.7 mL, 5 mmol) was then added, the mixture was refluxed for a further 20 h and concentrated. The residue was triturated with water and the solid was filtered, washed with water and dried under vacuum. Purification of the black solid by flash chromatography (SiO_2, EtOAc/cyclohexane: 1/1 to EtOAc/MeOH: 95/5) afforded **(*R*,*R*)-cat.6** (Using the same procedure with **G**, **(*S*,*S*)-cat.6** was obtained).

General Procedure for the Synthesis of Substrate

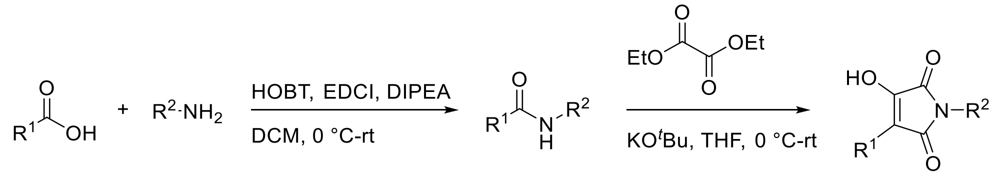

S1 **S2** **1a-1e, 1u-1x**

Supplementary Figure 2. Synthetic route of compound **1a-1e, 1u-1x**.

Procedure A：Preparation of substrate 1a-1e, 1u-1x.[2]

Step 1. To a 250 mL Schleck tube charged with a magnetic stirring bar were added successively compound **S1** (15 mmol), corresponding amine (10 mmol), HOBT (15 mmol), EDCI (15 mmol), *N,N*-Diisopropylethylamine (DIPEA, 30 mmol) and DCM (20 mL) at ambient temperature. The mixture was stirred at room temperature for 1~16 h until the start materials was consumed completely. The mixture was quenched by H_2O (15 mL) and DCM (20 mL), and then treated with 1 M HCl to adjust the pH to 7. After above process, the mixture was extracted by DCM (30 mL*2), the combined organic layer was washed by saturated $NaHCO_3$ (aq.) and brine, dried over anhydrous Na_2SO_4, filtered and concentrated under vacuum. The crude product **S2** was used into the next step without further purification.

Step 2. To a solution of KO^{t}Bu (2.48 g, 22 mmol, 2.2 equiv.) in THF (40 mL) was added amide **S2** (10 mmol, 1.0 equiv.) at 0℃ with stirring for 10 min and then the diethyl oxalate (10 mmol, 1.0 equiv.) was added immediately to the above THF solution. The mixture was stirred at room temperature for 1~16 h until the start materials was consumed completely. The mixture was quenched by H_2O (15 mL) and EtOAc (20 mL), and then treated with 2 M HCl to adjust the pH to 7. After above process, the mixture was extracted by EtOAc (30 mL*2), the combined organic layer was washed by brine, dried over anhydrous Na_2SO_4, filtered and concentrated under vacuum. The crude product was purified by chromatography (PE:EA from 10:1 to 1:3), and then recrystallization with EtOAc and hexane. Compound **1a-1e, 1u-1x** was obtained with medium to high yield (55% ~ 85%).

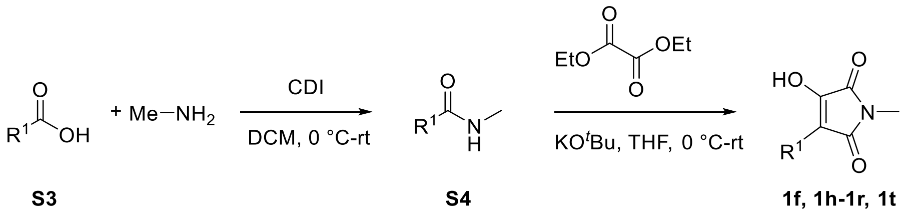

Supplementary Figure 3. Synthetic route of compound **1f, 1h-1r, 1t**.

Procedure B：Preparation of substrate 1f, 1h-1r, 1t

Step 1. To a 250 mL Schleck tube charged with a magnetic stirring bar were added successively acid **S3** (10 mmol, 1.0 equiv.), N,N'-Carbonyldiimidazole (CDI, 15 mmol,

1.5 equiv.) and DCM (20 mL), and then methylamine (5 mL, 33% in MeOH) was added at 0 °C slowly. After addition finished, the mixture was stirred at room temperature for 2-8 h until the start materials was consumed completely. The mixture was quenched by H_2O (15 mL) and DCM (20 mL), and then treated with 1 M HCl to adjust the pH to 7. After above process, the mixture was extracted by DCM (30 mL*2), the combined organic layer was washed by saturated $NaHCO_3$ (aq.) and brine, dried over anhydrous Na_2SO_4, filtered and concentrated under vacuum. The crude product **S4** was used into the next step without further purification.

Step 2. To a solution of KO^{t}Bu (2.48 g, 22 mmol, 2.2 equiv.) in THF (40 mL) was added amide **S4** (10 mmol, 1.0 equiv.) at 0℃ with stirring for 10 min and then the diethyl oxalate (10 mmol, 1.0 equiv.) was added immediately to the above THF solution. The mixture was stirred at room temperature for 1~16 h until the start materials was consumed completely. The mixture was quenched by H_2O (15 mL) and EtOAc (20 mL), and then treated with 2 M HCl to adjust the pH to 7. After above process, the mixture was extracted by EtOAc (30 mL*2), the combined organic layer was washed by brine, dried over anhydrous Na_2SO_4, filtered and concentrated under vacuum. The crude product was purified by chromatography (PE:EA from 10:1 to 1:3), and then recrystallization with EtOAc and hexane. Compound **1f, 1h-1r, 1t** was obtained with medium to high yield (60% ~ 91%).

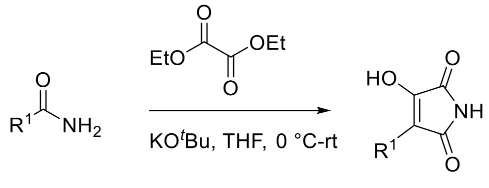

Supplementary Figure 4. Synthetic route of compound **1g, 1y-1aa**.

Procedure C：Preparation of substrate 1g, 1y-1aa.

To a solution of KO^{t}Bu (2.48 g, 22 mmol, 2.2 equiv.) in THF (40 mL) was added amide **S5** (10 mmol, 1.0 equiv.) at 0℃ with stirring for 10 min and then the diethyl oxalate (10 mmol, 1.0 equiv.) was added immediately to the above THF solution. The mixture was stirred at room temperature for 1~16 h until the start materials was consumed

completely. The mixture was quenched by H_2O (15 mL) and EtOAc (20 mL), and then treated with 2 M HCl to adjust the pH to 7. After above process, the mixture was extracted by EtOAc (30 mL*2), the combined organic layer was washed by brine, dried over anhydrous Na_2SO_4, filtered and concentrated under vacuum. The crude product was purified by chromatography (PE:EA from 10:1 to 1:3), and then recrystallization with EtOAc and hexane. Compound **1f, 1h-1r, 1t** was obtained with medium to high yield (75% ~ 85%).

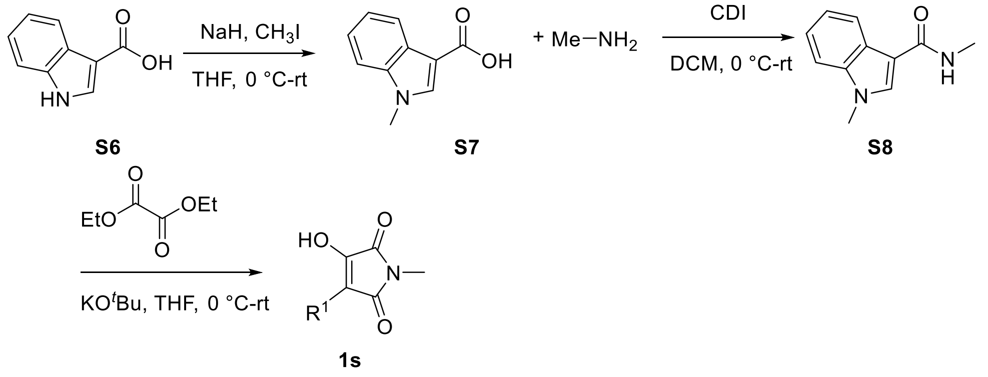

Supplementary Figure 5. Synthetic route of compound **1s**.

Procedure D：Preparation of substrate 1s

Step 1: To a solution of NaH (800 mg, 60% in oil, 1.0 equiv.) in THF (50 mL) was cooled to 0℃. The corresponding acid **S6** (20 mmol, 1.0 equiv.) was added slowly to the mixture with stirring 10 min. Then CH_3I (1.5 g, 40 mmol, 1.0 equiv.) was added into the mixture and the mixture was stirred at room temperature for 3 h. TLC indicated the acid was consumed completely and the mixture was quenched by H_2O (20 mL), HCl (3 M, 10 mL) was added into the mixture and extracted by EtOAc (50 mL*2), the combined organic layer was washed by saturated NH_4Cl (aq.) (20 mL*1) and brine (20 mL*1), dried over anhydrous Na_2SO_4, filtered and concentrated under vacuum. The crude product was purified by chromatography (PE:EA from 50:1 to 9:1). **S7** was obtained as a white solid (81% yield).

Step 2. To a 250 mL Schleck tube charged with a magnetic stirring bar were added successively acid **S3** (10 mmol, 1.0 equiv.), CDI (15 mmol, 1.5 equiv.) and DCM (20 mL), and then methylamine (5 mL, 33% in MeOH) was added at 0 °C slowly. After addition finished, the mixture was stirred at room temperature for 2-8 h until the start

materials was consumed completely. The mixture was quenched by H_2O (15 mL) and DCM (20 mL), and then treated with 1 M HCl to adjust the pH to 7. After above process, the mixture was extracted by DCM (30 mL*2), the combined organic layer was washed by saturated $NaHCO_3$ (aq.) and brine, dried over anhydrous Na_2SO_4, filtered and concentrated under vacuum. The crude product **S4** was used into the next step without further purification.

Step 3. To a solution of KO^{t}Bu (2.48 g, 22 mmol, 2.2 equiv.) in THF (40 mL) was added amide **S4** (10 mmol, 1.0 equiv.) at 0℃ with stirring for 10 min and then the diethyl oxalate (10 mmol, 1.0 equiv.) was added immediately to the above THF solution. The mixture was stirred at room temperature for 1~16 h until the start materials was consumed completely. The mixture was quenched by H_2O (15 mL) and EtOAc (20 mL), and then treated with 2 M HCl to adjust the pH to 7. After above process, the mixture was extracted by EtOAc (30 mL*2), the combined organic layer was washed by brine, dried over anhydrous Na_2SO_4, filtered and concentrated under vacuum. The crude product was purified by chromatography (PE:EA from 10:1 to 1:3), and then recrystallization with EtOAc and hexane. Compound **1s** was obtained in 68% yield.

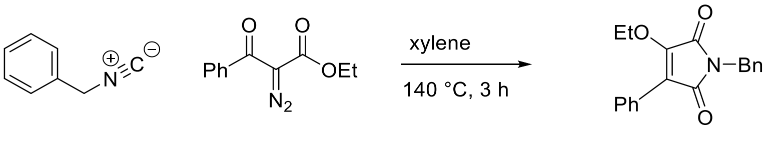

Supplementary Figure 6. Synthetic route of compound **1a'**.[3]

To a solution of Diazoketones **S9** (0.2 mmol, 1.0 equiv) and (isocyanomethyl)benzene 2 (0.25 mmol, 1.25 equiv) in xylene (2 mL) was stirred under Ar atmosphere in an oil bath at 140 °C for 3 h. Then, the reaction mixture was cooled to room temperature and concentrated in vacuo. The residue was purified by column chromatography on silica gel using PE:EA (20:1) as the eluent to give the desired product **1a'**.

Characterization Data of 1

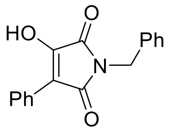

1-benzyl-3-hydroxy-4-phenyl-1*H*-pyrrole-2,5-dione (1a)[4]: yellow solid, 2.20 g, 80% yield. **^{1}H NMR** (400 MHz, DMSO-d_6) δ 8.05 – 7.92 (m, 2H), 7.46 – 7.39 (m, 2H), 7.39 – 7.33 (m, 2H), 7.33 – 7.25 (m, 4H), 4.66 (s, 2H). **^{13}C NMR** (101 MHz, DMSO-d_6) δ 171.1, 166.5, 153.6, 137.1, 129.8, 128.7, 128.5, 127.6, 127.6, 127.5, 127.4, 106.0, 40.5. **HRMS** (ESI-TOF) m/z: $[M-H]^-$ Calcd for $C_{17}H_{12}NO_3^-$ = 278.0823; Found 278.0820.

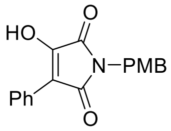

3-hydroxy-1-(4-methoxybenzyl)-4-phenyl-1*H*-pyrrole-2,5-dione (**1b)**: This is a new compound, yellow solid, 2.63 g, 85% yield. **^{1}H NMR** (600 MHz, DMSO-d_6) δ 7.96 (d, *J* = 7.9 Hz, 2H), 7.41 (t, *J* = 7.7 Hz, 2H), 7.29 (t, *J* = 7.4 Hz, 1H), 7.23 (d, *J* = 8.4 Hz, 2H), 6.90 (d, *J* = 8.4 Hz, 2H), 4.57 (s, 2H), 3.72 (s, 3H). **^{13}C NMR** (151 MHz, DMSO-d_6) δ 171.0, 166.4, 158.6, 153.5, 129.7, 129.0, 128.8, 128.3, 127.5, 127.4, 113.9, 105.7, 55.1, 39.9. **HRMS** (ESI-TOF) m/z: $[M-H]^-$ Calcd for $C_{18}H_{14}NO_4^-$ = 308.0928; Found 308.0926.

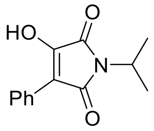

3-hydroxy-1-isopropyl-4-phenyl-1*H*-pyrrole-2,5-dione (**1c)**: This is a new compound, yellow solid, 1.62 g, 70% yield. **^{1}H NMR** (400 MHz, Chloroform-*d*) δ 8.13 – 7.95 (m, 2H), 7.49 – 7.36 (m, 2H), 7.36 – 7.29 (m, 1H), 4.48 – 4.34 (m, 1H), 1.44 (d, *J* = 6.9 Hz, 6H). **^{13}C NMR** (101 MHz, Chloroform-*d*) δ 170.6, 167.5, 149.0, 128.6,

128.5, 128.3, 128.3, 107.2, 43.1, 20.3. **HRMS** (ESI-TOF) m/z: $[M-H]^-$ Calcd for $C_{13}H_{12}NO_3^-$ = 230.0823; Found 230.0818.

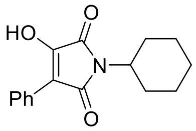

1-cyclohexyl-3-hydroxy-4-phenyl-1*H*-pyrrole-2,5-dione (**1d)**: This is a new compound, yellow solid, 1.76 g, 65% yield. **^{1}H NMR** (400 MHz, Chloroform-*d*) δ 8.03 (d, *J* = 7.4 Hz, 2H), 7.48 – 7.38 (m, 2H), 7.38 – 7.28 (m, 1H), 4.05 – 3.89 (m, 1H), 2.18 – 1.99 (m, 2H), 1.92 – 1.79 (m, 2H), 1.79 – 1.60 (m, 3H), 1.50 – 1.16 (m, 3H). **^{13}C NMR** (101 MHz, Chloroform-*d*) δ 170.8, 167.8, 149.2, 128.6, 128.5, 128.4, 107.2, 50.9, 30.2, 26.0, 25.1. **HRMS** (ESI-TOF) m/z: $[M-H]^-$ Calcd for $C_{16}H_{16}NO_3^-$ = 270.1136; Found 270.1134.

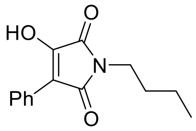

1-butyl-3-hydroxy-4-phenyl-1*H*-pyrrole-2,5-dione (**1e)**: This is a new compound, yellow solid, 1.77 g, 72% yield. **^{1}H NMR** (400 MHz, Chloroform-*d*) δ 8.05 (d, *J* = 7.4 Hz, 2H), 7.50 – 7.30 (m, 3H), 3.58 (t, *J* = 7.2 Hz, 2H), 1.62 (p, *J* = 7.5 Hz, 2H), 1.46 – 1.26 (m, 2H), 0.94 (t, *J* = 7.3 Hz, 3H). **^{13}C NMR** (101 MHz, Chloroform-*d*) δ 170.8, 168.0, 149.4, 128.5, 128.5, 128.4, 128.3, 107.8, 37.8, 30.6, 20.0, 13.6. **HRMS** (ESI-TOF) m/z: $[M-H]^-$ Calcd for $C_{14}H_{14}NO_3^-$ = 244.0979; Found 244.0975.

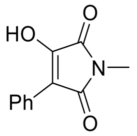

3-hydroxy-1-methyl-4-phenyl-1*H*-pyrrole-2,5-dione (**1f)**[2]: yellow solid, 1.52 g, 75% yield. **^{1}H NMR** (400 MHz, DMSO-*d*6) δ 7.95 (d, *J* = 6.9 Hz, 2H), 7.49 – 7.35 (m, 2H), 7.33 – 7.18 (m, 1H), 2.91 (s, 3H). **^{13}C NMR** (101 MHz, DMSO-*d*6) δ 171.3,

166.6, 153.2, 129.7, 128.3, 127.4, 127.3, 105.8, 23.2. **HRMS** (ESI-TOF) m/z: $[M-H]^-$ Calcd for $C_{11}H_8NO_3^-$ = 202.0510; Found 202.0503.

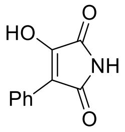

3-hydroxy-4-phenyl-1*H*-pyrrole-2,5-dione (1g)[2]: yellow solid, 1.61 g, 85% yield. **^{1}H NMR** (400 MHz, DMSO-d_6) δ 10.68 (s, 1H), 8.01 – 7.84 (m, 2H), 7.41 (t, *J* = 7.7 Hz, 2H), 7.29 (t, *J* = 7.4 Hz, 1H). **^{13}C NMR** (101 MHz, DMSO-d_6) δ 172.3, 167.8, 153.1, 129.8, 128.2, 127.6, 127.3, 106.5. **HRMS** (ESI-TOF) m/z: $[M-H]^-$ Calcd for $C_{10}H_6NO_3^-$ = 188.0353; Found 188.0344.

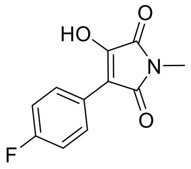

3-(4-fluorophenyl)-4-hydroxy-1-methyl-1*H*-pyrrole-2,5-dione (1h)[2]: yellow solid, 1.33 g, 60% yield. **^{1}H NMR** (400 MHz, DMSO-d_6) δ 8.09 – 7.95 (m, 2H), 7.32 – 7.18 (m, 2H), 2.91 (s, 3H). **^{13}C NMR** (101 MHz, DMSO-d_6) δ 171.3, 166.6, 161.0 (d, *J* = 245.6 Hz), 153.1, 129.4 (d, *J* = 8.0 Hz), 126.3 (d, *J* = 3.2 Hz), 115.3 (d, *J* = 21.4 Hz), 104.9, 23.2. **^{19}F NMR** (376 MHz, DMSO-d_6) δ -113.5. **HRMS** (ESI-TOF) m/z: $[M-H]^-$ Calcd for $C_{11}H_7FNO_3^-$ = 220.0415; Found 220.0410.

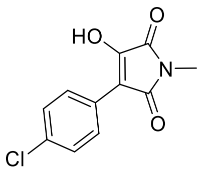

3-(4-chlorophenyl)-4-hydroxy-1-methyl-1*H*-pyrrole-2,5-dione (1i)[2]: yellow solid, 1.66 g, 70% yield. **^{1}H NMR** (600 MHz, DMSO-d_6) δ 7.99 (d, *J* = 7.8 Hz, 2H), 7.47 (d, *J* = 7.9 Hz, 2H), 2.91 (s, 3H). **^{13}C NMR** (151 MHz, DMSO-d_6) δ 171.2, 166.4, 154.2, 131.5, 128.9, 128.8, 128.4, 104.3, 23.2. **HRMS** (ESI-TOF) m/z: $[M-H]^-$ Calcd for $C_{11}H_7ClNO_3^-$ = 236.0120; Found 236.0116.

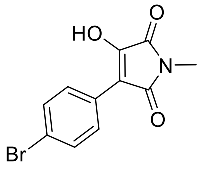

3-(4-bromophenyl)-4-hydroxy-1-methyl-1*H*-pyrrole-2,5-dione (1j): This is a new compound, yellow solid, 2.17 g, 77% yield. **^{1}H NMR** (400 MHz, Methanol-d_4) δ 8.03 – 7.90 (m, 2H), 7.57 – 7.45 (m, 2H), 2.96 (s, 3H). **^{13}C NMR** (101 MHz, Methanol-d_4) δ 172.7, 167.9, 154.4, 132.3, 130.6, 130.3, 122.3, 106.8, 23.5. **HRMS** (ESI-TOF) m/z: $[M-H]^-$ Calcd for $C_{11}H_7BrNO_3^-$ = 279.9615; Found 279.9613.

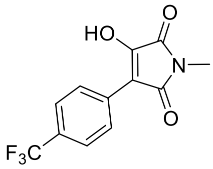

3-hydroxy-1-methyl-4-(4-(trifluoromethyl)phenyl)-1*H*-pyrrole-2,5-dione (1k): This is a new compound, yellow solid, 1.63 g, 60% yield. **^{1}H NMR** (400 MHz, DMSO-d_6) δ 8.17 (d, *J* = 8.3 Hz, 2H), 7.72 (d, *J* = 8.5 Hz, 2H), 2.91 (s, 3H). **^{13}C NMR** (101 MHz, DMSO-d_6) δ 171.0, 166.2, 156.1, 134.2, 127.3, 126.8 (q, *J* = 31.9 Hz), 125.1 (q, *J* = 3.8 Hz), 124.3 (q, *J* = 271.8 Hz), 103.5, 23.2. **^{19}F NMR** (376 MHz, DMSO-d_6) δ -61.1. **HRMS** (ESI-TOF) m/z: $[M-H]^-$ Calcd for $C_{12}H_7F_3NO_3^-$ = 270.0384; Found 270.0382.

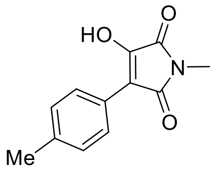

3-hydroxy-1-methyl-4-(p-tolyl)-1*H*-pyrrole-2,5-dione (1l): This is a new compound, yellow solid, 1.91 g, 88% yield. **^{1}H NMR** (400 MHz, DMSO-d_6) δ 7.85 (d, *J* = 8.1 Hz, 2H), 7.22 (d, *J* = 8.0 Hz, 2H), 2.90 (s, 3H), 2.30 (s, 3H). **^{13}C NMR** (101 MHz, DMSO-d_6) δ 171.4, 166.7, 152.4, 136.7, 128.9, 127.3, 126.9, 106.1, 23.2, 20.9. **HRMS** (ESI-TOF) m/z: $[M-H]^-$ Calcd for $C_{12}H_{10}NO_3^-$ = 216.0666; Found 216.0660.

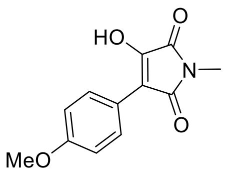

3-hydroxy-4-(4-methoxyphenyl)-1-methyl-1*H*-pyrrole-2,5-dione (1m)[2]: yellow solid, 1.98 g, 85% yield. **^{1}H NMR** (400 MHz, DMSO-d_6) δ 7.98 – 7.87 (m, 2H), 7.05 – 6.94 (m, 2H), 3.78 (s, 3H), 2.90 (s, 3H). **^{13}C NMR** (101 MHz, DMSO-d_6) δ 171.5, 166.9, 158.4, 151.2, 128.9, 122.2, 113.8, 106.2, 55.1, 23.2. **HRMS** (ESI-TOF) m/z: $[M-H]^-$ Calcd for $C_{12}H_{10}NO_4^-$ = 232.0615; Found 232.0610.

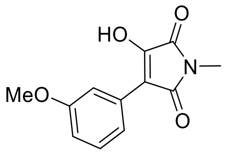

3-hydroxy-4-(3-methoxyphenyl)-1-methyl-1*H*-pyrrole-2,5-dione (1n): This is a new compound, yellow solid, 1.86 g, 80% yield. **^{1}H NMR** (600 MHz, DMSO-d_6) δ 7.63 – 7.48 (m, 2H), 7.37 – 7.26 (m, 1H), 6.91 – 6.83 (m, 1H), 3.76 (s, 3H), 2.90 (s, 3H). **^{13}C NMR** (151 MHz, DMSO-d_6) δ 171.2, 166.6, 159.0, 153.5, 131.0, 129.3, 119.9, 112.9, 112.8, 105.5, 54.9, 23.2. **HRMS** (ESI-TOF) m/z: $[M-H]^-$ Calcd for $C_{12}H_{10}NO_4^-$ = 232.0615; Found 232.0610.

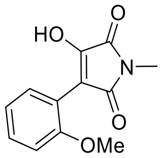

3-hydroxy-4-(2-methoxyphenyl)-1-methyl-1*H*-pyrrole-2,5-dione (1o): This is a new compound, yellow solid, 1.92 g, 86% yield. **^{1}H NMR** (400 MHz, DMSO-d_6) δ 7.42 – 7.34 (m, 1H), 7.30 – 7.20 (m, 1H), 7.10 – 7.03 (m, 1H), 7.02 – 6.95 (m, 1H), 3.76 (s, 3H), 2.91 (s, 3H). **^{13}C NMR** (151 MHz, DMSO-d_6) δ 170.9, 167.0, 157.4, 153.1, 131.1, 129.7, 119.9, 117.0, 111.3, 106.1, 55.4, 23.4. **HRMS** (ESI-TOF) m/z: $[M-H]^-$ Calcd for $C_{12}H_{10}NO_4^-$ = 232.0615; Found 232.0611.

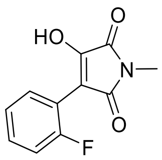

3-(2-fluorophenyl)-4-hydroxy-1-methyl-1*H*-pyrrole-2,5-dione (1p): This is a new compound, yellow solid, 1.41 g, 68% yield. **^{1}H NMR** (600 MHz, DMSO-d_6) δ 7.48 – 7.40 (m, 2H), 7.31 – 7.21 (m, 2H), 2.93 (s, 3H). **^{13}C NMR** (151 MHz, DMSO-d_6) δ 170.3, 166.6, 159.7 (d, J = 248.8 Hz), 154.6, 131.5 (d, J = 3.2 Hz), 130.3 (d, J = 8.1 Hz), 124.1 (d, J = 3.0 Hz), 116.3 (d, J = 16.1 Hz), 115.7 (d, J = 21.4 Hz), 103.0, 23.5. **^{19}F NMR** (376 MHz, DMSO-d_6) δ -109.8. **HRMS** (ESI-TOF) m/z: $[M-H]^-$ Calcd for $C_{11}H_7FNO_3^-$ = 220.0415; Found 220.0410.

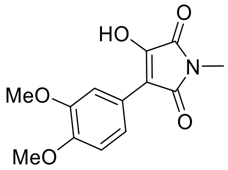

3-(3,4-dimethoxyphenyl)-4-hydroxy-1-methyl-1*H*-pyrrole-2,5-dione (1q)[5]: yellow solid, 2.08 g, 79% yield. **^{1}H NMR** (400 MHz, DMSO-d_6) δ 7.62 (d, J = 1.9 Hz, 1H), 7.58 (dd, J = 8.5, 1.9 Hz, 1H), 7.01 (d, J = 8.5 Hz, 1H), 3.78 (s, 3H), 3.75 (s, 3H), 2.90 (s, 3H). **^{13}C NMR** (151 MHz, DMSO-d_6) δ 171.5, 166.9, 151.4, 148.3, 122.4, 120.7, 111.6, 111.0, 106.2, 55.4, 55.4, 23.2. **HRMS** (ESI-TOF) m/z: $[M-H]^-$ Calcd for $C_{13}H_{12}NO_5^-$ = 262.0721; Found 262.0718.

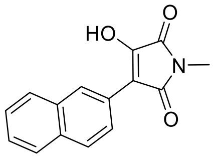

3-hydroxy-1-methyl-4-(naphthalen-2-yl)-1*H*-pyrrole-2,5-dione (1r)[5]: yellow solid, 2.3 g, 91% yield. **^{1}H NMR** (400 MHz, DMSO-d_6) δ 8.48 (s, 1H), 8.12 (dd, J = 8.7, 1.6 Hz, 1H), 7.97 – 7.79 (m, 3H), 7.60 – 7.40 (m, 2H), 2.91 (s, 3H). **^{13}C NMR** (151 MHz, DMSO-d_6) δ 171.3, 166.6, 153.7, 132.8, 131.9, 128.2, 127.6, 127.5, 127.5, 126.5, 126.3, 125.2, 105.8, 23.3. **HRMS** (ESI-TOF) m/z: $[M-H]^-$ Calcd for $C_{15}H_{10}NO_3^-$ = 252.0666; Found 252.0664.

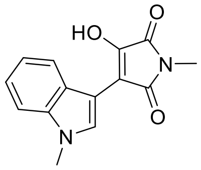

3-hydroxy-1-methyl-4-(1-methyl-1H-indol-3-yl)-1*H*-pyrrole-2,5-dione (1s)[6]: yellow solid, 1.74 g, 68% yield. **^{1}H NMR** (600 MHz, DMSO-d_6) δ 11.93 (br, 1H), 8.14 (d, J = 8.0 Hz, 1H), 7.85 (s, 1H), 7.45 (d, J = 8.1 Hz, 1H), 7.21 (t, J = 7.4 Hz, 1H), 7.09 (t, J = 7.4 Hz, 1H), 3.83 (s, 3H), 2.93 (s, 3H). **^{13}C NMR** (151 MHz, DMSO-d_6) δ 171.6, 167.6, 147.2, 136.5, 130.2, 125.8, 122.4, 121.8, 119.4, 109.9, 105.7, 103.5, 32.7, 23.4. **HRMS** (ESI-TOF) m/z: [M-H]$^-$ Calcd for $C_{14}H_{11}N_2O_3^-$ = 255.0775; Found 255.0771.

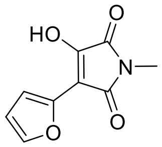

3-(furan-2-yl)-4-hydroxy-1-methyl-1*H*-pyrrole-2,5-dione (1t): This is a new compound, yellow solid, 1.29 g, 67% yield. **^{1}H NMR** (600 MHz, DMSO-d_6) δ 7.81 – 7.71 (m, 1H), 6.80 – 6.72 (m, 1H), 6.61 – 6.52 (m, 1H), 2.87 (s, 3H). **^{13}C NMR** (151 MHz, DMSO-d_6) δ 169.2, 166.8, 150.3, 144.8, 143.1, 111.6, 109.4, 100.1, 23.3. **HRMS** (ESI-TOF) m/z: [M-H]$^-$ Calcd for $C_9H_6NO_4^-$ = 192.0302; Found 192.0295.

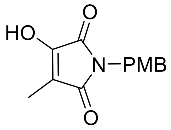

3-hydroxy-1-(4-methoxybenzyl)-4-methyl-1*H*-pyrrole-2,5-dione (1u): This is a new compound, white solid, 1.43 g, 58% yield. **^{1}H NMR** (400 MHz, Chloroform-*d*) δ 7.31 – 7.21 (m, 2H), 6.86 – 6.77 (m, 2H), 4.56 (s, 2H), 3.77 (s, 3H), 1.84 (s, 3H). **^{13}C NMR** (101 MHz, Chloroform-*d*) δ 172.0, 167.9, 159.1, 150.8, 129.8, 128.5, 114.0, 107.7, 55.2, 40.8, 5.8. **HRMS** (ESI-TOF) m/z: [M-H]$^-$ Calcd for $C_{13}H_{12}NO_4^-$ = 246.0772; Found 246.0769.

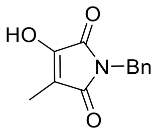

1-benzyl-3-hydroxy-4-methyl-1H-pyrrole-2,5-dione (**1v**): This is a new compound, white solid, 1.35 g, 62% yield. ^{1}**H NMR** (400 MHz, Chloroform-*d*) δ 7.37 – 7.26 (m, 5H), 4.63 (s, 2H), 1.86 (s, 3H). ^{13}**C NMR** (101 MHz, Chloroform-*d*) δ 171.9, 167.9, 150.8, 136.2, 128.7, 128.3, 127.8, 107.7, 41.4, 5.8. **HRMS** (ESI-TOF) m/z: $[M-H]^-$ Calcd for $C_{12}H_{10}NO_3^-$ = 216.0666; Found 216.0660.

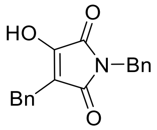

1,3-dibenzyl-4-hydroxy-1H-pyrrole-2,5-dione (**1w**): This is a new compound, white solid, 1.61 g, 55% yield. ^{1}**H NMR** (400 MHz, Chloroform-*d*) δ 7.34 – 7.22 (m, 10H), 4.61 (s, 2H), 3.63 (s, 2H). ^{13}**C NMR** (101 MHz, Chloroform-*d*) δ 171.2, 167.6, 150.7, 137.6, 136.0, 128.7, 128.7, 128.7, 128.4, 127.8, 126.6, 110.4, 41.5, 27.2. **HRMS** (ESI-TOF) m/z: $[M-H]^-$ Calcd for $C_{18}H_{14}NO_3^-$ = 292.0979; Found 292.0979.

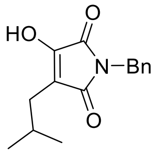

1-benzyl-3-hydroxy-4-isobutyl-1*H*-pyrrole-2,5-dione (**1x**): This is a new compound, white solid, 1.71 g, 66% yield. ^{1}**H NMR** (400 MHz, Chloroform-*d*) δ 7.33 – 7.27 (m, 4H), 7.24 – 7.17 (m, 1H), 4.63 (s, 2H), 2.20 (d, *J* = 7.2 Hz, 2H), 2.03 – 1.87 (m, 1H), 0.93 (s, 3H), 0.91 (s, 3H). ^{13}**C NMR** (101 MHz, Chloroform-*d*) δ 171.8, 167.9, 150.9, 136.2, 128.7, 128.3, 127.8, 111.0, 41.5, 30.2, 27.6, 22.5. **HRMS** (ESI-TOF) m/z: $[M-H]^-$ Calcd for $C_{15}H_{16}NO_3^-$ = 258.1136; Found 258.1133.

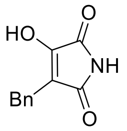

3-benzyl-4-hydroxy-1*H*-pyrrole-2,5-dione (1y)[7]: white solid, 1.52 g, 75% yield. **^{1}H NMR** (600 MHz, DMSO-d_6) δ 12.02 (br, 1H), 10.41 (s, 1H), 7.31 – 7.25 (m, 2H), 7.23 – 7.15 (m, 3H), 3.53 (s, 2H). **^{13}C NMR** (151 MHz, DMSO-d_6) δ 173.1, 168.5, 153.4, 138.9, 128.4, 128.2, 126.1, 109.3, 26.1. **HRMS** (ESI-TOF) m/z: [M-H]$^-$ Calcd for $C_{11}H_8NO_3^-$ = 202.0510; Found 202.0503.

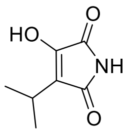

3-hydroxy-4-isopropyl-1H-pyrrole-2,5-dione (1z): This is a new compound, white solid, 1.27 g, 82% yield. **^{1}H NMR** (400 MHz, DMSO-d_6) δ 10.30 (s, 1H), 2.80 – 2.66 (m, 1H), 1.15 (s, 3H), 1.14 (s, 3H). **^{13}C NMR** (101 MHz, DMSO-d_6) δ 173.1, 168.8, 151.8, 115.4, 22.6, 20.7. **HRMS** (ESI-TOF) m/z: [M-H]$^-$ Calcd for $C_7H_8NO_3^-$ = 154.0510; Found 154.0500.

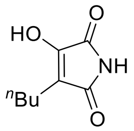

3-butyl-4-hydroxy-1H-pyrrole-2,5-dione (1aa): This is a new compound, white solid, 1.44 g, 85% yield. **^{1}H NMR** (400 MHz, DMSO-d_6) δ 11.56 (br, 1H), 10.28 (s, 1H), 2.16 (t, *J* = 7.4 Hz, 2H), 1.46 – 1.35 (m, 2H), 1.33 – 1.20 (m, 2H), 0.87 (t, *J* = 7.3 Hz, 3H). **^{13}C NMR** (101 MHz, DMSO-d_6) δ 173.3, 168.6, 152.7, 110.7, 29.7, 21.9, 20.1, 13.7. **HRMS** (ESI-TOF) m/z: [M-H]$^-$ Calcd for $C_8H_{10}NO_3^-$ = 168.0666; Found 168.0657.

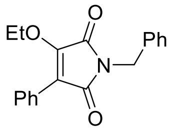

1-benzyl-3-ethoxy-4-phenyl-1H-pyrrole-2,5-dione (1a’):[3] yellow solid, 0.30 g, 50% yield. **^{1}H NMR** (400 MHz, Chloroform-*d*) δ 7.88 (d, *J* = 7.4 Hz, 2H), 7.47 – 7.26 (m, 8H), 4.70 (s, 2H), 4.65 (q, *J* = 7.1 Hz, 2H), 1.40 (t, *J* = 7.1 Hz, 3H). **^{13}C NMR** (101 MHz, Chloroform-*d*) δ 169.9, 165.8, 151.4, 136.4, 128.9, 128.6, 128.5, 128.2, 127.7,

113.1, 69.0, 41.2, 15.7. **HRMS** (ESI-TOF) m/z: $[M+H]^+$ Calcd for $C_{19}H_{18}NO_3^+$ = 308.1281; Found 308.1278.

General Procedure of Asymmetric Transfer Hydrogenation of 1

General Procedure

Procedure E: To a 10 mL Schleck tube charged with a magnetic stirring bar were added successively substrate **1** (0.2 mmol), formic acid/trimethylamine azeotropic mixture (5/2) (40 μL), the catalyst (3 mg) and the solvent (2 mL). The mixture was then stirred at room temperature for the indicated reaction time. After completion, the reaction solution was concentrated and the residue was passed through a short column of silica gel (eluent: EtOAc:PE = 2:1) to remove the metal complex. The ee or dr values of compounds **2a-2t** were determined by HPLC analysis on a chiral stationary phase. The physical data were identical in all respect to those previously reported.

Procedure F: To a 10 mL Schleck tube charged with a magnetic stirring bar were added successively substrate **1** (0.2 mmol), formic acid (15.2 μL), trimethylamine (0.56 μL), the catalyst (3 mg) and the solvent (2 mL). The mixture was then stirred at room temperature for the indicated reaction time. After completion, the reaction solution was concentrated and the residue was passed through a short column of silica gel (eluent: EtOAc:PE = 2:1) to remove the metal complex. The ee or dr values of compounds **3a-3t** were determined by HPLC analysis on a chiral stationary phase. The physical data were identical in all respect to those previously reported.

Procedure G: To a 10 mL Schleck tube charged with a magnetic stirring bar were added successively substrate **1** (0.2 mmol), formic acid/trimethylamine azeotropic mixture (5/2) (80 μL), the catalyst (7.5 mg) and the solvent (1 mL). The mixture was then stirred at room temperature for the indicated reaction time. After completion, the reaction solution was concentrated and the residue was passed through a short column of silica gel (eluent: EtOAc:PE = 1:3) to remove the metal complex. The ee of compounds **4y-4z** were determined by HPLC analysis on a chiral stationary phase. The dr values was determined by ^{1}H NMR of the crude product. The physical data were identical in all respect to those previously reported.

Supplementary Notes: Both procedure A and B were going to obtained compound

3u~3aa. Reaction time control is very important to obtain high diastereoselectivity of 3a~3t.

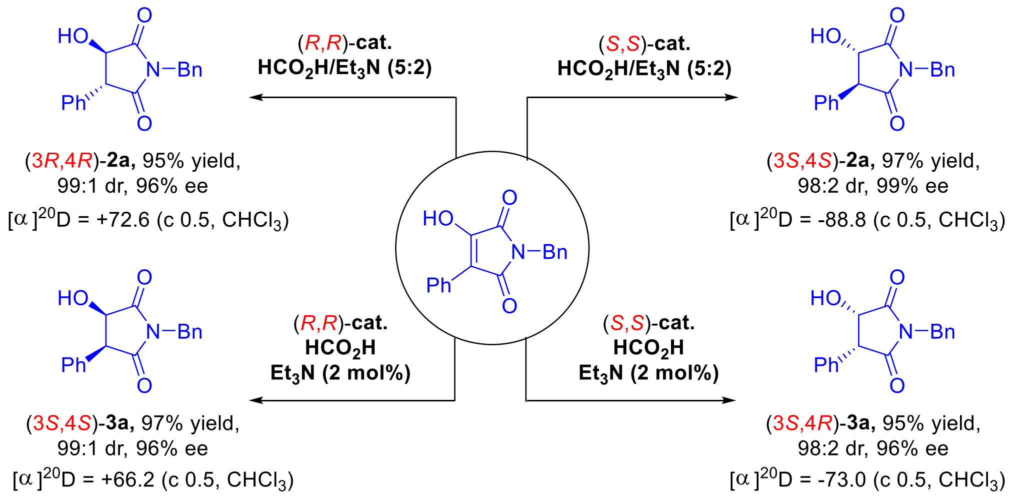

Supplementary Figure 7. Stereodivergent synthesis of 2a and 3a

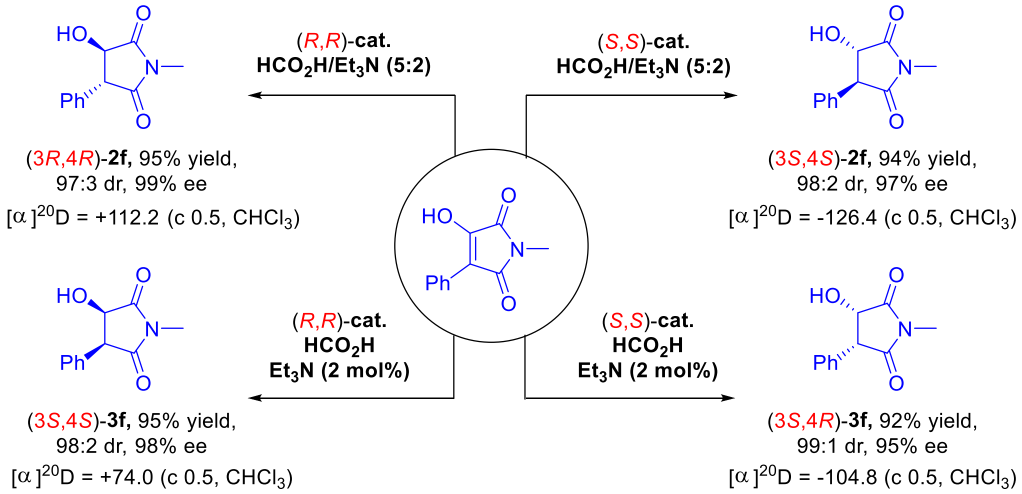

Supplementary Figure 8. Stereodivergent synthesis of 2f and 3f

Characterization Data of 2, 3 and 4

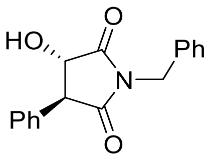

(3*S*,4*S*)-1-benzyl-3-hydroxy-4-phenylpyrrolidine-2,5-dione [(3*S*,4*S*)-2a]: This is a new compound, white solid, 55 mg, 97% yield, 99% ee, 98:2 dr; $[\alpha]^{20}_{D}$ = -88.8 (c 0.5, $CHCl_3$); **HPLC** (Chiralpak IE column, hexane/isopropanol = 80/20; flow rate = 1.0

mL/min; UV detection at 210 nm; t_1 = 8.9 min, t_2 = 9.9 min, t_3 = 10.6 min, t_4 = 13.4 min (major). **^{1}H NMR** (400 MHz, Chloroform-*d*) δ 7.46 – 7.25 (m, 8H), 7.23 – 7.12 (m, 2H), 4.69 (q, *J* = 14.1 Hz, 2H), 4.61 – 4.52 (m, 1H), 3.98 (s, 1H), 3.91 (d, *J* = 6.0 Hz, 1H). **^{13}C NMR** (101 MHz, Chloroform-*d*) δ 176.6, 174.1, 135.2, 134.6, 129.0, 128.7, 128.2, 128.1, 128.0, 74.7, 54.7, 42.7. **HRMS** (ESI-TOF) m/z: $[M-H]^-$ Calcd for $C_{17}H_{14}NO_3^-$ = 280.0979; Found 280.0978.

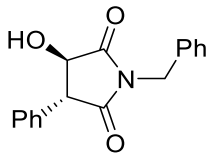

(3*R*,4*R*)-1-benzyl-3-hydroxy-4-phenylpyrrolidine-2,5-dione [(3*R*,4*R*)-2a]: This is a new compound, white solid, 53 mg, 95% yield, 96% ee, 99:1 dr; $[\alpha]^{20}_D$ = +72.6 (c 0.5, $CHCl_3$); **HPLC** (Chiralpak IE column, hexane/isopropanol = 80/20; flow rate = 1.0 mL/min; UV detection at 210 nm; t_1 = 8.9 min (major), t_2 = 9.9 min, t_3 = 10.6 min, t_4 = 13.4 min. **HRMS** (ESI-TOF) m/z: $[M-H]^-$ Calcd for $C_{17}H_{14}NO_3^-$ = 280.0979; Found 280.0978.

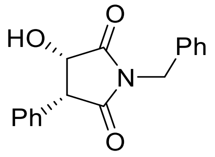

(3*S*,4*R*)-1-benzyl-3-hydroxy-4-phenylpyrrolidine-2,5-dione [(3*S*,4*R*)-3a]: This is a new compound, white solid, 53 mg, 95% yield, 96% ee, 98:2 dr; $[\alpha]^{20}_D$ = -73.0 (c 0.5, $CHCl_3$); **HPLC** (Chiralpak IE column, hexane/isopropanol = 80/20; flow rate = 1.0 mL/min; UV detection at 210 nm; t_1 = 8.9 min, t_2 = 9.9 min (major), t_3 = 10.6 min, t_4 = 13.4 min. **^{1}H NMR** (400 MHz, Chloroform-*d*) δ 7.41 – 7.29 (m, 2H), 7.29 – 7.21 (m, 3H), 7.21 – 7.13 (m, 3H), 6.99 – 6.82 (m, 2H), 4.66 (s, 2H), 4.65 – 4.60 (m, 1H), 4.11 (d, *J* = 8.3 Hz, 1H), 2.82 (d, *J* = 5.0 Hz, 1H). **^{13}C NMR** (151 MHz, Chloroform-*d*) δ 177.1, 175.0, 135.2, 131.4, 129.2, 128.9, 128.8, 128.7, 128.3, 128.2, 69.0, 52.1, 42.7. **HRMS** (ESI-TOF) m/z: $[M-H]^-$ Calcd for $C_{17}H_{14}NO_3^-$ = 280.0979; Found 280.0978.

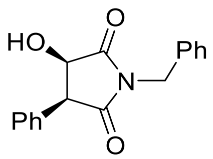

(3*R*,4*S*)-1-benzyl-3-hydroxy-4-phenylpyrrolidine-2,5-dione [(3*R*,4*S*)-3a]: This is a new compound, white solid, 55 mg, 97% yield, 96% ee, 99:1 dr; $[\alpha]^{20}_{D}$ = +66.2 (c 0.5, $CHCl_3$); **HPLC** (Chiralpak IE column, hexane/isopropanol = 80/20; flow rate = 1.0 mL/min; UV detection at 210 nm; t_1 = 8.9 min, t_2 = 9.9 min, t_3 = 10.6 min (major), t_4 = 13.4 min. **HRMS** (ESI-TOF) m/z: $[M-H]^-$ Calcd for $C_{17}H_{14}NO_3^-$ = 280.0979; Found 280.0978.

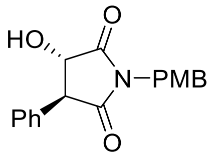

(3*S*,4*S*)-3-hydroxy-1-(4-methoxybenzyl)-4-phenylpyrrolidine-2,5-dione (2b): This is a new compound, white solid, 61 mg, 98% yield, >99% ee, 99:1 dr; $[\alpha]^{20}_{D}$ = -45.6 (c 0.25, $CHCl_3$/MeOH); **HPLC** (Chiralpak IE column, hexane/isopropanol = 80/20; flow rate = 1.0 mL/min; UV detection at 210 nm; t_1 = 13.1 min, t_2 = 14.9 min, t_3 = 17.4 min, t_4 = 21.7 min (major). **^{1}H NMR** (600 MHz, DMSO-d_6) δ 7.40 – 7.29 (m, 5H), 7.24 (d, *J* = 8.1 Hz, 2H), 6.91 (d, *J* = 8.2 Hz, 2H), 6.39 (s, 1H), 4.70 (d, *J* = 5.7 Hz, 1H), 4.62 – 4.50 (m, 2H), 4.01 (d, *J* = 6.4 Hz, 1H), 3.74 (s, 3H). **^{13}C NMR** (151 MHz, DMSO-d_6) δ 176.5, 174.4, 158.7, 135.9, 129.1, 128.8, 128.5, 128.1, 127.5, 113.9, 74.1, 55.2, 55.1, 41.1. **HRMS** (ESI-TOF) m/z: $[M-H]^-$ Calcd for $C_{18}H_{16}NO_4^-$ = 310.1085; Found 310.1085.

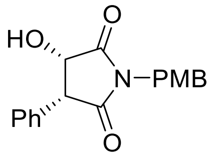

(3*S*,4*R*)-3-hydroxy-1-(4-methoxybenzyl)-4-phenylpyrrolidine-2,5-dione (3b): This is a new compound, white solid, 59 mg, 93% yield, 95% ee, 93:7 dr; $[\alpha]^{20}_{D}$ = -54.4 (c 0.5, $CHCl_3$); **HPLC** (Chiralpak IE column, hexane/isopropanol = 80/20; flow rate = 1.0 mL/min; UV detection at 210 nm; t_1 = 13.1 min, t_2 = 14.7 min (major), t_3 = 17.3 min,

t_4 = 21.6 min. ^{1}H NMR (400 MHz, Chloroform-*d*) δ 7.37 (d, *J* = 8.6 Hz, 2H), 7.32 – 7.27 (m, 3H), 7.04 – 6.95 (m, 2H), 6.85 (d, *J* = 8.6 Hz, 2H), 4.80 – 4.73 (m, 1H), 4.70 (d, *J* = 1.9 Hz, 2H), 4.21 (d, *J* = 8.3 Hz, 1H), 3.79 (s, 3H), 2.51 – 2.39 (m, 1H). **^{13}C NMR** (101 MHz, Chloroform-*d*) δ 176.9, 174.9, 159.5, 131.4, 130.5, 129.2, 129.0, 128.4, 127.6, 114.1, 69.2, 55.3, 52.2, 42.2. **HRMS** (ESI-TOF) m/z: $[M-H]^-$ Calcd for $C_{18}H_{16}NO_4^-$ = 310.1085; Found 310.1086.

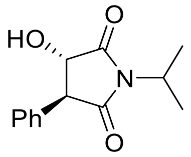

(3*S*,4*S*)-3-hydroxy-1-isopropyl-4-phenylpyrrolidine-2,5-dione (2c): This is a new compound, white solid, 43 mg, 93% yield, 88% ee, 94:6 dr; $[\alpha]^{20}_D$ = -76.0 (c 1.3, $CHCl_3$); **HPLC** (Chiralpak IC column, hexane/isopropanol = 85/15; flow rate = 0.8 mL/min; UV detection at 210 nm; t_1 = 8.5 min, t_2 = 9.7 min (major), t_3 = 10.7 min, t_4 = 12.0 min. **^{1}H NMR** (600 MHz, Chloroform-*d*) δ 7.38 (t, *J* = 7.3 Hz, 2H), 7.33 (t, *J* = 7.2 Hz, 1H), 7.26 (d, *J* = 7.0 Hz, 2H), 4.54 (d, *J* = 4.2 Hz, 1H), 4.44 (p, *J* = 6.9 Hz, 1H), 3.88 (d, *J* = 5.9 Hz, 1H), 3.78 (s, 1H), 1.43 (d, *J* = 6.9 Hz, 6H). **^{13}C NMR** (151 MHz, Chloroform-*d*) δ 177.0, 174.2, 135.0, 129.1, 128.1, 128.0, 74.4, 54.5, 44.4, 19.5, 19.1. **HRMS** (ESI-TOF) m/z: $[M-H]^-$ Calcd for $C_{13}H_{14}NO_3^-$ = 232.0979; Found 232.0975.

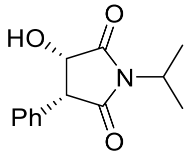

(3*S*,4*R*)-3-hydroxy-1-isopropyl-4-phenylpyrrolidine-2,5-dione (3c): This is a new compound, white solid, 46 mg, 98% yield, 88% ee, 98:2 dr; $[\alpha]^{20}_D$ = -39.6 (c 0.5, $CHCl_3$); **HPLC** (Chiralpak IC column, hexane/isopropanol = 85/15; flow rate = 0.8 mL/min; UV detection at 210 nm; t_1 = 8.5 min, t_2 = 9.7 min, t_3 = 10.7 min (major), t_4 = 12.0 min. **^{1}H NMR** (400 MHz, Chloroform-*d*) δ 7.35 – 7.21 (m, 3H), 7.12 – 6.96 (m, 2H), 4.64 (d, *J* = 7.8 Hz, 1H), 4.47 – 4.32 (m, 1H), 4.09 (d, *J* = 8.5 Hz, 1H), 2.86 (s, 1H), 1.44 – 1.29 (m, 6H). **^{13}C NMR** (101 MHz, Chloroform-*d*) δ 177.7, 175.1, 131.8,

129.1, 128.9, 128.3, 68.8, 52.1, 44.2, 19.5, 19.0. **HRMS** (ESI-TOF) m/z: $[M-H]^-$ Calcd for $C_{13}H_{14}NO_3^-$ = 232.0979; Found 232.0976.

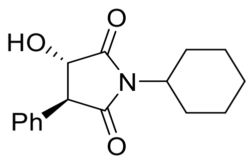

(3*S*,4*S*)-1-cyclohexyl-3-hydroxy-4-phenylpyrrolidine-2,5-dione (2d): This is a new compound, This is a new compound, white solid, 52 mg, 95% yield, 91% ee, 96:4 dr; $[\alpha]^{20}_D$ = -78.2 (c 0.5, $CHCl_3$); **HPLC** (Chiralpak IE column, hexane/isopropanol = 85/15; flow rate = 0.8 mL/min; UV detection at 210 nm; t_1 = 11.9 min, t_2 = 12.9 min, t_3 = 13.7 min, t_4 = 15.0 min (major). **^{1}H NMR** (400 MHz, Chloroform-*d*) δ 7.43 – 7.30 (m, 3H), 7.26 (dd, *J* = 6.5, 1.7 Hz, 2H), 4.54 (dd, *J* = 6.0, 2.6 Hz, 1H), 4.04 (tt, *J* = 12.3, 3.8 Hz, 1H), 3.89 (d, *J* = 6.0 Hz, 1H), 3.44 – 3.35 (m, 1H), 2.29 – 2.08 (m, 2H), 1.93 – 1.76 (m, 2H), 1.73 – 1.61 (m, 3H), 1.44 – 1.14 (m, 3H). **^{13}C NMR** (101 MHz, Chloroform-*d*) δ 176.9, 174.2, 135.1, 129.1, 128.1, 128.0, 74.5, 54.5, 52.2, 29.1, 28.7, 25.7, 25.7, 24.9. **HRMS** (ESI-TOF) m/z: $[M-H]^-$ Calcd for $C_{16}H_{18}NO_3^-$ = 272.1292; Found 272.1292.

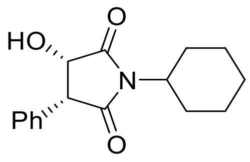

(3*S*,4*R*)-1-cyclohexyl-3-hydroxy-4-phenylpyrrolidine-2,5-dione (3d): This is a new compound, white solid, 52 mg, 95% yield, 96% ee, 99:1 dr; $[\alpha]^{20}_D$ = -60.2 (c 0.5, $CHCl_3$); **HPLC** (Chiralpak IE column, hexane/isopropanol = 85/15; flow rate = 0.8 mL/min; UV detection at 210 nm; t_1 = 11.9 min, t_2 = 12.9 min, t_3 = 13.7 min (major), t_4 = 15.0 min. **^{1}H NMR** (400 MHz, Chloroform-*d*) δ 7.42 – 7.23 (m, 3H), 7.10 – 6.93 (m, 2H), 4.65 (dd, *J* = 8.4, 4.7 Hz, 1H), 4.11 (d, *J* = 8.5 Hz, 1H), 4.07 – 3.93 (m, 1H), 2.60 (d, *J* = 4.4 Hz, 1H), 2.24 – 2.00 (m, 2H), 1.79 (d, *J* = 12.7 Hz, 2H), 1.62 (s, 3H), 1.35 – 1.06 (m, 3H). **^{13}C NMR** (101 MHz, Chloroform-*d*) δ 177.8, 175.1, 131.9, 129.1, 129.0, 128.4, 68.9, 52.1, 29.2, 28.6, 25.8, 25.7, 25.0. **HRMS** (ESI-TOF) m/z: $[M-H]^-$ Calcd for $C_{16}H_{18}NO_3^-$ = 272.1292; Found 272.1292.

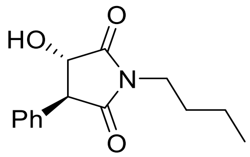

(3*S*,4*S*)-1-butyl-3-hydroxy-4-phenylpyrrolidine-2,5-dione (2e): This is a new compound, white solid, 47 mg, 96% yield, >99% ee, 98:2 dr; $[\alpha]^{20}_{D}$ = -86.4 (c 0.5, $CHCl_3$); **HPLC** (Chiralpak IC column, hexane/isopropanol = 85/15; flow rate = 0.8 mL/min; UV detection at 210 nm; t_1 = 10.3 min, t_2 = 13.1 min, t_3 = 14.8 min (major), t_4 = 15.7 min. **^{1}H NMR** (400 MHz, Chloroform-*d*) δ 7.34 – 7.14 (m, 5H), 4.51 (d, *J* = 5.9 Hz, 1H), 4.10 (s, 1H), 3.85 (d, *J* = 5.9 Hz, 1H), 3.49 (t, *J* = 7.4 Hz, 2H), 1.52 (p, *J* = 7.4 Hz, 2H), 1.33 – 1.13 (m, 2H), 0.86 (t, *J* = 7.3 Hz, 3H). **^{13}C NMR** (101 MHz, Chloroform-*d*) δ 177.2, 174.5, 134.9, 129.0, 128.0, 128.0, 74.7, 54.7, 38.9, 29.6, 20.0, 13.5. **HRMS** (ESI-TOF) m/z: $[M-H]^-$ Calcd for $C_{14}H_{16}NO_3^-$ = 246.1136; Found 246.1132.

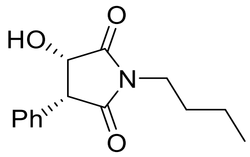

(3*S*,4*R*)-1-butyl-3-hydroxy-4-phenylpyrrolidine-2,5-dione (3e): This is a new compound, white solid, 47 mg, 95% yield, 98% ee, >99:1 dr; $[\alpha]^{20}_{D}$ = -59.6 (c 0.5, $CHCl_3$); **The absolute configurations of 3e was assigned as (*S*,*R*) by X-ray**; **HPLC** (Chiralpak IC column, hexane/isopropanol = 85/15; flow rate = 0.8 mL/min; UV detection at 210 nm; t_1 = 10.3 min, t_2 = 13.1 min (major), t_3 = 14.8 min, t_4 = 15.7 min. **^{1}H NMR** (400 MHz, Chloroform-*d*) δ 7.39 – 7.22 (m, 3H), 7.11 – 6.98 (m, 2H), 4.69 (d, *J* = 8.4 Hz, 1H), 4.13 (d, *J* = 8.4 Hz, 1H), 3.61 – 3.45 (m, 2H), 2.91 (brs, 1H), 1.55 (p, *J* = 7.4 Hz, 2H), 1.37 – 1.21 (m, 2H), 0.87 (t, *J* = 7.4 Hz, 3H). **^{13}C NMR** (151 MHz, Chloroform-*d*) δ 177.6, 175.4, 131.6, 129.3, 128.9, 128.3, 68.9, 52.1, 38.9, 29.7, 20.0, 13.5. **HRMS** (ESI-TOF) m/z: $[M-H]^-$ Calcd for $C_{14}H_{16}NO_3^-$ = 246.1136; Found

246.1133.

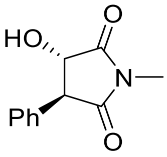

(3*S*,4*S*)-3-hydroxy-1-methyl-4-phenylpyrrolidine-2,5-dione [(3*S*,4*S*)-2f]: This is a new compound, white solid, 38 mg, 94% yield, 97% ee, 96:4 dr; $[\alpha]^{20}_{D}$ = -126.4 (c 0.5, $CHCl_3$); **HPLC** (Chiralpak IC column, hexane/isopropanol = 85/15; flow rate = 1.0 mL/min; UV detection at 210 nm; t_1 = 11.8 min, t_2 = 17.1 min, t_3 = 18.1 min (major), t_4 = 20.2 min. **^{1}H NMR** (400 MHz, Methanol-*d*4) δ 7.40 – 7.34 (m, 2H), 7.34 – 7.27 (m, 3H), 4.62 (d, *J* = 6.2 Hz, 1H), 3.90 (d, *J* = 6.2 Hz, 1H), 3.02 (s, 3H). **^{13}C NMR** (101 MHz, Methanol-*d*4) δ 178.3, 176.8, 137.1, 129.9, 129.6, 128.8, 76.2, 57.0, 25.1. **HRMS** (ESI-TOF) m/z: $[M-H]^-$ Calcd for $C_{11}H_{10}NO_3^-$ = 204.0666; Found 204.0661.

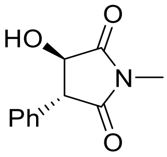

(3*R*,4*R*)-3-hydroxy-1-methyl-4-phenylpyrrolidine-2,5-dione [(3*R*,4*R*)-2f]: This is a new compound, white solid, 39 mg, 95% yield, 99% ee, 97:3 dr; $[\alpha]^{20}_{D}$ = +112.2 (c 0.5, $CHCl_3$); **HPLC** (Chiralpak IC column, hexane/isopropanol = 85/15; flow rate = 1.0 mL/min; UV detection at 210 nm; t_1 = 11.7 min (major), t_2 = 17.1 min, t_3 = 18.3 min, t_4 = 20.0 min. **HRMS** (ESI-TOF) m/z: $[M-H]^-$ Calcd for $C_{11}H_{10}NO_3^-$ = 204.0666; Found 204.0661.

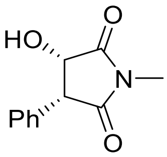

(3*S*,4*R*)-3-hydroxy-1-methyl-4-phenylpyrrolidine-2,5-dione [(3*S*,4*R*)-3f]: This is a new compound, white solid, 38 mg, 92% yield, 99% ee, 99:1 dr; $[\alpha]^{20}_{D}$ = -104.8 (c 0.5, $CHCl_3$); **HPLC** (Chiralpak IC column, hexane/isopropanol = 85/15; flow rate = 1.0 mL/min; UV detection at 210 nm; t_1 = 11.8 min, t_2 = 16.7 min (major), t_3 = 20.1 min,

t_4 = 20.2 min. **^{1}H NMR** (400 MHz, Chloroform-*d*) δ 7.36 – 7.23 (m, 3H), 7.11 – 7.01 (m, 2H), 4.74 (dd, *J* = 8.3, 4.9 Hz, 1H), 4.19 (d, *J* = 8.3 Hz, 1H), 3.07 (s, 3H), 2.40 (d, *J* = 5.0 Hz, 1H). **^{13}C NMR** (101 MHz, Chloroform-*d*) δ 177.2, 175.4, 131.3, 129.4, 129.1, 128.5, 69.1, 52.3, 25.1. **HRMS** (ESI-TOF) m/z: $[M-H]^-$ Calcd for $C_{11}H_{10}NO_3^-$ = 204.0666; Found 204.0659.

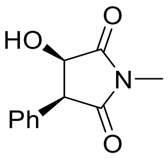

(3*R*,4*S*)-3-hydroxy-1-methyl-4-phenylpyrrolidine-2,5-dione [(3*R*,4*S*)-3f]: This is a new compound, white solid, 39 mg, 95% yield, 98% ee, 98:2 dr; $[\alpha]^{20}_D$ = +74.0 (c 0.5, $CHCl_3$); **HPLC** (Chiralpak IC column, hexane/isopropanol = 85/15; flow rate = 1.0 mL/min; UV detection at 210 nm; t_1 = 11.8 min, t_2 = 17.3 min, t_3 = 18.6 min, t_4 = 20.6 min (major). **HRMS** (ESI-TOF) m/z: $[M-H]^-$ Calcd for $C_{11}H_{10}NO_3^-$ = 204.0666; Found 204.0661.

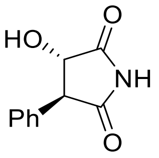

(3*S*,4*S*)-3-hydroxy-4-phenylpyrrolidine-2,5-dione (2g): This is a new compound, white solid, 36 mg, 94% yield, >99% ee, 18:1 dr; $[\alpha]^{20}_D$ = -116.2 (c 0.5, MeOH); **The absolute configurations of 2g was assigned as (*S*,*S*) by X-ray**; **HPLC** (Chiralpak IG column, hexane/isopropanol = 90/10; flow rate = 1.0 mL/min; UV detection at 210 nm; t_1 = 27.8 min (major), t_2 = 34.7 min. **^{1}H NMR** (400 MHz, Methanol-d_4) δ 7.41 – 7.27 (m, 5H), 4.63 (d, *J* = 6.6 Hz, 1H), 3.93 (d, *J* = 6.6 Hz, 1H). **^{13}C NMR** (151 MHz, Methanol-d_4) δ 179.3, 177.5, 137.1, 129.9, 129.6, 128.8, 77.0, 58.1. **HRMS** (ESI-TOF) m/z: $[M-H]^-$ Calcd for $C_{10}H_8NO_3^-$ = 190.0510; Found 190.0502.

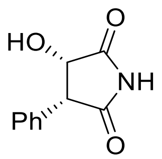

(3*S*,4*R*)-3-hydroxy-4-phenylpyrrolidine-2,5-dione (3g): This is a new compound, colorless oil, 37 mg, 96% yield, 98% ee, >20:1 dr; $[\alpha]^{20}_{D}$ = -50.8 (c 0.5, MeOH); **HPLC** (Chiralpak IC column, hexane/isopropanol = 70/30; flow rate = 1.0 mL/min; UV detection at 210 nm; t_1 = 7.8 min, t_2 = 10.0 min (major). **^{1}H NMR** (400 MHz, Methanol-*d*$_4$) δ 7.38 – 7.25 (m, 3H), 7.21 – 7.14 (m, 2H), 4.78 (d, *J* = 8.2 Hz, 1H), 4.24 (d, *J* = 8.2 Hz, 1H). **^{13}C NMR** (101 MHz, Methanol-*d*$_4$) δ 180.6, 179.4, 134.4, 130.9, 129.4, 128.6, 71.2, 55.1. **HRMS** (ESI-TOF) m/z: $[M-H]^-$ Calcd for $C_{10}H_8NO_3^-$ = 190.0510; Found 190.0503.

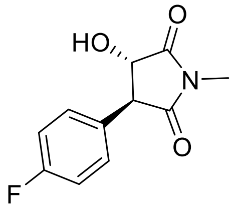

(3*S*,4*S*)-3-(4-fluorophenyl)-4-hydroxy-1-methylpyrrolidine-2,5-dione (2h): This is a new compound, white solid, 43 mg, 96% yield, 99% ee, 96:4 dr; $[\alpha]^{20}_{D}$ = -102.4 (c 0.5, $CHCl_3$); **HPLC** (Chiralpak IC column, hexane/isopropanol = 85/15; flow rate = 1.0 mL/min; UV detection at 210 nm; t_1 = 9.9 min, t_2 = 10.7 min, t_3 = 12.2 min, t_4 = 14.5 min (major). **^{1}H NMR** (400 MHz, Methanol-*d*$_4$) δ 7.40 – 7.30 (m, 2H), 7.15 – 7.03 (m, 2H), 4.61 (d, *J* = 6.4 Hz, 1H), 3.92 (d, *J* = 6.4 Hz, 1H), 3.02 (s, 3H). **^{13}C NMR** (101 MHz, Methanol-*d*$_4$) δ 178.2, 176.5, 163.8 (d, *J* = 244.9 Hz), 133.0 (d, *J* = 3.3 Hz), 131.5 (d, *J* = 8.2 Hz), 116.5 (d, *J* = 21.8 Hz), 76.1, 56.1, 25.1. **^{19}F NMR** (376 MHz, Methanol-*d*$_4$) δ -116.9. **HRMS** (ESI-TOF) m/z: $[M-H]^-$ Calcd for $C_{11}H_9FNO_3^-$ = 222.0572; Found 222.0568.

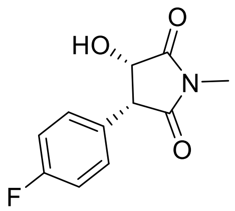

(3*R*,4*S*)-3-(4-fluorophenyl)-4-hydroxy-1-methylpyrrolidine-2,5-dione (3h): This is a new compound, white solid, 43 mg, 97% yield, 98% ee, 99:1 dr; $[\alpha]^{20}_{D}$ = -98.6 (c 0.5, $CHCl_3$); **HPLC** (Chiralpak IC column, hexane/isopropanol = 85/15; flow rate = 1.0 mL/min; UV detection at 210 nm; t_1 = 9.9 min, t_2 = 10.6 min (major), t_3 = 12.1 min, t_4 = 14.7 min. **^{1}H NMR** (400 MHz, Chloroform-*d*) δ 7.17 – 6.95 (m, 4H), 4.78 (d, *J* = 8.3 Hz, 1H), 4.23 (d, *J* = 8.3 Hz, 1H), 3.11 (s, 3H), 3.08 (s, 1H). **^{13}C NMR** (101 MHz, Chloroform-*d*) δ 177.4, 175.4, 162.5 (d, *J* = 247.8 Hz), 131.2 (d, *J* = 8.3 Hz), 127.1 (d, *J* = 3.4 Hz), 115.8 (d, *J* = 21.7 Hz), 68.8, 51.3, 25.1. **^{19}F NMR** (376 MHz, Chloroform-*d*) δ -113.3. **HRMS** (ESI-TOF) m/z: $[M-H]^-$ Calcd for $C_{11}H_9FNO_3^-$ = 222.0572; Found 222.0566.

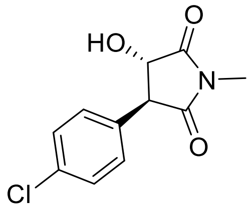

(3*S*,4*S*)-3-(4-chlorophenyl)-4-hydroxy-1-methylpyrrolidine-2,5-dione (2i): This is a new compound, white solid, 46 mg, 95% yield, 98% ee, 96:4 dr; $[\alpha]^{20}_{D}$ = -103.8 (c 0.5, $CHCl_3$); **HPLC** (Chiralpak IC column, hexane/isopropanol = 85/15; flow rate = 1.0 mL/min; UV detection at 210 nm; t_1 = 10.6 min, t_2 = 11.5 min, t_3 = 12.4 min, t_4 = 14.8 min (major). **^{1}H NMR** (600 MHz, Chloroform-*d*) δ 7.41 – 7.34 (m, 2H), 7.29 – 7.23 (m, 2H), 4.60 (d, *J* = 6.0 Hz, 1H), 3.95 (d, *J* = 5.9 Hz, 1H), 3.09 (s, 3H). **^{13}C NMR** (101 MHz, Chloroform-*d*) δ 176.7, 173.9, 134.3, 133.0, 129.4, 129.3, 74.7, 54.0, 25.3. **HRMS** (ESI-TOF) m/z: $[M-H]^-$ Calcd for $C_{11}H_9ClNO_3^-$ = 238.0276; Found 238.0273.

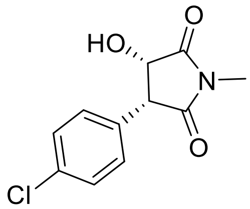

(3*R*,4*S*)-3-(4-chlorophenyl)-4-hydroxy-1-methylpyrrolidine-2,5-dione (2i): This is a new compound, white solid, 47 mg, 98% yield, 96% ee, 99:1 dr; $[\alpha]^{20}_{D}$ = -72.8 (c 0.5,

CHCl3); **HPLC** (Chiralpak IC column, hexane/isopropanol = 85/15; flow rate = 1.0 mL/min; UV detection at 210 nm; t_1 = 10.6 min, t_2 = 11.5 min (major), t_3 = 12.4 min, t_4 = 14.8 min. **^{1}H NMR** (400 MHz, Chloroform-*d*) δ 7.37 – 7.30 (m, 2H), 7.14 – 7.01 (m, 2H), 4.79 (d, *J* = 8.3 Hz, 1H), 4.21 (d, *J* = 8.3 Hz, 1H), 3.11 (s, 3H), 3.04 (s, 1H). **^{13}C NMR** (101 MHz, Chloroform-*d*) δ 177.3, 175.2, 134.4, 130.8, 129.8, 129.0, 68.7, 51.4, 25.1. **HRMS** (ESI-TOF) m/z: $[M-H]^-$ Calcd for $C_{11}H_9ClNO_3^-$ = 238.0276; Found 238.0274.

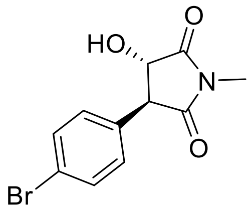

(3*S*,4*S*)-3-(4-bromophenyl)-4-hydroxy-1-methylpyrrolidine-2,5-dione (2j): This is a new compound, white solid, 54 mg, 95% yield, 99% ee, 97:3 dr; $[\alpha]^{20}_D$ = -90.2 (c 0.5, $CHCl_3$); **HPLC** (Chiralpak IC column, hexane/isopropanol = 90/10; flow rate = 1.0 mL/min; UV detection at 210 nm; t_1 = 16.4 min, t_2 = 19.0 min, t_3 = 20.5 min, t_4 = 22.8 min (major). **^{1}H NMR** (400 MHz, Methanol-d_4) δ 7.61 – 7.49 (m, 2H), 7.31 – 7.22 (m, 2H), 4.61 (d, *J* = 6.4 Hz, 1H), 3.91 (d, *J* = 6.4 Hz, 1H), 3.02 (s, 3H). **^{13}C NMR** (101 MHz, Methanol-d_4) δ 178.1, 176.1, 136.3, 132.9, 131.6, 122.7, 75.9, 56.3, 25.1. **HRMS** (ESI-TOF) m/z: $[M-H]^-$ Calcd for $C_{11}H_9BrNO_3^-$ = 281.9771; Found 281.9770.

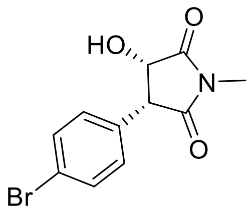

(3*R*,4*S*)-3-(4-bromophenyl)-4-hydroxy-1-methylpyrrolidine-2,5-dione (3j): This is a new compound, white solid, 54 mg, 95% yield, 98% ee, 98:2 dr; $[\alpha]^{20}_D$ = -57.8 (c 0.5, $CHCl_3$); **HPLC** (Chiralpak IC column, hexane/isopropanol = 90/10; flow rate = 1.0 mL/min; UV detection at 210 nm; t_1 = 16.4 min, t_2 = 19.1 min (major), t_3 = 20.5 min, t_4 = 22.8 min. **^{1}H NMR** (600 MHz, Chloroform-*d*) δ 7.41 (d, *J* = 8.2 Hz, 2H), 6.94 (d,

J = 8.2 Hz, 2H), 4.71 (dd, J = 8.2, 4.1 Hz, 1H), 4.12 (d, J = 8.3 Hz, 1H), 3.05 (d, J = 4.7 Hz, 1H), 3.03 (s, 3H). **^{13}C NMR** (151 MHz, Chloroform-*d*) δ 177.3, 175.1, 131.9, 131.2, 130.4, 122.5, 68.7, 51.5, 25.2. **HRMS** (ESI-TOF) m/z: [M-H]$^-$ Calcd for $C_{11}H_9BrNO_3^-$ = 281.9771; Found 281.9770.

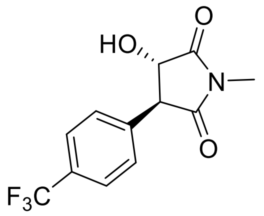

(3*S*,4*S*)-3-hydroxy-1-methyl-4-(4-(trifluoromethyl)phenyl)pyrrolidine-2,5-dione (2k): This is a new compound, white solid, 48 mg, 88% yield, 96% ee, 90:10 dr; $[\alpha]^{20}_{D}$ = -100.3 (c 0.3, MeOH); **HPLC** (Chiralpak IG column, hexane/isopropanol =80/20; flow rate = 1.0 mL/min; UV detection at 210 nm; t_1 = 6.0 min, t_2 = 6.4 min, t_3 = 6.9 min, t_4 = 7.7 min (major). **^{1}H NMR** (600 MHz, Methanol-*d*$_4$) δ 7.68 (d, J = 8.1 Hz, 2H), 7.55 (d, J = 8.1 Hz, 2H), 4.67 (d, J = 6.5 Hz, 1H), 4.05 (d, J = 6.5 Hz, 1H), 3.03 (s, 3H). **^{13}C NMR** (151 MHz, Methanol-*d*$_4$) δ 178.0, 175.9, 141.5, 131.0 (q, J = 32.4 Hz), 130.4, 126.6 (q, J = 3.8 Hz), 125.6 (q, J = 271.8 Hz), 75.8, 56.5, 25.2. **^{19}F NMR** (376 MHz, Methanol-*d*$_4$) δ -64.1. **HRMS** (ESI-TOF) m/z: [M-H]$^-$ Calcd for $C_{12}H_9F_3NO_3^-$ = 272.0540; Found 272.0538.

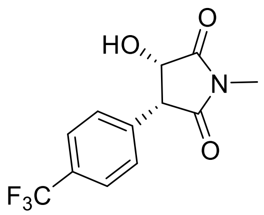

(3*S*,4*R*)-3-hydroxy-1-methyl-4-(4-(trifluoromethyl)phenyl)pyrrolidine-2,5-dione (3k): This is a new compound, white solid, 50 mg, 92% yield, 92% ee, 97:3 dr; $[\alpha]^{20}_{D}$ = -60.2 (c 0.5, $CHCl_3$); **HPLC** (Chiralpak IG column, hexane/isopropanol =80/20; flow rate = 1.0 mL/min; UV detection at 210 nm; t_1 = 6.0 min, t_2 = 6.4 min, t_3 = 6.9 min (major), t_4 = 7.7 min. **^{1}H NMR** (600 MHz, Methanol-*d*$_4$) δ 7.62 (d, J = 8.0 Hz, 2H), 7.36 (d, J = 7.9 Hz, 2H), 4.80 (d, J = 8.1 Hz, 1H), 4.38 (d, J = 8.1 Hz, 1H), 3.06 (s, 3H). **^{13}C NMR** (151 MHz, Methanol-*d*$_4$) δ 178.9, 177.9, 139.0, 131.8, 130.7 (q, J = 32.3 Hz),

126.1 (q, J = 3.8 Hz), 125.7 (q, J = 271.1 Hz), 70.1, 53.3, 25.1. **^{19}F NMR** (565 MHz, Methanol-d_4) δ -64.1. **HRMS** (ESI-TOF) m/z: $[M-H]^-$ Calcd for $C_{12}H_9F_3NO_3^-$ = 272.0540; Found 272.0540.

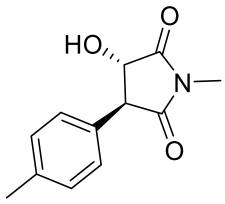

(3*S*,4*S*)-3-hydroxy-1-methyl-4-(p-tolyl)pyrrolidine-2,5-dione (2l): This is a new compound, white solid, 42 mg, 97% yield, >99% ee, 99:1 dr; $[\alpha]^{20}_D$ = -108.0 (c 0.5, $CHCl_3$); **HPLC** (Chiralpak IC column, hexane/isopropanol = 90/10; flow rate = 1.0 mL/min; UV detection at 210 nm; t_1 = 19.7 min, t_2 = 32.2 min (major), t_3 = 35.9 min, t_4 = 40.2 min. **^{1}H NMR** (400 MHz, Methanol-d_4) δ 7.22 – 7.09 (m, 4H), 4.58 (d, J = 6.1 Hz, 1H), 3.84 (d, J = 6.1 Hz, 1H), 3.01 (s, 3H), 2.32 (s, 3H). **^{13}C NMR** (101 MHz, Methanol-d_4) δ 178.4, 177.0, 138.7, 134.0, 130.5, 129.4, 76.2, 56.7, 25.1, 21.1. **HRMS** (ESI-TOF) m/z: $[M-H]^-$ Calcd for $C_{12}H_{12}NO_3^-$ = 218.0823; Found 218.0817.

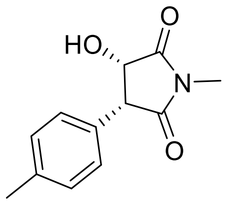

(3*S*,4*R*)-3-hydroxy-1-methyl-4-(p-tolyl)pyrrolidine-2,5-dione (3l): This is a new compound, white solid, 43 mg, 95% yield, >99% ee, 96:4 dr; $[\alpha]^{20}_D$ = -60.8 (c 0.5, $CHCl_3$); **HPLC** (Chiralpak IC column, hexane/isopropanol = 90/10; flow rate = 1.0 mL/min; UV detection at 210 nm; t_1 = 19.7 min, t_2 = 32.2 min, t_3 = 35.9 min (major), t_4 = 40.2 min. **^{1}H NMR** (600 MHz, Chloroform-d) δ 7.10 (d, J = 7.7 Hz, 2H), 6.93 (d, J = 7.8 Hz, 2H), 4.76 – 4.63 (m, 1H), 4.14 (d, J = 8.1 Hz, 1H), 3.04 (s, 3H), 2.69 – 2.57 (m, 1H), 2.26 (s, 3H). **^{13}C NMR** (151 MHz, Chloroform-d) δ 177.4, 175.6, 138.4, 129.7, 129.2, 128.1, 69.0, 51.9, 25.0, 21.1. **HRMS** (ESI-TOF) m/z: $[M-H]^-$ Calcd for $C_{12}H_{12}NO_3^-$ = 218.0823; Found 218.0818.

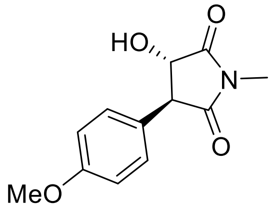

(3*S*,4*S*)-3-hydroxy-4-(4-methoxyphenyl)-1-methylpyrrolidine-2,5-dione (2m): This is a new compound, white solid, 45 mg, 96% yield, >99% ee, 95:5 dr; $[\alpha]^{20}_{D}$ = -115.8 (c 0.5, $CHCl_3$); **HPLC** (Chiralpak IC column, hexane/isopropanol = 85/15; flow rate = 1.0 mL/min; UV detection at 210 nm; t_1 = 25.3 min, t_2 = 31.6 min (major), t_3 = 33.8 min, t_4 = 39.1 min. **^{1}H NMR** (600 MHz, Methanol-*d*$_4$) δ 7.21 (d, *J* = 8.2 Hz, 2H), 6.92 (d, *J* = 8.3 Hz, 2H), 4.57 (d, *J* = 6.0 Hz, 1H), 3.83 (d, *J* = 5.9 Hz, 1H), 3.78 (s, 3H), 3.01 (s, 3H). **^{13}C NMR** (151 MHz, Methanol-*d*$_4$) δ 178.4, 177.1, 160.8, 130.6, 128.9, 115.3, 76.2, 56.3, 55.7, 25.1. **HRMS** (ESI-TOF) m/z: $[M-H]^-$ Calcd for $C_{12}H_{12}NO_4^-$ = 234.0772; Found 234.0767.

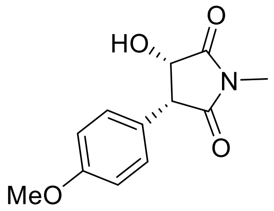

(3*S*,4*R*)-3-hydroxy-4-(4-methoxyphenyl)-1-methylpyrrolidine-2,5-dione (3m): This is a new compound, white solid, 44 mg, 95% yield, >99% ee, 95:5 dr; $[\alpha]^{20}_{D}$ = -49.6 (c 0.5, $CHCl_3$); **HPLC** (Chiralpak IC column, hexane/isopropanol = 85/15; flow rate = 1.0 mL/min; UV detection at 210 nm; t_1 = 25.3 min, t_2 = 31.6 min, t_3 = 33.8 min, t_4 = 39.1 min (major). **^{1}H NMR** (400 MHz, Methanol-*d*$_4$) δ 7.10 – 7.01 (m, 2H), 6.92 – 6.82 (m, 2H), 4.73 (d, *J* = 8.1 Hz, 1H), 4.18 (d, *J* = 8.1 Hz, 1H), 3.76 (s, 3H). **^{13}C NMR** (101 MHz, Methanol-*d*$_4$) δ 179.4, 178.8, 160.6, 132.0, 126.2, 114.8, 70.2, 55.7, 53.1, 25.0. **HRMS** (ESI-TOF) m/z: $[M-H]^-$ Calcd for $C_{12}H_{12}NO_4^-$ = 234.0772; Found 234.0769.

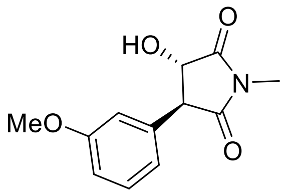

(3*S*,4*S*)-3-hydroxy-4-(3-methoxyphenyl)-1-methylpyrrolidine-2,5-dione (2n): This is a new compound, white solid, 44 mg, 94% yield, 98% ee, 96:4 dr; $[\alpha]^{20}_{D}$ = -89.0 (c 0.5, $CHCl_3$); **HPLC** (Chiralpak IE column, hexane/isopropanol = 70/30; flow rate = 1.0 mL/min; UV detection at 210 nm; t_1 = 19.7 min, t_2 = 26.2 min (major), t_3 = 29.1 min, t_4 = 33.2 min. **^{1}H NMR** (600 MHz, Chloroform-*d*) δ 7.27 – 7.15 (m, 1H), 6.82 – 6.71 (m, 3H), 4.55 (d, *J* = 5.9 Hz, 1H), 3.85 (br, 1H), 3.83 (d, *J* = 5.8 Hz, 1H), 3.73 (s, 3H), 3.00 (s, 3H). **^{13}C NMR** (151 MHz, Chloroform-*d*) δ 177.0, 174.4, 160.0, 136.1, 130.2, 120.1, 114.0, 113.4, 74.8, 55.3, 54.8, 25.2. **HRMS** (ESI-TOF) m/z: $[M-H]^-$ Calcd for $C_{12}H_{12}NO_4^-$ = 234.0772; Found 234.0768.

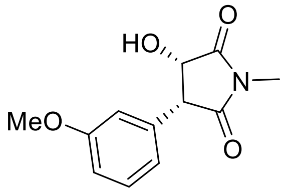

(3*S*,4*R*)-3-hydroxy-4-(3-methoxyphenyl)-1-methylpyrrolidine-2,5-dione (3n): This is a new compound, white solid, 46 mg, 97% yield, 94% ee, 98:2 dr; $[\alpha]^{20}_{D}$ = -31.6 (c 0.5, $CHCl_3$); **HPLC** (Chiralpak IE column, hexane/isopropanol = 70/30; flow rate = 1.0 mL/min; UV detection at 210 nm; t_1 = 19.7 min, t_2 = 26.2 min, t_3 = 29.1 min, t_4 = 33.2 min (major). **^{1}H NMR** (600 MHz, Chloroform-*d*) δ 7.26 – 7.14 (m, 1H), 6.84 – 6.74 (m, 1H), 6.67 – 6.53 (m, 2H), 4.70 (d, *J* = 8.3 Hz, 1H), 4.13 (d, *J* = 8.3 Hz, 1H), 3.71 (s, 3H), 3.04 (s, 3H), 2.77 (brs, 1H). **^{13}C NMR** (151 MHz, Chloroform-*d*) δ 177.3, 175.3, 159.9, 132.7, 130.0, 121.3, 115.6, 113.5, 69.0, 55.2, 52.2, 25.0. **HRMS** (ESI-TOF) m/z: $[M-H]^-$ Calcd for $C_{12}H_{12}NO_4^-$ = 234.0772; Found 234.0769.

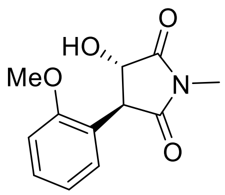

(3*S*,4*S*)-3-hydroxy-4-(2-methoxyphenyl)-1-methylpyrrolidine-2,5-dione (2o): This is a new compound, white solid, 45 mg, 95% yield, 97% ee, 91:9 dr; $[\alpha]^{20}_{D}$ = -107.0 (c 0.5, $CHCl_3$); **HPLC** (Chiralpak IC column, hexane/isopropanol = 60/40; flow rate = 1.0 mL/min; UV detection at 210 nm; t_1 = 6.5 min, t_2 = 7.9 min, t_3 = 9.1 min, t_4 = 10.6 min (major). **^{1}H NMR** (600 MHz, Chloroform-*d*) δ 7.35 – 7.30 (m, 1H), 7.26 – 7.21 (m, 1H), 7.00 – 6.94 (m, 1H), 6.92 – 6.86 (m, 1H), 4.69 (s, 1H), 3.75 (d, *J* = 4.7 Hz, 1H), 3.73 (s, 3H), 3.08 (s, 3H), 1.84 (brs, 1H). **^{13}C NMR** (151 MHz, Chloroform-*d*) δ 177.5, 175.4, 156.7, 131.8, 129.8, 123.7, 121.2, 111.1, 73.5, 55.5, 53.4, 25.0. **HRMS** (ESI-TOF) m/z: $[M-H]^-$ Calcd for $C_{12}H_{12}NO_4^-$ = 234.0772; Found 234.0767.

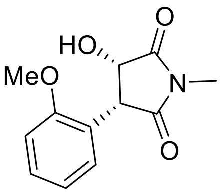

(3*S*,4*R*)-3-hydroxy-4-(2-methoxyphenyl)-1-methylpyrrolidine-2,5-dione (3o): This is a new compound, white solid, 45 mg, 95% yield, 93% ee, 99:1 dr; $[\alpha]^{20}_{D}$ = +6.0 (c 0.5, $CHCl_3$); **HPLC** (Chiralpak IC column, hexane/isopropanol = 60/40; flow rate = 1.0 mL/min; UV detection at 210 nm; t_1 = 6.5 min, t_2 = 7.9 min (major), t_3 = 9.1 min, t_4 = 10.6 min. **^{1}H NMR** (600 MHz, Chloroform-*d*) δ 7.29 – 7.22 (m, 1H), 7.15 – 7.10 (m, 1H), 6.95 – 6.89 (m, 1H), 6.84 – 6.79 (m, 1H), 4.69 (t, *J* = 8.1 Hz, 1H), 4.14 (d, *J* = 8.9 Hz, 1H), 3.66 (s, 3H), 3.01 (s, 3H), 2.73 (d, *J* = 7.4 Hz, 1H). **^{13}C NMR** (151 MHz, Chloroform-*d*) δ 177.9, 176.1, 157.1, 132.6, 129.9, 121.7, 120.8, 111.1, 68.7, 55.7, 49.8, 24.8. **HRMS** (ESI-TOF) m/z: $[M-H]^-$ Calcd for $C_{12}H_{12}NO_4^-$ = 234.0772; Found 234.0769.

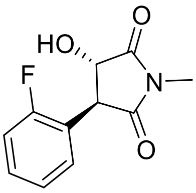

(3*S*,4*S*)-3-(2-fluorophenyl)-4-hydroxy-1-methylpyrrolidine-2,5-dione (2p): This is a new compound, white solid, 43 mg, 96% yield, 97% ee, 91:9 dr; $[\alpha]^{20}_{D}$ = -143.0 (c

0.5, $CHCl_3$); **The absolute configurations of 2p was assigned as (*S*,*S*) by X-ray**; **HPLC** (Chiralpak IB column, hexane/isopropanol = 85/15; flow rate = 1.0 mL/min; UV detection at 210 nm; t_1 = 10.9 min, t_2 = 12.2 min (major), t_3 = 16.8 min, t_4 = 18.1 min. **^{1}H NMR** (600 MHz, Chloroform-*d*) δ 7.39 – 7.24 (m, 2H), 7.20 – 7.06 (m, 2H), 4.69 (t, *J* = 4.4 Hz, 1H), 4.09 (s, 1H), 3.94 (d, *J* = 5.4 Hz, 1H), 3.08 (s, 3H). **^{13}C NMR** (151 MHz, Chloroform-*d*) δ 177.0, 173.9, 160.7 (d, *J* = 246.2 Hz), 131.6 (d, *J* = 3.4 Hz), 130.3 (d, *J* = 8.4 Hz), 124.7, 122.4 (d, *J* = 14.0 Hz), 115.9 (d, *J* = 20.8 Hz), 74.0, 51.6, 25.2. **^{19}F NMR** (565 MHz, Chloroform-*d*) δ -115.7. **HRMS** (ESI-TOF) m/z: $[M-H]^-$ Calcd for $C_{11}H_9FNO_3^-$ = 222.0572; Found 222.0567.

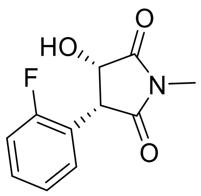

(3*S*,4*R*)-3-(2-fluorophenyl)-4-hydroxy-1-methylpyrrolidine-2,5-dione (3p): This is a new compound, white solid, 43 mg, 97% yield, 94% ee, 99:1 dr; $[\alpha]^{20}_D$ = -80.2 (c 0.5, $CHCl_3$); **HPLC** (Chiralpak IB column, hexane/isopropanol = 85/15; flow rate = 1.0 mL/min; UV detection at 210 nm; t_1 = 10.9 min, t_2 = 12.2 min, t_3 = 16.8 min (major), t_4 = 18.1 min. **^{1}H NMR** (400 MHz, Methanol-*d*$_4$) δ 7.38 – 7.28 (m, 1H), 7.26 – 7.18 (m, 1H), 7.18 – 7.11 (m, 1H), 7.11 – 7.02 (m, 1H), 4.78 (d, *J* = 8.4 Hz, 1H), 4.41 (d, *J* = 8.4 Hz, 1H), 3.04 (s, 3H). **^{13}C NMR** (101 MHz, Methanol-*d*$_4$) δ 179.2, 178.0, 163.0 (d, *J* = 245.3 Hz), 133.3 (d, *J* = 4.1 Hz), 130.8 (d, *J* = 8.5 Hz), 125.2 (d, *J* = 3.4 Hz), 122.0 (d, *J* = 15.1 Hz), 116.1 (d, *J* = 22.1 Hz), 69.3, 25.1. **^{19}F NMR** (376 MHz, Methanol-*d*$_4$) δ -116.2. **HRMS** (ESI-TOF) m/z: $[M-H]^-$ Calcd for $C_{11}H_9FNO_3^-$ = 222.0572; Found 222.0568.

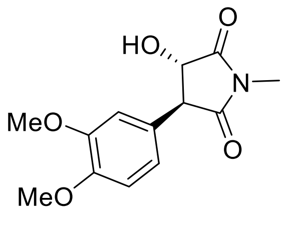

(3*S*,4*S*)-3-(3,4-dimethoxyphenyl)-4-hydroxy-1-methylpyrrolidine-2,5-dione (2q): This is a new compound, white solid, 50 mg, 95% yield, 99% ee, 99:1 dr; $[\alpha]^{20}_{D}$ = -107.2 (c 0.5, $CHCl_3$); **HPLC** (Chiralpak IE column, hexane/isopropanol = 70/30; flow rate = 1.0 mL/min; UV detection at 210 nm; t_1 = 19.7 min, t_2 = 26.2 min (major), t_3 = 29.1min, t_4 = 33.2 min. **^{1}H NMR** (400 MHz, DMSO-d_6) δ 6.97 – 6.89 (m, 2H), 6.86 – 6.80 (m, 1H), 4.60 (d, *J* = 6.4 Hz, 1H), 3.83 (d, *J* = 6.5 Hz, 1H), 3.74 (s, 3H), 3.73 (s, 3H), 3.51 (br, 1H), 2.90 (s, 3H). **^{13}C NMR** (151 MHz, DMSO-d_6) δ 177.3, 175.4, 149.3, 148.7, 128.9, 121.7, 112.8, 112.2, 74.8, 56.0, 56.0, 55.3, 25.0. **HRMS** (ESI-TOF) m/z: $[M-H]^-$ Calcd for $C_{13}H_{14}NO_5^-$ = 264.0877; Found 264.0876.

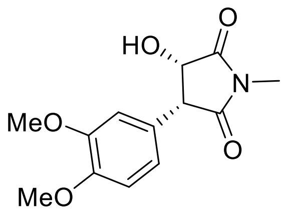

(3*R*,4*S*)-3-(3,4-dimethoxyphenyl)-4-hydroxy-1-methylpyrrolidine-2,5-dione (3q): This is a new compound, white solid, 51 mg, 97% yield, 96% ee, 98:2 dr; $[\alpha]^{20}_{D}$ = -48.2 (c 0.5, $CHCl_3$); **HPLC** (Chiralpak IE column, hexane/isopropanol = 70/30; flow rate = 1.0 mL/min; UV detection at 210 nm; t_1 = 19.7 min, t_2 = 26.2 min, t_3 = 29.1min, t_4 = 33.2 min (major). **^{1}H NMR** (400 MHz, Chloroform-*d*) δ 6.92 – 6.78 (m, 1H), 6.70 – 6.61 (m, 2H), 4.77 (d, *J* = 7.2 Hz, 1H), 4.19 (d, *J* = 8.2 Hz, 1H), 3.86 (s, 3H), 3.85 (s, 3H), 3.12 (s, 3H), 2.70 (s, 1H). **^{13}C NMR** (151 MHz, Chloroform-*d*) δ 177.2, 175.5, 149.2, 149.2, 123.3, 121.4, 112.8, 111.4, 69.0, 55.9, 55.9, 51.8, 25.0. **HRMS** (ESI-TOF) m/z: $[M-H]^-$ Calcd for $C_{13}H_{14}NO_5^-$ = 264.0877; Found 264.0877.

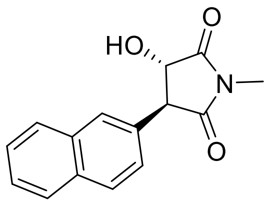

(3*S*,4*S*)-3-hydroxy-1-methyl-4-(naphthalen-2-yl)pyrrolidine-2,5-dione (2r): This is a new compound, white solid, 50 mg, 97% yield, >99% ee, >99:1 dr; $[\alpha]^{20}_{D}$ = -110.0 (c

0.5, $CHCl_3$); **HPLC** (Chiralpak IC column, hexane/isopropanol = 85/15; flow rate = 1.0 mL/min; UV detection at 210 nm; t_1 = 17.5 min, t_2 = 26.1 min, t_3 = 28.9min (major), t_4 = 30.7 min. **^{1}H NMR** (400 MHz, DMSO-d_6) δ 8.00 – 7.82 (m, 4H), 7.61 – 7.42 (m, 3H), 4.73 (d, *J* = 6.5 Hz, 1H), 4.13 (d, *J* = 6.5 Hz, 1H), 2.95 (s, 3H). **^{13}C NMR** (151 MHz, DMSO-d_6) δ 177.2, 175.2, 134.1, 133.3, 132.7, 128.6, 128.4, 128.0, 128.0, 127.0, 126.8, 126.6, 74.7, 55.8, 25.1. **HRMS** (ESI-TOF) m/z: [M-H]$^-$ Calcd for $C_{15}H_{12}NO_3^-$ = 254.0823; Found 254.0821.

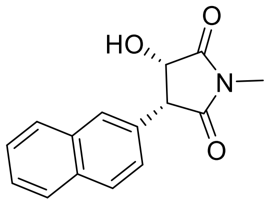

(3*S*,4*R*)-3-hydroxy-1-methyl-4-(naphthalen-2-yl)pyrrolidine-2,5-dione (3r): This is a new compound, white solid, 50 mg, 98% yield, 95% ee, >99:1 dr; $[\alpha]^{20}_D$ = -46.0 (c 0.5, $CHCl_3$); **HPLC** (Chiralpak IC column, hexane/isopropanol = 85/15; flow rate = 1.0 mL/min; UV detection at 210 nm; t_1 = 17.5 min, t_2 = 25.1 min (major), t_3 = 28.9min, t_4 = 30.7 min. **^{1}H NMR** (400 MHz, Chloroform-*d*) δ 7.89 – 7.75 (m, 3H), 7.64 (s, 1H), 7.54 – 7.44 (m, 2H), 7.18 (dd, *J* = 8.5, 1.6 Hz, 1H), 4.87 (dd, *J* = 8.3, 4.6 Hz, 1H), 4.42 (d, *J* = 8.3 Hz, 1H), 3.18 (s, 3H), 2.46 (d, *J* = 4.7 Hz, 1H). **^{13}C NMR** (151 MHz, Chloroform-*d*) δ 177.3, 175.4, 133.3, 132.9, 128.9, 128.7, 128.7, 127.8, 127.7, 126.6, 126.6, 126.5, 69.1, 52.3, 25.1. **HRMS** (ESI-TOF) m/z: [M-H]$^-$ Calcd for $C_{15}H_{12}NO_3^-$ = 254.0823; Found 254.0821.

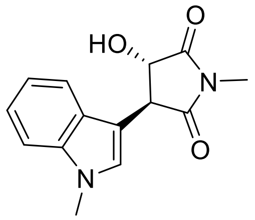

(3*S*,4*S*)-3-hydroxy-1-methyl-4-(1-methyl-1H-indol-3-yl)pyrrolidine-2,5-dione (2s): This is a new compound, white solid, 68 mg, 92% yield, 97% ee, 84:16 dr; $[\alpha]^{20}_D$ = +6.4 (c 0.25, $CHCl_3$); **HPLC** (Chiralpak IC column, hexane/isopropanol = 60/40; flow rate = 1.0 mL/min; UV detection at 210 nm; t_1 = 7.1 min, t_2 = 7.9 min, t_3 = 8.4 min

(major), t_4 = 10.4 min. **^{1}H NMR** (400 MHz, DMSO-d_6) δ 7.54 – 7.39 (m, 2H), 7.33 (s, 1H), 7.18 (t, *J* = 7.5 Hz, 1H), 7.02 (t, *J* = 7.4 Hz, 1H), 6.36 (d, *J* = 6.8 Hz, 1H), 4.60 (t, *J* = 6.4 Hz, 1H), 4.13 (d, *J* = 6.1 Hz, 1H), 3.77 (s, 3H), 2.95 (s, 3H). **^{13}C NMR** (151 MHz, DMSO-d_6) δ 177.4, 175.4, 137.2, 128.9, 127.1, 121.9, 119.4, 119.3, 110.3, 108.5, 74.1, 47.7, 32.9, 25.0. **HRMS** (ESI-TOF) m/z: $[M-H]^-$ Calcd for $C_{14}H_{13}N_2O_3^-$ = 257.0932; Found 257.0930.

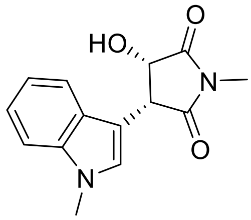

(3*S*,4*R*)-3-hydroxy-1-methyl-4-(1-methyl-1H-indol-3-yl)pyrrolidine-2,5-dione (3s): This is a new compound, white solid, 68 mg, 96% yield, 91% ee, 99:1 dr; $[\alpha]^{20}_D$ = -70.2 (c 0.5, $CHCl_3$); **HPLC** (Chiralpak IC column, hexane/isopropanol = 60/40; flow rate = 1.0 mL/min; UV detection at 210 nm; t_1 = 7.1 min, t_2 = 7.9 min, t_3 = 8.4 min, t_4 = 10.4 min (major). **^{1}H NMR** (600 MHz, Chloroform-*d*) δ 7.38 (d, *J* = 8.0 Hz, 1H), 7.33 (d, *J* = 8.2 Hz, 1H), 7.28 – 7.23 (m, 1H), 7.14 (t, *J* = 7.5 Hz, 1H), 7.04 (s, 1H), 4.80 (d, *J* = 7.9 Hz, 1H), 4.52 (d, *J* = 8.0 Hz, 1H), 3.77 (s, 3H), 3.14 (s, 3H), 2.44 (s, 1H). **^{13}C NMR** (151 MHz, Chloroform-*d*) δ 177.0, 175.9, 137.1, 129.1, 127.1, 122.5, 120.1, 118.6, 109.8, 103.0, 68.8, 44.5, 32.9, 25.0. **HRMS** (ESI-TOF) m/z: $[M-H]^-$ Calcd for $C_{14}H_{13}N_2O_3^-$ = 257.0932; Found 257.0931.

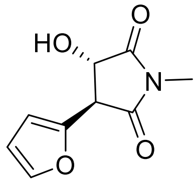

(3*R*,4*S*)-3-(furan-2-yl)-4-hydroxy-1-methylpyrrolidine-2,5-dione (2t): This is a new compound, white solid, 36 mg, 93% yield, 98% ee, 98:2 dr; $[\alpha]^{20}_D$ = -144.2 (c 0.5, $CHCl_3$); **HPLC** (Chiralpak IC column, hexane/isopropanol = 70/30; flow rate = 1.0 mL/min; UV detection at 210 nm; t_1 = 7.2 min, t_2 = 8.1 min, t_3 = 8.7 min (major), t_4 = 9.7 min. **^{1}H NMR** (400 MHz, Chloroform-*d*) δ 7.46 – 7.36 (m, 1H), 6.48 – 6.30 (m,

2H), 4.82 (s, 1H), 4.07 (d, J = 5.8 Hz, 1H), 4.00 (s, 1H), 3.07 (s, 3H). **^{13}C NMR** (101 MHz, Chloroform-*d*) δ 176.7, 172.1, 146.7, 143.0, 110.7, 109.6, 72.0, 49.0, 25.2. **HRMS** (ESI-TOF) m/z: $[M-H]^-$ Calcd for $C_9H_8NO_4^-$ = 194.0459; Found 194.0452.

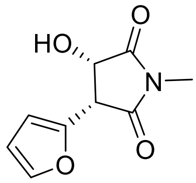

(3*S*,4*S*)-3-(furan-2-yl)-4-hydroxy-1-methylpyrrolidine-2,5-dione (3t): This is a new compound, white solid, 37 mg, 95% yield, 94% ee, 96:4 dr; $[\alpha]^{20}_D$ = -53.6 (c 0.5, $CHCl_3$); **HPLC** (Chiralpak IC column, hexane/isopropanol = 70/30; flow rate = 1.0 mL/min; UV detection at 210 nm; t_1 = 7.2 min, t_2 = 8.1 min (major), t_3 = 8.7 min, t_4 = 9.7 min. **^{1}H NMR** (400 MHz, Chloroform-*d*) δ 7.37 – 7.26 (m, 1H), 6.35 – 6.24 (m, 2H), 4.78 – 4.66 (m, 1H), 4.32 (d, J = 8.3 Hz, 1H), 3.04 (s, 3H), 2.97 – 2.87 (m, 1H). **^{13}C NMR** (101 MHz, Chloroform-*d*) δ 176.8, 172.7, 145.0, 143.3, 110.9, 110.8, 68.8, 46.4, 25.2. **HRMS** (ESI-TOF) m/z: $[M-H]^-$ Calcd for $C_9H_8NO_4^-$ = 194.0459; Found 194.0452.

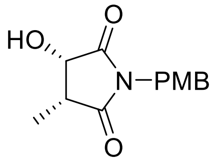

(3*S*,4*R*)-3-hydroxy-1-(4-methoxybenzyl)-4-methylpyrrolidine-2,5-dione (3u)[8]: white solid, 48 mg, 97% yield, 97% ee, >20:1 dr; {$[\alpha]^{20}_D$ = -57.0 (c 0.5, $CHCl_3$); **lit.**9 $[\alpha]^{28}_D$ = -45.5 (*c* 1.0, $CHCl_3$) } **HPLC** (Chiralpak IC column, hexane/isopropanol = 85/15; flow rate = 1.0 mL/min; UV detection at 210 nm; t_1 = 11.5 min (major), t_2 = 12.5 min. **^{1}H NMR** (400 MHz, Chloroform-*d*) δ 7.25 – 7.17 (m, 2H), 6.79 – 6.71 (m, 2H), 4.50 (s, 2H), 4.48 (s, 1H), 3.70 (s, 3H), 3.65 (s, 1H), 2.92 (p, J = 7.7 Hz, 1H), 1.17 (d, J = 7.6 Hz, 3H). **^{13}C NMR** (101 MHz, Chloroform-*d*) δ 178.4, 178.2, 159.3, 130.1, 127.6, 114.0, 68.5, 55.2, 41.7, 40.1, 10.2. **HRMS** (ESI-TOF) m/z: $[M-H]^-$ Calcd for $C_{13}H_{14}NO_4^-$ = 248.0928; Found 248.0926.

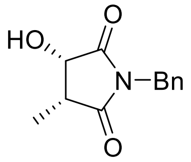

(3*S*,4*R*)-1-benzyl-3-hydroxy-4-methylpyrrolidine-2,5-dione (3v)[10]: white solid, 42 mg, 95% yield, 98% ee, >20:1 dr; $[\alpha]^{20}_{D}$ = -40.2 (c 1.0, $CHCl_3$); lit. $[\alpha]^{20}_{D}$ = -54.2 (c 0.38, $CHCl_3$) **HPLC** (Chiralpak ID column, hexane/isopropanol = 85/15; flow rate = 0.8 mL/min; UV detection at 210 nm; t_1 = 10.9 min, t_2 = 11.5 min (major). **^{1}H NMR** (400 MHz, Chloroform-*d*) δ 7.30 – 7.15 (m, 5H), 4.56 (s, 2H), 4.51 (d, *J* = 8.1 Hz, 1H), 3.70 (s, 1H), 2.93 (p, *J* = 7.7 Hz, 1H), 1.18 (d, *J* = 7.6 Hz, 3H). **^{13}C NMR** (101 MHz, Chloroform-*d*) δ 178.3, 178.3, 135.3, 128.7, 128.5, 128.0, 68.5, 42.2, 40.1, 10.3. **HRMS** (ESI-TOF) m/z: $[M-H]^-$ Calcd for $C_{12}H_{12}NO_3^-$ = 218.0823; Found 218.0817.

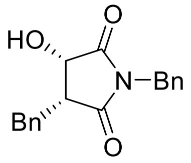

(3*R*,4*S*)-1,3-dibenzyl-4-hydroxypyrrolidine-2,5-dione (3w)[8]: white solid, 56 mg, 95% yield, 99% ee, >20:1 dr; $[\alpha]^{20}_{D}$ = -37.3 (c 1.5, $CHCl_3$); **HPLC** (Chiralpak IC column, hexane/isopropanol = 85/15; flow rate = 0.8 mL/min; UV detection at 210 nm; t_1 = 8.3 min (major), t_2 = 8.8 min. **^{1}H NMR** (600 MHz, Chloroform-*d*) δ 7.32 – 7.25 (m, 5H), 7.23 – 7.21 (m, 4H), 7.20 – 7.15 (m, 1H), 4.59 – 4.50 (m, 2H), 4.50 – 4.47 (m, 1H), 3.62 (d, *J* = 3.1 Hz, 1H), 3.22 – 3.16 (m, 1H), 3.14 – 3.06 (m, 2H). **^{13}C NMR** (151 MHz, Chloroform-*d*) δ 177.6, 176.6, 138.0, 135.2, 129.1, 128.7, 128.6, 128.4, 128.0, 126.6, 68.1, 46.9, 42.2, 30.4. **HRMS** (ESI-TOF) m/z: $[M-H]^-$ Calcd for $C_{18}H_{16}NO_3^-$ = 294.1136; Found 294.1136.

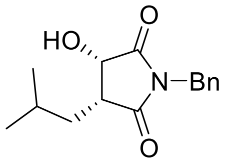

(3*S*,4*R*)-1-benzyl-3-hydroxy-4-isobutylpyrrolidine-2,5-dione (3x): This is a new compound, white solid, 50 mg, 95% yield, 98% ee, >20:1 dr; $[\alpha]^{20}_D$ = -39.0 (c 0.8, $CHCl_3$); **HPLC** (Chiralpak IC column, hexane/isopropanol = 90/10; flow rate = 1.0 mL/min; UV detection at 210 nm; t_1 = 6.9 min (major), t_2 = 7.3 min. **^{1}H NMR** (400 MHz, Chloroform-*d*) δ 7.50 – 7.11 (m, 5H), 4.62 (s, 2H), 4.60 – 4.53 (m, 1H), 3.75 (d, *J* = 3.9 Hz, 1H), 2.94 (q, *J* = 7.9 Hz, 1H), 1.94 – 1.78 (m, 1H), 1.78 – 1.64 (m, 1H), 1.56 – 1.38 (m, 1H), 0.94 (dd, *J* = 10.6, 6.6 Hz, 6H). **^{13}C NMR** (101 MHz, Chloroform-*d*) δ 178.3, 177.9, 135.3, 128.6, 128.5, 128.0, 68.5, 43.0, 42.2, 33.8, 25.8, 22.4, 22.2. **HRMS** (ESI-TOF) m/z: $[M-H]^-$ Calcd for $C_{15}H_{18}NO_3^-$ = 260.1292; Found 260.1290.

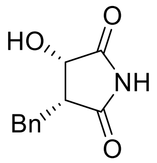

(3*R*,4*S*)-3-benzyl-4-hydroxypyrrolidine-2,5-dione (3y): This is a new compound, white solid, 39 mg, 95% yield, >99% ee, >20:1 dr; $[\alpha]^{20}_D$ = -52.2 (c 0.5, MeOH); **HPLC** (Chiralpak IC column, hexane/isopropanol = 85/15; flow rate = 1.0 mL/min; UV detection at 210 nm; t_1 = 10.0min, t_2 = 11.6 min (major), t_3 = 12.8 min, t_4 = 13.8 min. **^{1}H NMR** (600 MHz, Methanol-*d_4*) δ 7.45 – 7.36 (m, 2H), 7.36 – 7.30 (m, 2H), 7.27 – 7.22 (m, 1H), 4.57 (d, *J* = 7.6 Hz, 1H), 3.30 (td, *J* = 8.1, 5.3 Hz, 1H), 3.19 – 3.06 (m, 2H). **^{13}C NMR** (151 MHz, Methanol-*d_4*) δ 180.7, 180.7, 140.4, 130.2, 129.3, 127.3, 70.1, 49.5, 31.2. **HRMS** (ESI-TOF) m/z: $[M-H]^-$ Calcd for $C_{11}H_{10}NO_3^-$ = 204.0666; Found 204.0660.

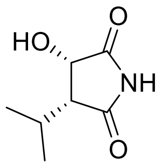

(3*S*,4*R*)-3-hydroxy-4-isopropylpyrrolidine-2,5-dione: This is a new compound, white solid, 30 mg, 95% yield, 99% ee, >20:1 dr; $[\alpha]^{20}_D$ = -61.4 (c 0.5, MeOH); **HPLC** (Chiralpak IC column, hexane/isopropanol = 80/20; flow rate = 1.0 mL/min; UV

detection at 210 nm; t_1 = 5.2 min, t_2 = 6.3 min (major). **^{1}H NMR** (400 MHz, Methanol-d_4) δ 4.65 (d, *J* = 8.3 Hz, 1H), 2.82 (dd, *J* = 8.3, 3.9 Hz, 1H), 2.35 – 2.21 (m, 1H), 1.13 (d, *J* = 7.0 Hz, 3H), 0.93 (d, *J* = 6.8 Hz, 3H). **^{13}C NMR** (101 MHz, Methanol-d_4) δ 181.7, 179.6, 70.5, 53.1, 27.3, 21.4, 19.0. **HRMS** (ESI-TOF) m/z: $[M-H]^-$ Calcd for $C_7H_{10}NO_3^-$ = 156.0666; Found 156.0657.

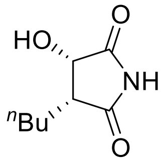

(3*R*,4*S*)-3-butyl-4-hydroxypyrrolidine-2,5-dione (3aa): This is a new compound, white solid, 33 mg, 97% yield, 98% ee, >20:1 dr; $[\alpha]^{20}_D$ = -69.1 (c 1.0, $CHCl_3$); **HPLC** (Chiralpak IC column, hexane/isopropanol = 80/20; flow rate = 1.0 mL/min; UV detection at 210 nm; t_1 = 6.6 min, t_2 = 7.3 min (major). **^{1}H NMR** (600 MHz, Methanol-d_4) δ 4.56 (d, *J* = 7.9 Hz, 1H), 2.86 (td, *J* = 7.8, 6.0 Hz, 1H), 1.79 – 1.69 (m, 1H), 1.67 – 1.56 (m, 1H), 1.50 – 1.40 (m, 2H), 1.39 – 1.31 (m, 2H), 0.92 (t, *J* = 7.3 Hz, 3H). **^{13}C NMR** (151 MHz, Methanol-d_4) δ 181.5, 181.1, 70.5, 47.5, 30.6, 26.2, 23.7, 14.2. **HRMS** (ESI-TOF) m/z: $[M-H]^-$ Calcd for $C_8H_{12}NO_3^-$ = 170.0823; Found 170.0814.

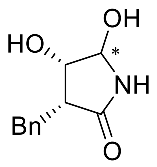

(3*R*,4*S*)-3-benzyl-4,5-dihydroxy-5λ^3-pyrrolidin-2-one (4y): This is a new compound, white solid, 38 mg, 91% yield, >99% ee; $[\alpha]^{20}_D$ = -73.6 (c 0.5, MeOH); **HPLC** (Chiralpak IA column, hexane/isopropanol = 85/15; flow rate = 1.0 mL/min; UV detection at 210 nm; t_1 = 8.9 min, t_2 = 10.1 min (major). **^{1}H NMR** (600 MHz, Methanol-d_4) δ 7.32 – 7.28 (m, 2H), 7.27 – 7.23 (m, 2H), 7.18 – 7.12 (m, 1H), 5.10 (d, *J* = 4.1 Hz, 1H), 4.09 (dd, *J* = 5.6, 4.1 Hz, 1H), 3.04 – 2.93 (m, 2H), 2.73 – 2.66 (m, 1H). **^{13}C NMR** (151 MHz, Methanol-d_4) δ 178.6, 141.8, 129.9, 129.3, 127.0, 80.6, 70.3, 50.2, 30.8. **HRMS** (ESI-TOF) m/z: $[M-H]^-$ Calcd for $C_{11}H_{12}NO_3^-$ = 206.0823;
Found 206.0817.

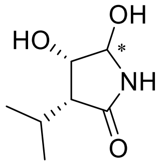

(3*R*,4*S*)-4,5-dihydroxy-3-isopropyl-5λ^3-pyrrolidin-2-one (4z): This is a new compound, white solid, 26 mg, 83% yield, 99% ee; $[\alpha]^{20}_{D}$ = -20.8 (c 0.5, MeOH); **HPLC** (Chiralpak IC column, hexane/isopropanol = 60/40; flow rate = 1.0 mL/min; UV detection at 210 nm; t_1 = 7.0 min, t_2 = 9.1 min (major). **^{1}H NMR** (600 MHz, Methanol-d_4) δ 5.07 (d, *J* = 4.0 Hz, 1H), 4.29 (t, *J* = 4.5 Hz, 1H), 2.20 – 2.05 (m, 2H), 1.15 (d, *J* = 6.1 Hz, 3H), 1.07 (d, *J* = 6.1 Hz, 3H). **^{13}C NMR** (151 MHz, Methanol-d_4) δ 179.1, 79.8, 71.4, 53.4, 26.4, 22.3, 21.3. **HRMS** (ESI-TOF) m/z: $[M+H]^+$ Calcd for $C_7H_{14}NO_3^+$ = 160.0968; Found 160.0968.

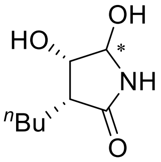

(3*R*,4*S*)-3-butyl-4,5-dihydroxy-5λ^3-pyrrolidin-2-one (4aa): This is a new compound, white solid, 30 mg, 87% yield, 99% ee; $[\alpha]^{20}_{D}$ = -28.4 (c 0.5, MeOH); **HPLC** (Chiralpak IC column, hexane/isopropanol = 60/40; flow rate = 1.0 mL/min; UV detection at 210 nm; t_1 = 8.6 min, t_2 = 10.5 min (major). **^{1}H NMR** (400 MHz, Methanol-d_4) δ 5.11 (d, *J* = 4.4 Hz, 1H), 4.25 (dd, *J* = 6.1, 4.5 Hz, 1H), 2.34 (q, *J* = 6.7 Hz, 1H), 1.67 (q, *J* = 7.8, 7.1 Hz, 2H), 1.54 – 1.28 (m, 4H), 0.93 (t, *J* = 7.1 Hz, 3H). **^{13}C NMR** (151 MHz, Methanol-d_4) δ 180.0, 80.5, 70.5, 47.6, 31.1, 25.4, 23.8, 14.3. **HRMS** (ESI-TOF) m/z: $[M+H]^+$ Calcd for $C_8H_{16}NO_3^+$ = 174.1125; Found 174.1125.

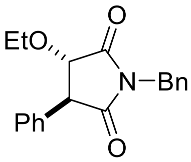

***trans*-1-benzyl-3-ethoxy-4-phenylpyrrolidine-2,5-dione (2a')**: white solid, 56 mg, 90% yield, 37% ee, 85:15 dr; $[\alpha]^{20}_{D}$ = -37.3 (c 1.5, $CHCl_3$); **HPLC** (Chiralpak IC column,

hexane/isopropanol = 80/20; flow rate = 1.0 mL/min; UV detection at 210 nm; t_1 = 8.9 min (major), t_2 = 7.8 min. t_1 = 9.8 min, t_2 = 10.7 min. **^{1}H NMR** (400 MHz, Chloroform-*d*) δ 7.47 – 7.27 (m, 8H), 7.22 – 7.14 (m, 2H), 4.85 – 4.66 (m, 2H), 4.32 (d, *J* = 5.2 Hz, 1H), 4.03 – 3.93 (m, 1H), 3.92 (d, *J* = 5.2 Hz, 1H), 3.73 – 3.57 (m, 1H), 1.20 (t, *J* = 7.0 Hz, 3H). **^{13}C NMR** (101 MHz, Chloroform-*d*) δ 174.6, 174.3, 135.5, 135.4, 129.2, 128.8, 128.8, 128.1, 128.1, 127.8, 81.3, 67.7, 54.2, 42.6, 15.2. **HRMS** (ESI-TOF) m/z: $[M+H]^+$ Calcd for $C_{19}H_{20}NO_3^+$ = 310.1438; Found 310.1435.

Detailed Optimization of Reaction Conditions and Gram-Scale DKR-ATH Procedure

Supplementary Table 1. Optimization of the reaction condition.

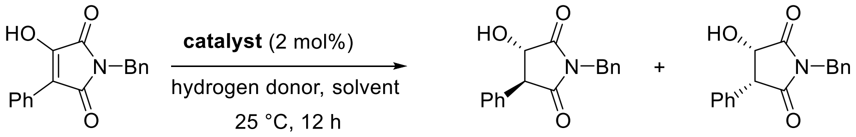

1a *anti*-**2a** *syn*-**3a**

entry	catalyst	hydrogen donor	solvent	conv. (%)	ee_{anti} (%)	ee_{syn} (%)	dr (*anti*/*syn*)
1	*(S,S)-cat.6*	*HCO_2H: Et_3N (5:2)*	*EtOAc*	*>99*	*99*	*-*	*98:2*
2[b]	(*S*,*S*)-**cat.6**	HCO_2H: Et_3N (5:1)	EtOAc	>99	97	-	97:3
3[b]	(*S*,*S*)-**cat.6**	HCO_2H: Et_3N (10:1)	EtOAc	>99	97	-	96:4
4[b]	*(S,S)-cat.6*	*HCO_2H: Et_3N (100:1)*	*EtOAc*	*>99*	*-*	*96*	*2:98*
5[b]	(*S*,*S*)-**cat.6**	HCO_2H: Et_3N (200:1)	EtOAc	86	-	96	3:97
6[b]	(*S*,*S*)-**cat.6**	HCO_2H: Et_3N (2:0)	EtOAc	80	-	94	<1:99
7[c]	(*S*,*S*)-**cat.6**	HCO_2H: Et_3N (2:0)	EtOAc	>99	-	93	<1:99
8[d]	(*S*,*S*)-**cat.6**	^{i}PrOH	-	<5	-	-	-
9[e]	(*S*,*S*)-**cat.6**	HCO_2Na	^{i}PrOH : H_2O (1v/1v)	<5	-	-	-

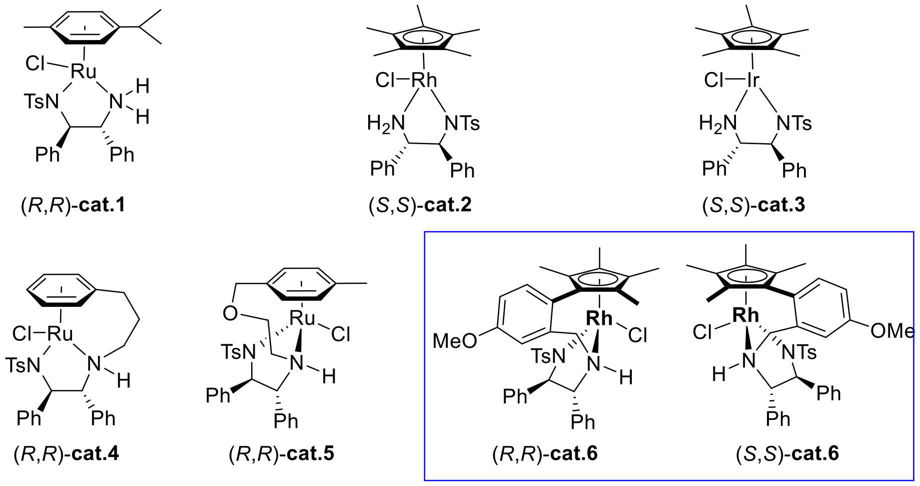

[a] Conducted with catalyst/substrate (0.1 mmol) ratio of 1:50 in 1 mL of solvent, HCO_2H/Et_3N azeotropic mixture (20 μL) at 25 °C for 12 h. Conversions (conv.) were determined by ^1H NMR analysis. Enantiomeric excesses (ee) and diastereomeric ratios (dr) were determined by HPLC analysis using a chiral stationary phase. [b] HCO_2H (2.0 equiv.) was used. [c] HCO_2H (2.0 equiv.) was used for 48 h. [d] KO^{t}Bu (3.0 equiv.) was used in 1.0 mL of ^{i}PrOH at 60 °C for 12 h. [e] HCO_2Na (5.0 equiv.) was used in 2.0 mL of ^{i}PrOH /H_2O (1.0 mL/1.0 mL) at 60 °C for 12 h.

Supplementary Table 2 TON Study ofDKR-ATH of 1u

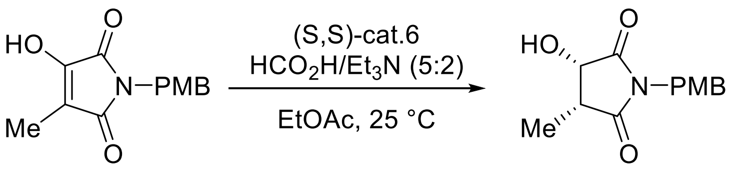

entry	S/C	time (h)	conv. (%)	ee(%)
1	200	2	>99	98
2	500	3	>99	98
3	1000	8	>99	97
4	2000	24	>99	97
5	5000	48	41	97

Conditions: the reactions were carried out with different catalyst/substrate (0.2 mmol) ratio in 2 mL of solvent, HCOOH/Et_3N azeotropic mixture (40 μL) at 25 °C.

The detailed operation process is the same as **procedure E.**

Gram-Scale DKR-ATH Procedure: In a nitrogen filled glovebox, to a 50 mL Schleck tube charged with a magnetic stirring bar were added successively substrate **1u** (5 mmol, 1.24 g), formic acid/trimethylamine azeotropic mixture (5/2 molar ratio) (1.0 mL), the catalyst (**cat.6**, 1.9 mg, 0.0025 mmol) and the solvent (25 mL). The mixture was then stirred at room temperature for 24 h. After completion, The reaction solution was concentrated and the residue was passed through a short column of silica gel (eluent: EA:PE = 2:1) to remove the metal complex. Compounds **3u** was obtained as a white solid (1.24 g, 99% yield, 97% ee, >20:1 dr).

3. Supplementary Discussion

Mechanistic Study

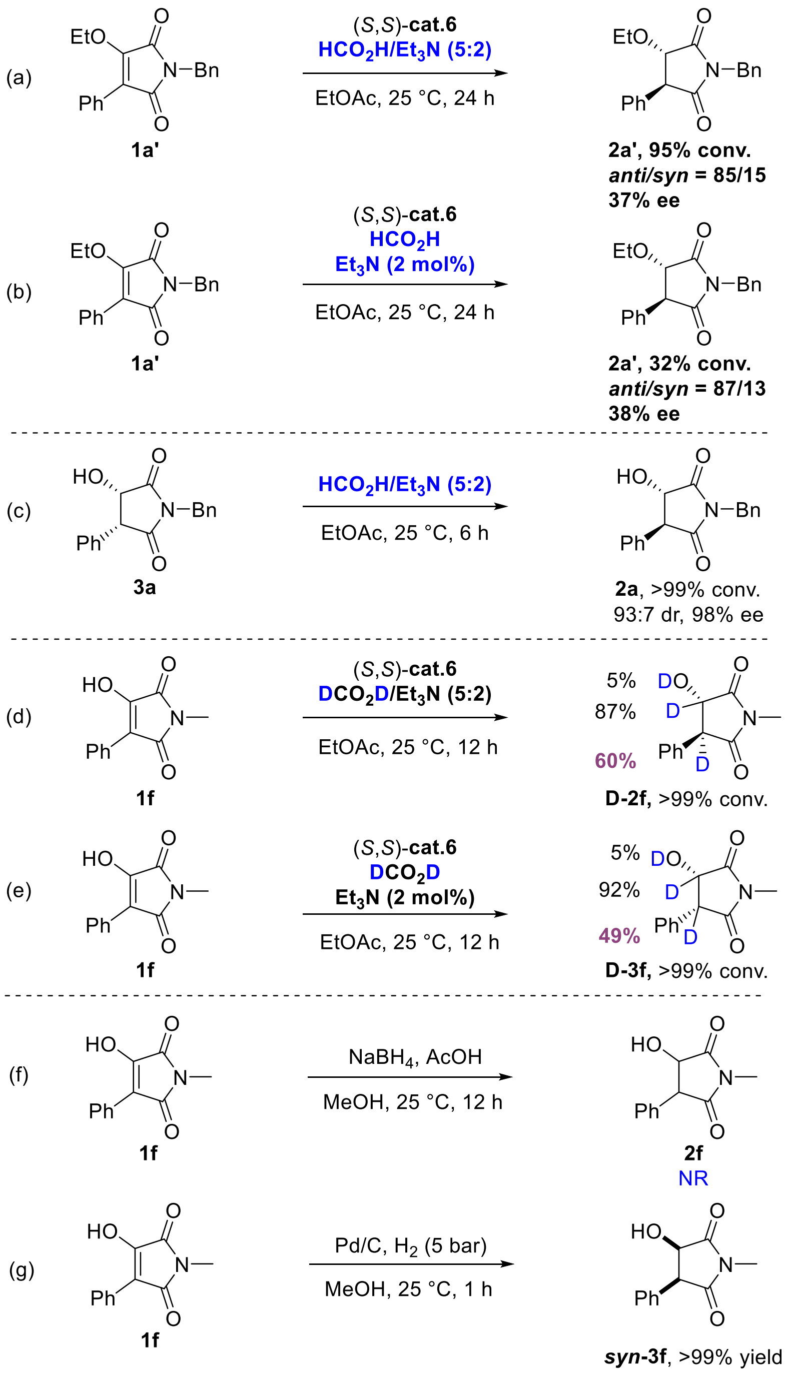

Supplementary Figure 9. Deuterium labeling experiment and control experiment.

Control experiments:

For eq. a: According to **procedure E**, **1a'** was reduced and *anti*-2a' was obtained as major product (90% yield, 37% ee, 85/15 *anti/syn*)

For eq. b: According to **procedure F**, **1a'** was reduced and *anti*-2a' was obtained as major product (30% yield, 38% ee, 87/13 *anti/syn*)

For eq. c: To a solution of **3a** (0.1 mmol) in EtOAc was added HCO_2H/Et_3N (5:2) (20 uL) and the mixture was then stirred at room temperature. After 6 h, the reaction solution was concentrated to obtained **2a** with 97% ee and 93:7 dr.

Deuterium labeling experiments:

For eq. d: To a 10 mL Schleck tube charged with a magnetic stirring bar were added successively substrate **1f** (0.1 mmol), DCO_2D (2.0 equiv., 7.6 μL)/Et_3N (0.8 equiv.), the catalyst (1.5 mg) and the solvent (1 mL). The mixture was then stirred at room temperature for the indicated reaction time. After completion, the reaction solution was concentrated to obtain the crude product **D-2f.**

For eq. e: To a 10 mL Schleck tube charged with a magnetic stirring bar were added successively substrate **1f** (0.1 mmol), DCO_2D (2.0 equiv., 7.6 μL), Et_3N (0.28 μL), the catalyst (**cat.6,** 1.5 mg) and the solvent (1 mL). The mixture was then stirred at room temperature for the indicated reaction time. After completion, the reaction solution was concentrated to obtain the crude product **D-3f.**

Supplementary Table 3. Kinetic experiments of 1a.

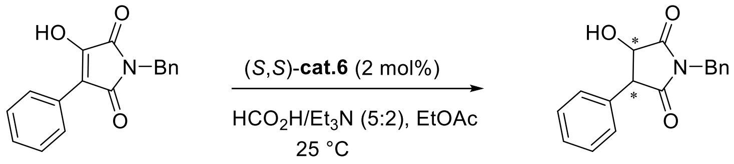

entry	reaction time	conv.%	syn/anti
1	5 min	23	72/28
2	10 min	50	54/46
3	30 min	73	46/54
4	1 h	91	7/93
5	2 h	98	2/98
6	3 h	--	--
7	4 h	--	--

Reaction conditons: Conducted with catalyst/substrate (0.1 mmol) ratio of 1:50 in 1 mL of solvent, HCO_2H/Et_3N azeotropic mixture (20 μL) at 25 °C.

Supplementary Table 4. Kinetic experiments of 1a.

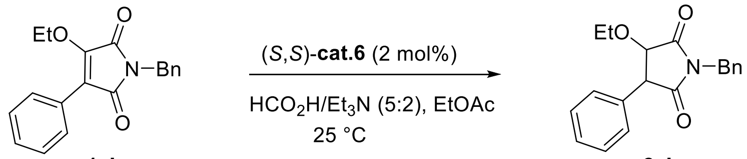

entry	reaction time	conv.%	syn/anti
1	5 min	<5	--
2	10 min	7	--
3	30 min	21	11/89
4	1 h	68	12/88
5	2 h	88	12/88
6	3 h	95	12/88
7	4 h	98	12/88

Reaction conditons: Conducted with catalyst/substrate (0.1 mmol) ratio of 1:50 in 1 mL of solvent, HCO_2H/Et_3N azeotropic mixture (20 μL) at 25 °C.

Supplementary Table 5. pH effect on the keto-enol equilibrium.

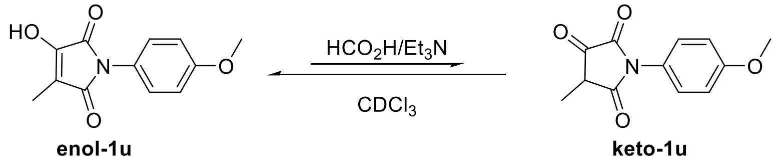

entry	HCO_2H/Et_3N	pH	**keto-1u/enol-1u**
1	2:0	0.7	1/15.2
2	2:0.002	0.84	1/15.3
3	2:0.02	1.28	1/15.7
4	2:0.2	1.9	1/17.2
5	2:0.8	3.22	--

Reaction conditons: Conducted with substrate (0.1 mmol) in 0.5 mL of solvent using varieties of ratio of HCO_2H (2.0 equiv.)/Et_3N at 25 °C, the **keto-1u** and the **enol-1u** was determined by HNMR.

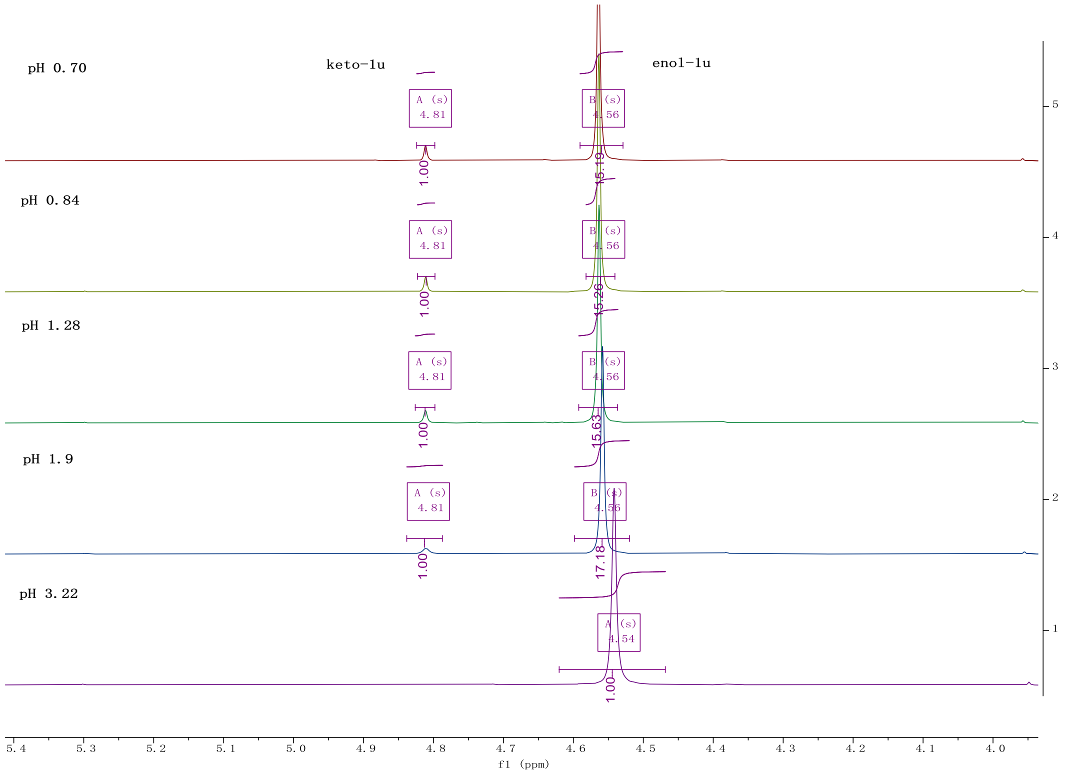

Supplementary Figure 10. The NMR spectra tracking for the keto-enol equilibrium experiment.

Computational methods

Molecular geometries were optimized without constraints via DFT calculations using the B3LYP functional[11] with Grimme's D3(BJ) dispersion correction[12]. The effective core potential (ECP) of the SDD[13] was chosen to describe Rh, and the 6-31G(d) basis set was used for other atoms. Frequency calculations were carried out at the same level of theory to identify all of the stationary points as transition states (one imaginary frequency) or as minima (zero imaginary frequencies) and to provide the thermal correction to free energies at 298.15 K and 1 atm. Intrinsic reaction coordinates (IRC) were calculated for all transition states to confirm that these structures indeed connect two relevant minima. The single-point energy calculations were performed at the MN15[14] level with def2TZVPP[15] for all atoms in order to get higher accuracy electronic energy. Solvation effect are account for all calculations using the continuum solvent model IEFPCM1[16], ethyl ethanoate, which was used as the solvent. All calculations were performed with the Gaussian 16 software package.[17]

Results

Based on the experimental results as well as computational analysis, a plausible pathway for the formation of the active catalyst was shown in Supplementary Figure 11. First, the Et_3N captures HCl from pre-catalyst **cat.6** to generate the 16-electron amide complex **cat0-0** with a strong endergonic process of 19.8 kcal/mol. The **cat0-0** with vacant coordination is able to coordinate with one equivalent of HCOOH to generate the complex **cat0-1**. This is exergonic by 18.2 kcal/mol relative to **cat0-0**. Subsequently, **cat0-1** undergoes a rotation of $HCOO^-$ to produce intermediate **cat0-2** with Rh-H agnostic, followed by the hydride transfer step through **cat0-TS1** to produce **cat1** and CO_2. It is found that transition state **cat0-2** and intermediate **cat0-TS1** are in free energies very close, and it means that the process from **cat0-2** to **cat0-TS1** should be very easy.

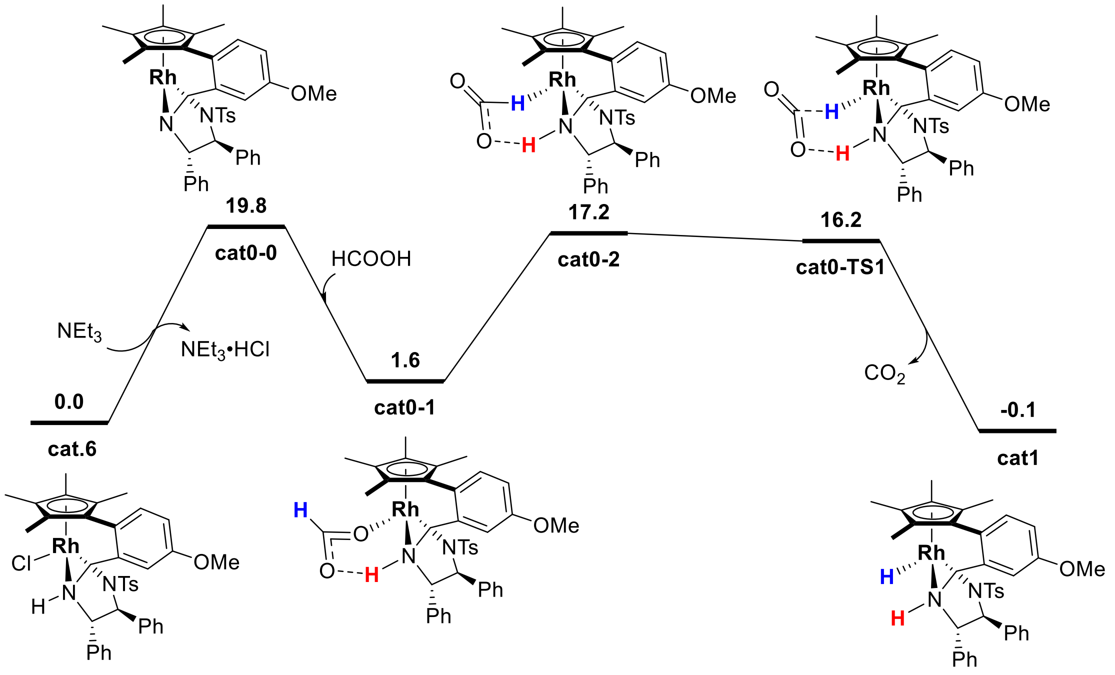

Supplementary Figure 11. The formation of active catalyst. The energies are given in kcal/mol.

The detailed calculations were also carried out for the substrate with the protected OH group (Supplementary Figure 12). There are four pathways respectively leading to the enantio- and diastereoselectivity from reactant to the products. First, the pathway to generate *S* configuration at C3 position can experience a more stable transition states **b-S-TS1** (18.2 kcal/mol) than **b-R-TS1** (20.9 kcal/mol) forming *R* configuration at C3 position. From intermediate **b-S-int2** and **b-R-int2**, via four transition states **b-SS-TS2** (18.1 kcal/mol), **b-RS-TS2** (20.1 kcal/mol), **b-RR-TS2** (19.2 kcal/mol), and **b-SR-TS2** (23.7 kcal/mol), respectively, four different stereoselective products are generated. The results show that the pathway leading to the product **SS-pro1** is more favorable and has a relatively low energy barrier (18.2 kcal/mol from **cat1** to **b-S-TS1**). This is also consistent with the experimental results involving the substrates with the protected OH group

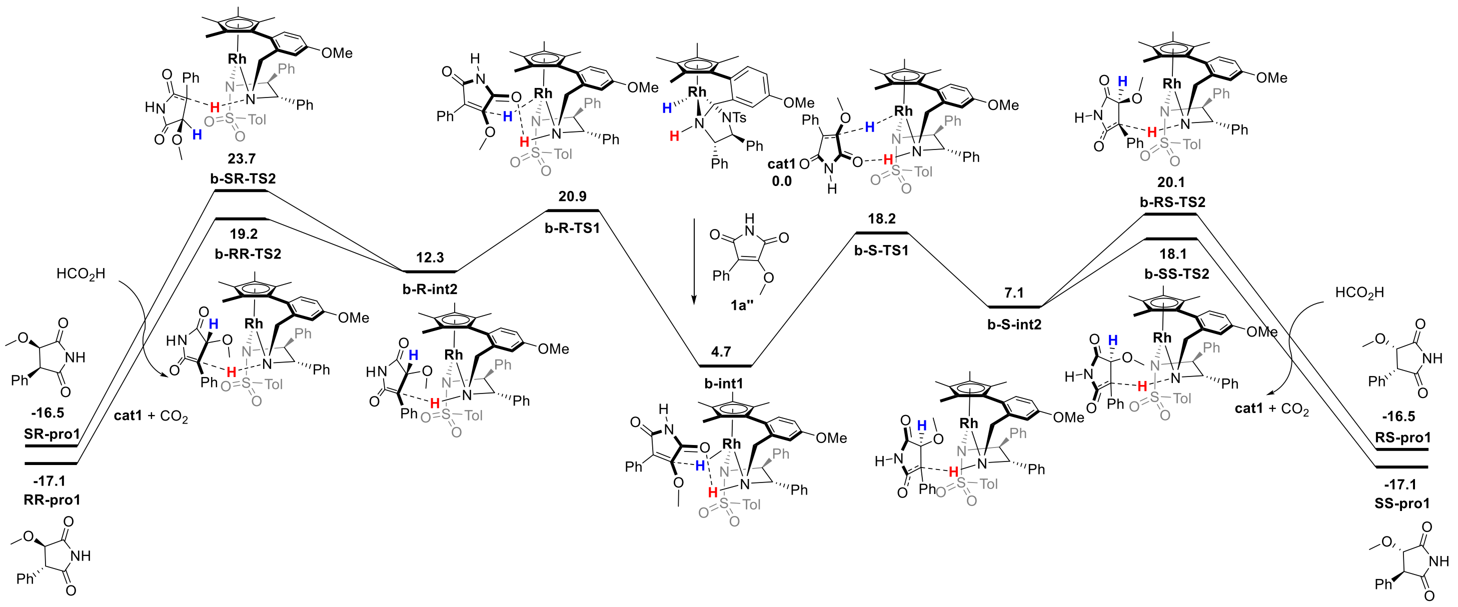

Supplementary Figure 12. Proposal catalytic cycle for ATH of 1a". The energies are given in kcal/mol

Synthetic Applications

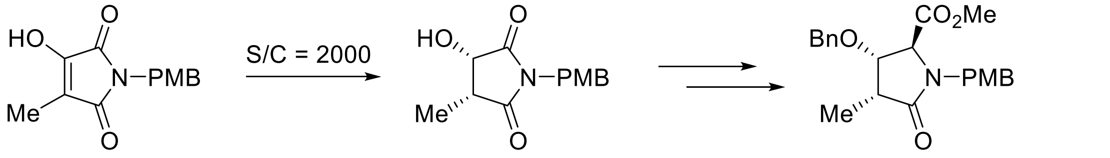

Scale-Up Reaction: In a nitrogen filled glovebox, to a 50 mL Schleck tube charged with a magnetic stirring bar were added successively substrate **1u** (5 mmol, 1.24 g), formic acid/trimethylamine azeotropic mixture (5/2 molar ratio) (1.0 mL), the catalyst (**cat.6**, 1.9 mg, 0.0025 mmol) and the solvent (25 mL). The mixture was then stirred at room temperature for 24 h. After completion, The reaction solution was concentrated and the residue was passed through a short column of silica gel (eluent: EA:PE = 2:1) to remove the metal complex. Compounds **3u** was obtained as a white solid (1.24 g, 99% yield, 97% ee, >20:1 dr)

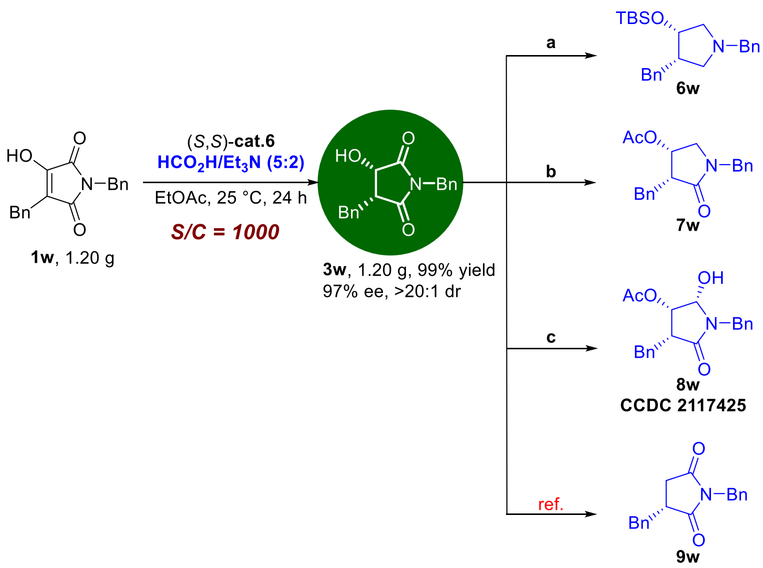

Synthesis of 6w

Step 1: In a nitrogen filled glovebox, to a 50 mL Schleck tube charged with a magnetic stirring bar were added successively substrate **1w** (4 mmol, 1.2 g), formic acid/trimethylamine azeotropic mixture (5/2 molar ratio) (0.8 mL), the catalyst (**cat.6**, 3.0 mg, 0.004 mmol) and the solvent (30 mL). The mixture was then stirred at room temperature for 12 h. After completion, The reaction solution was concentrated and the residue was passed through a short column of silica gel (eluent: EA:PE = 2:1) to remove the metal complex. Compounds **3w** was obtained as a white solid (1.2 g, 99% yield, 97% ee, >20:1 dr).

Step 2: To a solution of compound **3w** (0.6 g, 2 mmol, 1.0 equiv.) and 1*H*-imidazole (0.4 g, 6 mmol, 3.0 equiv.) in CH_3CN was cooled to 0 ºC, and then TBSOTf (0.92 mL, 4 mmol, 2 equiv.) was added into the mixture slowly. The reaction mixture was then warmed up to room temperature and was stirred for 2 hours. After then, it was diluted with water (5 mL) and extracted by ethyl acetate twice (10 mL for each). The combined organic layer was washed with brine (10 mL), and then solvent was removed by rotary evaporator to produce the crude products, which were purified by flash chromatography (SiO_2, 10% EtOAc in petroleum ether). The product were obtained as colorless oil (0.8 g, 97% yield).

Step 3: The product from step 2 (0.8 g, 1.0 equiv.) and $AlCl_3$ (5.0 equiv.) was dissolved in THF (20 mL), and the mixture was cooled to 0 ºC. $NaBH_4$ (5.0 equiv.) was added into the mixture in batches. The mixture was warmed to 10 ºC for 12 h until the start

material consumed completely. The resulting product was treated with brine (10 mL), extracted with EA (10 mL × 3), combined organic layer, dried over anhydrous Na_2SO_4, and concentrated in vacuo. The crude product was purified by flash chromatography (8% EtOAc in petroleum ether) to yield **6w** (1.14 g, 75% yield, 97% ee) as a colorless oil. **^{1}H NMR** (400 MHz, Chloroform-*d*) δ 7.41 – 7.35 (m, 2H), 7.35 – 7.29 (m, 3H), 7.25 – 7.19 (m, 2H), 7.17 – 7.06 (m, 3H), 4.39 (q, *J* = 4.7 Hz, 1H), 4.07 – 3.95 (m, 2H), 3.37 – 3.28 (m, 1H), 3.08 – 2.85 (m, 4H), 2.82 – 2.74 (m, 1H), 2.55 – 2.42 (m, 1H), 0.86 (s, 9H), -0.00 (s, 3H), -0.06 (s, 3H). **^{13}C NMR** (101 MHz, Chloroform-*d*) δ 139.7, 132.8, 132.1, 128.8, 128.5, 128.4, 128.0, 126.2, 72.6, 68.3, 68.0, 63.9, 43.2, 33.4, 25.8, 18.0, -4.5, -5.3. $[\alpha]^{25}_D$ = +26.8 (c 0.25, $CHCl_3$); **HPLC** (Chiralpak IC column, hexane/isopropanol = 85/15; flow rate = 1.0 mL/min; UV detection at 210 nm; t_1 = 5.5 min (major), t_2 = 6.1 min. **HRMS** (ESI-TOF) m/z: $[M+H]^+$ Calcd for $C_{24}H_{36}NOSi^+$ = 382.2561; Found 382.2556.

Synthesis of 7w and 8w[16]

Step 1: To a solution of compound **3w** (0.6 g, 2 mmol, 1.0 equiv.), DMAP (25 mg, 0.2 mmol, 0.1 equiv.), Ac_2O (3 mmol, 1.5 equiv.) in DCM (10 mL) was cooled to 0 ºC, and then Et_3N (3 mmol, 1.5 equiv.) was added into the mixture slowly. The reaction mixture was then warmed up to room temperature and was stirred for 1 hours. After then, it was diluted with brine (5 mL) and extracted by DCM twice (20 mL for each). The combined organic layer was dried over anhydrous Na_2SO_4, and then solvent was removed by rotary evaporator to produce the crude products, which were purified by flash chromatography (SiO_2, 15% EtOAc in petroleum ether). The product were obtained as colorless oil (0.6 g, 88% yield).

Step 2: The product from **step 1** (0.34 g, 1 mmol, 1.0 equiv.) was dissolved in THF (5 mL), and the mixture was cooled to 0 ºC. $NaBH_4$ (1.5 equiv.) was added into the mixture in batches. The mixture was stirred for 10 min and sat. $NaHCO_3$ (2 mL) was added into the mixture. After 30 min, the resulting product was treated with brine (10 mL), extracted with EA (10 mL × 3), combined organic layer, dried over anhydrous Na_2SO_4, and concentrated in vacuo. The crude product was purified by flash chromatography (30% EtOAc in petroleum ether) to obtained **8w** (0.32 g, 95% yield, >20:1 dr) as a

white solid. **CCDC** 2117425 ^{1}H NMR (600 MHz, Chloroform-*d*) δ 7.26 – 7.17 (m, 7H), 7.17 – 7.08 (m, 3H), 5.17 (t, *J* = 5.3 Hz, 1H), 4.90 (s, 1H), 4.81 (d, *J* = 14.6 Hz, 1H), 4.03 (d, *J* = 14.6 Hz, 1H), 3.14 (q, *J* = 8.4 Hz, 1H), 3.01 – 2.87 (m, 2H), 2.01 (s, 3H). ^{13}C NMR (151 MHz, Chloroform-*d*) δ 172.4, 169.9, 138.6, 135.9, 129.0, 128.7, 128.6, 128.5, 127.8, 126.8, 80.2, 68.9, 45.8, 43.4, 31.4, 20.5. $[\alpha]^{20}_D$ = -108.7 (c 1.0, $CHCl_3$); **HRMS** (ESI-TOF) m/z: $[M+H]^+$ Calcd for $C_{20}H_{22}NO_4^+$ = 340.1543; Found 340.1544.

Step 3: To a solution of compound **8w** (0.17 g, 0.5 mmol, 1.0 equiv.), DMAP (12 mg, 0.1 mmol, 0.1 equiv.), Ac_2O (0.75 mmol, 1.5 equiv.) in DCM (5 mL) was cooled to 0 ºC, and then Et_3N (0.75 mmol, 1.5 equiv.) was added into the mixture slowly. The reaction mixture was then warmed up to room temperature and was stirred for 1 hours. After then, it was diluted with brine (3 mL) and extracted by DCM twice (10 mL for each). The combined organic layer was dried over anhydrous Na_2SO_4, and then solvent was removed by rotary evaporator to produce the crude products, which were purified by flash chromatography (SiO_2, 15% EtOAc in petroleum ether). The product (2*S*,3*S*,4*R*)-1,4-dibenzyl-5-oxopyrrolidine-2,3-diyl diacetate were obtained as white solid (0.19 g, 99% yield).

Step 4: To a solution of (2*S*,3*S*,4*R*)-1,4-dibenzyl-5-oxopyrrolidine-2,3-diyl diacetate (0.19, 0.5 mmol) in DCM (5 mL) was added dropwise $BF_3.Et_2O$ (0.4 mL, 3.1 mmol) at 78 °C (over 10 min) under N_2 atmosphere. The mixture was stirred for 20 min and then treated with triethylsilane (5equiv.). The temperature was gradually raised to rt over 3 h. After stirring for 18 h, the mixture was poured into ice-water containing Na_2CO_3. The mixture was washed with DCM and the organic layer was dried (MgSO4), concentrated, and purified by flash column chromatography (EtOAc/hexane=1:4) to give **7w** (0.15 g, 93%) as a white solid. **^{1}H NMR (600 MHz, Chloroform-*d*)** δ 7.31 – 7.17 (m, 5H), 7.16 – 7.06 (m, 5H), 5.04 (t, *J* = 4.3 Hz, 1H), 4.52 – 4.33 (m, 2H), 3.39 – 3.19 (m, 2H), 3.03 (d, *J* = 11.9 Hz, 1H), 2.89 – 2.76 (m, 2H), 1.96 (s, 3H). **^{13}C NMR (151 MHz, Chloroform-*d*)** δ 173.0, 170.1, 139.2, 135.8, 128.7, 128.6, 128.5, 128.0, 127.7, 126.4, 68.7, 51.5, 47.8, 46.4, 30.1, 20.8. $[\alpha]^{20}_D$ = -155.2 (c 1.0, $CHCl_3$); **HRMS** (ESI-TOF) m/z: $[M+H]^+$ Calcd for $C_{20}H_{22}NO_3^+$ = 324.1594; Found 324.1594.

4. Supplementary Figures

Crystallographic Information

The crystal data of compound **2g** has been deposited in CCDC with number 2040537.

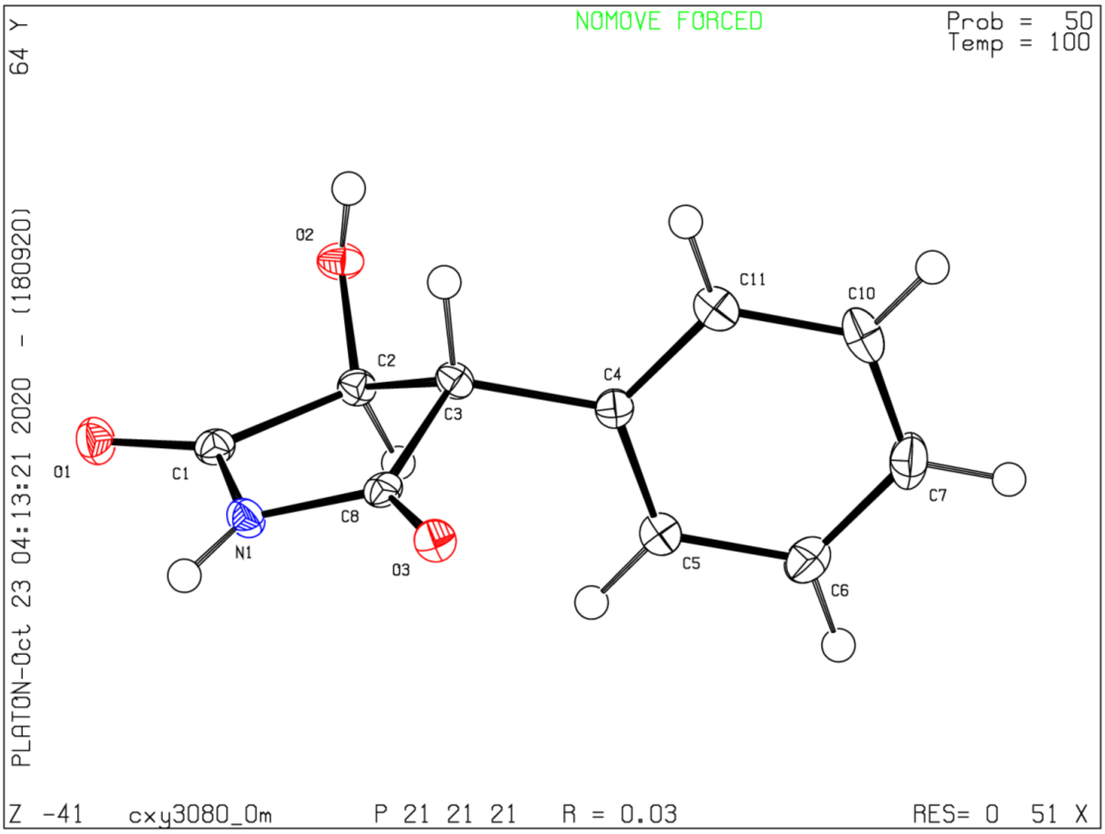

Supplementary Table 6. Crystal data and structure refinement for 2g.	
Identification code	cxy3080_0m
Empirical formula	$C_{10}H_9NO_3$
Formula weight	191.18
Temperature/K	100
Crystal system	orthorhombic
Space group	$P2_12_12_1$
a/Å	5.0555(2)
b/Å	8.7313(4)
c/Å	19.4510(10)
α/°	90
β/°	90
γ/°	90
Volume/Å^3	858.59(7)

Z	4
ρ_{calc}g/cm^3	1.479
μ/mm^{-1}	0.927
F(000)	400.0
Crystal size/mm^3	0.32 × 0.21 × 0.19
Radiation	CuKα (λ = 1.54178)
2Θ range for data collection/°	11.108 to 143.786
Index ranges	-6 ≤ h ≤ 5, -9 ≤ k ≤ 10, -19 ≤ l ≤ 23
Reflections collected	8866
Independent reflections	1686 [R_{int} = 0.0308, R_{sigma} = 0.0206]
Data/restraints/parameters	1686/0/129
Goodness-of-fit on F^2	1.075
Final R indexes [I>=2σ (I)]	R_1 = 0.0255, wR_2 = 0.0663
Final R indexes [all data]	R_1 = 0.0259, wR_2 = 0.0666
Largest diff. peak/hole / e Å^{-3}	0.25/-0.19
Flack parameter	-0.02(5)

Supplementary Table 7. Fractional Atomic Coordinates (×10^4) and Equivalent Isotropic Displacement Parameters (Å^2×10^3) for 2g. U_{eq} is defined as 1/3 of of the trace of the orthogonalised U_{IJ} tensor.

Atom	*x*	*y*	*z*	U(eq)
O1	1948(2)	5286.2(13)	5416.5(6)	17.5(3)
O2	2921(2)	7675.0(13)	4398.2(6)	14.8(3)
O3	9104(2)	3501.5(13)	4242.5(6)	14.8(3)
C1	3530(3)	5181.1(19)	4950.2(8)	13.2(4)
C2	3724(3)	6154.2(18)	4302.5(8)	12.7(3)
C3	6569(3)	5862.5(19)	4046.9(8)	12.2(3)
C4	6985(3)	5868.5(18)	3275.9(8)	12.8(3)
C5	5471(4)	4929.3(19)	2852.2(9)	17.8(4)
C6	5881(4)	4929(2)	2144.8(9)	21.9(4)
C7	7821(4)	5855(2)	1859.0(9)	22.5(4)
C8	7247(3)	4331.1(18)	4385.7(8)	12.4(3)
N1	5443(3)	4062.4(16)	4902.5(7)	13.8(3)
C10	9325(4)	6799(2)	2278.3(9)	21.0(4)
C11	8899(4)	6813.6(19)	2984.1(9)	16.4(4)

The crystal data of compound **2p** has been deposited in CCDC with number 2074951.

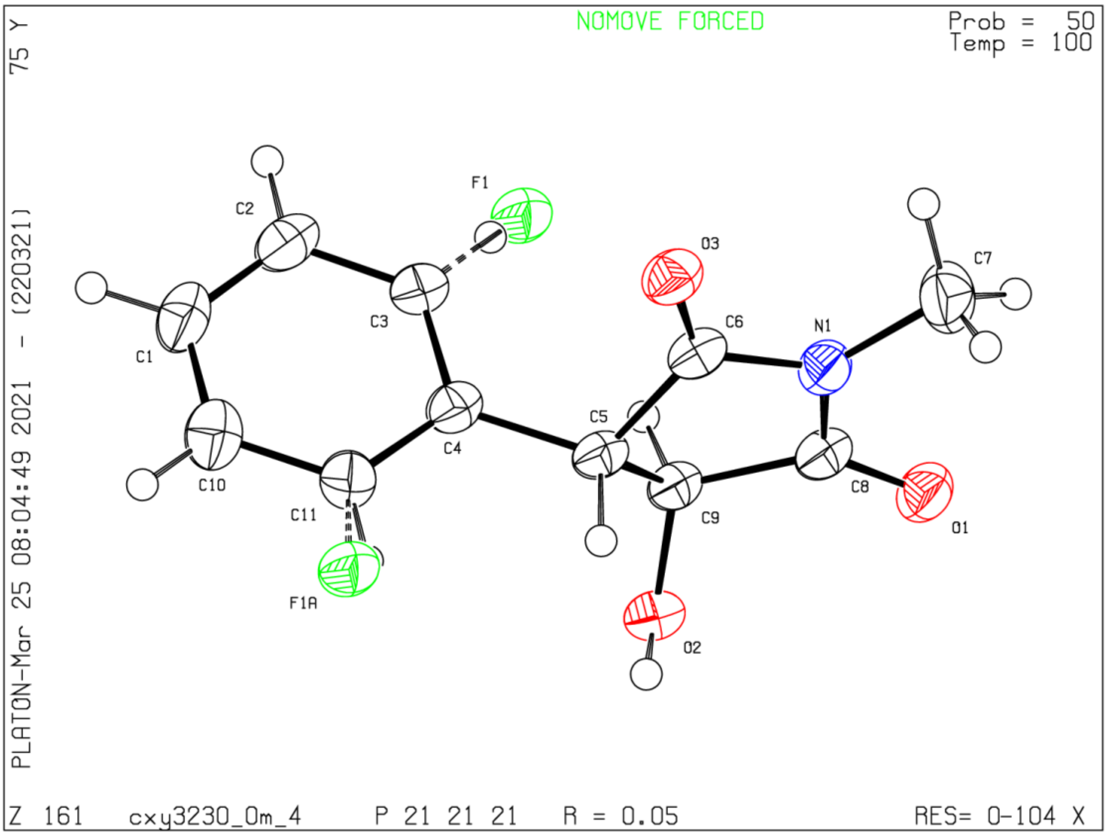

**Supplementary Table 8. Crystal data and structure refinement for 2p.**	
Identification code	cxy3230_0m_4
Empirical formula	$C_{11}H_{10}FNO_3$
Formula weight	223.20
Temperature/K	100.0
Crystal system	orthorhombic
Space group	$P2_12_12_1$
a/Å	4.9616(4)
b/Å	8.3141(7)
c/Å	25.024(2)
α/°	90
β/°	90
γ/°	90
Volume/Å^3	1032.27(15)
Z	4
ρ_{calc}g/cm^3	1.436
μ/mm^{-1}	0.999
F(000)	464.0
Crystal size/mm^3	0.31 × 0.25 × 0.22
Radiation	CuKα (λ = 1.54178)

2Θ range for data collection/°	7.064 to 144.238
Index ranges	? ≤ h ≤ ?, ? ≤ k ≤ ?, ? ≤ l ≤ ?
Reflections collected	2020
Independent reflections	2020 [R_{int} = ?, R_{sigma} = 0.0282]
Data/restraints/parameters	2020/1/152
Goodness-of-fit on F^2	1.185
Final R indexes [I>=2σ (I)]	R_1 = 0.0524, wR_2 = 0.1579
Final R indexes [all data]	R_1 = 0.0527, wR_2 = 0.1582
Largest diff. peak/hole / e Å^{-3}	0.31/-0.24
Flack parameter	0.11(17)

Supplementary Table 9. Fractional Atomic Coordinates (×10^4) and Equivalent Isotropic Displacement Parameters (Å^2×10^3) for 2p. U_{eq} is defined as 1/3 of of the trace of the orthogonalised U_{IJ} tensor.

Atom	*x*	*y*	*z*	U(eq)
F1	5263(5)	2981(3)	6928.0(8)	39.4(7)
O1	3214(5)	4959(3)	5178.0(9)	35.8(6)
O2	4167(5)	1534(3)	5352.3(9)	32.0(6)
O3	10307(5)	4917(3)	6332.0(9)	35.0(6)
N1	6753(7)	5319(3)	5753.0(11)	32.1(7)
C1	9084(8)	-544(5)	7281.8(15)	41.5(9)
C2	7282(9)	722(5)	7324.0(13)	39.7(9)
C3	6951(8)	1719(4)	6889.5(13)	33.0(8)
C4	8309(7)	1512(4)	6409.9(12)	29.3(7)
C5	7856(7)	2621(4)	5942.9(12)	28.7(7)
C6	8513(7)	4386(4)	6052.7(12)	30.3(7)
C7	6957(10)	7067(5)	5711.8(17)	47.4(10)
C8	4811(7)	4430(4)	5501.2(12)	31.1(7)
C9	4961(7)	2720(4)	5718.7(13)	30.5(8)
C10	10515(8)	-782(5)	6815.1(15)	41.1(9)
C11	10125(8)	233(4)	6381.6(13)	34.1(8)
F1A	12000(70)	-50(60)	6063(15)	39.4(7)

The crystal data of compound **3e** has been deposited in CCDC with number 2074952.

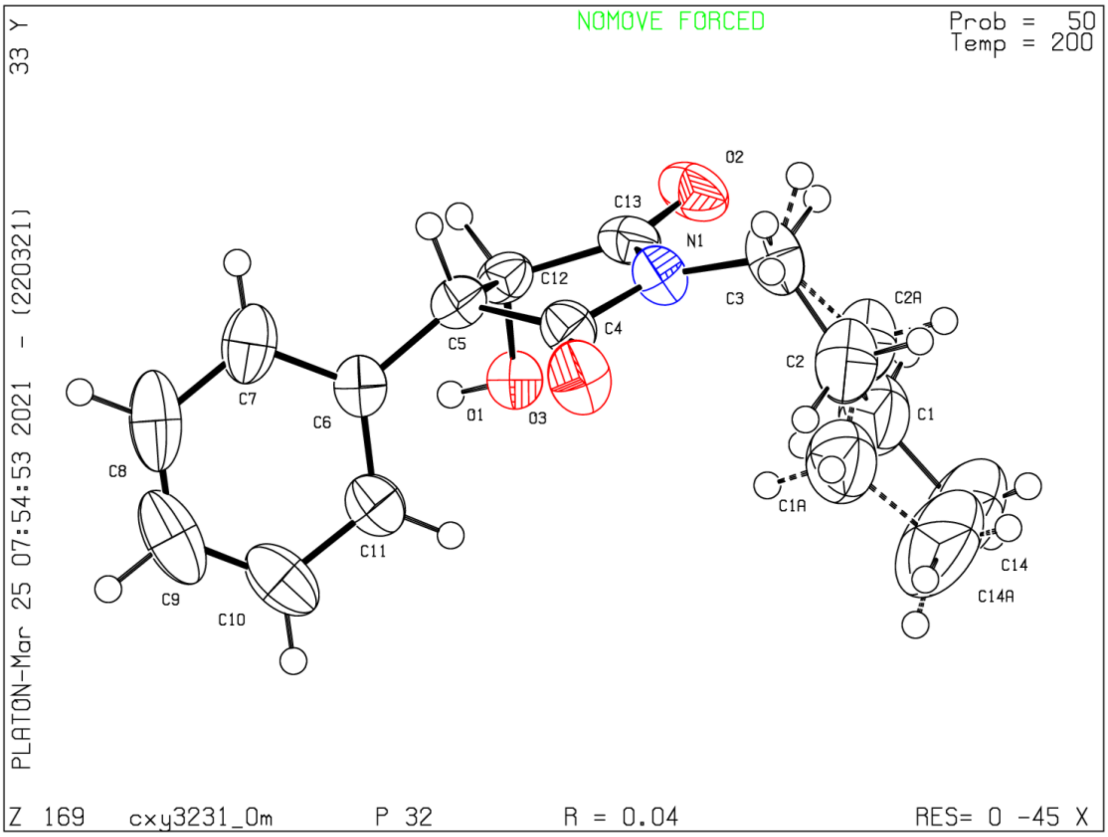

**Supplementary Table 10. Crystal data and structure refinement for 3e.**	
Identification code	cxy3231_0m
Empirical formula	$C_{14}H_{17}NO_3$
Formula weight	247.28
Temperature/K	200.0
Crystal system	trigonal
Space group	$P3_2$
a/Å	14.2104(3)
b/Å	14.2104(3)
c/Å	5.6788(2)
α/°	90
β/°	90
γ/°	120
Volume/Å^3	993.12(5)
Z	3
ρ_{calc}g/cm^3	1.240
μ/mm^{-1}	0.711
F(000)	396.0

Crystal size/mm^3	0.35 × 0.28 × 0.26
Radiation	CuKα (λ = 1.54178)
2Θ range for data collection/°	7.182 to 136.452
Index ranges	$-17 \le h \le 16$, $-17 \le k \le 17$, $-6 \le l \le 6$
Reflections collected	11562
Independent reflections	2424 [R_{int} = 0.0301, R_{sigma} = 0.0186]
Data/restraints/parameters	2424/12/177
Goodness-of-fit on F^2	1.074
Final R indexes [I>=2σ (I)]	R_1 = 0.0368, wR_2 = 0.1027
Final R indexes [all data]	R_1 = 0.0373, wR_2 = 0.1032
Largest diff. peak/hole / e Å^{-3}	0.37/-0.13
Flack parameter	0.01(5)

Supplementary Table 11. Fractional Atomic Coordinates (×10^4) and Equivalent Isotropic Displacement Parameters (Å^2×10^3) for 3e. U_{eq} is defined as 1/3 of of the trace of the orthogonalised U_{IJ} tensor.

**Atom**	***x***	***y***	***z***	**U(eq)**
O1	1467.2(14)	1588.8(14)	2811(3)	41.5(4)
O2	54.2(15)	1477.3(19)	6854(4)	59.0(6)
O3	3679.2(17)	3830.3(17)	7397(4)	58.9(6)
N1	1821.2(18)	2786.6(18)	7536(4)	45.8(5)
C1	1096(6)	4310(6)	5731(12)	86.5(17)
C2	1811(7)	4510(6)	7812(12)	83.9(17)
C3	1618(3)	3520(3)	8988(7)	66.1(8)
C4	2868(2)	2987(2)	7011(4)	42.0(6)
C5	2747.0(19)	1934.9(19)	6077(4)	39.0(5)
C6	3608(2)	2012(2)	4442(4)	40.1(5)
C7	4058(3)	1353(3)	4831(6)	58.6(8)
C8	4841(3)	1390(4)	3276(9)	78.1(12)
C9	5149(3)	2056(4)	1354(8)	79.4(13)
C10	4710(3)	2713(3)	962(6)	64.8(9)
C11	3948(2)	2694(2)	2491(5)	47.8(6)
C12	1564.8(19)	1331.1(19)	5179(5)	39.9(5)
C13	1027(2)	1831(2)	6620(5)	44.5(6)
C14	1230(50)	5380(30)	4780(90)	147(10)
C2A	1181(17)	4124(16)	7420(40)	83.9(17)
C1A	1998(16)	4863(16)	5780(30)	86.5(17)
C14A	1630(70)	5580(70)	4470(170)	147(10)

The crystal data of compound **8w** has been deposited in CCDC with number 2117425.

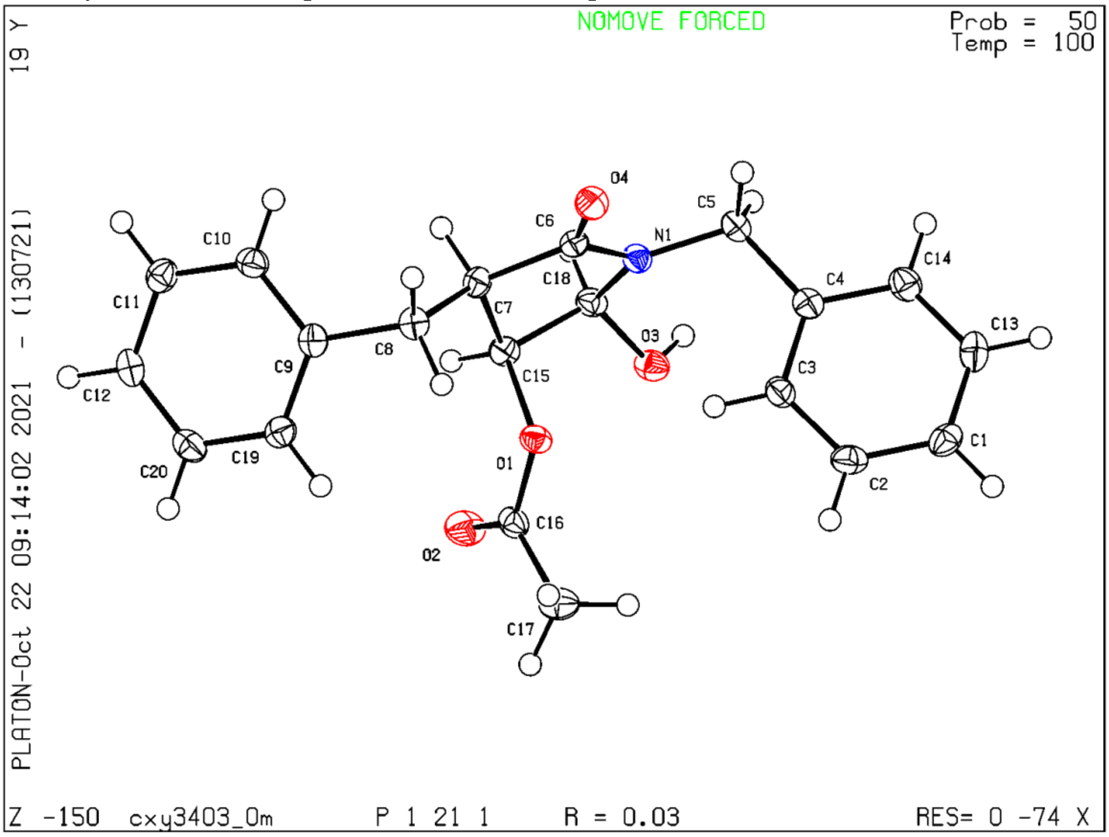

**Supplementary Table 12. Crystal data and structure refinement for 8w.**	
Identification code	cxy3403_0m
Empirical formula	$C_{20}H_{21}NO_4$
Formula weight	339.38
Temperature/K	100.00
Crystal system	monoclinic
Space group	$P2_1$
a/Å	9.3338(4)
b/Å	9.2673(4)
c/Å	9.9618(4)
α /°	90
β /°	99.2090(10)
γ /°	90
Volume/Å^3	850.58(6)
Z	2
ρ_{calc}g/cm^3	1.325
μ /mm^{-1}	0.753
F(000)	360.0
Crystal size/mm^3	0.32 × 0.29 × 0.26
Radiation	CuKα (λ = 1.54178)

2Θ range for data collection/°	8.992 to 136.828
Index ranges	$-11 \leqslant h \leqslant 11$, $-11 \leqslant k \leqslant 11$, $-11 \leqslant l \leqslant 12$
Reflections collected	14224
Independent reflections	3113 [R_{int} = 0.0485, R_{sigma} = 0.0367]
Data/restraints/parameters	3113/1/229
Goodness-of-fit on F^2	1.055
Final R indexes [I>=2σ (I)]	R_1 = 0.0258, wR_2 = 0.0629
Final R indexes [all data]	R_1 = 0.0261, wR_2 = 0.0630
Largest diff. peak/hole / e Å^{-3}	0.19/-0.14
Flack parameter	0.07(6)

Supplementary Table 13. Fractional Atomic Coordinates (×10^4) and Equivalent Isotropic Displacement Parameters (Å^2×10^3) for 8w. U_{eq} is defined as 1/3 of the trace of the orthogonalised U_{IJ} tensor.

Atom	*x*	*y*	*z*	U(eq)
O1	4577.3(13)	5097.3(13)	1693.0(12)	18.3(3)
O2	3908.8(16)	3934.6(16)	-297.9(14)	28.3(3)
O3	3243.9(14)	3016.6(14)	2920.1(13)	21.4(3)
O4	6901.8(14)	5760.6(14)	5307.4(13)	21.6(3)
N1	5098.0(16)	4154.6(16)	4493.7(15)	17.4(3)
C1	545(2)	6895(2)	5249(2)	22.2(4)
C2	1470(2)	6986(2)	4303.3(19)	23.2(4)
C3	2686(2)	6094(2)	4401.3(18)	19.9(4)
C4	2982.6(19)	5109(2)	5464.2(17)	17.2(3)
C5	4317(2)	4146.1(19)	5641.1(18)	19.0(4)
C6	6305.8(18)	4930(2)	4429.0(17)	17.4(4)
C7	6834.9(19)	4551.3(19)	3104.0(18)	17.8(4)
C8	7588.9(19)	5773(2)	2450.0(18)	19.0(4)
C9	8326.0(19)	5190.9(19)	1314.0(18)	17.8(4)
C10	9596(2)	4378.2(19)	1624.8(19)	20.1(4)
C11	10246(2)	3760(2)	609(2)	21.4(4)

C12	9634(2)	3954(2)	-746.6(19)	21.8(4)
C13	831(2)	5910(2)	6312(2)	25.0(4)
C14	2048(2)	5030(2)	6415.5(19)	22.1(4)
C15	5476.2(19)	3912.6(19)	2252.4(18)	17.8(4)
C16	3826.0(19)	4962(2)	424.9(18)	19.2(4)
C17	2893(2)	6267(2)	91(2)	25.6(4)
C18	4728(2)	3170.9(19)	3319.9(17)	18.2(4)
C19	7735(2)	5387.3(19)	-44.7(19)	19.7(4)
C20	8383(2)	4774(2)	-1069.8(19)	22.7(4)

NMR Spectra of 1

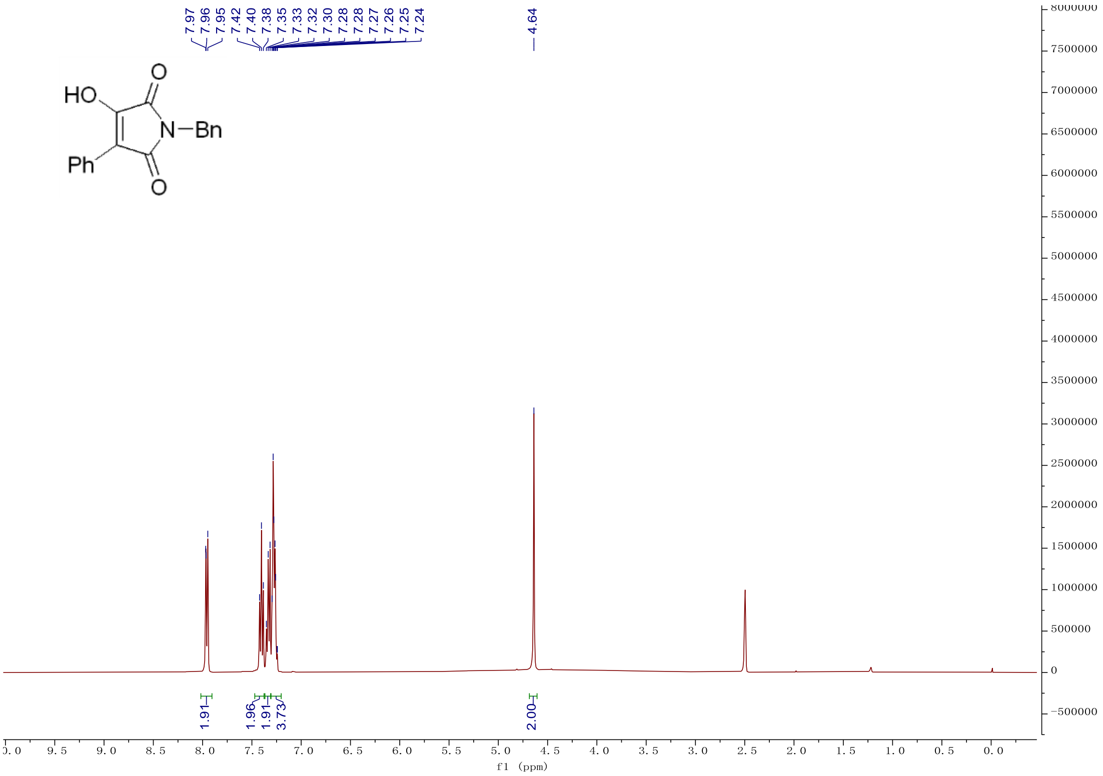

Supplementary Figure 13. ^{1}H NMR of 1a (400 MHz, DMSO-d_6).

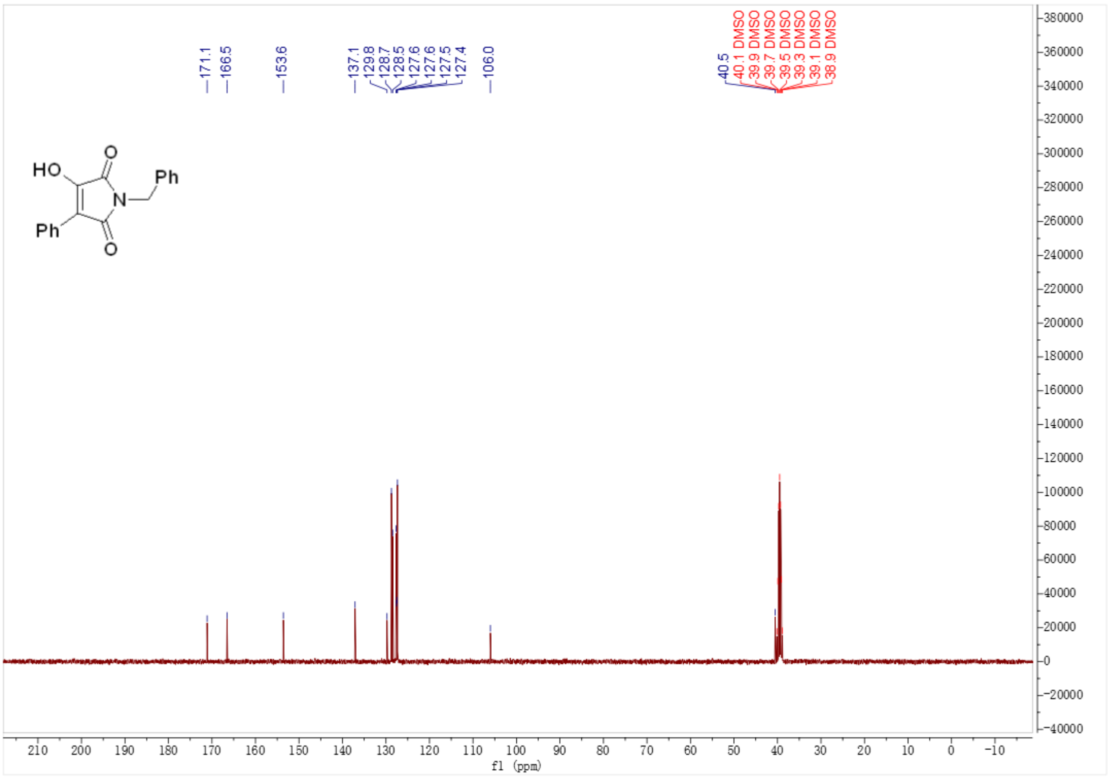

Supplementary Figure 14. ^{13}C NMR of 1a (101 MHz, DMSO-d_6)

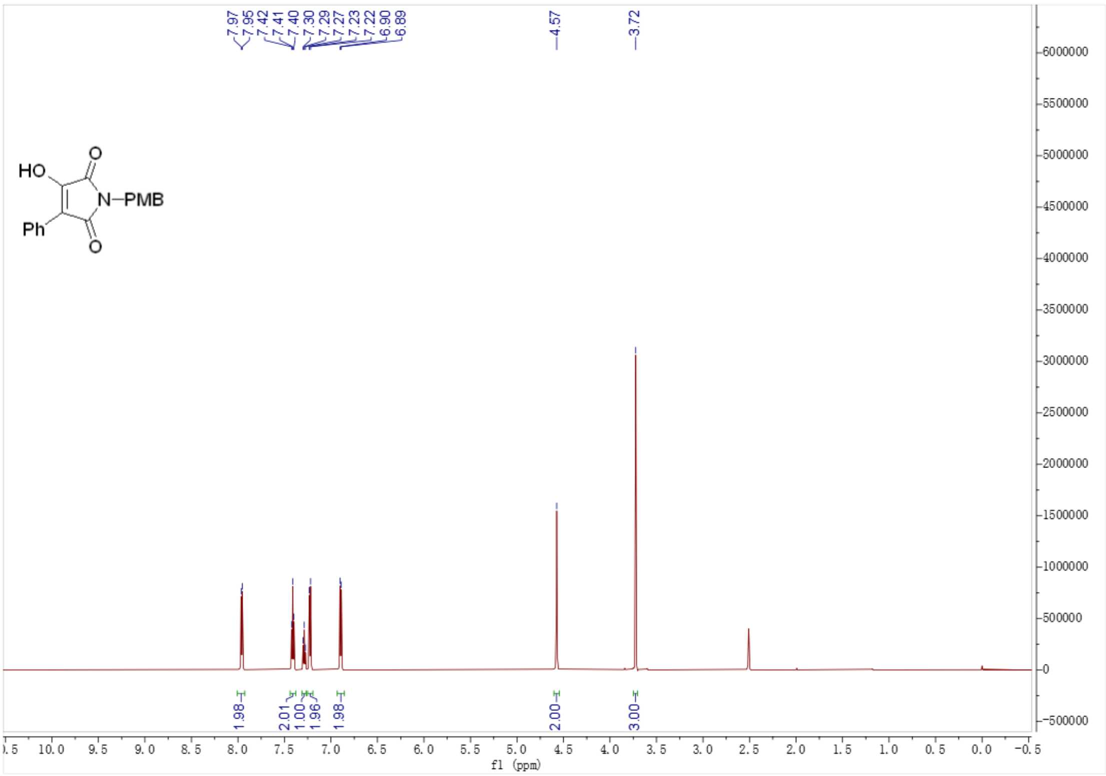

Supplementary Figure 15. ^{1}**H NMR of 1b (600 MHz, DMSO-*d*$_6$).**

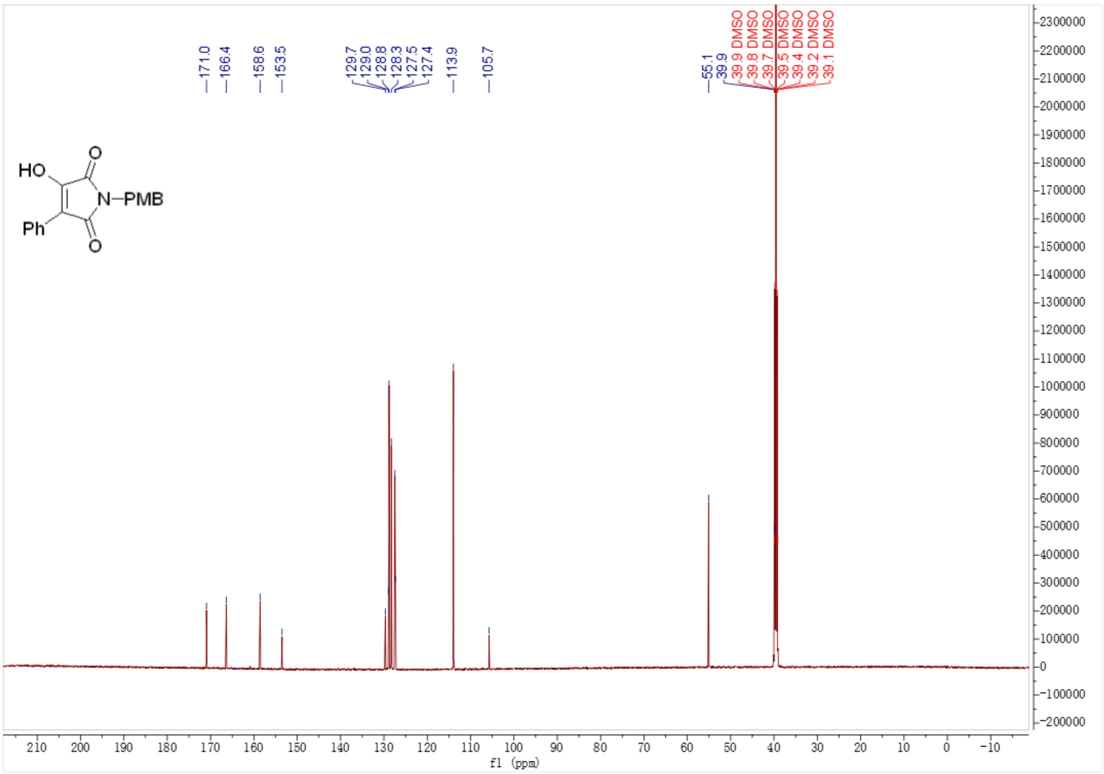

Supplementary Figure 16. ^{13}**C NMR of 1b (151 MHz, DMSO-*d*$_6$).**

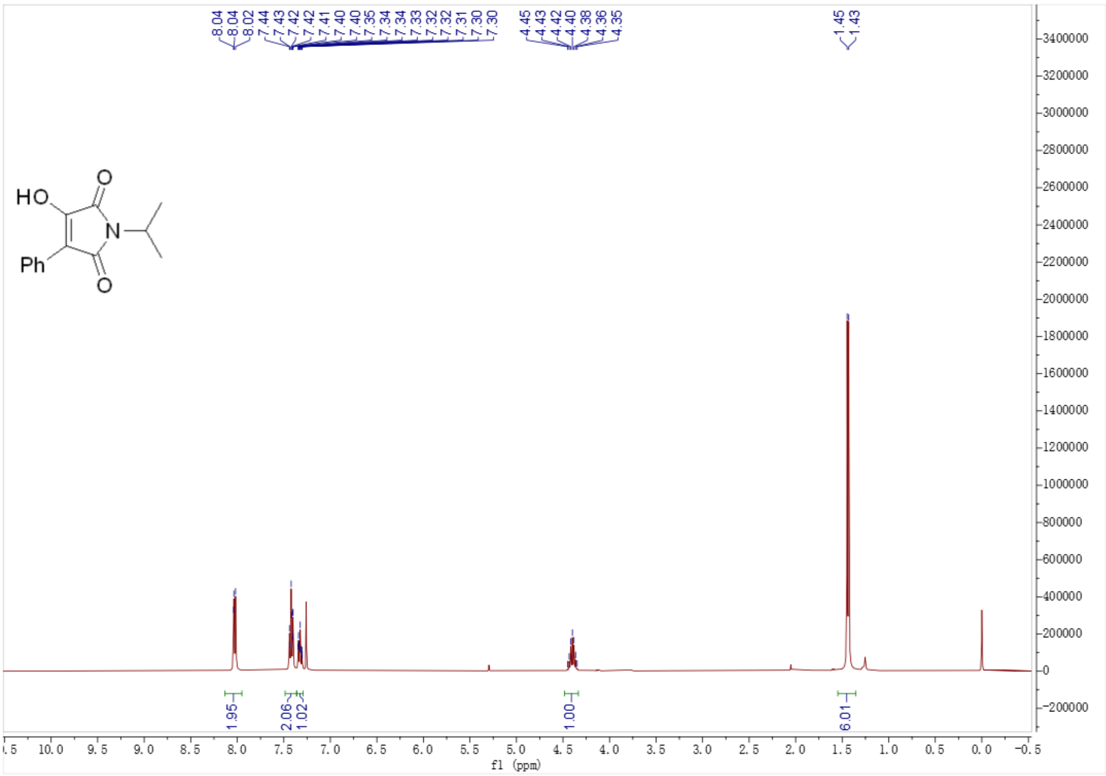

Supplementary Figure 17. ^{1}H NMR of 1c (400 MHz, Chloroform-*d*).

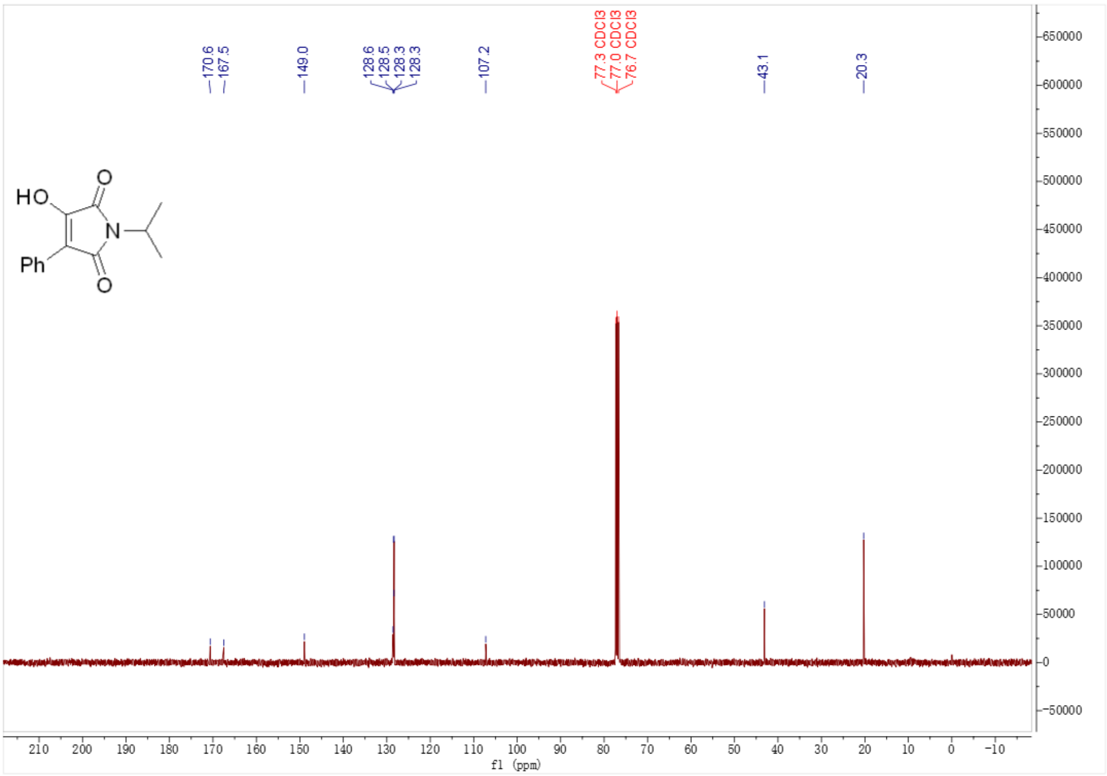

Supplementary Figure 18. ^{13}C NMR of 1c (101 MHz, Chloroform-*d*).

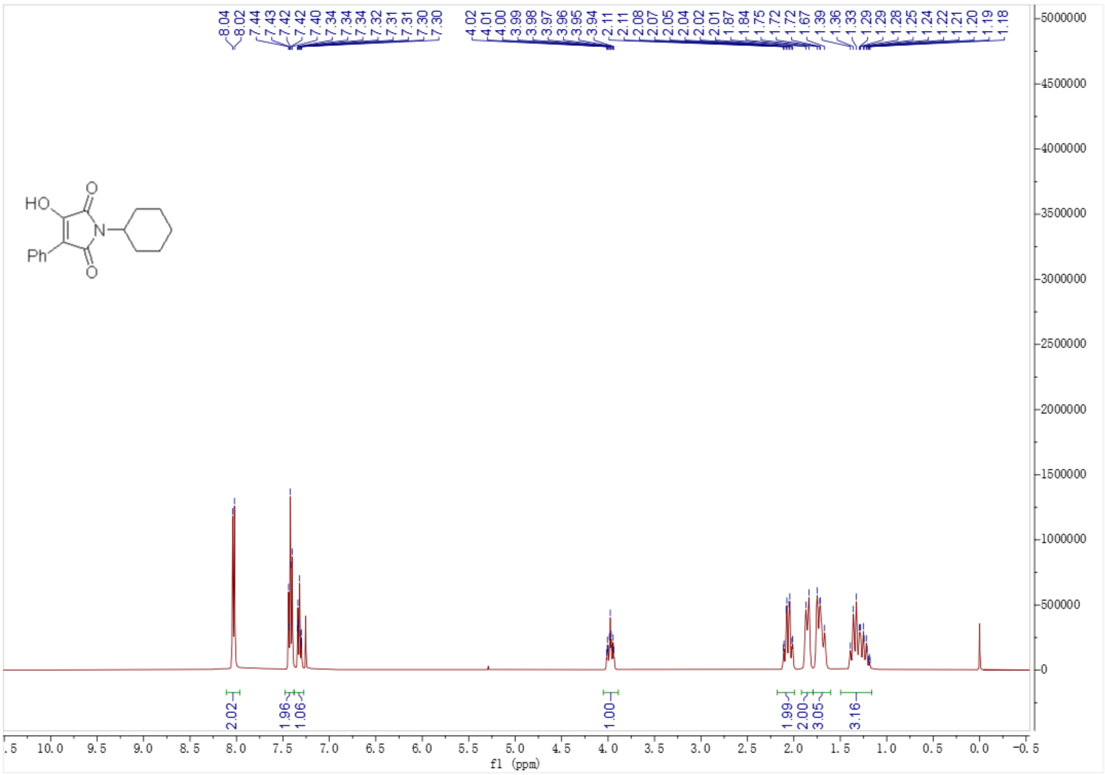

Supplementary Figure 19. ^{1}**H NMR of 1d (400 MHz, Chloroform-*d*).**

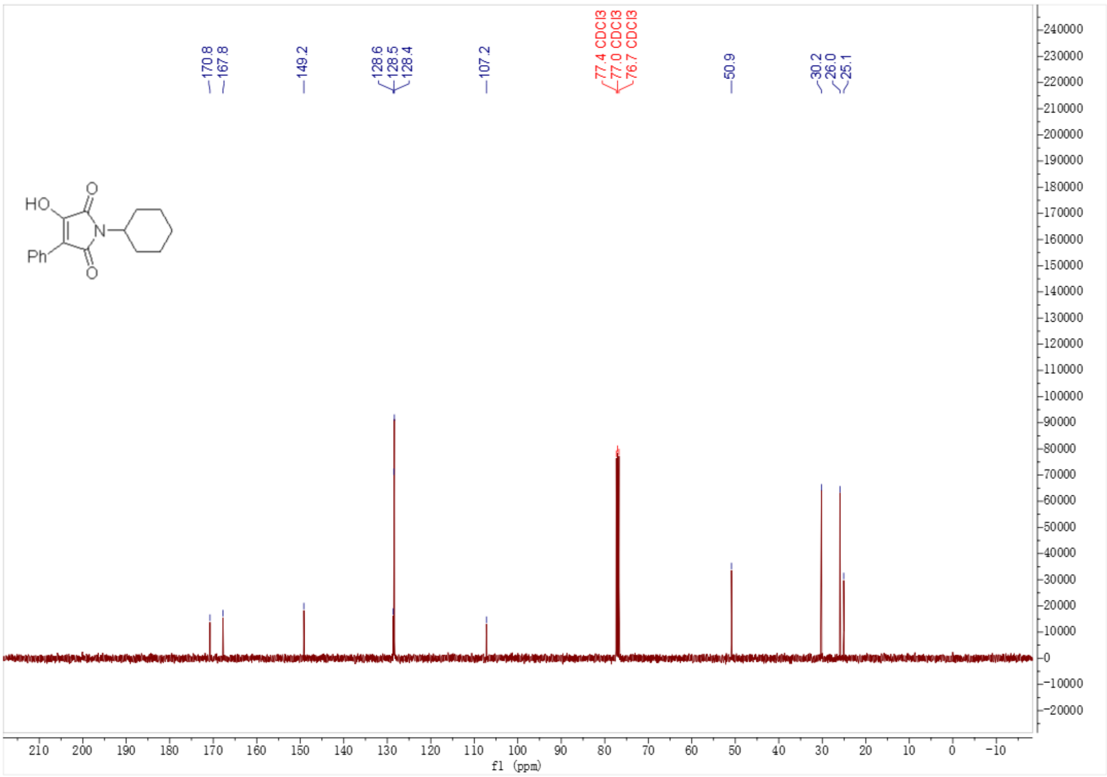

Supplementary Figure 20. ^{13}**C NMR of 1d (101 MHz, Chloroform-*d*).**

Supplementary Figure 21. ^{1}H NMR of 1e (400 MHz, Chloroform-*d*).

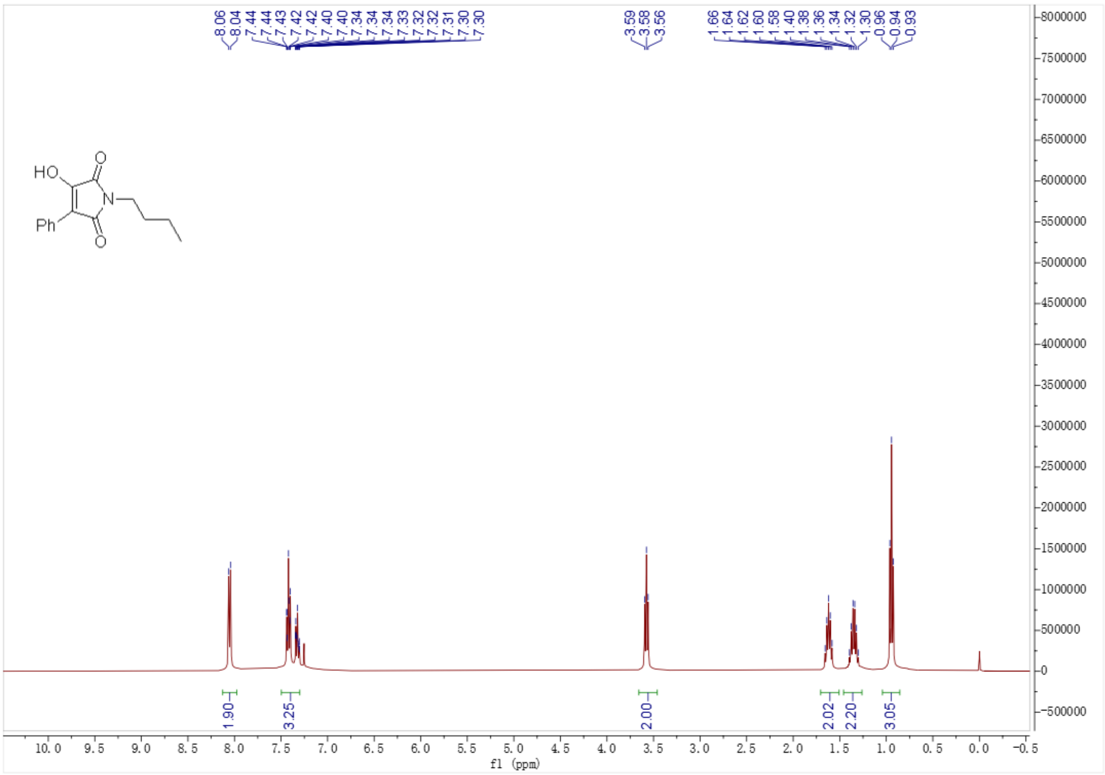

Supplementary Figure 22. ^{13}C NMR of 1e (101 MHz, Chloroform-*d*).

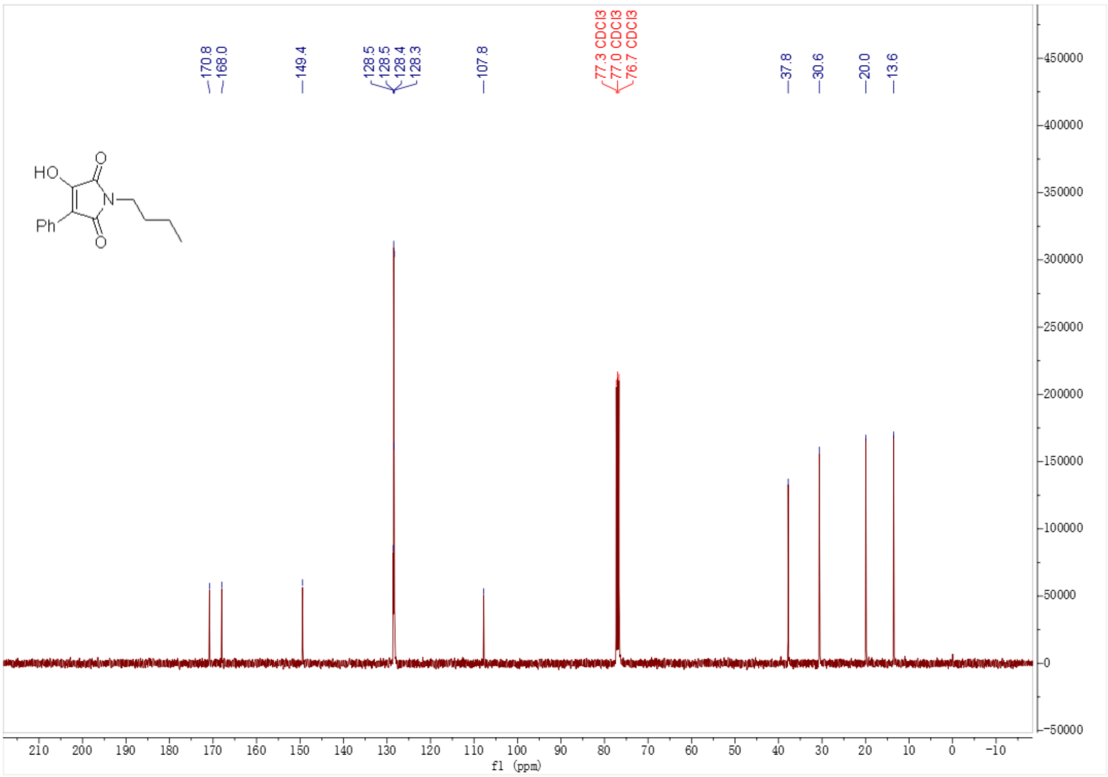

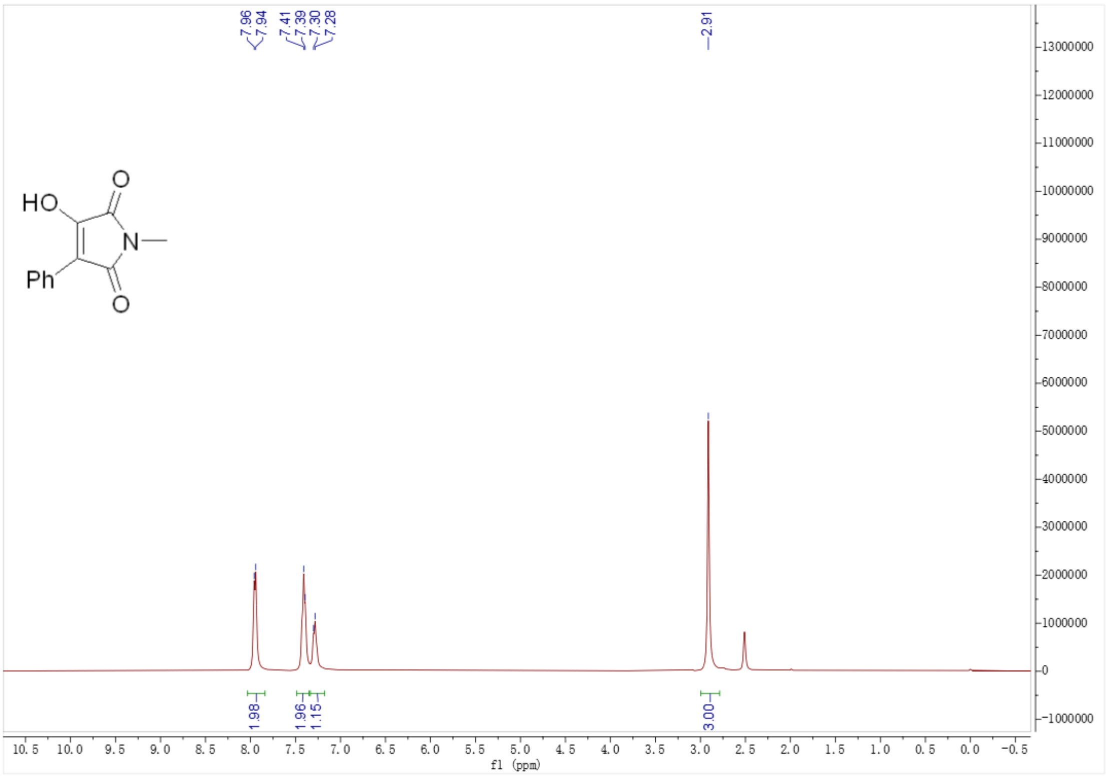

Supplementary Figure 23. **^{1}H NMR of 1f (400 MHz, DMSO-d_6).**

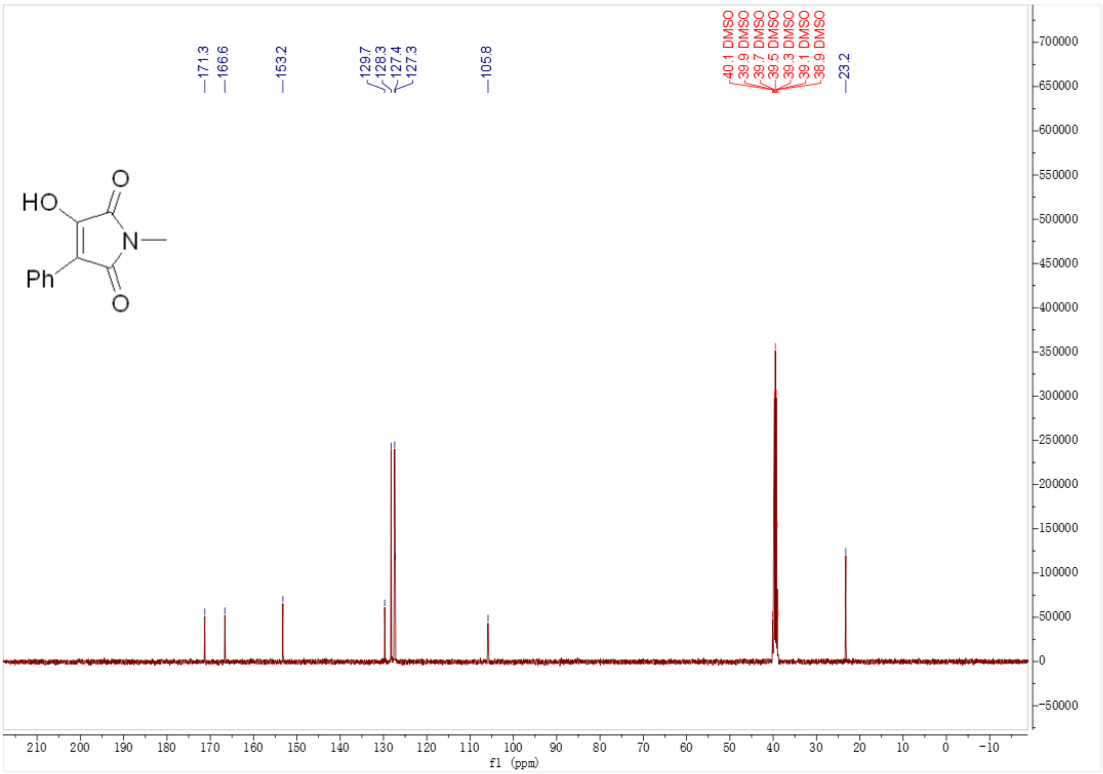

Supplementary Figure 24. **^{13}C NMR of 1f (101 MHz, DMSO-d_6).**

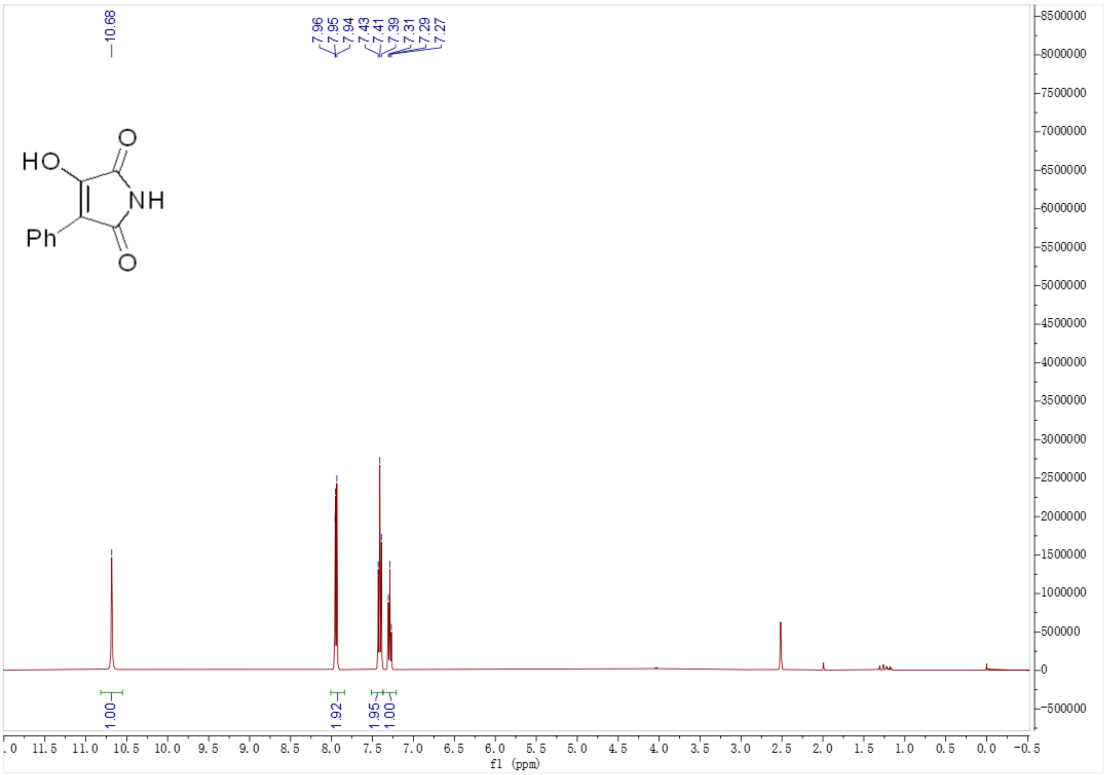

Supplementary Figure 25. ^{1}H **NMR of 1g (400 MHz, DMSO-*d*$_6$).**

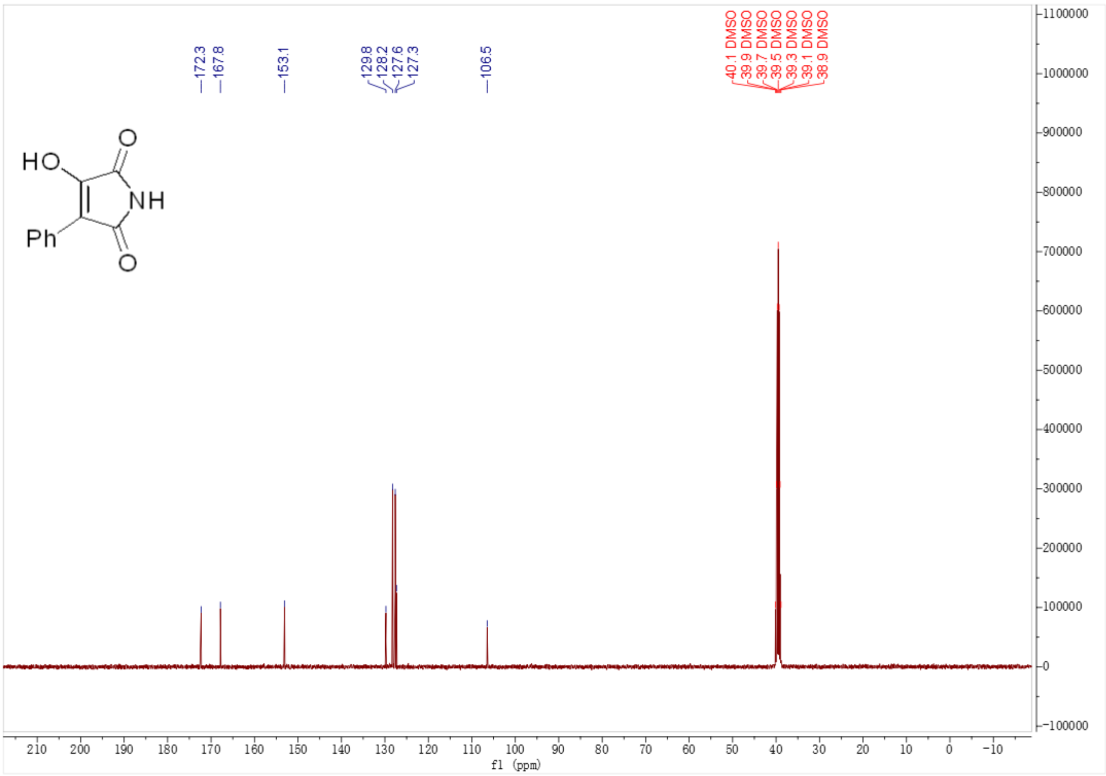

Supplementary Figure 26. ^{13}C **NMR of 1g (101 MHz, DMSO-*d*$_6$).**

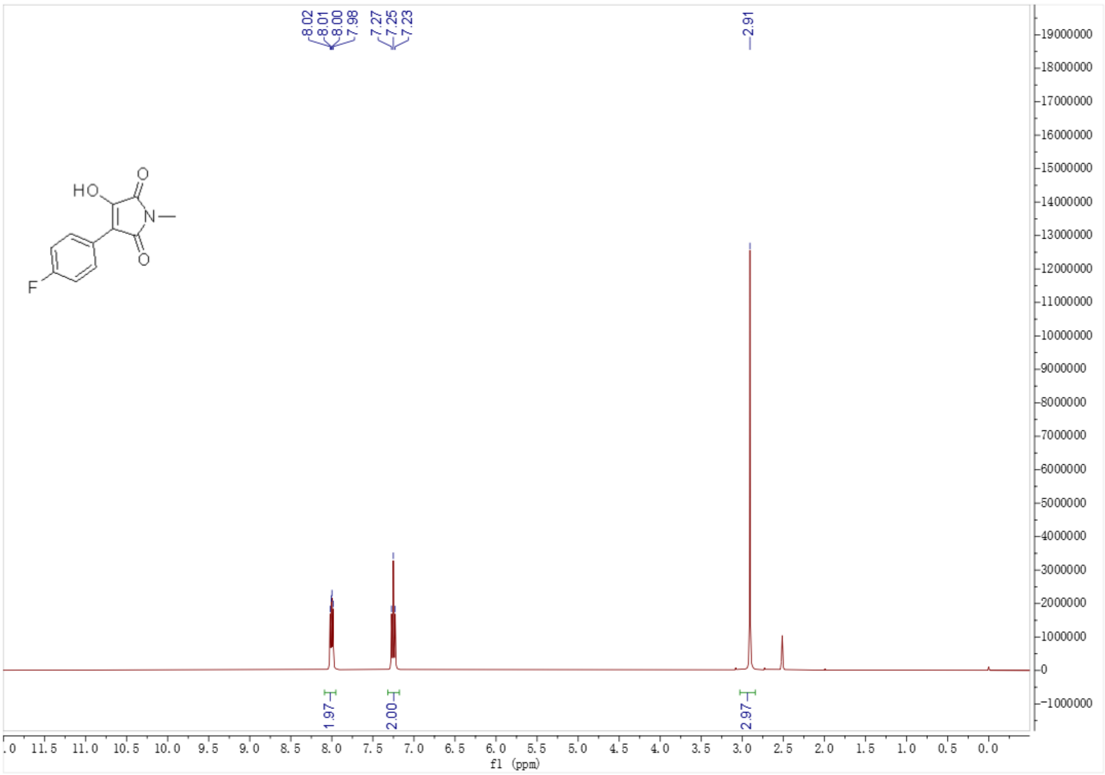

Supplementary Figure 27. ^{1}H NMR of 1h (400 MHz, DMSO-d_6).

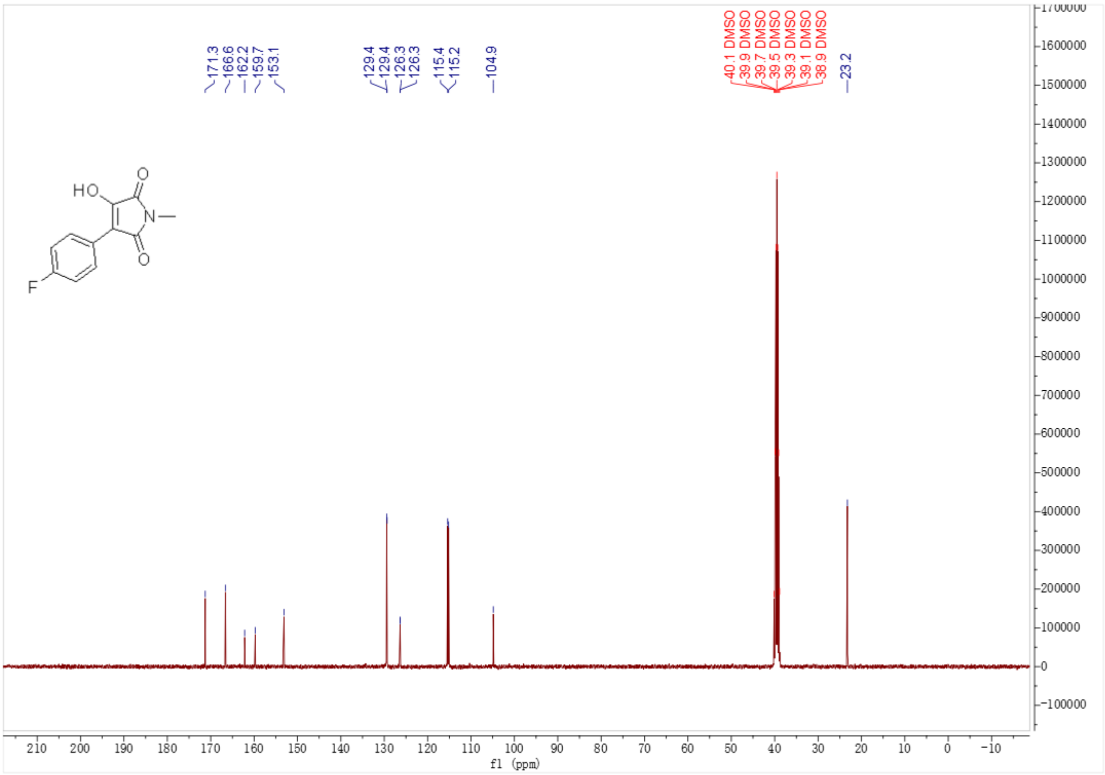

Supplementary Figure 28. ^{13}C NMR of 1h (101 MHz, DMSO-d_6).

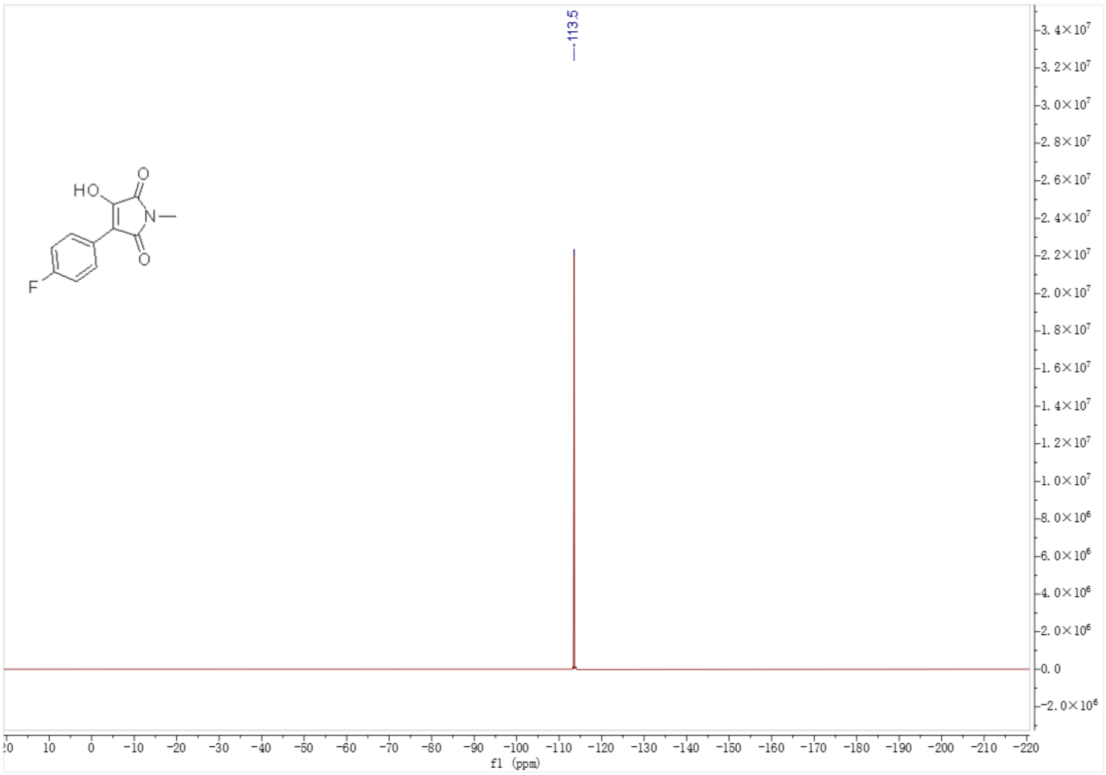

Supplementary Figure 29. ^{19}F NMR of 1h (376 MHz, DMSO-d_6).

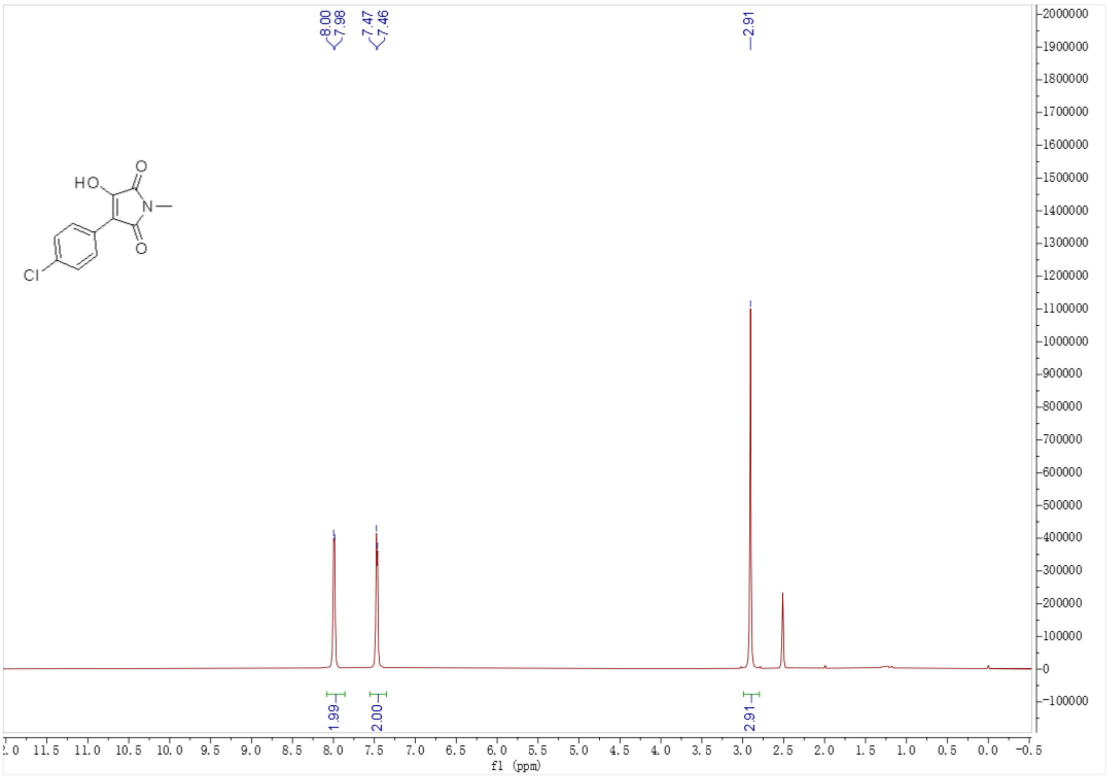

Supplementary Figure 30. ^{1}H NMR of 1i (600 MHz, DMSO-d_6).

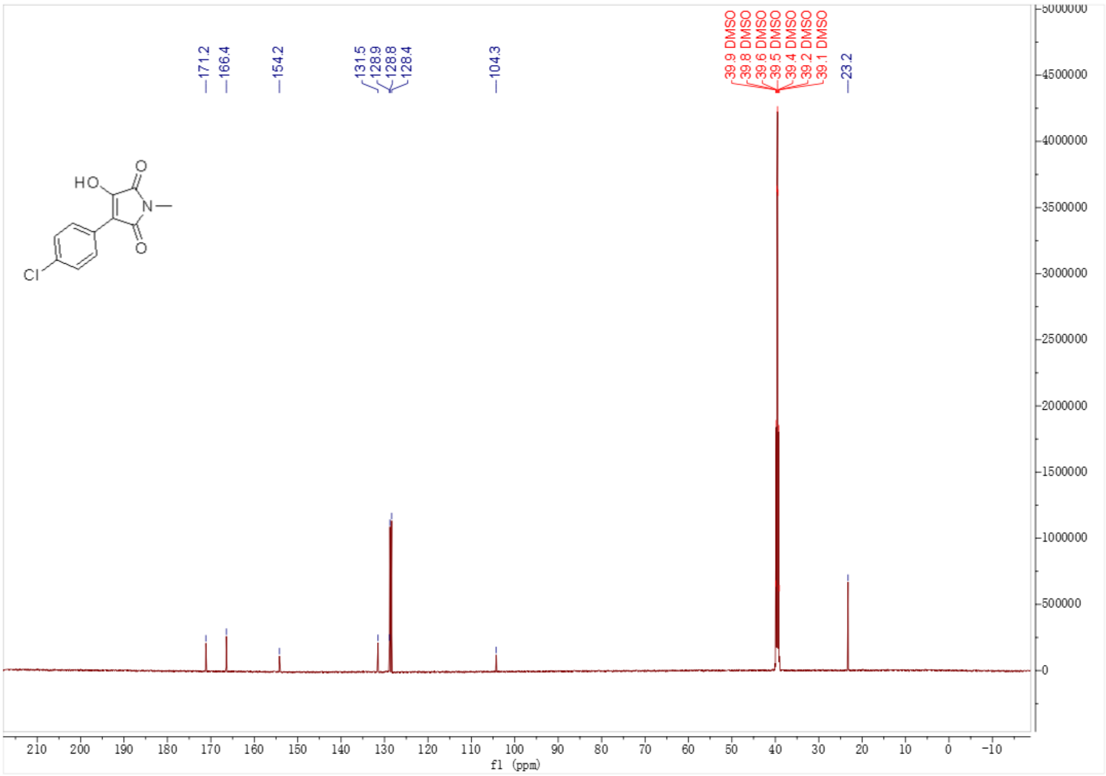

Supplementary Figure 31. ^{13}C NMR of 1i (151 MHz, DMSO-d_6).

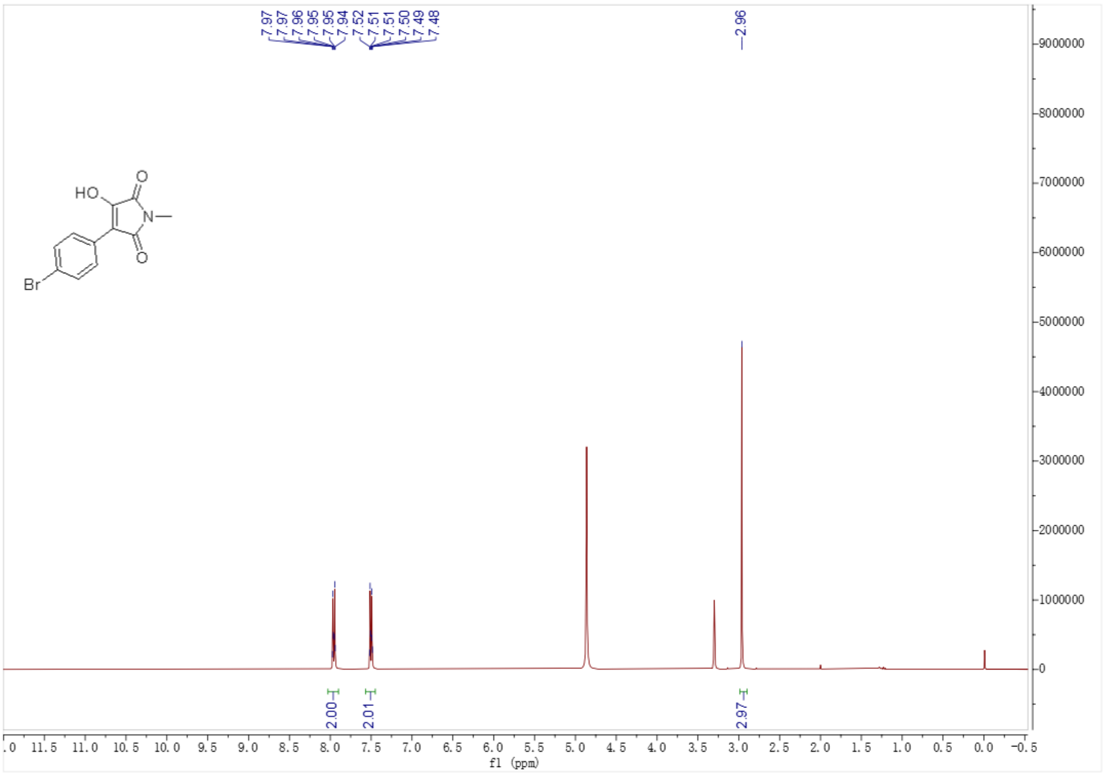

Supplementary Figure 32. ^{1}H NMR of 1j (400 MHz, Methanol-d_4).

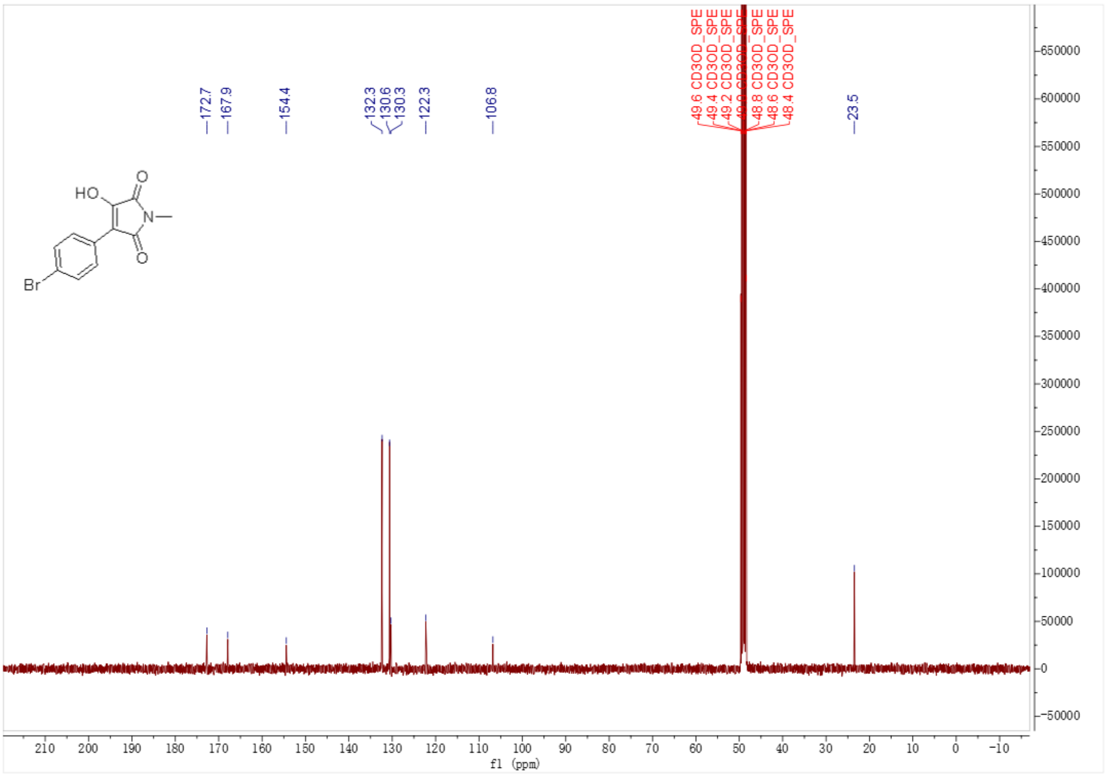

Supplementary Figure 33. ^{13}C NMR of 1j (101 MHz, Methanol-d_4).

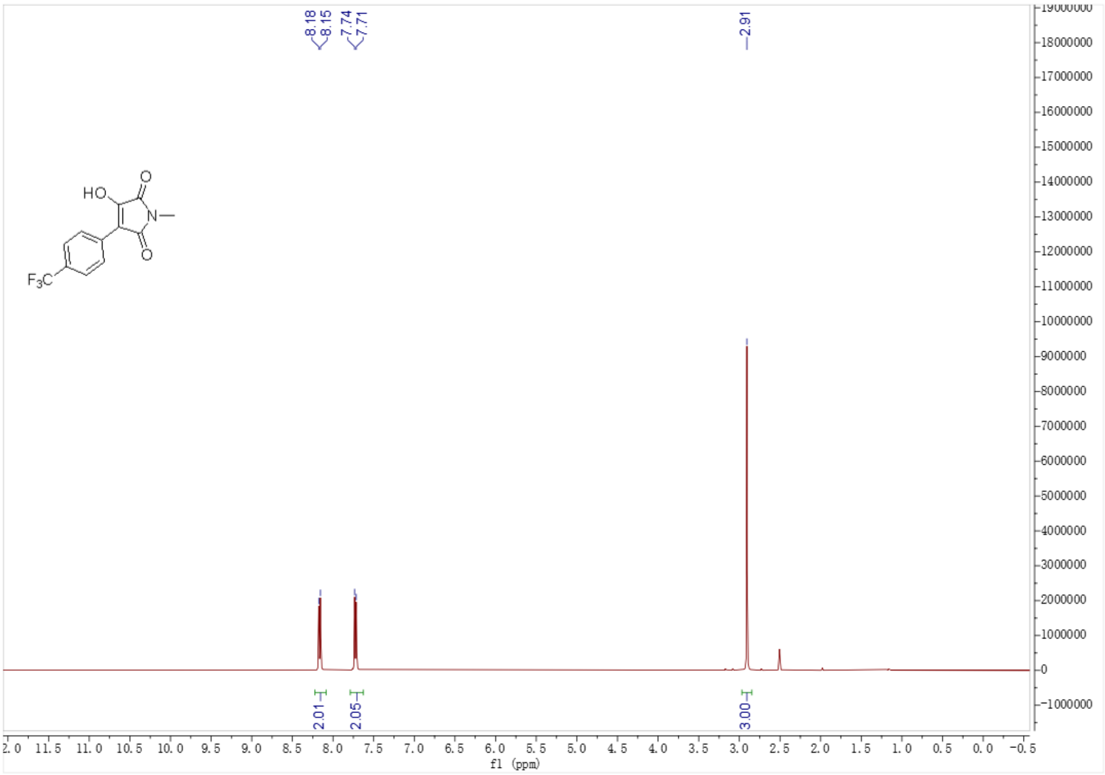

Supplementary Figure 34. ^{1}**H NMR of 1k (400 MHz, DMSO-d_6).**

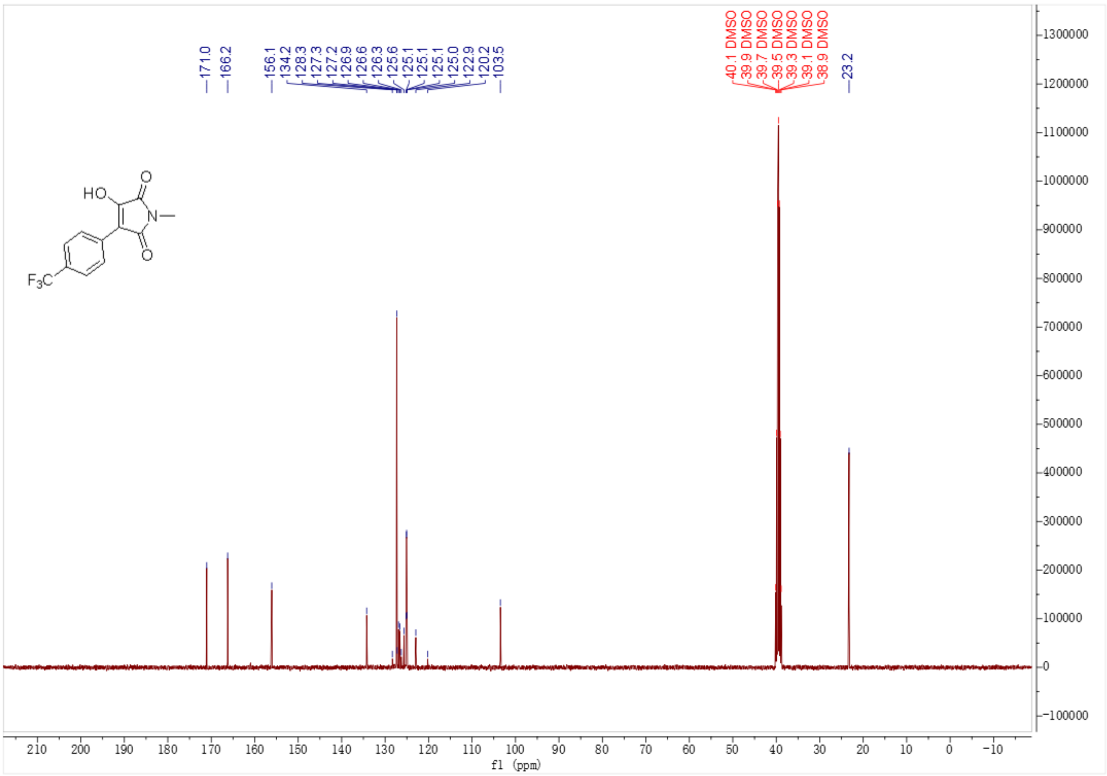

Supplementary Figure 35. ^{13}**C NMR of 1k (101 MHz, DMSO-d_6).**

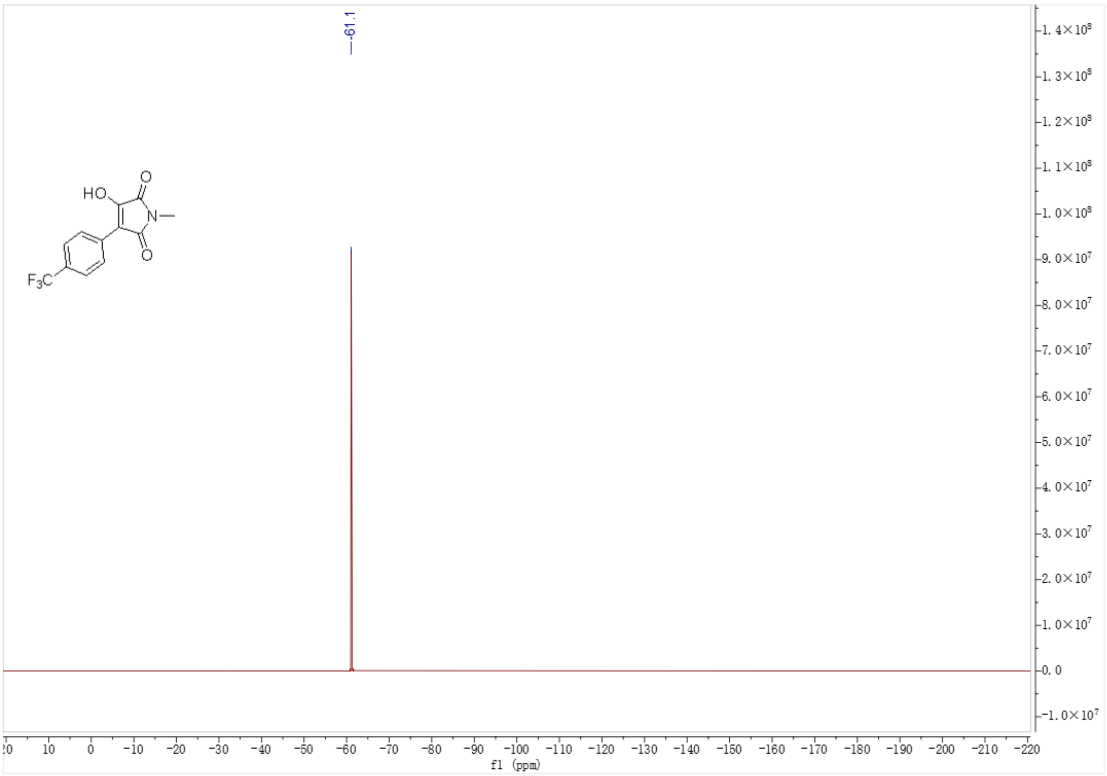

Supplementary Figure 36. ^{19}F NMR of 1k (376 MHz, DMSO-d_6).

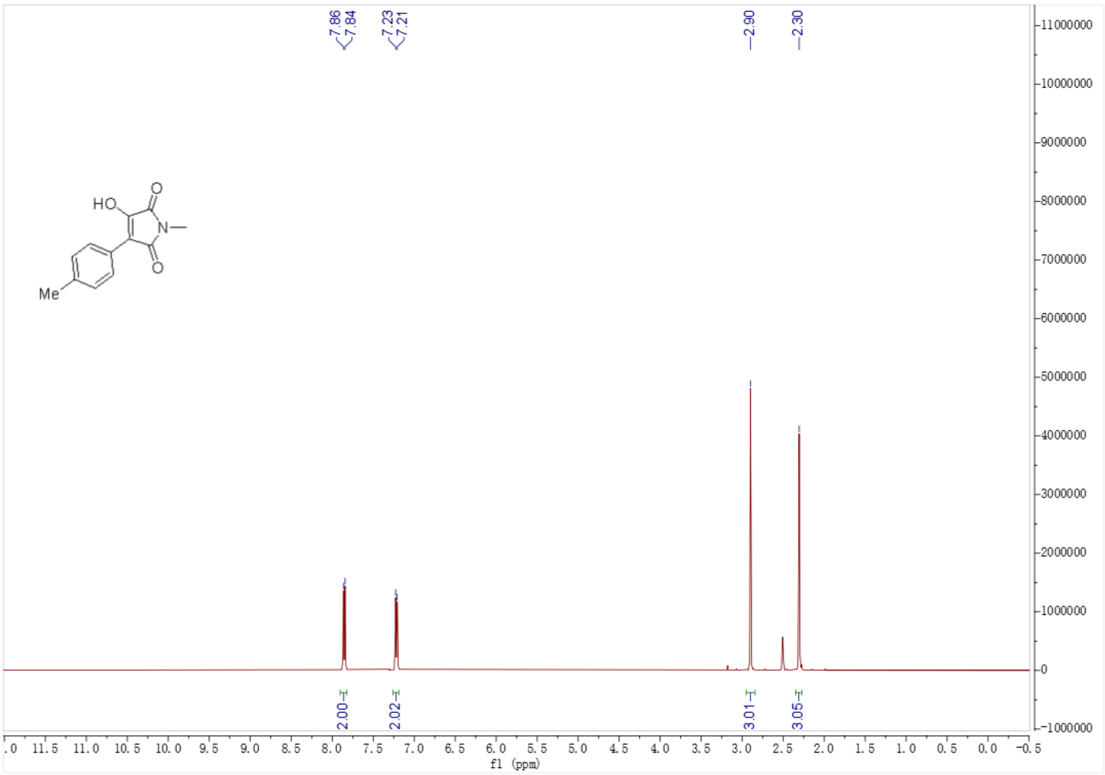

Supplementary Figure 37. ^{1}H NMR of 1l (400 MHz, DMSO-d_6).

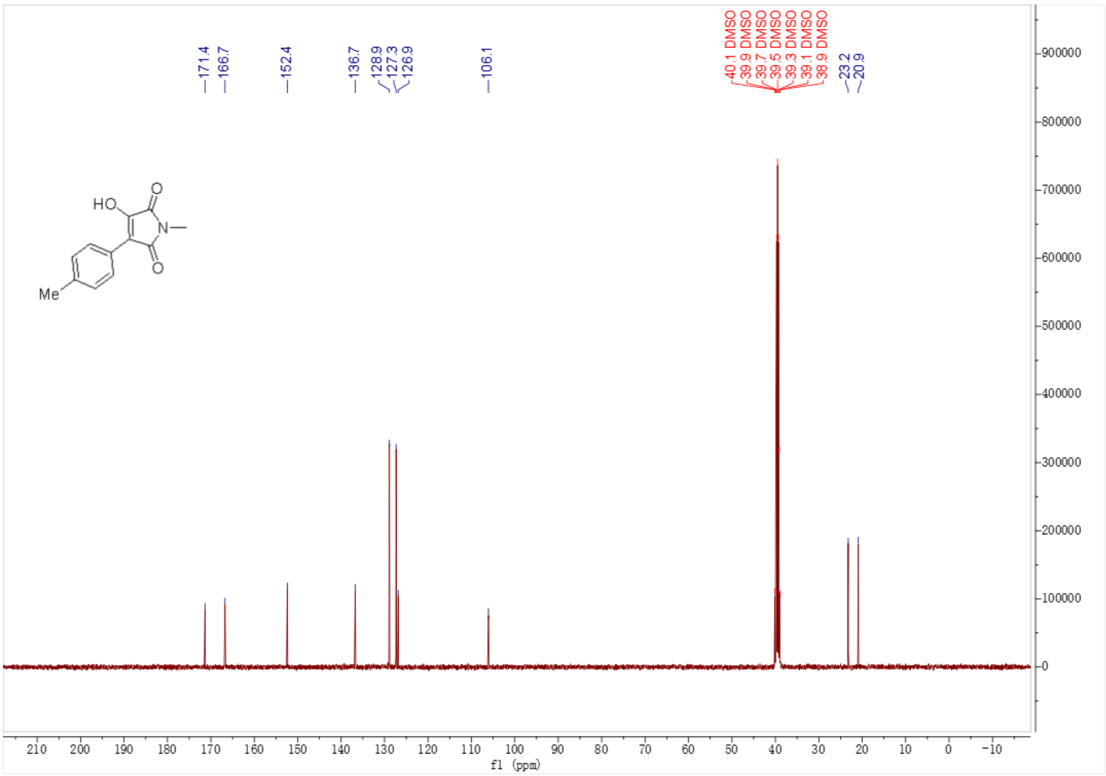

Supplementary Figure 38. ^{13}C NMR of 1l (101 MHz, DMSO-d_6).

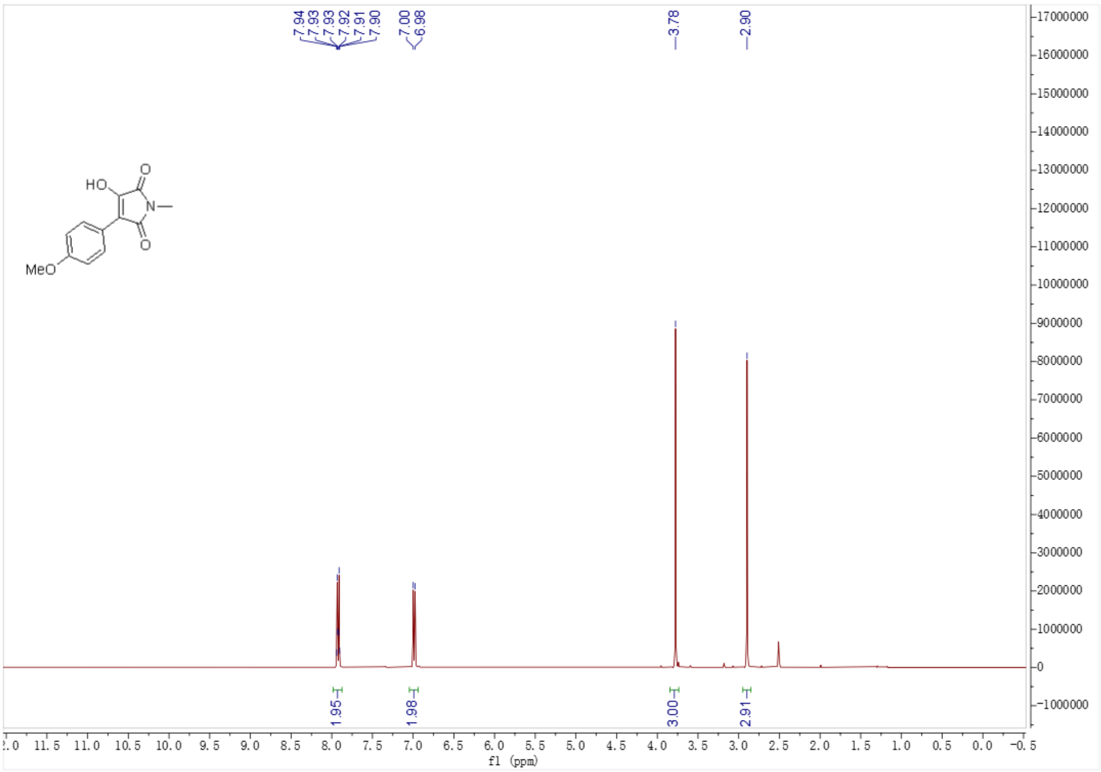

Supplementary Figure 39. ^{1}**H NMR of 1m (400 MHz, DMSO-d_6).**

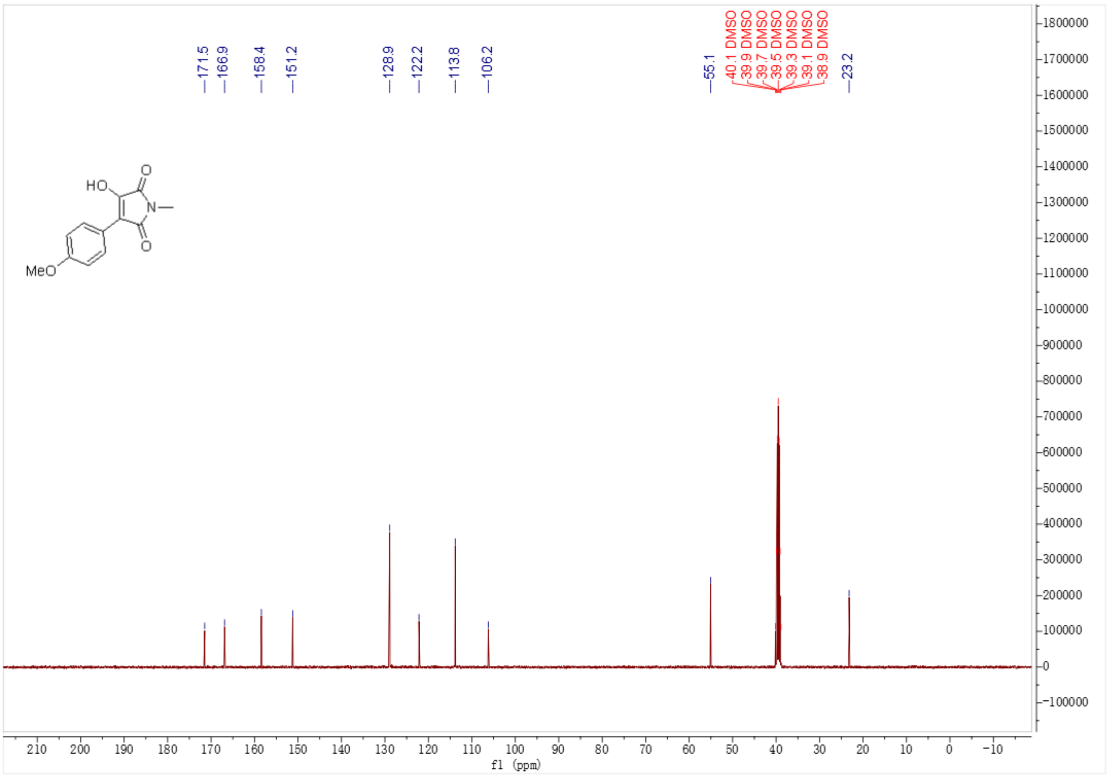

Supplementary Figure 40. ^{13}**C NMR of 1m (101 MHz, DMSO-d_6).**

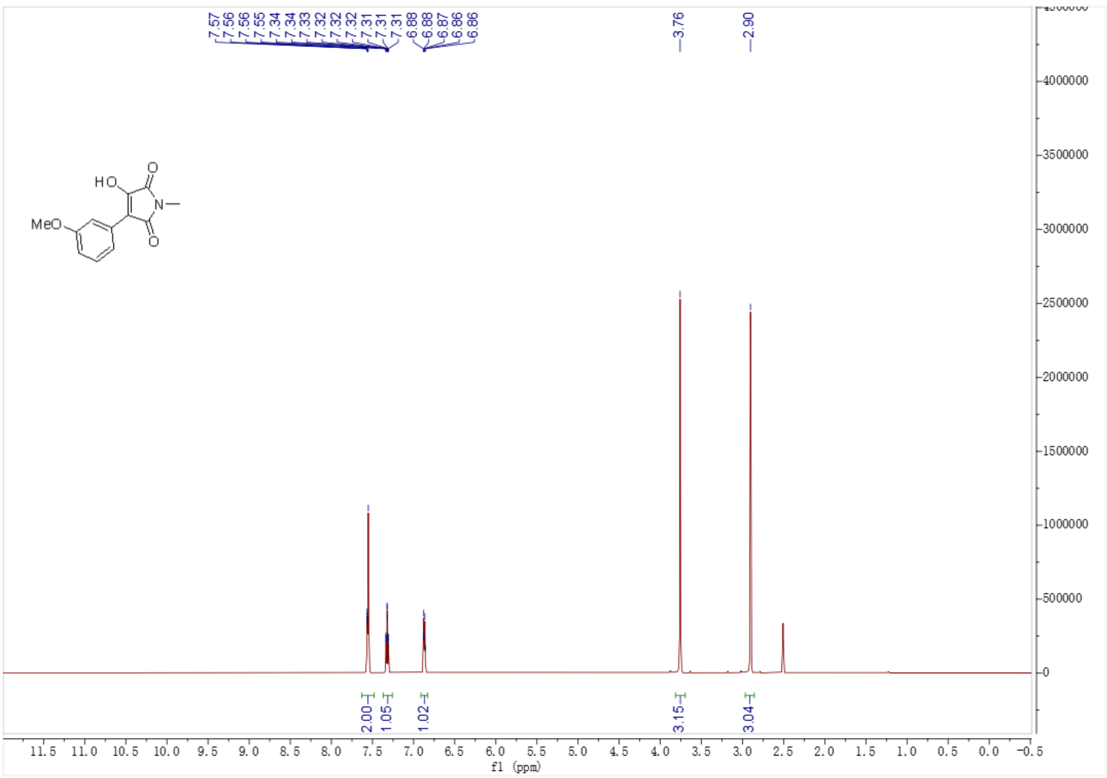

Supplementary Figure 41. ^{1}H NMR of 1n (600 MHz, DMSO-d_6).

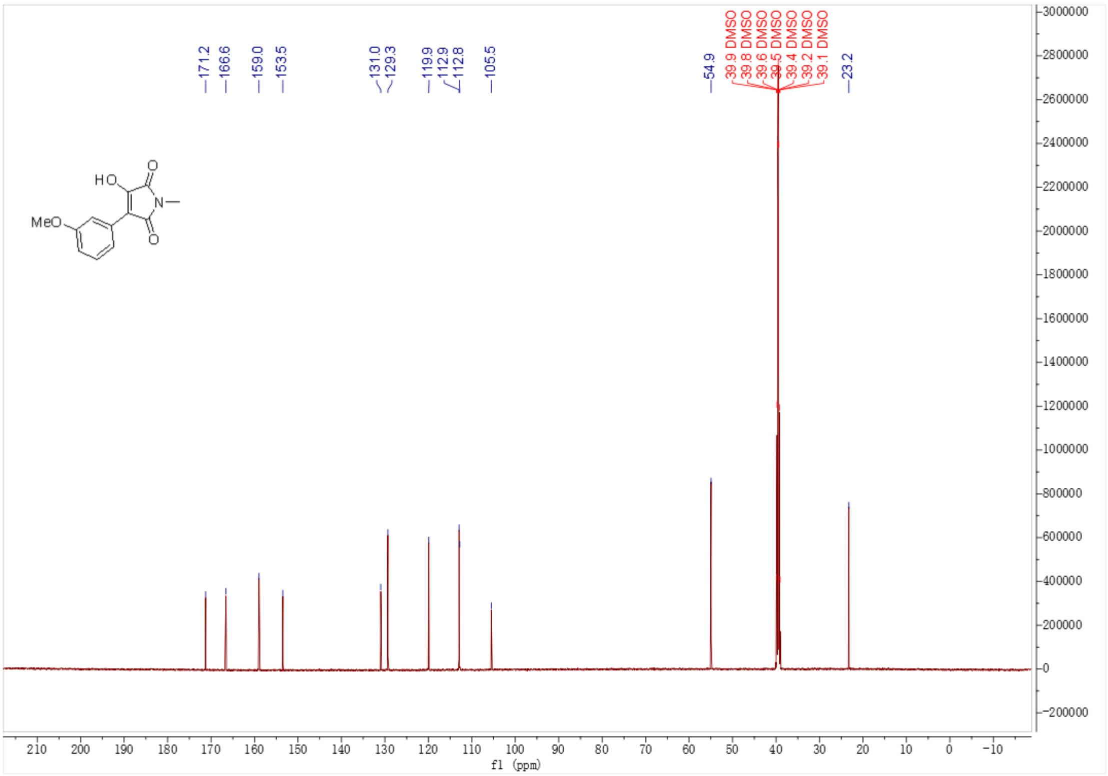

Supplementary Figure 42. ^{13}C NMR of 1n (151 MHz, DMSO-d_6).

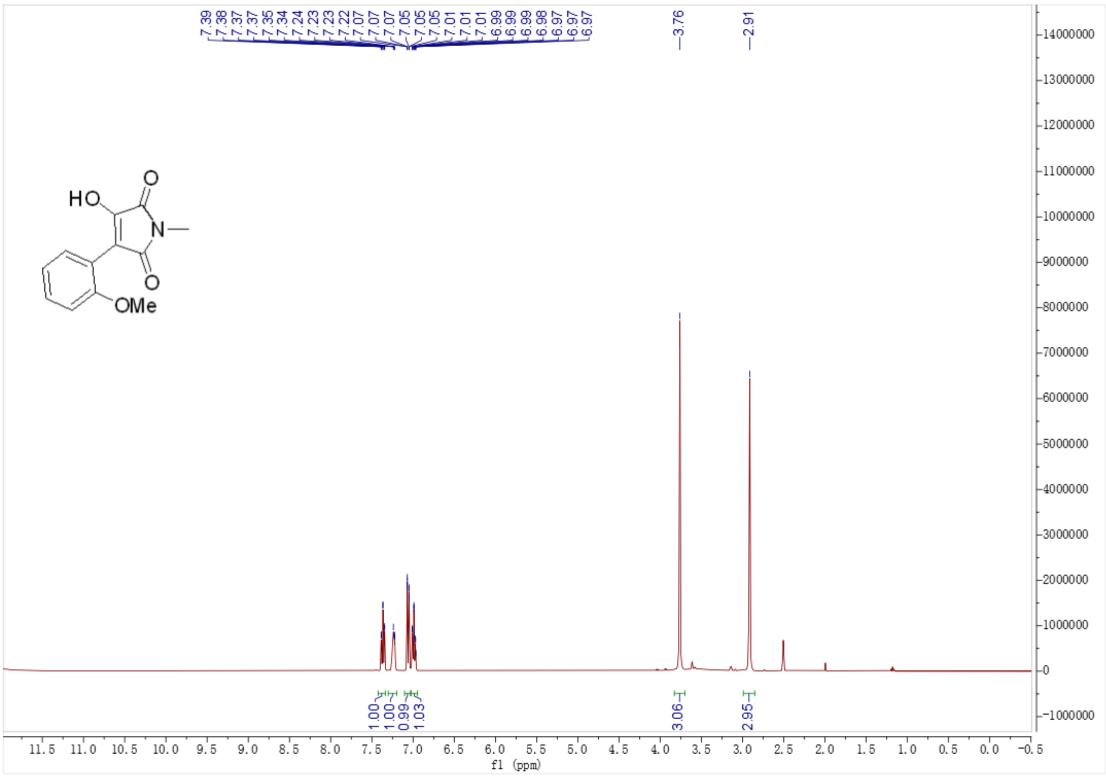

Supplementary Figure 42. ^{1}H NMR of 1o (400 MHz, DMSO-d_6).

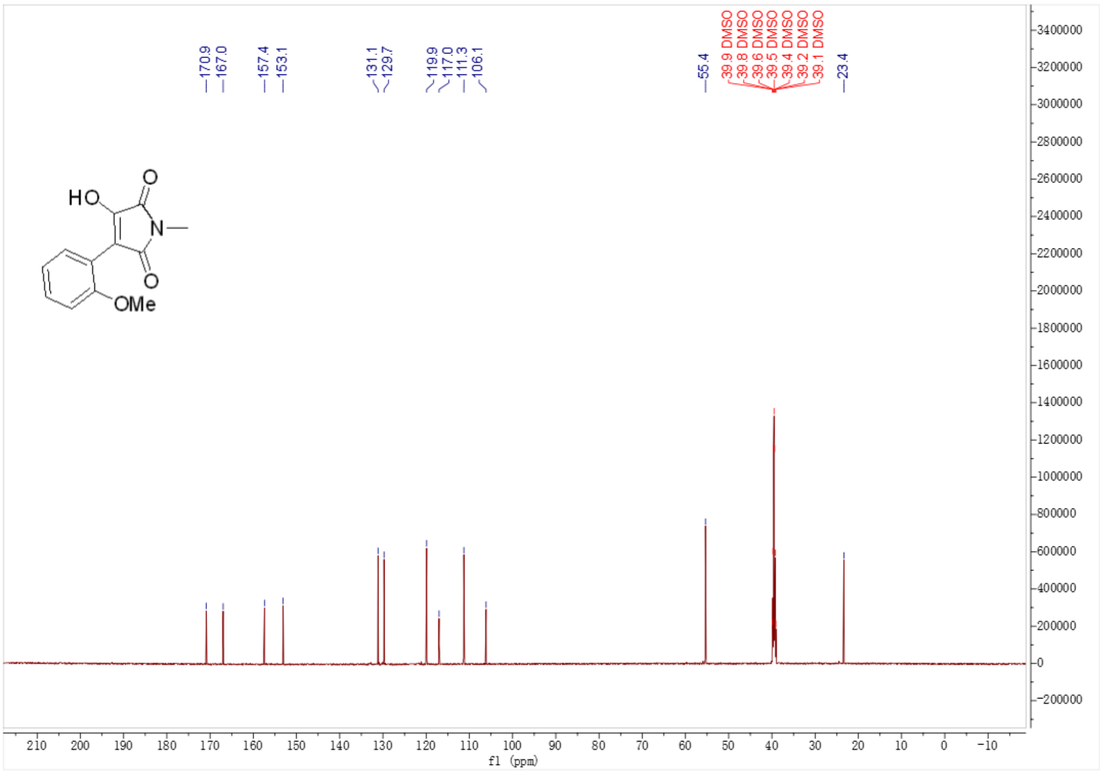

Supplementary Figure 43. ^{13}C NMR of 1o (151 MHz, DMSO-d_6).

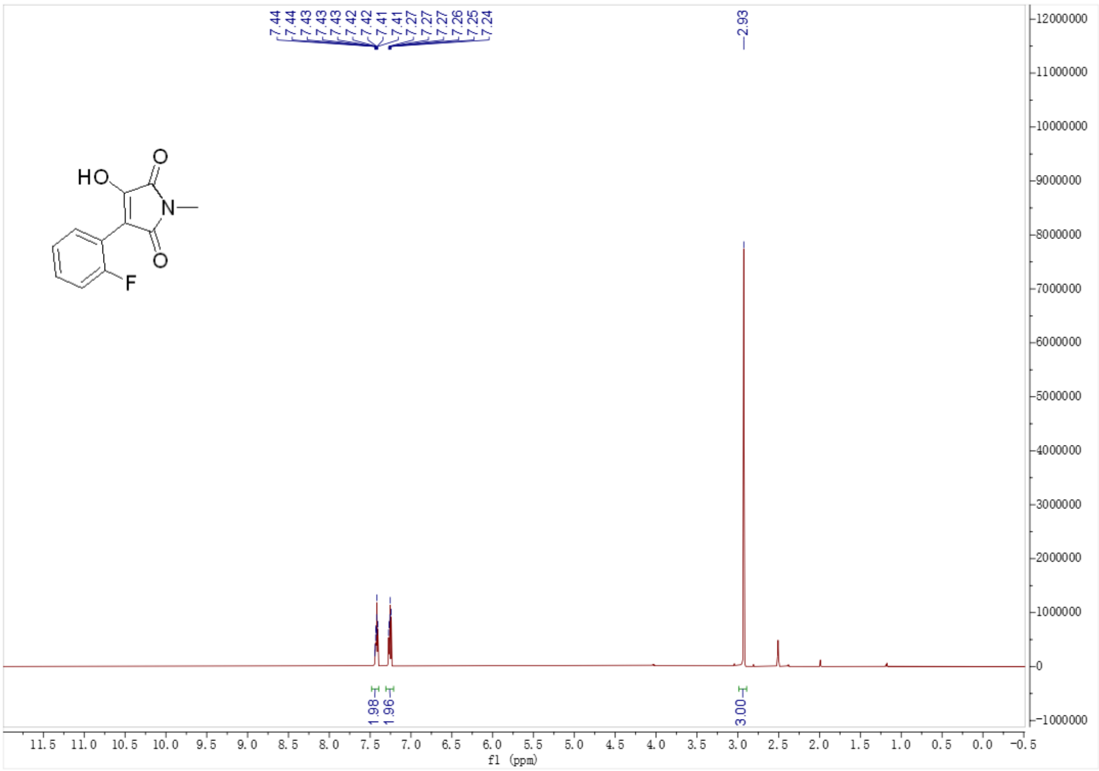

Supplementary Figure 44. ^{1}**H NMR of 1p (600 MHz, DMSO-*d*$_6$).**

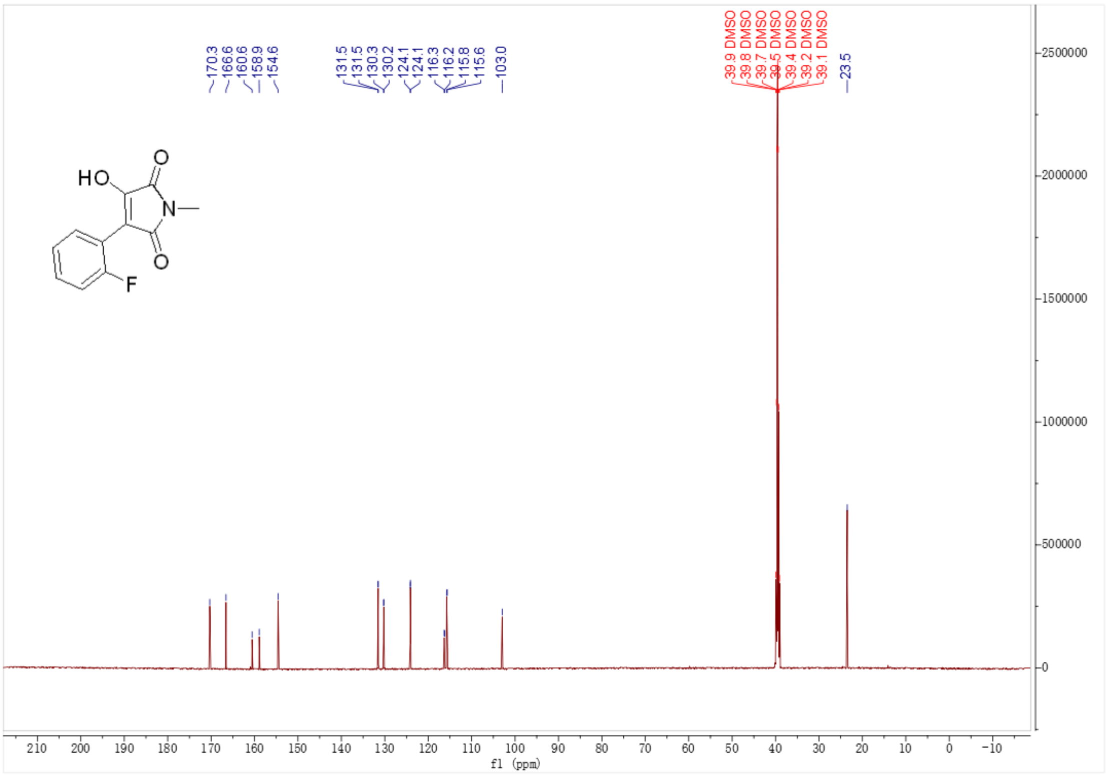

Supplementary Figure 45. ^{13}**C NMR of 1p (151 MHz, DMSO-*d*$_6$).**

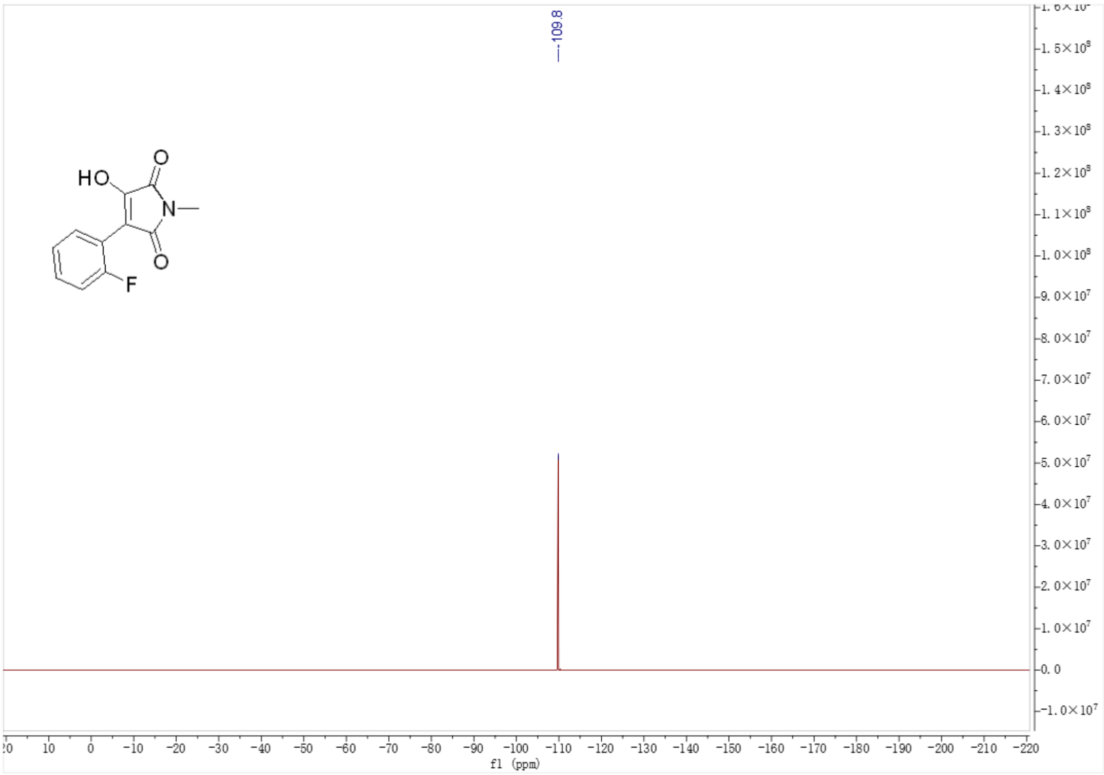

Supplementary Figure 46. ^{19}F NMR of 1p (376 MHz, DMSO-d_6).

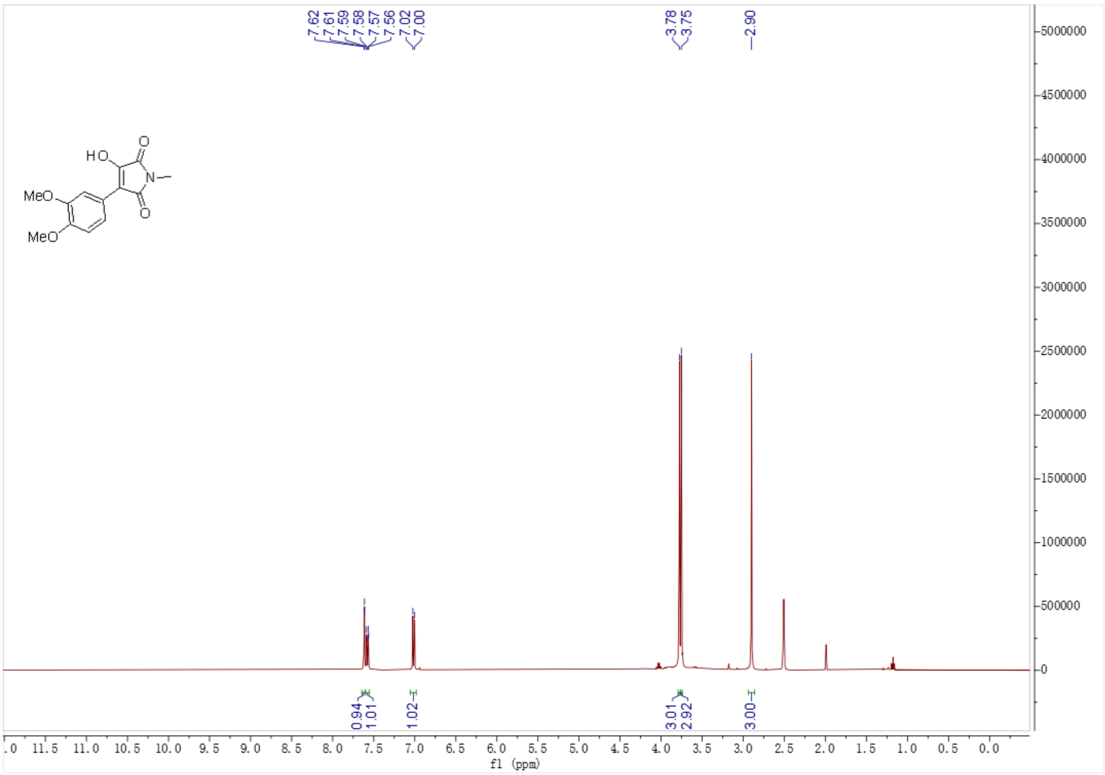

Supplementary Figure 47. ^{1}H NMR of 1q (400 MHz, DMSO-d_6).

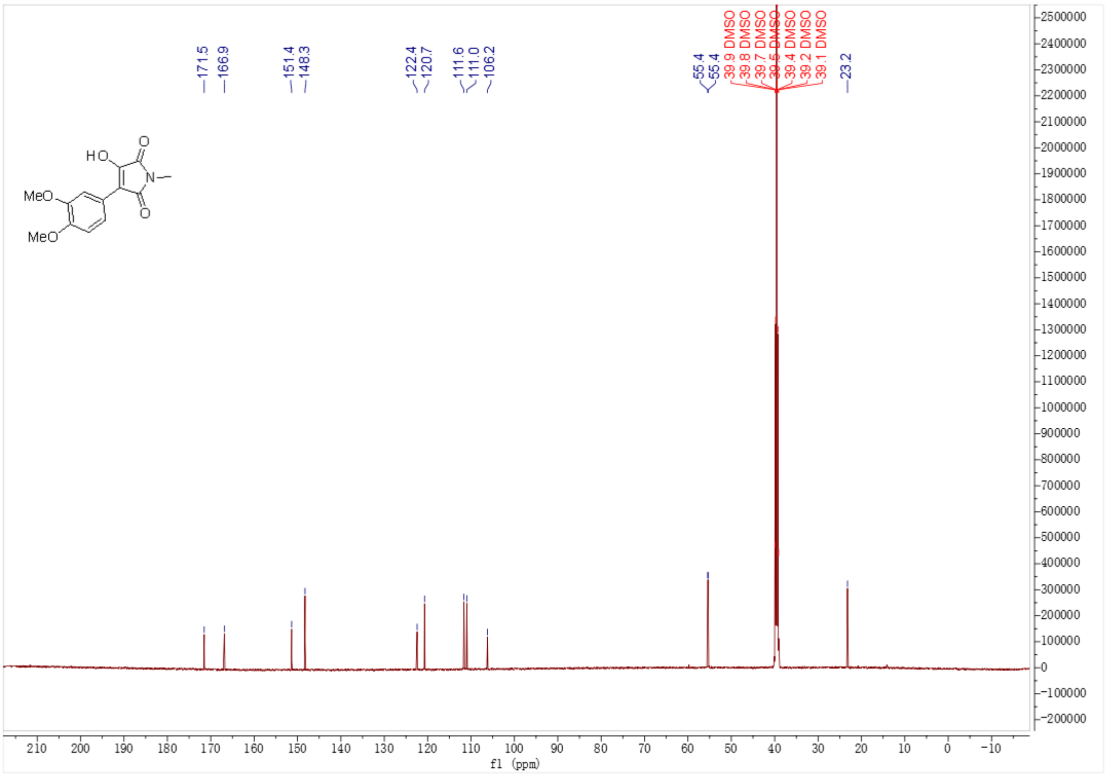

Supplementary Figure 48. ^{13}**C NMR of 1q (151 MHz, DMSO-*d*$_6$).**

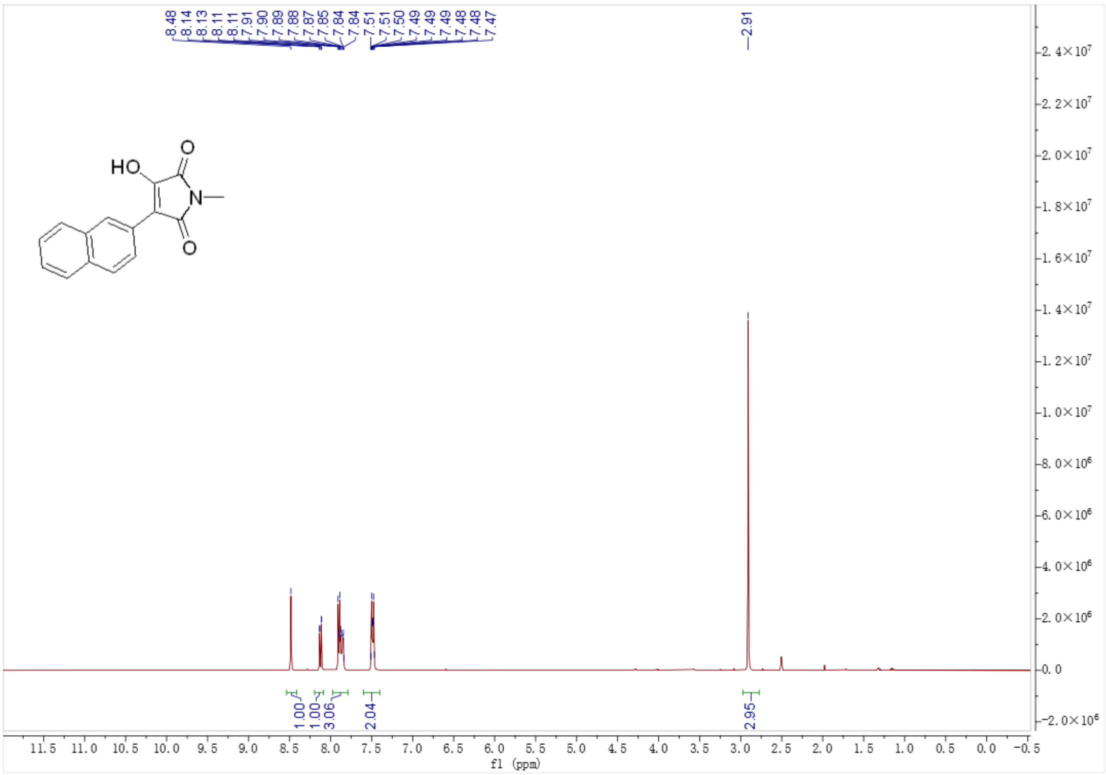

Supplementary Figure 49. ^{1}**H NMR of 1r (400 MHz, DMSO-*d*$_6$).**

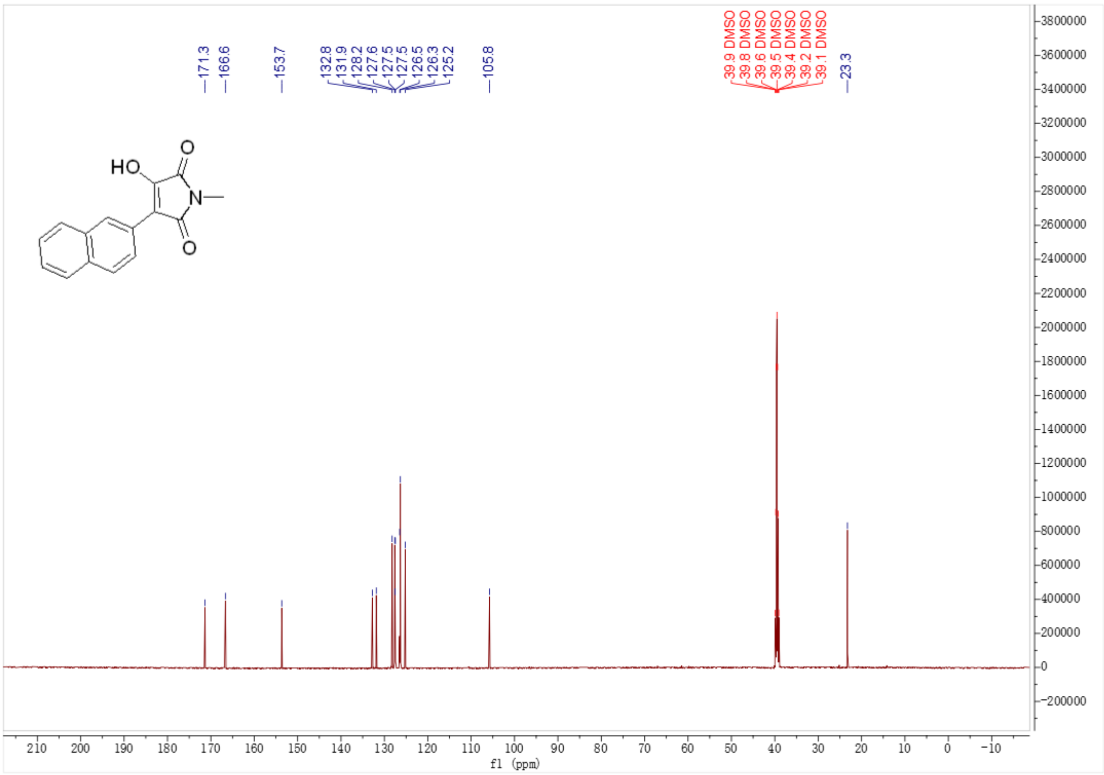

Supplementary Figure 50. ^{13}C NMR of 1r (151 MHz, DMSO-d_6).

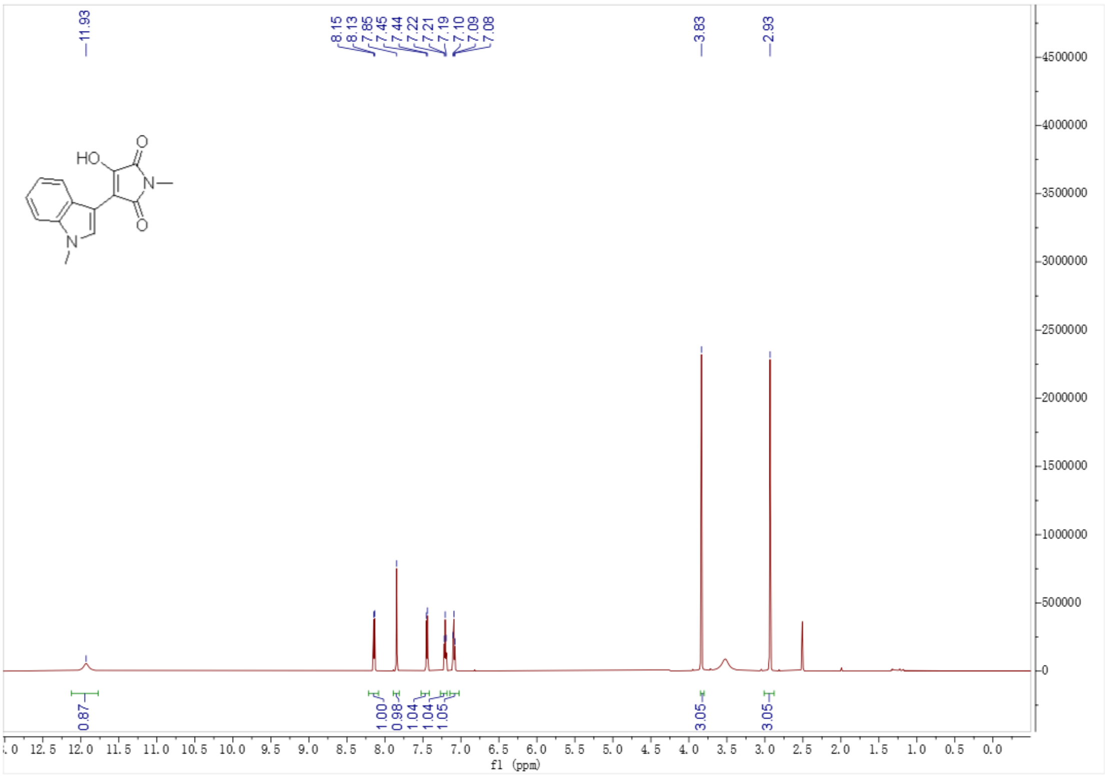

Supplementary Figure 51. ^{1}H NMR of 1s (600 MHz, DMSO-d_6).

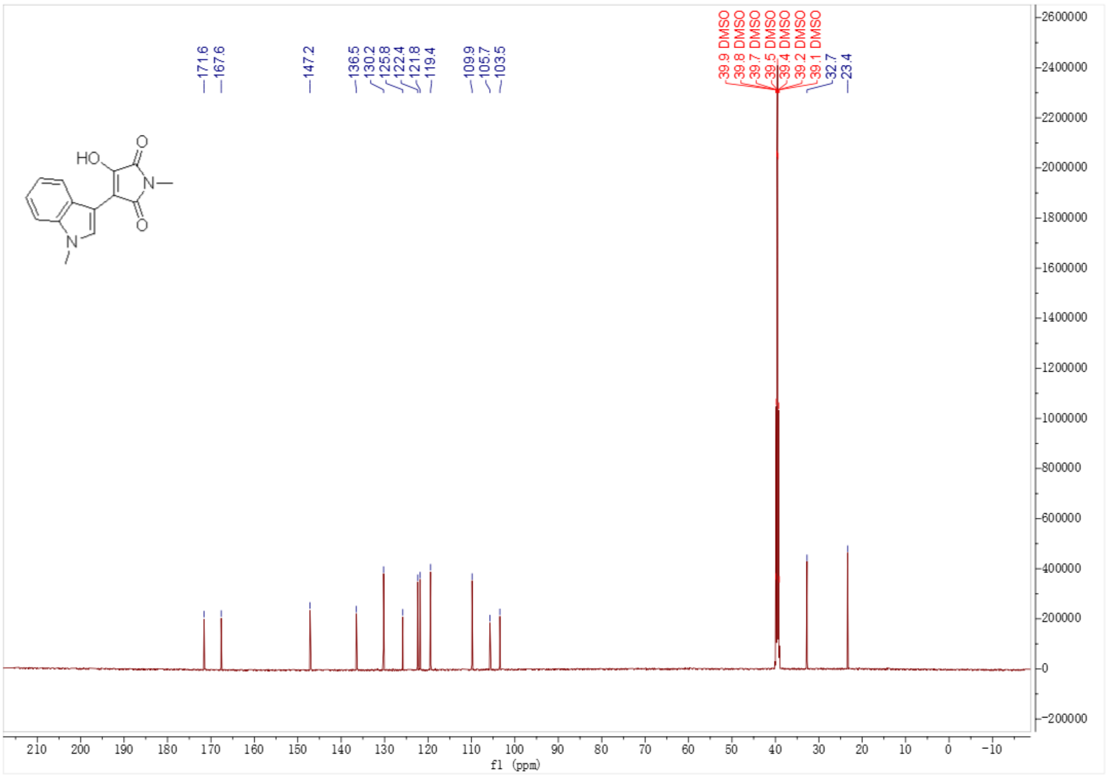

Supplementary Figure 52. ^{13}C NMR of 1s (151 MHz, DMSO-d_6).

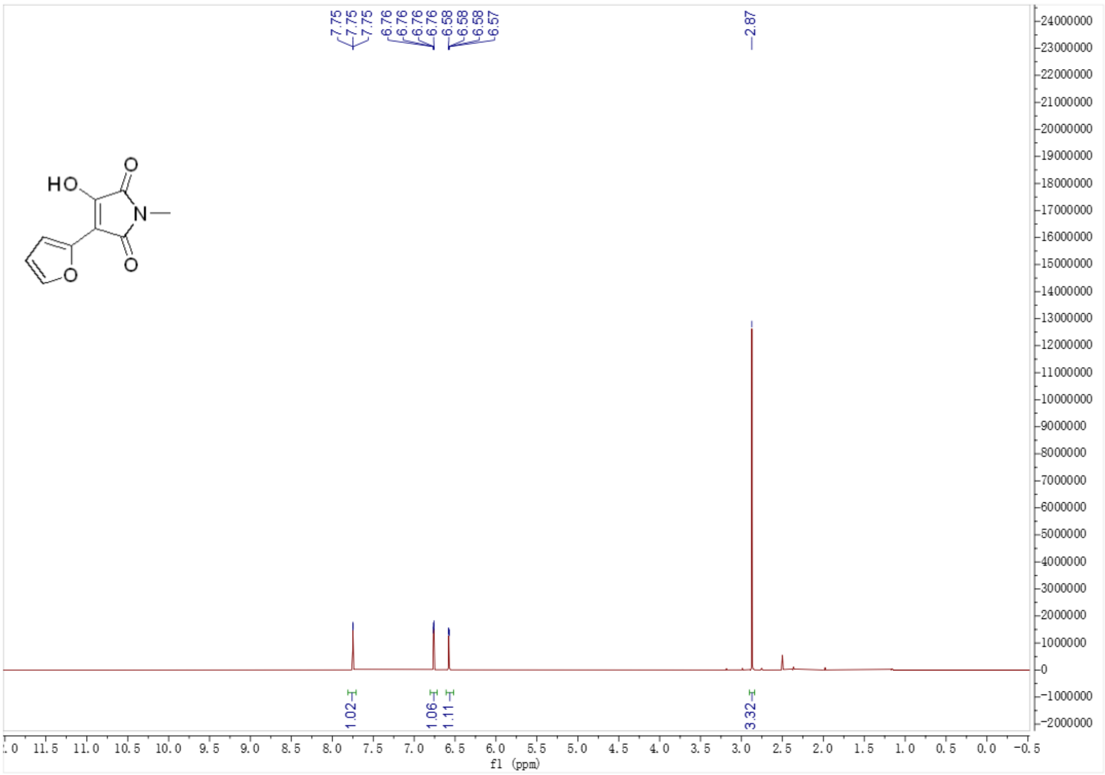

Supplementary Figure 53. ^{1}H NMR of 1t (600 MHz, DMSO-d_6).

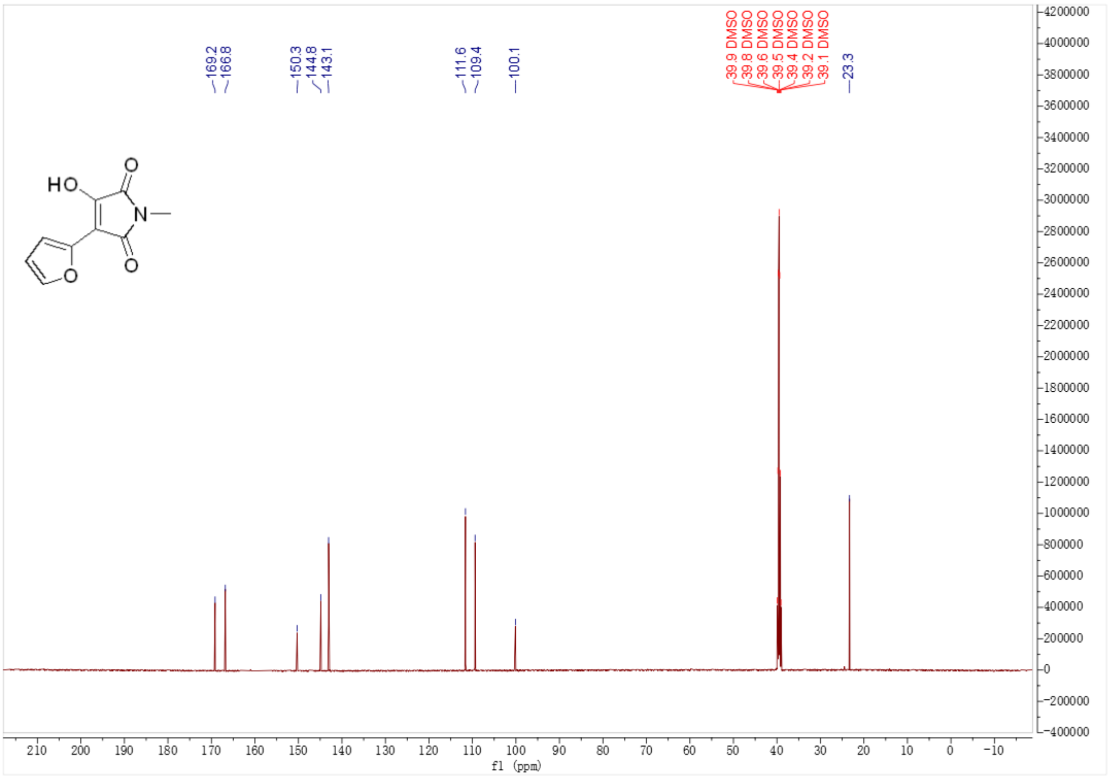

Supplementary Figure 54. ^{13}C **NMR of 1t (151 MHz, DMSO-d_6).**

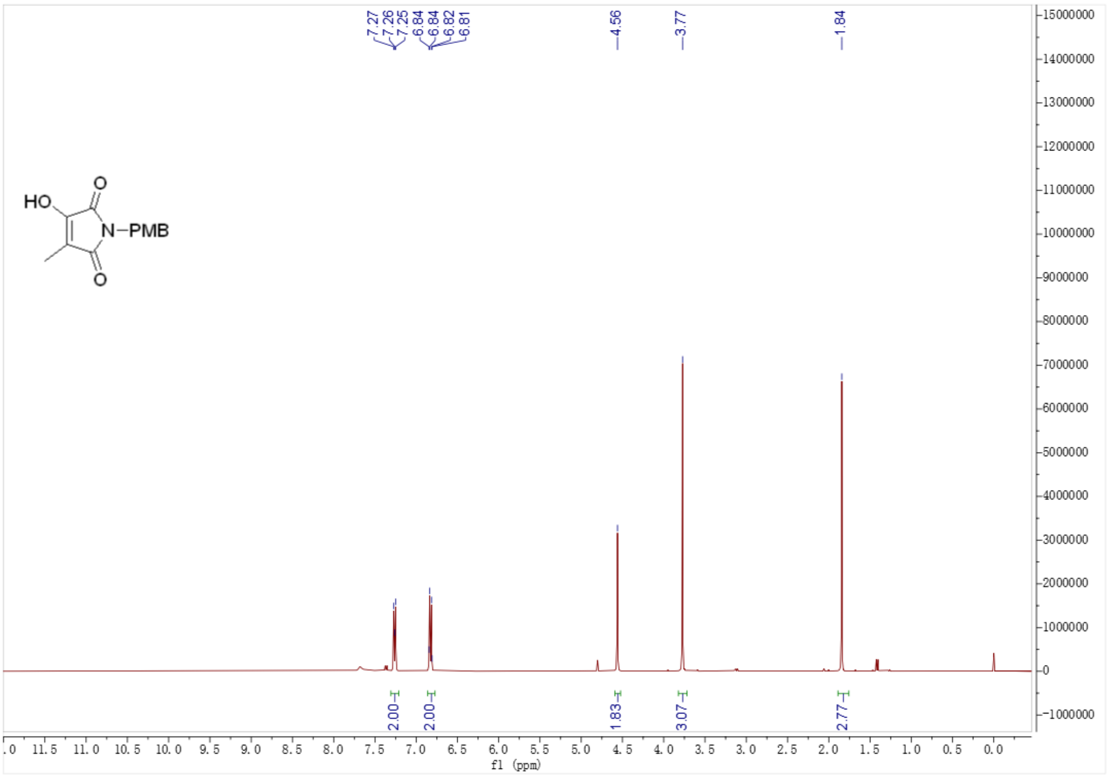

Supplementary Figure 55. ^{1}H **NMR of 1u (400 MHz, Chloroform-d).**

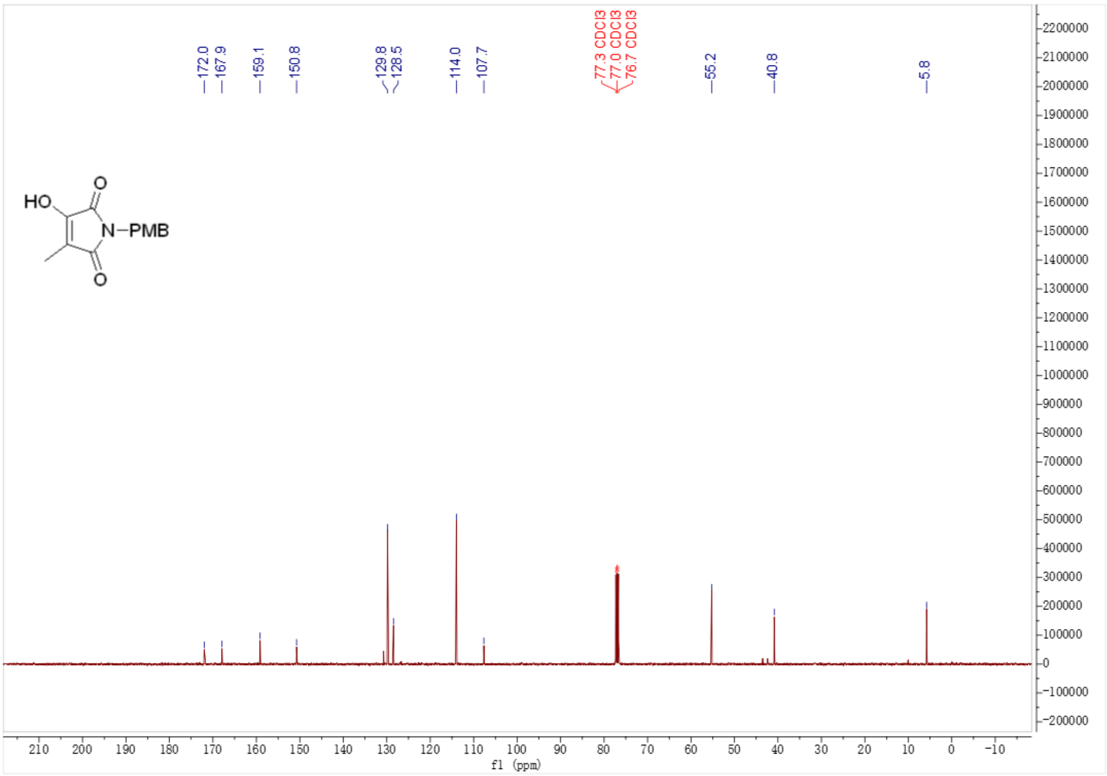

Supplementary Figure 56. ^{13}**C NMR of 1u (101 MHz, Chloroform-*d*).**

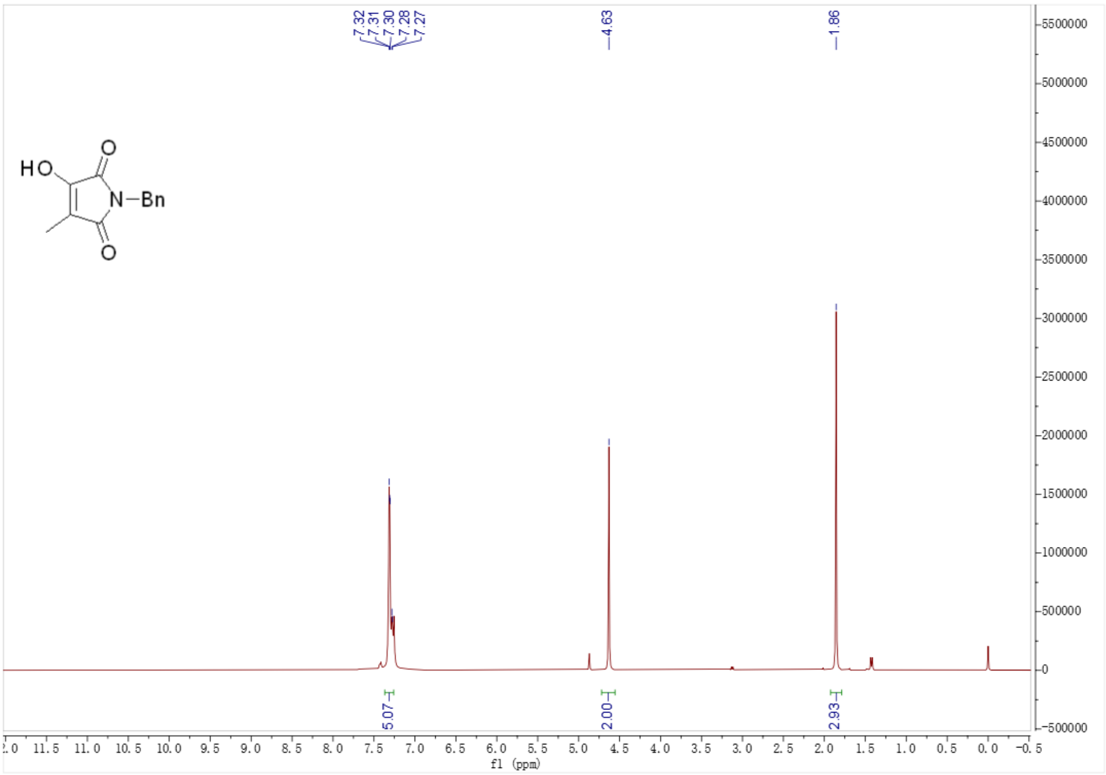

Supplementary Figure 57. ^{1}**H NMR of 1v (400 MHz, Chloroform-*d*).**

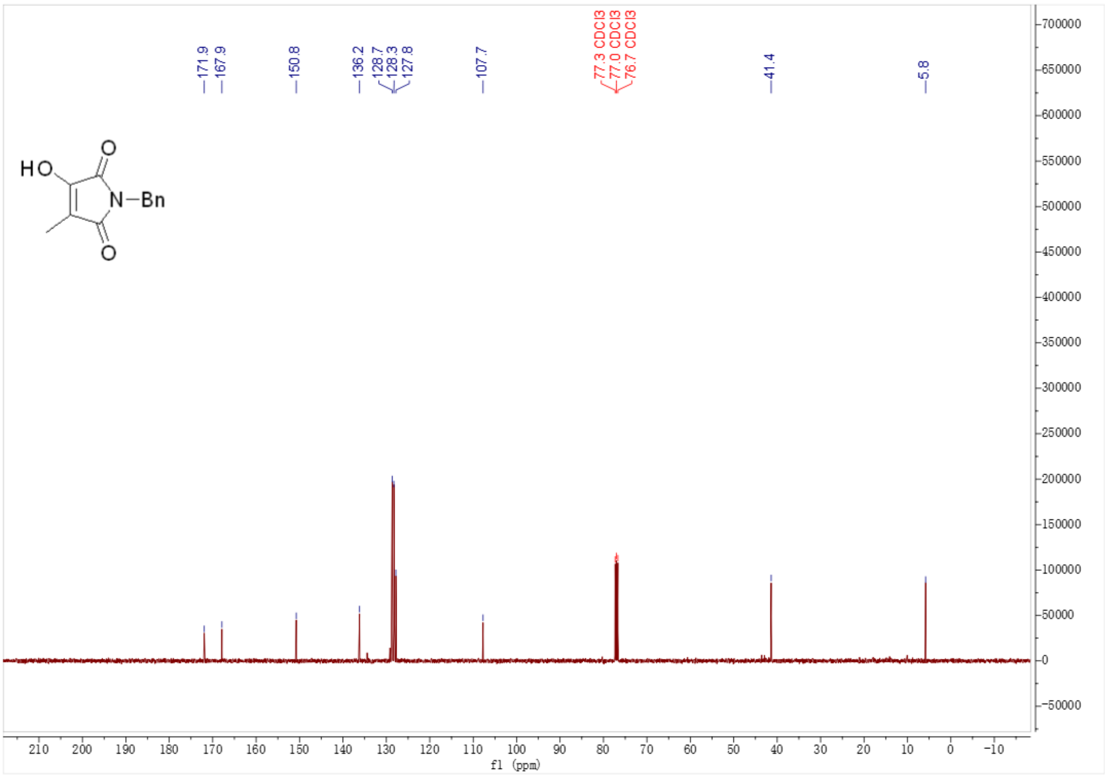

Supplementary Figure 58. ^{13}**C NMR of 1v (101 MHz, Chloroform-*d*).**

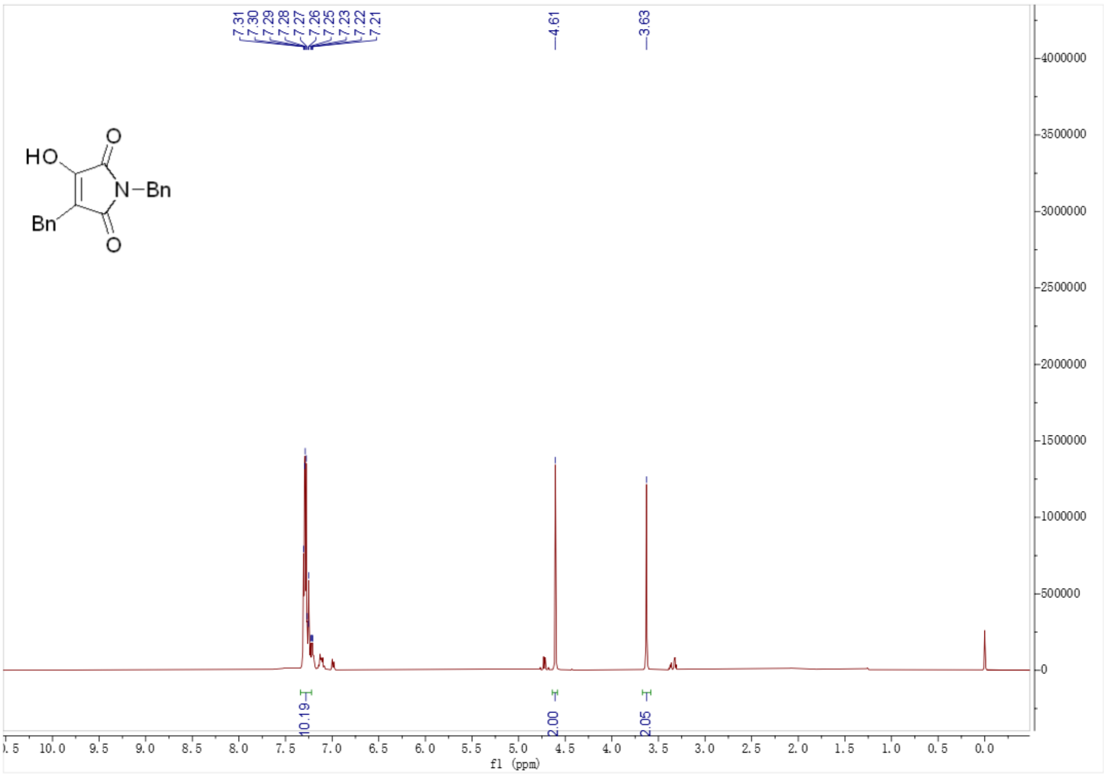

Supplementary Figure 59. ^{1}**H NMR of 1w (400 MHz, Chloroform-*d*).**

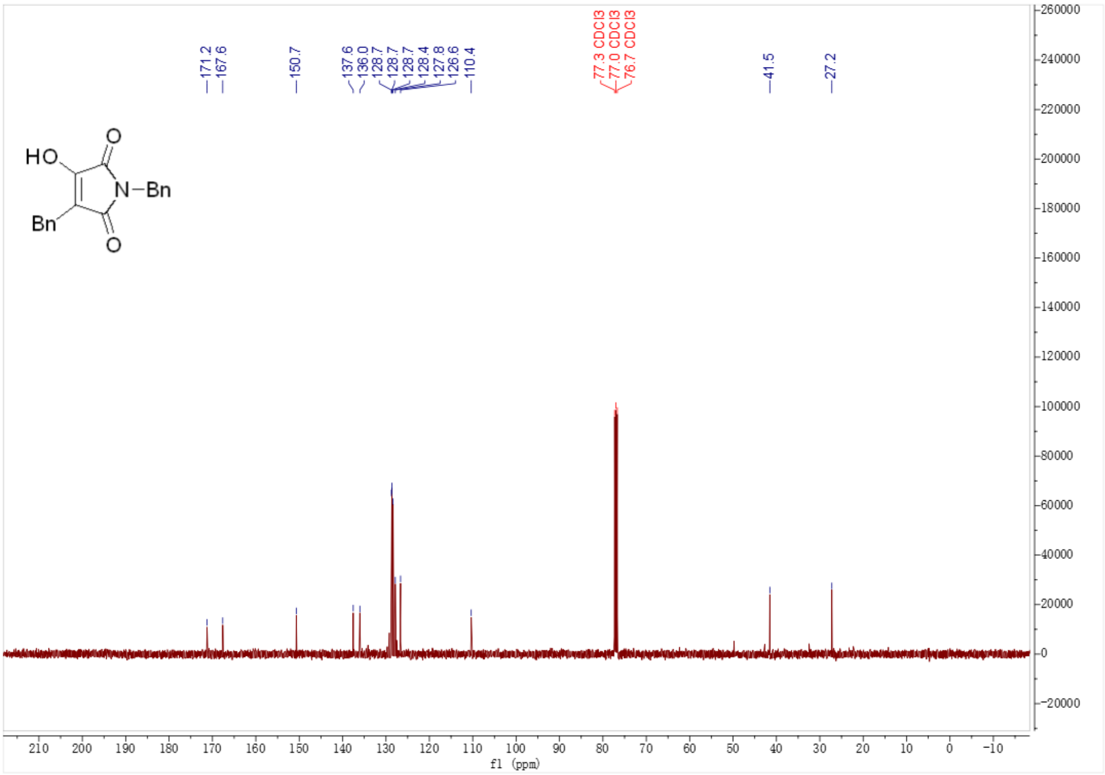

Supplementary Figure 60. ^{13}**C NMR of 1w (101 MHz, Chloroform-*d*).**

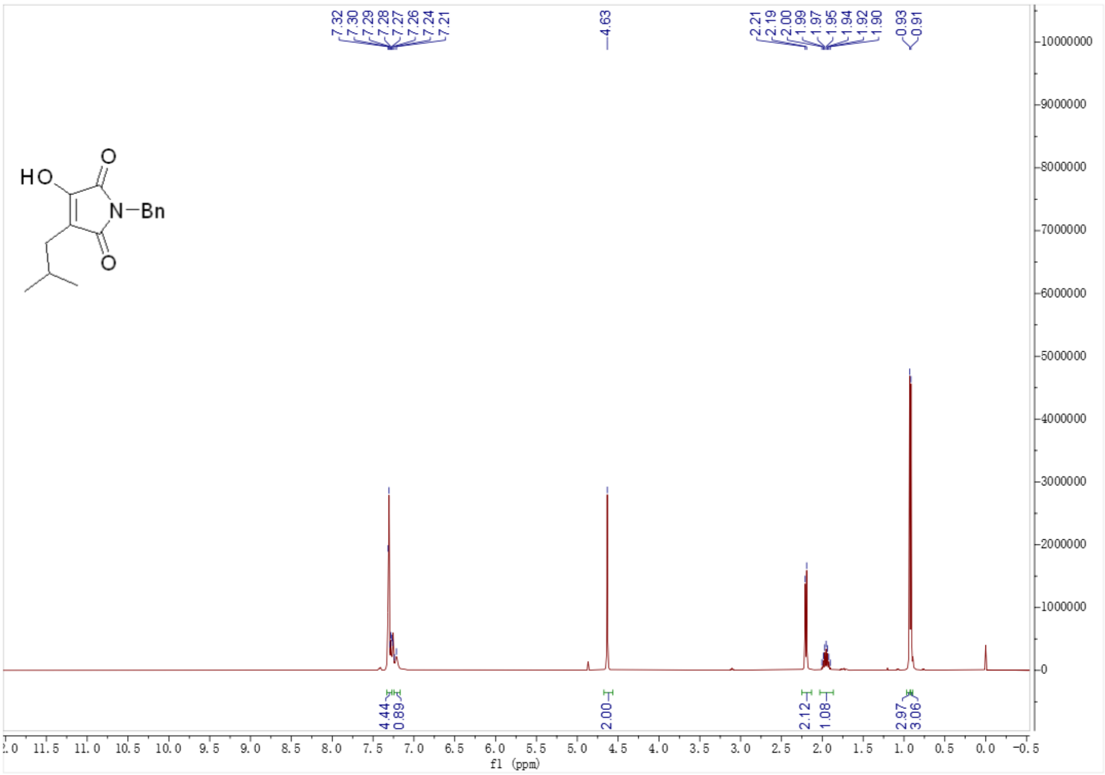

Supplementary Figure 61. ^{1}**H NMR of 1x (400 MHz, Chloroform-*d*).**

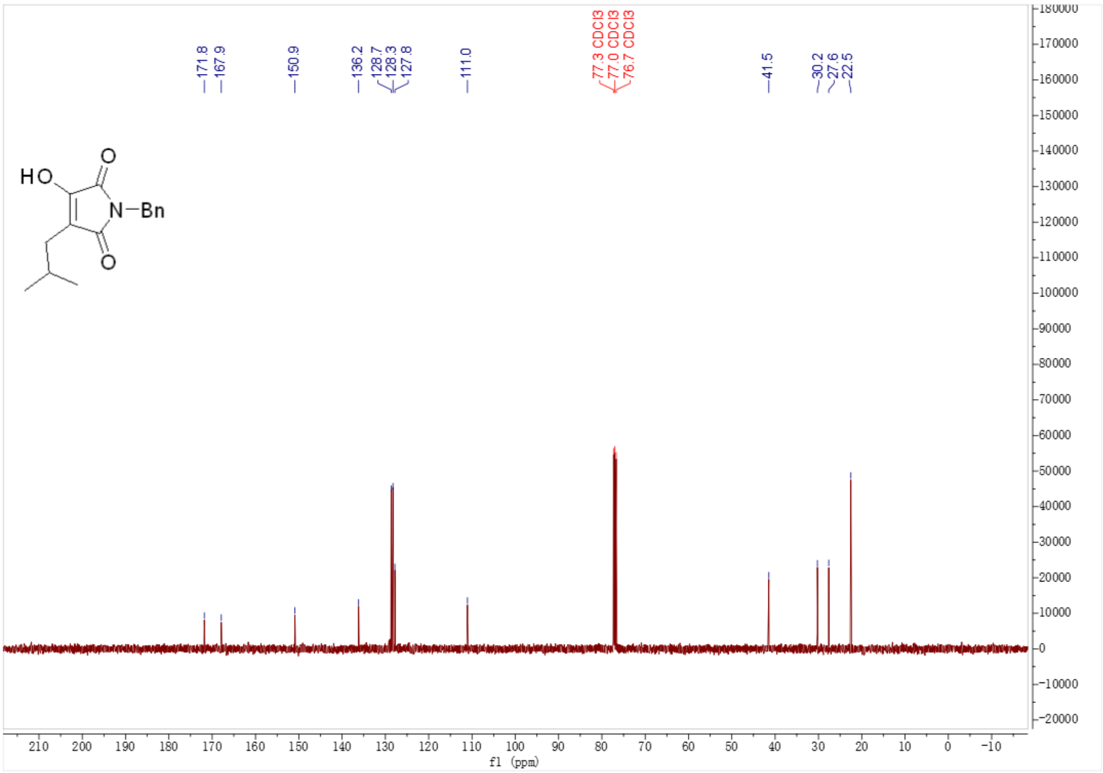

Supplementary Figure 62. ^{13}C NMR of 1x (101 MHz, Chloroform-*d*).

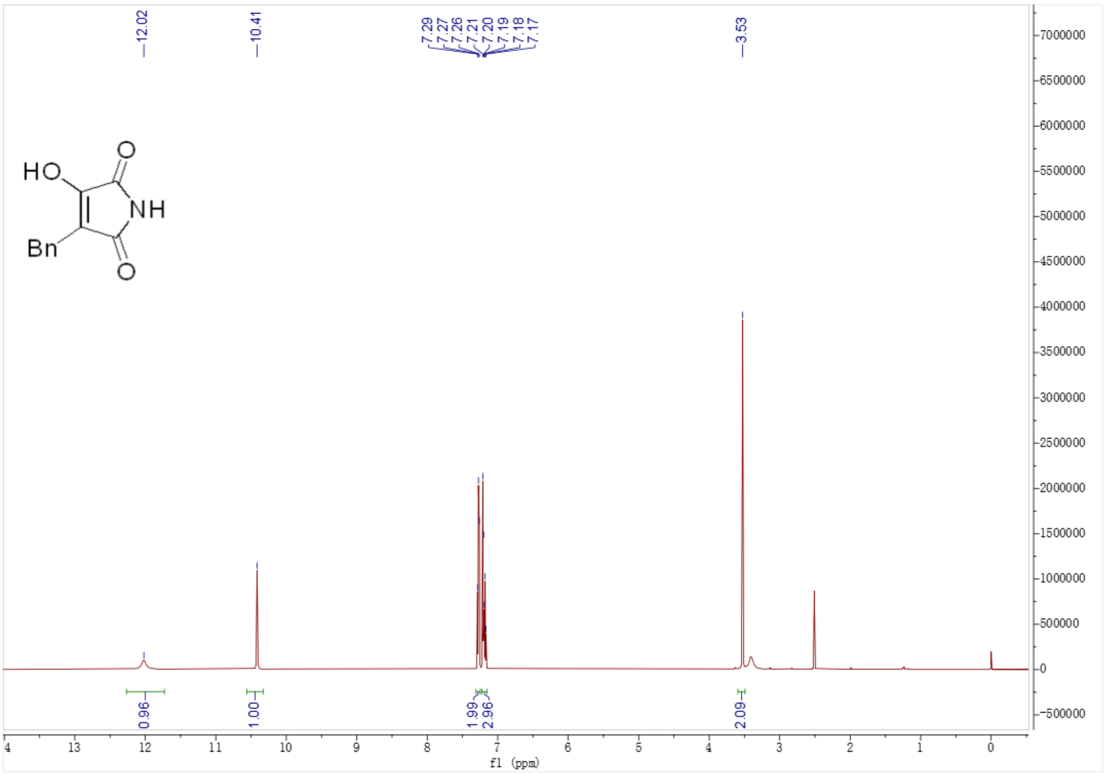

Supplementary Figure 63. ^{1}H NMR of 1y (600 MHz, DMSO-d_6).

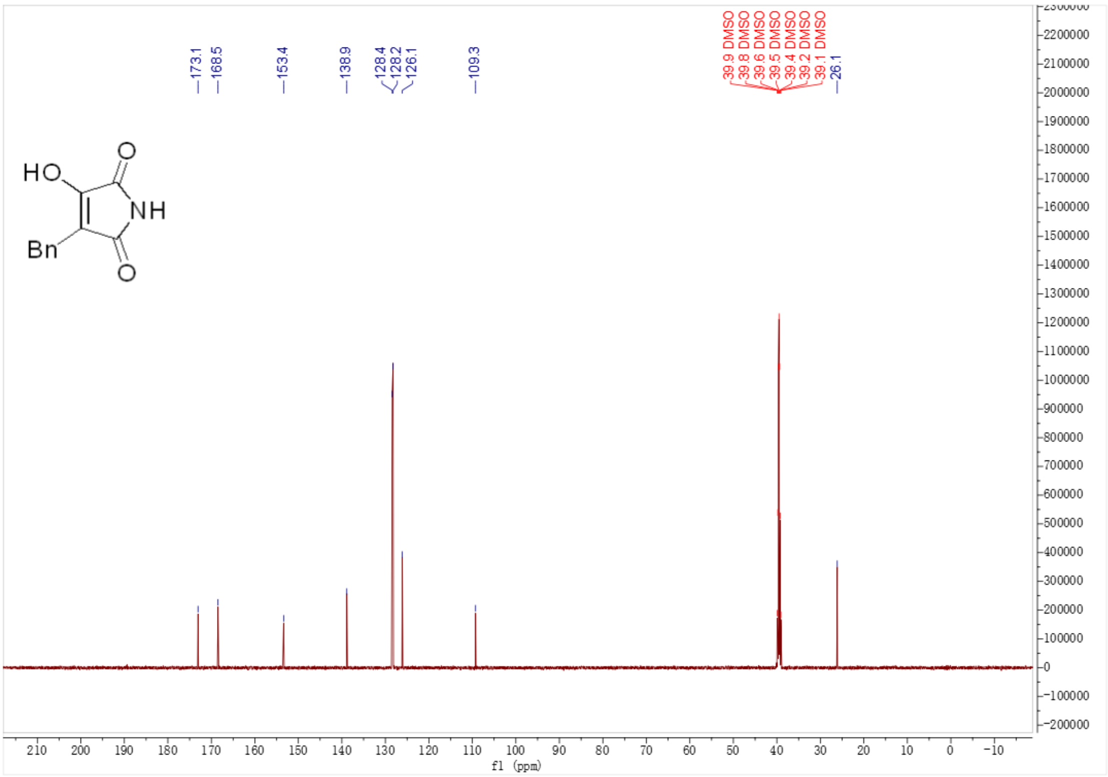

Supplementary Figure 64. ^{13}**C NMR of 1y (151 MHz, DMSO-d_6).**

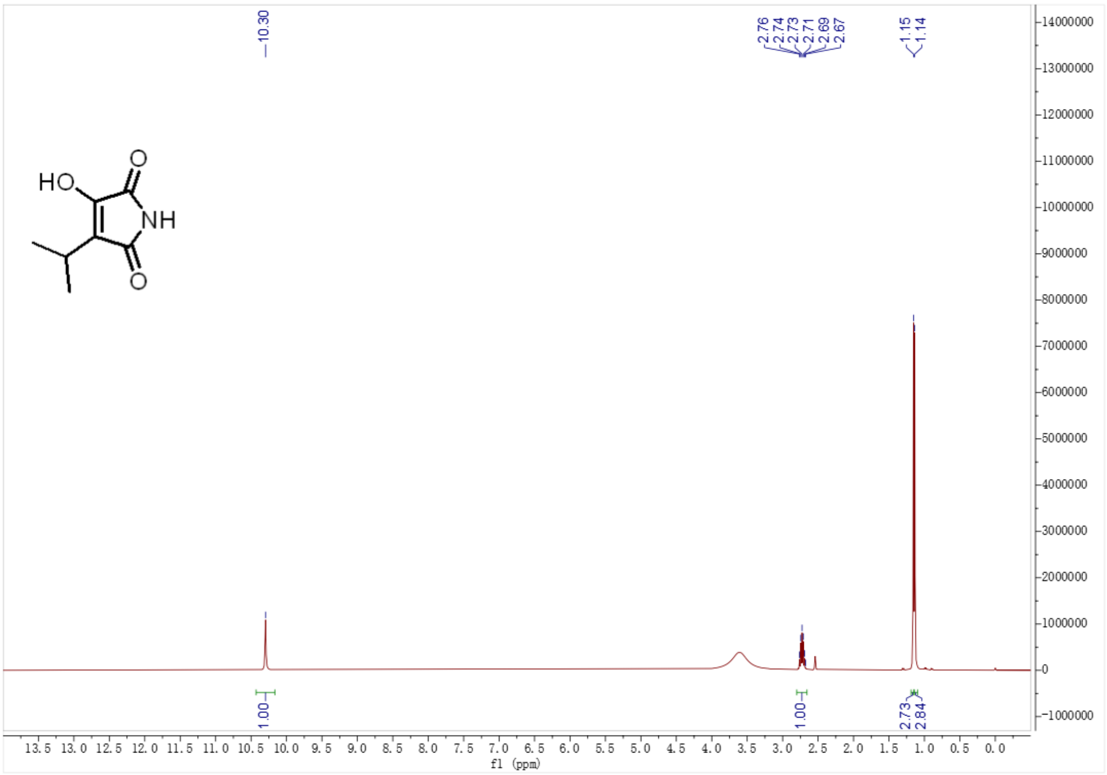

Supplementary Figure 65. ^{1}**H NMR of 1z (400 MHz, DMSO-d_6).**

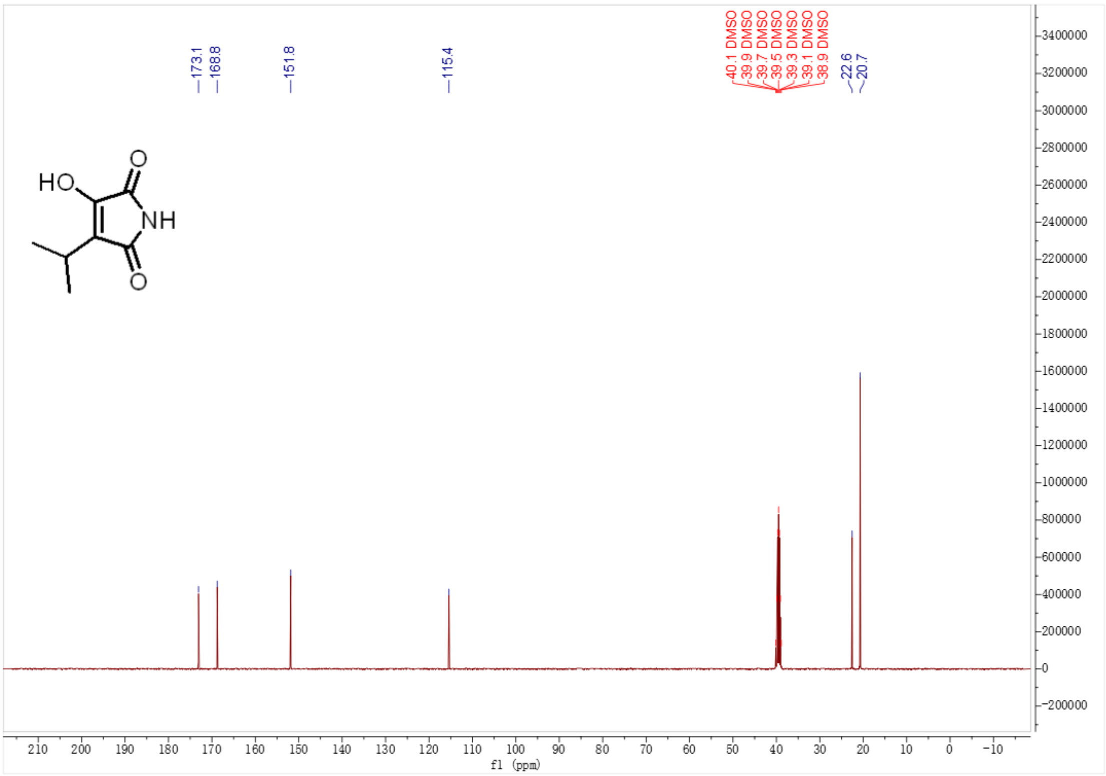

Supplementary Figure 66. ^{13}C NMR of 1z (101 MHz, DMSO-d_6).

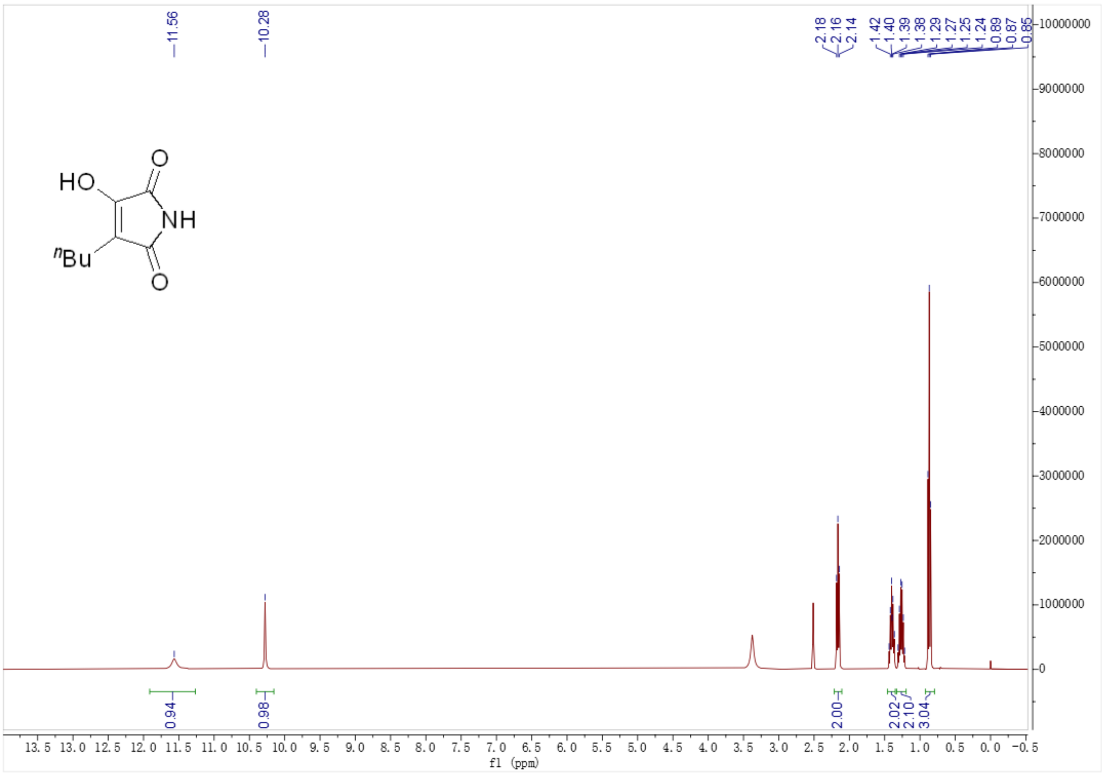

Supplementary Figure 67. ^{1}H NMR of 1aa (400 MHz, DMSO-d_6).

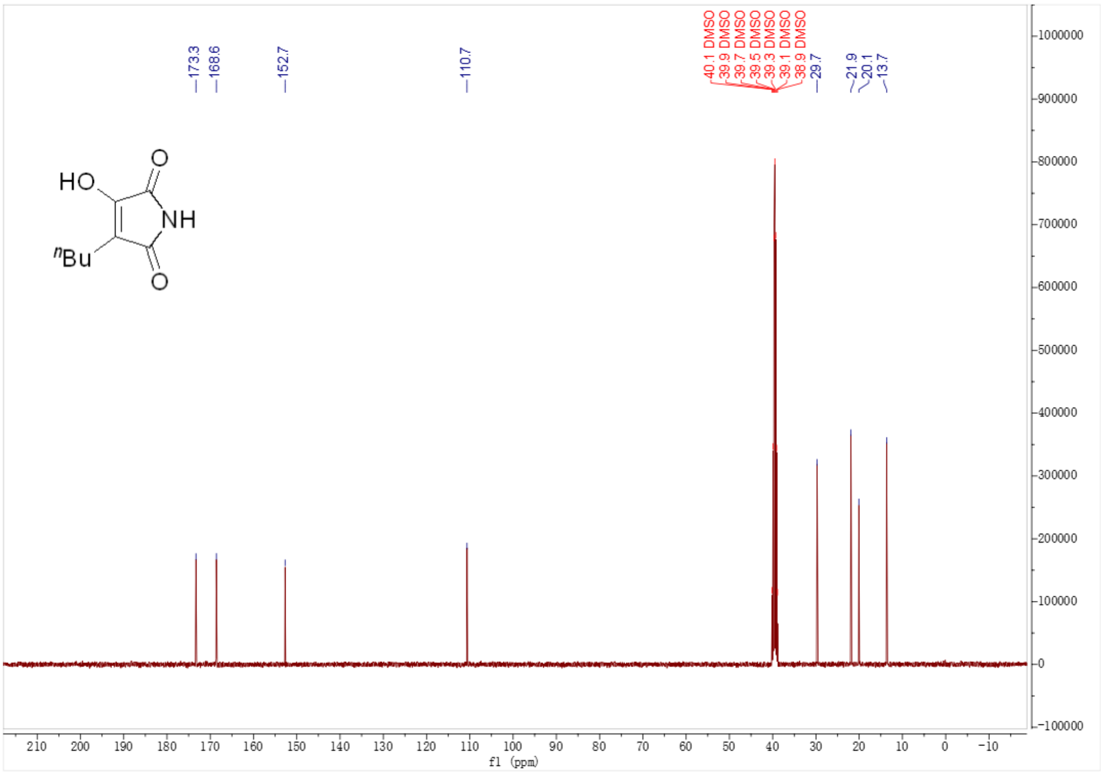

Supplementary Figure 68. ^{13}C NMR of 1aa (101 MHz, DMSO-d_6).

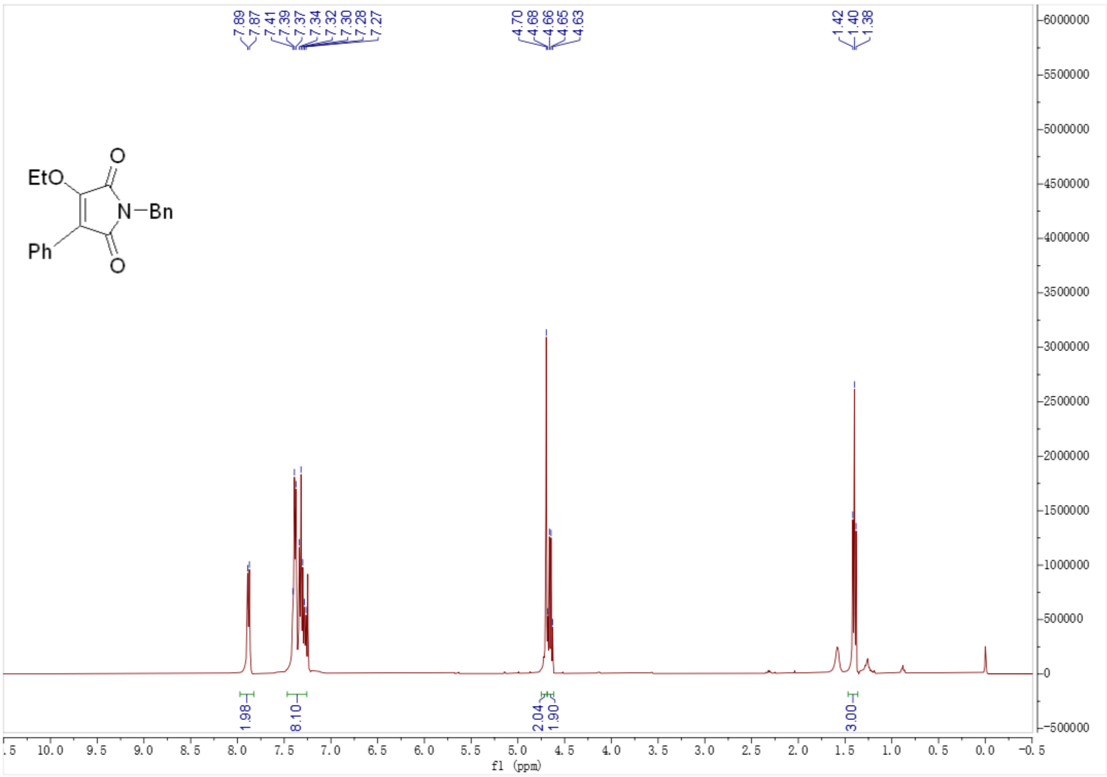

Supplementary Figure 69. ^{1}H NMR of 1a' (400 MHz, Chloroform-d).

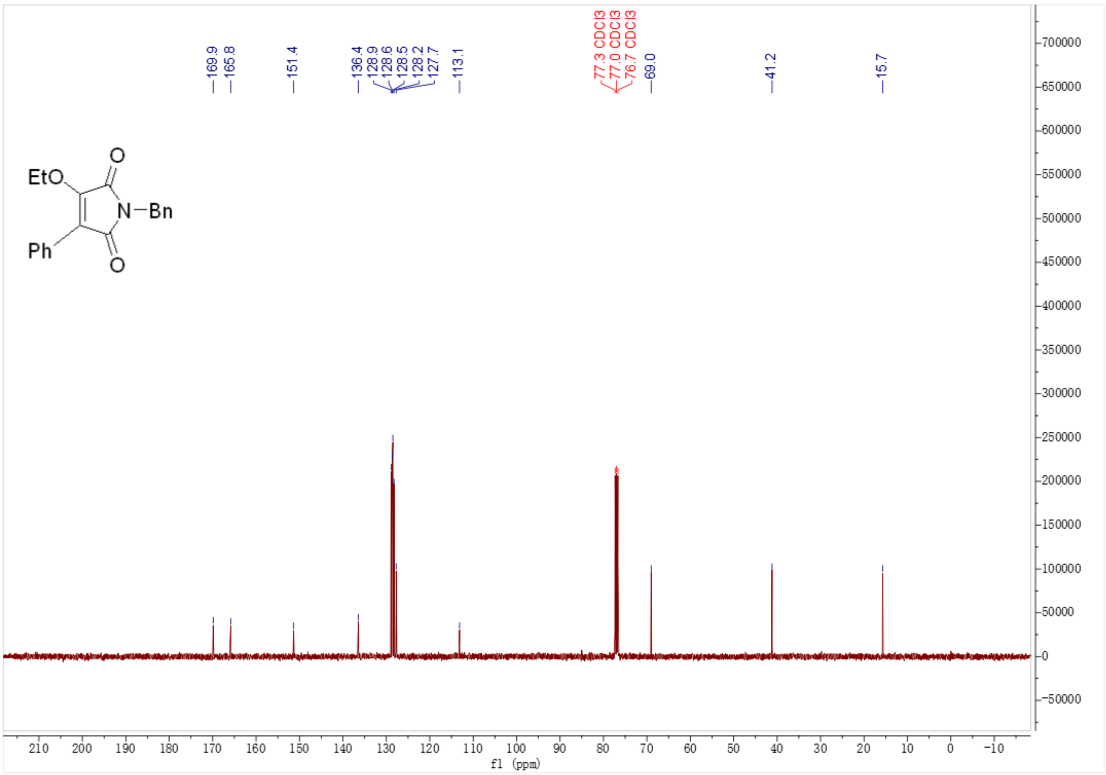

Supplementary Figure 70. ^{13}C NMR of 1a’ (101 MHz, Chloroform-*d*)

NMR Spectra of the 2, 3, 4

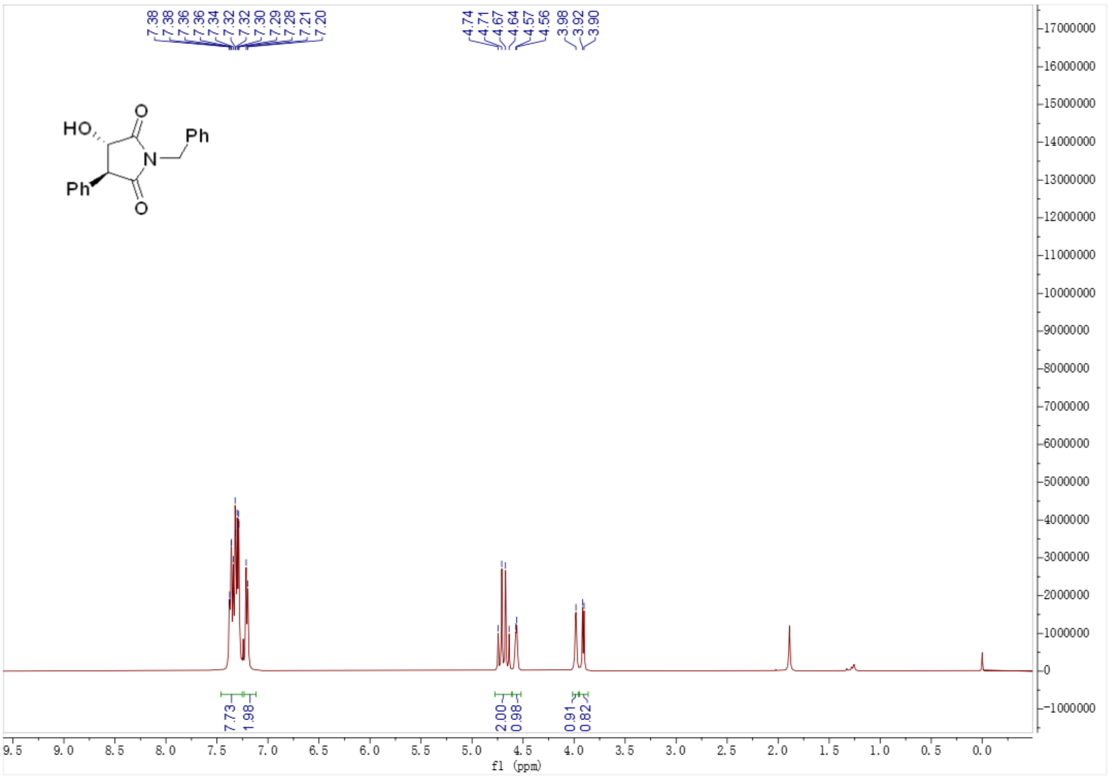

Supplementary Figure 71. ^{1}H NMR of 2a (400 MHz, Chloroform-*d*).

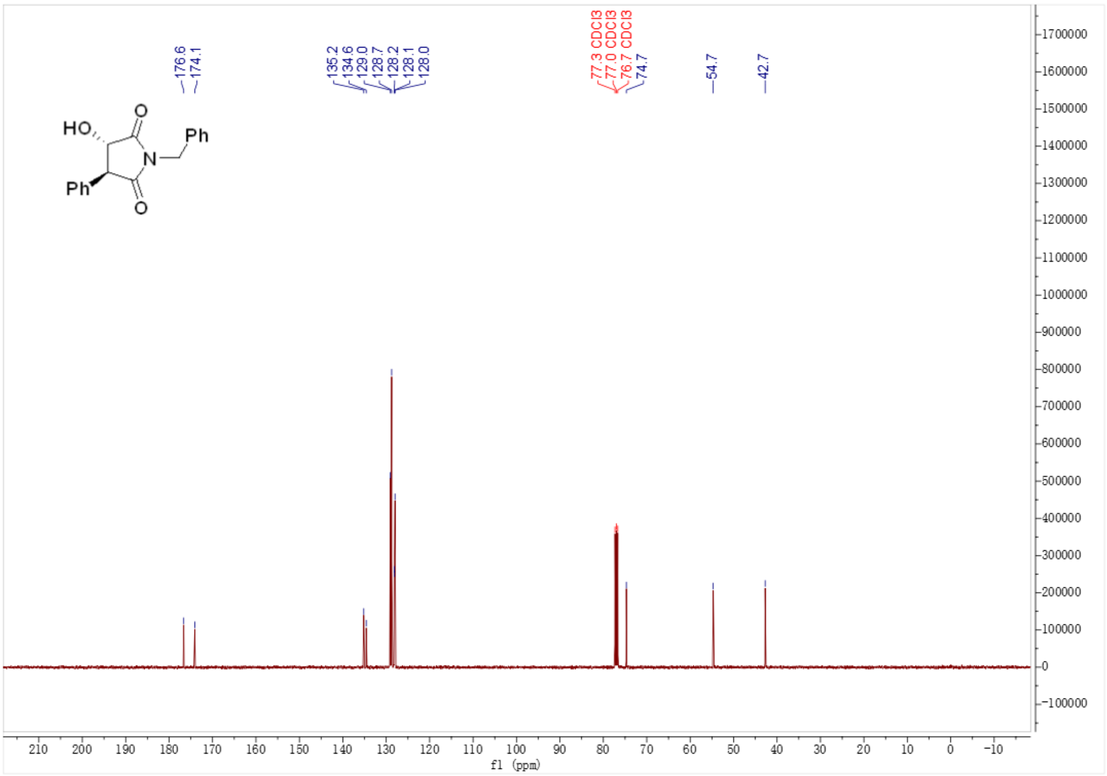

Supplementary Figure 72. ^{13}C NMR of 2a (101 MHz, Chloroform-*d*)

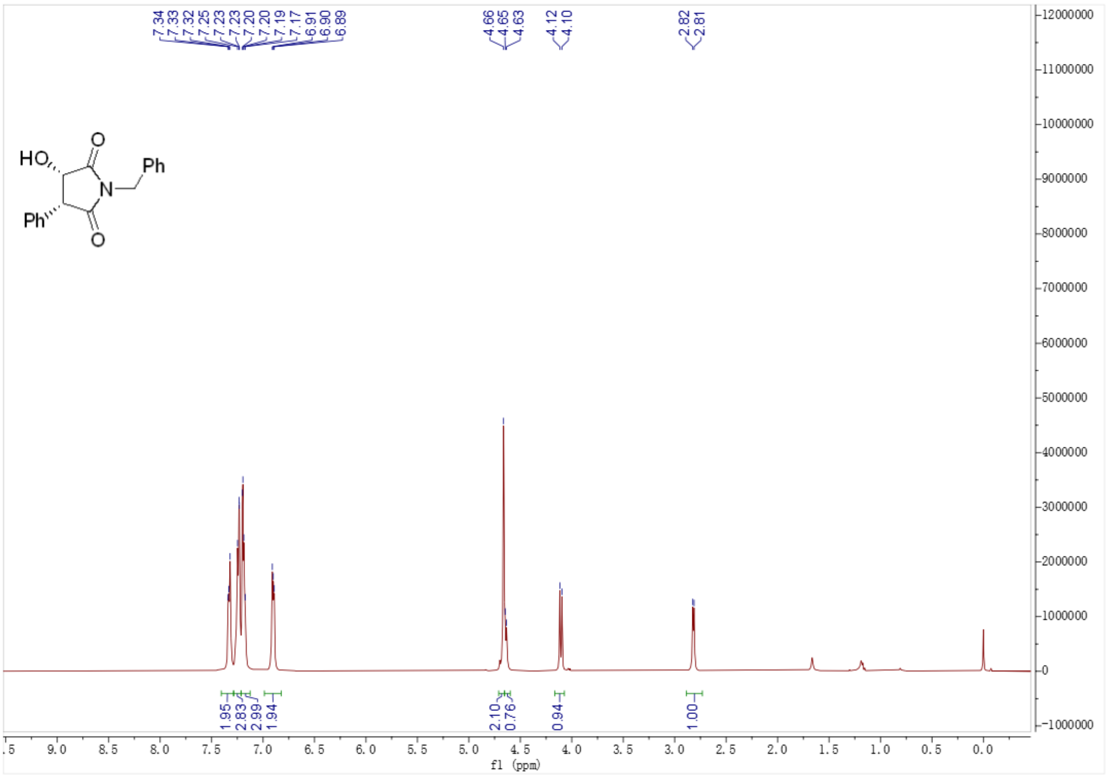

Supplementary Figure 73. ^{1}H NMR of 3a (400 MHz, Chloroform-*d*).

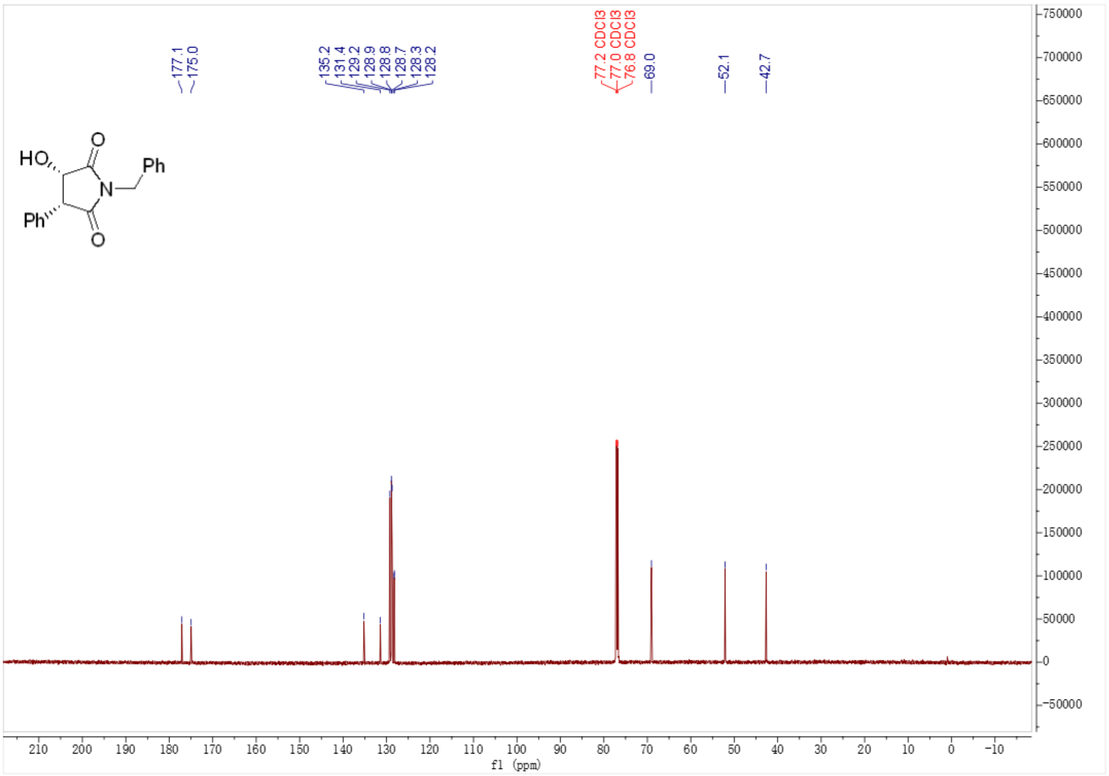

Supplementary Figure 74. ^{13}C NMR of 3a (151 MHz, Chloroform-*d*).

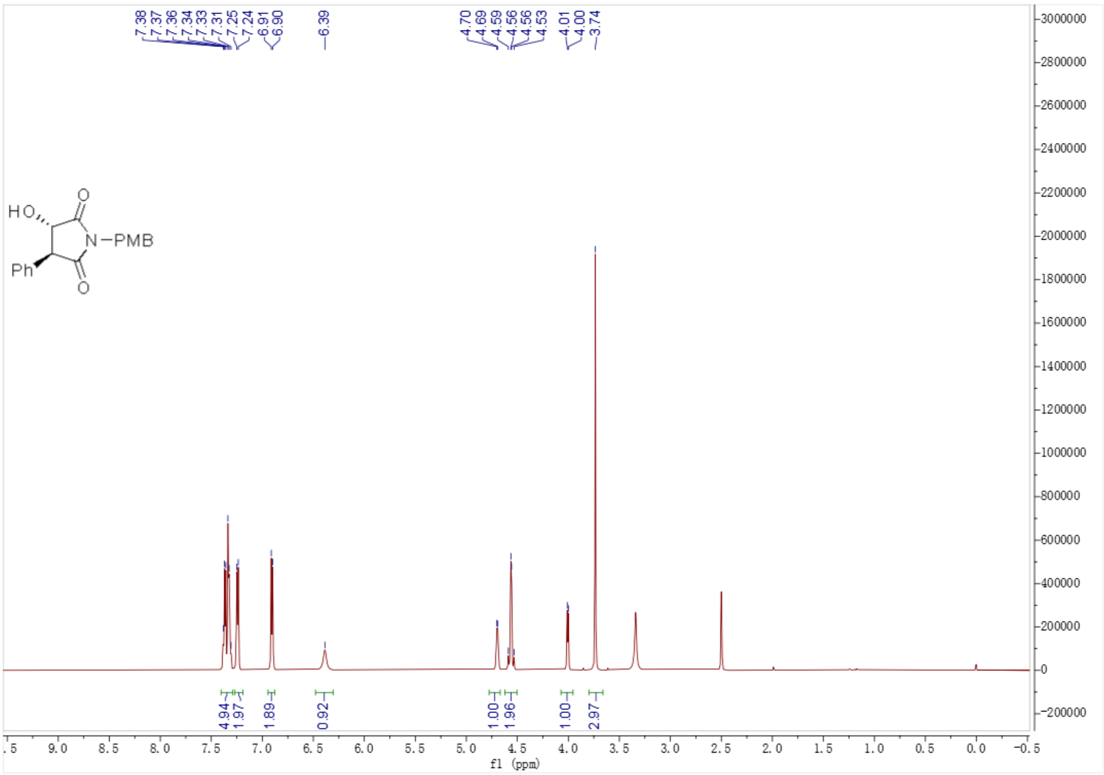

Supplementary Figure 75. ^{1}H NMR of 2b (600 MHz, DMSO-d_6).

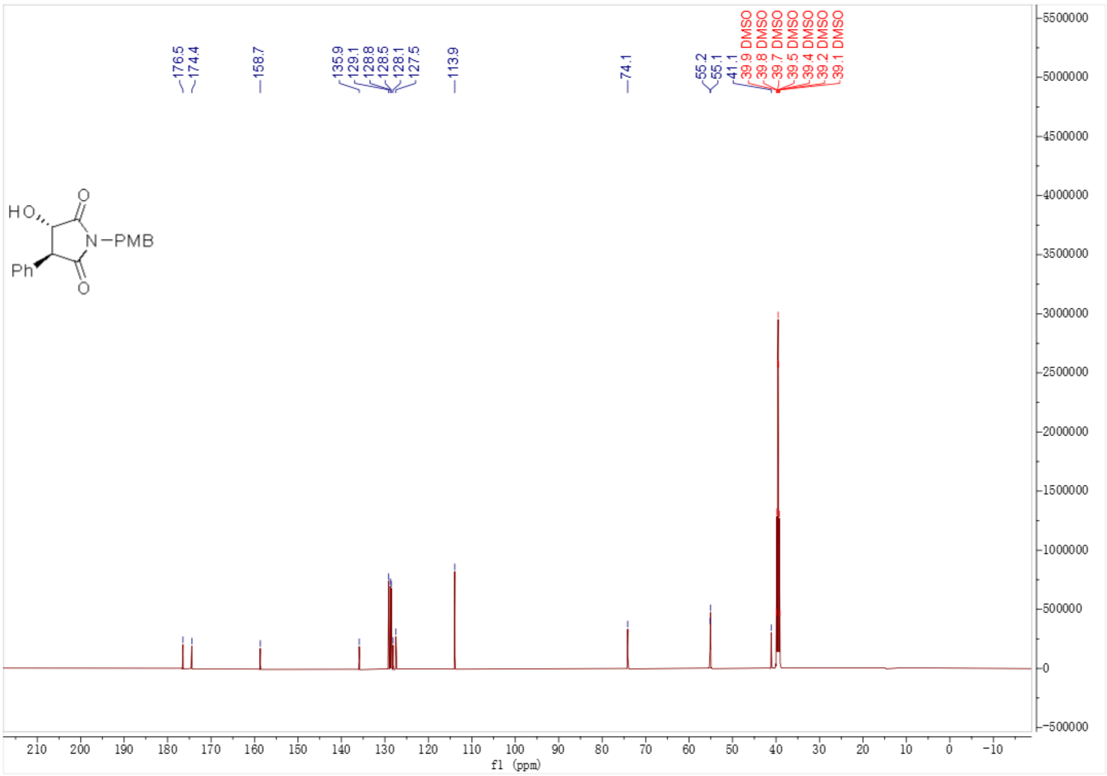

Supplementary Figure 76. ^{13}C NMR of 2b (151 MHz, DMSO-d_6).

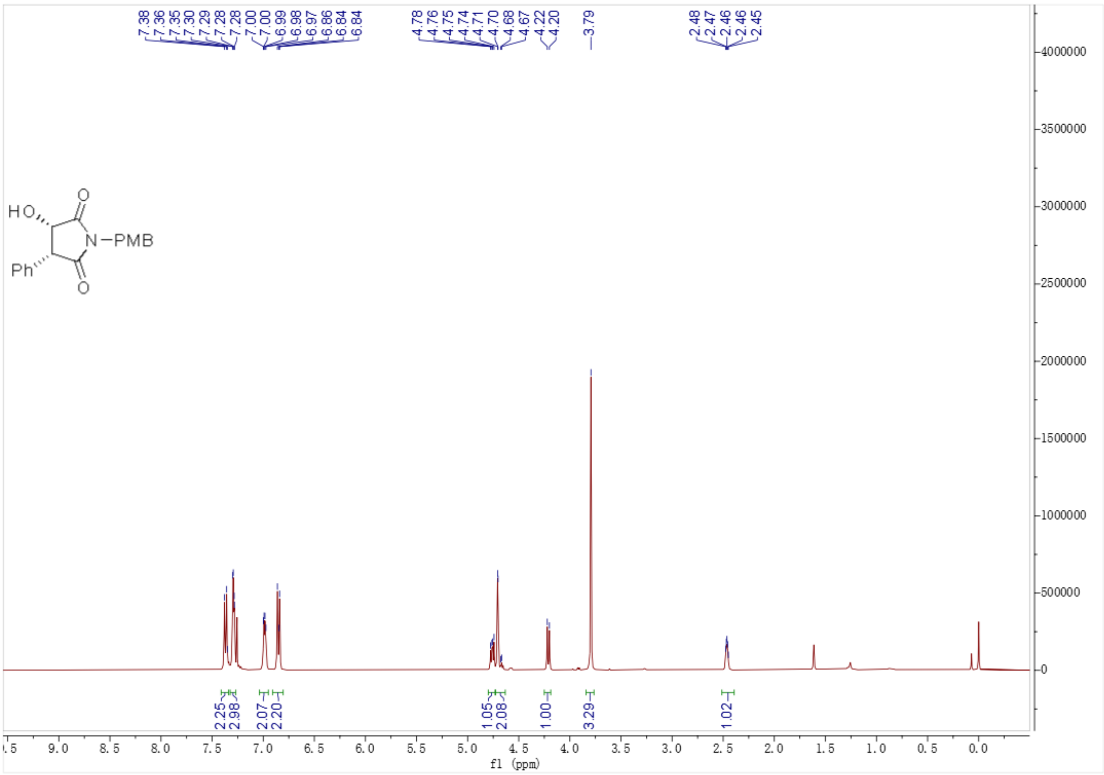

Supplementary Figure 77. ^{1}H NMR of 3b (400 MHz, Chloroform-*d*).

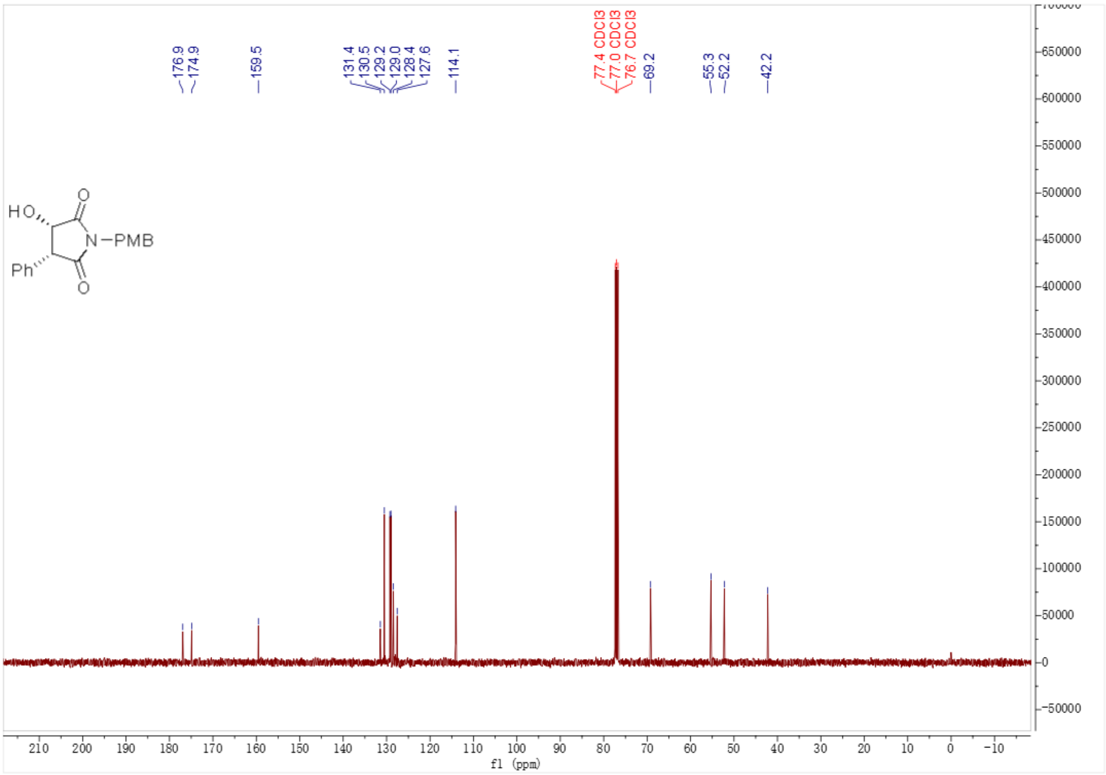

Supplementary Figure 78. ^{13}C NMR of 3b (101 MHz, Chloroform-*d*).

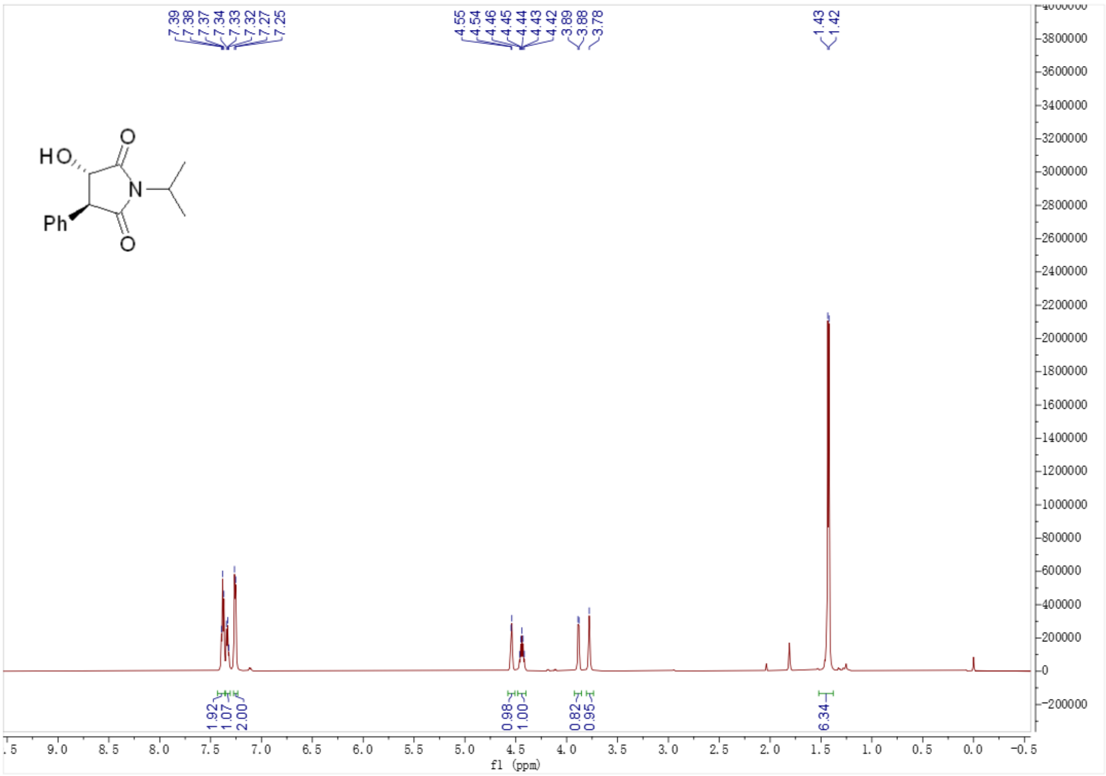

Supplementary Figure 79. ^{1}**H NMR of 2c (600 MHz, Chloroform-*d*).**

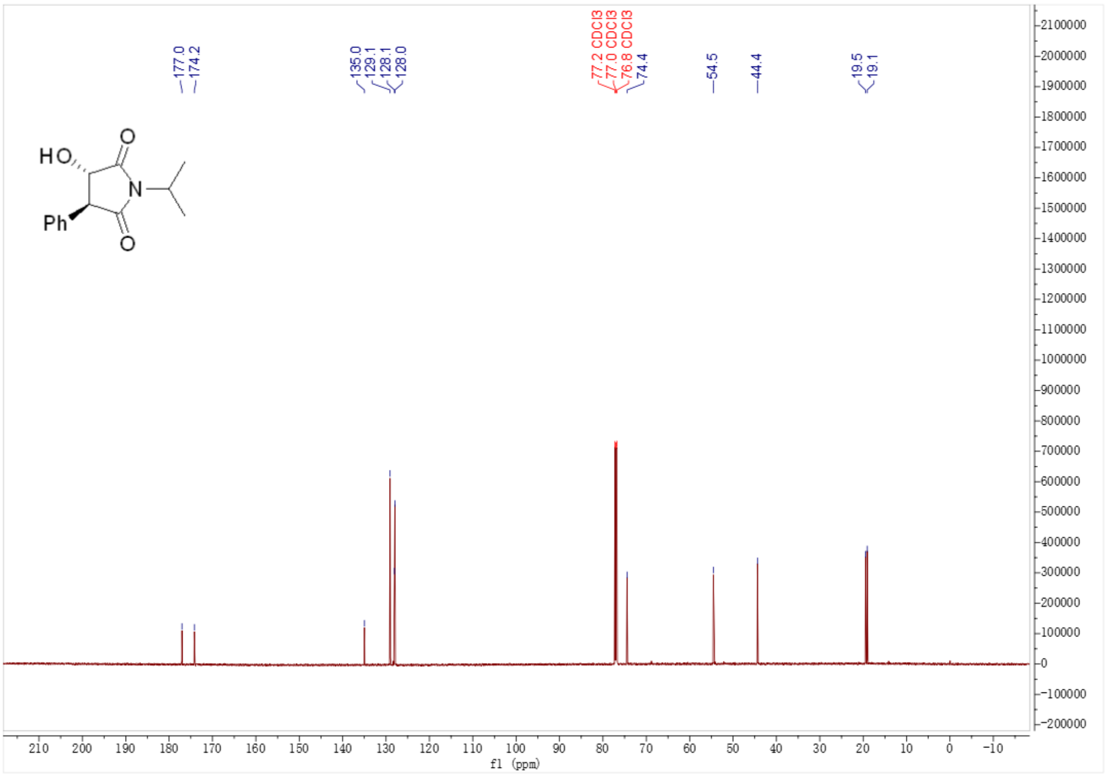

Supplementary Figure 80. ^{13}**C NMR of 2c (151 MHz, Chloroform-*d*).**

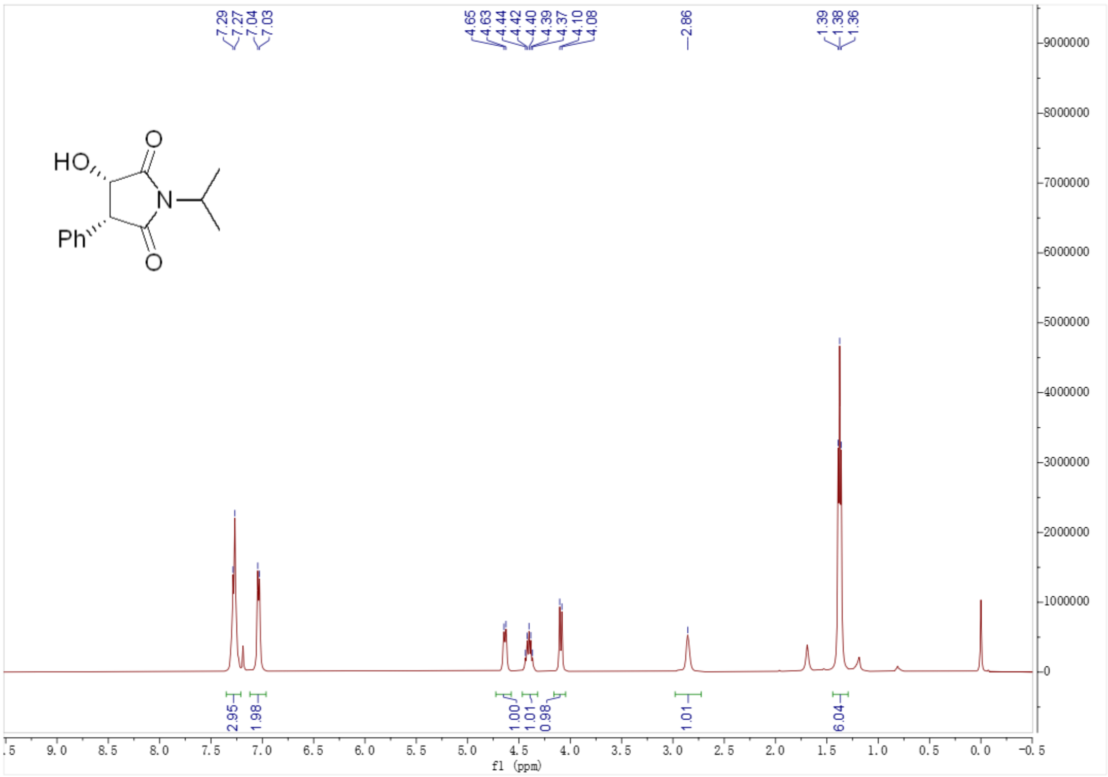

Supplementary Figure 81. ^{1}**H NMR of 3c (400 MHz, Chloroform-*d*).**

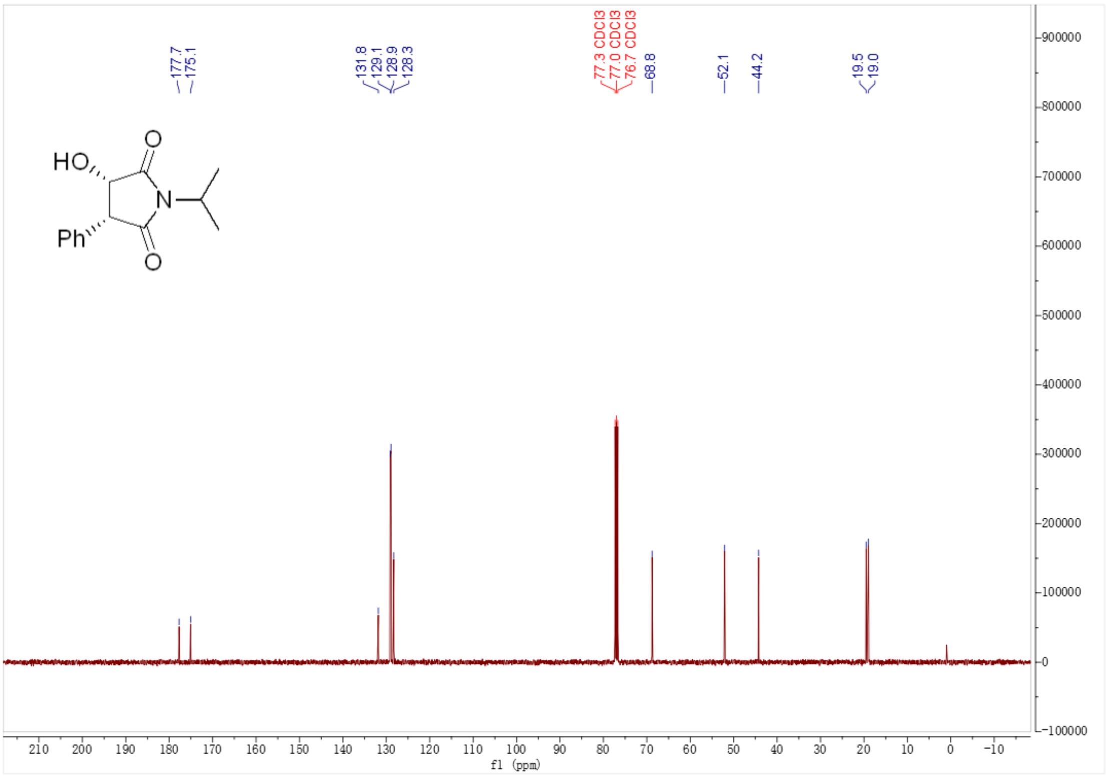

Supplementary Figure 82. ^{13}**C NMR of 3c (101 MHz, Chloroform-*d*).**

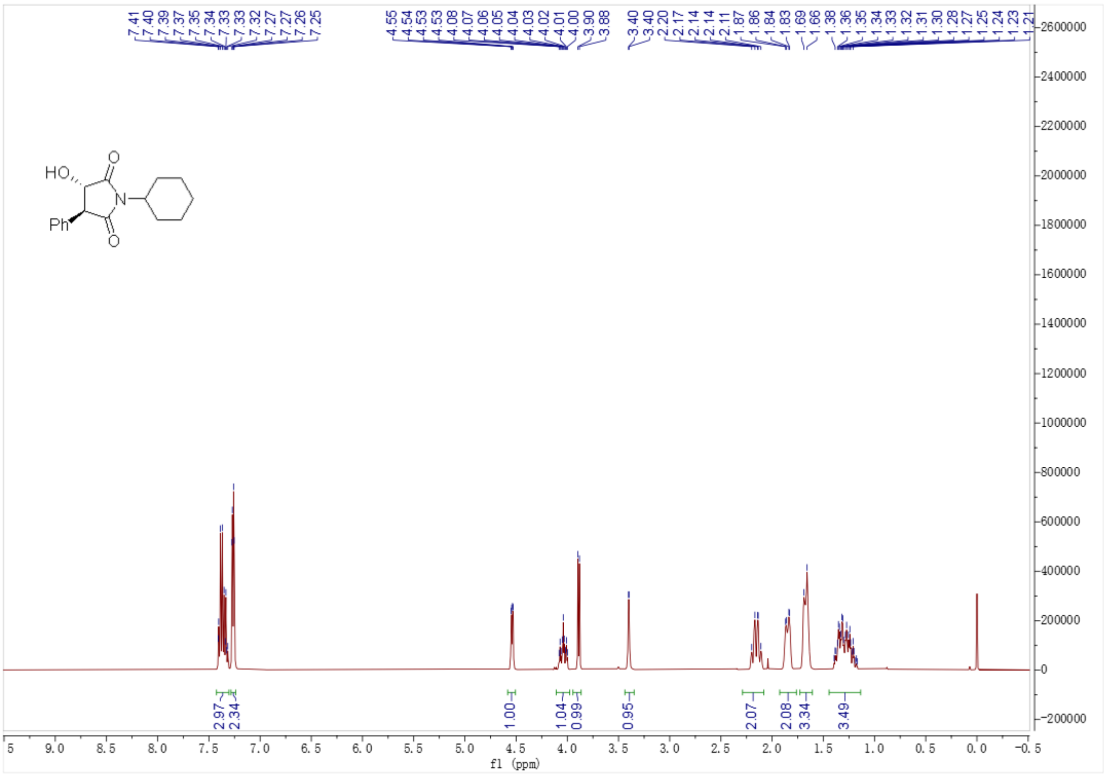

Supplementary Figure 83. **^{1}H NMR of 2d (400 MHz, Chloroform-*d*).**

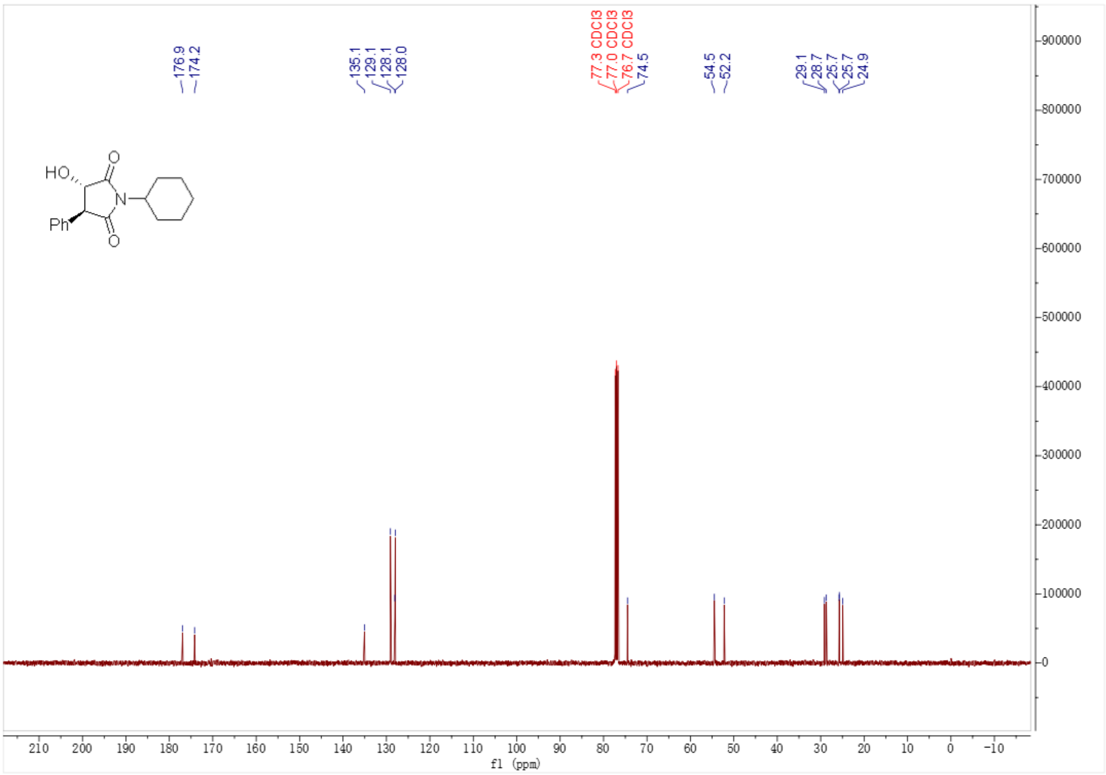

Supplementary Figure 84. **^{13}C NMR of 2d (101 MHz, Chloroform-*d*).**

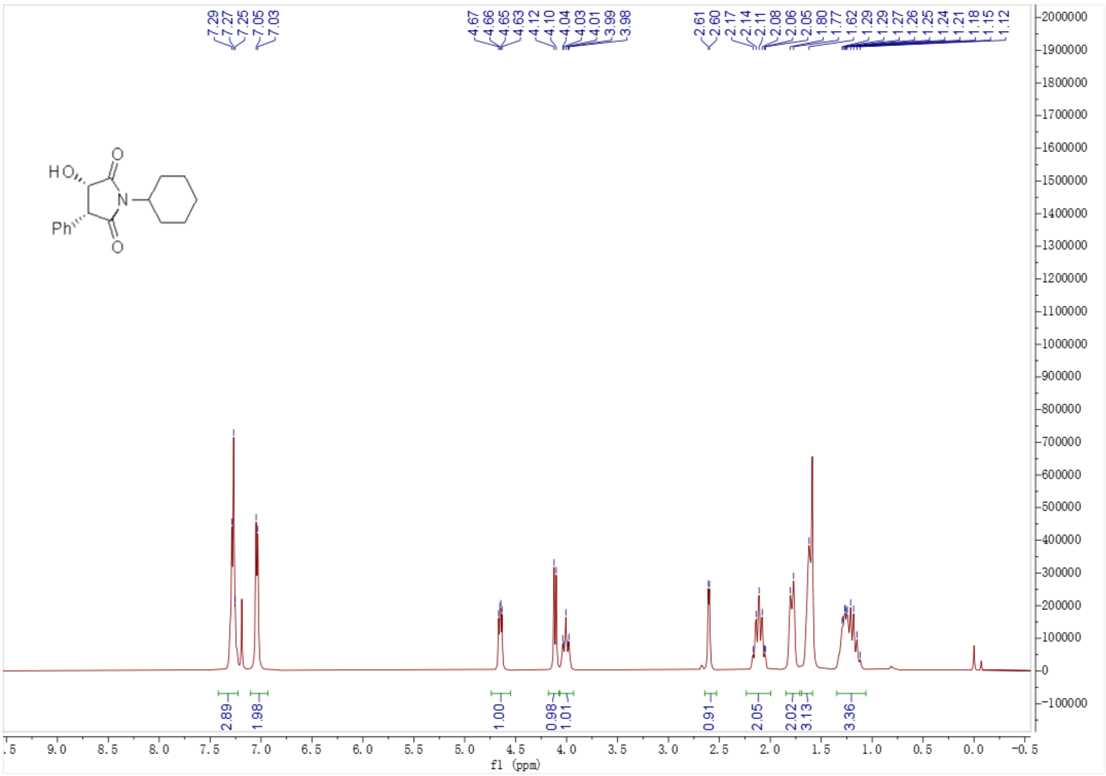

Supplementary Figure 85. ^{1}H NMR of 3d (400 MHz, Chloroform-*d*).

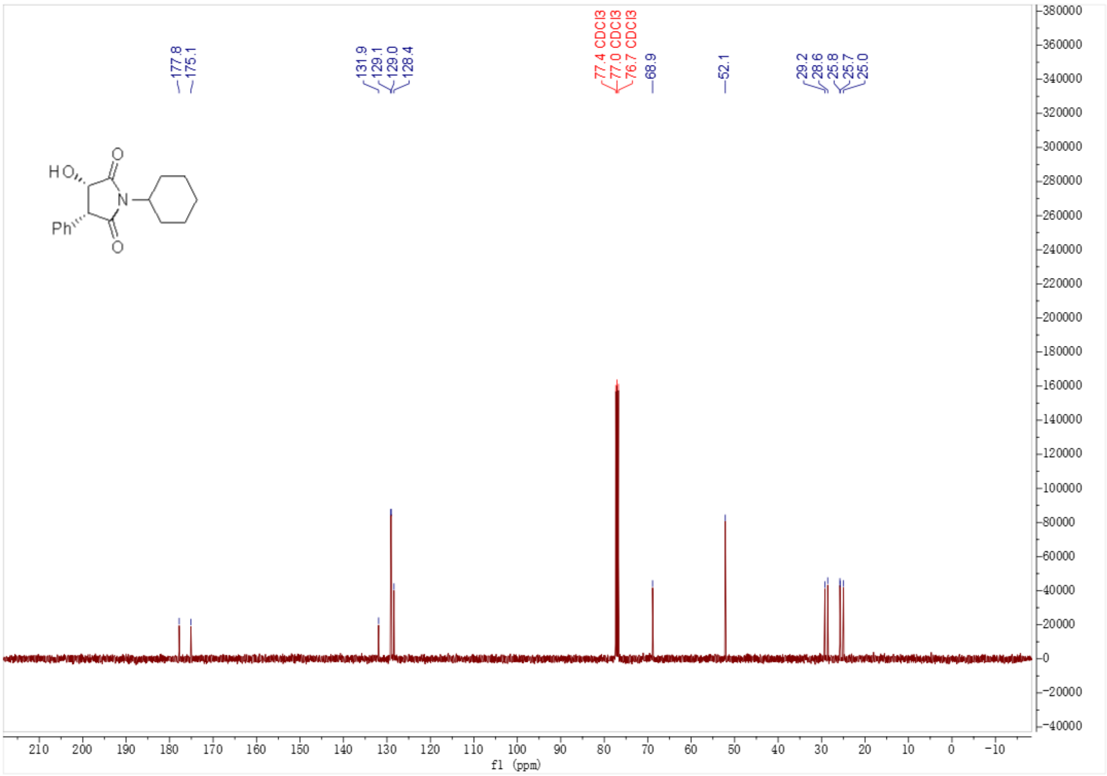

Supplementary Figure 86. ^{13}C NMR of 3d (101 MHz, Chloroform-*d*).

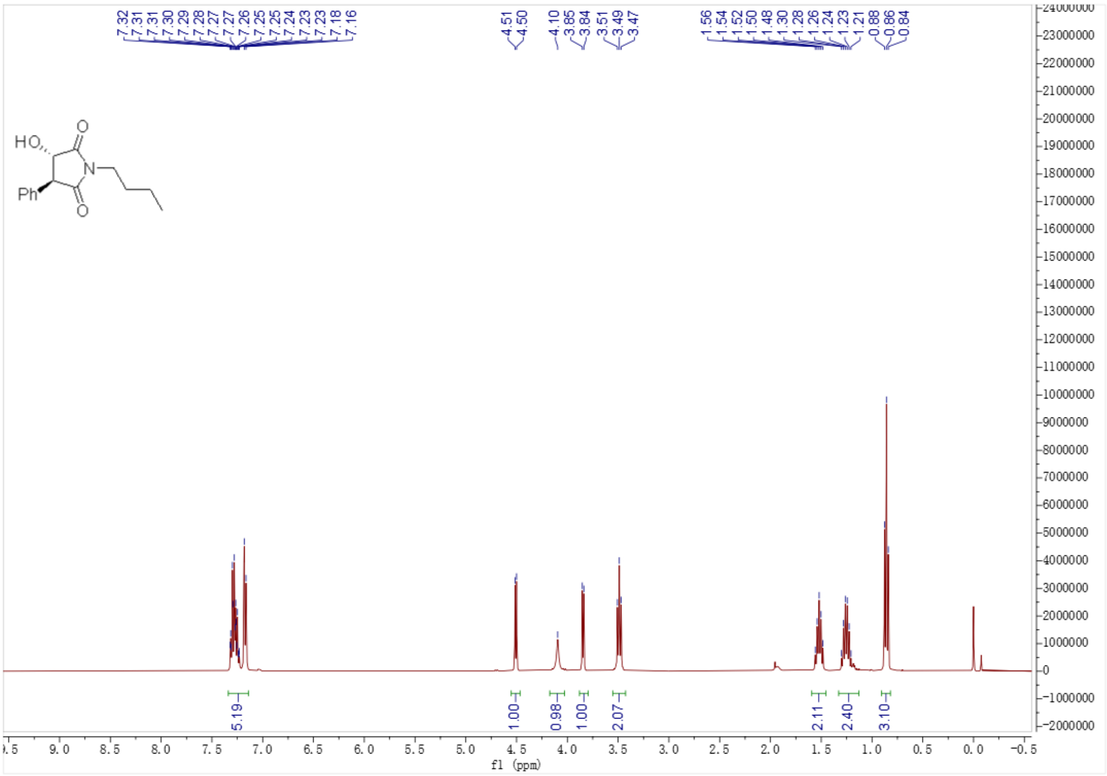

Supplementary Figure 87. ^{1}H NMR of 2e (400 MHz, Chloroform-*d*).

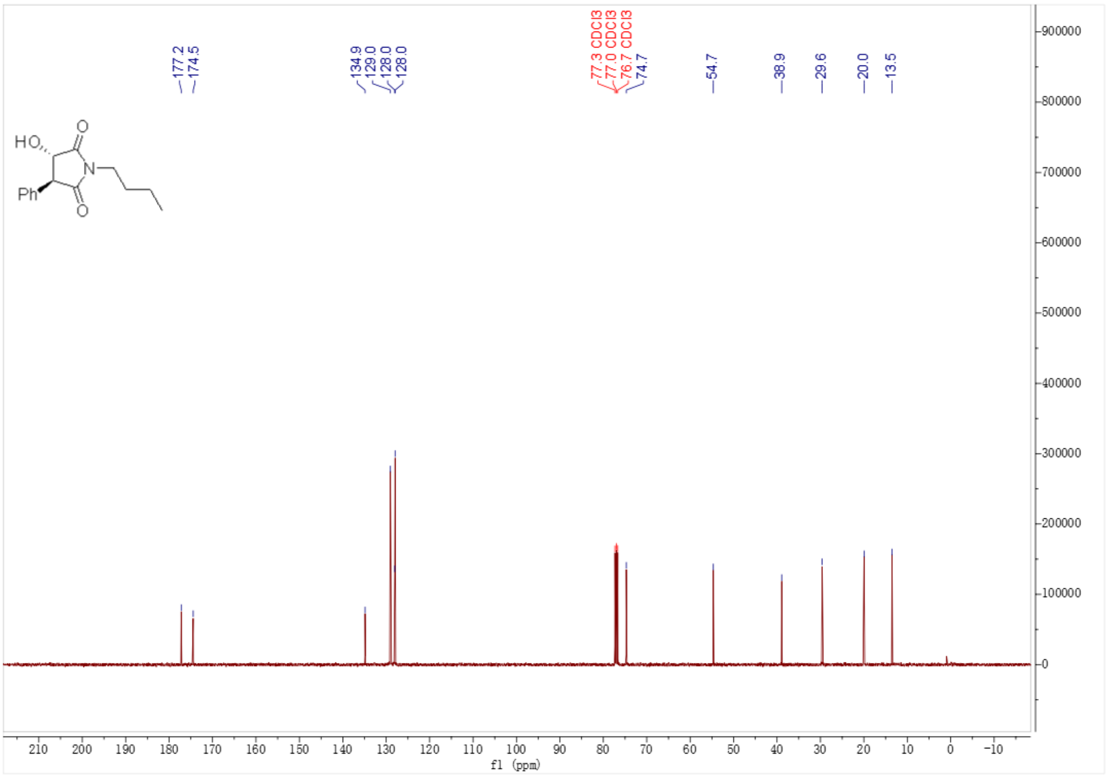

Supplementary Figure 88. ^{13}C NMR of 2e (101 MHz, Chloroform-*d*).

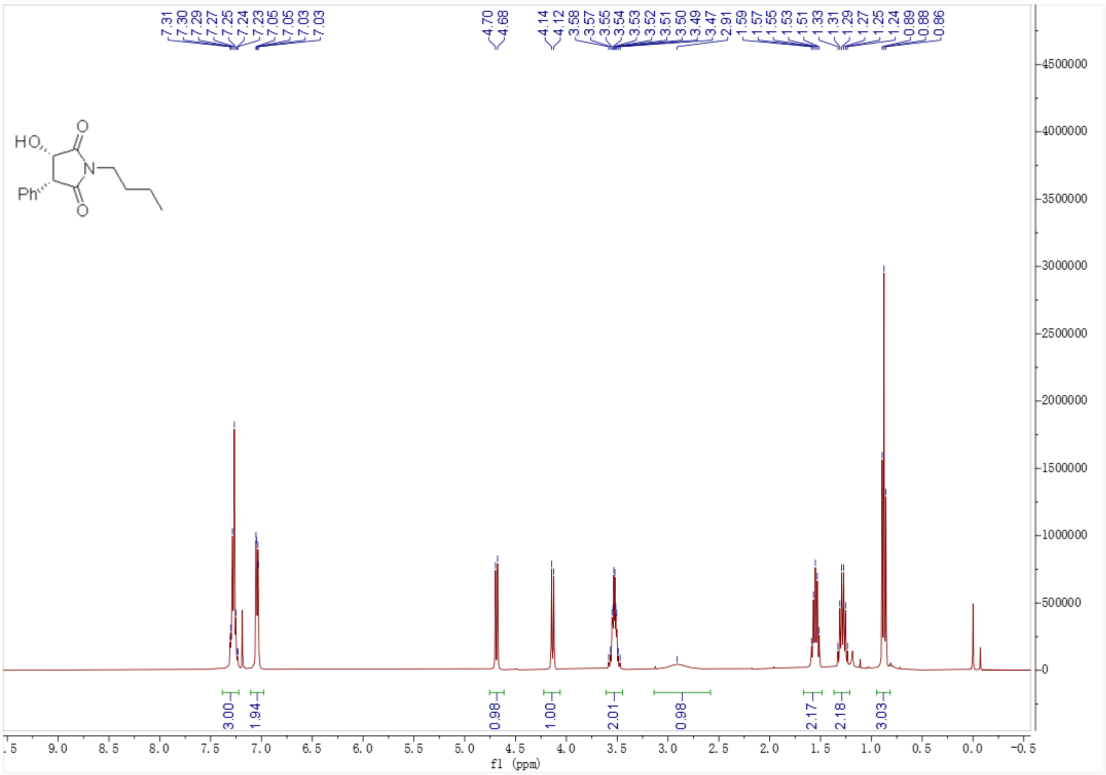

Supplementary Figure 89. ^{1}H NMR of 3e (400 MHz, Chloroform-*d*).

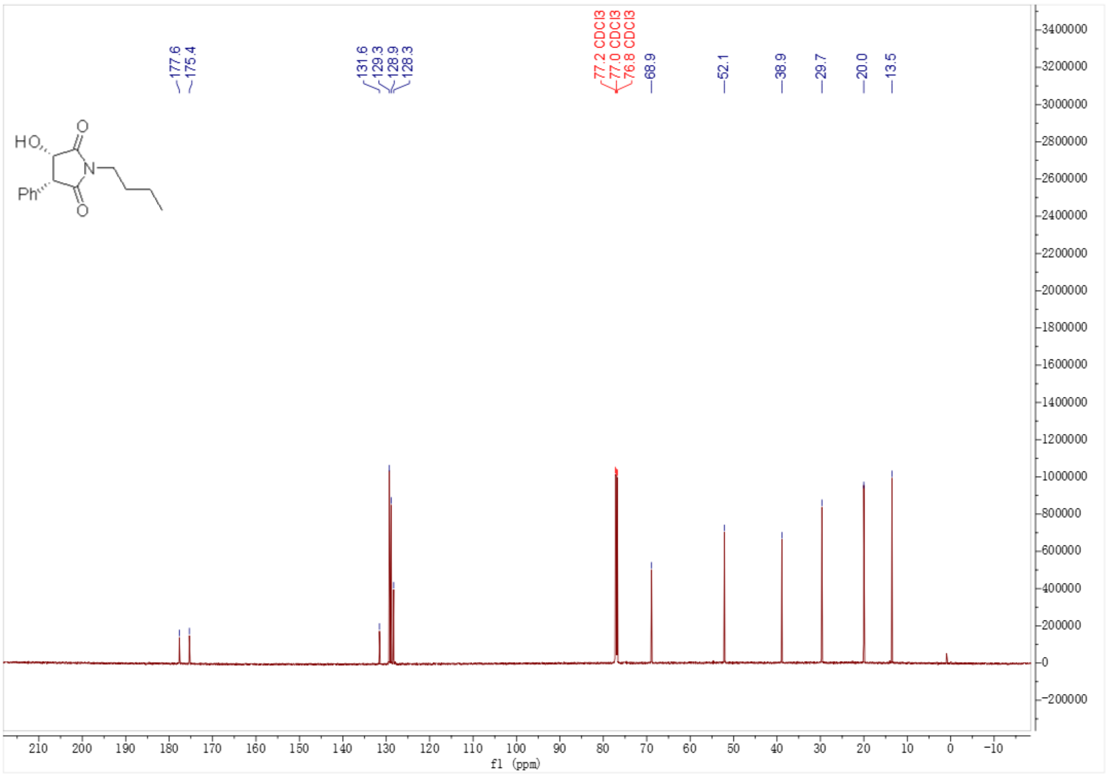

Supplementary Figure 90. ^{13}C NMR of 3e (151 MHz, Chloroform-*d*).

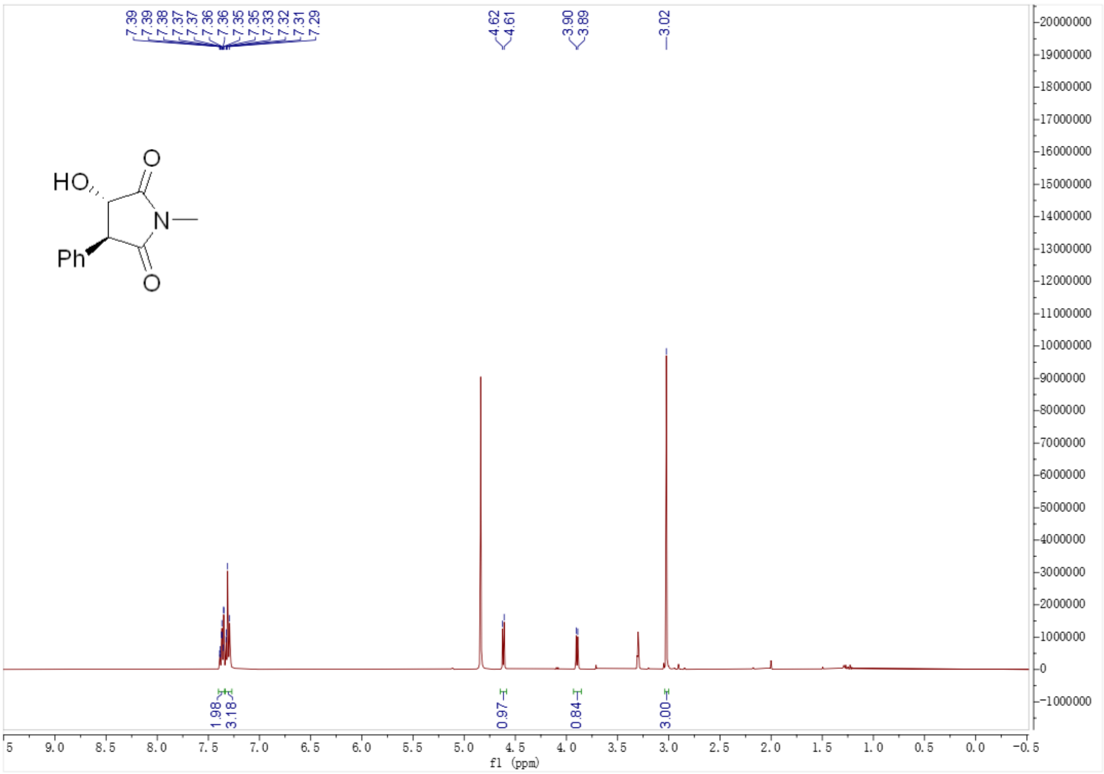

Supplementary Figure 91. ^{1}H NMR of 2f (400 MHz, Methanol-d_4).

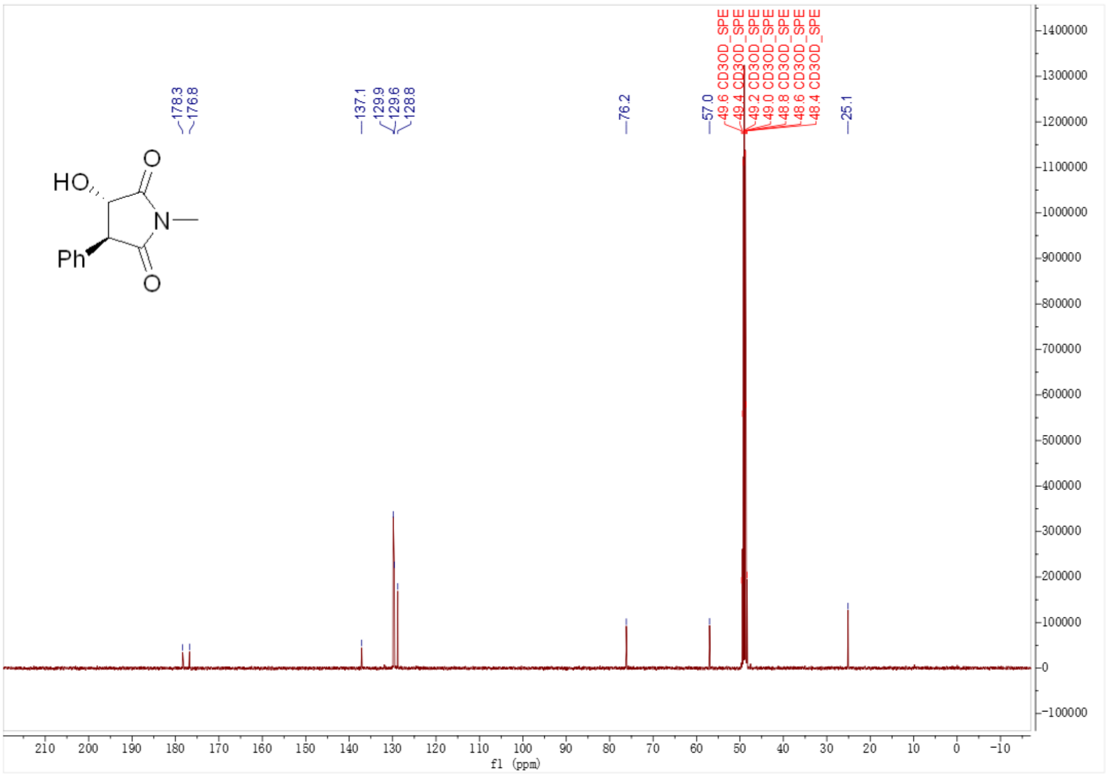

Supplementary Figure 92. ^{13}C NMR of 2f (101 MHz, Methanol-d_4).

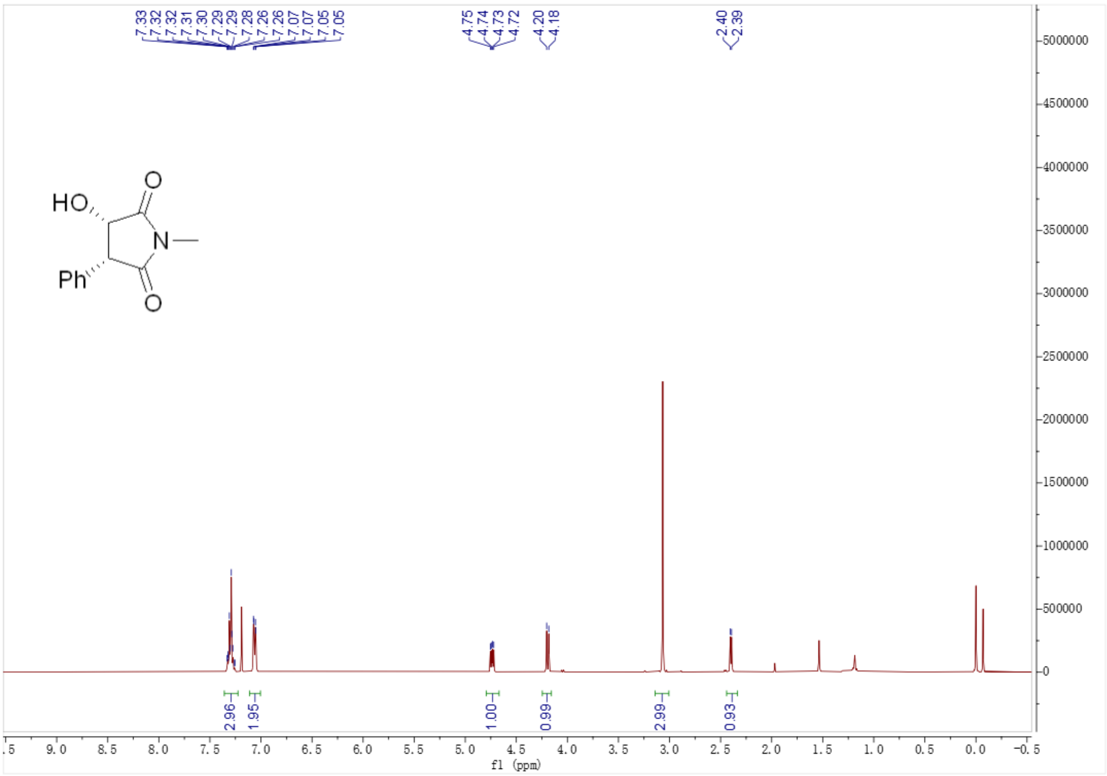

Supplementary Figure 93. ^{1}H NMR of 3f (400 MHz, Chloroform-*d*).

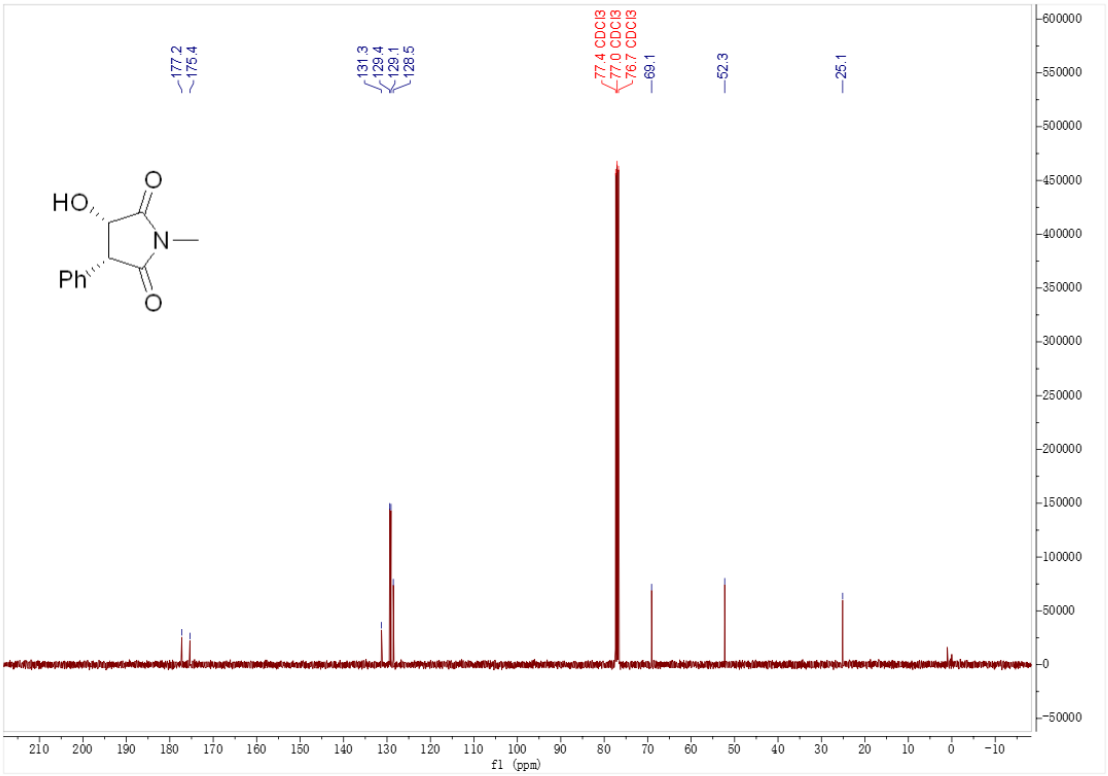

Supplementary Figure 94. ^{13}C NMR of 3f (101 MHz, Chloroform-*d*).

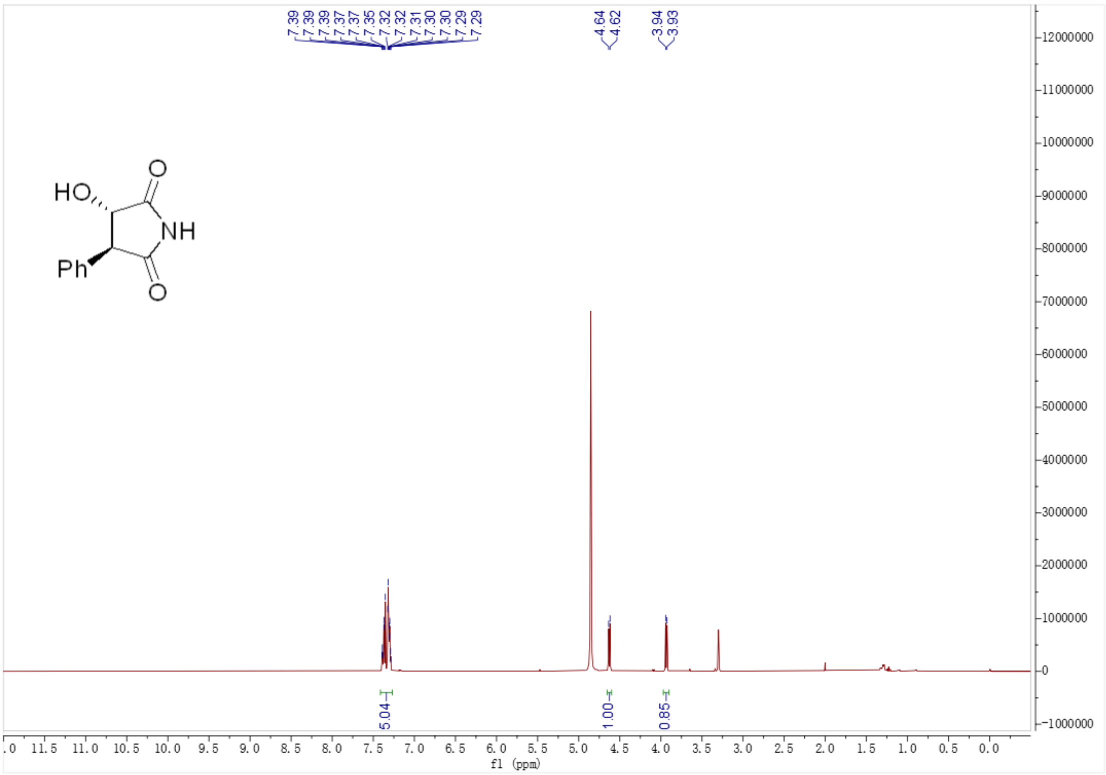

Supplementary Figure 95. ^{1}H NMR of 2g (400 MHz, Methanol-d_4).

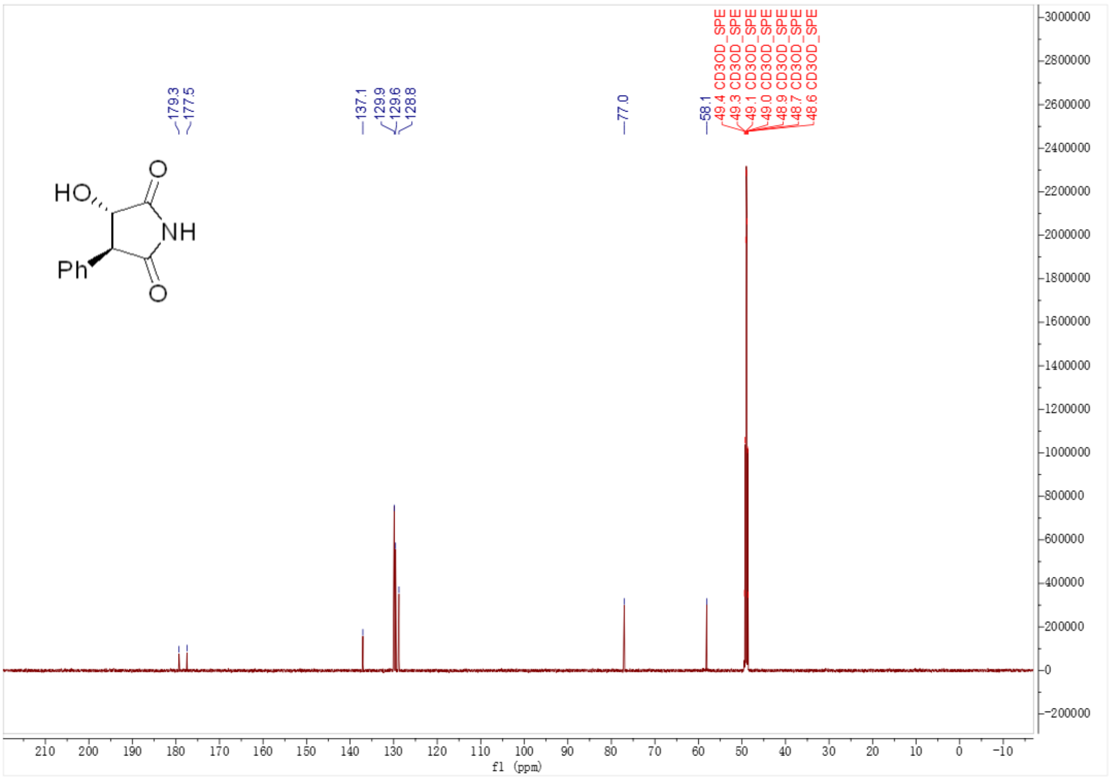

Supplementary Figure 96. ^{13}C NMR of 2g (151 MHz, Methanol-d_4).

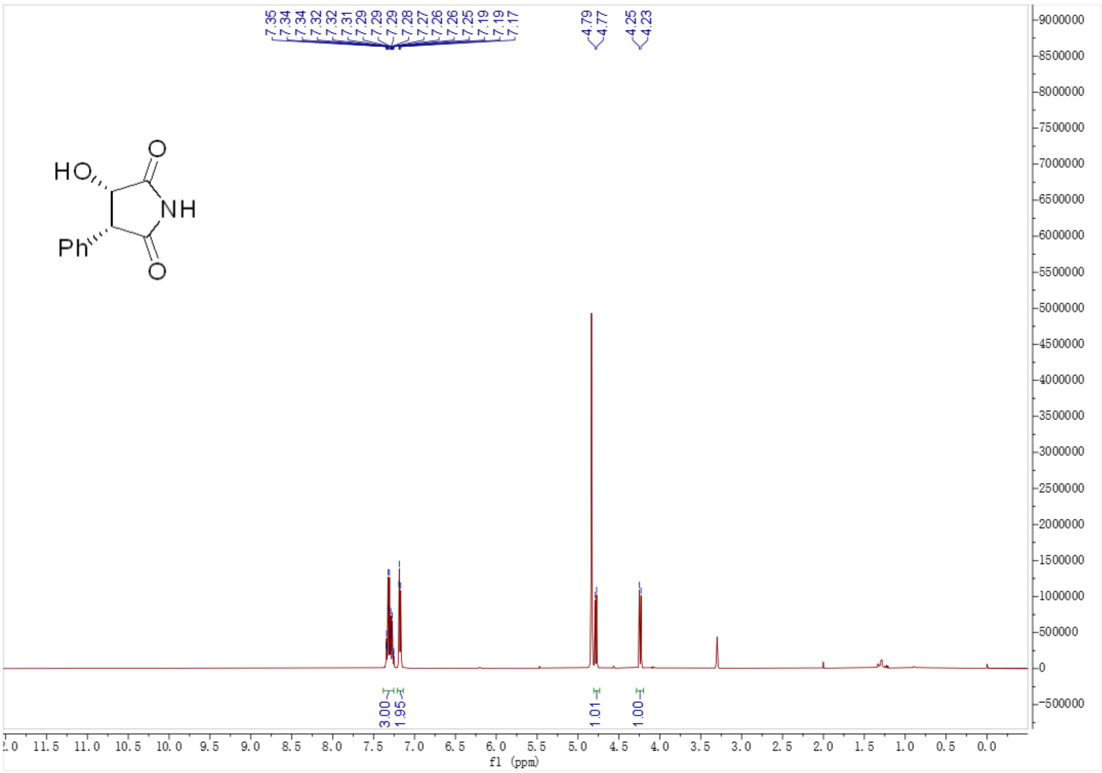

Supplementary Figure 97. ^{1}H NMR of 3g (400 MHz, Methanol-d_4).

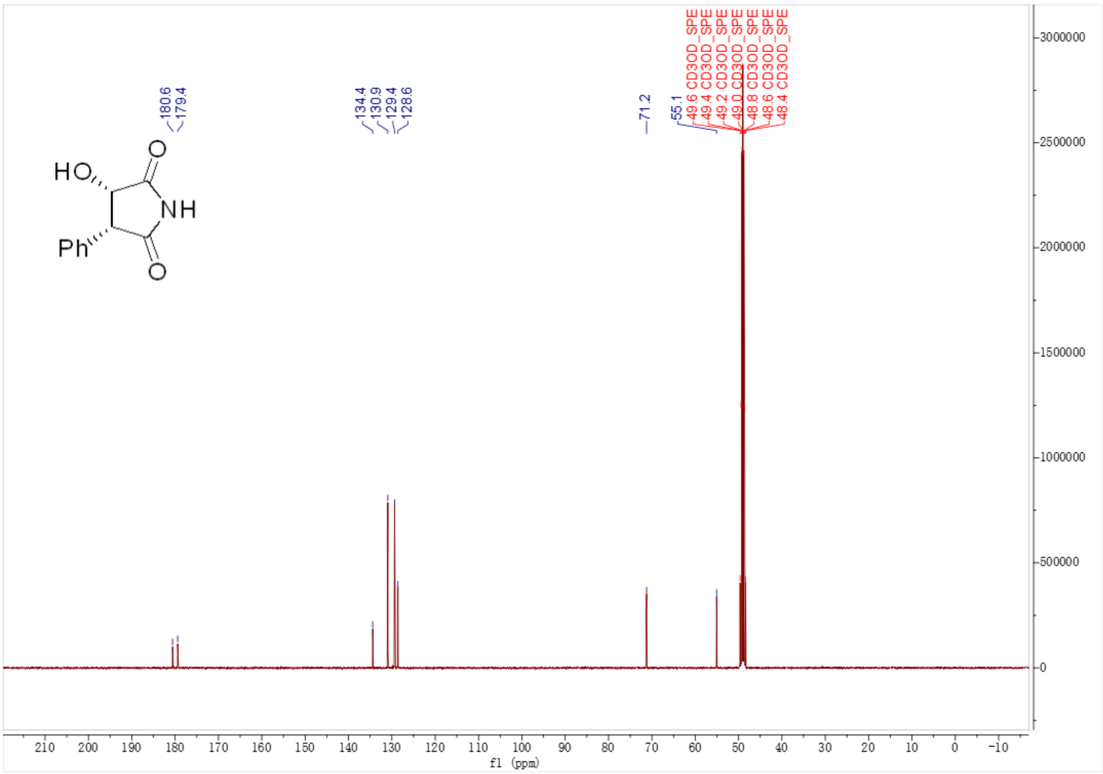

Supplementary Figure 98. ^{13}C NMR of 3g (101 MHz, Methanol-d_4).

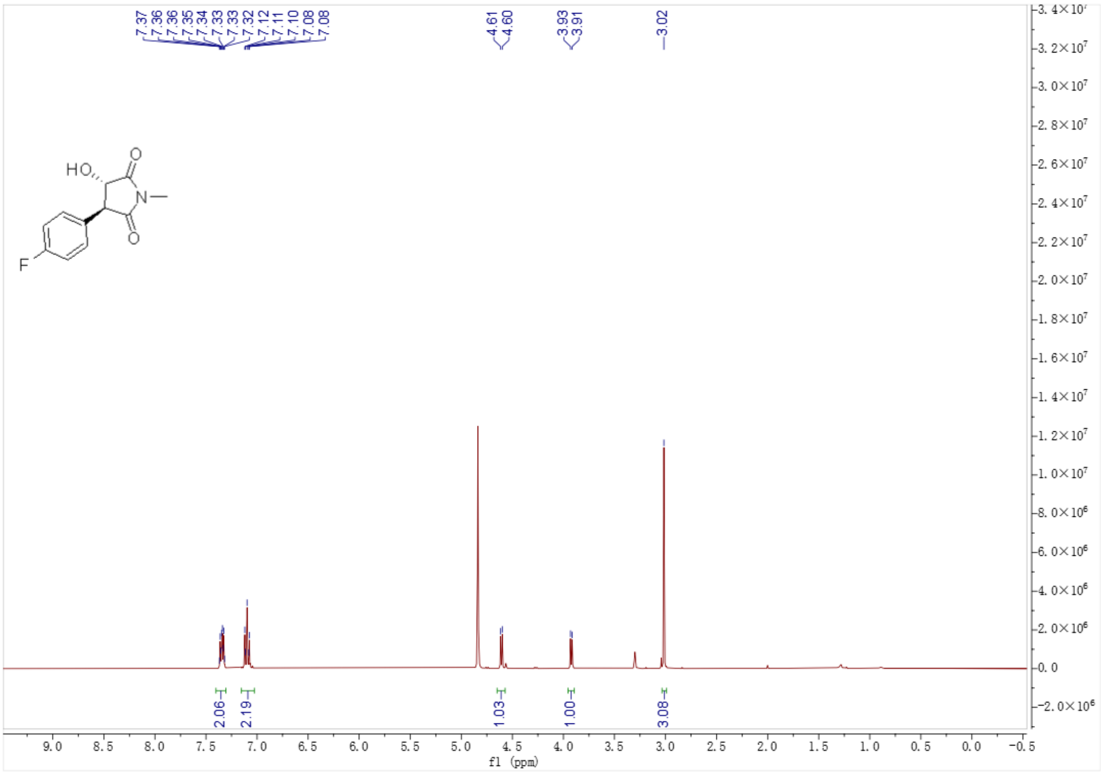

Supplementary Figure 99. ^{1}H NMR of 2h (400 MHz, Methanol-d_4).

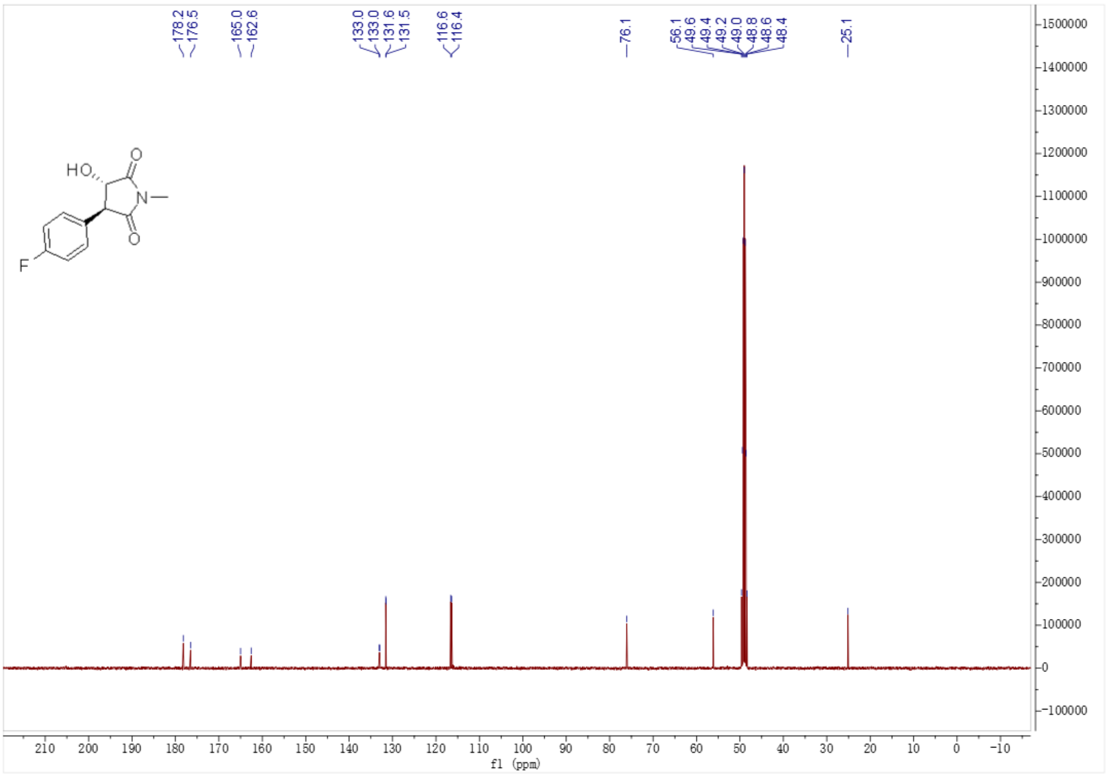

Supplementary Figure 100. ^{13}C NMR of 2h (101 MHz, Methanol-d_4).

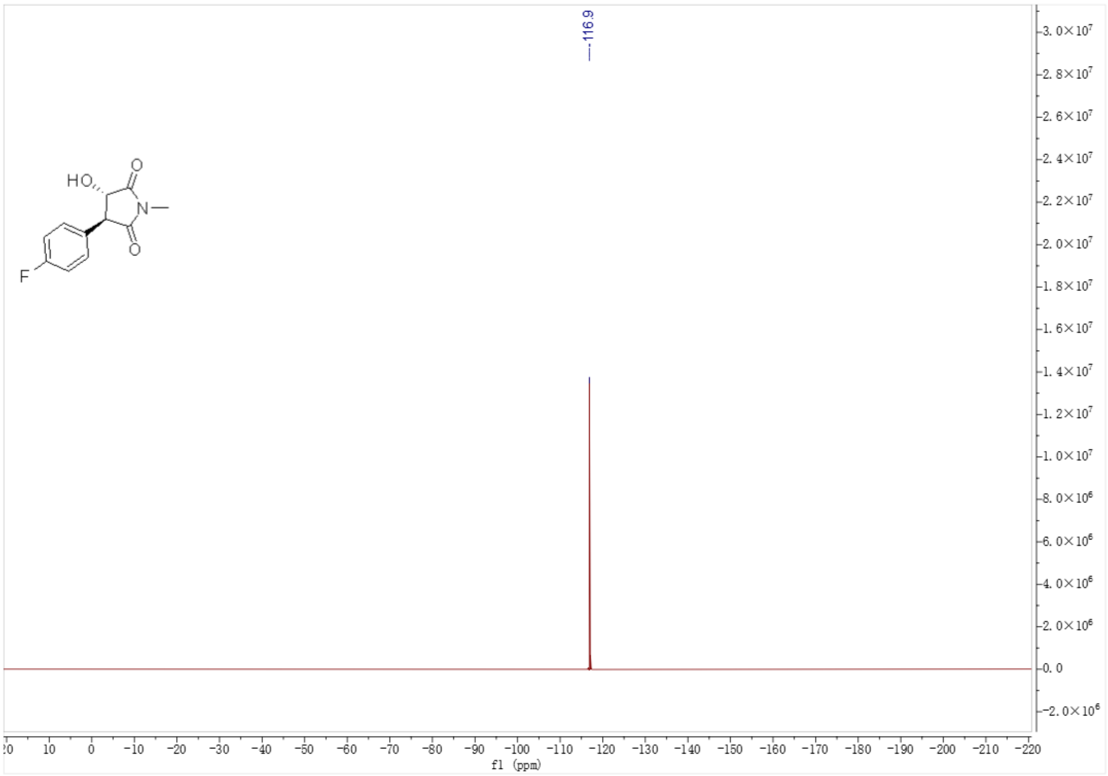

Supplementary Figure 101. ^{19}F NMR of 2h (376 MHz, Methanol-d_4).

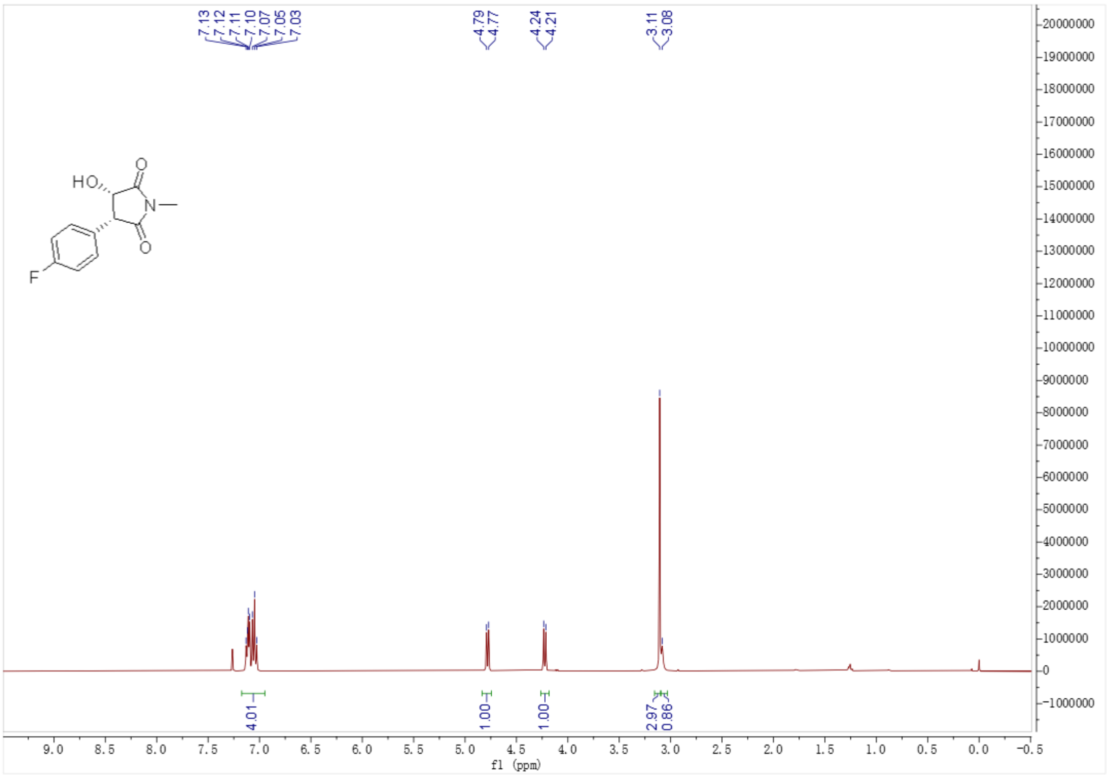

Supplementary Figure 102. ^{1}H NMR of 3h (400 MHz, Chloroform-d).

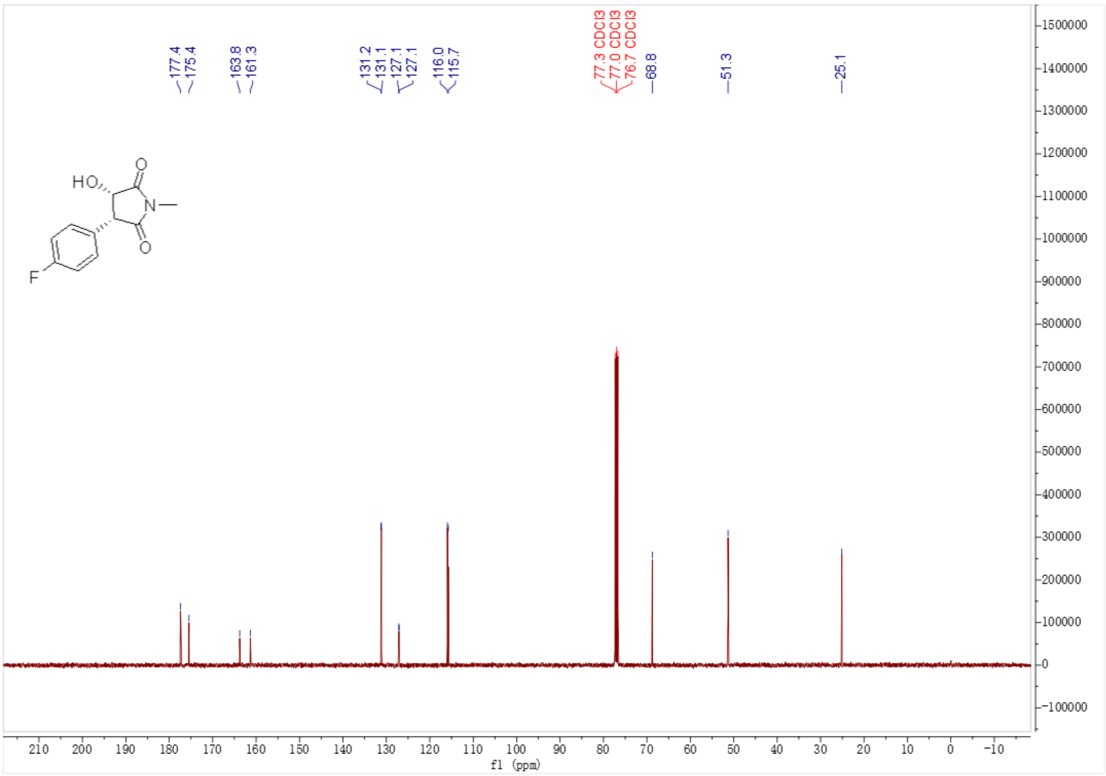

Supplementary Figure 103. ^{13}**C NMR of 3h (101 MHz, Chloroform-*d*).**

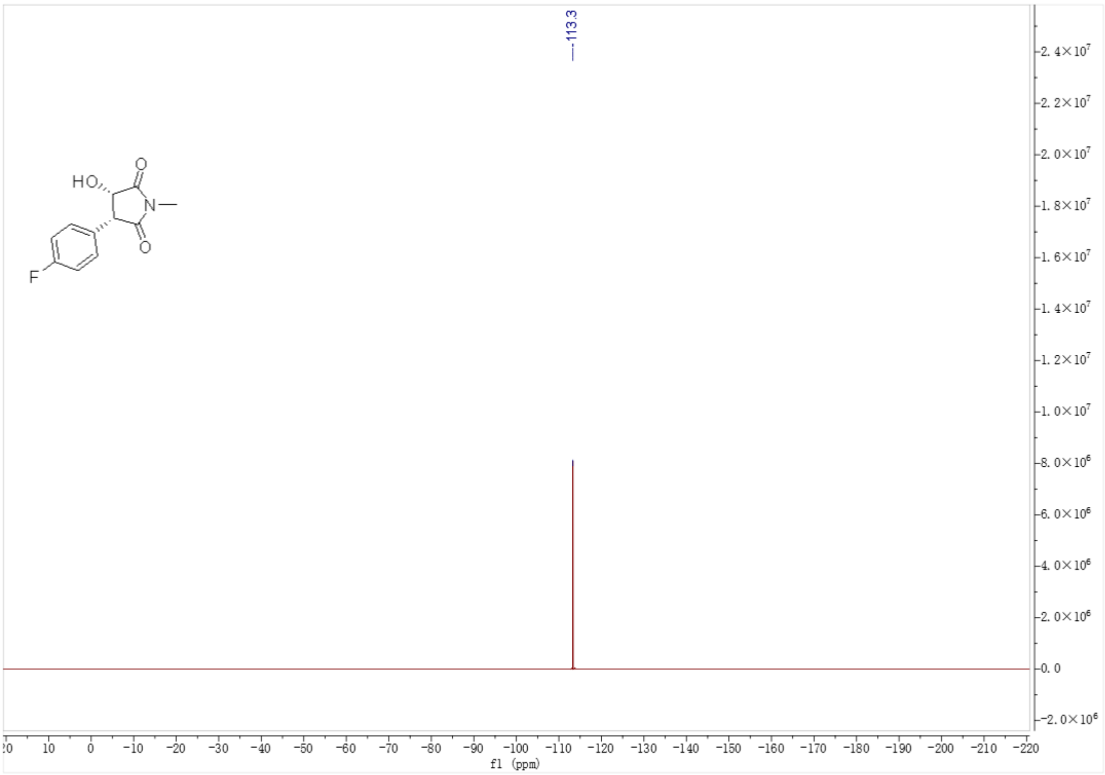

Supplementary Figure 104. ^{19}**F NMR of 3h (376 MHz, Chloroform-*d*).**

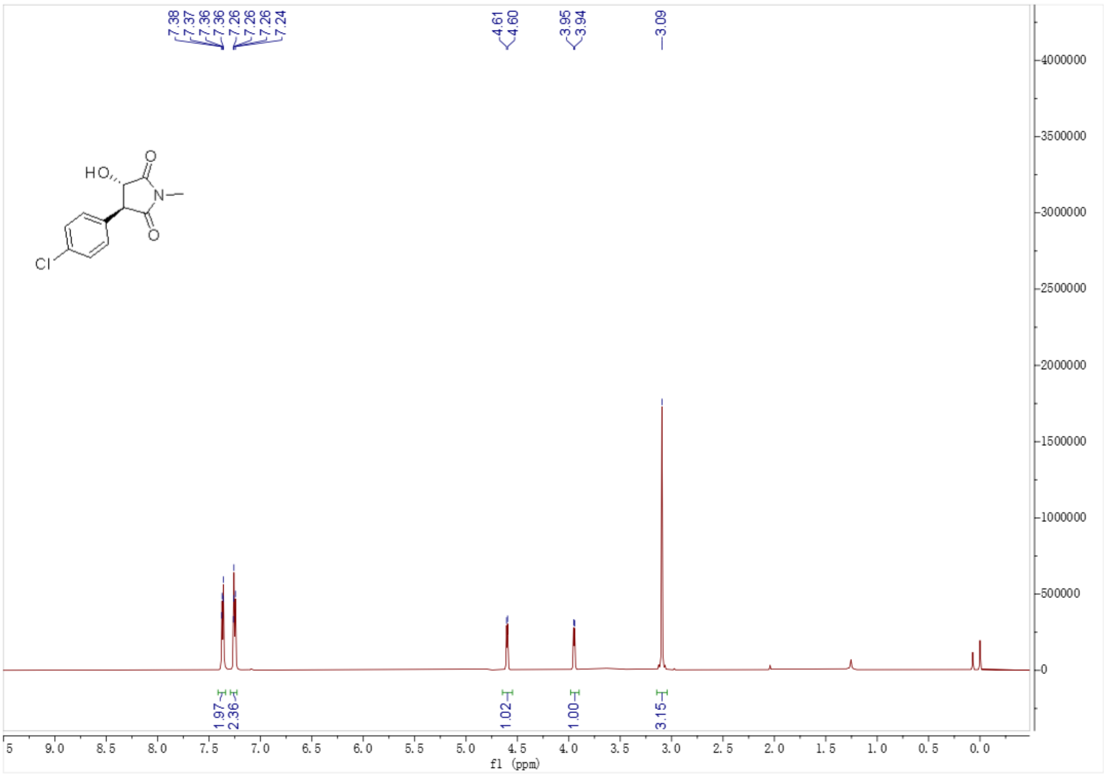

Supplementary Figure 105. ^{1}H NMR of 2i (600 MHz, Chloroform-*d*).

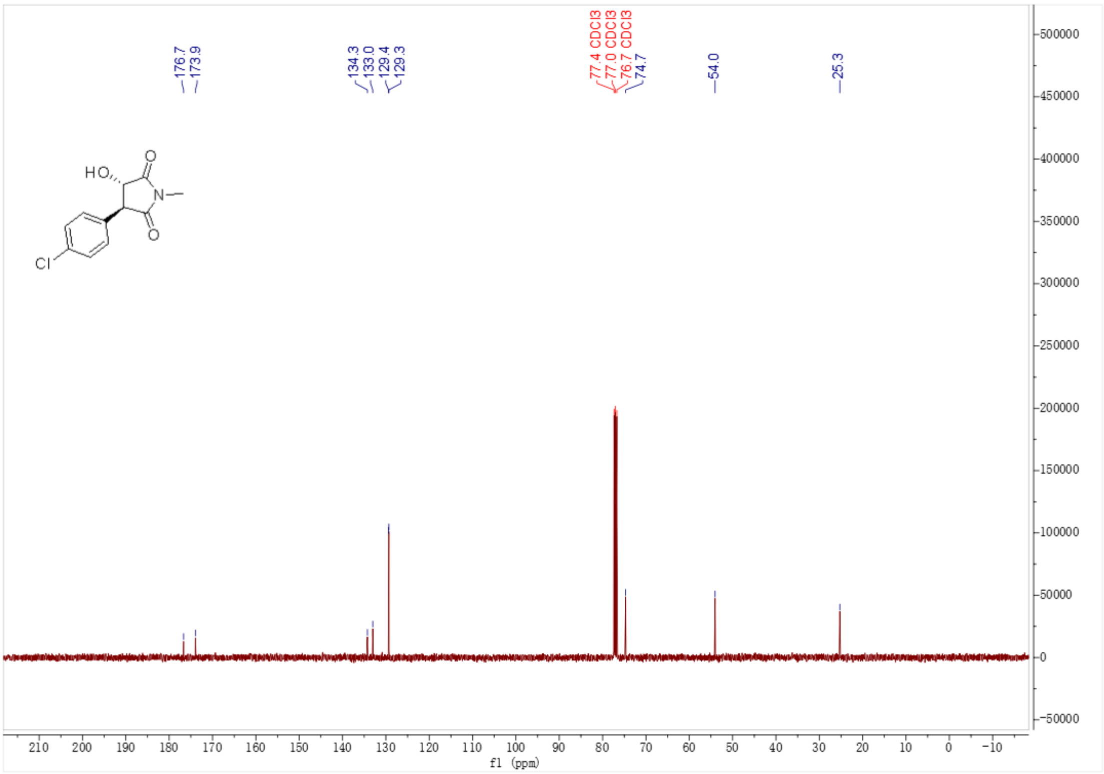

Supplementary Figure 106. ^{13}C NMR of 2i (101 MHz, Chloroform-*d*).

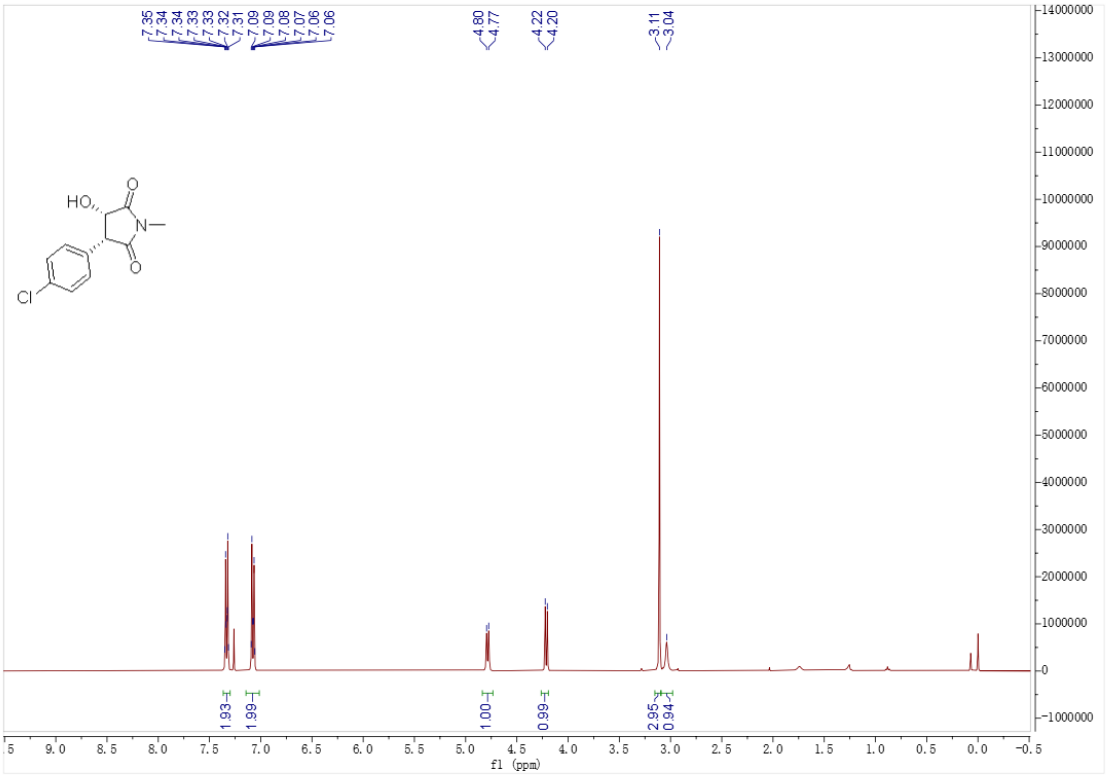

Supplementary Figure 107. ^{1}H NMR of 3i (400 MHz, Chloroform-*d*).

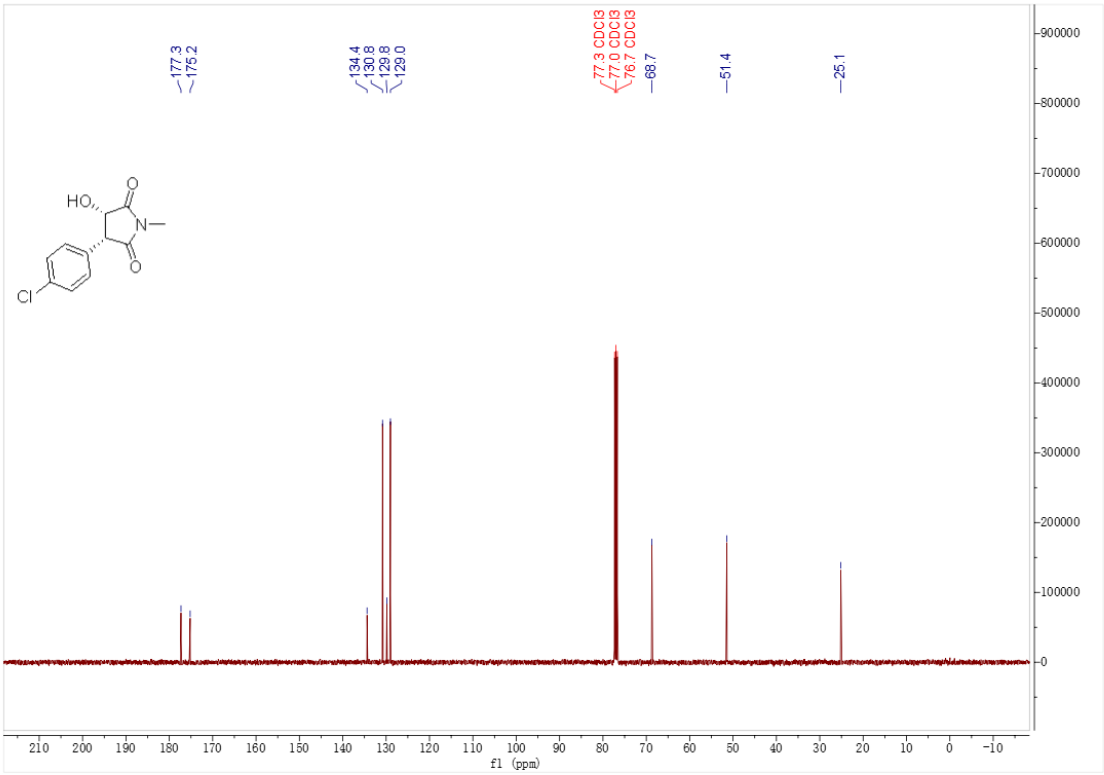

Supplementary Figure 108. ^{13}C NMR of 3i (101 MHz, Chloroform-*d*).

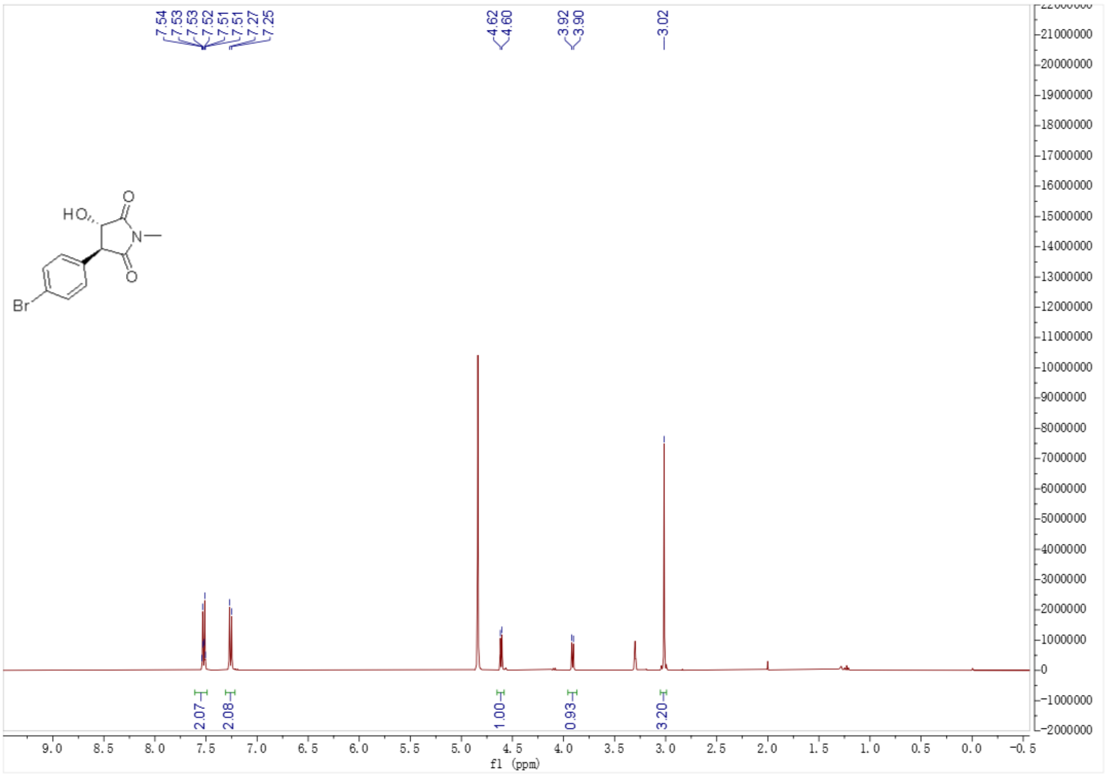

Supplementary Figure 109. **^{1}H NMR of 2j (400 MHz, Methanol-d_4).**

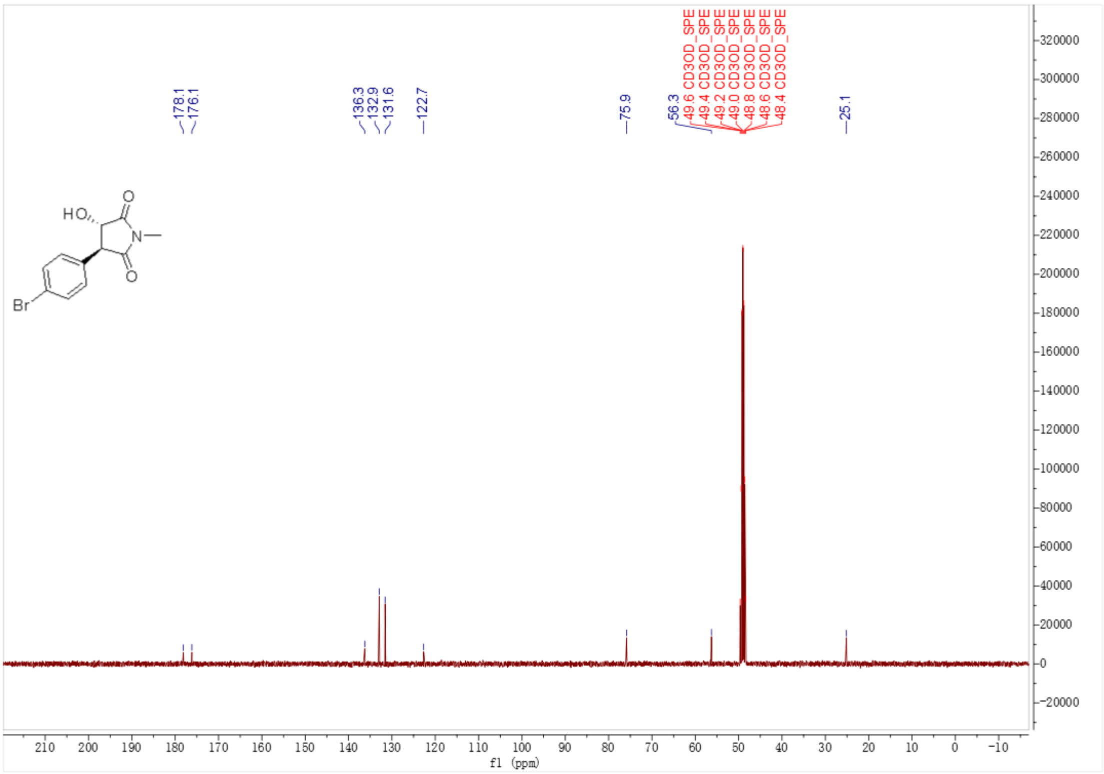

Supplementary Figure 110. **^{13}C NMR of 2j (101 MHz, Methanol-d_4).**

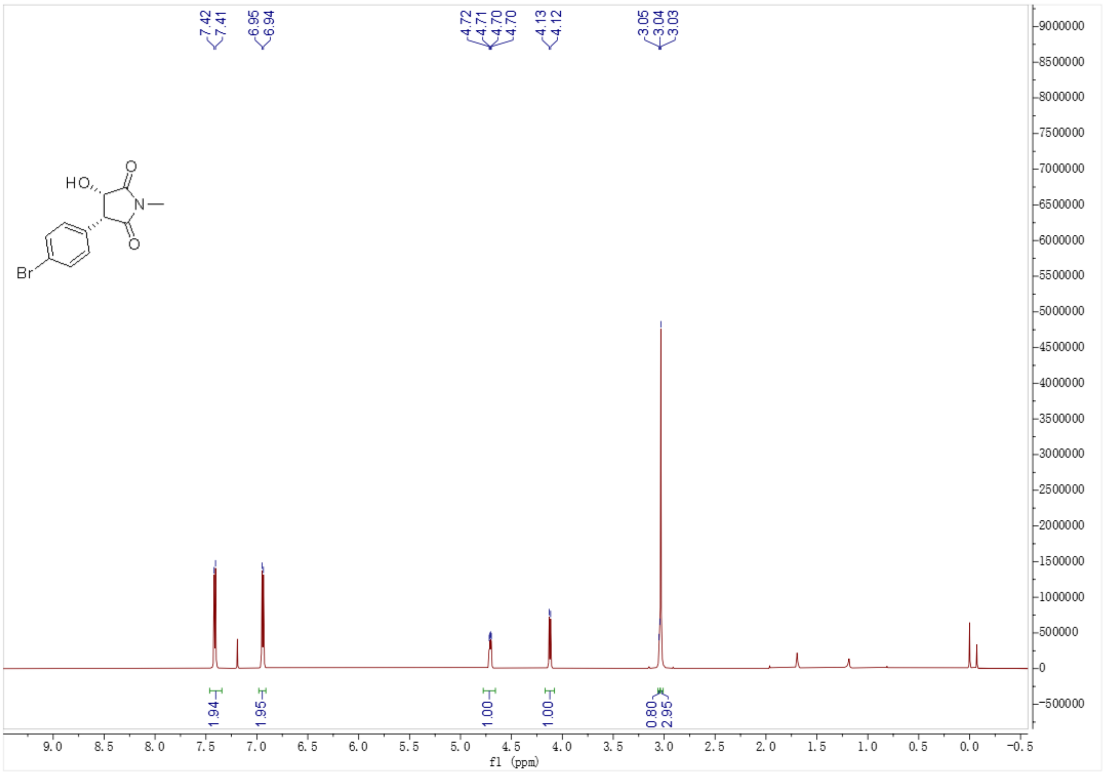

Supplementary Figure 111. ^{1}**H NMR of 3j (600 MHz, Chloroform-*d*).**

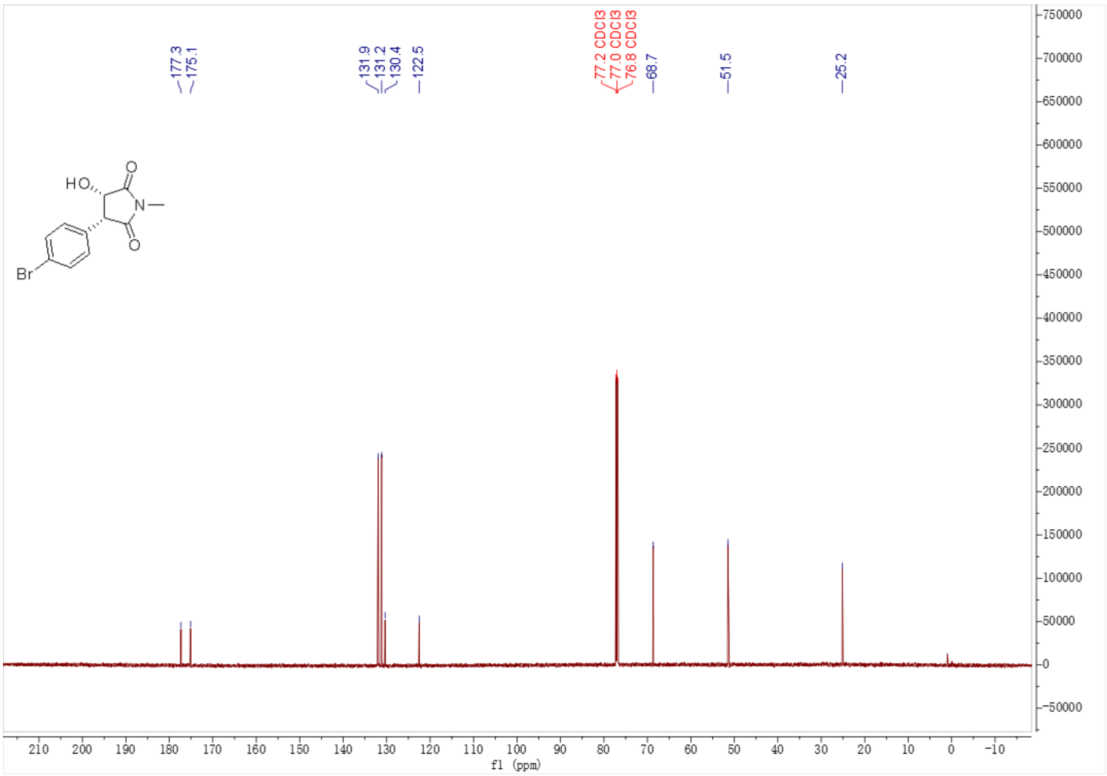

Supplementary Figure 112. ^{13}**C NMR of 3j (151 MHz, Chloroform-*d*).**

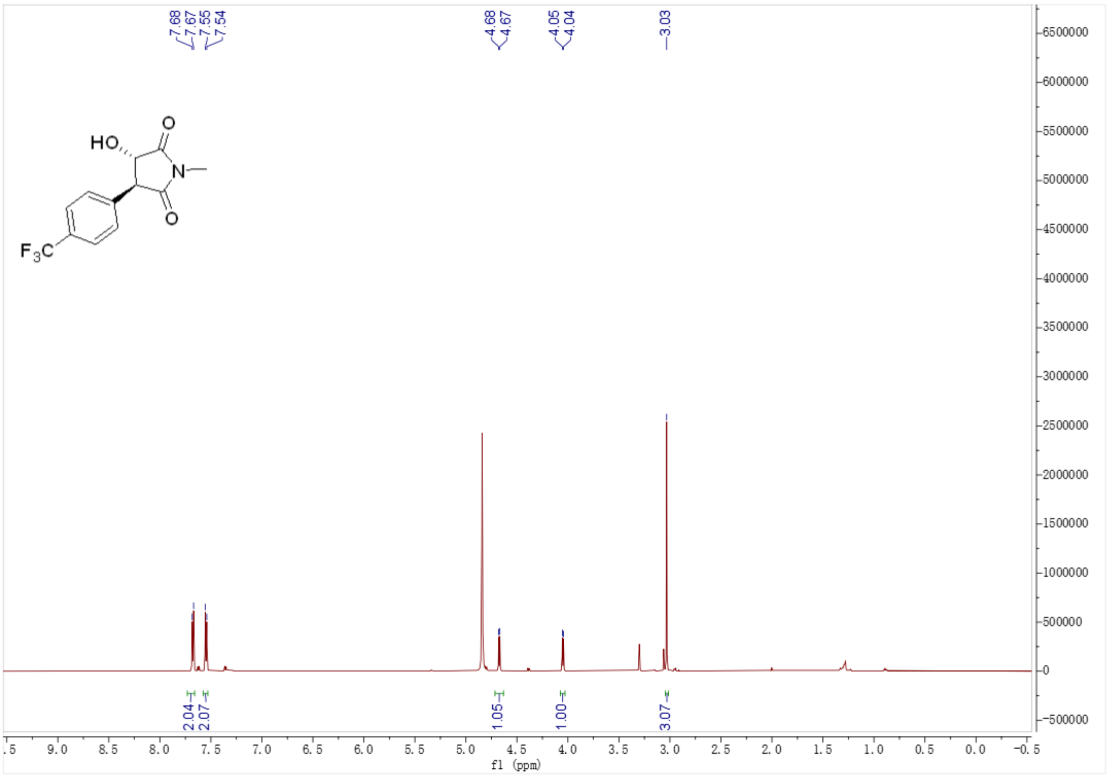

Supplementary Figure 113. ^{1}H NMR of 2k (600 MHz, Methanol-d_4).

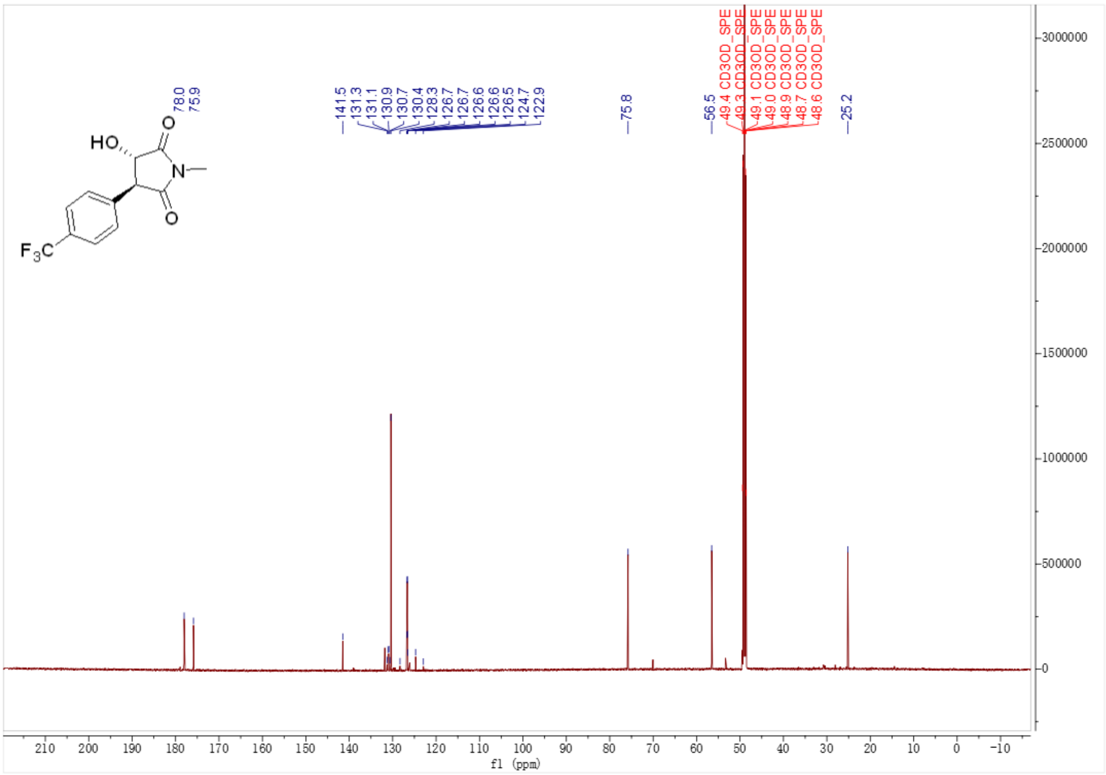

Supplementary Figure 114. ^{13}C NMR of 2k (151 MHz, Methanol-d_4).

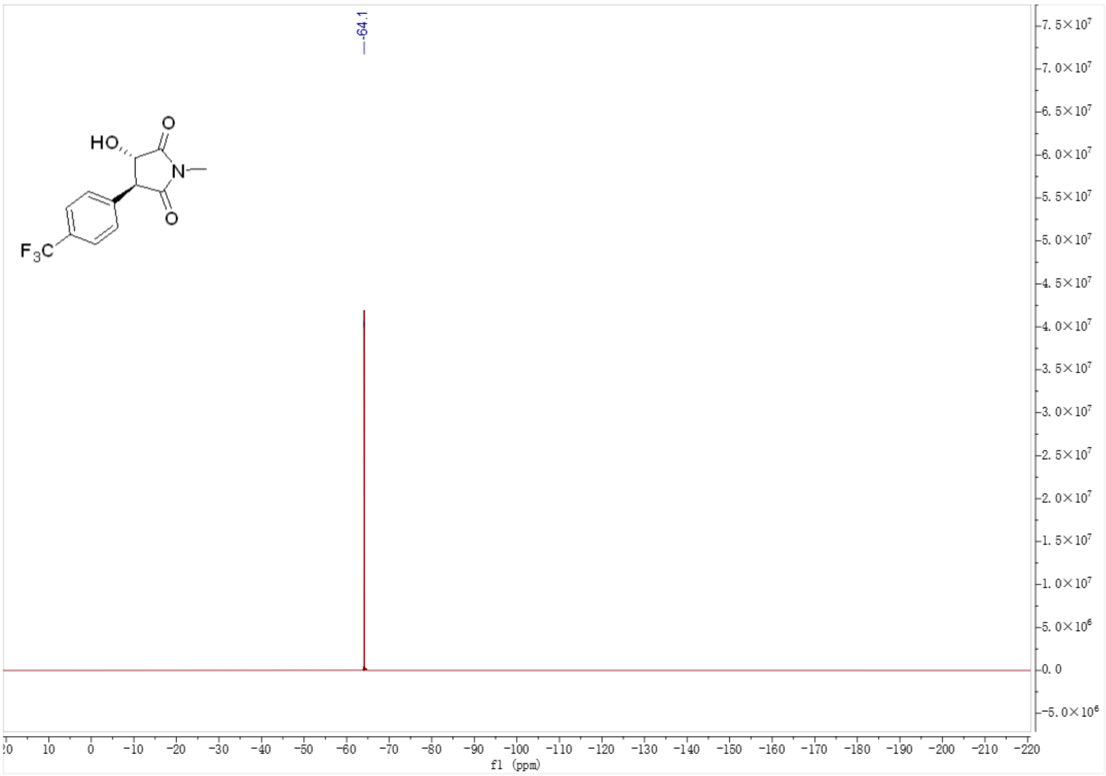

Supplementary Figure 115. ^{19}**F NMR of 2k (376 MHz, Methanol-*d*$_4$).**

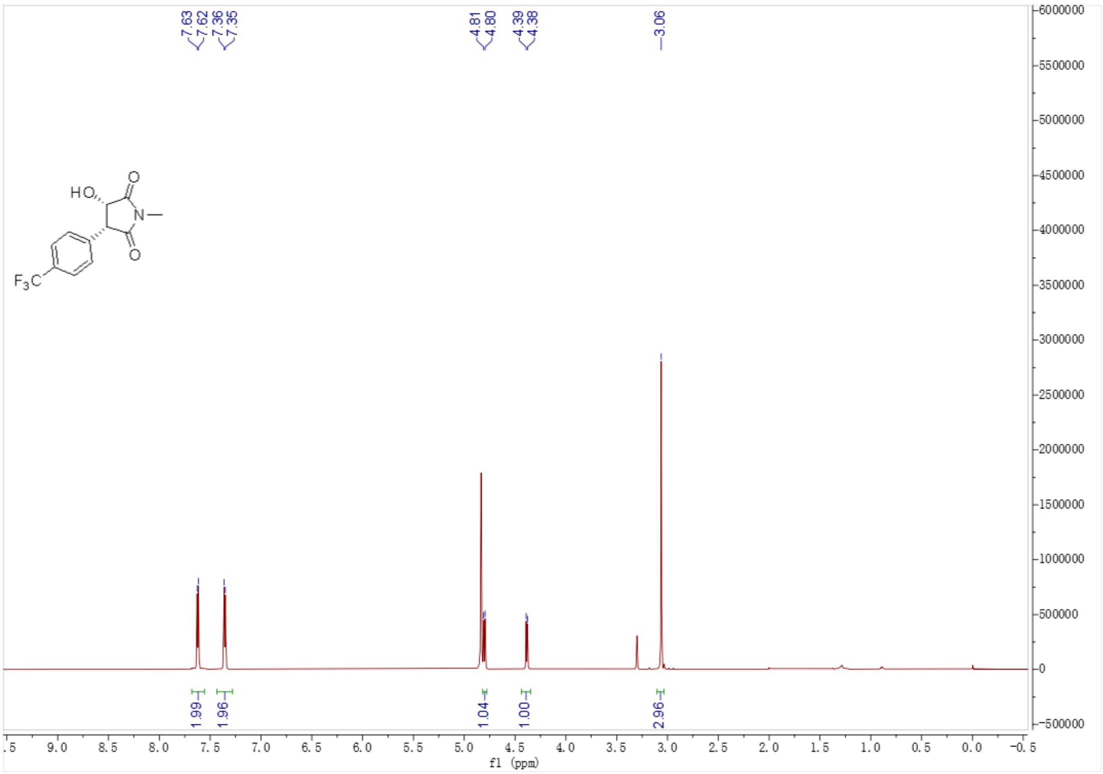

Supplementary Figure 116. ^{1}**H NMR of 3k (600 MHz, Methanol-*d*$_4$).**

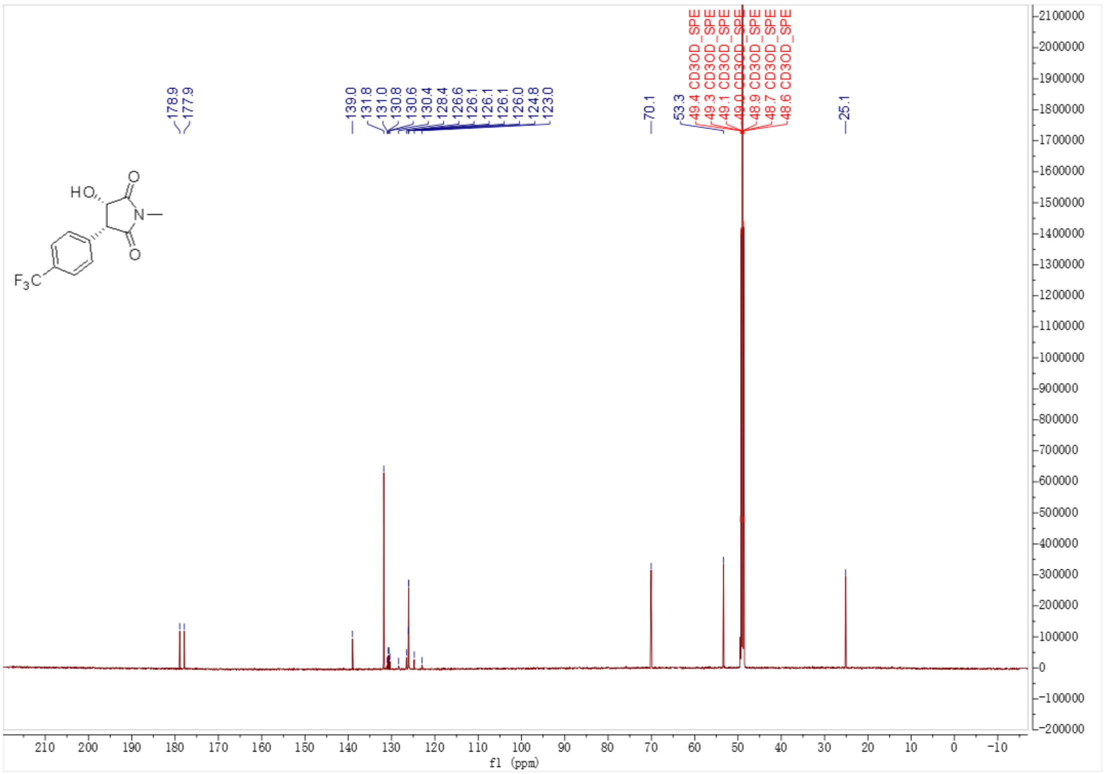

Supplementary Figure 117. ^{13}C NMR of 3k (151 MHz, Methanol-d_4).

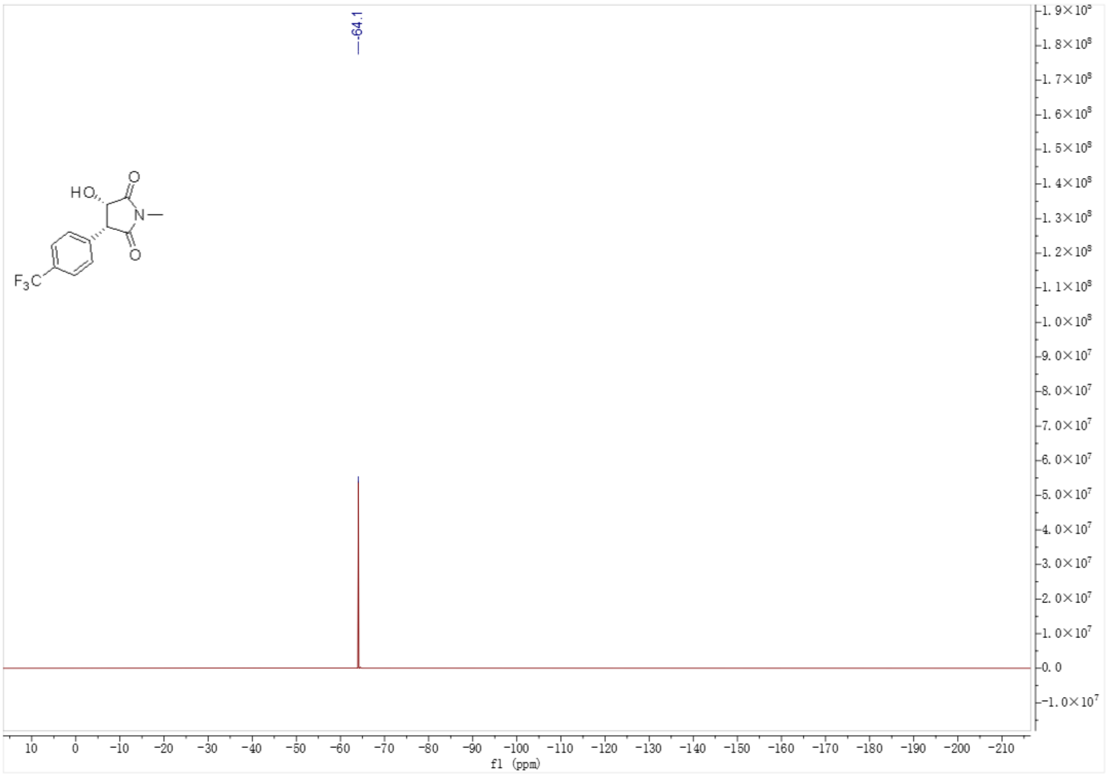

Supplementary Figure 118. ^{19}F NMR of 3k (565 MHz, Methanol-d_4).

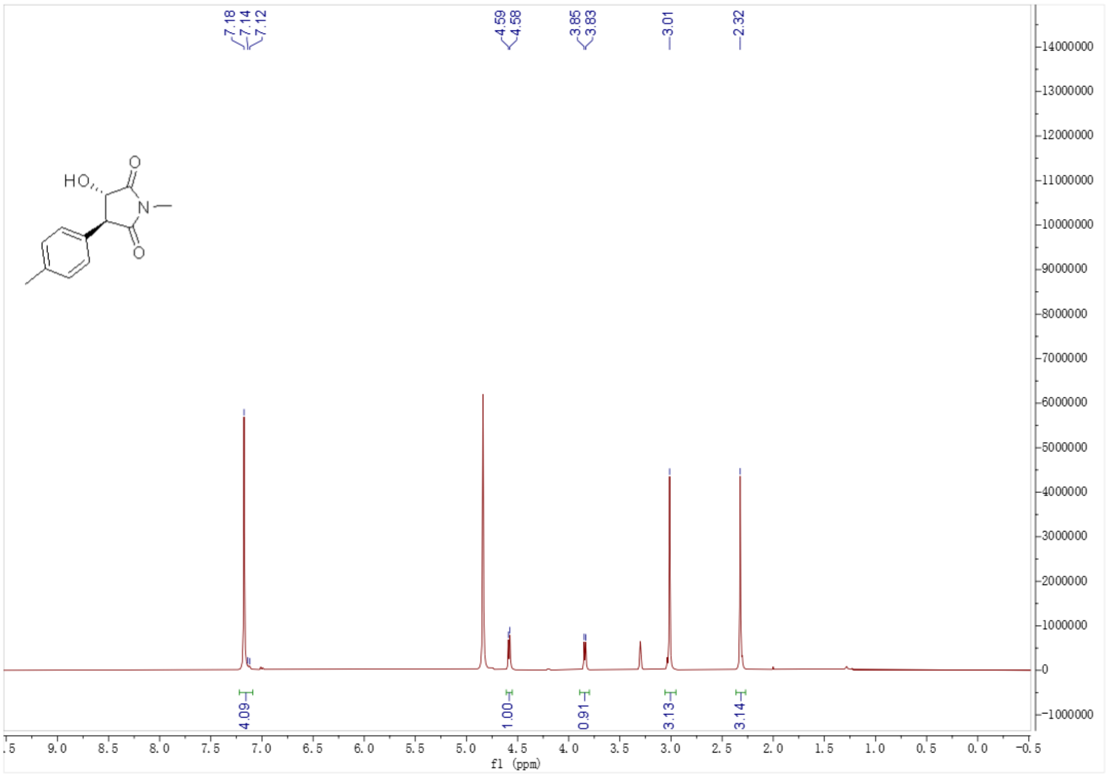

Supplementary Figure 119. **^{1}H NMR of 2l (400 MHz, Methanol-d_4).**

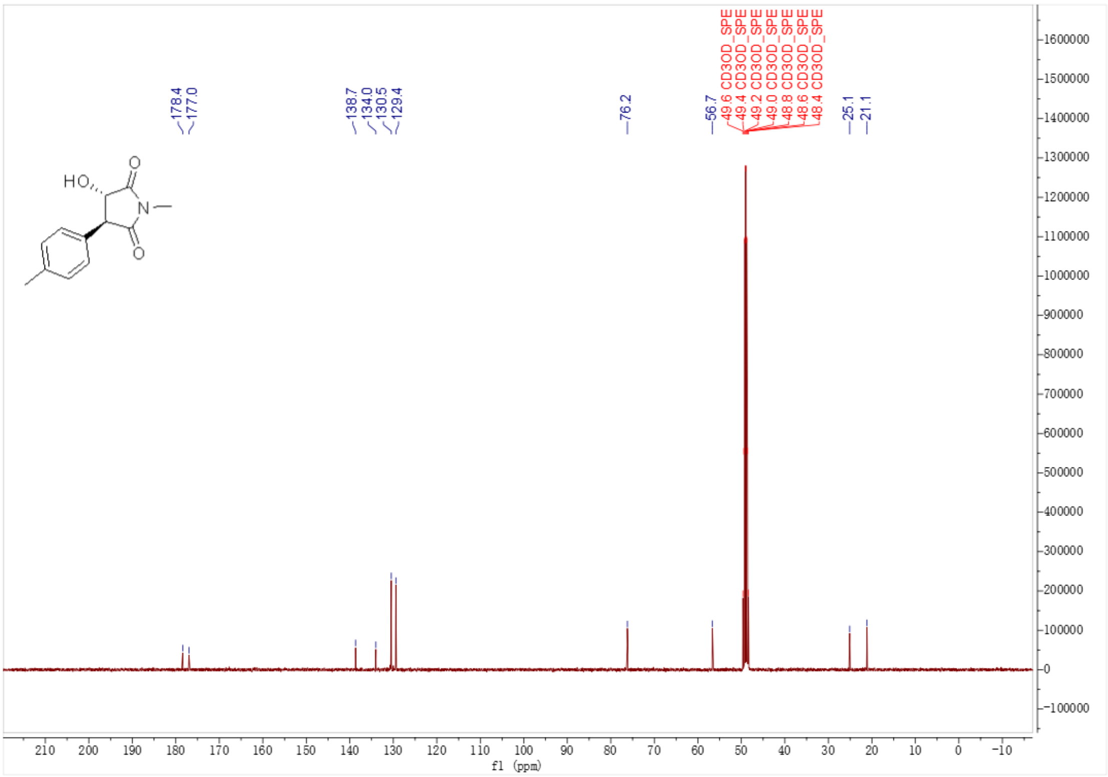

Supplementary Figure 120. **^{13}C NMR of 2l (101 MHz, Methanol-d_4).**

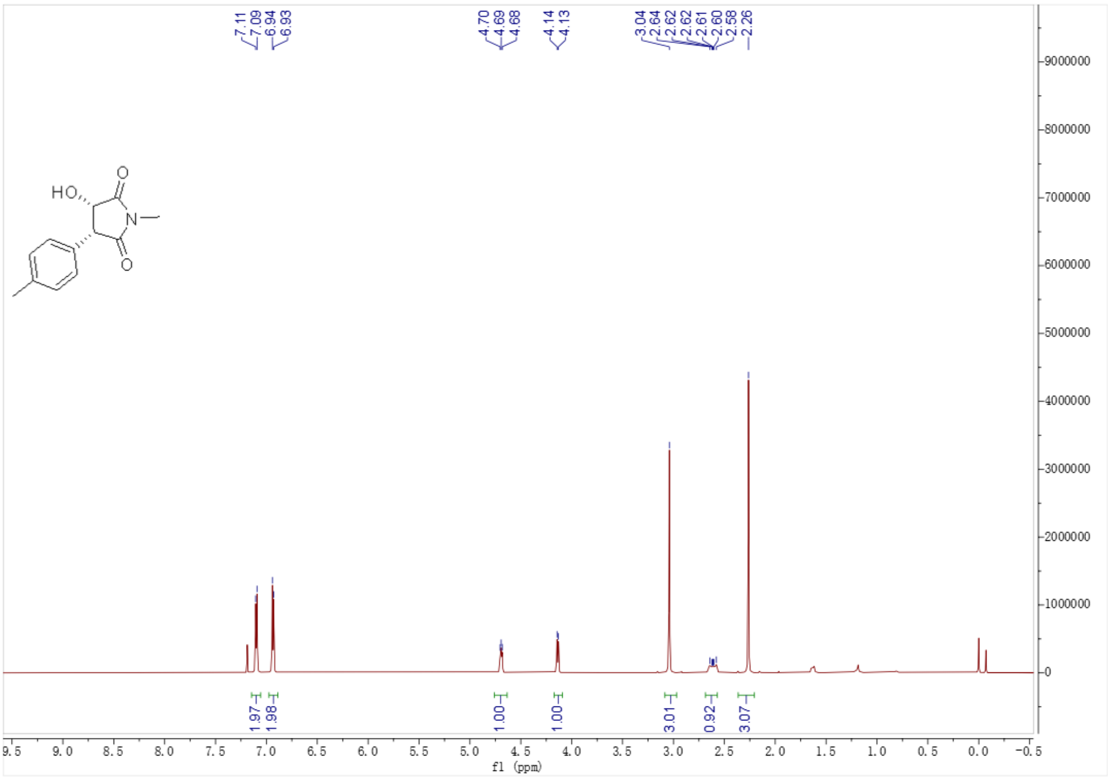

Supplementary Figure 121. ^{1}H NMR of 3l (600 MHz, Chloroform-*d*).

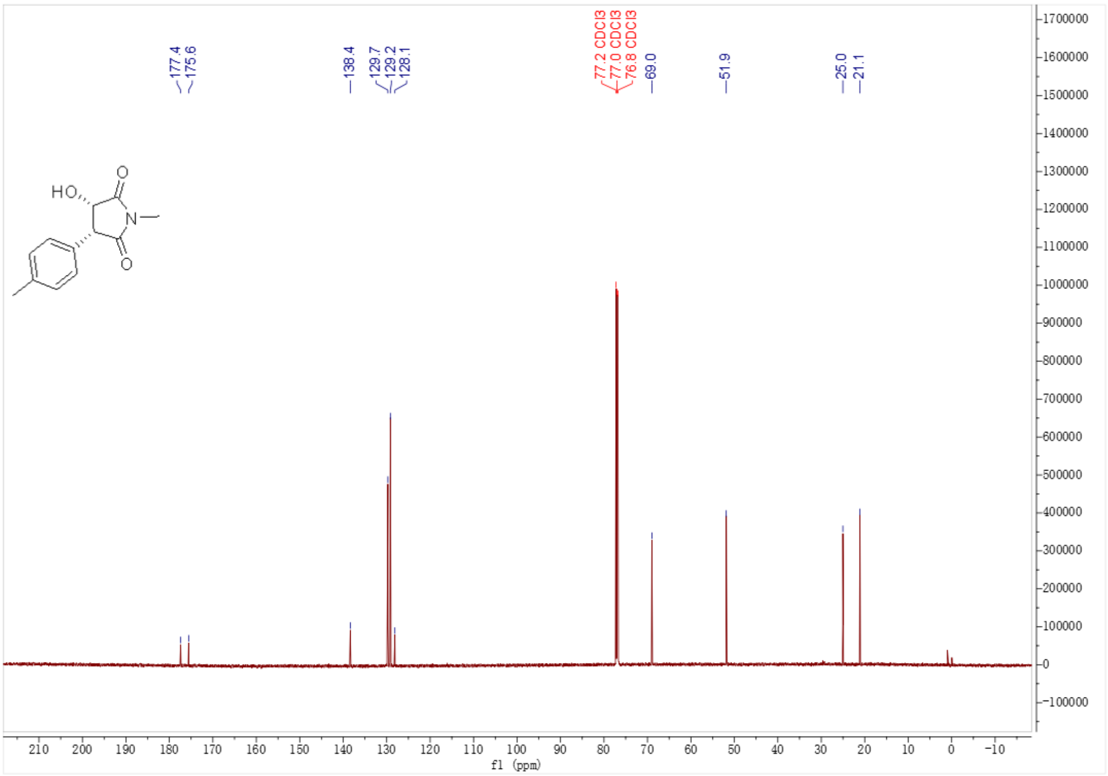

Supplementary Figure 122. ^{13}C NMR of 3l (151 MHz, Chloroform-*d*).

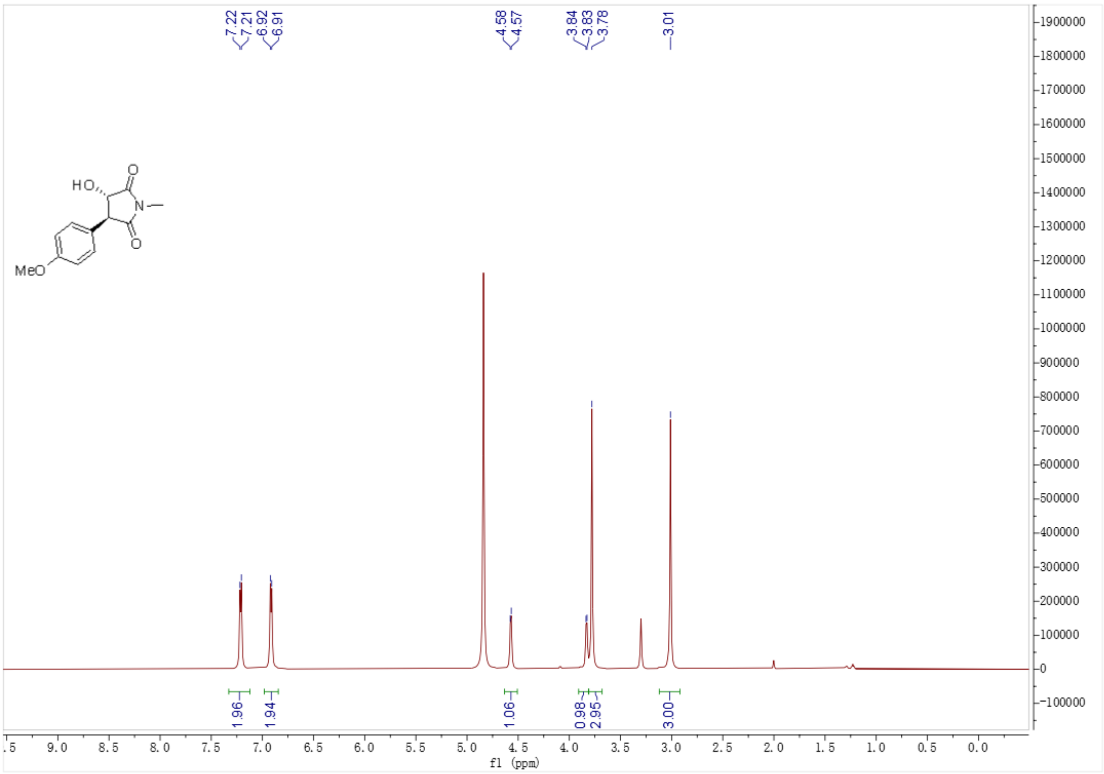

Supplementary Figure 123. ^{1}**H NMR of 2m (600 MHz, Methanol-*d*$_4$).**

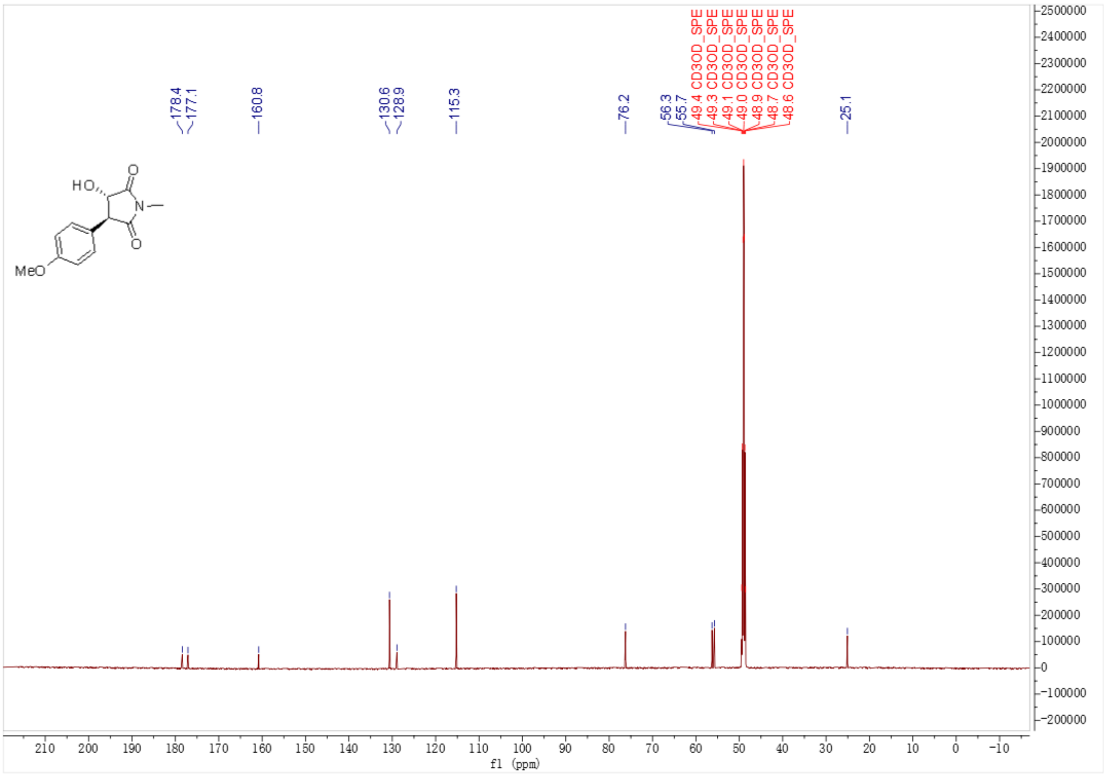

Supplementary Figure 124. ^{13}**C NMR of 2m (151 MHz, Methanol-*d*$_4$).**

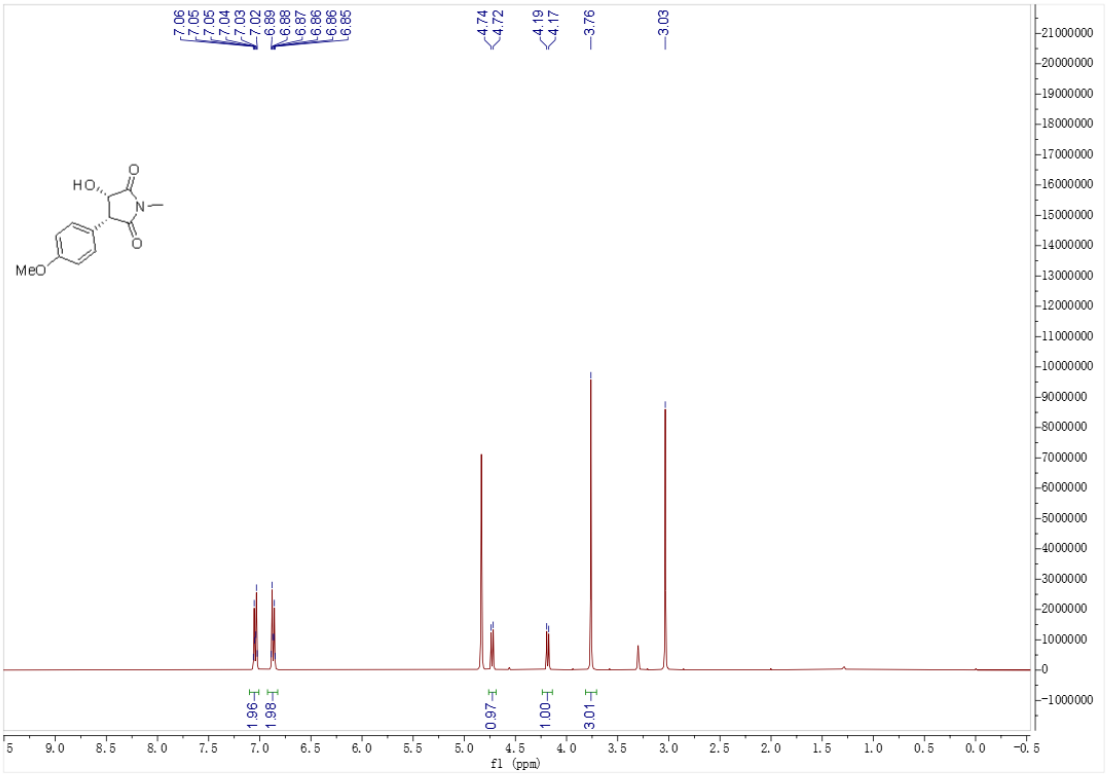

Supplementary Figure 125. ^{1}H NMR of 3m (400 MHz, Methanol-d_4).

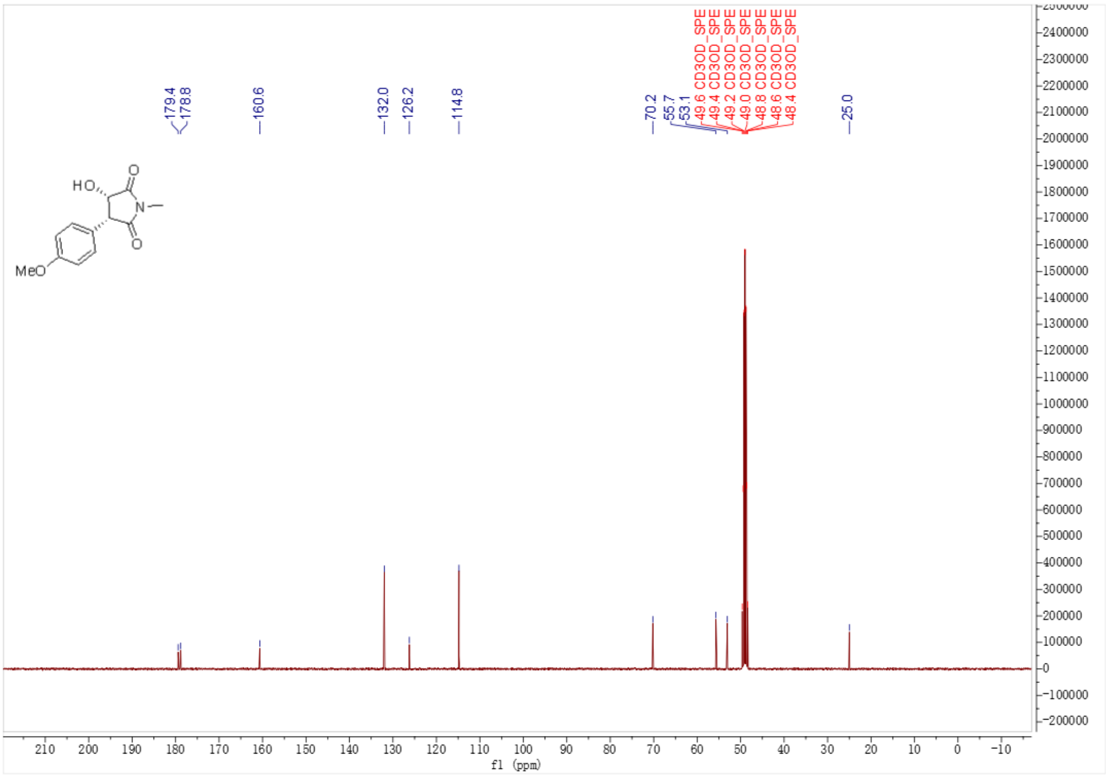

Supplementary Figure 126. ^{13}C NMR of 3m (101 MHz, Methanol-d_4).

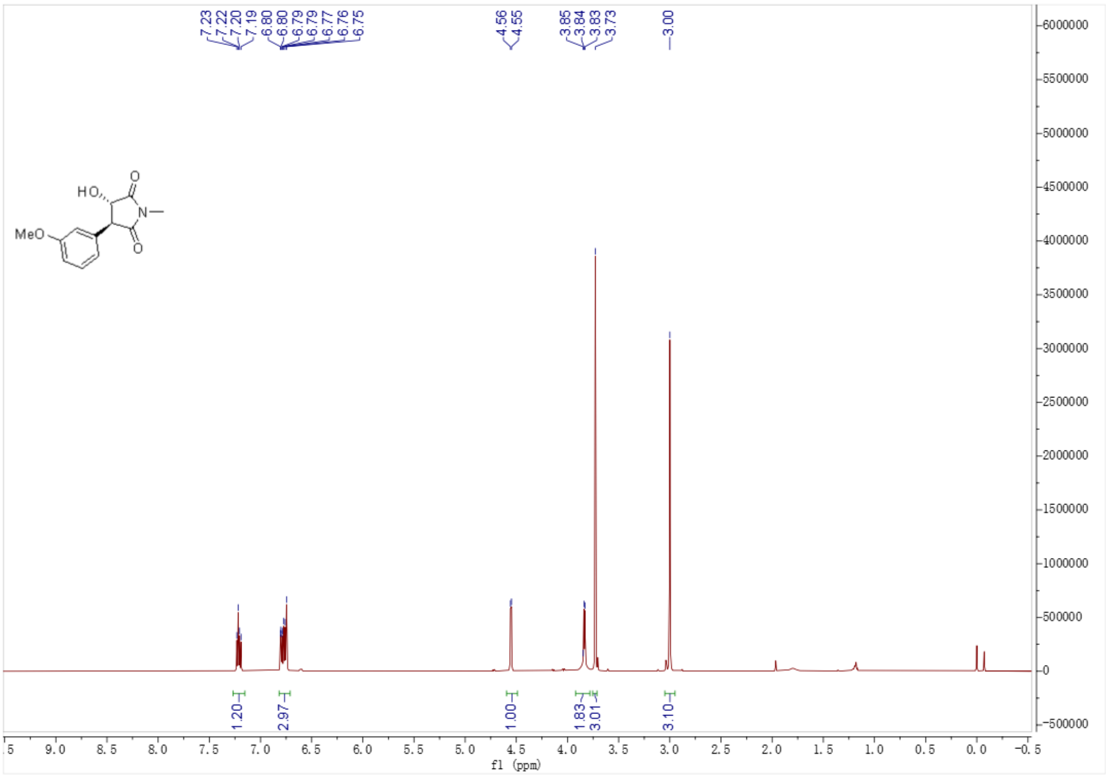

Supplementary Figure 127. ^{1}H NMR of 2n (600 MHz, Chloroform-*d*).

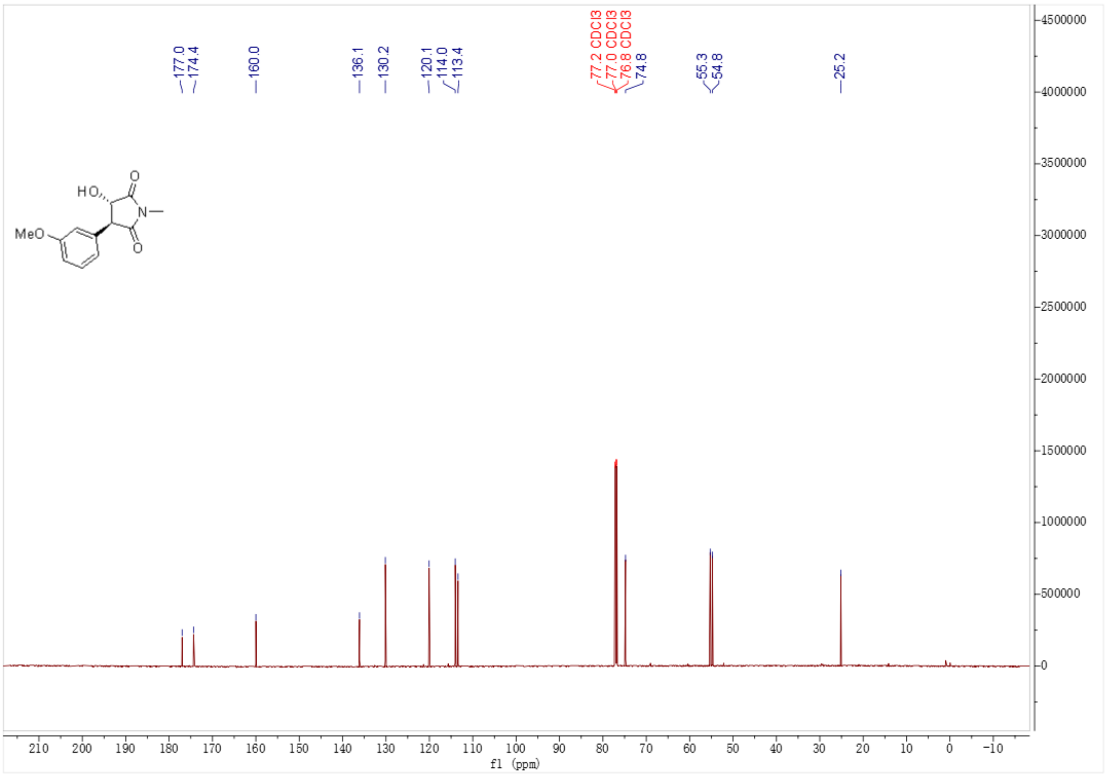

Supplementary Figure 128. ^{13}C NMR of 2n (151 MHz, Chloroform-*d*).

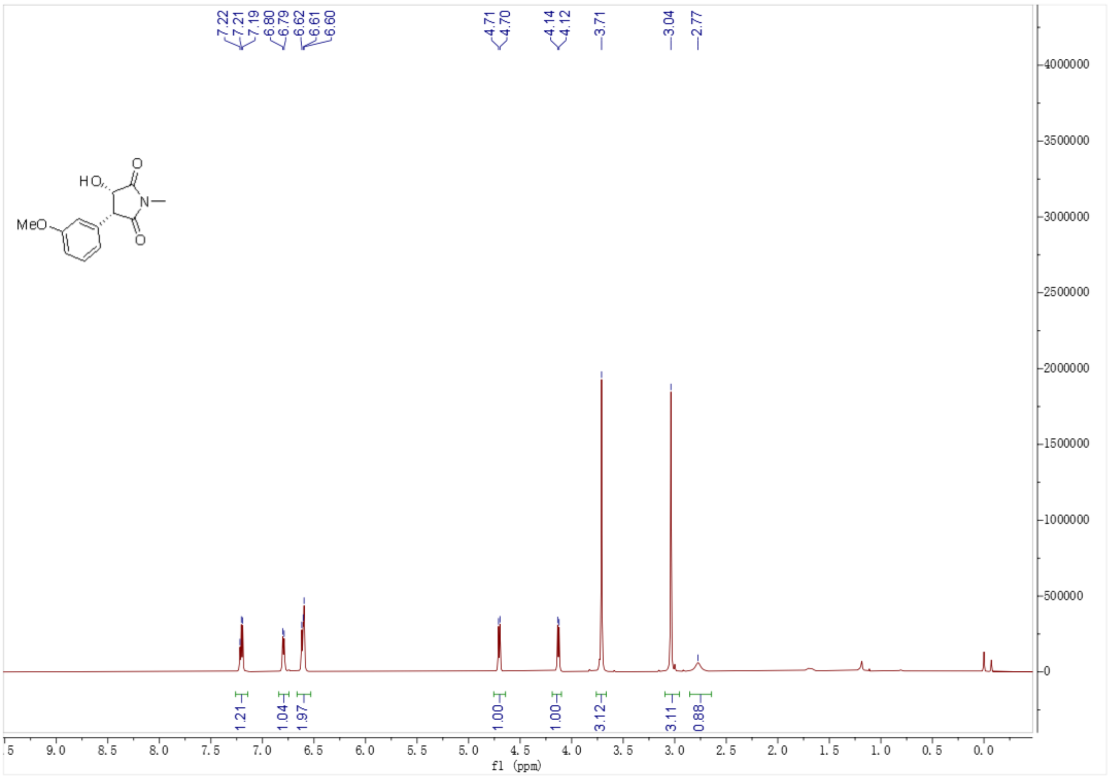

Supplementary Figure 129. ^{1}H NMR of 3n (600 MHz, Chloroform-*d*).

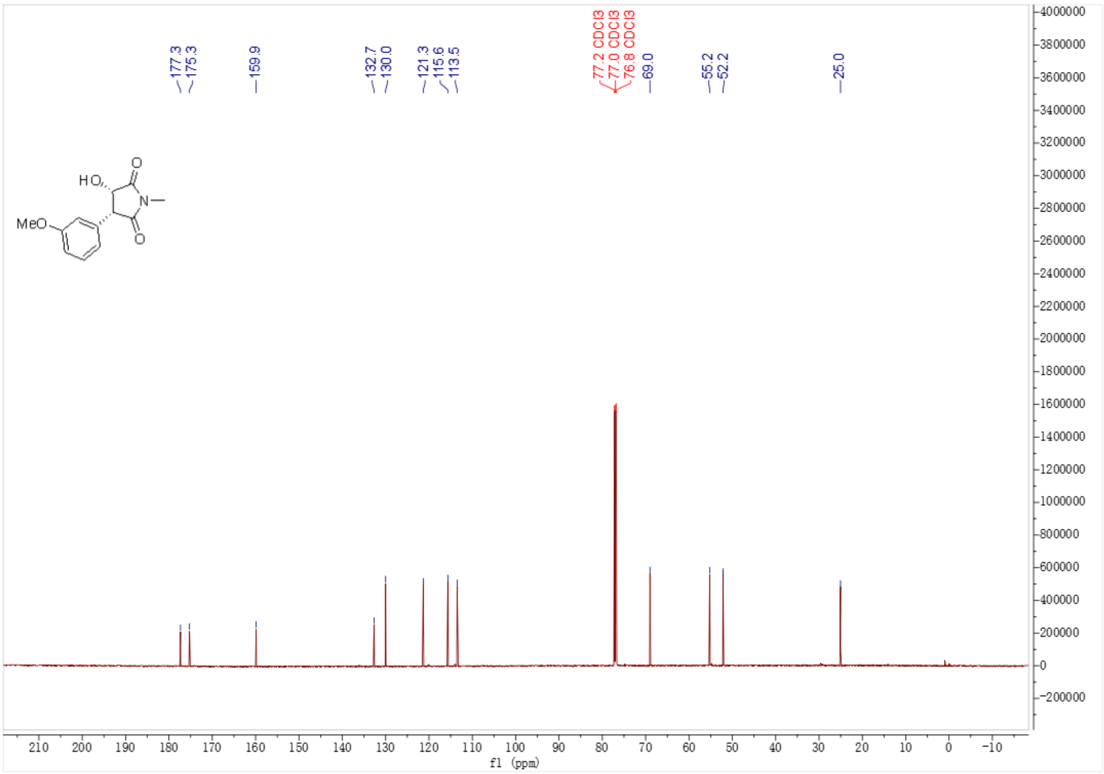

Supplementary Figure 130. ^{13}C NMR of 3n (151 MHz, Chloroform-*d*).

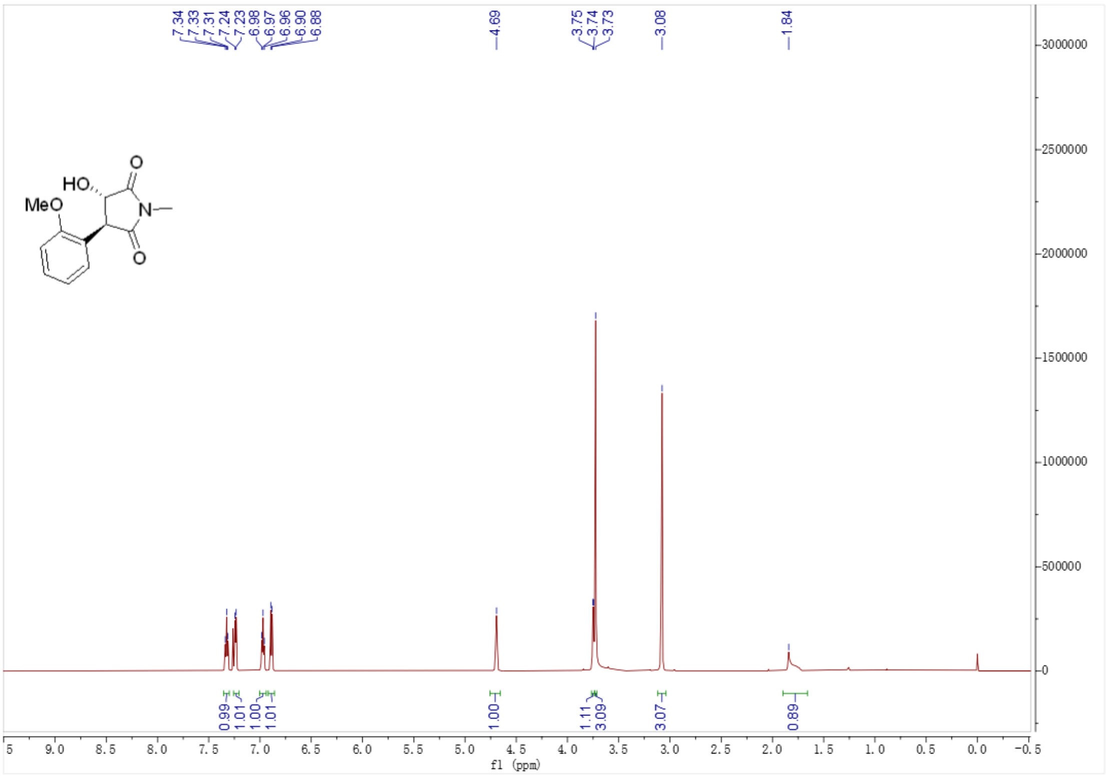

Supplementary Figure 131. **^{1}H NMR of 2o (600 MHz, Chloroform-*d*).**

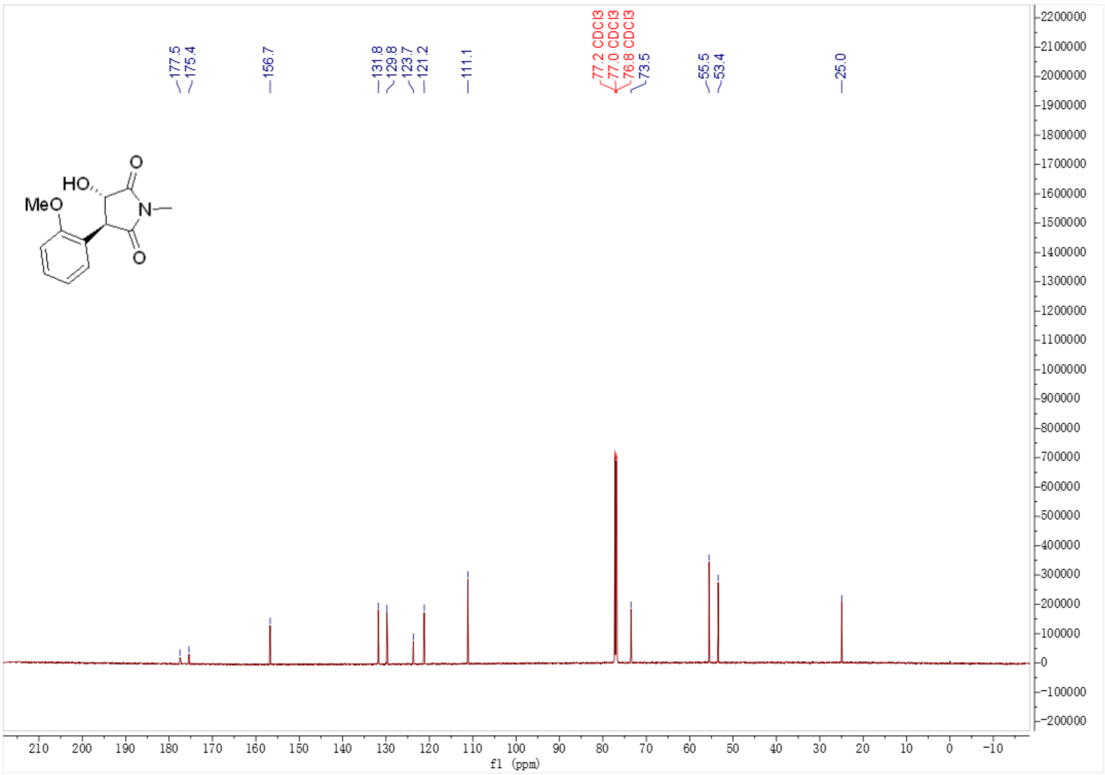

Supplementary Figure 132. **^{13}C NMR of 2o (151 MHz, Chloroform-*d*).**

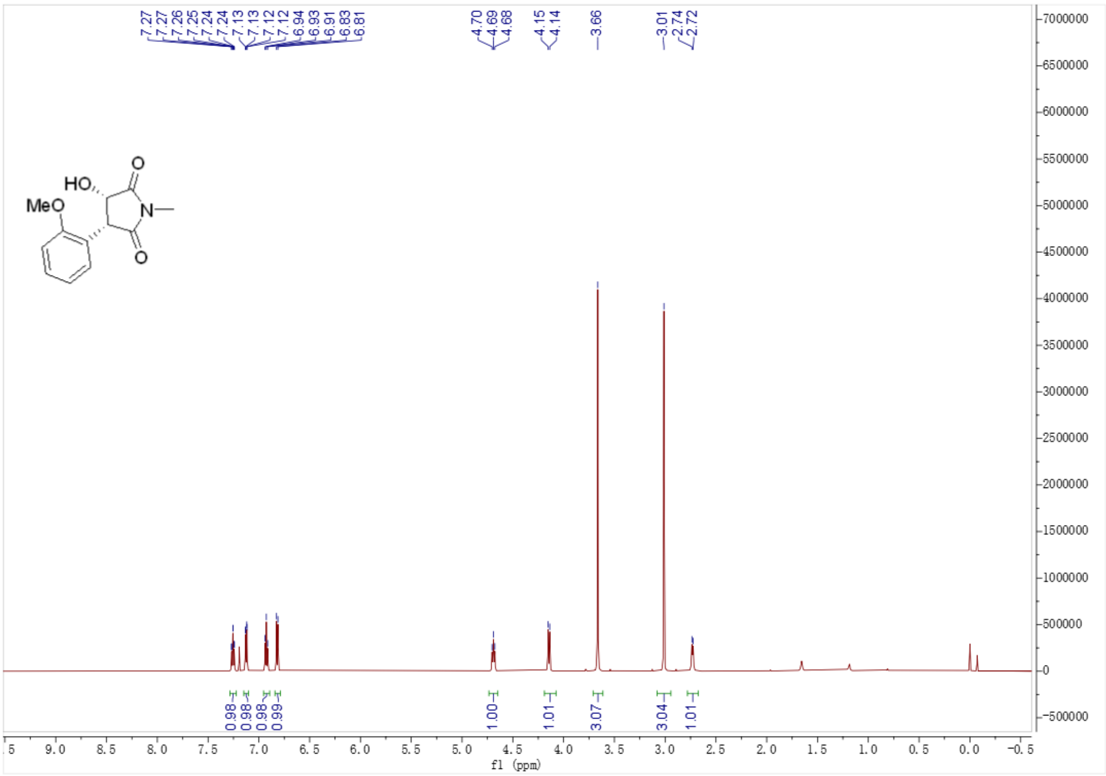

Supplementary Figure 133. ^{1}H NMR of 3o (600 MHz, Chloroform-*d*).

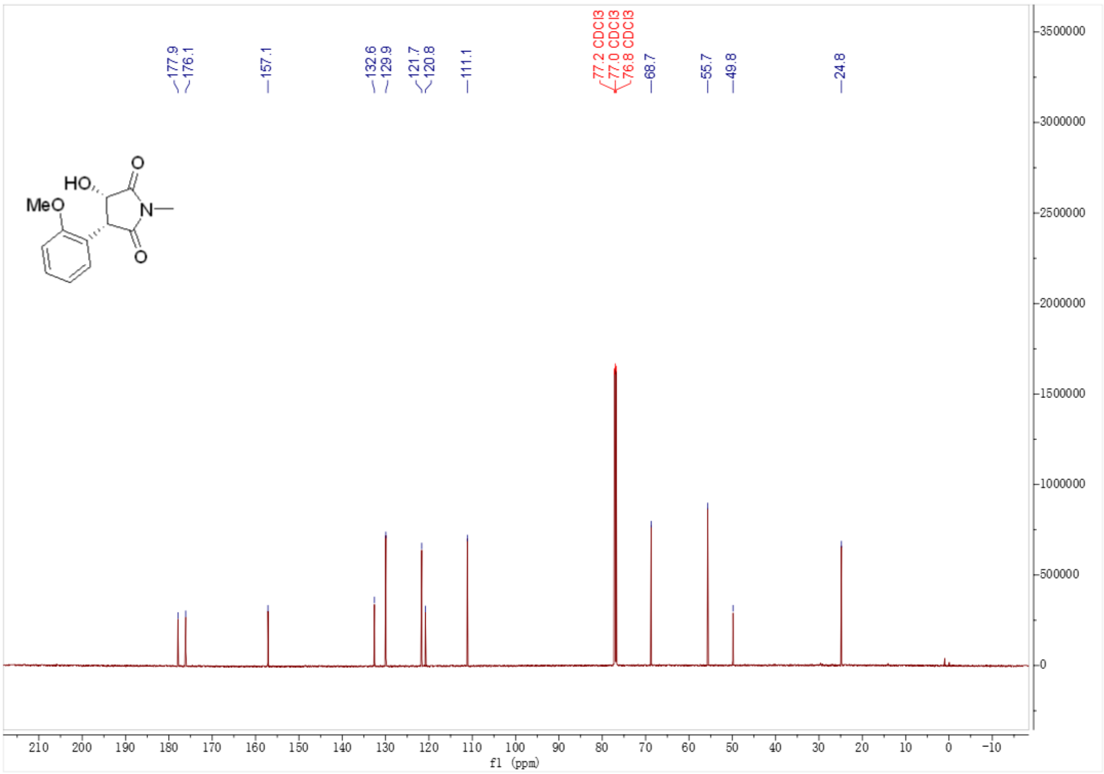

Supplementary Figure 134. ^{13}C NMR of 3o (151 MHz, Chloroform-*d*).

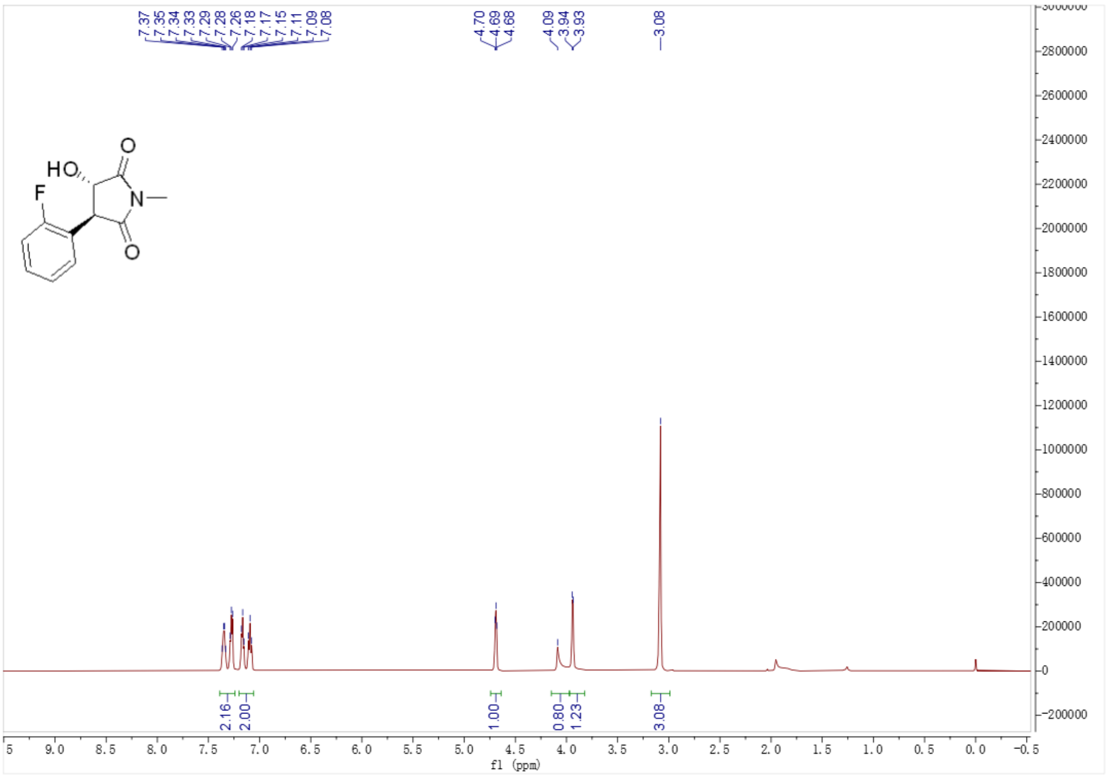

Supplementary Figure 135. **^{1}H NMR of 2p (600 MHz, Chloroform-*d*).**

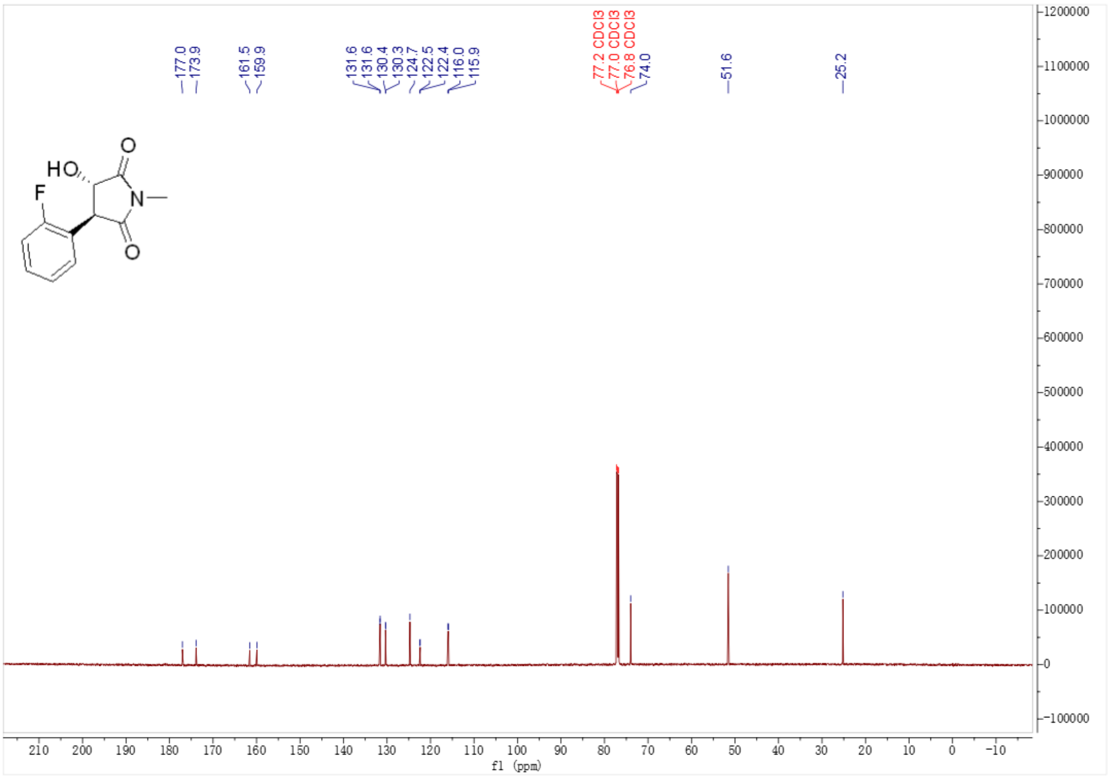

Supplementary Figure 136. **^{13}C NMR of 2p (151 MHz, Chloroform-*d*).**

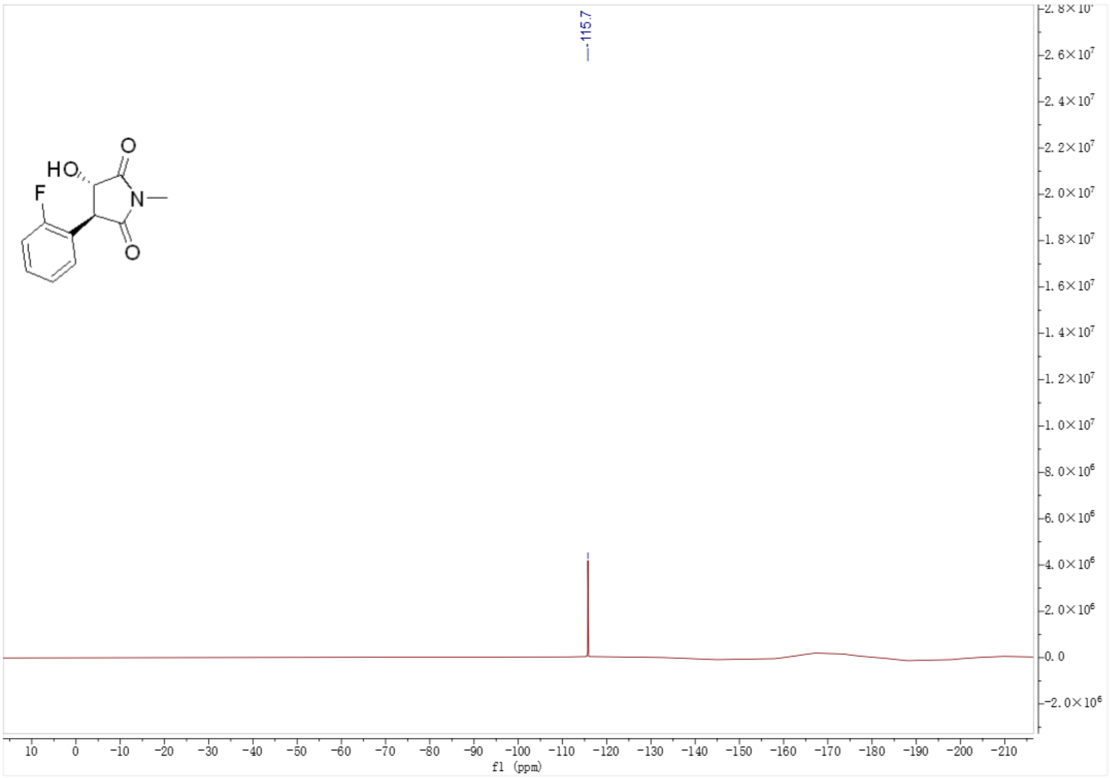

Supplementary Figure 137. ^{19}F NMR of 2p (565 MHz, Chloroform-*d*).

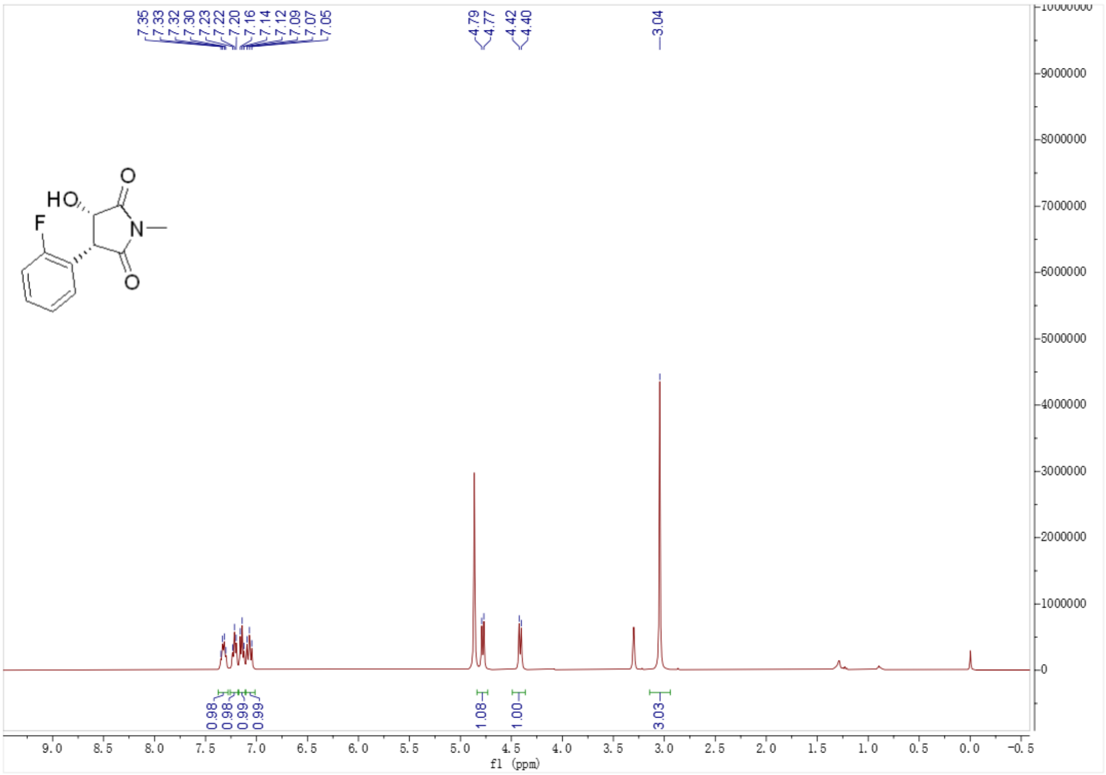

Supplementary Figure 138. ^{1}H NMR of 3p (400 MHz, Methanol-*d*$_4$).

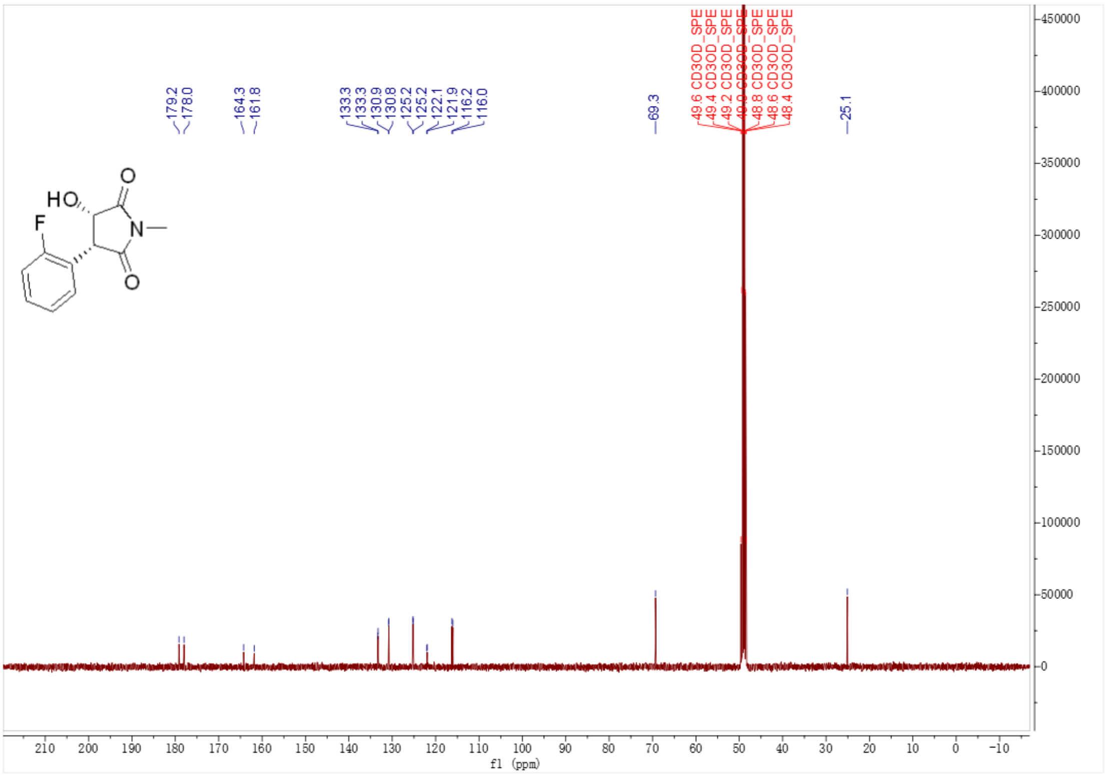

Supplementary Figure 139. ^{13}C NMR of 3p (101 MHz, Methanol-d_4).

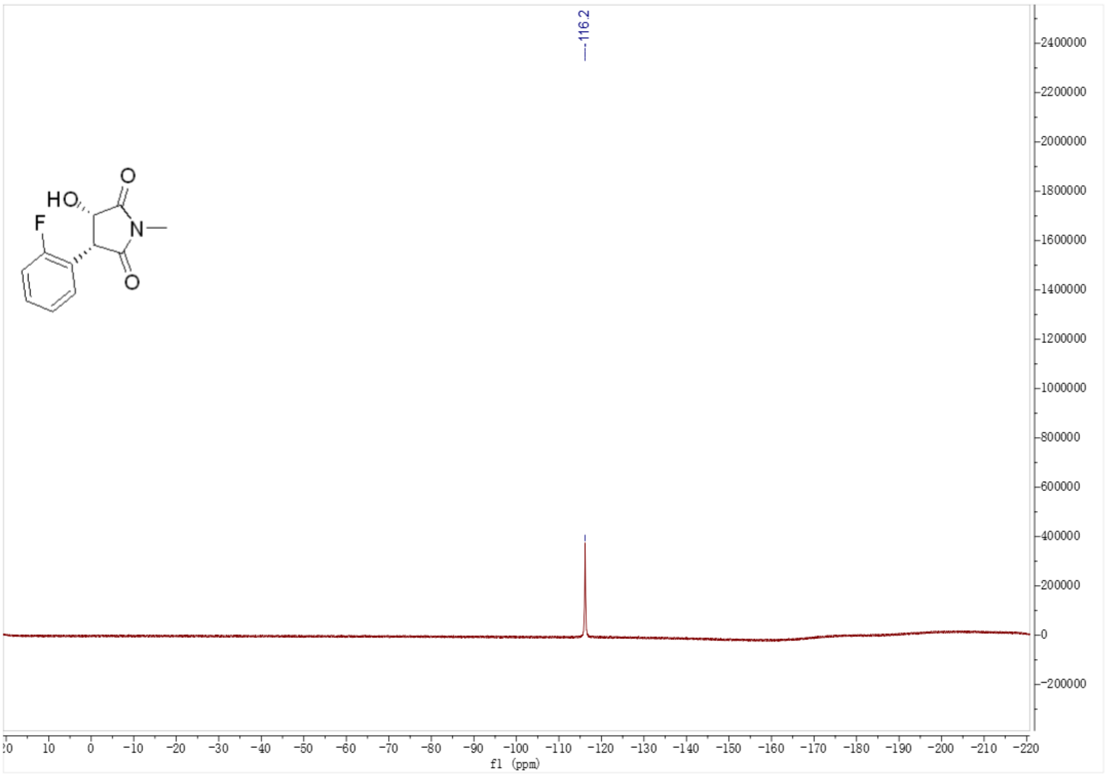

Supplementary Figure 140. ^{19}F NMR of 3p (376 MHz, Methanol-d_4).

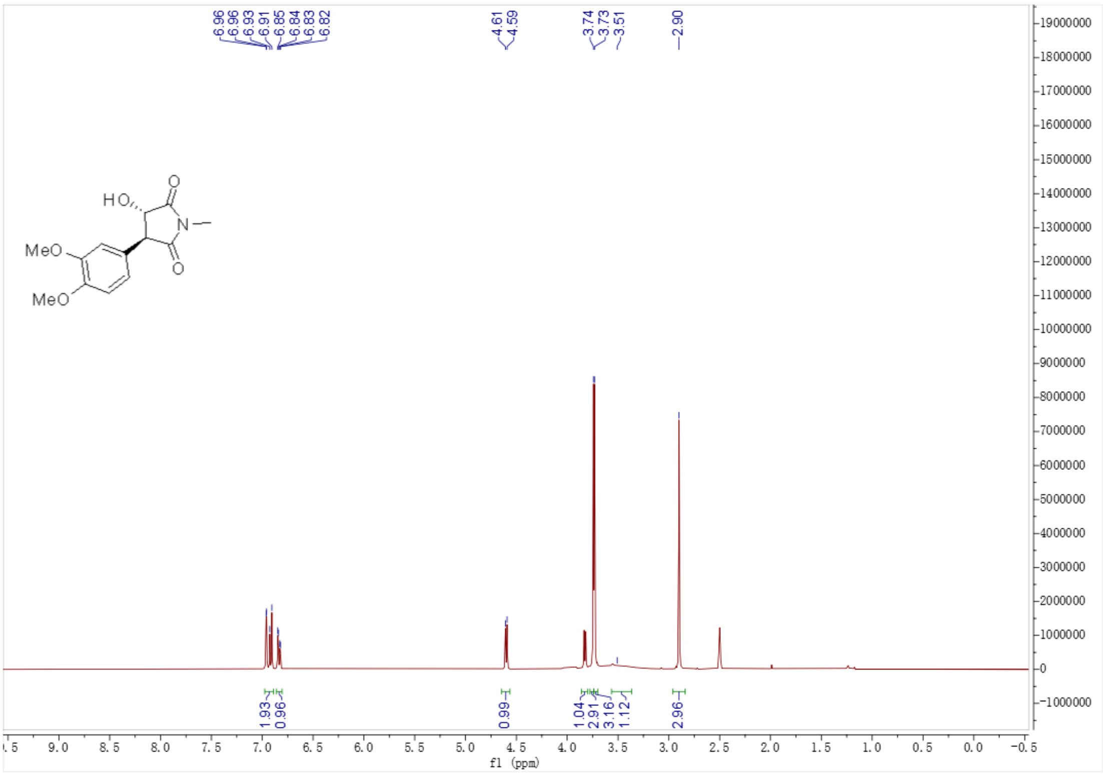

Supplementary Figure 141. ^{1}**H NMR of 2q (400 MHz, DMSO-*d*$_6$).**

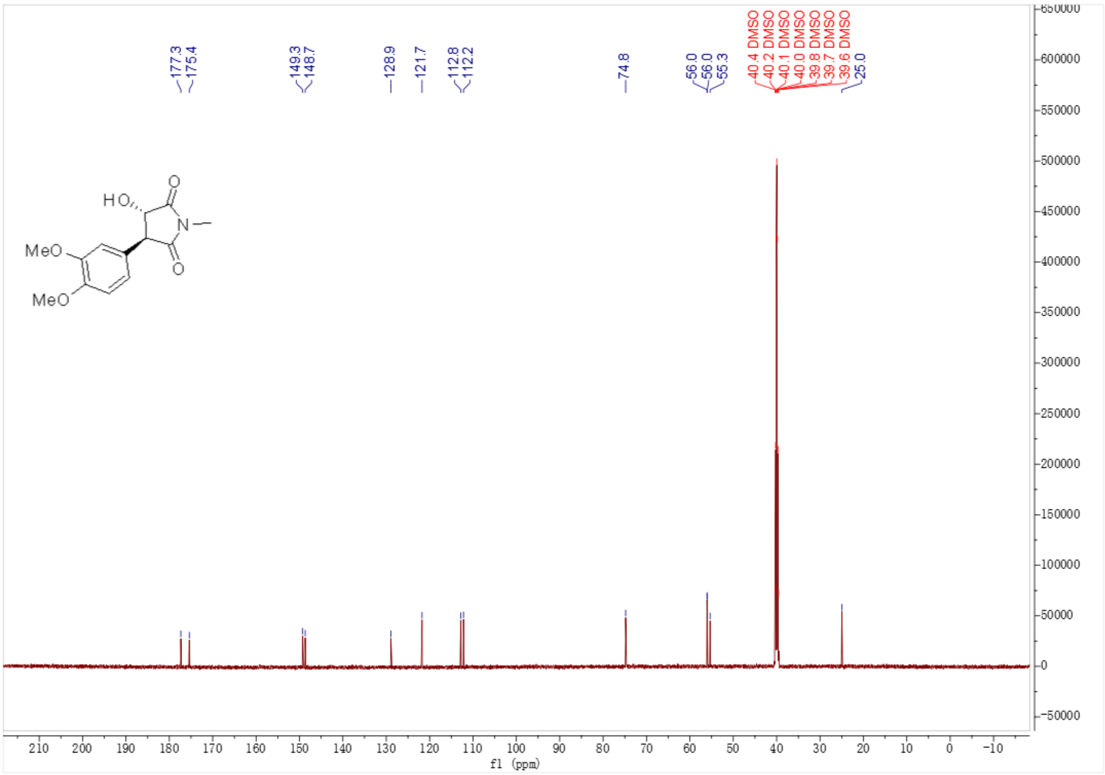

Supplementary Figure 142. ^{13}**C NMR of 2q (151 MHz, DMSO-*d*$_6$)**

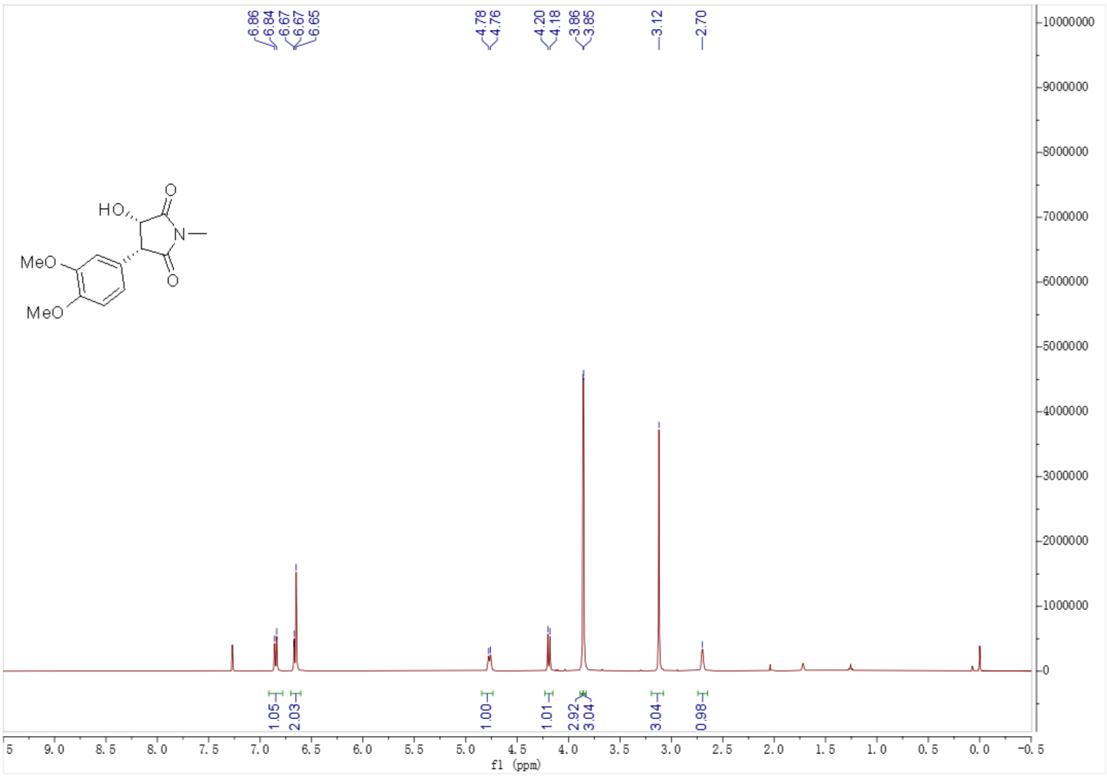

Supplementary Figure 143. ^{1}**H NMR of 3q (400 MHz, Chloroform-*d*).**

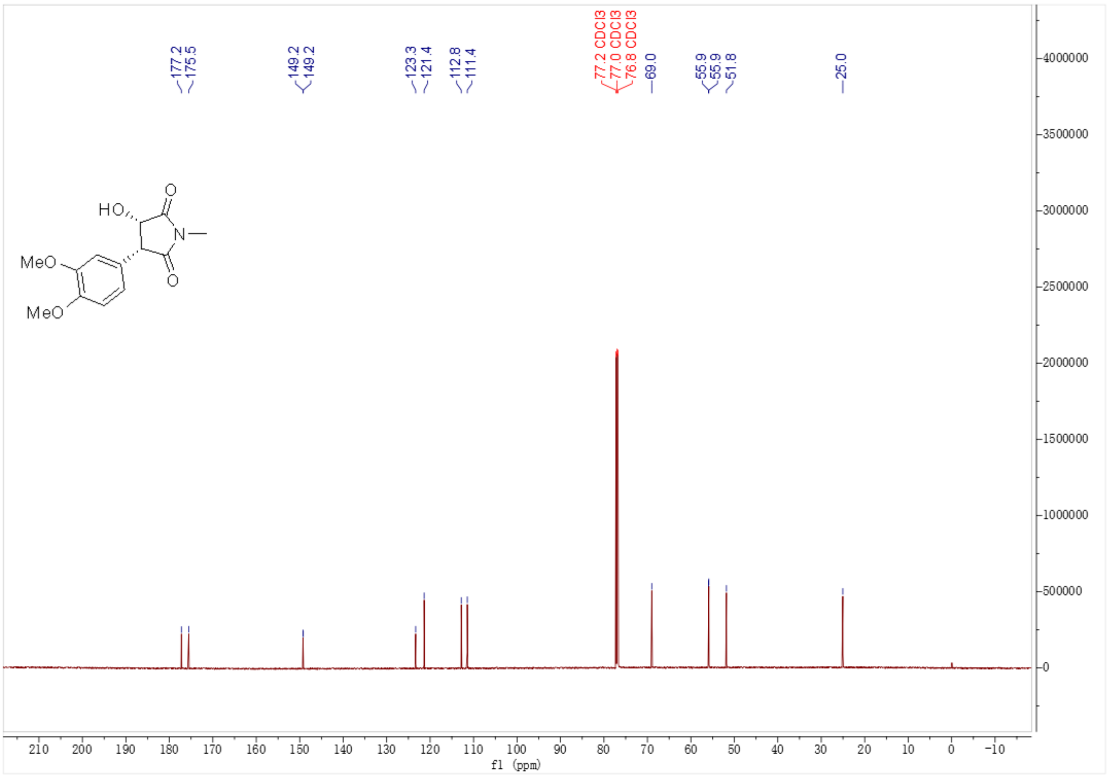

Supplementary Figure 144. ^{13}**C NMR of 3q (151 MHz, Chloroform-*d*).**

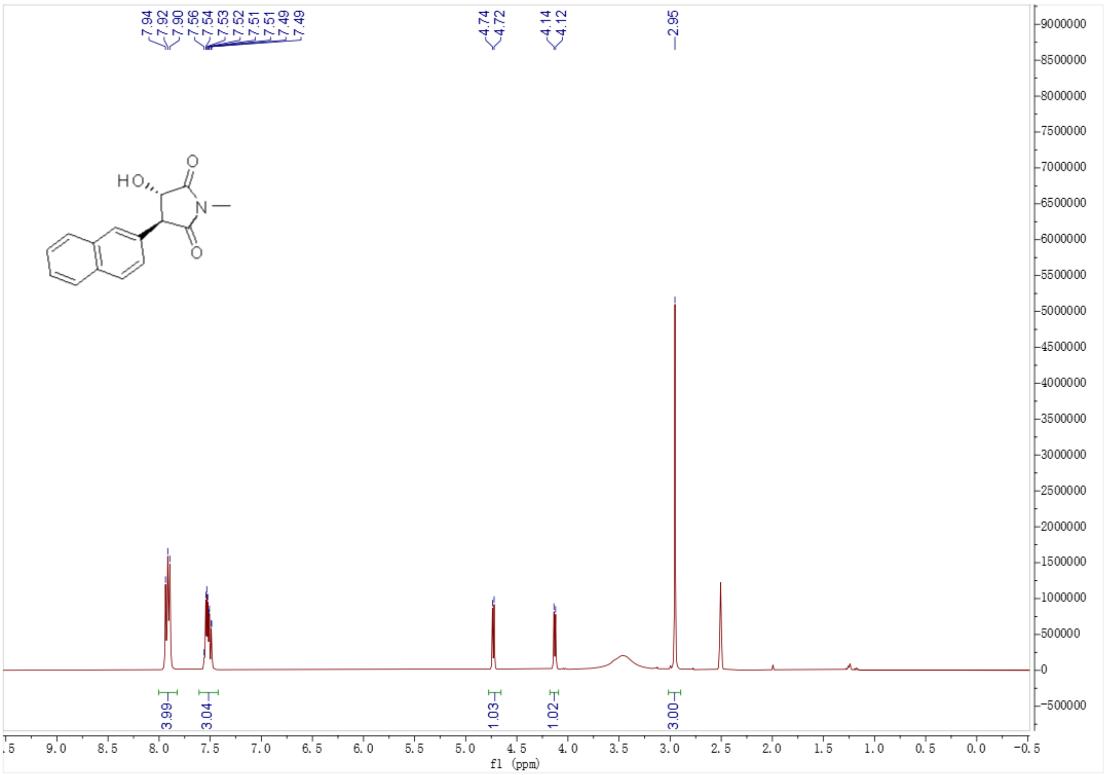

Supplementary Figure 145. ^{1}H NMR of 2r (400 MHz, DMSO-d_6).

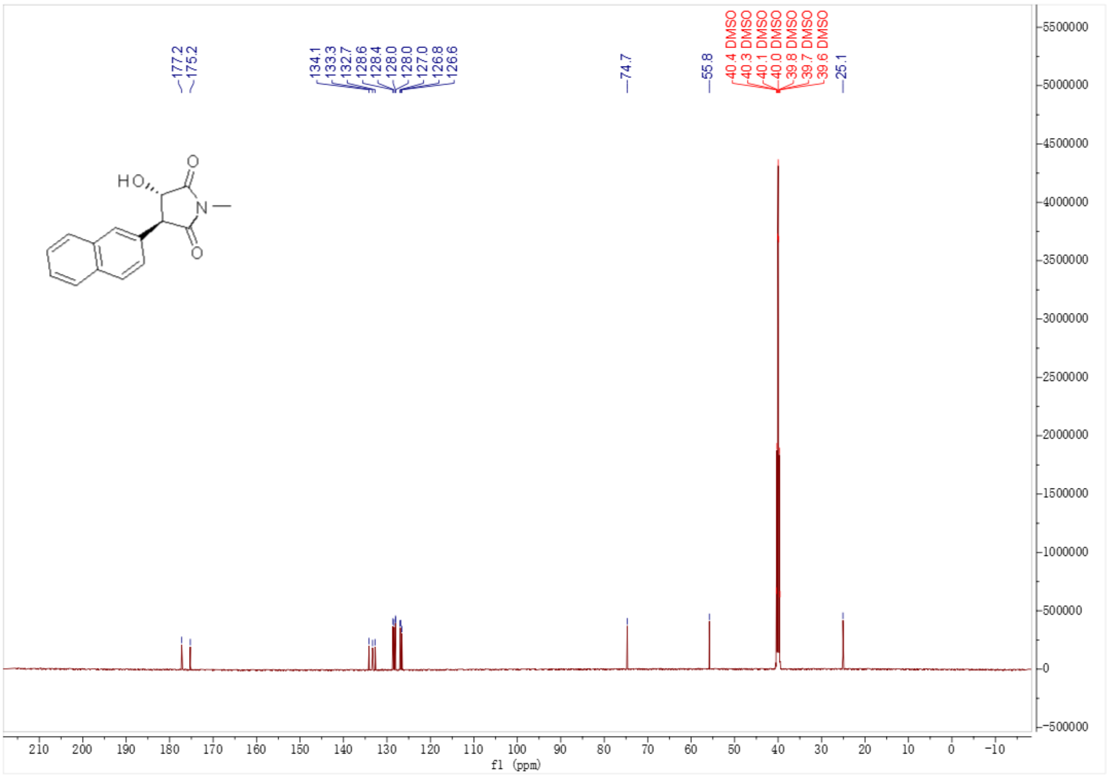

Supplementary Figure 146. ^{13}C NMR of 2r (151 MHz, DMSO-d_6).

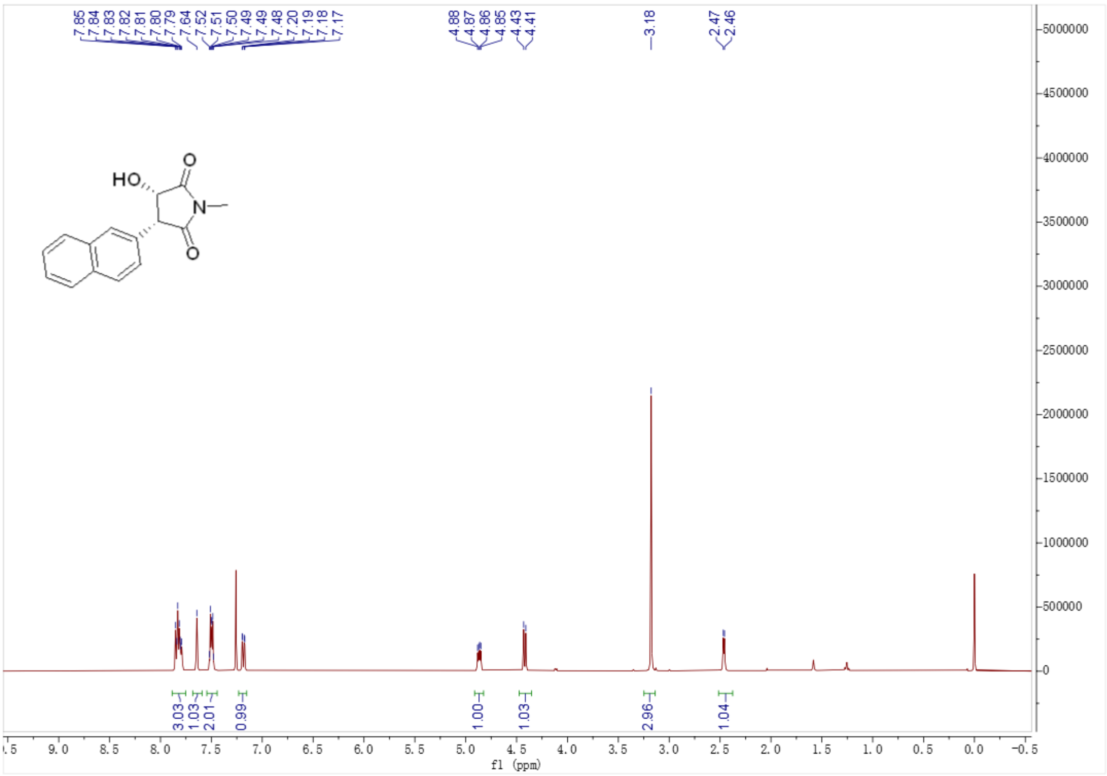

Supplementary Figure 147. ^{1}H NMR of 3r (400 MHz, Chloroform-*d*).

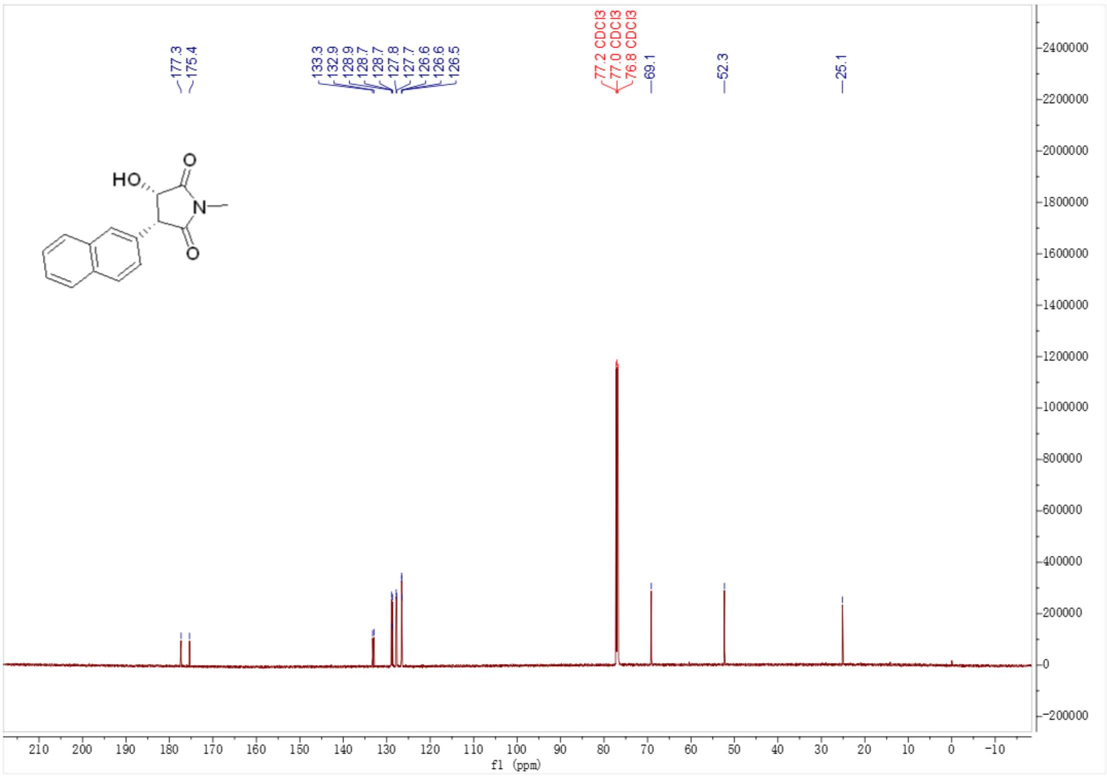

Supplementary Figure 148. ^{13}C NMR of 3r (151 MHz, Chloroform-*d*).

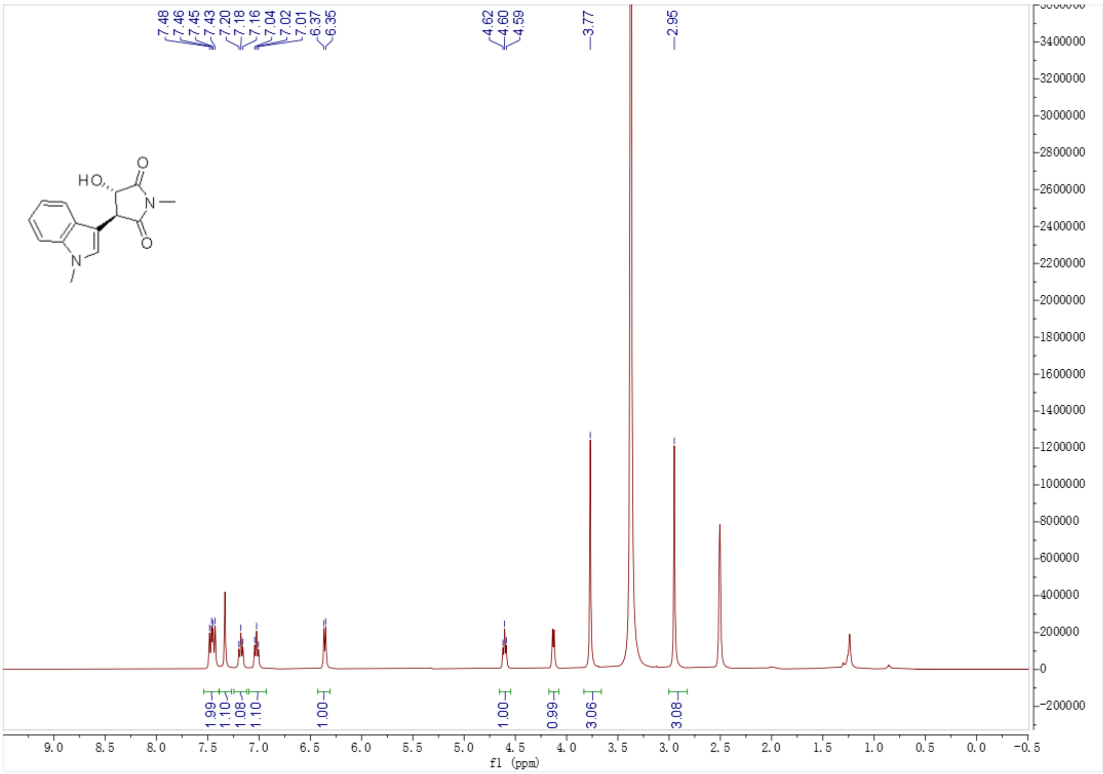

Supplementary Figure 149. ^{1}H NMR of 2s (400 MHz, DMSO-d_6).

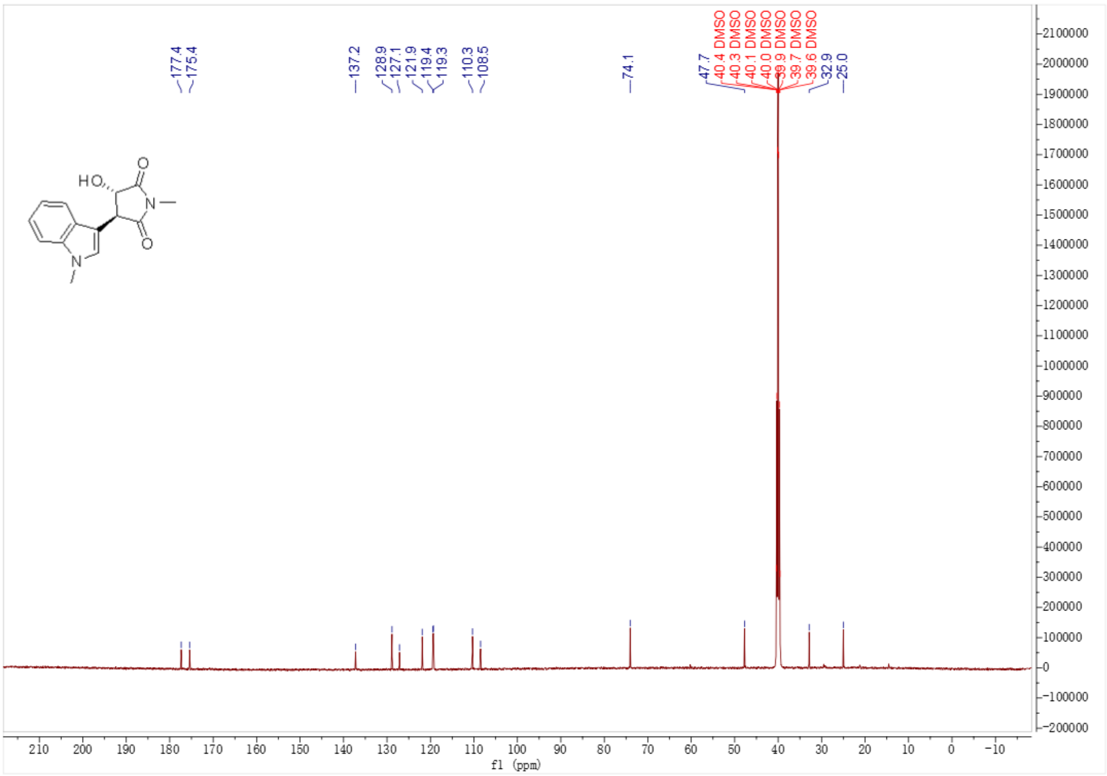

Supplementary Figure 150. ^{13}C NMR of 2s (151 MHz, DMSO-d_6).

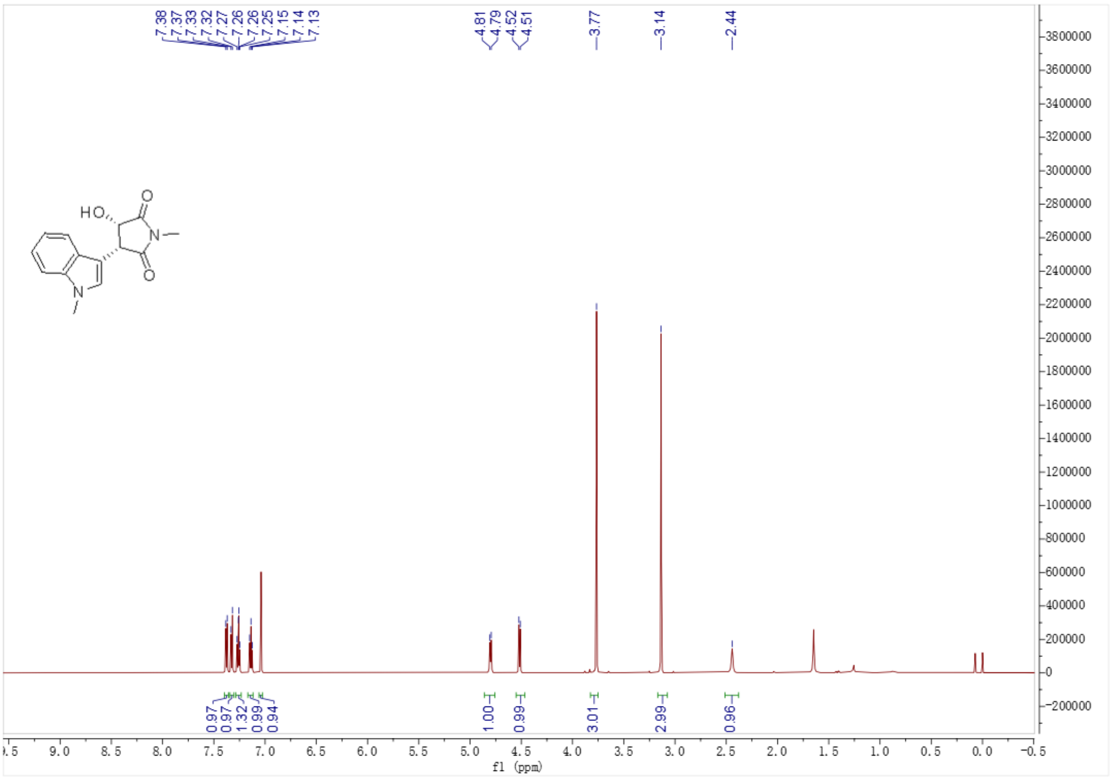

Supplementary Figure 151. ^{1}H NMR of 3s (600 MHz, Chloroform-*d*).

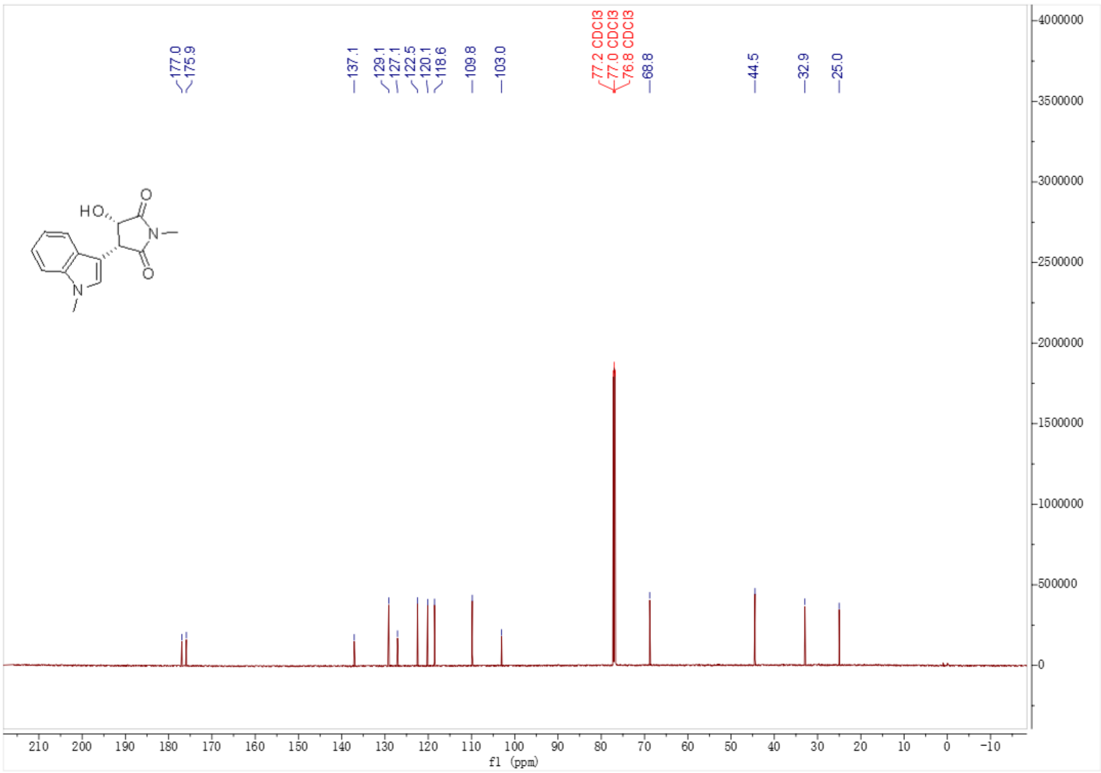

Supplementary Figure 152. ^{13}C NMR of 3s (151 MHz, Chloroform-*d*).

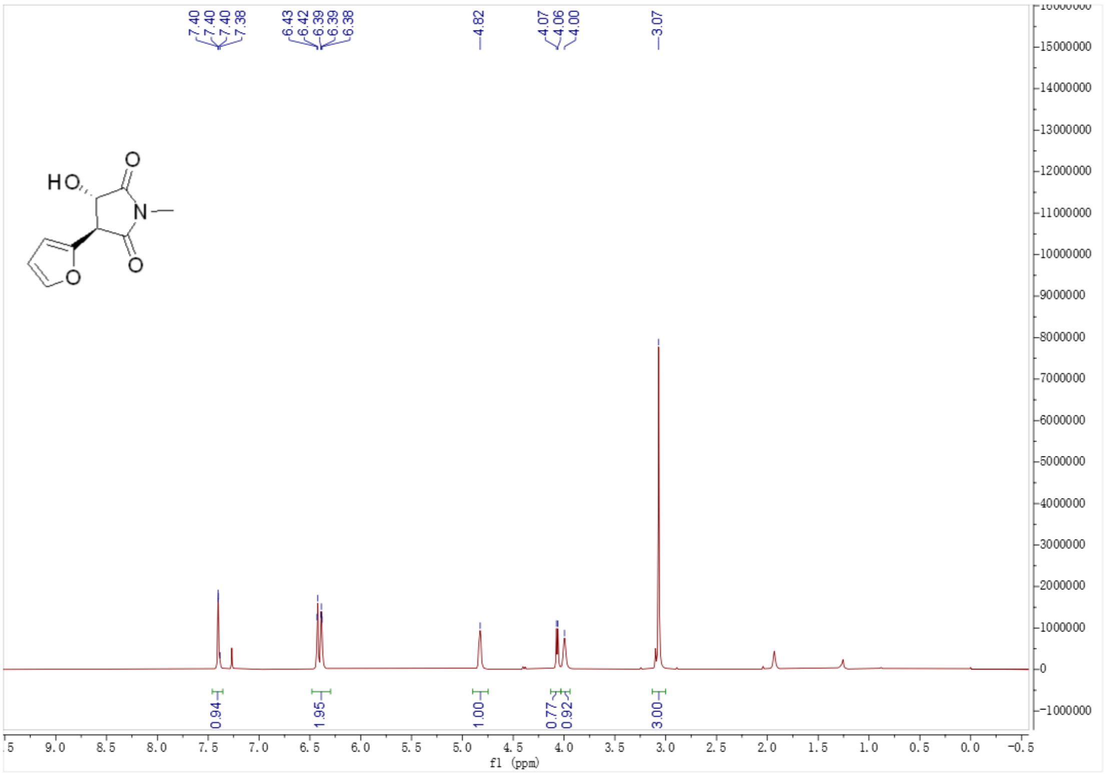

Supplementary Figure 153. **^{1}H NMR of 2t (400 MHz, Chloroform-*d*).**

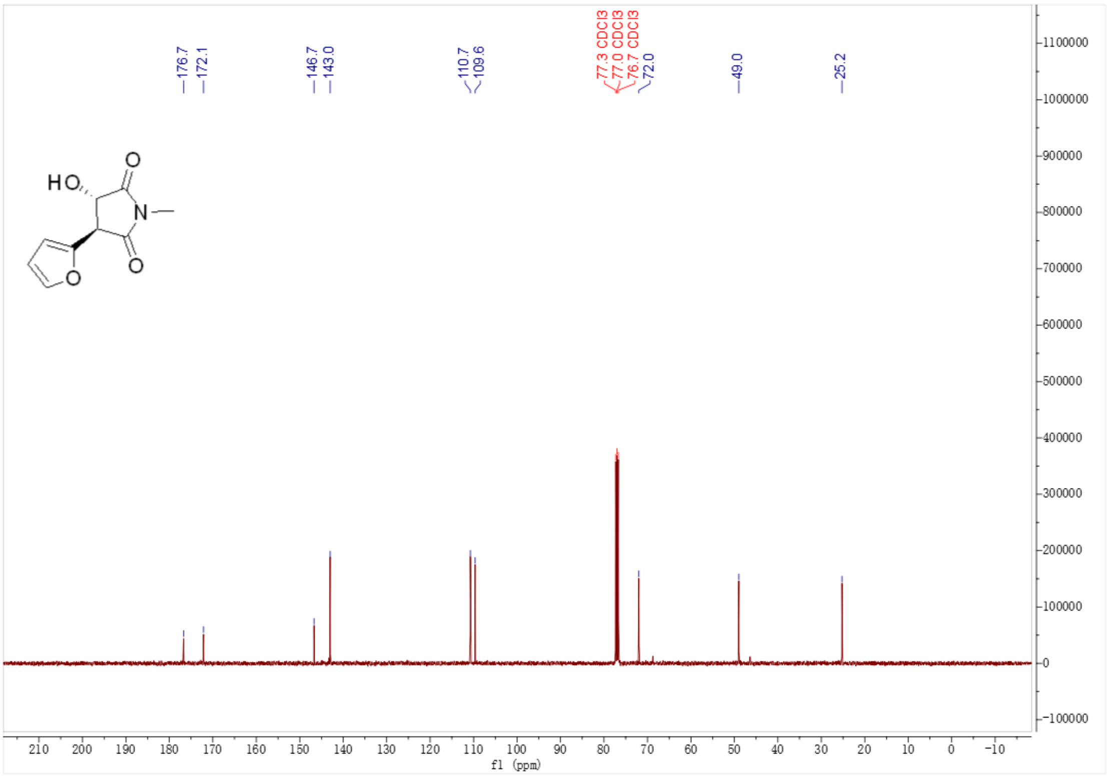

Supplementary Figure 154. **^{13}C NMR of 2t (101 MHz, Chloroform-*d*).**

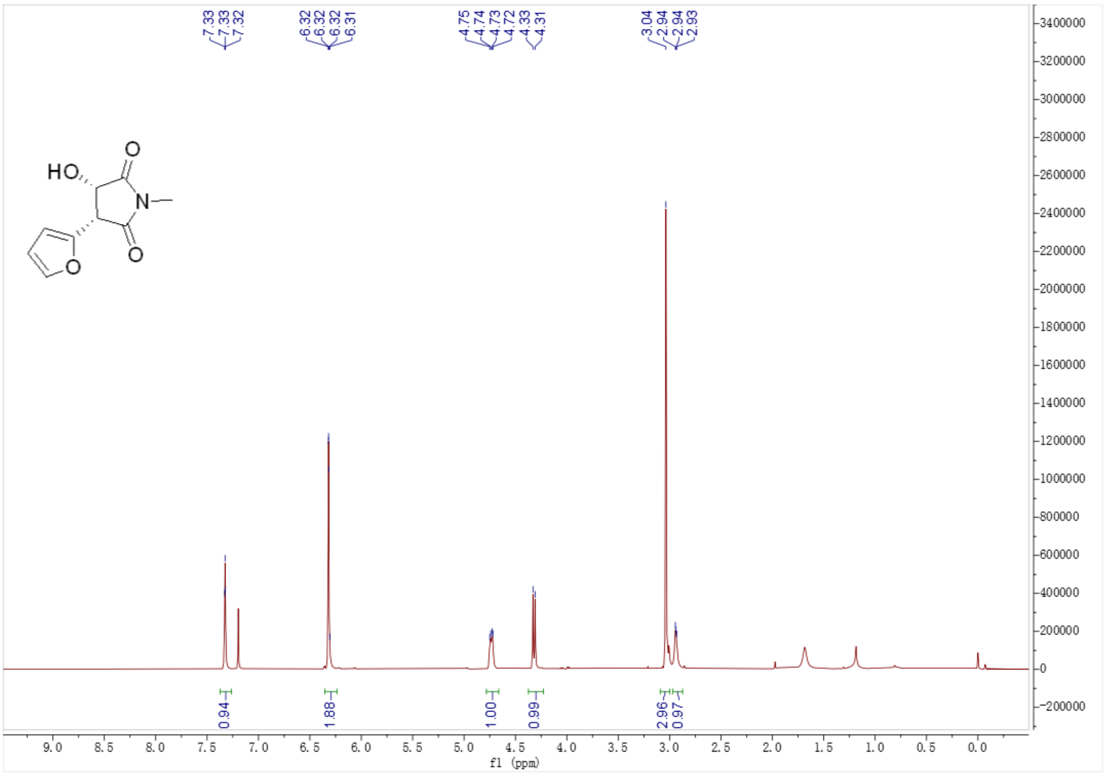

Supplementary Figure 155. **^{1}H NMR of 3t (400 MHz, Chloroform-*d*).**

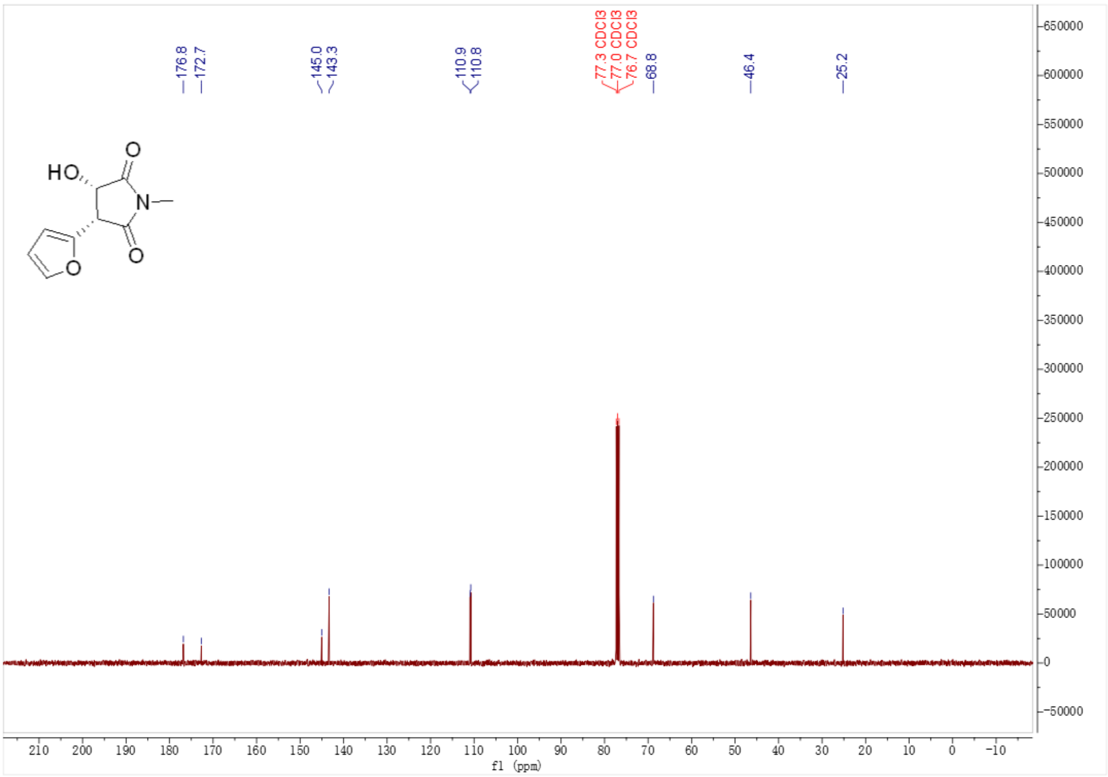

Supplementary Figure 156. **^{13}C NMR of 3t (101 MHz, Chloroform-*d*).**

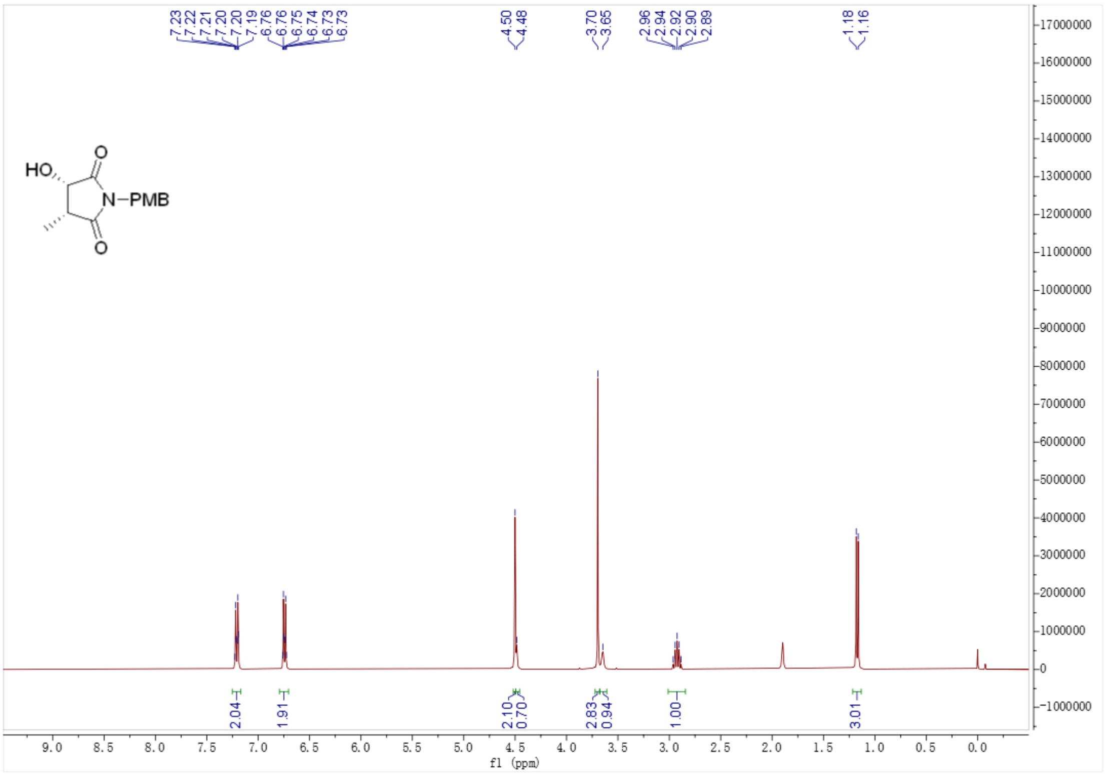

Supplementary Figure 157. [11]H NMR of 3u (400 MHz, Chloroform-*d*).

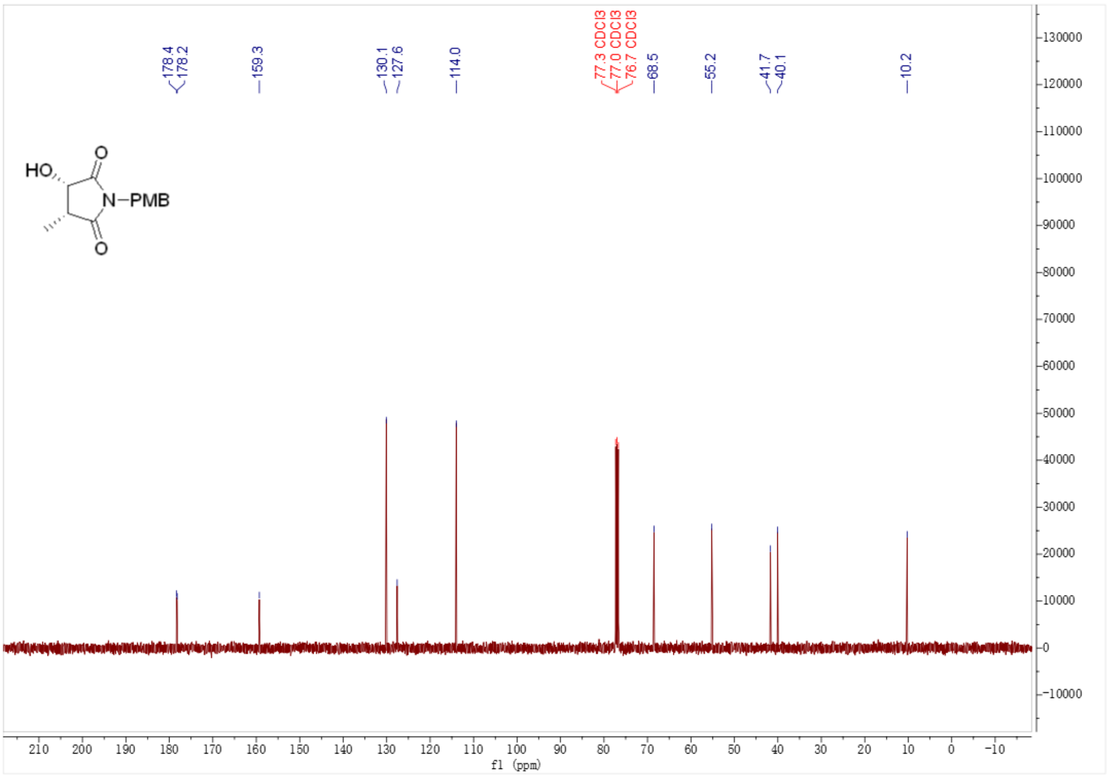

Supplementary Figure 158. ^{13}C NMR of 3u (101 MHz, Chloroform-*d*).

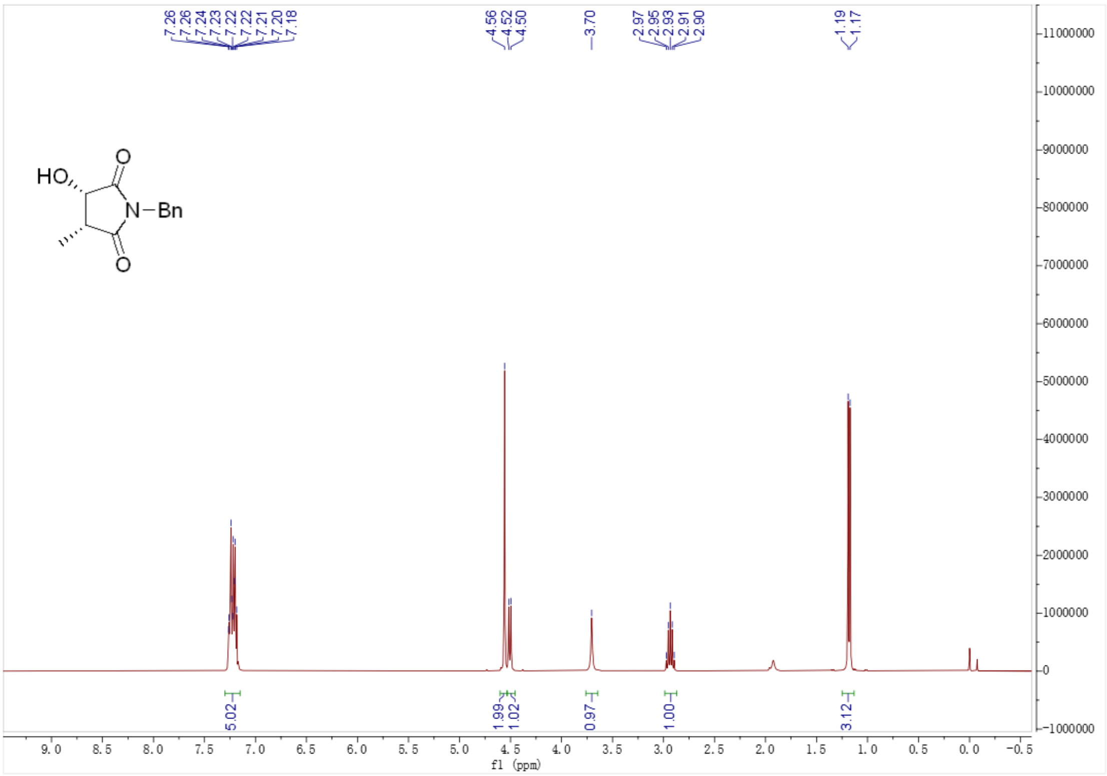

Supplementary Figure 159. ^{1}H NMR of 3v (400 MHz, Chloroform-*d*).

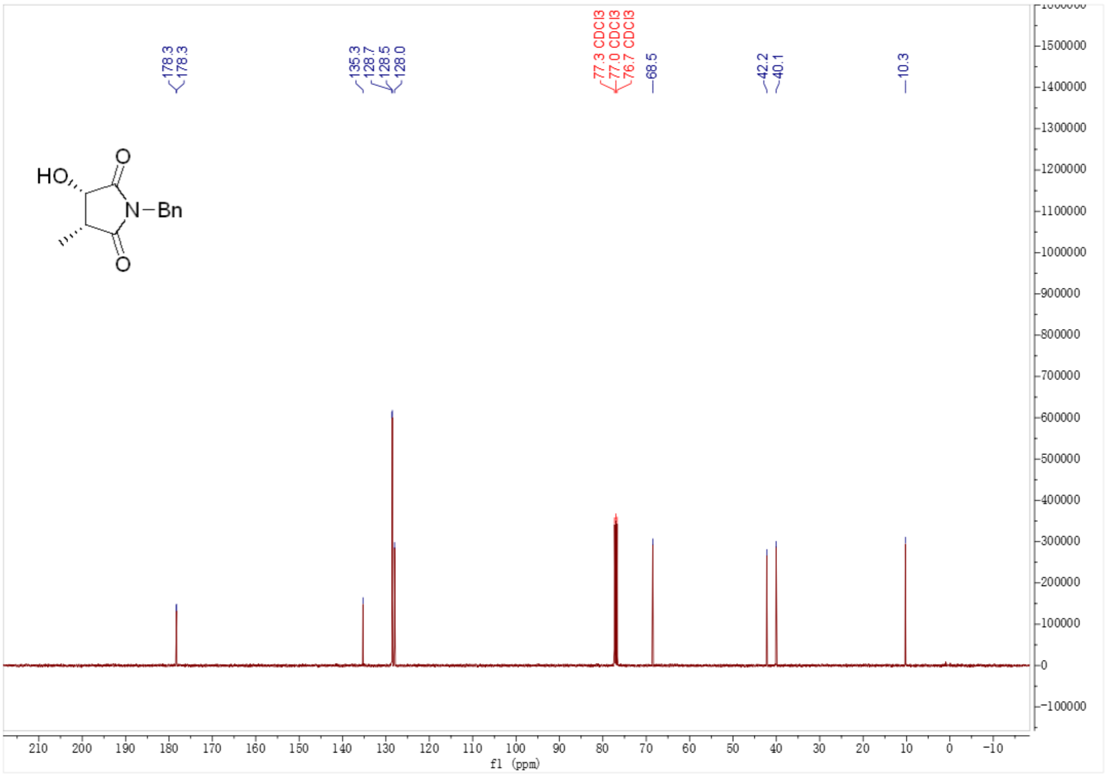

Supplementary Figure 160. ^{13}C NMR of 3v (101 MHz, Chloroform-*d*).

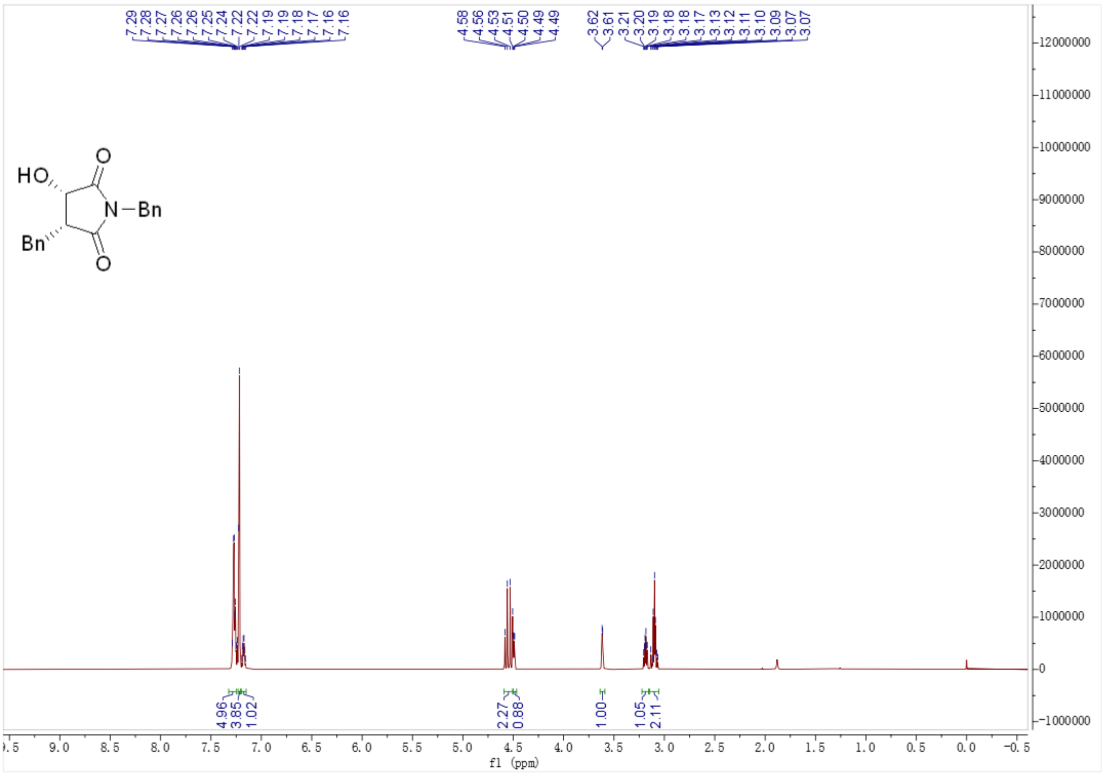

Supplementary Figure 161. ^{1}H NMR of 3w (600 MHz, Chloroform-*d*).

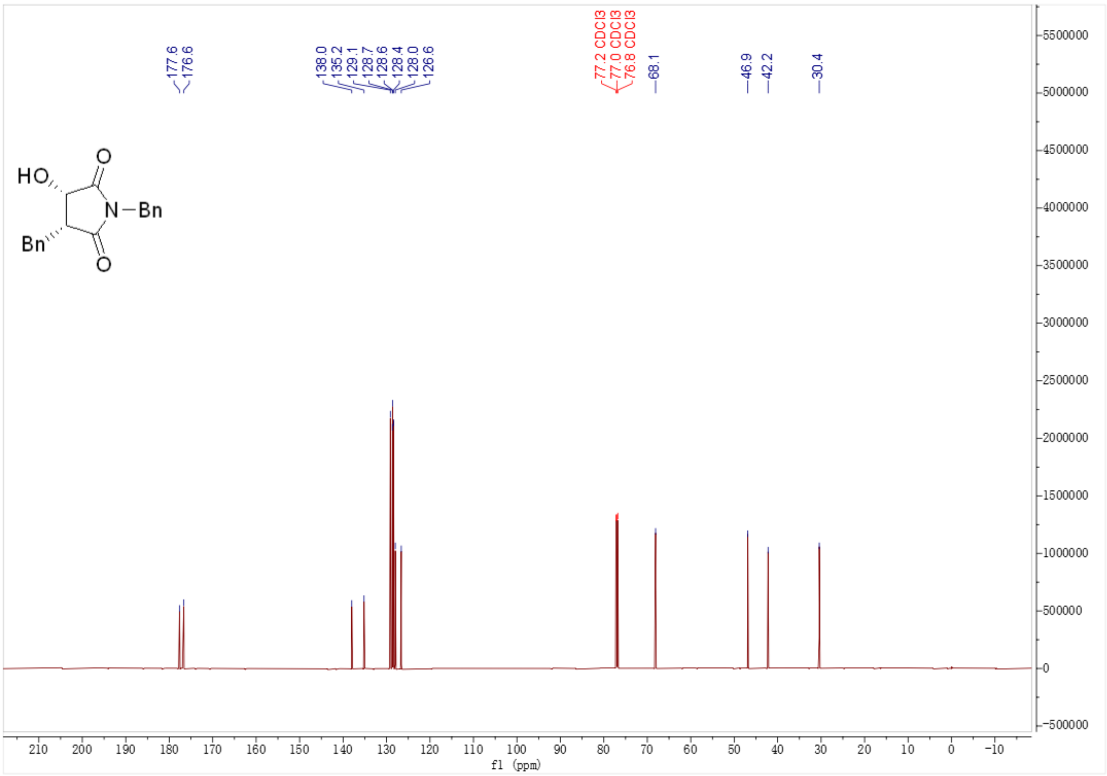

Supplementary Figure 162. ^{13}C NMR of 3w (151 MHz, Chloroform-*d*).

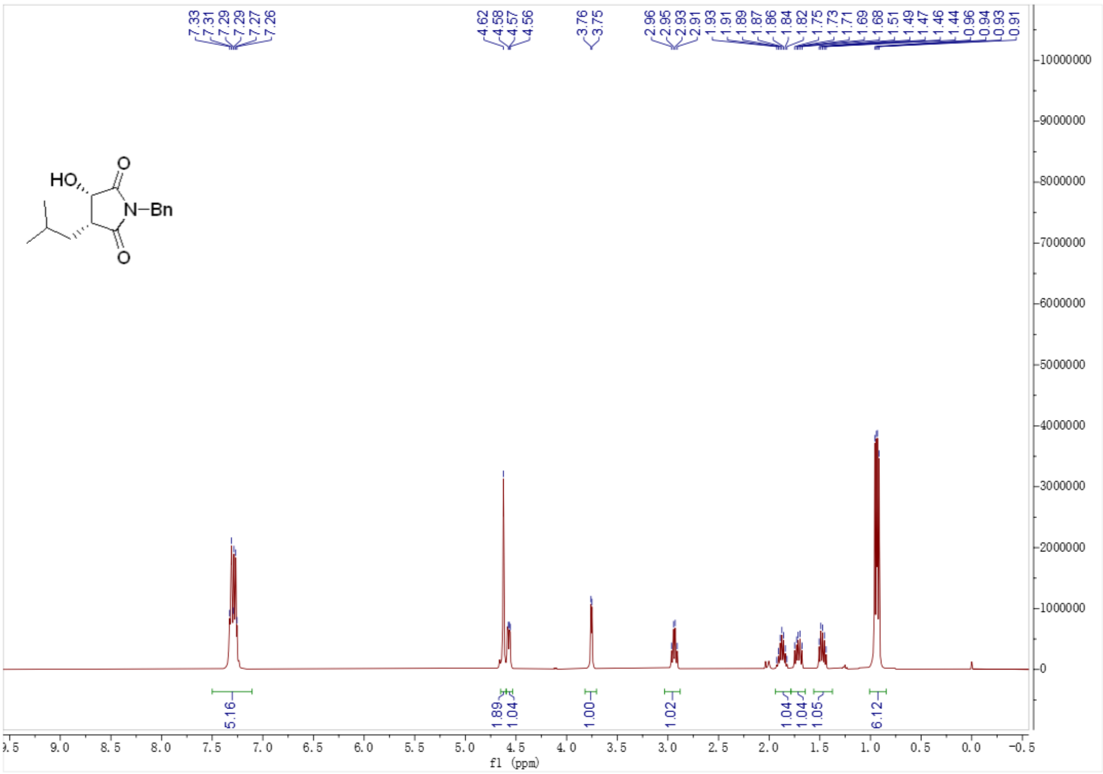

Supplementary Figure 163. ^{1}**H NMR of 3x (400 MHz, Chloroform-*d*).**

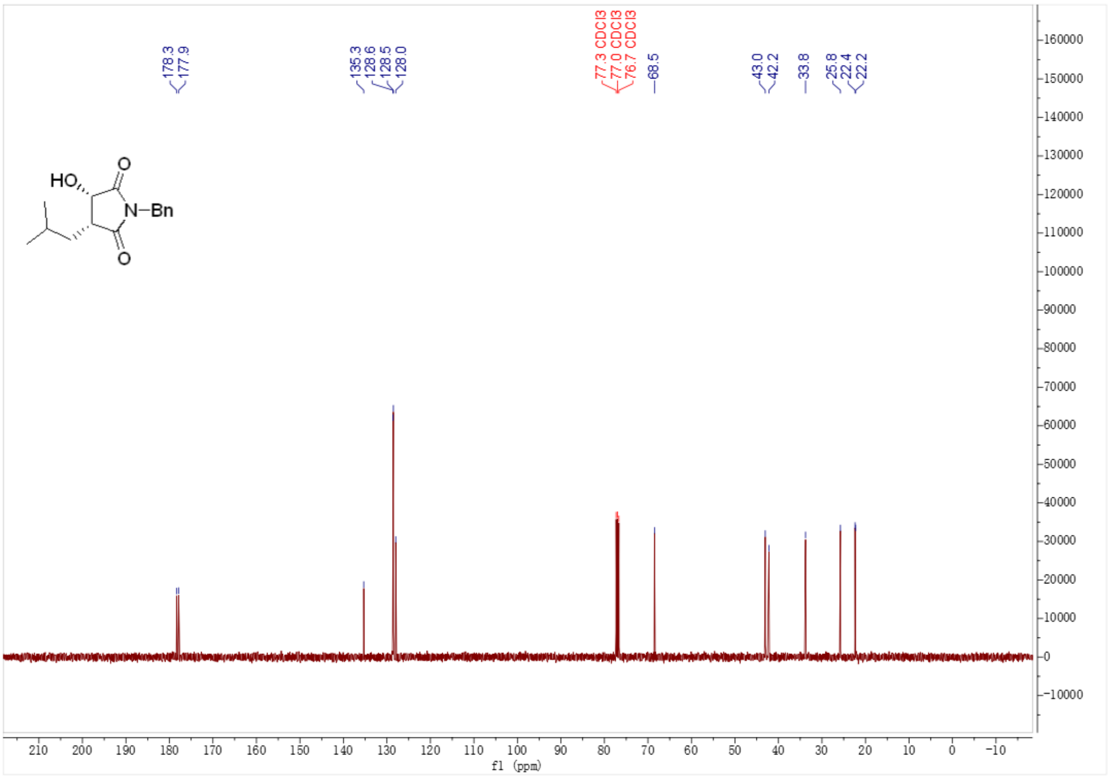

Supplementary Figure 164. ^{13}**C NMR of 3x (101 MHz, Chloroform-*d*).**

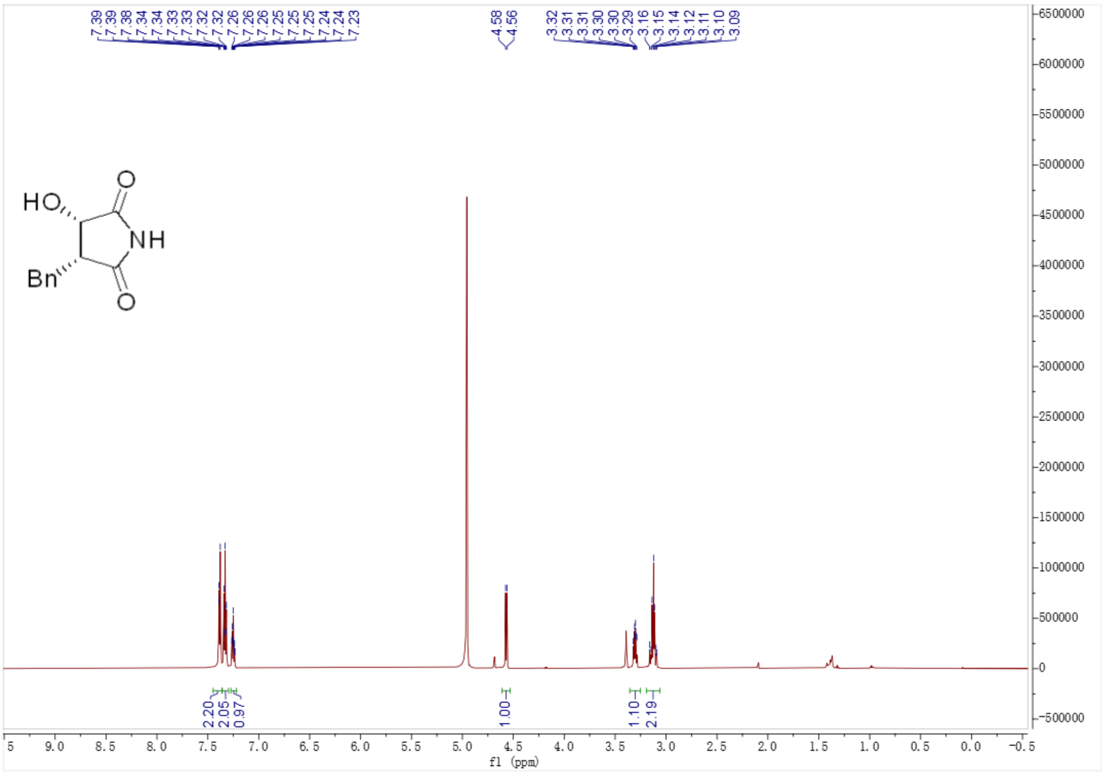

Supplementary Figure 165. ^{1}H NMR of 3y (600 MHz, Methanol-d_4).

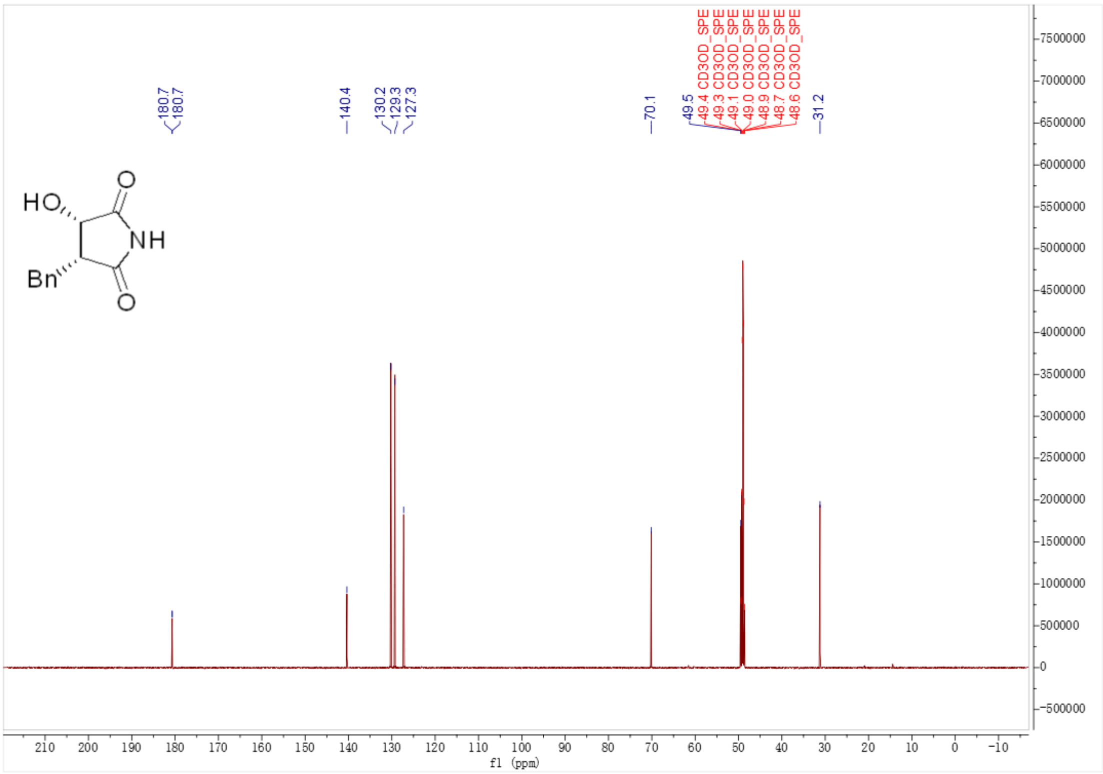

Supplementary Figure 166. ^{13}C NMR of 3y (151 MHz, Methanol-d_4).

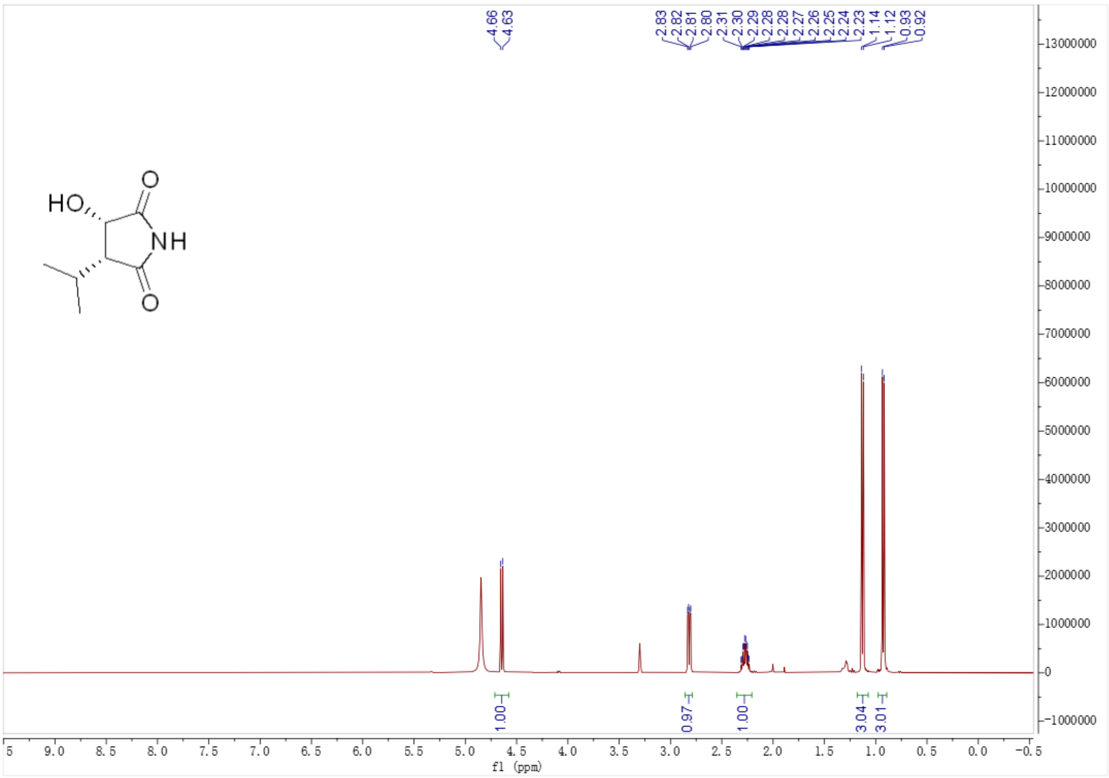

Supplementary Figure 167. ^{1}**H NMR of 3z (400 MHz, Methanol-*d*$_4$).**

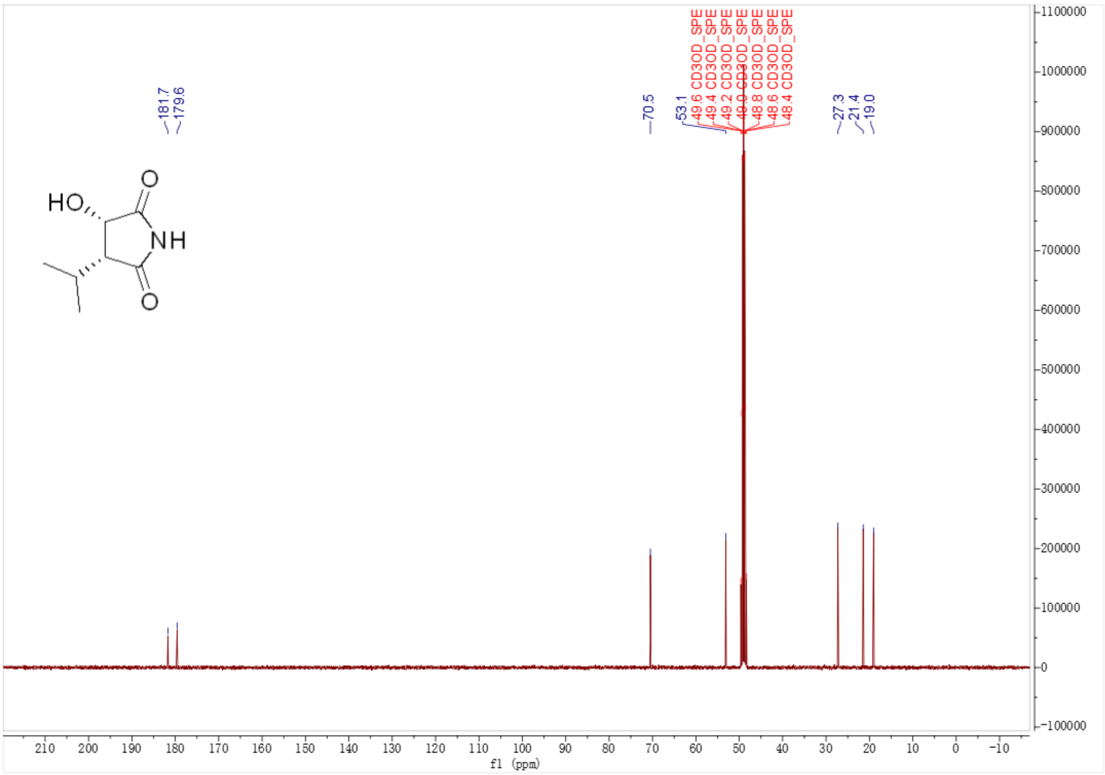

Supplementary Figure 168. ^{13}**C NMR of 3z (101 MHz, Methanol-*d*$_4$).**

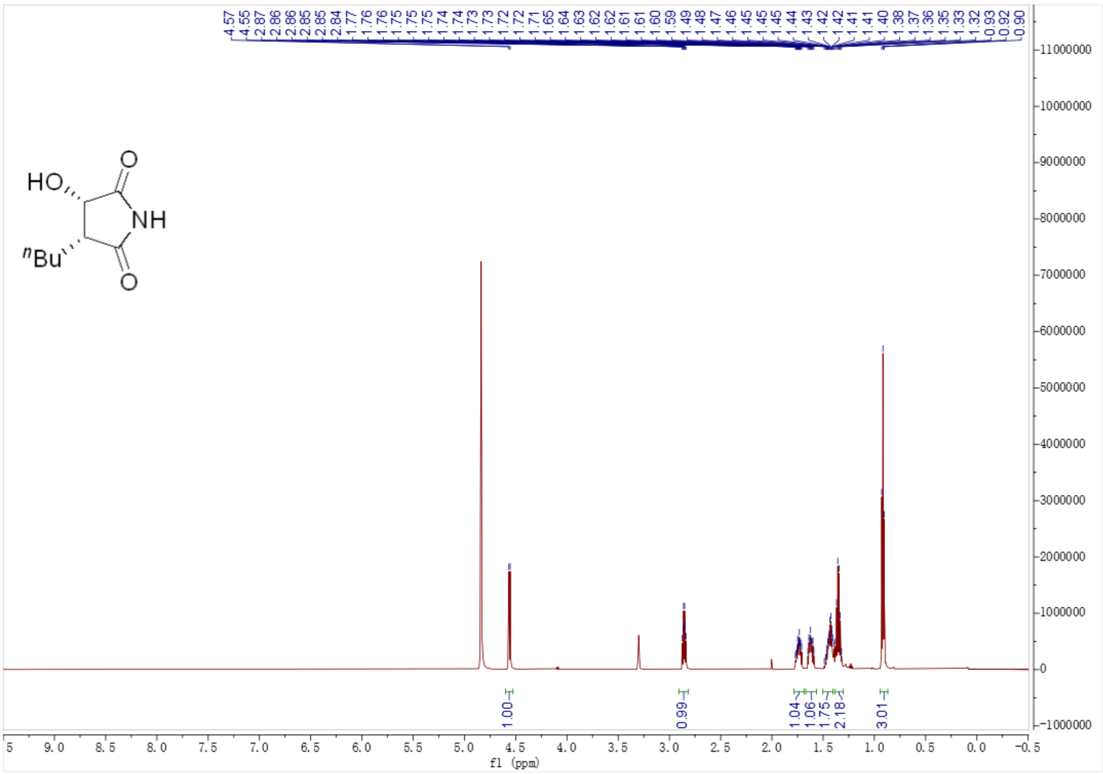

Supplementary Figure 169. ^{1}**H NMR of 3aa (600 MHz, Methanol-*d*$_4$).**

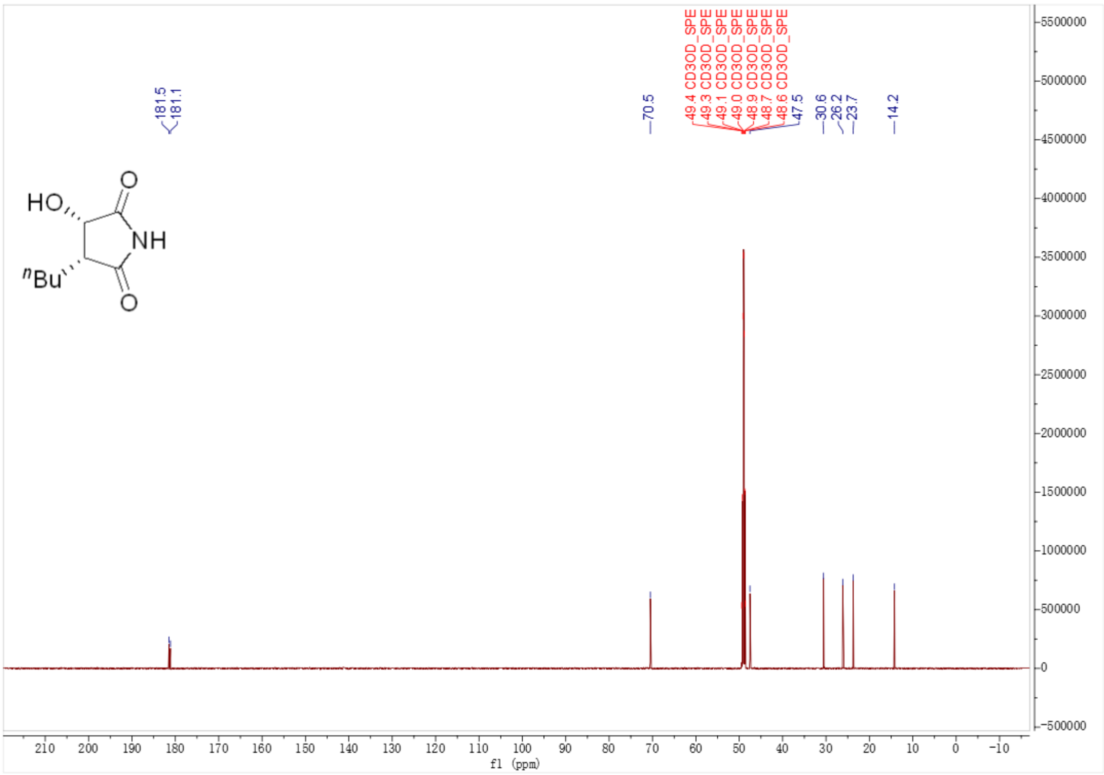

Supplementary Figure 170. ^{13}**C NMR of 3aa (151 MHz, Methanol-*d*$_4$).**

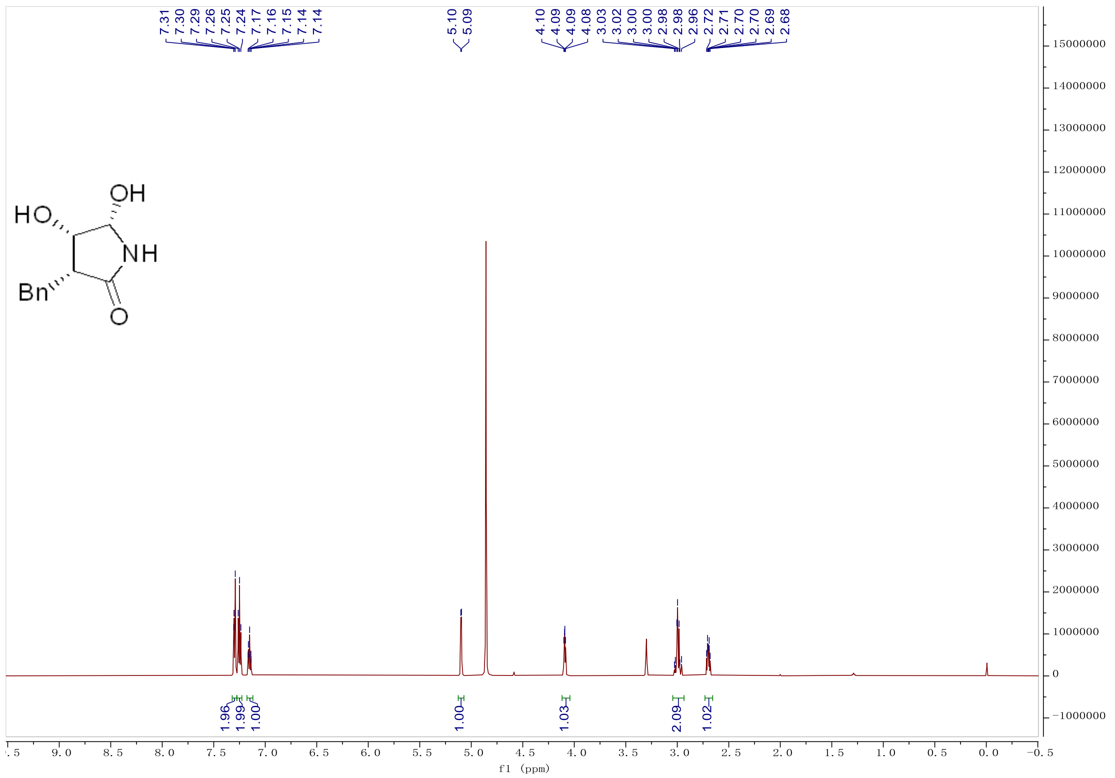

Supplementary Figure 171. ^{1}H NMR of 4y (600 MHz, Methanol-d_4).

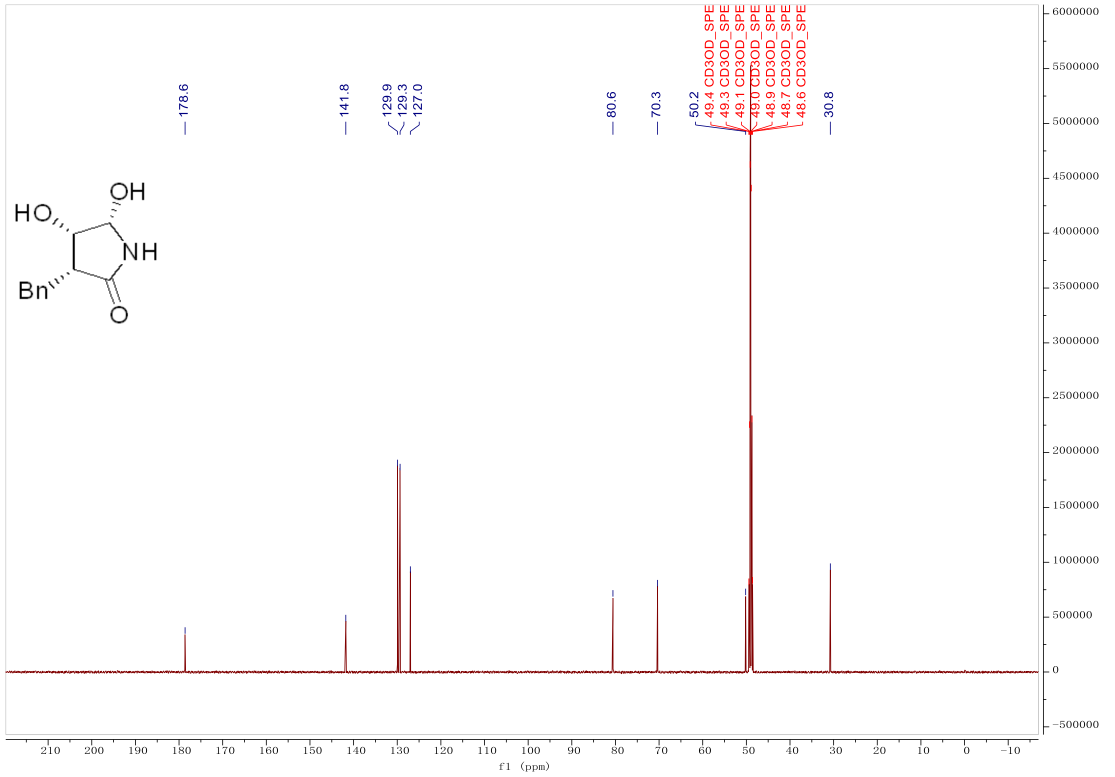

Supplementary Figure 172. ^{13}C NMR of 4y (151 MHz, Methanol-d_4).

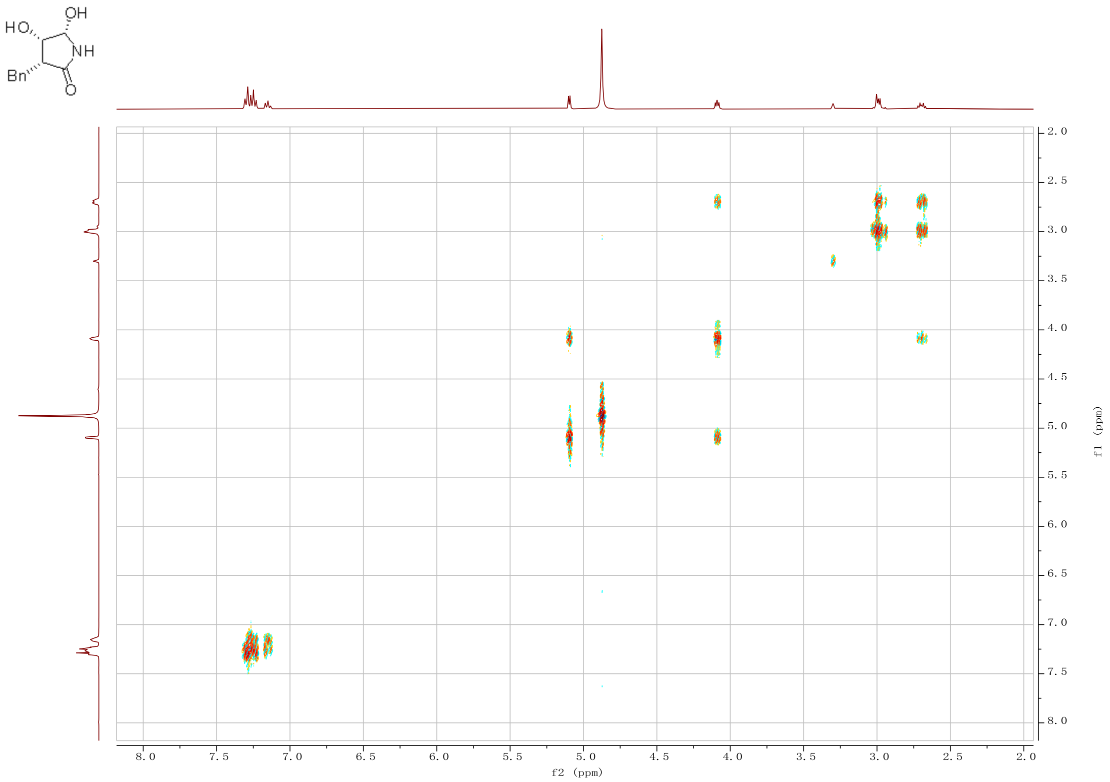

Supplementary Figure 173. 2D-NMR Data of 4y.

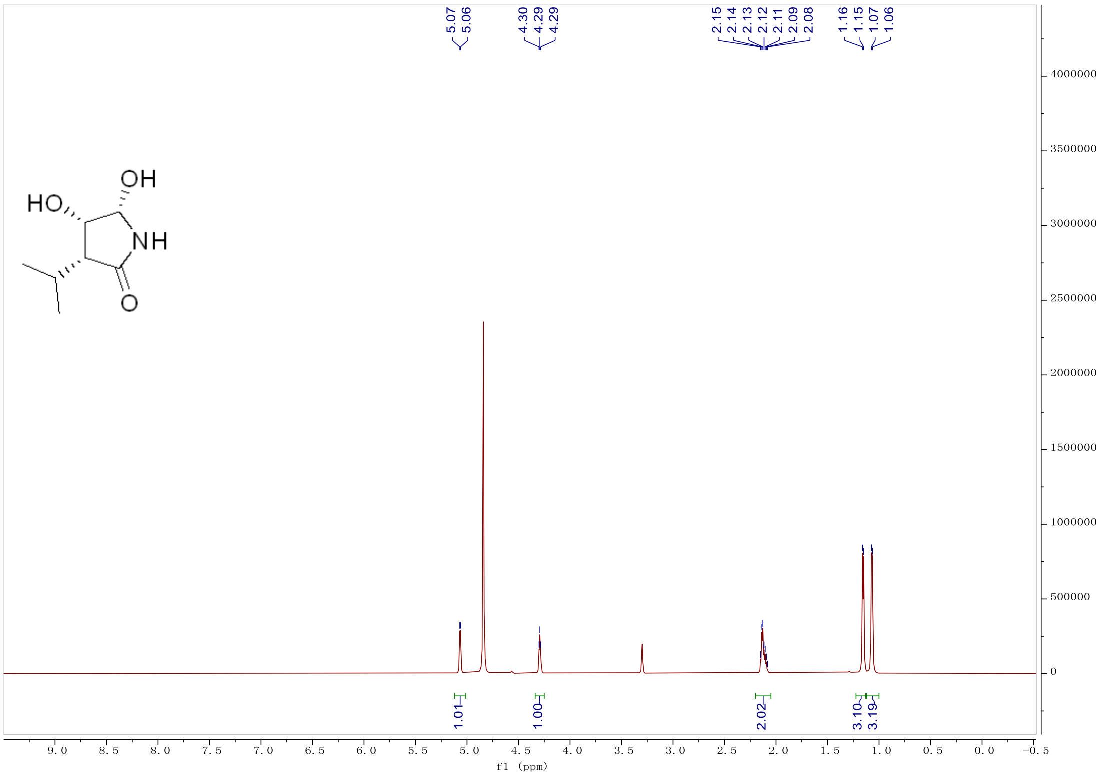

Supplementary Figure 174. ^{1}**H NMR of 4z (600 MHz, Methanol-*d*$_4$).**

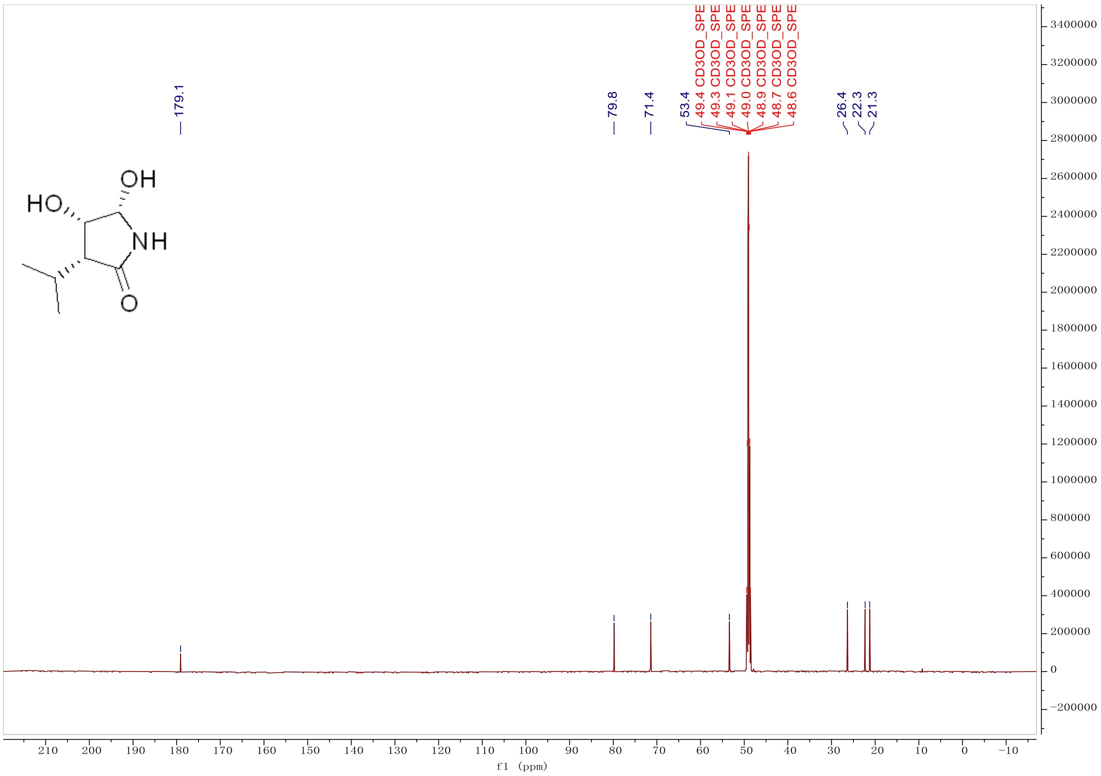

Supplementary Figure 175. ^{13}**C NMR of 4z (151 MHz, Methanol-*d*$_4$).**

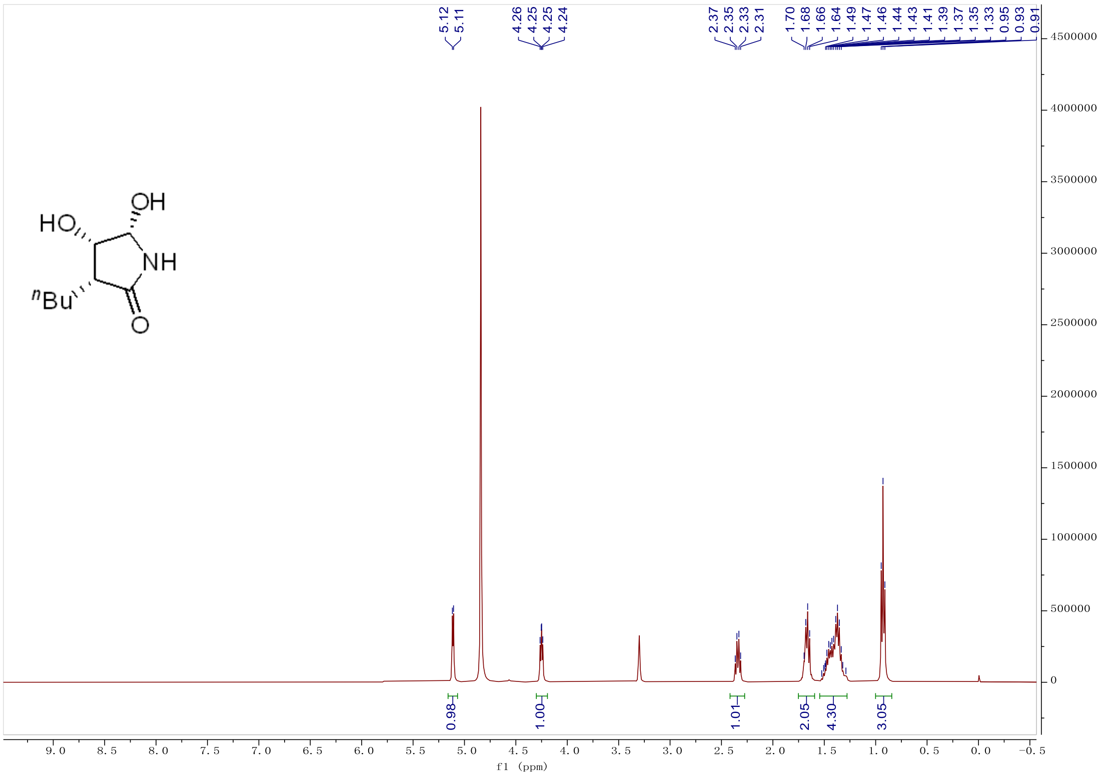

Supplementary Figure 176. ^{1}**H NMR of 4aa (400 MHz, Methanol-*d*$_4$).**

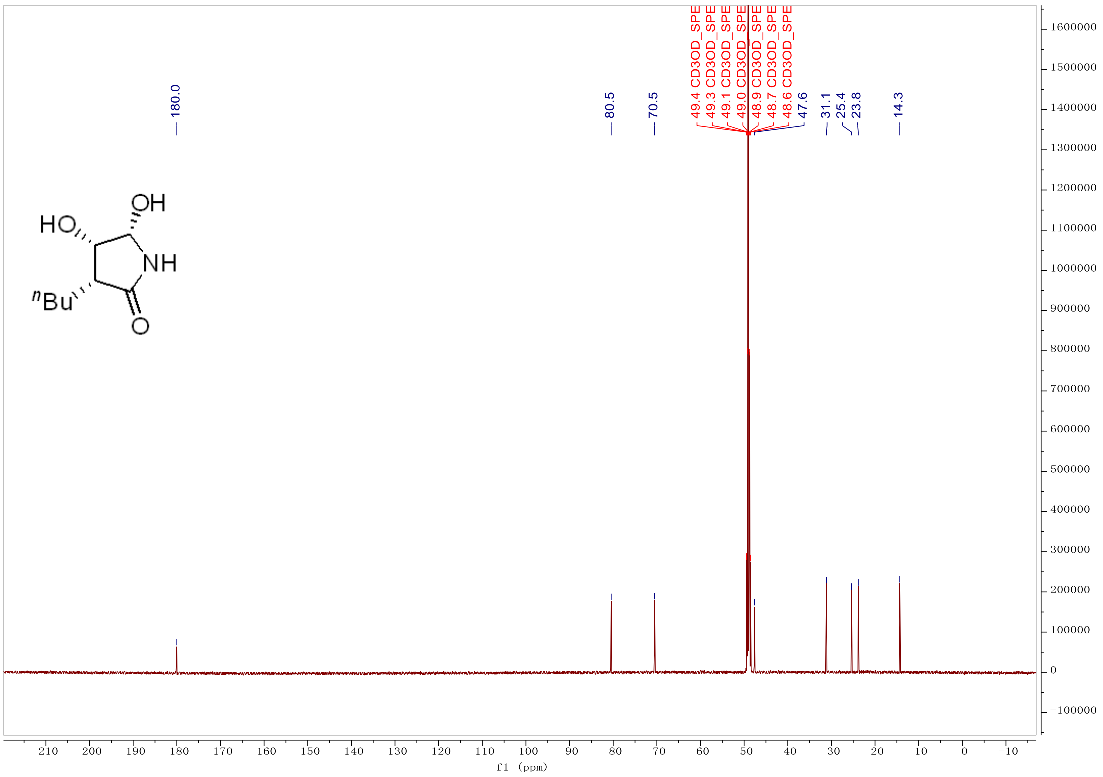

Supplementary Figure 177. ^{13}**C NMR of 4aa (151 MHz, Methanol-*d*$_4$).**

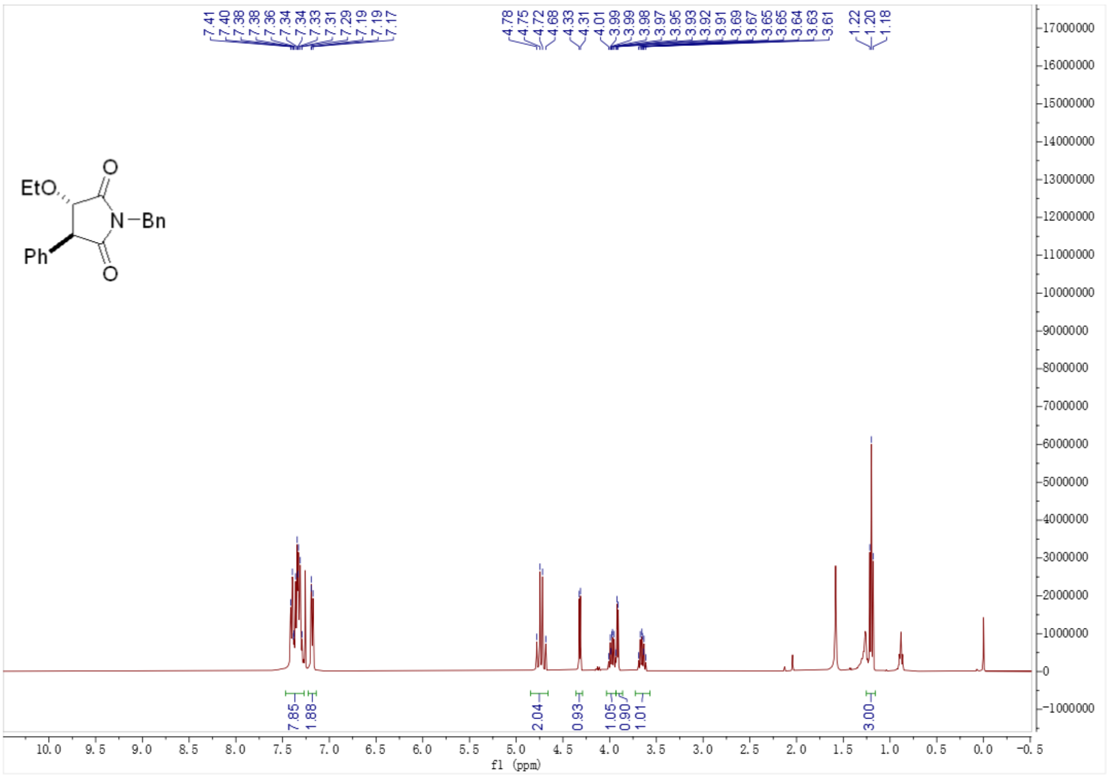

Supplementary Figure 178. ^{1}**H NMR of** ***anti*****-2a' (400 MHz, Chloroform-*d*).**

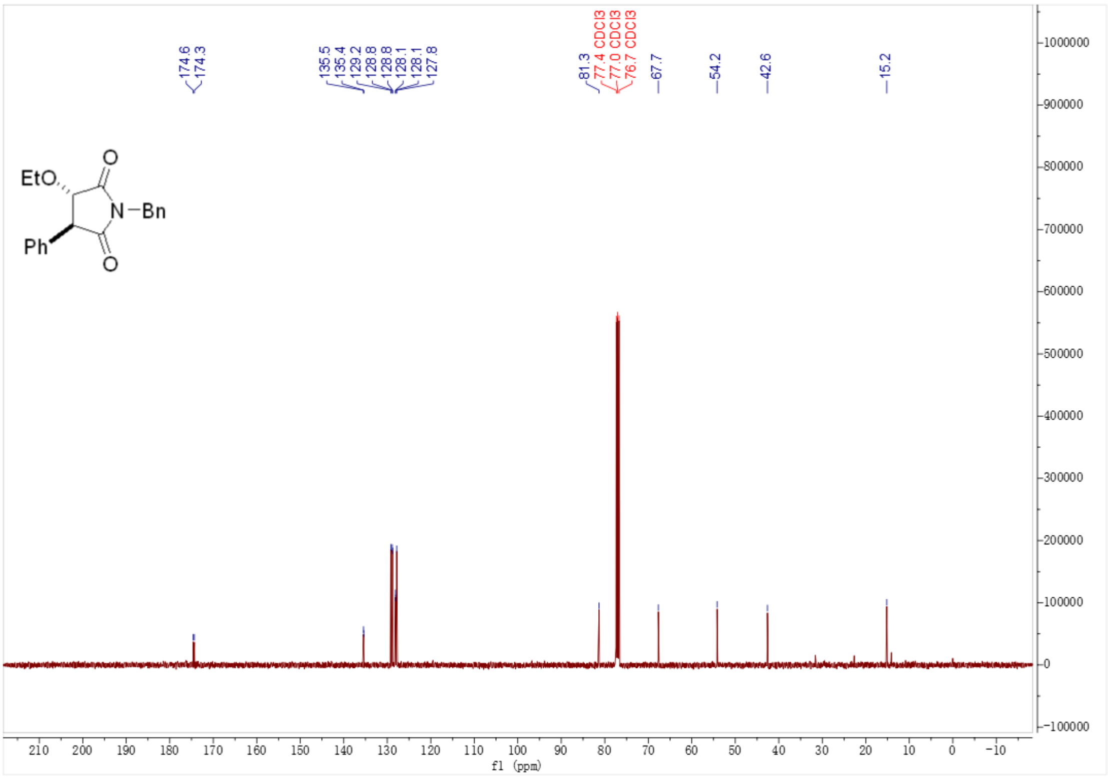

Supplementary Figure 179. ^{13}**C NMR of** ***anti*****-2a' (101 MHz, Chloroform-*d*).**

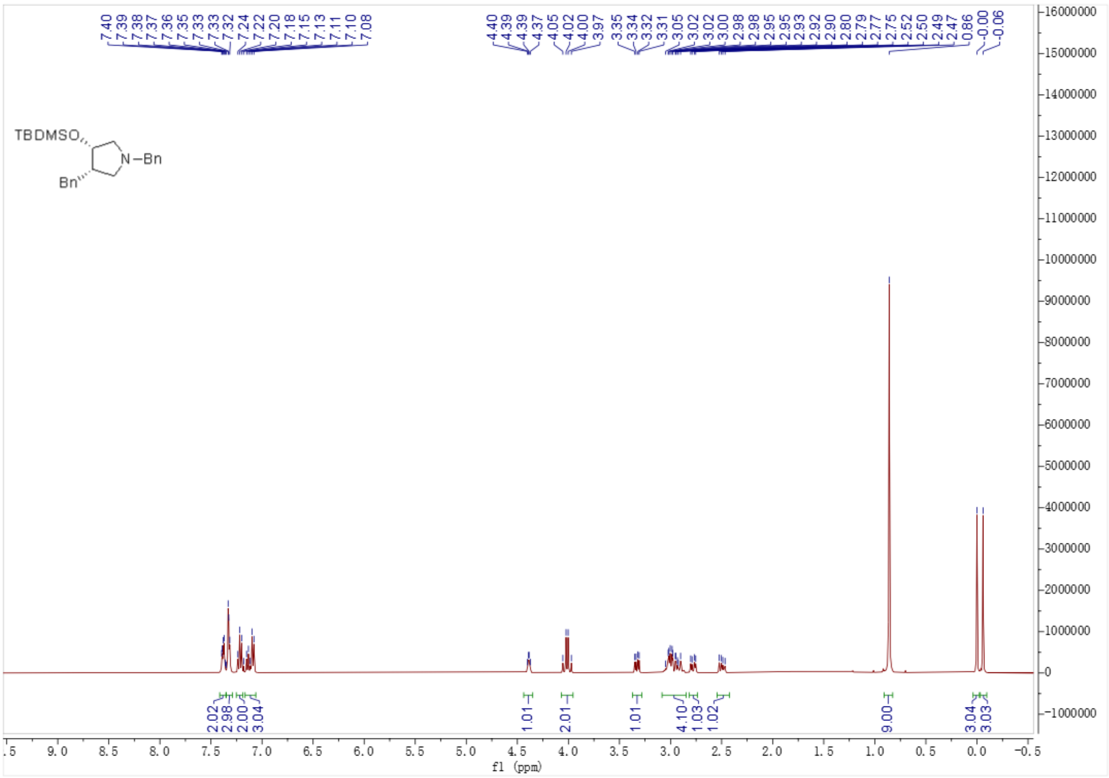

Supplementary Figure 180. ^{1}H NMR of 6w (400 MHz, Chloroform-*d*).

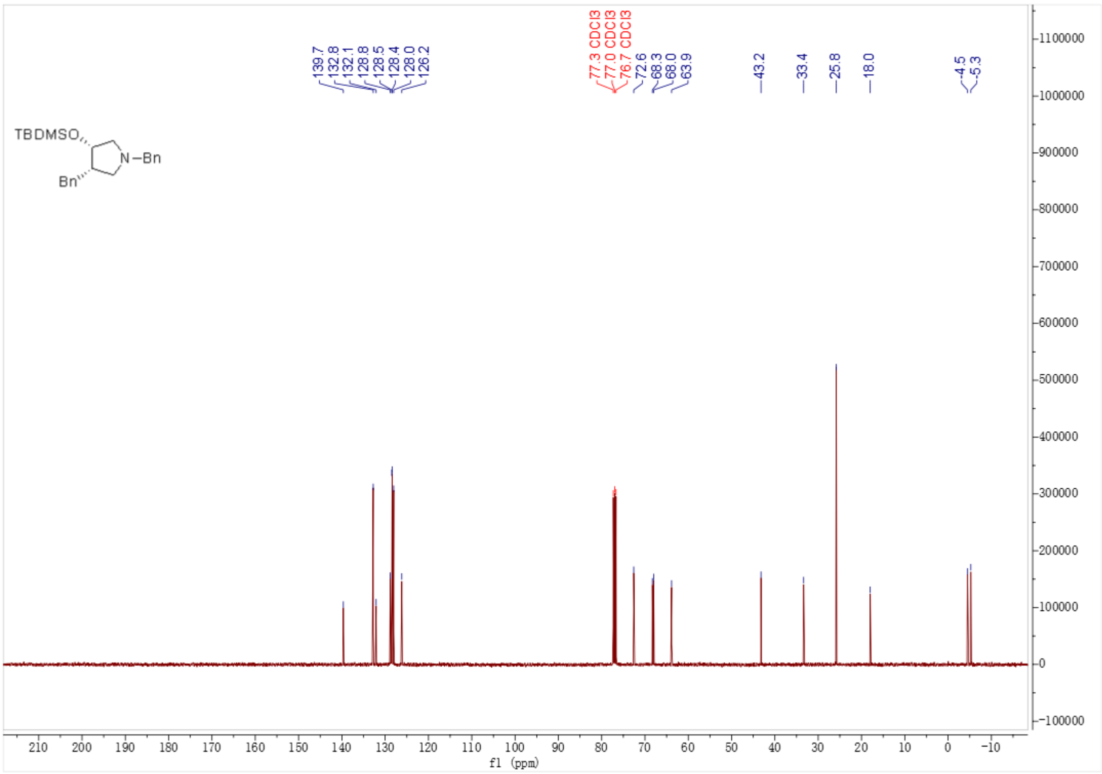

Supplementary Figure 181. ^{13}C NMR of 6w (101 MHz, Chloroform-*d*).

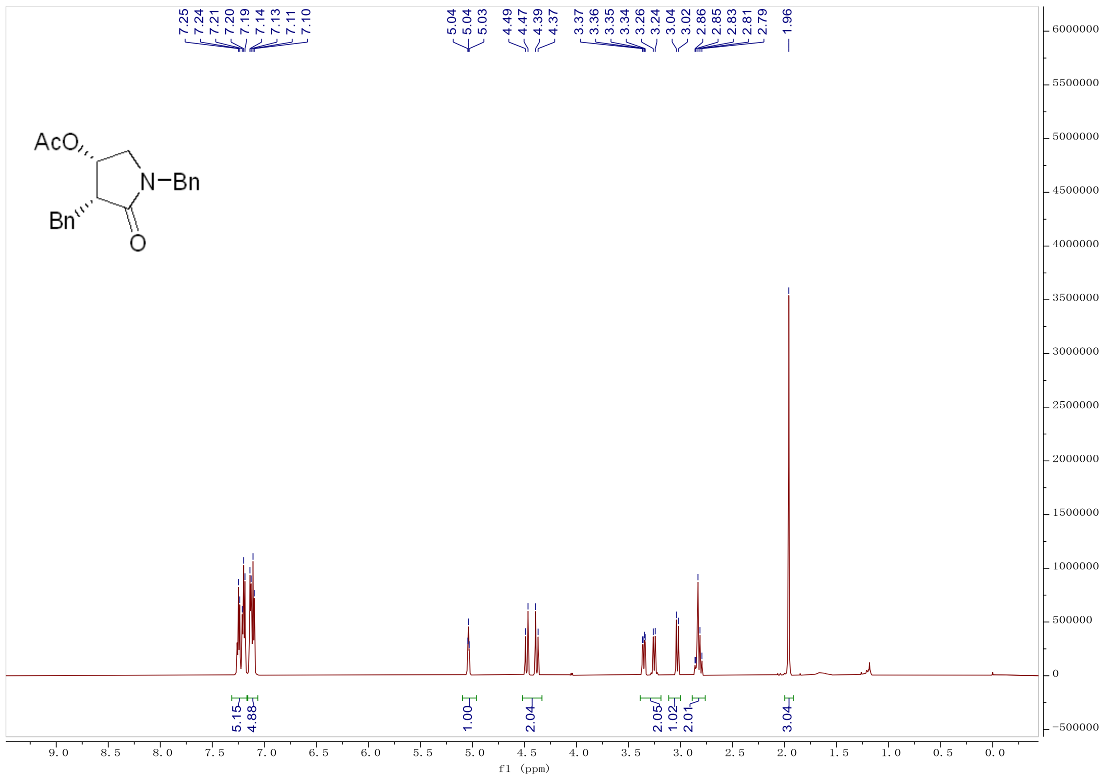

Supplementary Figure 182. ^{1}H NMR of 7w (600 MHz, Chloroform-*d*).

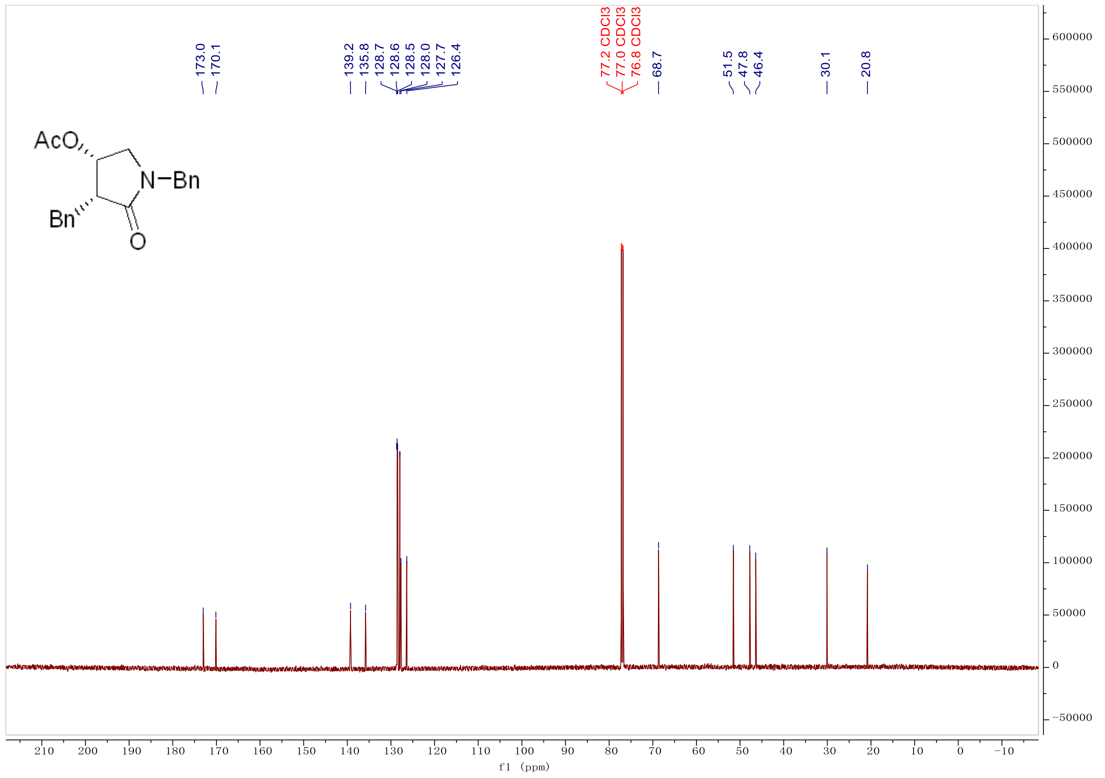

Supplementary Figure 183. ^{13}C NMR of 7w (151 MHz, Chloroform-*d*).

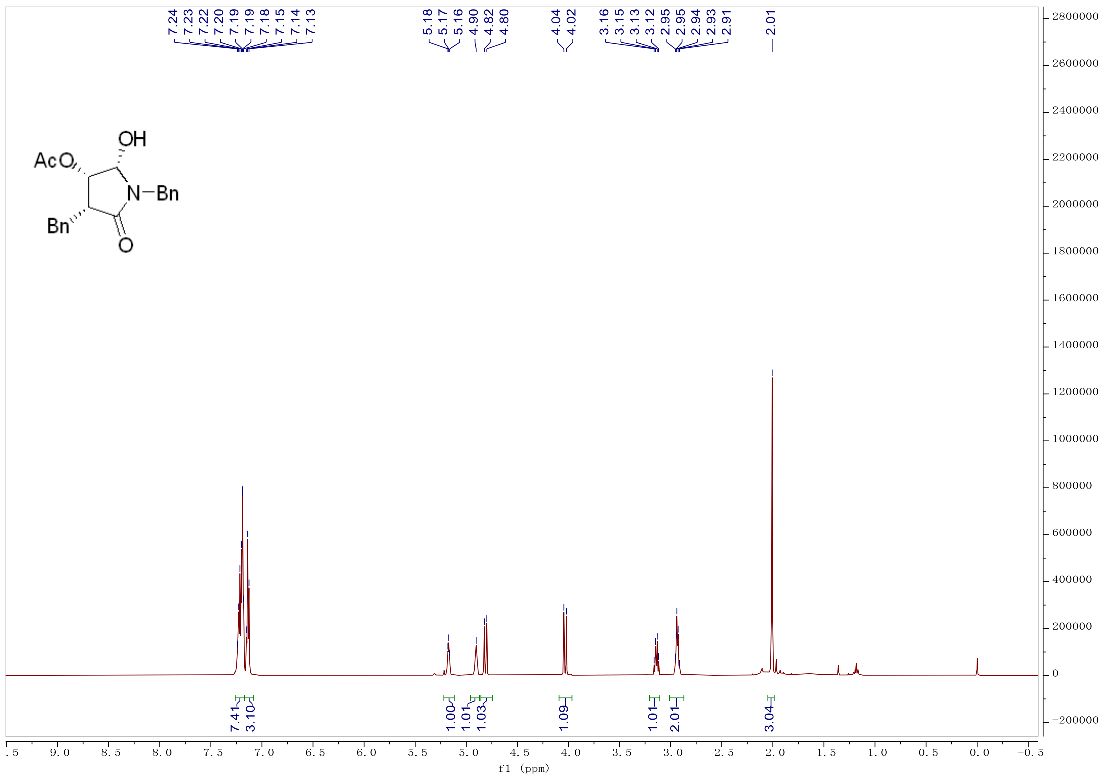

Supplementary Figure 184. ^{1}H NMR of 8w (600 MHz, Chloroform-*d*).

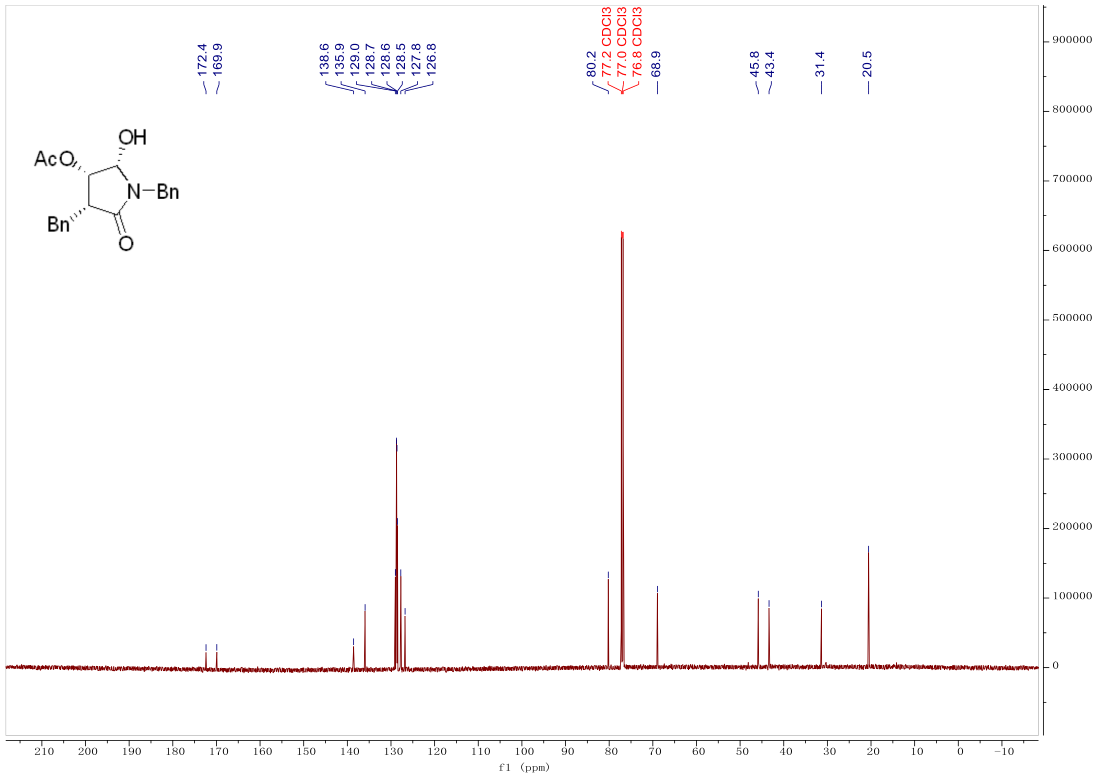

Supplementary Figure 185. ^{13}C NMR of 8w (151 MHz, Chloroform-*d*).

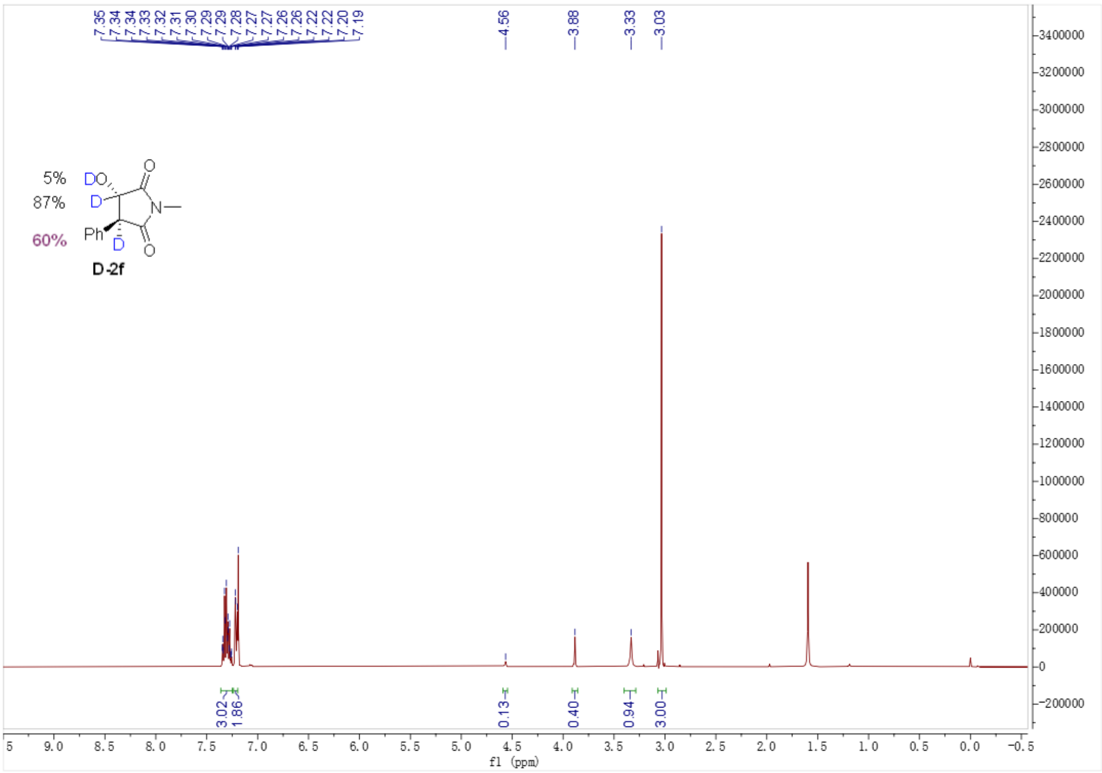

Supplementary Figure 186. ^{1}**H NMR of D-2f (400 MHz, Chloroform-*d*).**

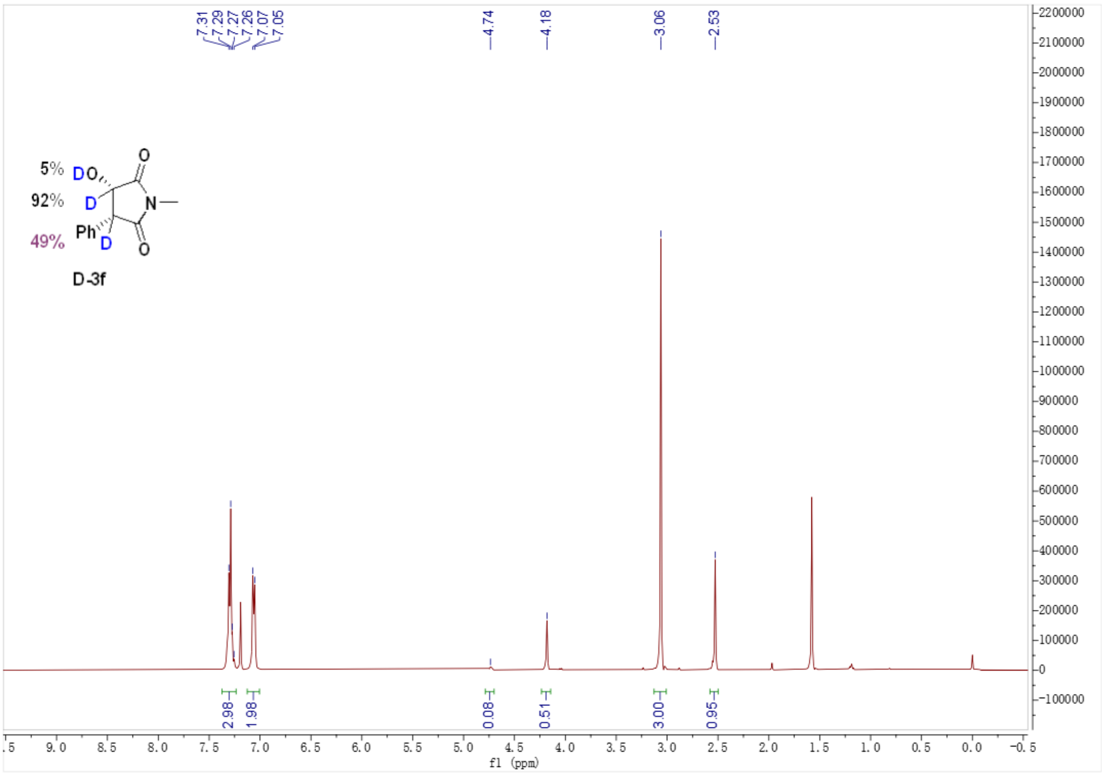

Supplementary Figure 187. ^{1}**H NMR of D-3f (400 MHz, Chloroform-*d*).**

HPLC Traces of the Products

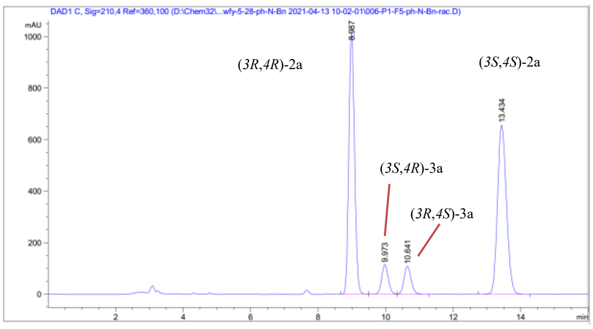

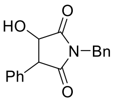

rac-**2a**+*rac*-**3a**

Signal 1: DAD1 C, Sig=210,4 Ref=360,100

Peak #	RetTime [min]	Type	Width [min]	Area [mAU*s]	Height [mAU]	Area %
1	8.987	BB	0.1829	1.18705e4	1011.76978	43.9234
2	9.973	BV	0.2092	1541.71338	114.43020	5.7047
3	10.641	VB	0.2235	1573.96338	108.35789	5.8240
4	13.434	BB	0.2860	1.20393e4	656.44855	44.5480

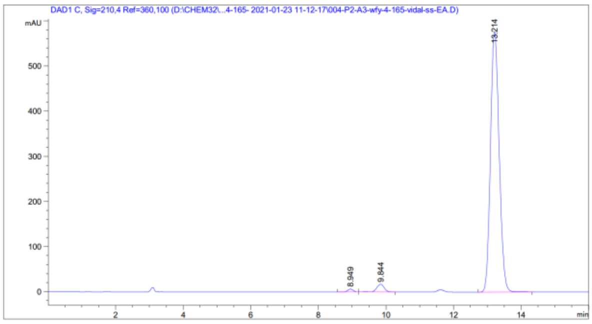

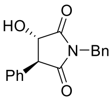

(3*S*,4*S*)-**2a**

Signal 1: DAD1 C, Sig=210,4 Ref=360,100

Peak #	RetTime [min]	Type	Width [min]	Area [mAU*s]	Height [mAU]	Area %
1	8.949	BB	0.1726	68.58959	6.22356	0.6486
2	9.844	VB R	0.2402	259.86292	17.01633	2.4573
3	13.214	BB	0.2772	1.02468e4	576.92462	96.8941

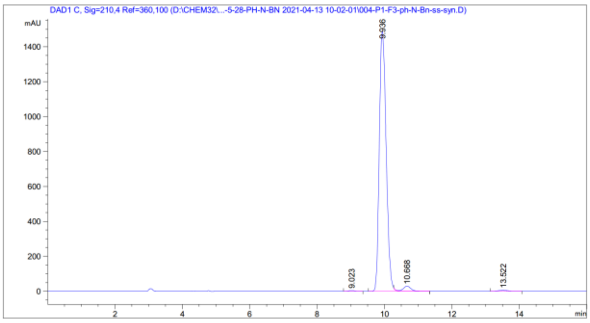

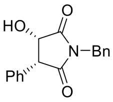

(3*S*,4*R*)-**3a**

Signal 1: DAD1 C, Sig=210,4 Ref=360,100

Peak #	RetTime [min]	Type	Width [min]	Area [mAU*s]	Height [mAU]	Area %
1	9.023	BB	0.1707	41.82415	3.56957	0.1936
2	9.936	BV R	0.2231	2.10202e4	1484.96826	97.3091
3	10.668	VB E	0.2393	443.59378	28.55054	2.0535
4	13.522	BB	0.2645	95.84493	5.21198	0.4437

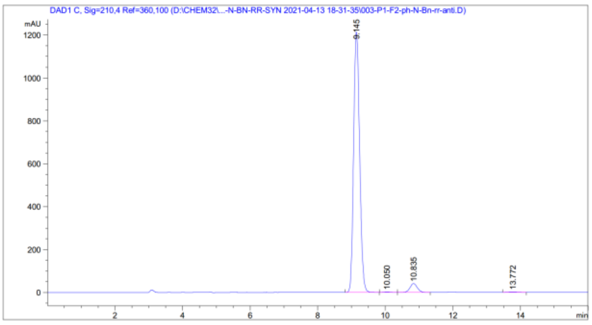

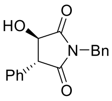

(3*R*,4*R*)-**2a**

Signal 1: DAD1 C, Sig=210,4 Ref=360,100

Peak #	RetTime [min]	Type	Width [min]	Area [mAU*s]	Height [mAU]	Area %
1	9.145	BB	0.1939	1.49472e4	1211.87732	95.7045
2	10.050	BB	0.1890	27.38448	2.17311	0.1753
3	10.835	BB	0.2323	602.15302	40.30375	3.8555
4	13.772	BB	0.2316	41.33091	2.30074	0.2646

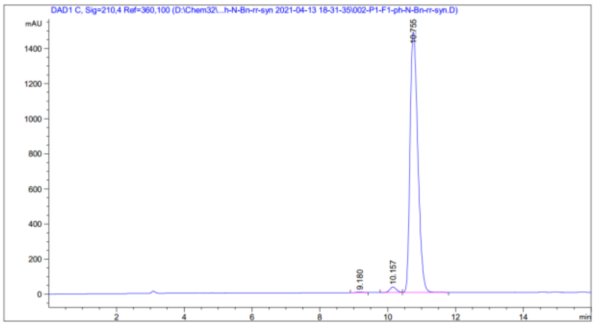

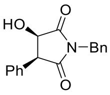

(3*R*,4*S*)-**3a**

Signal 1: DAD1 C, Sig=210,4 Ref=360,100

Peak #	RetTime [min]	Type	Width [min]	Area [mAU*s]	Height [mAU]	Area %
1	9.180	BB	0.1866	50.46713	4.24854	0.2090
2	10.157	BV	0.2129	426.29337	30.90923	1.7651
3	10.755	VB	0.2502	2.36738e4	1483.32959	98.0259

Supplementary Figure 188. HPLC spectrum of 2a and 3a.

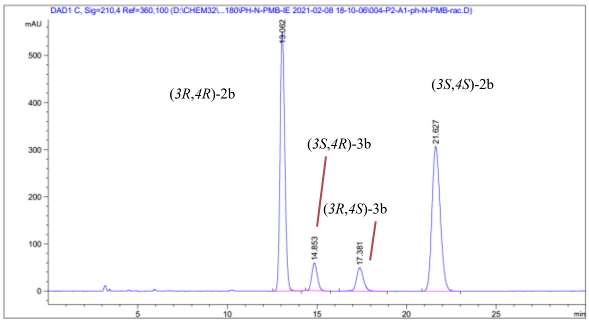

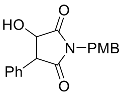

rac-**2b**+*rac*-**3b**

Signal 1: DAD1 C, Sig=210,4 Ref=360,100

Peak #	RetTime [min]	Type	Width [min]	Area [mAU*s]	Height [mAU]	Area %
1	13.062	BB	0.2839	9973.51563	549.12555	44.5631
2	14.853	BB	0.3318	1256.14917	58.66521	5.6127
3	17.381	BB	0.4150	1353.34607	49.81076	6.0469
4	21.627	BB	0.4936	9797.63867	307.60492	43.7773

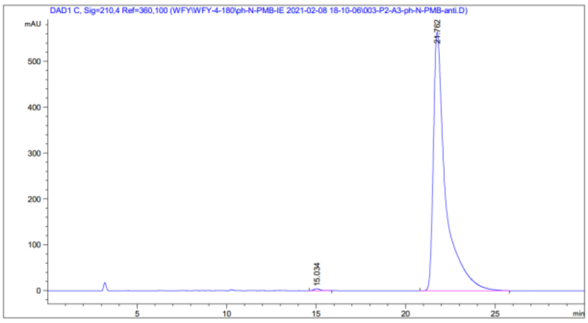

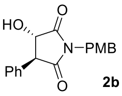

2b

Signal 1: DAD1 C, Sig=210,4 Ref=360,100

Peak #	RetTime [min]	Type	Width [min]	Area [mAU*s]	Height [mAU]	Area %
1	15.034	BB	0.3416	95.21124	4.08981	0.3543
2	21.762	BB	0.6759	2.67772e4	563.99310	99.6457

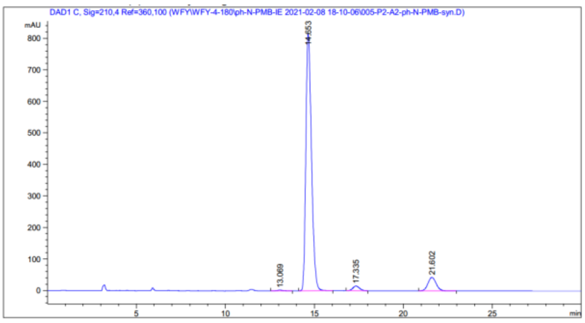

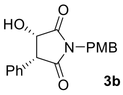

3b

Signal 1: DAD1 C, Sig=210,4 Ref=360,100

Peak #	RetTime [min]	Type	Width [min]	Area [mAU*s]	Height [mAU]	Area %
1	13.069	BB	0.3038	49.94906	2.27807	0.2547
2	14.653	BB	0.3390	1.78219e4	815.27399	90.8682
3	17.335	BB	0.4035	384.76297	14.79100	1.9618
4	21.602	BB	0.4983	1356.30115	42.73129	6.9154

Supplementary Figure 189. HPLC spectrum of 2b and 3b.

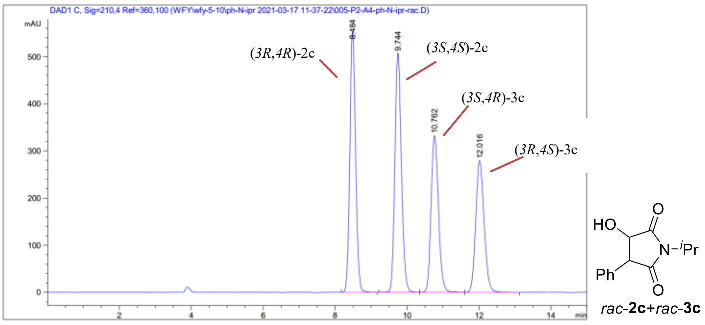

Signal 1: DAD1 C, Sig=210,4 Ref=360,100

Peak #	RetTime [min]	Type	Width [min]	Area [mAU*s]	Height [mAU]	Area %
1	8.484	BB	0.1724	6168.43555	551.78650	28.2835
2	9.744	BB	0.1975	6405.77637	506.82608	29.3717
3	10.762	BB	0.2194	4704.78564	331.94870	21.5724
4	12.016	BB	0.2514	4530.33447	279.12256	20.7725

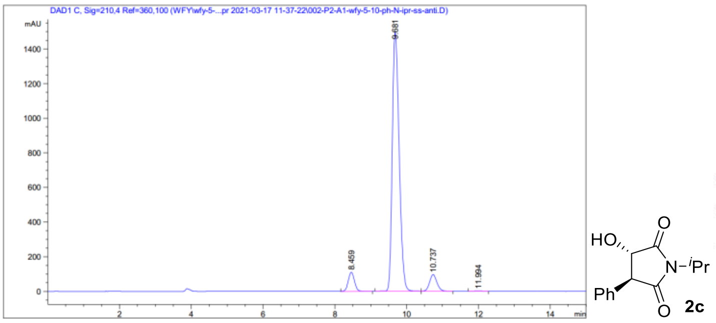

Signal 1: DAD1 C, Sig=210,4 Ref=360,100

Peak #	RetTime [min]	Type	Width [min]	Area [mAU*s]	Height [mAU]	Area %
1	8.459	BB	0.1820	1305.62488	110.37617	5.7615
2	9.681	VV R	0.2094	1.99578e4	1496.74475	88.0707
3	10.737	VB	0.2204	1372.39282	96.19571	6.0562
4	11.994	BB	0.2222	25.29548	1.63810	0.1116

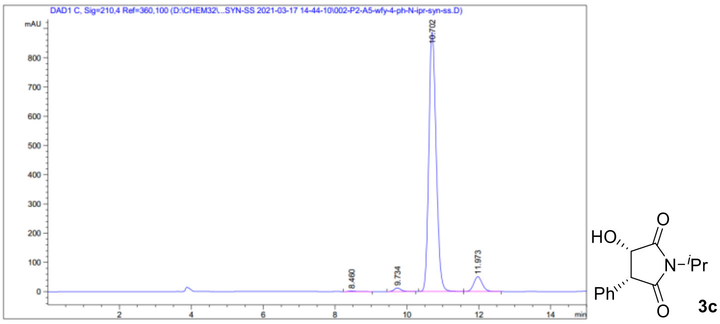

Peak #	RetTime [min]	Type	Width [min]	Area [mAU*s]	Height [mAU]	Area %
1	8.460	BB	0.2357	33.44870	1.97404	0.2459
2	9.734	BB	0.2044	161.40421	12.19450	1.1864
3	10.702	BB	0.2216	1.25859e4	886.46332	92.5089
4	11.973	BB	0.2517	824.30878	50.70269	6.0589

Supplementary Figure 190. HPLC spectrum of 2c and 3c.

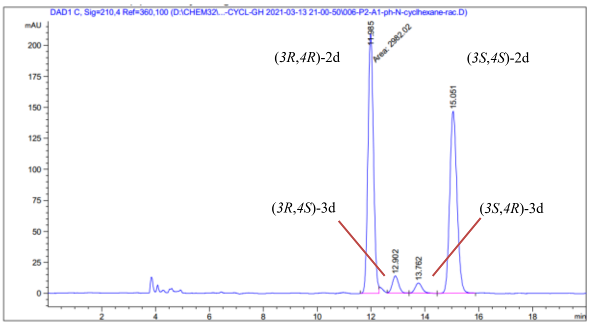

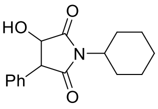

rac-**2d**+*rac*-**3d**

Signal 1: DAD1 C, Sig=210,4 Ref=360,100

Peak #	RetTime [min]	Type	Width [min]	Area [mAU*s]	Height [mAU]	Area %
1	11.985	MF	0.2376	2982.02441	209.19566	48.7429
2	12.902	VB	0.2552	226.62941	13.82799	3.7044
3	13.762	BB	0.2820	148.11926	8.15102	2.4211
4	15.051	BB	0.2917	2761.09668	146.59666	45.1317

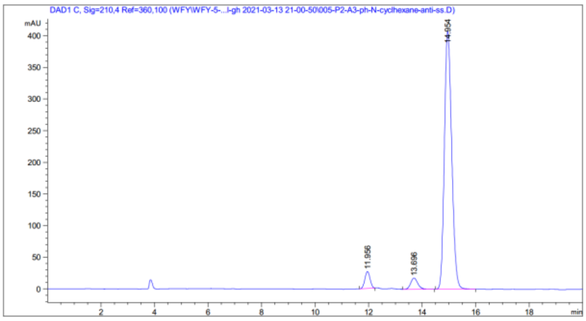

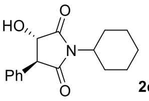

2d

Signal 1: DAD1 C, Sig=210,4 Ref=360,100

Peak #	RetTime [min]	Type	Width [min]	Area [mAU*s]	Height [mAU]	Area %
1	11.956	BB	0.2109	359.16931	26.70136	4.2734
2	13.696	BB	0.2844	326.00449	17.73683	3.8788
3	14.954	BB	0.2953	7719.66553	407.04800	91.8479

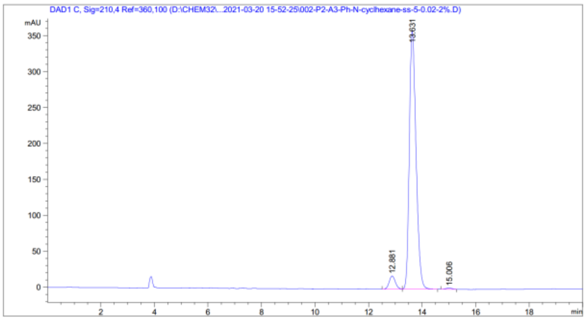

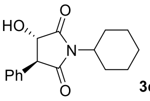

3d

Signal 1: DAD1 C, Sig=210,4 Ref=360,100

Peak #	RetTime [min]	Type	Width [min]	Area [mAU*s]	Height [mAU]	Area %
1	12.881	BV	0.2473	289.47476	18.22820	4.4018
2	13.631	VB	0.2718	6261.53223	358.53290	95.2149
3	15.006	BB	0.2066	25.20657	1.48074	0.3833

Supplementary Figure 191. HPLC spectrum of 2d and 3d.

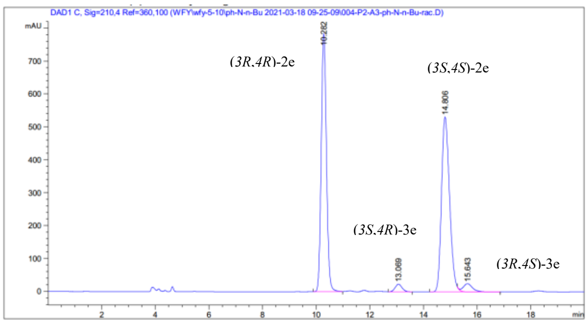

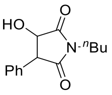

rac-**2e**+*rac*-**3e**

Signal 1: DAD1 C, Sig=210,4 Ref=360,100

Peak #	RetTime [min]	Type	Width [min]	Area [mAU*s]	Height [mAU]	Area %
1	10.282	BB	0.2117	1.05815e4	782.85449	46.6403
2	13.069	BB	0.2724	407.29294	23.02354	1.7952
3	14.806	BV R	0.3237	1.10996e4	531.40137	48.9242
4	15.643	VB E	0.3614	599.02087	24.83741	2.6403

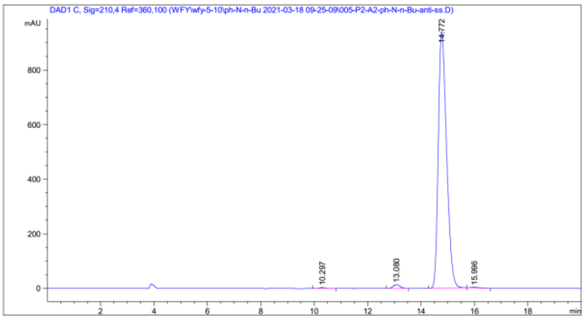

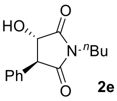

2e

Signal 1: DAD1 C, Sig=210,4 Ref=360,100

Peak #	RetTime [min]	Type	Width [min]	Area [mAU*s]	Height [mAU]	Area %
1	10.297	BB	0.2151	52.55459	3.62669	0.2555
2	13.080	BB	0.2815	246.78821	13.87369	1.1997
3	14.772	BB	0.3328	2.02233e4	940.67950	98.3093
4	15.996	BB	0.2740	48.44646	2.41500	0.2355

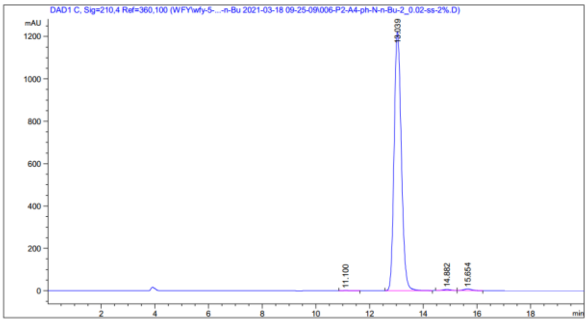

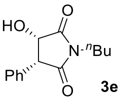

3e

Signal 1: DAD1 C, Sig=210,4 Ref=360,100

Peak #	RetTime [min]	Type	Width [min]	Area [mAU*s]	Height [mAU]	Area %
1	11.100	BB	0.2360	17.46226	1.03950	0.0760
2	13.039	BB	0.2907	2.27001e4	1221.86658	98.7752
3	14.882	BB	0.2993	99.77814	5.03312	0.4342
4	15.654	BB	0.3237	164.24055	7.61409	0.7147

Supplementary Figure 192. HPLC spectrum of 2e and 3e.

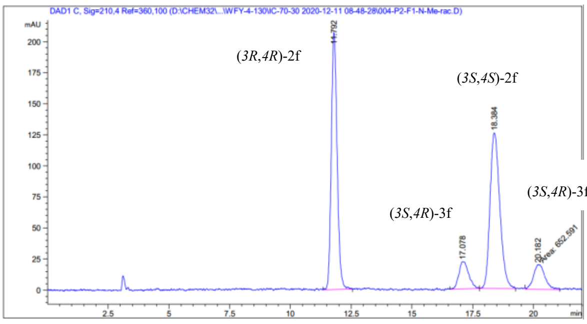

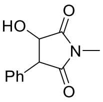

rac-**2f**+*rac*-**3f**

Signal 1: DAD1 C, Sig=210,4 Ref=360,100

Peak #	RetTime [min]	Type	Width [min]	Area [mAU*s]	Height [mAU]	Area %
1	11.792	VV R	0.2590	3452.62500	206.53889	42.0484
2	17.078	VV R	0.3398	617.80194	21.98939	7.5240
3	18.384	BV R	0.3829	3488.04736	125.23566	42.4798
4	20.182	MM	0.5447	652.59125	19.96681	7.9477

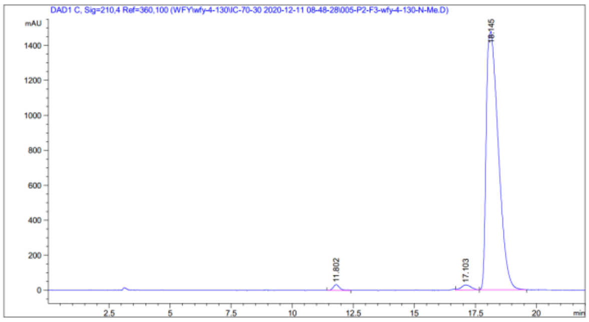

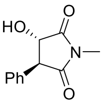

(3*S*,4*S*)-**2f**

Signal 1: DAD1 C, Sig=210,4 Ref=360,100

Peak #	RetTime [min]	Type	Width [min]	Area [mAU*s]	Height [mAU]	Area %
1	11.802	BV R	0.2493	586.07831	32.64530	1.1200
2	17.103	VV R	0.3351	788.67322	28.65168	1.5071
3	18.145	BV R	0.4168	5.09545e4	1475.95313	97.3729

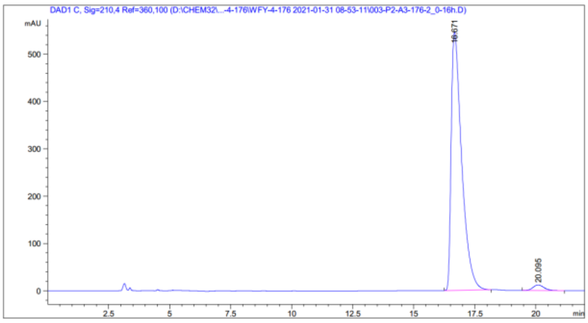

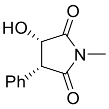

(3*S*,4*R*)-**3f**

Signal 1: DAD1 C, Sig=210,4 Ref=360,100

Peak #	RetTime [min]	Type	Width [min]	Area [mAU*s]	Height [mAU]	Area %
1	16.671	BB	0.4504	1.63004e4	545.81439	97.7784
2	20.095	BB	0.4778	370.36017	11.87546	2.2216

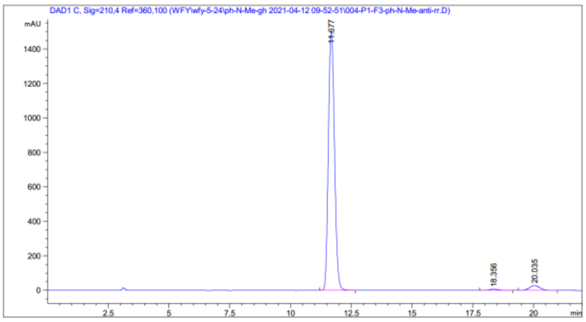

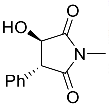

(3*R*,4*R*)-**2f**

Signal 1: DAD1 C, Sig=210,4 Ref=360,100

Peak #	RetTime [min]	Type	Width [min]	Area [mAU*s]	Height [mAU]	Area %
1	11.677	BB	0.2755	2.61188e4	1497.41528	96.2270
2	18.356	BB	0.3838	180.19313	6.53360	0.6639
3	20.035	BB	0.4545	843.90204	28.09366	3.1091

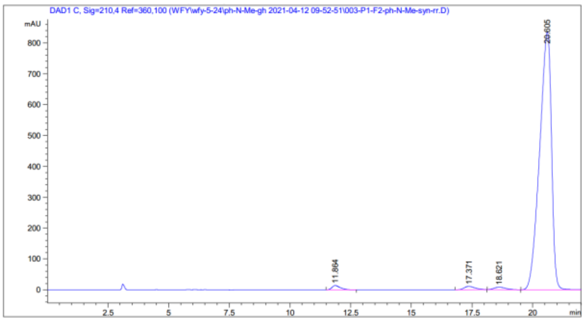

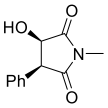

(3*R*,4*S*)-**3f**

Signal 1: DAD1 C, Sig=210,4 Ref=360,100

Peak #	RetTime [min]	Type	Width [min]	Area [mAU*s]	Height [mAU]	Area %
1	11.864	BB	0.3461	329.78915	13.93379	1.1268
2	17.371	BB	0.4365	330.20874	10.68675	1.1282
3	18.621	BB	0.4264	270.49686	8.16664	0.9242
4	20.605	BB	0.5240	2.83377e4	834.94324	96.8208

Supplementary Figure 193. HPLC spectrum of 2f and 3f.

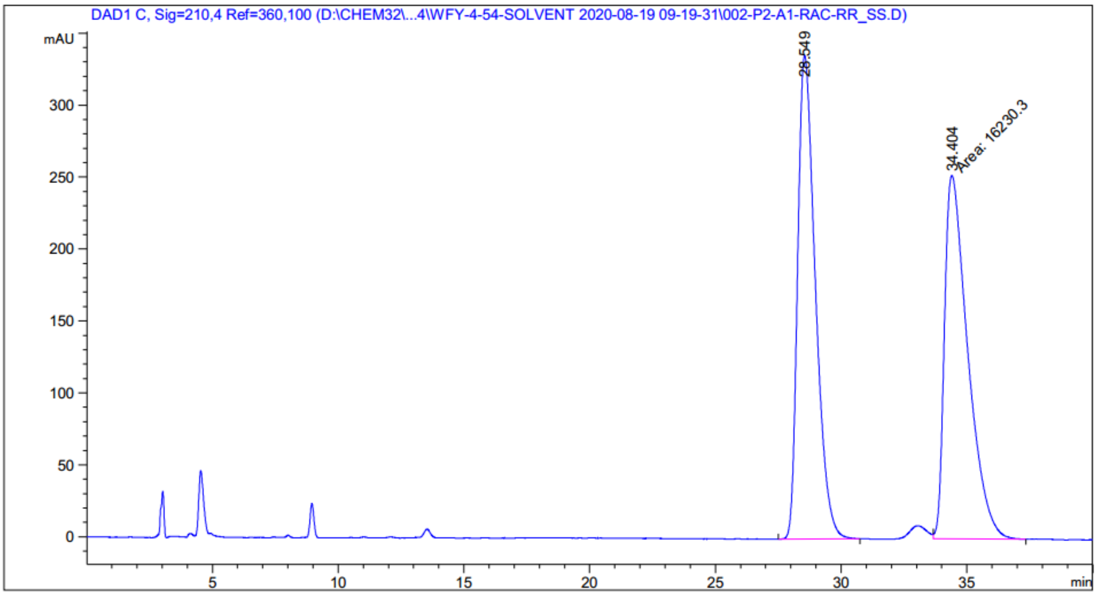

Signal 1: DAD1 C, Sig=210,4 Ref=360,100

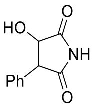

rac-**2g**

Peak #	RetTime [min]	Type	Width [min]	Area [mAU*s]	Height [mAU]	Area %
1	28.549	BB	0.7422	1.64269e4	336.11041	50.3010
2	34.404	FM	1.0708	1.62303e4	252.62325	49.6990

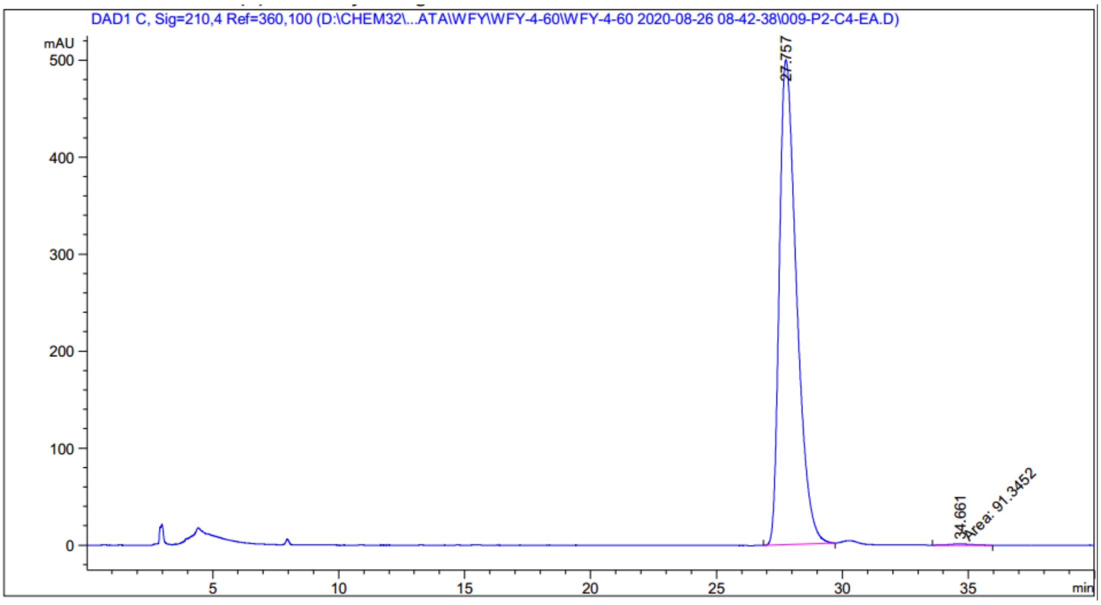

Signal 1: DAD1 C, Sig=210,4 Ref=360,100

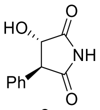

2g

Peak #	RetTime [min]	Type	Width [min]	Area [mAU*s]	Height [mAU]	Area %
1	27.757	BB	0.7230	2.37731e4	499.74857	99.6172
2	34.661	MM	1.0967	91.34515	1.38816	0.3828

Supplementary Figure 194. HPLC spectrum of 2g and 3g.

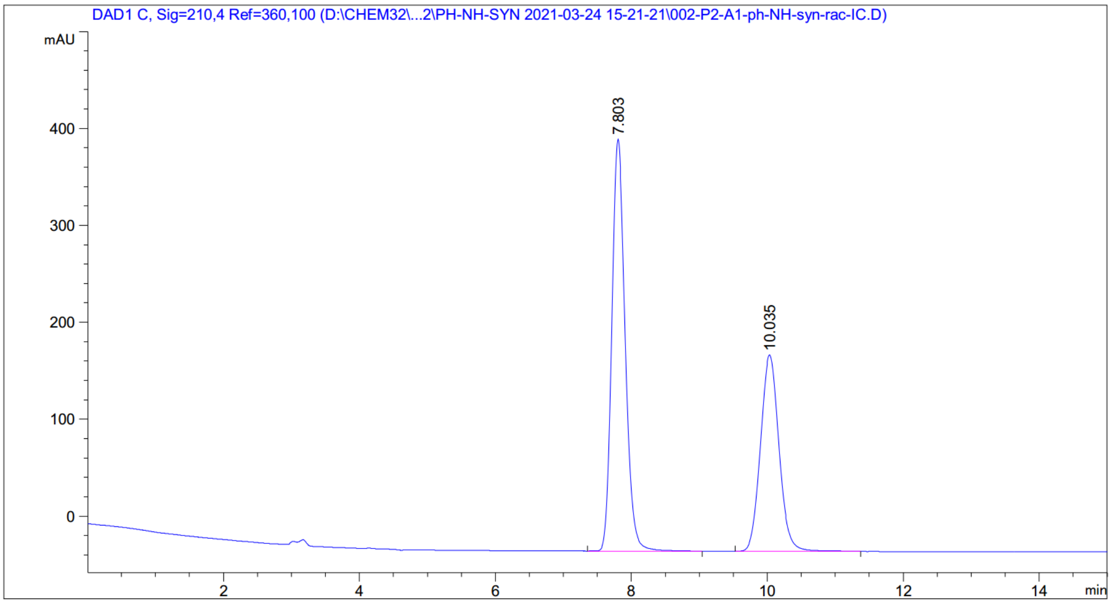

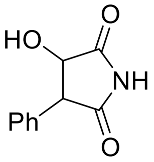

rac-**3g**

Signal 1: DAD1 C, Sig=210,4 Ref=360,100

Peak #	RetTime [min]	Type	Width [min]	Area [mAU*s]	Height [mAU]	Area %
1	7.803	BB	0.2091	5795.01123	424.81644	60.1569
2	10.035	BB	0.2951	3838.14478	202.56071	39.8431

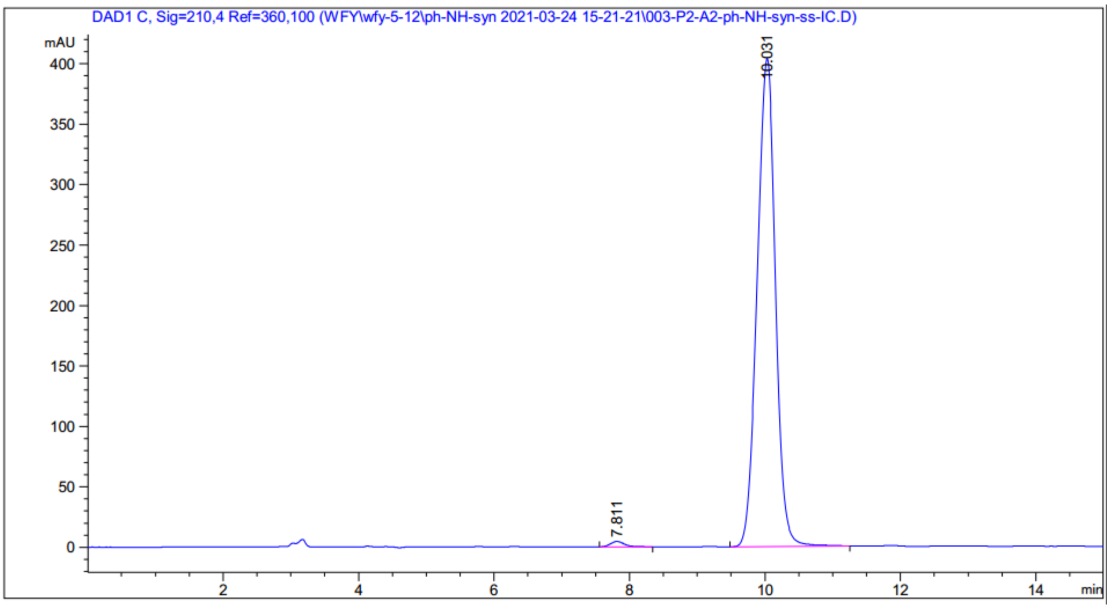

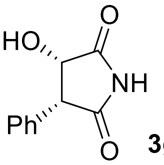

3g

Signal 1: DAD1 C, Sig=210,4 Ref=360,100

Peak #	RetTime [min]	Type	Width [min]	Area [mAU*s]	Height [mAU]	Area %
1	7.811	BB	0.2136	63.56745	4.42503	0.8334
2	10.031	BB	0.2926	7564.27979	403.75253	99.1666

Supplementary Figure 195. HPLC spectrum of 3g.

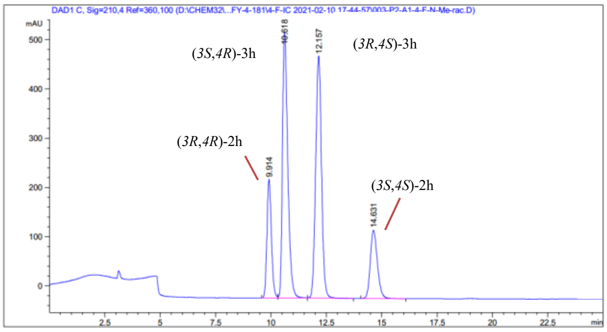

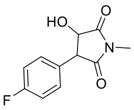

rac-**2h**+*rac*-**3h**

Signal 1: DAD1 C, Sig=210,4 Ref=360,100

Peak #	RetTime [min]	Type	Width [min]	Area [mAU*s]	Height [mAU]	Area %
1	9.914	BV	0.2181	3388.80249	240.91788	13.8457
2	10.618	VB	0.2546	8985.71582	538.81805	36.7131
3	12.157	BB	0.2837	9002.44043	491.54105	36.7814
4	14.631	BB	0.3445	3098.54736	138.78181	12.6598

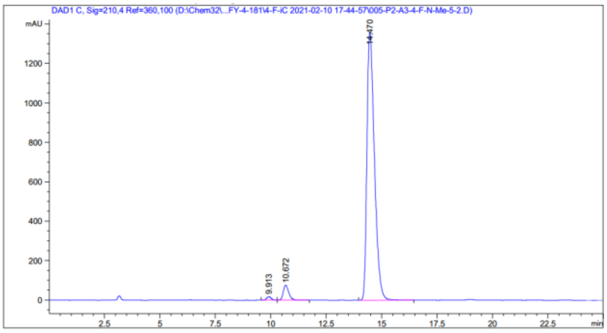

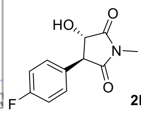

2h

Signal 1: DAD1 C, Sig=210,4 Ref=360,100

Peak #	RetTime [min]	Type	Width [min]	Area [mAU*s]	Height [mAU]	Area %
1	9.913	BB	0.2223	246.23917	17.27936	0.7355
2	10.672	BB	0.2609	1306.05029	76.62646	3.9013
3	14.470	BB	0.3649	3.19252e4	1354.94165	95.3632

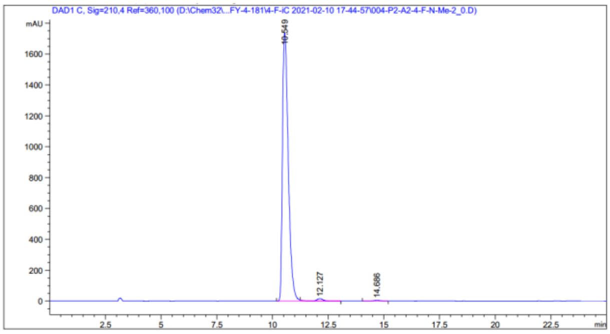

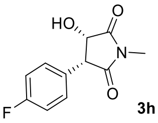

3h

Signal 1: DAD1 C, Sig=210,4 Ref=360,100

Peak #	RetTime [min]	Type	Width [min]	Area [mAU*s]	Height [mAU]	Area %
1	10.549	BV R	0.2820	3.15507e4	1736.53320	98.5130
2	12.127	VB E	0.3307	348.45157	15.59382	1.0880
3	14.686	BB	0.3467	127.79789	5.54846	0.3990

Supplementary Figure 196. HPLC spectrum of 2h and 3h.

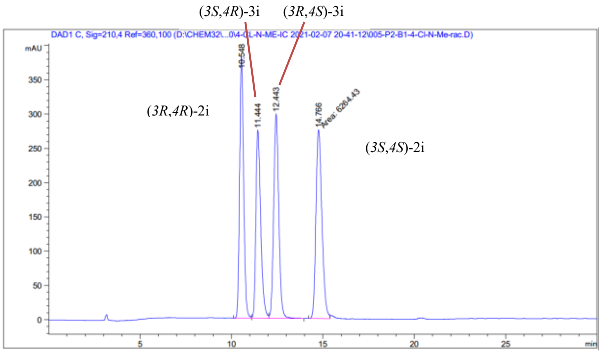

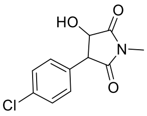

rac-**2i**+*rac*-**3i**

Signal 1: DAD1 C, Sig=210,4 Ref=360,100

Peak #	RetTime [min]	Type	Width [min]	Area [mAU*s]	Height [mAU]	Area %
1	10.548	BV	0.2365	5838.89795	381.52402	25.4358
2	11.444	VV	0.2825	5090.94287	274.29379	22.1775
3	12.443	VB	0.2975	5761.19434	298.09537	25.0973
4	14.766	MF	0.3790	6264.42969	275.45059	27.2895

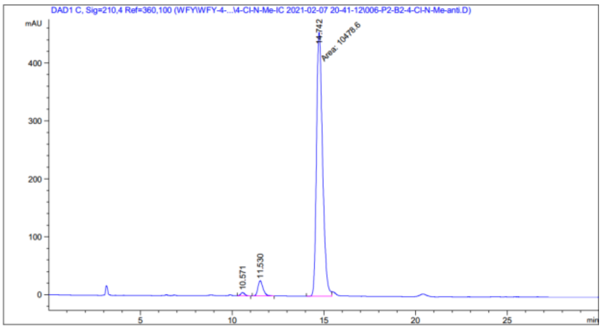

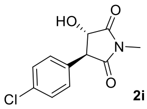

2i

Signal 1: DAD1 C, Sig=210,4 Ref=360,100

Peak #	RetTime [min]	Type	Width [min]	Area [mAU*s]	Height [mAU]	Area %
1	10.571	BB	0.2367	89.15577	5.75641	0.8051
2	11.530	BB	0.2977	505.64423	26.14009	4.5663
3	14.742	MF	0.3837	1.04786e4	455.16248	94.6286

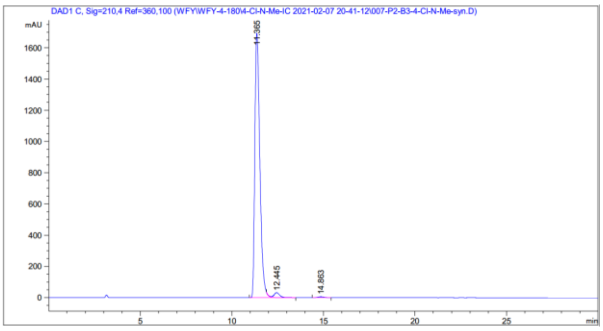

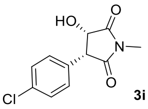

3i

Peak #	RetTime [min]	Type	Width [min]	Area [mAU*s]	Height [mAU]	Area %
1	11.365	BV R	0.2961	3.22368e4	1693.31323	97.4998
2	12.445	VB E	0.3323	697.14661	31.48215	2.1085
3	14.863	BB	0.3262	129.50240	5.67647	0.3917

Supplementary Figure 197. HPLC spectrum of 2i and 3i

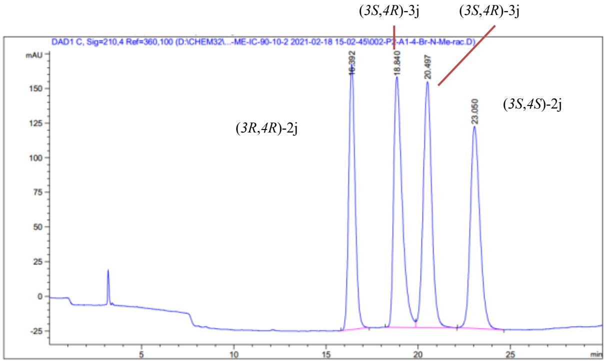

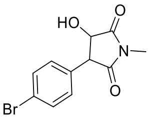

rac-**2j**+*rac*-**3j**

Signal 1: DAD1 C, Sig=210,4 Ref=360,100

Peak #	RetTime [min]	Type	Width [min]	Area [mAU*s]	Height [mAU]	Area %
1	16.392	BB	0.3716	4588.35791	191.46353	21.6205
2	18.840	BV	0.4792	5752.08008	180.77650	27.1040
3	20.497	VB	0.4932	5646.61621	177.47049	26.6070
4	23.050	BB	0.5550	5235.23926	145.77473	24.6686

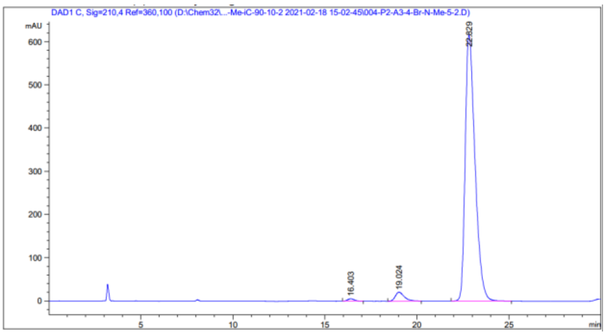

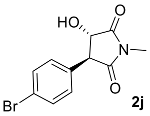

2j

Signal 1: DAD1 C, Sig=210,4 Ref=360,100

Peak #	RetTime [min]	Type	Width [min]	Area [mAU*s]	Height [mAU]	Area %
1	16.403	BB	0.3407	132.53369	5.50749	0.5620
2	19.024	BB	0.5012	680.86475	20.40882	2.8871
3	22.829	BB	0.5673	2.27696e4	615.86407	96.5509

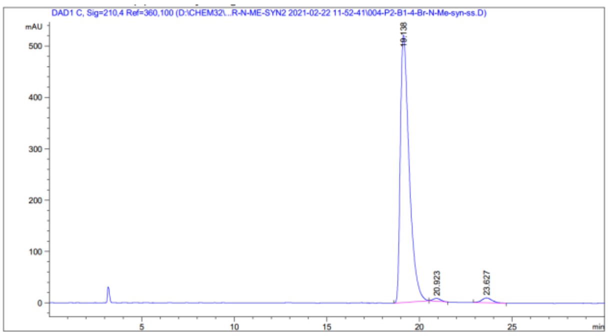

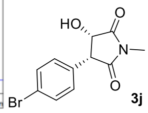

3j

Signal 1: DAD1 C, Sig=210,4 Ref=360,100

Peak #	RetTime [min]	Type	Width [min]	Area [mAU*s]	Height [mAU]	Area %
1	19.138	BB	0.5053	1.72640e4	520.06824	97.2333
2	20.923	BB	0.3951	146.59415	5.29354	0.8256
3	23.627	BB	0.5106	344.63730	9.16796	1.9410

Supplementary Figure 198. HPLC spectrum of 2j and 3j.

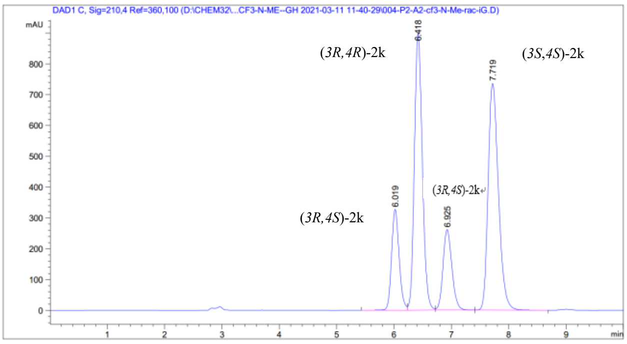

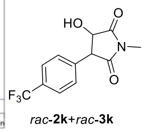

rac-**2k**+*rac*-**3k**

Signal 1: DAD1 C, Sig=210,4 Ref=360,100

Peak #	RetTime [min]	Type	Width [min]	Area [mAU*s]	Height [mAU]	Area %
1	6.019	BV	0.1401	2970.35352	326.76434	12.6280
2	6.418	VV	0.1500	8608.76855	897.13721	36.5988
3	6.925	VB	0.1675	2806.93872	261.03534	11.9333
4	7.719	BB	0.1909	9135.92383	735.73059	38.8399

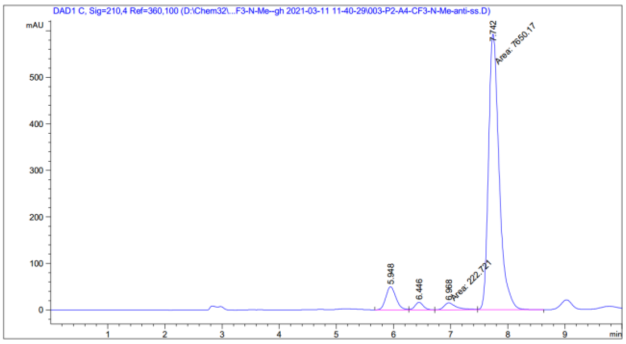

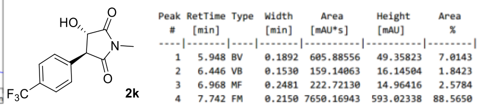

2k

Signal 1: DAD1 C, Sig=210,4 Ref=360,100

Peak #	RetTime [min]	Type	Width [min]	Area [mAU*s]	Height [mAU]	Area %
1	5.948	BV	0.1892	605.88556	49.35823	7.0143
2	6.446	VB	0.1530	159.14063	16.14504	1.8423
3	6.968	MF	0.2481	222.72130	14.96416	2.5784
4	7.742	FM	0.2150	7650.16943	593.02338	88.5650

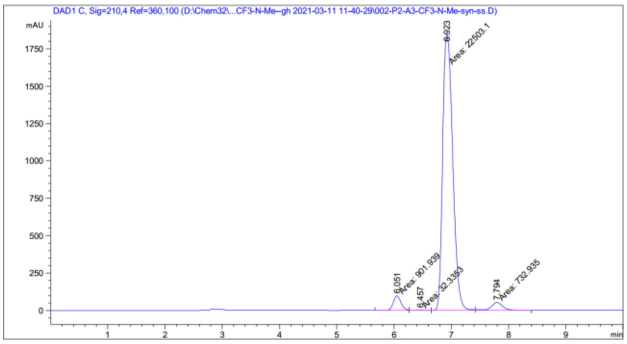

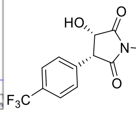

3k

Signal 1: DAD1 C, Sig=210,4 Ref=360,100

Peak #	RetTime [min]	Type	Width [min]	Area [mAU*s]	Height [mAU]	Area %
1	6.051	MF	0.1560	901.93884	96.37147	3.7316
2	6.457	FM	0.1993	32.33525	2.70420	0.1338
3	6.923	MF	0.2029	2.25031e4	1848.74438	93.1023
4	7.794	FM	0.2331	732.93524	52.40259	3.0324

Supplementary Figure 199. HPLC spectrum of 2k and 3k.

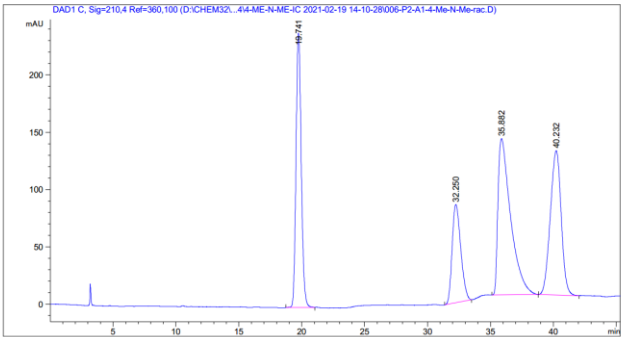

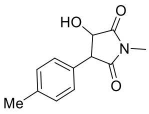

rac-**2l**+*rac*-**3l**

Signal 1: DAD1 C, Sig=210,4 Ref=360,100

Peak #	RetTime [min]	Type	Width [min]	Area [mAU*s]	Height [mAU]	Area %
1	19.741	BB	0.4493	6928.21582	239.63028	24.6366
2	32.250	BB	0.7241	4087.59448	85.75805	14.5354
3	35.882	BB	1.0054	9543.21191	136.54620	33.9355
4	40.232	BB	0.9185	7562.58447	125.95538	26.8924

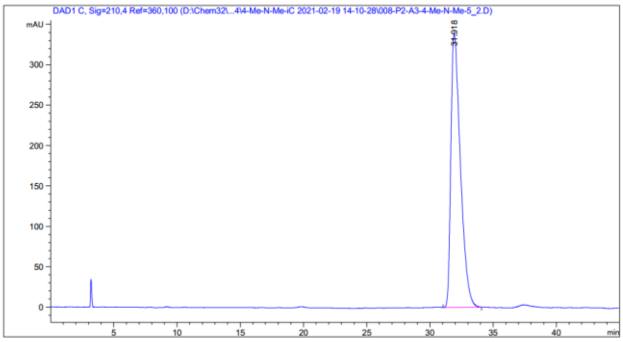

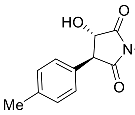

2l

Signal 1: DAD1 C, Sig=210,4 Ref=360,100

Peak #	RetTime [min]	Type	Width [min]	Area [mAU*s]	Height [mAU]	Area %
1	31.918	BB	0.7894	1.77385e4	338.49649	100.0000

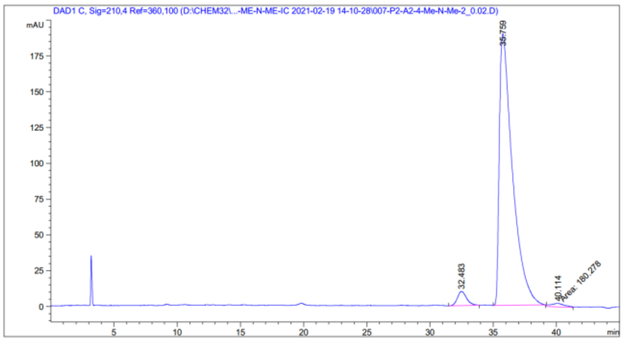

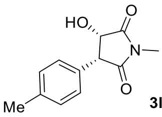

3l

Signal 1: DAD1 C, Sig=210,4 Ref=360,100

Peak #	RetTime [min]	Type	Width [min]	Area [mAU*s]	Height [mAU]	Area %
1	32.483	BB	0.6415	500.82480	9.91092	3.4821
2	35.759	BB	1.0350	1.37018e4	189.61919	95.2645
3	40.114	MM	1.2753	180.27843	2.35594	1.2534

Supplementary Figure 200. HPLC spectrum of 2l and 3l.

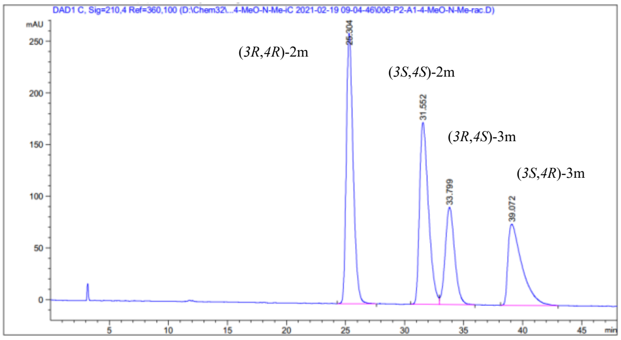

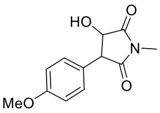

rac-**2m**+*rac*-3**m**

Signal 1: DAD1 C, Sig=210,4 Ref=360,100

Peak #	RetTime [min]	Type	Width [min]	Area [mAU*s]	Height [mAU]	Area %
1	25.304	BB	0.6159	1.05319e4	261.49316	33.4298
2	31.552	BV	0.7933	9276.45801	175.89235	29.4449
3	33.799	VB	0.8337	5208.19629	94.09803	16.5316
4	39.072	BB	1.1453	6487.91650	78.41243	20.5937

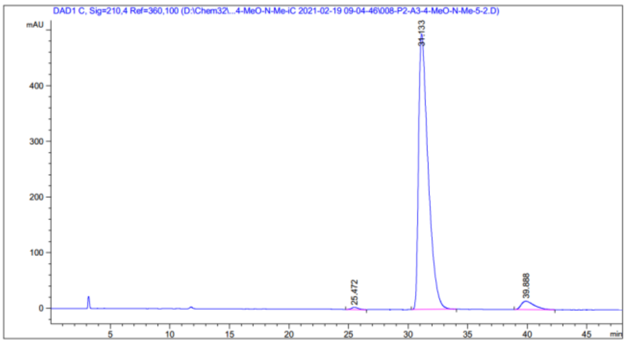

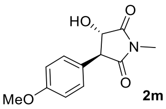

2m

Signal 1: DAD1 C, Sig=210,4 Ref=360,100

Peak #	RetTime [min]	Type	Width [min]	Area [mAU*s]	Height [mAU]	Area %
1	25.472	BB	0.5177	171.33064	4.04908	0.5935
2	31.133	BB	0.8298	2.75475e4	494.56934	95.4329
3	39.888	BB	0.9186	1147.01611	14.95925	3.9736

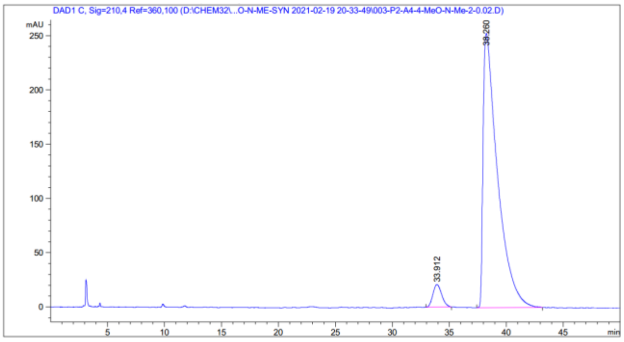

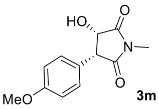

3m

Signal 1: DAD1 C, Sig=210,4 Ref=360,100

Peak #	RetTime [min]	Type	Width [min]	Area [mAU*s]	Height [mAU]	Area %
1	33.912	BB	0.7588	1110.34351	20.73287	4.7663
2	38.260	BB	1.1927	2.21854e4	252.59190	95.2337

Supplementary Figure 201. HPLC spectrum of 2m and 3m.

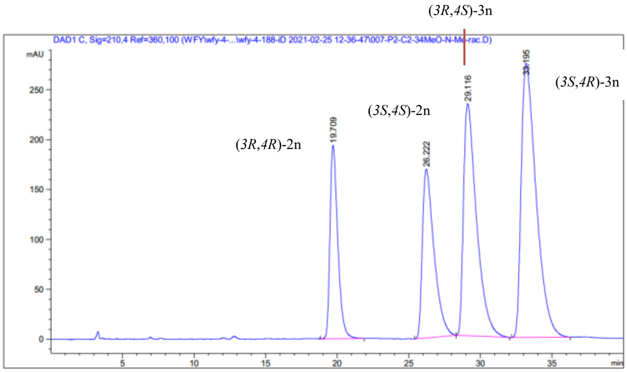

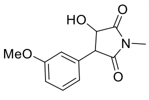

rac-**2n**+*rac*-**3n**

Signal 1: DAD1 C, Sig=210,4 Ref=360,100

Peak #	RetTime [min]	Type	Width [min]	Area [mAU*s]	Height [mAU]	Area %
1	19.709	BB	0.5743	7308.38330	193.66742	14.1761
2	26.222	BB	0.8425	9568.07520	168.99763	18.5592
3	29.116	BB	0.9184	1.47688e4	232.71100	28.6469
4	33.195	BB	1.0440	1.99092e4	274.56754	38.6178

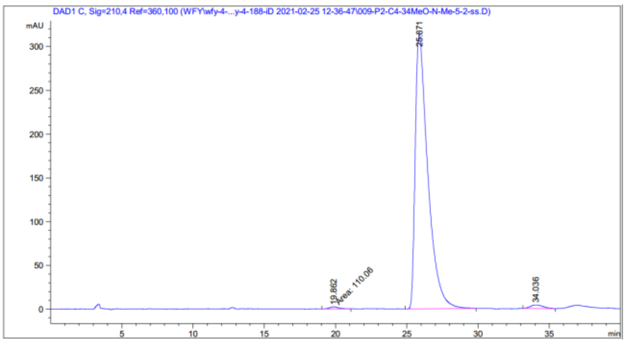

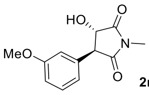

2n

Signal 1: DAD1 C, Sig=210,4 Ref=360,100

Peak #	RetTime [min]	Type	Width [min]	Area [mAU*s]	Height [mAU]	Area %
1	19.862	MM	0.8110	110.05970	2.26184	0.5586
2	25.871	BB	0.8835	1.93373e4	313.55331	98.1416
3	34.036	BB	0.7466	256.11478	4.04574	1.2998

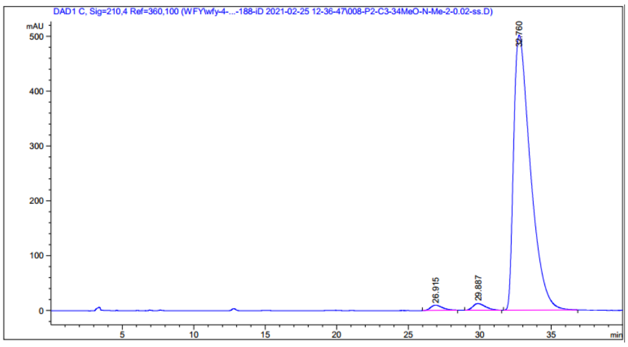

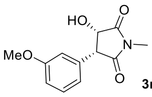

3n

Signal 1: DAD1 C, Sig=210,4 Ref=360,100

Peak #	RetTime [min]	Type	Width [min]	Area [mAU*s]	Height [mAU]	Area %
1	26.915	BB	0.6853	553.32019	9.58747	1.3107
2	29.887	BB	0.7322	753.38367	12.27248	1.7846
3	32.760	BB	1.1953	4.09095e4	501.68649	96.9047

Supplementary Figure 202. HPLC spectrum of 2n and 3n.

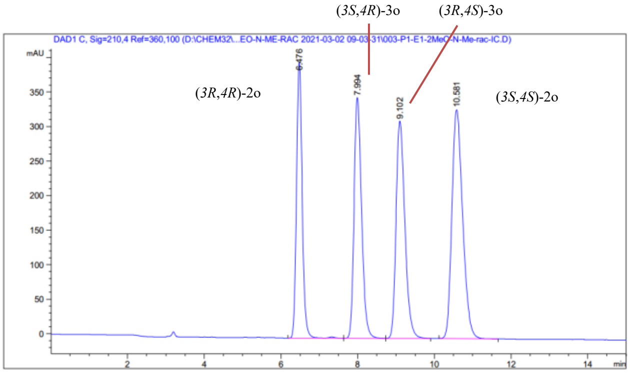

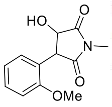

rac-**2o**+*rac*-**3o**

Signal 1: DAD1 C, Sig=210,4 Ref=360,100

Peak #	RetTime [min]	Type	Width [min]	Area [mAU*s]	Height [mAU]	Area %
1	6.476	BV R	0.1575	4104.71973	398.97318	20.2201
2	7.994	BB	0.2086	4735.72754	348.37698	23.3285
3	9.102	BB	0.2457	5005.52051	314.42807	24.6575
4	10.581	BB	0.3012	6454.23438	331.36960	31.7939

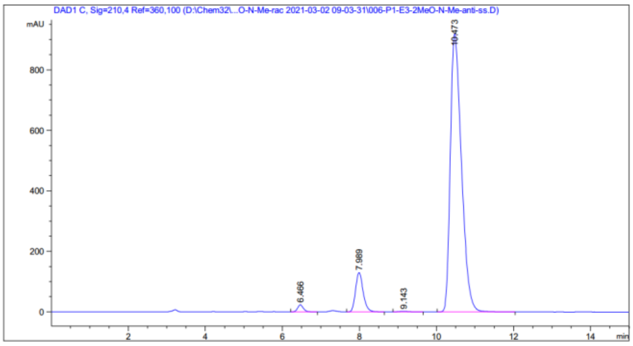

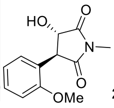

2o

Signal 1: DAD1 C, Sig=210,4 Ref=360,100

Peak #	RetTime [min]	Type	Width [min]	Area [mAU*s]	Height [mAU]	Area %
1	6.466	BB	0.1622	246.28621	23.51783	1.2044
2	7.989	BB	0.2120	1780.91016	129.83890	8.7092
3	9.143	BB	0.2386	30.74656	1.90179	0.1504
4	10.473	BB	0.3092	1.83906e4	919.90302	89.9360

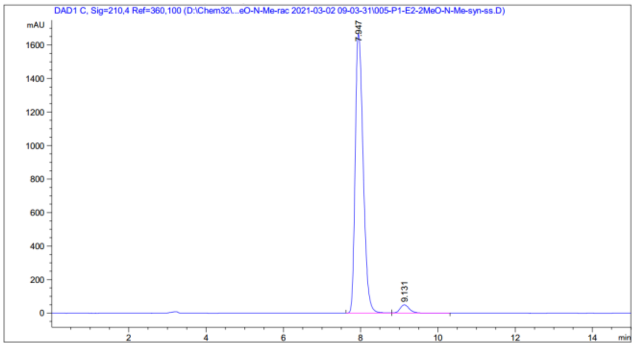

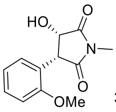

3o

Signal 1: DAD1 C, Sig=210,4 Ref=360,100

Peak #	RetTime [min]	Type	Width [min]	Area [mAU*s]	Height [mAU]	Area %
1	7.947	BV	0.2263	2.40293e4	1665.76819	96.6246
2	9.131	VB	0.2608	839.42590	49.25992	3.3754

Supplementary Figure 203. HPLC spectrum of 2o and 3o.

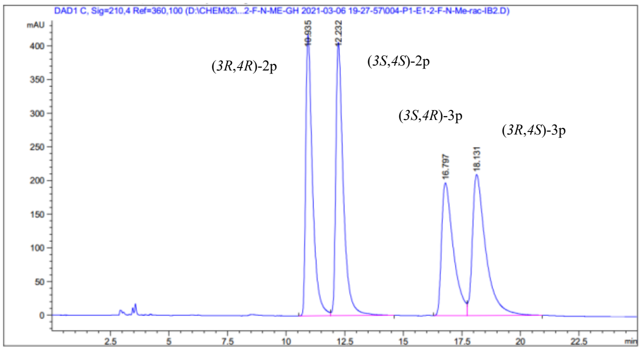

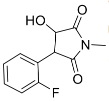

rac-**2p**+rac-**3p**

Signal 1: DAD1 C, Sig=210,4 Ref=360,100

Peak #	RetTime [min]	Type	Width [min]	Area [mAU*s]	Height [mAU]	Area %
1	10.935	BV	0.3030	8492.67383	418.03378	25.9192
2	12.232	VB	0.3299	9044.73438	406.01266	27.6040
3	16.797	BV	0.5196	6896.23730	197.39912	21.0469
4	18.131	VB	0.5740	8332.35547	209.49240	25.4299

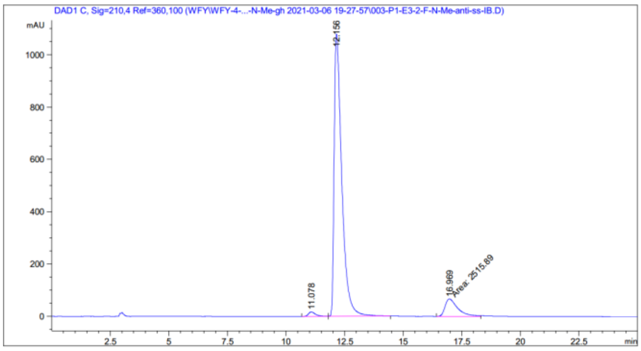

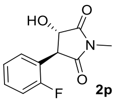

2p

Signal 1: DAD1 C, Sig=210,4 Ref=360,100

Peak #	RetTime [min]	Type	Width [min]	Area [mAU*s]	Height [mAU]	Area %
1	11.078	BB	0.3124	358.33875	17.10595	1.3144
2	12.156	BB	0.3379	2.43881e4	1078.32605	89.4571
3	16.969	MF	0.6278	2515.89087	66.79250	9.2285

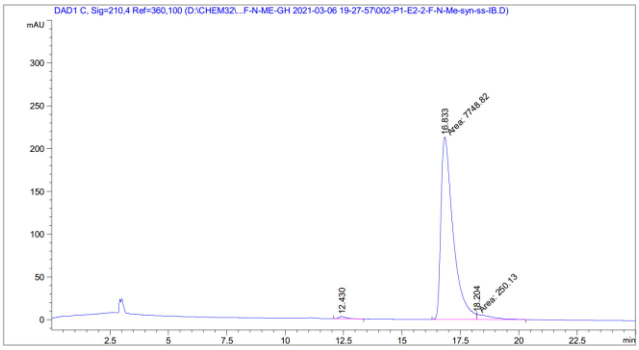

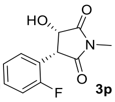

3p

Signal 1: DAD1 C, Sig=210,4 Ref=360,100

Peak #	RetTime [min]	Type	Width [min]	Area [mAU*s]	Height [mAU]	Area %
1	12.430	BB	0.3276	63.29460	2.64087	0.7851
2	16.833	MF	0.6058	7748.81641	213.19093	96.1124
3	18.204	FM	0.6808	250.12999	6.12376	3.1025

Supplementary Figure 204. HPLC spectrum of 2p and 3p.

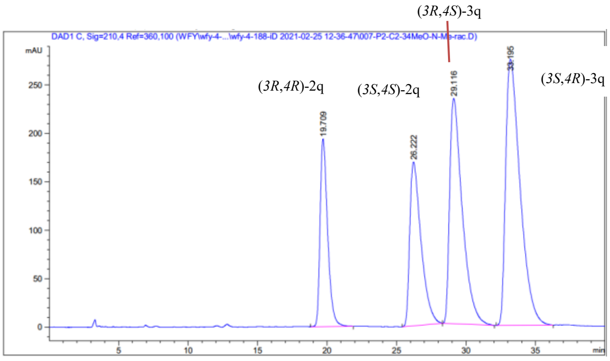

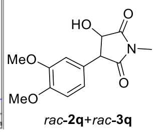

rac-**2q**+*rac*-**3q**

Signal 1: DAD1 C, Sig=210,4 Ref=360,100

Peak #	RetTime [min]	Type	Width [min]	Area [mAU*s]	Height [mAU]	Area %
1	19.709	BB	0.5743	7308.38330	193.66742	14.1761
2	26.222	BB	0.8425	9568.07520	168.99763	18.5592
3	29.116	BB	0.9184	1.47688e4	232.71100	28.6469
4	33.195	BB	1.0440	1.99092e4	274.56754	38.6178

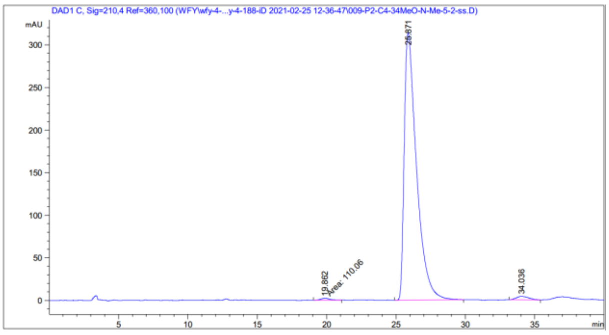

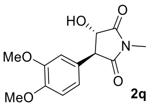

2q

Signal 1: DAD1 C, Sig=210,4 Ref=360,100

Peak #	RetTime [min]	Type	Width [min]	Area [mAU*s]	Height [mAU]	Area %
1	19.862	MM	0.8110	110.05970	2.26184	0.5586
2	25.871	BB	0.8835	1.93373e4	313.55331	98.1416
3	34.036	BB	0.7466	256.11478	4.04574	1.2998

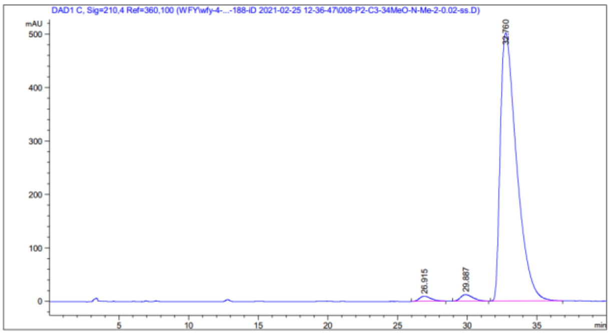

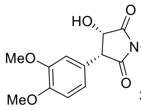

3q

Signal 1: DAD1 C, Sig=210,4 Ref=360,100

Peak #	RetTime [min]	Type	Width [min]	Area [mAU*s]	Height [mAU]	Area %
1	26.915	BB	0.6853	553.32019	9.58747	1.3107
2	29.887	BB	0.7322	753.38367	12.27248	1.7846
3	32.760	BB	1.1953	4.09095e4	501.68649	96.9047

Supplementary Figure 205. HPLC spectrum of 2q and 3q.

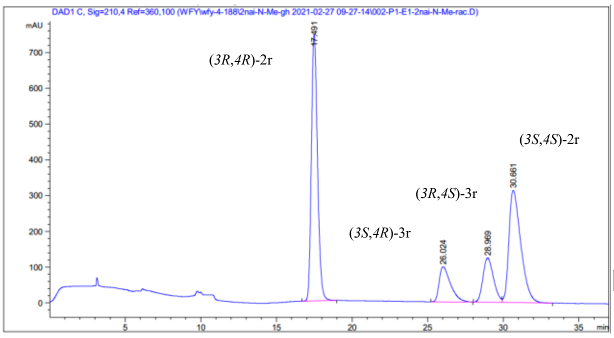

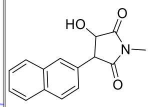

rac-**2r**+*rac*-**3r**

Signal 1: DAD1 C, Sig=210,4 Ref=360,100

Peak #	RetTime [min]	Type	Width [min]	Area [mAU*s]	Height [mAU]	Area %
1	17.491	BB	0.4331	2.08761e4	744.58783	42.4990
2	26.024	BB	0.7581	5086.75732	97.92147	10.3555
3	28.969	BV	0.7400	5979.66016	123.69154	12.1732
4	30.661	VB	0.8287	1.71789e4	313.77274	34.9723

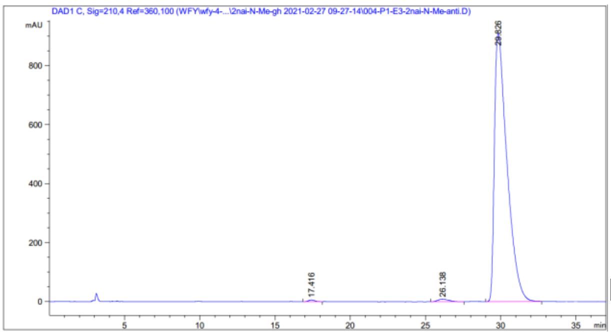

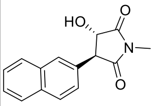

2r

Signal 1: DAD1 C, Sig=210,4 Ref=360,100

Peak #	RetTime [min]	Type	Width [min]	Area [mAU*s]	Height [mAU]	Area %
1	17.416	BB	0.4235	165.65111	5.82996	0.3105
2	26.138	BB	0.6428	445.10355	8.47381	0.8343
3	29.826	BB	0.8412	5.27394e4	908.53595	98.8552

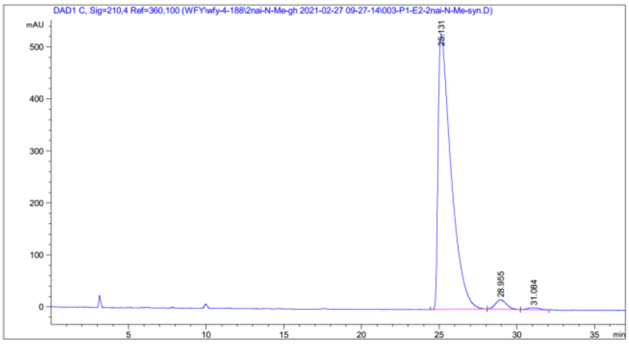

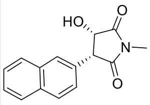

3r

Signal 1: DAD1 C, Sig=210,4 Ref=360,100

Peak #	RetTime [min]	Type	Width [min]	Area [mAU*s]	Height [mAU]	Area %
1	25.131	BB	0.8239	3.07379e4	530.58673	96.6195
2	28.955	BB	0.7023	879.85114	18.18976	2.7657
3	31.084	BB	0.6027	195.59460	3.86285	0.6148

Supplementary Figure 206. HPLC spectrum of 2r and 3r.

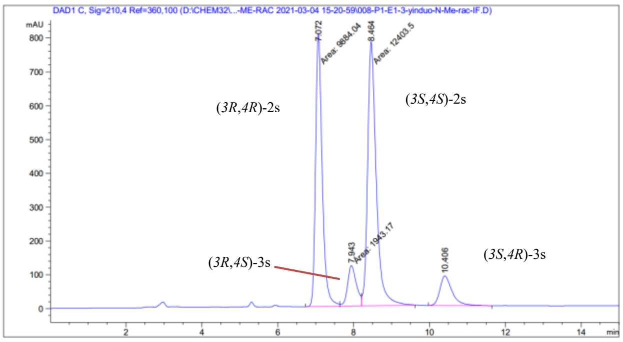

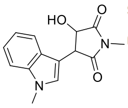

rac-**2s**+*rac*-**3s**

Signal 1: DAD1 C, Sig=210,4 Ref=360,100

Peak #	RetTime [min]	Type	Width [min]	Area [mAU*s]	Height [mAU]	Area %
1	7.072	MF	0.2044	9884.04297	806.08612	37.7272
2	7.943	MF	0.2705	1943.17285	119.70770	7.4170
3	8.464	FM	0.2648	1.24035e4	780.65063	47.3441
4	10.406	BB	0.3388	1967.98120	87.37034	7.5117

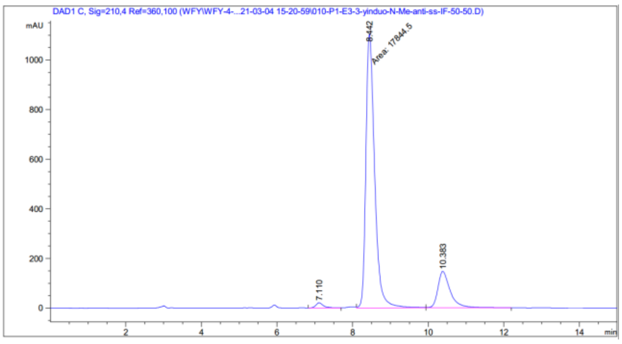

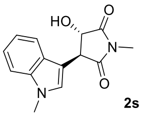

2s

Signal 1: DAD1 C, Sig=210,4 Ref=360,100

Peak #	RetTime [min]	Type	Width [min]	Area [mAU*s]	Height [mAU]	Area %
1	7.110	BV	0.2085	289.48233	20.78885	1.3481
2	8.442	FM	0.2697	1.78445e4	1102.92102	83.1023
3	10.383	VB	0.3389	3338.93091	147.02534	15.5495

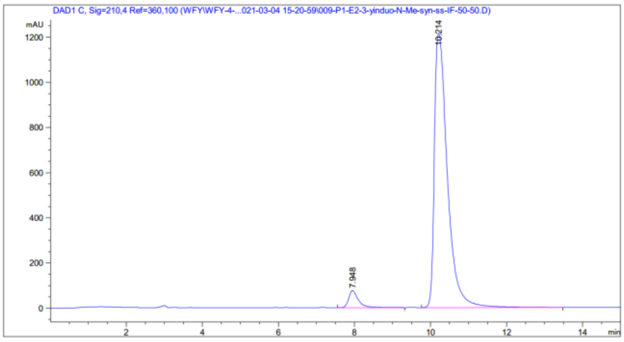

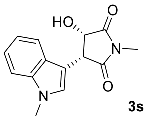

3s

Signal 1: DAD1 C, Sig=210,4 Ref=360,100

Peak #	RetTime [min]	Type	Width [min]	Area [mAU*s]	Height [mAU]	Area %
1	7.948	BB	0.2653	1363.16968	75.99920	4.5214
2	10.214	BB	0.3626	2.87858e4	1214.10669	95.4786

Supplementary Figure 207. HPLC spectrum of 2s and 3s.

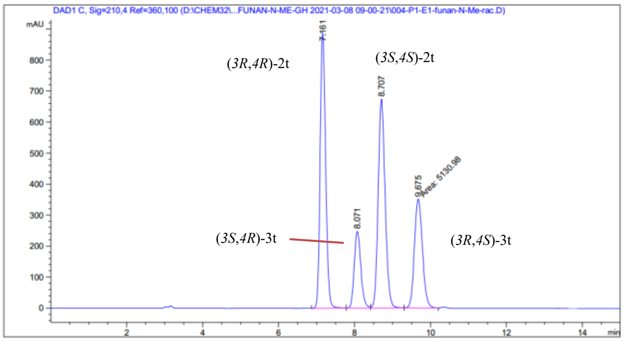

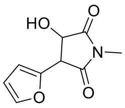

rac-**2t**+*rac*-**3t**

Signal 1: DAD1 C, Sig=210,4 Ref=360,100

Peak #	RetTime [min]	Type	Width [min]	Area [mAU*s]	Height [mAU]	Area %
1	7.161	BV	0.1550	8881.64063	885.60522	34.7293
2	8.071	VV	0.1839	2965.23242	247.20732	11.5948
3	8.707	VB	0.1988	8596.03516	673.79553	33.6125
4	9.675	MF	0.2436	5130.98389	351.11282	20.0634

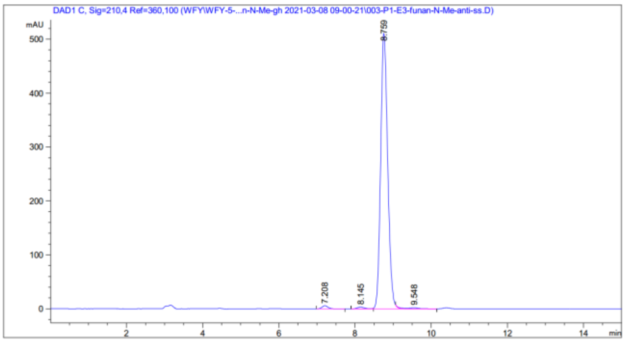

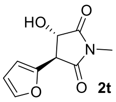

2t

Signal 1: DAD1 C, Sig=210,4 Ref=360,100

Peak #	RetTime [min]	Type	Width [min]	Area [mAU*s]	Height [mAU]	Area %
1	7.208	BB	0.1652	62.69970	5.84176	0.9297
2	8.145	BV E	0.1984	42.96050	3.33190	0.6370
3	8.759	VV R	0.2006	6592.96826	510.67633	97.7551
4	9.548	VB E	0.2976	45.74754	2.05584	0.6783

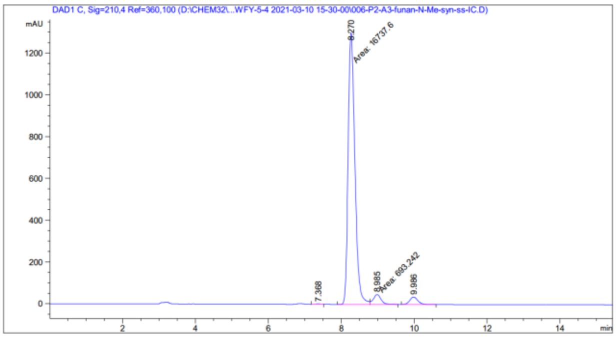

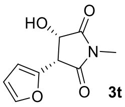

3t

Signal 1: DAD1 C, Sig=210,4 Ref=360,100

Peak #	RetTime [min]	Type	Width [min]	Area [mAU*s]	Height [mAU]	Area %
1	7.368	BB	0.1382	11.15477	1.32484	0.0620
2	8.270	MF	0.2146	1.67376e4	1299.90833	93.0579
3	8.985	FM	0.2455	693.24231	47.05786	3.8543
4	9.986	VB	0.2381	544.22198	35.25238	3.0258

Supplementary Figure 208. HPLC spectrum of 2t and 3t.

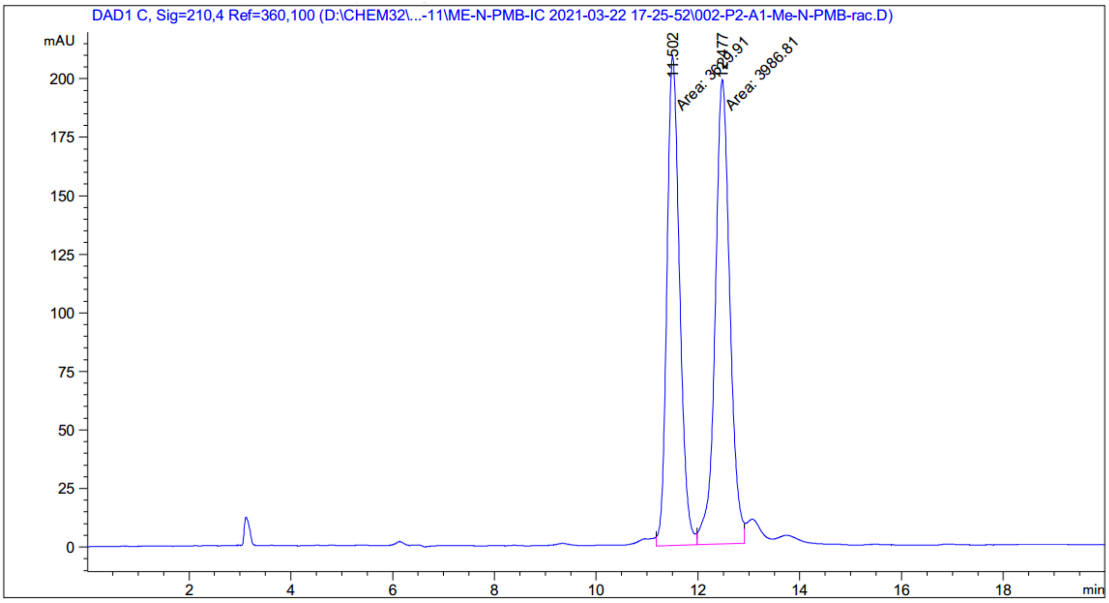

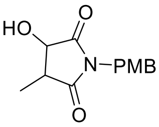

rac-**3u**

Signal 1: DAD1 C, Sig=210,4 Ref=360,100

Peak #	RetTime [min]	Type	Width [min]	Area [mAU*s]	Height [mAU]	Area %
1	11.502	FM	0.2896	3629.90869	208.91261	47.6571
2	12.477	FM	0.3349	3986.80957	198.41626	52.3429

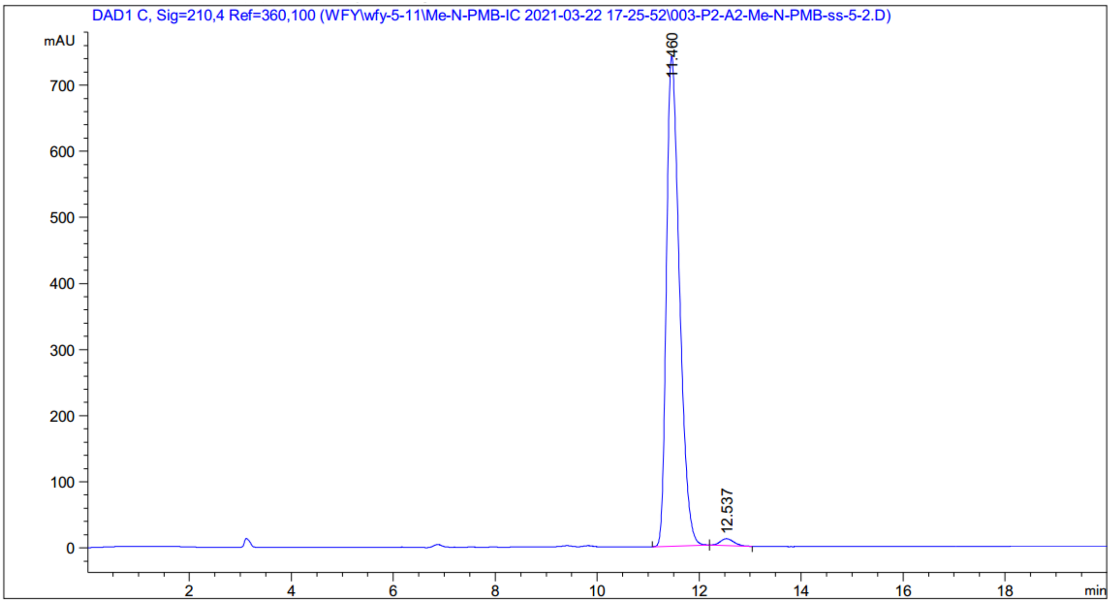

Signal 1: DAD1 C, Sig=210,4 Ref=360,100

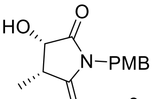

3u

Peak #	RetTime [min]	Type	Width [min]	Area [mAU*s]	Height [mAU]	Area %
1	11.460	BB	0.2751	1.31536e4	740.95862	98.6124
2	12.537	BB	0.2779	185.08482	10.18947	1.3876

Supplementary Figure 209. HPLC spectrum of 3u.

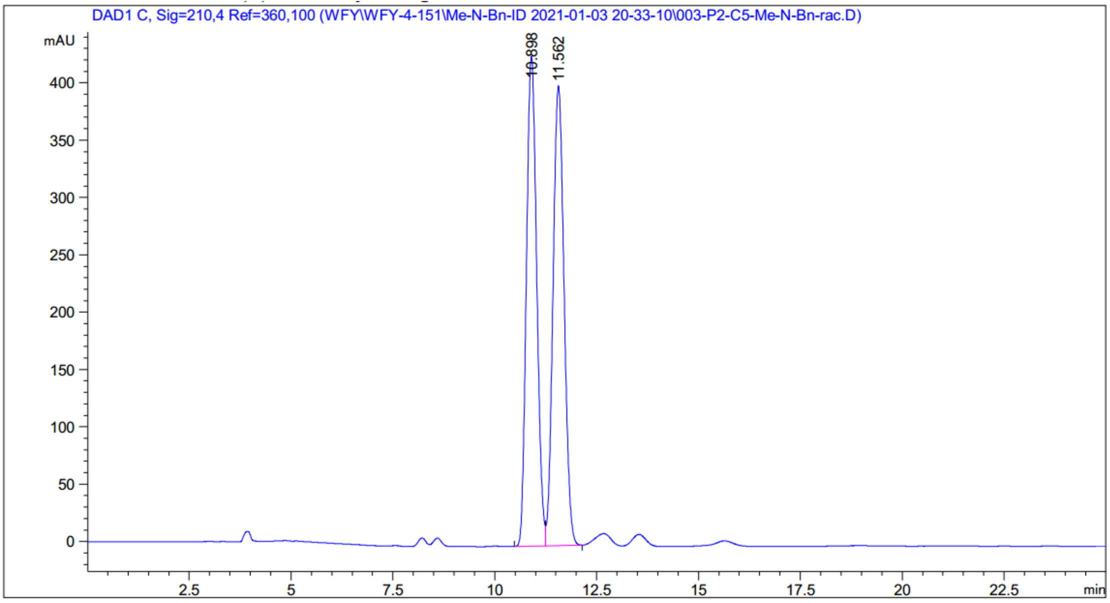

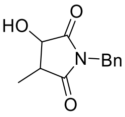

rac-**3v**

Signal 1: DAD1 C, Sig=210,4 Ref=360,100

Peak #	RetTime [min]	Type	Width [min]	Area [mAU*s]	Height [mAU]	Area %
1	10.898	BV	0.2761	7471.90088	427.15399	49.7945
2	11.562	VB	0.2972	7533.57080	400.96533	50.2055

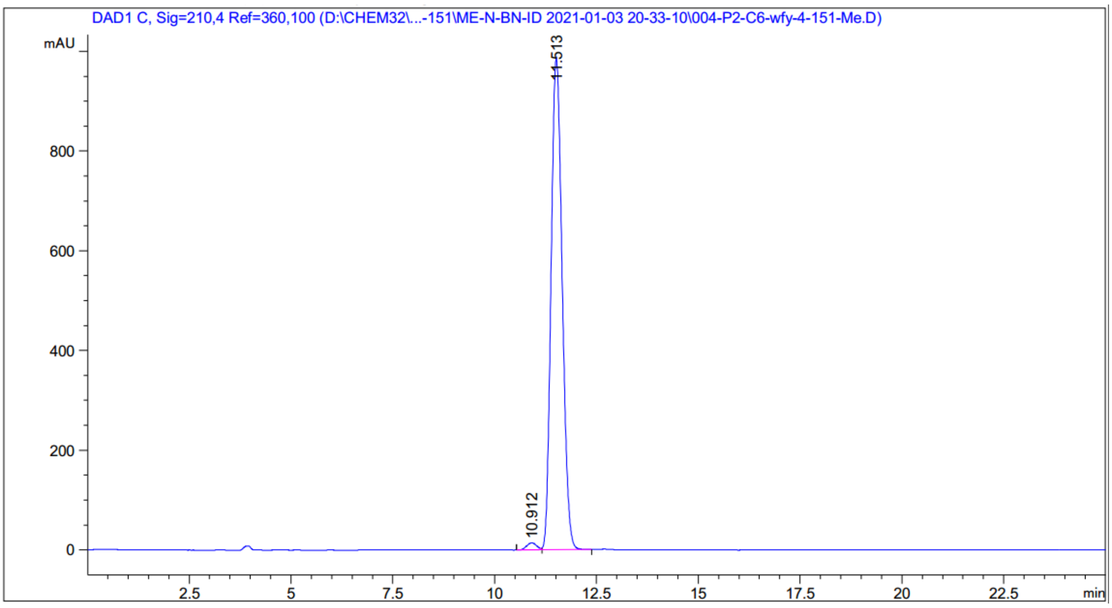

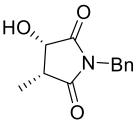

3v

Signal 1: DAD1 C, Sig=210,4 Ref=360,100

Peak #	RetTime [min]	Type	Width [min]	Area [mAU*s]	Height [mAU]	Area %
1	10.912	BV E	0.2706	240.05655	14.24430	1.2737
2	11.513	VB R	0.2984	1.86064e4	984.71478	98.7263

Supplementary Figure 210. HPLC spectrum of 3v.

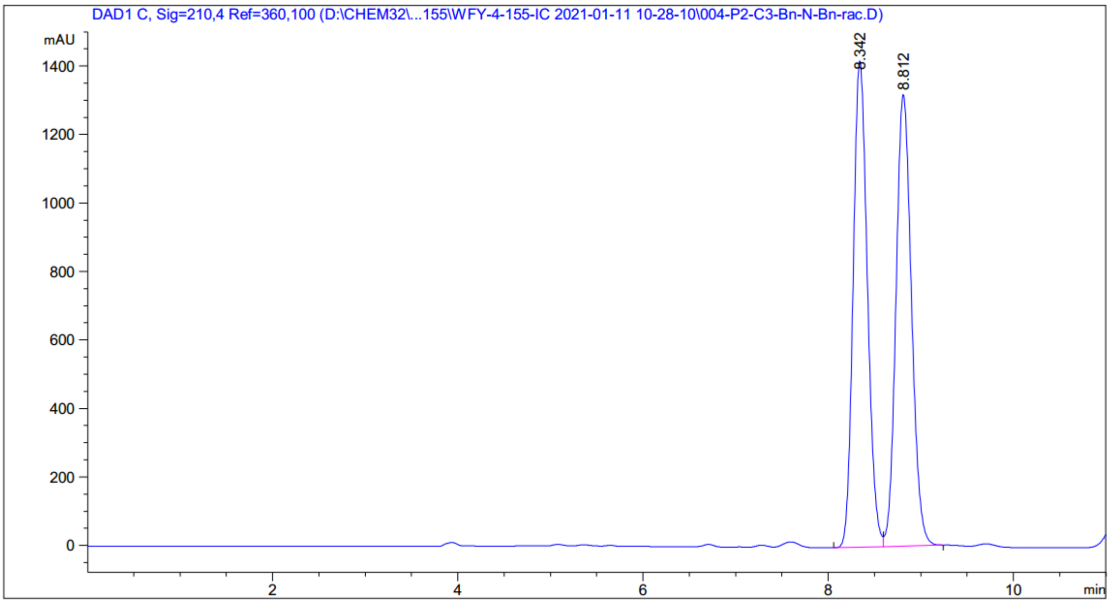

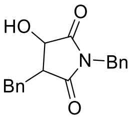

rac-**3w**

Signal 1: DAD1 C, Sig=210,4 Ref=360,100

Peak #	RetTime [min]	Type	Width [min]	Area [mAU*s]	Height [mAU]	Area %
1	8.342	BV	0.1663	1.51184e4	1419.24426	49.7048
2	8.812	VB	0.1794	1.52980e4	1318.59619	50.2952

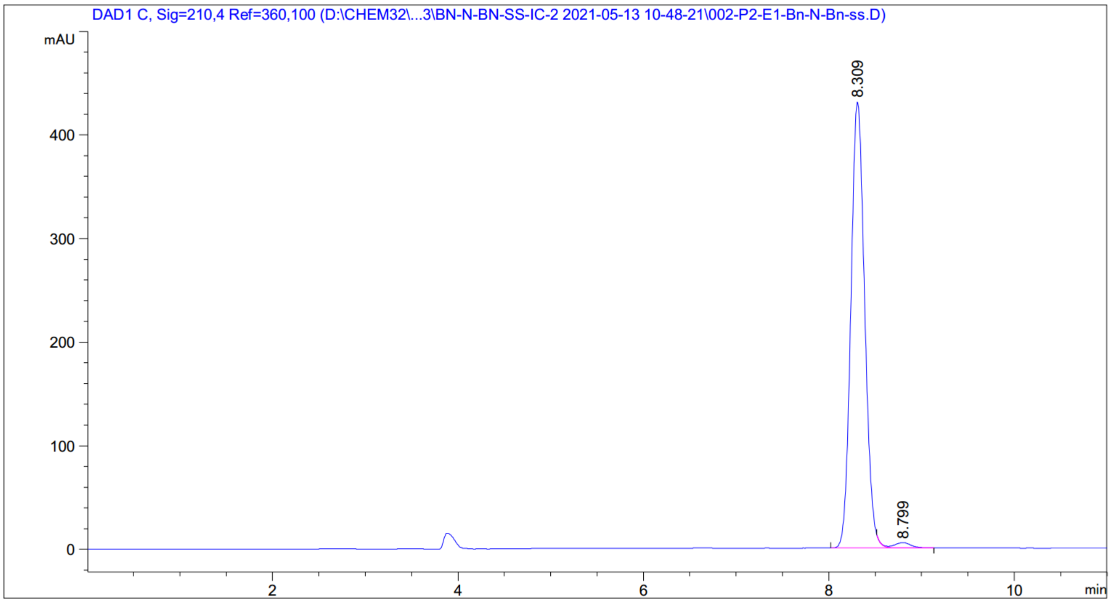

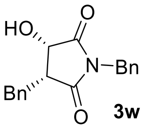

3w

Signal 1: DAD1 C, Sig=210,4 Ref=360,100

Peak #	RetTime [min]	Type	Width [min]	Area [mAU*s]	Height [mAU]	Area %
1	8.309	BV R	0.1607	4452.74609	430.53940	98.5531
2	8.799	VB E	0.1870	65.37309	5.18871	1.4469

Supplementary Figure 211. HPLC spectrum of 3w.

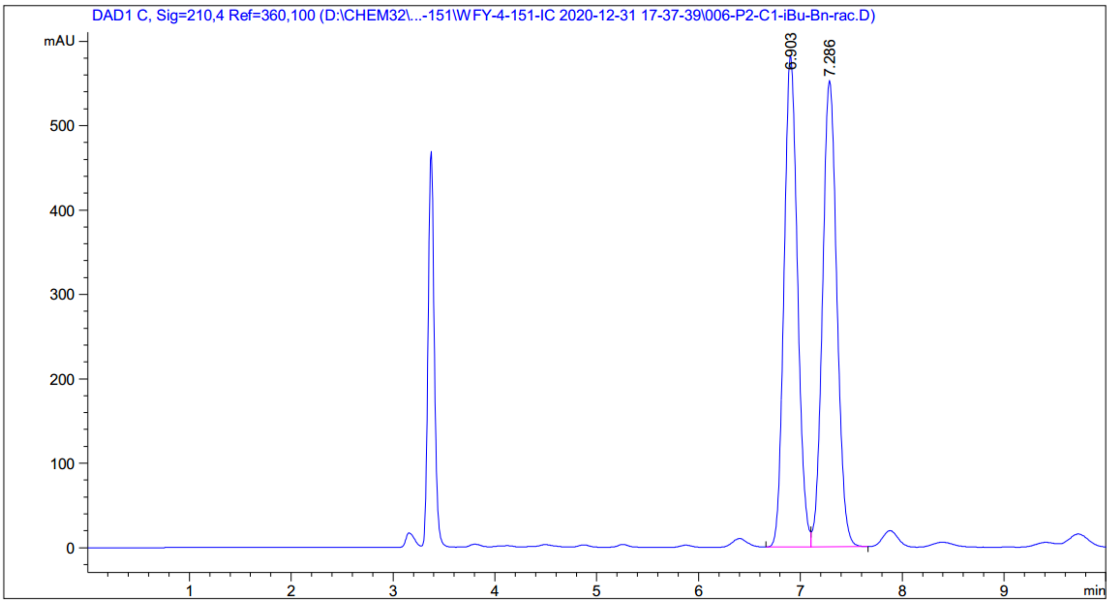

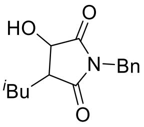

rac-**3x**

Signal 1: DAD1 C, Sig=210,4 Ref=360,100

Peak #	RetTime [min]	Type	Width [min]	Area [mAU*s]	Height [mAU]	Area %
1	6.903	BV	0.1432	5344.88232	581.60333	49.7163
2	7.286	VB	0.1523	5405.89160	552.01306	50.2837

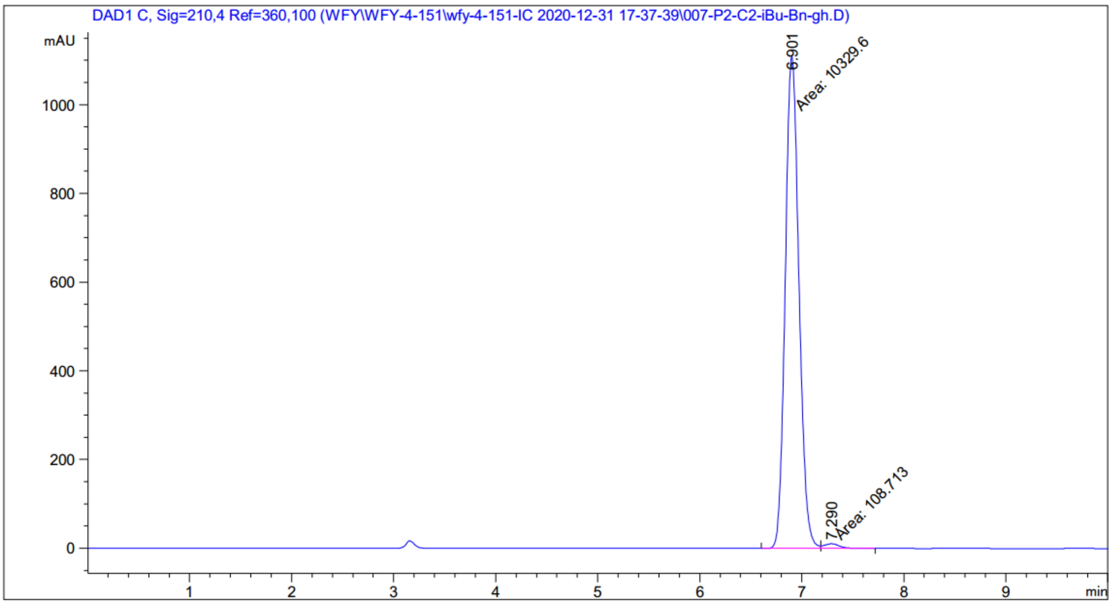

Signal 1: DAD1 C, Sig=210,4 Ref=360,100

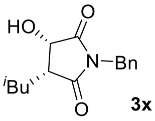

3x

Peak #	RetTime [min]	Type	Width [min]	Area [mAU*s]	Height [mAU]	Area %
1	6.901	MF	0.1552	1.03296e4	1109.61743	98.9585
2	7.290	FM	0.1744	108.71309	10.38903	1.0415

Supplementary Figure 212. HPLC spectrum of 3x.

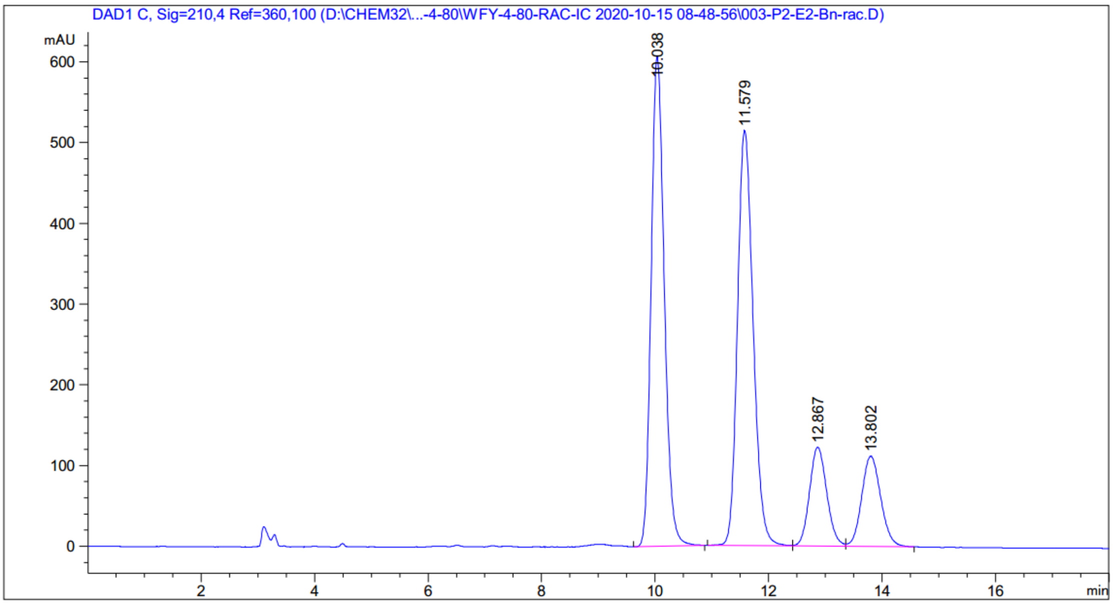

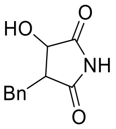

rac-**3y**

Signal 1: DAD1 C, Sig=210,4 Ref=360,100

Peak #	RetTime [min]	Type	Width [min]	Area [mAU*s]	Height [mAU]	Area %
1	10.038	BB	0.2495	9757.09473	606.97229	39.3258
2	11.579	BV	0.2951	9836.90430	514.34143	39.6475
3	12.867	VV	0.3268	2594.47729	122.66529	10.4570
4	13.802	VB	0.3651	2622.45117	112.04375	10.5697

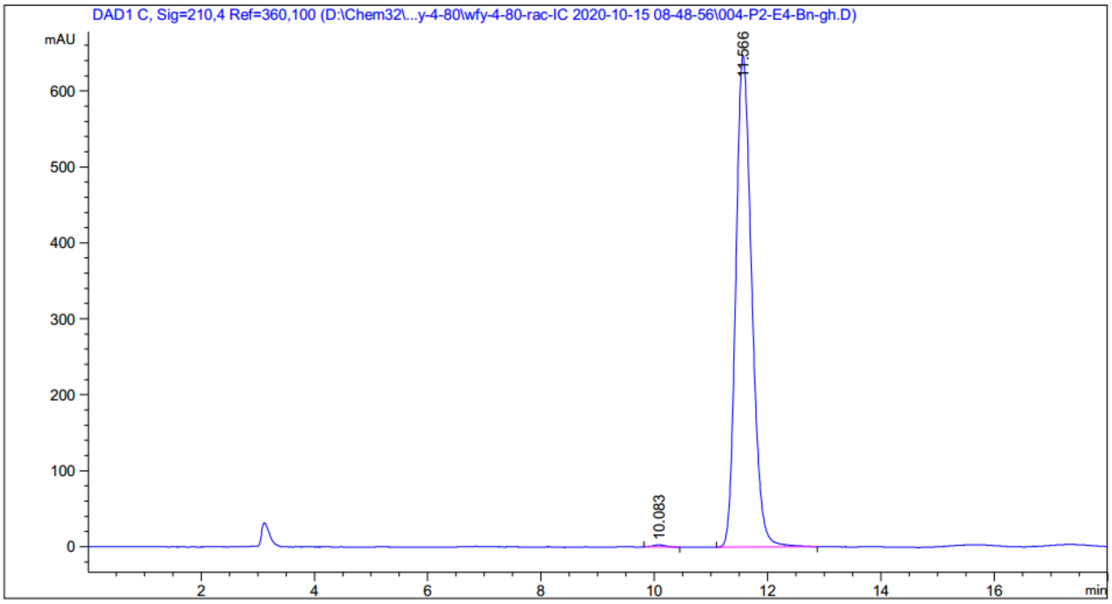

Signal 1: DAD1 C, Sig=210,4 Ref=360,100

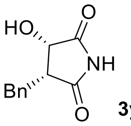

3y

Peak #	RetTime [min]	Type	Width [min]	Area [mAU*s]	Height [mAU]	Area %
1	10.083	BB	0.1970	43.18199	2.81434	0.3412
2	11.566	BB	0.3037	1.26144e4	646.24652	99.6588

Supplementary Figure 213. HPLC spectrum of 3y.

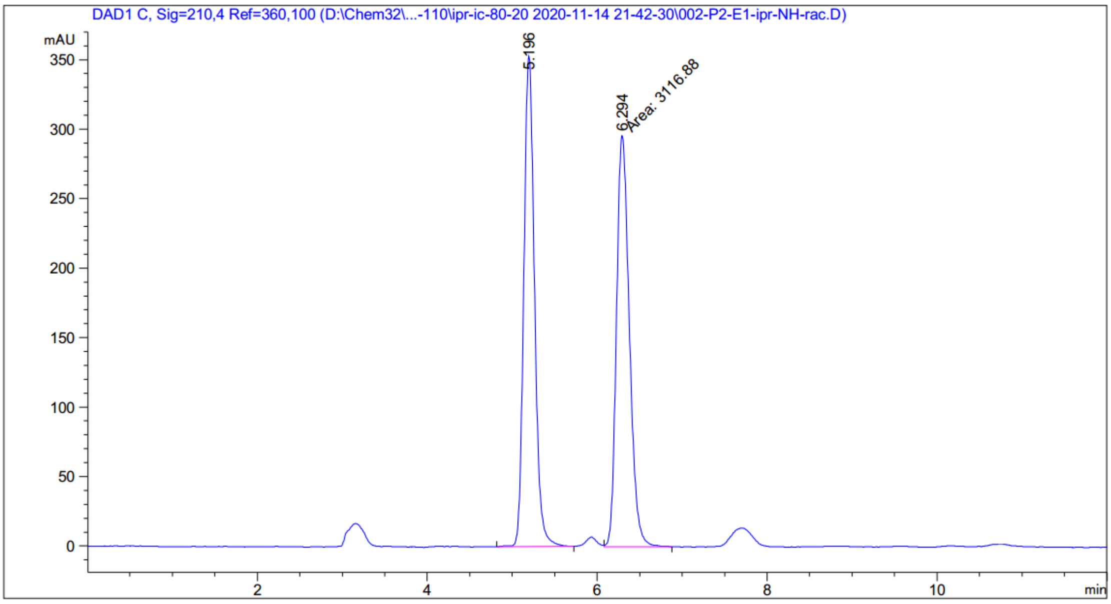

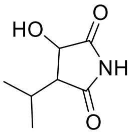

rac-**3z**

Signal 1: DAD1 C, Sig=210,4 Ref=360,100

Peak #	RetTime [min]	Type	Width [min]	Area [mAU*s]	Height [mAU]	Area %
1	5.196	VB R	0.1359	3083.83691	352.90137	49.7335
2	6.294	FM	0.1754	3116.88159	296.11624	50.2665

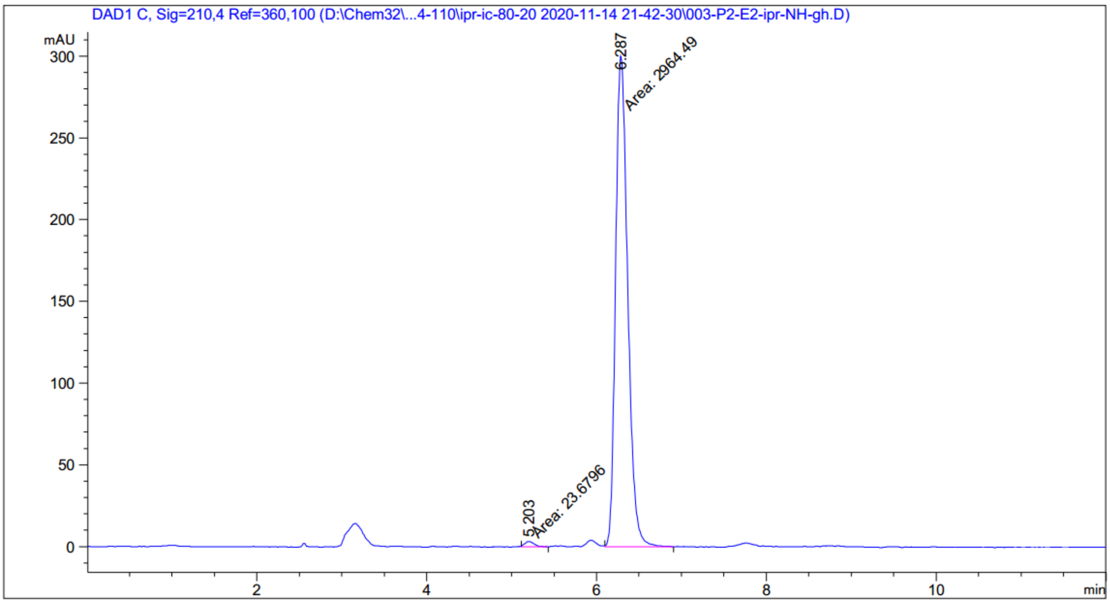

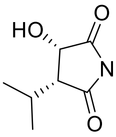

3z

Signal 1: DAD1 C, Sig=210,4 Ref=360,100

Peak #	RetTime [min]	Type	Width [min]	Area [mAU*s]	Height [mAU]	Area %
1	5.203	MM	0.1247	23.67964	3.16456	0.7924
2	6.287	FM	0.1646	2964.48584	300.07996	99.2076

Supplementary Figure 214. HPLC spectrum of 3z.

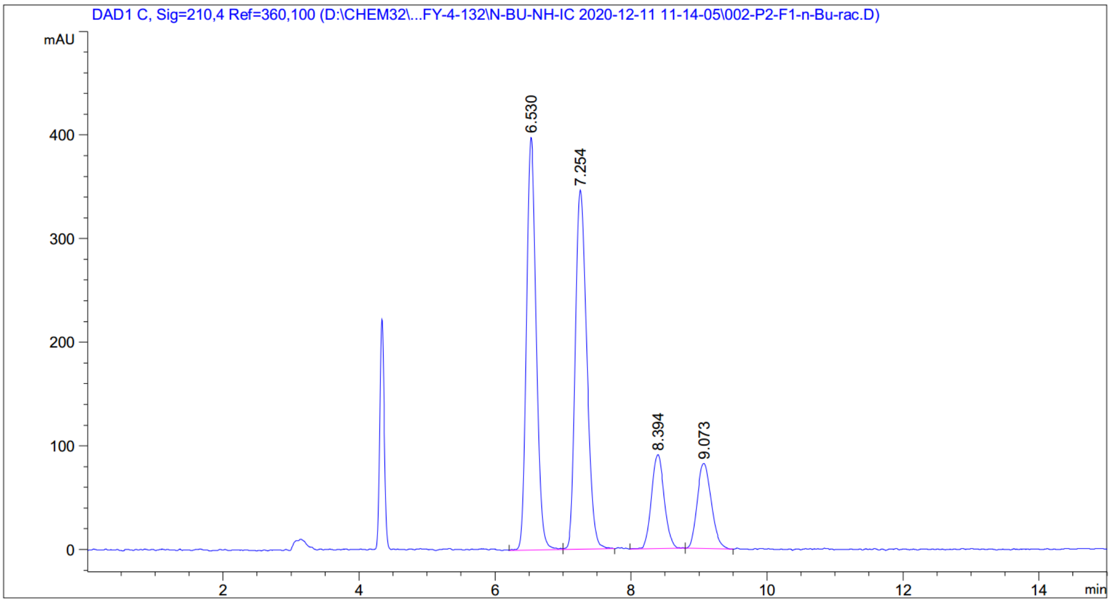

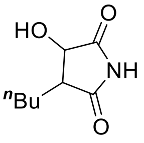

rac-**3aa**

Signal 1: DAD1 C, Sig=210,4 Ref=360,100

Peak #	RetTime [min]	Type	Width [min]	Area [mAU*s]	Height [mAU]	Area %
1	6.530	VV R	0.1551	3996.33008	398.16174	38.3832
2	7.254	VV R	0.1781	4040.66016	346.37314	38.8090
3	8.394	VV R	0.2046	1187.39160	90.77835	11.4044
4	9.073	BB	0.2207	1187.27612	82.11418	11.4033

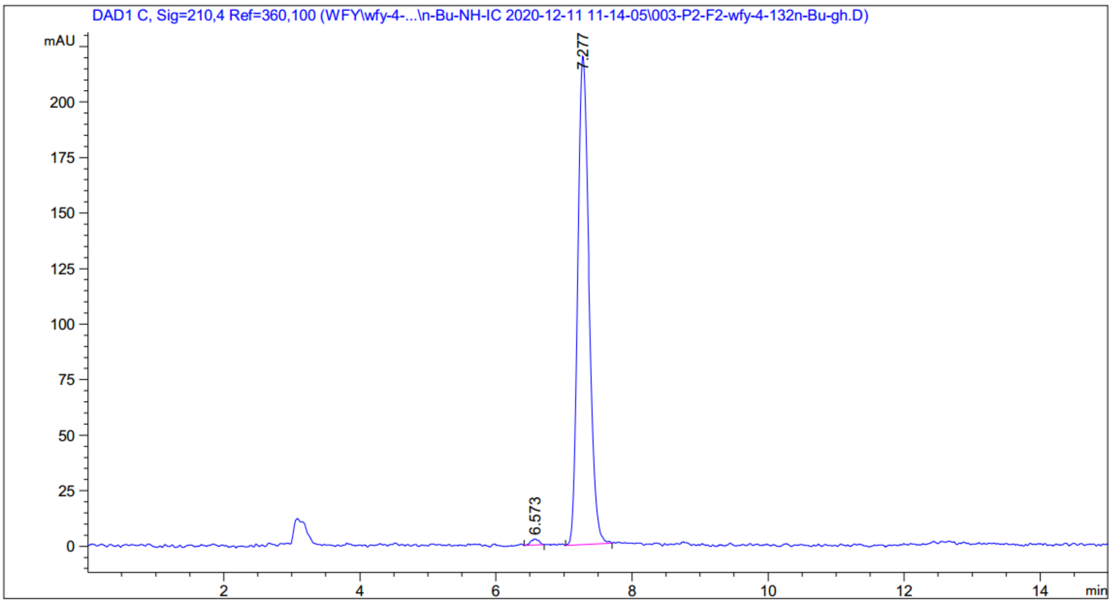

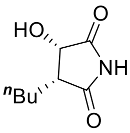

3aa

Signal 1: DAD1 C, Sig=210,4 Ref=360,100

Peak #	RetTime [min]	Type	Width [min]	Area [mAU*s]	Height [mAU]	Area %
1	5.203	MM	0.1247	23.67964	3.16456	0.7924
2	6.287	FM	0.1646	2964.48584	300.07996	99.2076

Supplementary Figure 215. HPLC spectrum of 3aa.

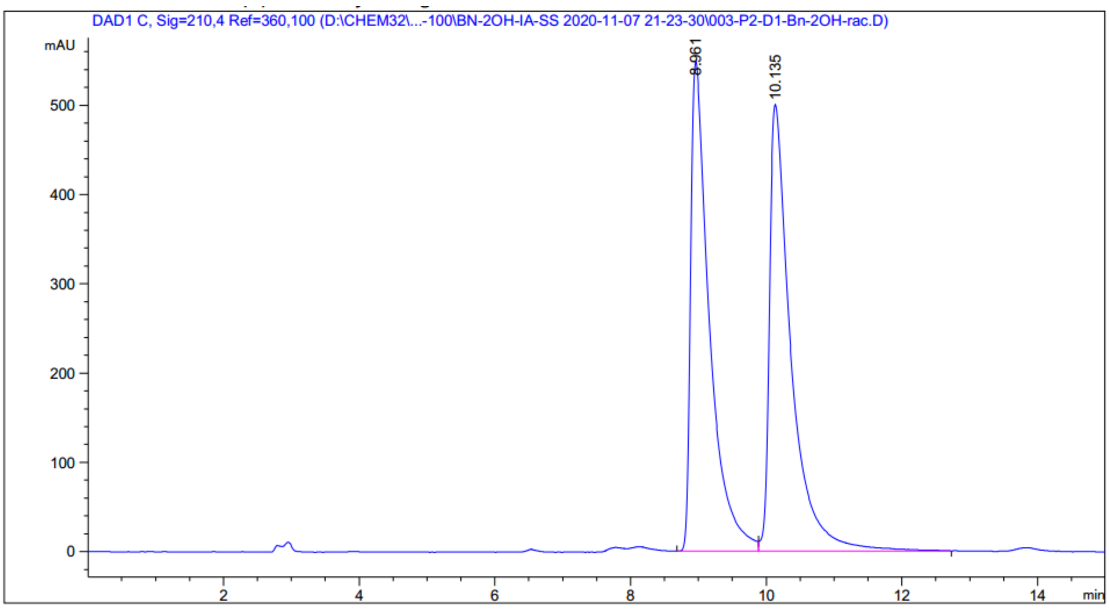

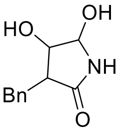

rac-**4y**

Signal 1: DAD1 C, Sig=210,4 Ref=360,100

Peak #	RetTime [min]	Type	Width [min]	Area [mAU*s]	Height [mAU]	Area %
1	8.961	BV	0.2818	1.06077e4	548.17206	48.9695
2	10.135	VV R	0.3178	1.10542e4	500.29910	51.0305

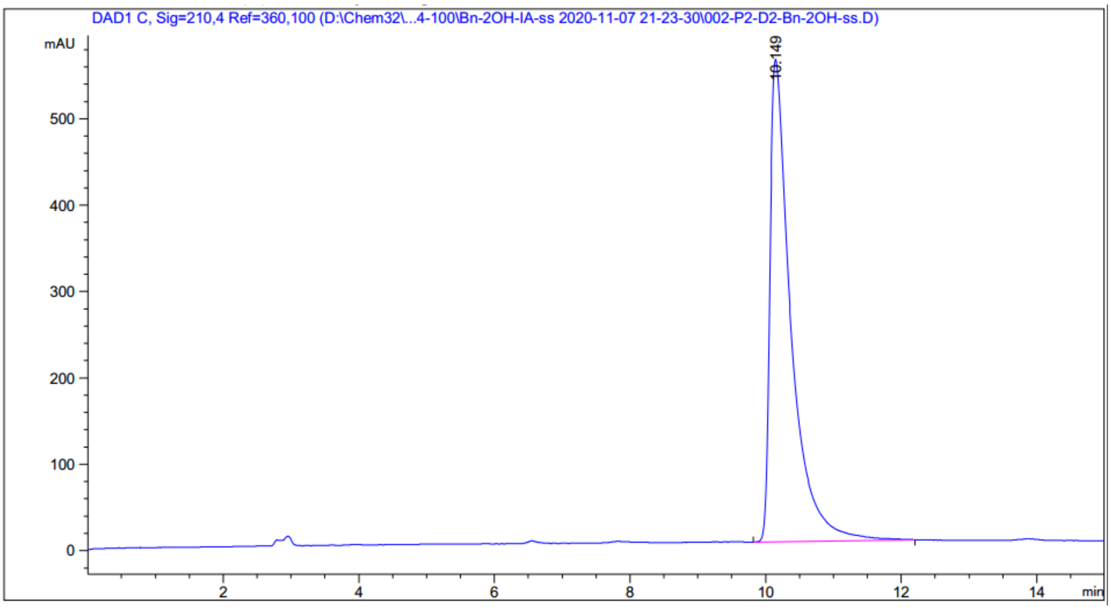

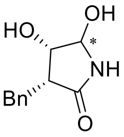

4y

Signal 1: DAD1 C, Sig=210,4 Ref=360,100

Peak #	RetTime [min]	Type	Width [min]	Area [mAU*s]	Height [mAU]	Area %
1	10.149	BV R	0.3112	1.20179e4	558.07745	100.0000

Supplementary Figure 216. HPLC spectrum of 4y.

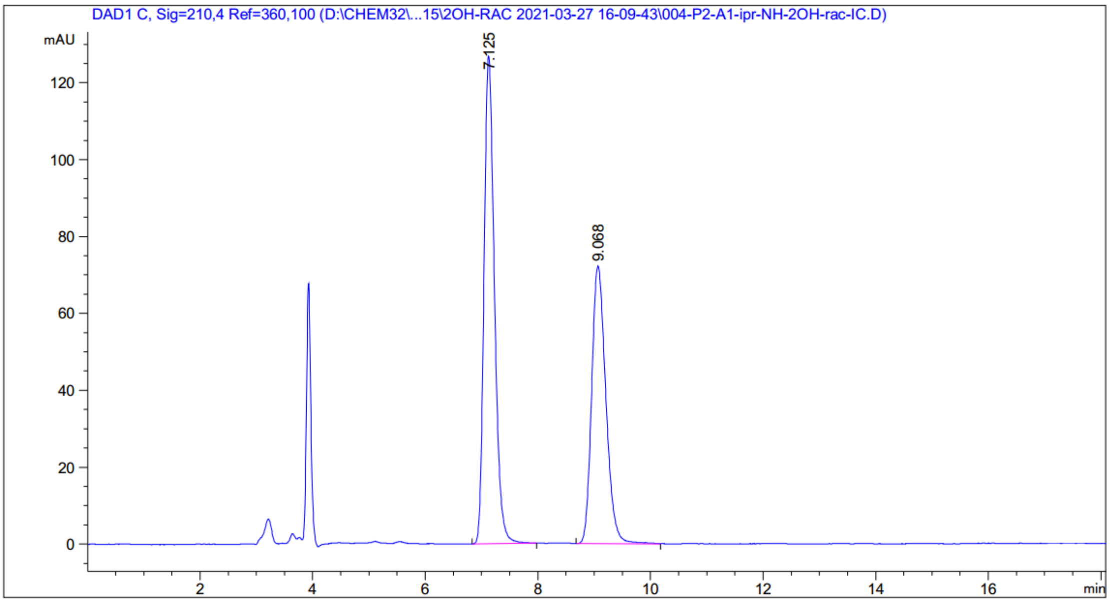

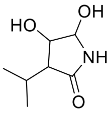

rac-**4z**

Signal 1: DAD1 C, Sig=210,4 Ref=360,100

Peak #	RetTime [min]	Type	Width [min]	Area [mAU*s]	Height [mAU]	Area %
1	7.125	BB	0.1972	1623.59473	126.93580	56.2132
2	9.068	BB	0.2703	1264.68750	72.23688	43.7868

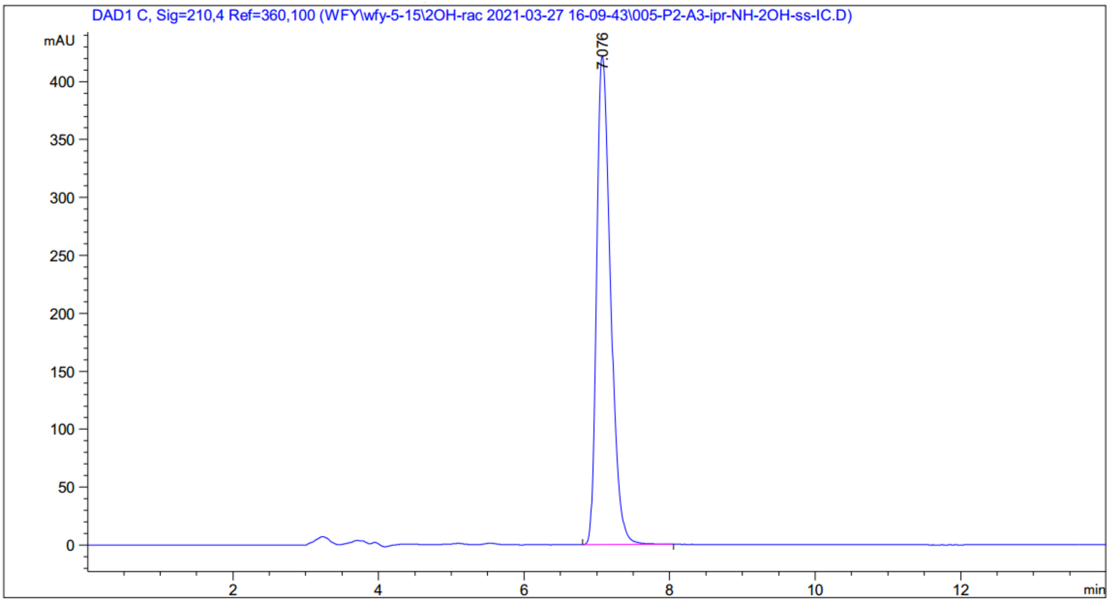

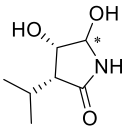

4z

Signal 1: DAD1 C, Sig=210,4 Ref=360,100

Peak #	RetTime [min]	Type	Width [min]	Area [mAU*s]	Height [mAU]	Area %
1	7.076	BB	0.2055	5616.02100	421.30676	100.0000

Supplementary Figure 217. HPLC spectrum of 4z.

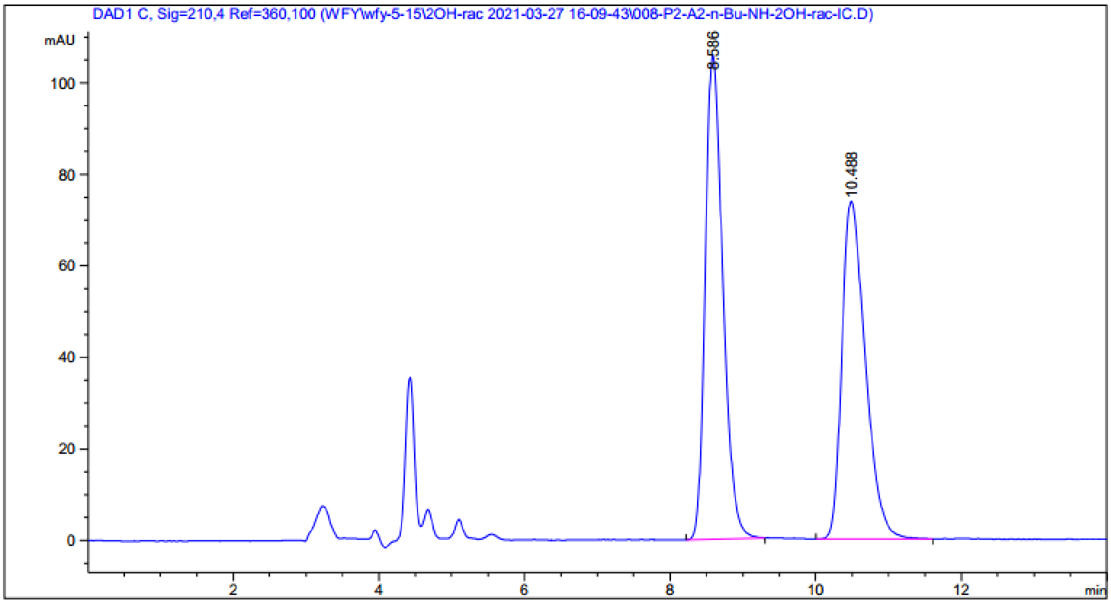

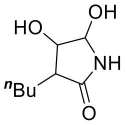

rac-**4aa**

Signal 1: DAD1 C, Sig=210,4 Ref=360,100

Peak #	RetTime [min]	Type	Width [min]	Area [mAU*s]	Height [mAU]	Area %
1	8.586	BB	0.2611	1783.07300	105.54889	51.9565
2	10.488	BB	0.3410	1648.78418	73.69838	48.0435

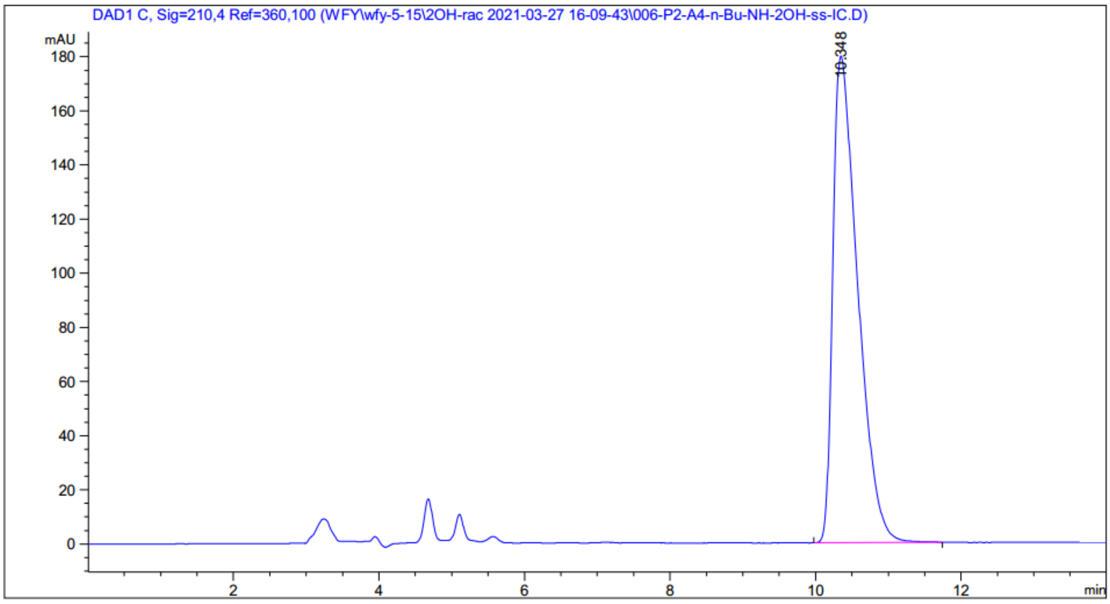

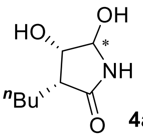

4aa

Signal 1: DAD1 C, Sig=210,4 Ref=360,100

Peak #	RetTime [min]	Type	Width [min]	Area [mAU*s]	Height [mAU]	Area %
1	10.348	BB	0.3565	4260.72754	179.76801	100.0000

Supplementary Figure 218. HPLC spectrum of 4aa.

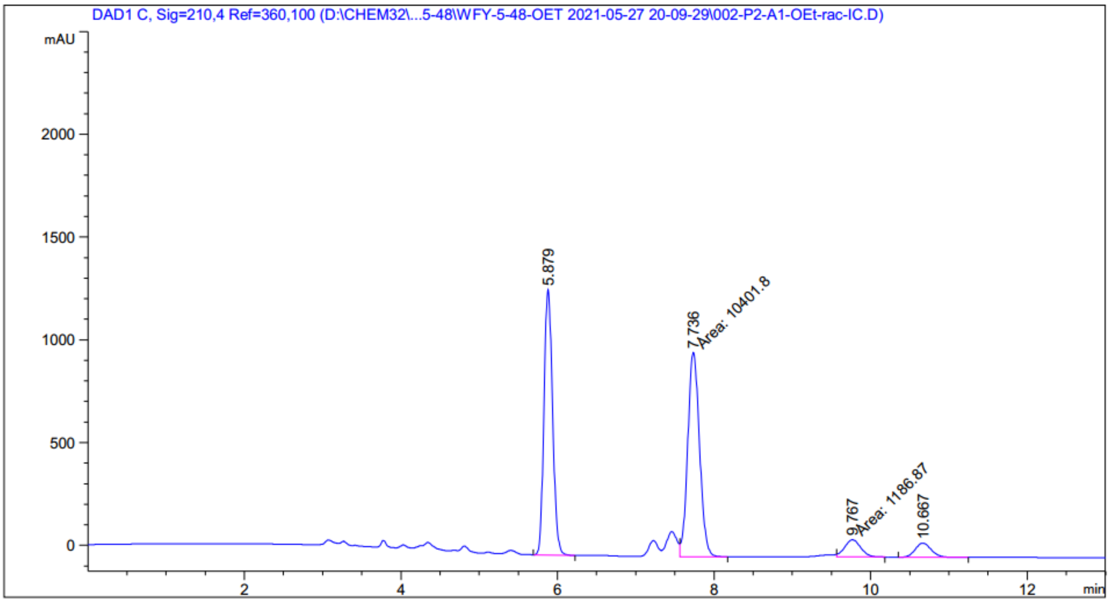

Signal 1: DAD1 C, Sig=210,4 Ref=360,100

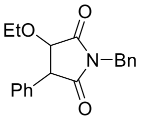

rac-**2a'**

Peak #	RetTime [min]	Type	Width [min]	Area [mAU*s]	Height [mAU]	Area %
1	5.879	BB	0.1190	9849.63770	1290.84302	43.9204
2	7.736	FM	0.1744	1.04018e4	994.21576	46.3826
3	9.767	FM	0.2363	1186.86584	83.70430	5.2923
4	10.667	BB	0.2213	987.79840	68.88770	4.4047

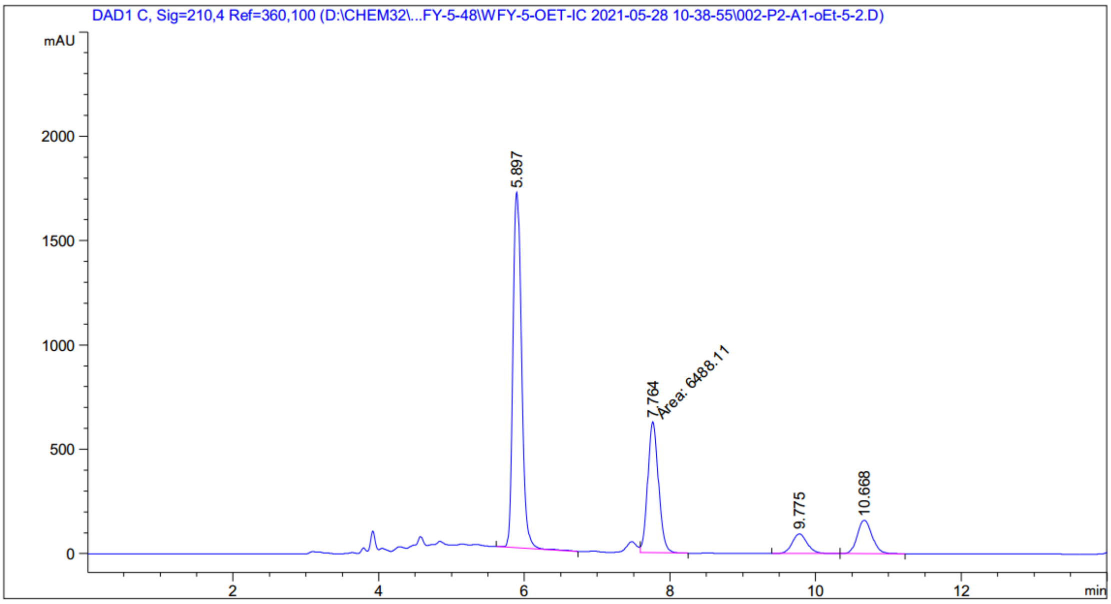

Signal 1: DAD1 C, Sig=210,4 Ref=360,100

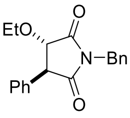

anti-**2a'**

Peak #	RetTime [min]	Type	Width [min]	Area [mAU*s]	Height [mAU]	Area %
1	5.897	VV R	0.1299	1.40486e4	1701.86487	58.1655
2	7.764	FM	0.1724	6488.11328	627.30084	26.8627
3	9.775	BV	0.2237	1359.08252	93.44040	5.6270
4	10.668	VB	0.2162	2257.02246	160.37088	9.3448

Supplementary Figure 219. HPLC spectrum of anti-2a'.

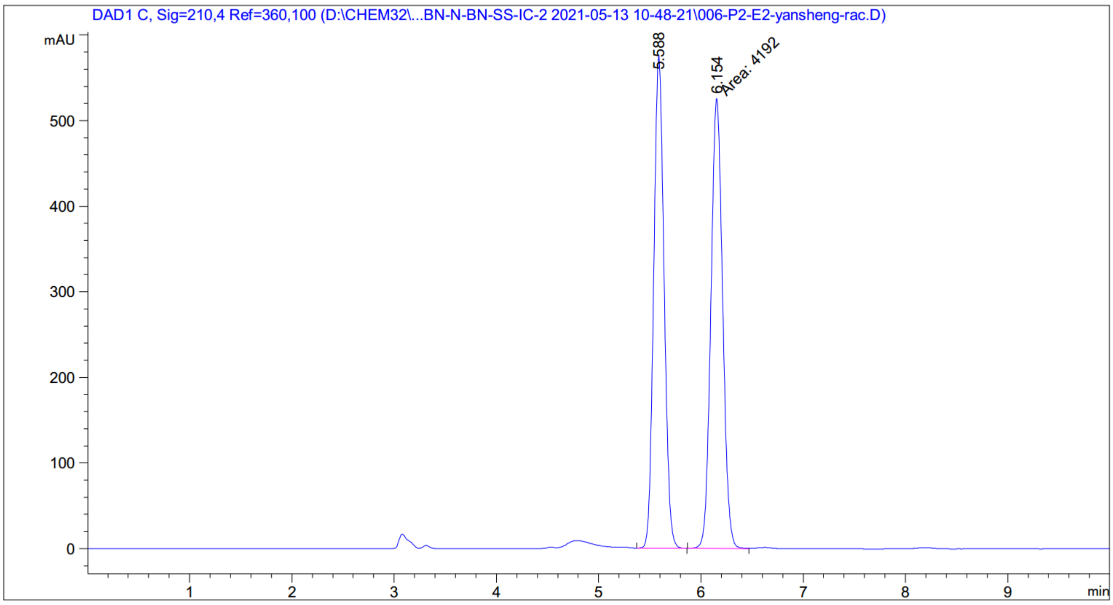

Signal 1: DAD1 C, Sig=210,4 Ref=360,100

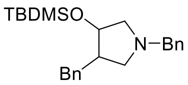

rac-**6w**

Peak #	RetTime [min]	Type	Width [min]	Area [mAU*s]	Height [mAU]	Area %
1	5.588	BB	0.1101	4050.44946	575.32410	49.1413
2	6.154	MF	0.1326	4191.99805	526.70441	50.8587

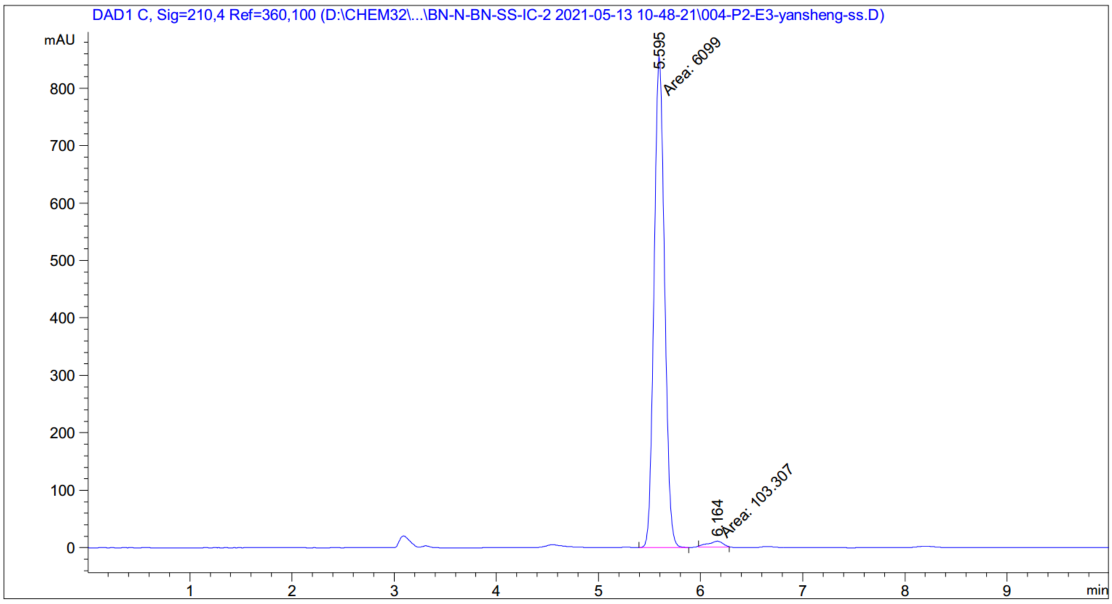

Signal 1: DAD1 C, Sig=210,4 Ref=360,100

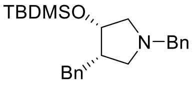

6w

Peak #	RetTime [min]	Type	Width [min]	Area [mAU*s]	Height [mAU]	Area %
1	5.595	FM	0.1188	6099.00195	855.77783	98.3344
2	6.164	MM	0.1721	103.30714	10.00595	1.6656

Supplementary Figure 220. HPLC spectrum of 6w.

5. Supplementary References

(1) Echeverria, P.-G.; Férard, C.; Phansavath, P.; Ratovelomanana-Vidal, V., Synthesis, characterization and use of a new tethered Rh(III) complex in asymmetric transfer hydrogenation of ketones. *Catal. Commun.* **2015**, *62*, 95.

(2) Tantray, M. A.; Khan, I.; Hamid, H.; Alam, M. S.; Dhulap, A.; Kalam, A., Synthesis of aryl anilinomaleimide based derivatives as glycogen synthase kinase-3β inhibitors with potential role as antidepressant agents. *New J. Chem.* **2016,** *40*, 6109.

(3) Zhang, X.; Cao, W. B.; Li, H. Y.; Xu, X. P.; Ji, S. J., Synthesis of Polysubstituted Maleimides via Metal-Free Cascade Reaction of Isocyanides and alpha-Diazoketones. *J. Org. Chem.* **2019,** *84*, 16237.

(4) Jaye, M. C.; Krawiec, J. A.; Campobasso, N.; Smallwood, A.; Qiu, C.; Lu, Q.; Kerrigan, J. J.; De Los Frailes Alvaro, M.; Laffitte, B.; Liu, W. S.; Marino, J. P., Jr.; Meyer, C. R.; Nichols, J. A.; Parks, D. J.; Perez, P.; Sarov-Blat, L.; Seepersaud, S. D.; Steplewski, K. M.; Thompson, S. K.; Wang, P.; Watson, M. A.; Webb, C. L.; Haigh, D.; Caravella, J. A.; Macphee, C. H.; Willson, T. M.; Collins, J. L., Discovery of substituted maleimides as liver X receptor agonists and determination of a ligand-bound crystal structure. *J. Med. Chem.* **2005,** *48*, 5419.

(5) Xu, G.; He, Q.; Yang, B.; Hu, Y., Synthesis and Antitumor Activity of Novel 4-Chloro-3-Arylmaleimide Derivatives. *Letters in Drug Design & Discovery* **2009,** *6*, 51.

(6) Neel, D. A.; Jirousek, M. R.; McDonald, J. H., Synthesis of bisindolylmaleimides using a palladium catalyzed cross-coupling reaction. *Bioorg. Med. Chem. Lett.* **1998,** *8*, 47.

(7) Rooney, C. S.; Randall, W. C.; Streeter, K. B.; Ziegler, C.; Cragoe, E. J., Jr.; Schwam, H.; Michelson, S. R.; Williams, H. W.; Eichler, E.; Duggan, D. E.; Ulm, E. H.; Noll, R. M., Inhibitors of glycolic acid oxidase. 4-Substituted 3-hydroxy-1H-pyrrole-2,5-dione derivatives. *J. Med. Chem.* **1983,** *26*, 700.

(8) Lin, G.-J.; Luo, S.-P.; Zheng, X.; Ye, J.-L.; Huang, P.-Q., Enantiodivergent synthesis of trans-3,4-disubstituted succinimides by SmI_2-mediated Reformatsky-type reaction. *Tetrahedron Letters* **2008,** *49*, 4007.

(9) Huang, P.; Meng, W., A New Approach to (2S, 3S, 4S)-3-Hydroxy-4-Methylproline, A Subunit in Echinocandin B and Related Oligopeptide Antibiotics. *Letters in Organic Chemistry* **2004,** *1*, 99.

(10) Raelin, J. M. Extension of Ketene-Mediated Asymmetric Methedology. Expansion of the Acyl Halide-Aldehyde Cyclocondensation Reactiion (AAC) and its Application in the Approach to Motuporin. Development of a Ketene-Claisen Rearrangement (MSc Thesis). **2005**.

(11) (a) Becke, A. D. *J. Chem. Phys.* **1993**, *98*, 5648; (b) Lee, C.; Yang, W.; Parr, R. G. *Phys. Rev. B*. **1988**, *37*, 785.

(12) Grimme, S.; Ehrlich, S.; Goerigk, L. *J. Comput. Chem*. **2011**, *32*, 1456.

(13) (a) Schwerdtfeger, P.; Dolg, M.; Schwarz, W. H.; Bowmaker, G. A.; Boyd, P. D. *J. Chem. Phys*. **1989**, *91*, 1762; (b) Bergner, A.; Dolg, M.; Küchle, W.; Stoll, H. *Mol. Phys*. **1993**, *80*, 1431; (c) Dolg, M.; Wedig, U.; Stoll, H.; Preuss, H. *J. Chem. Phys*. **1987**, *86*, 866; (d) Andrae, D.; Haüßermann, U.; Dolg, M.; Stoll, H.; Preuss, H. *Theor. Chim. Acta*. **1990**, *77*, 123.

(14) Yu, H. S.; He, X.; Li, S. L.; Truhlar, D. G. Chem. Sci. 2016, 7, 5032−5051.

(15) (a) Weigend, F.; Ahlrichs, R. *Phys. Chem. Chem. Phys*. **2005**, *7*, 3297−3305. (b) Weigend, F.

Phys. Chem. Chem. Phys. **2006**, *8,* 1057−1065.

(16) Cancès, E.; Mennucci, B.; Tomasi, J. *J. Chem. Phys*. **1997**, *107*, 3032−3041. pcm

(17) M. J. Frisch, G. W. Trucks, H. B. Schlegel, G. E. Scuseria, M. A. Robb, J. R. Cheeseman, G. Scalmani, V. Barone, G. A. Petersson, H. Nakatsuji, X. Li, M. Caricato, A. V. Marenich, J. Bloino, B. G. Janesko, R. Gomperts, B. Mennucci, H. P. Hratchian, J. V. Ortiz, A. F. Izmaylov, J. L. Sonnenberg, D. Williams-Young, F. Ding, F. Lipparini, F. Egidi, J. Goings, B. Peng, A. Petrone, T. Henderson, D. Ranasinghe, V. G. Zakrzewski, J. Gao, N. Rega, G. Zheng, W. Liang, M. Hada, M. Ehara, K. Toyota, R. Fukuda, J. Hasegawa, M. Ishida, T. Nakajima, Y. Honda, O. Kitao, H. Nakai, T. Vreven, K. Throssell, J. A. Montgomery, Jr., J. E. Peralta, F. Ogliaro, M. J. Bearpark, J. J. Heyd, E. N. Brothers, K. N. Kudin, V. N. Staroverov, T. A. Keith, R. Kobayashi, J. Normand, K. Raghavachari, A. P. Rendell, J. C. Burant, S. S. Iyengar, J. Tomasi, M. Cossi, J. M. Millam, M. Klene, C. Adamo, R. Cammi, J. W. Ochterski, R. L. Martin, K. Morokuma, O. Farkas, J. B. Foresman and D. J. Fox, *Gaussian 16, Revision A.03*, Gaussian, Inc., Wallingford, CT, 2016.
