# Supplementary material for: Stereodivergent synthesis of chiral succinimides via Rh-catalyzed asymmetric transfer hydrogenation
Source: Nat Commun. 2022 Dec 17;13:7794. doi: 10.1038/s41467-022-35124-5 (PMC9759521; doi:10.1038/s41467-022-35124-5)
Supplement: Supplementary file 2 — Description of Additional Supplementary Files [file 41467_2022_35124_MOESM2_ESM.docx]

**Description of Additional Supplementary Files**

File Name: Supplementary Data 1

Description: Cartesian coordinates of all optimized structures.
