# Supplementary material for: Stereodivergent synthesis of chiral succinimides via Rh-catalyzed asymmetric transfer hydrogenation
Source: Nat Commun. 2022 Dec 17;13:7794. doi: 10.1038/s41467-022-35124-5 (PMC9759521; doi:10.1038/s41467-022-35124-5)
Supplement: Supplementary file 3 — Supplementary Data 1 [file 41467_2022_35124_MOESM3_ESM.pdf]

Supplementary Data

Stereodivergent Synthesis of Chiral Succinimides via Rh-Catalyzed Asymmetric Transfer Hydrogenation

Fangyuan Wang#, Zongpeng Zhang#, Yu Chen#, Virginie Ratovelomanana-Vidal[c], Peiyuan Yu*, Gen-Qiang Chen* and Xumu Zhang*

Coordinates

----

cat6

Gsol = -2773.89430882 Hartree

Rh	0.550496	-1.758327	-0.379755
S	-2.368000	-1.385351	1.055346
O	-2.312994	-2.814439	0.682493
O	-2.586042	-1.103460	2.494583
O	5.795203	2.809163	0.569863
N	-1.013880	-0.658786	0.506991
N	0.830020	0.136060	-1.257076
C	-1.093048	0.751062	0.107859
C	0.333715	1.166996	-0.303859
C	-1.598027	1.711950	1.169434
C	-1.067266	1.693553	2.462893
C	-1.483374	2.628056	3.409470
C	-2.439417	3.590724	3.072042
C	-2.976401	3.610669	1.783689
C	-2.554808	2.674221	0.838519
C	0.415455	2.570127	-0.859175
C	-0.245058	2.922021	-2.043000
C	-0.153174	4.220715	-2.544028
C	0.598219	5.182809	-1.864498
C	1.256146	4.840061	-0.681583
C	1.165593	3.538993	-0.185248
C	-3.738141	-0.632039	0.177044
C	-3.813609	-0.773346	-1.211243
C	-4.847960	-0.144476	-1.899218
C	-5.813177	0.618025	-1.222266
C	-5.712235	0.740903	0.169450
C	-4.677830	0.124795	0.872960
C	-6.943059	1.272410	-1.978059
C	2.214207	0.359484	-1.753354
C	3.244753	0.416522	-0.647590
C	4.039996	1.565962	-0.575509
C	5.003881	1.715518	0.426981
C	5.178165	0.694640	1.371700
C	4.383572	-0.439881	1.308537
C	3.411046	-0.606885	0.307884
C	5.606660	3.895869	-0.333717
C	2.561426	-1.815171	0.362414
C	2.427468	-2.842581	-0.644651
C	1.453463	-3.792103	-0.154527

C	0.989561	-3.367351	1.125070
C	1.662015	-2.125016	1.447965
C	3.235568	-2.989772	-1.896230
C	1.034604	-5.022659	-0.889995
C	0.073696	-4.108676	2.043787
C	1.470185	-1.347019	2.708873
H	-1.734314	0.869594	-0.779623
H	0.975022	1.095072	0.577328
H	-0.345320	0.929115	2.726981
H	-1.068517	2.601638	4.413358
H	-2.767038	4.316239	3.811385
H	-3.725038	4.350577	1.514695
H	-2.977257	2.682309	-0.162156
H	-0.839586	2.185625	-2.578098
H	-0.670209	4.481691	-3.462686
H	0.668173	6.193961	-2.254493
H	1.837991	5.583642	-0.144539
H	1.678845	3.270074	0.733326
H	-3.059121	-1.350164	-1.737859
H	-4.908445	-0.243935	-2.980128
H	-6.445647	1.334749	0.708591
H	-4.581808	0.236966	1.946337
H	-7.354652	2.122456	-1.425112
H	-6.613438	1.626589	-2.960456
H	-7.762886	0.562328	-2.147865
H	2.413689	-0.450297	-2.458812
H	2.241766	1.300193	-2.310878
H	3.876648	2.352928	-1.300508
H	5.925729	0.816368	2.148272
H	4.511692	-1.221861	2.050946
H	4.584026	4.287466	-0.274720
H	6.312276	4.666643	-0.022763
H	5.822999	3.596313	-1.365991
H	3.867388	-3.884597	-1.841228
H	2.592598	-3.089960	-2.776616
H	3.893901	-2.129171	-2.039408
H	0.067341	-5.386842	-0.538416
H	0.957492	-4.825826	-1.961893
H	1.777284	-5.816852	-0.739344
H	0.667993	-4.723862	2.733182
H	-0.537069	-3.423608	2.633821
H	-0.604346	-4.760559	1.492827
H	1.857805	-0.330900	2.604412
H	0.408732	-1.290789	2.963166

H	1.999687	-1.827016	3.541469
H	0.211001	0.094037	-2.071352
Cl	-0.678759	-2.233866	-2.450562

NEt3

Gsol = -291.961871863 Hartree

C	-1.361706	-0.653974	-0.150039
C	-2.640028	0.160550	0.058065
H	-1.466233	-1.606926	0.381674
H	-1.257600	-0.905633	-1.222683
H	-3.512637	-0.418175	-0.265632
H	-2.761184	0.412092	1.117074
H	-2.635889	1.093989	-0.514215
C	0.906542	-0.935241	0.699178
C	1.558691	-1.670649	-0.482785
H	0.484511	-1.672528	1.393021
H	1.679550	-0.398191	1.259403
H	2.319522	-2.369356	-0.116386
H	0.822309	-2.246070	-1.054520
H	2.050693	-0.975236	-1.171588
C	0.257812	1.125686	-0.474952
C	1.381355	1.964192	0.133244
H	-0.612157	1.772933	-0.623794
H	0.558054	0.781592	-1.483558
H	1.541914	2.865124	-0.468788
H	1.121093	2.270998	1.152290
H	2.331408	1.421316	0.171987
N	-0.166931	0.012147	0.377297

NEt_3_HCl

Gsol = -752.713858007 Hartree

C	-1.404098	-0.718747	-0.108259
C	-2.645437	0.165223	-0.106605
H	-1.550938	-1.573921	0.555246
H	-1.178295	-1.095063	-1.108733
H	-3.516109	-0.462983	-0.317333
H	-2.785544	0.632537	0.872481
H	-2.607003	0.943700	-0.873024
C	0.947266	-0.970744	0.690355
C	1.569683	-1.598439	-0.548398
H	0.524920	-1.730703	1.351284
H	1.685404	-0.414789	1.269478

H	2.353676	-2.290240	-0.226485
H	0.843655	-2.169992	-1.133261
H	2.034149	-0.852746	-1.200203
C	0.213402	1.160801	-0.451580
C	1.424088	1.911439	0.086901
H	-0.653312	1.821754	-0.465631
H	0.375685	0.790024	-1.466937
H	1.544884	2.832324	-0.491245
H	1.274979	2.182271	1.136335
H	2.349675	1.336791	-0.006090
N	-0.178352	-0.011408	0.405878
H	-0.440414	0.390492	1.363820
Cl	-0.845936	1.085647	3.129009

----

cat0-0

Gsol = -2313.11081524 Hartree

Rh	-0.444593	-1.364759	0.167556
S	-2.801398	0.387676	1.370124
O	-3.241356	-0.878677	1.987699
O	-3.170169	1.657409	2.030665
O	6.347656	-0.706452	-0.724145
N	-1.181937	0.311845	1.145620
N	0.399160	0.016295	-0.911723
C	-0.588407	1.578014	0.662353
C	0.696361	1.213466	-0.116934
C	-0.260875	2.554254	1.774136
C	0.227047	2.097274	3.003239
C	0.606877	3.001833	3.994834
C	0.506587	4.377153	3.767123
C	0.020834	4.839515	2.542295
C	-0.359935	3.931005	1.553198
C	1.181855	2.374135	-0.963138
C	0.406235	2.850440	-2.028757
C	0.851672	3.919015	-2.805706
C	2.081153	4.524885	-2.527825
C	2.861271	4.052877	-1.471211
C	2.411940	2.980860	-0.696251
C	-3.530247	0.451944	-0.271148
C	-3.763578	-0.738238	-0.966564
C	-4.203072	-0.687479	-2.285241
C	-4.411933	0.541511	-2.932874
C	-4.182080	1.719964	-2.213494
C	-3.747750	1.684240	-0.886434

C	-4.903419	0.582095	-4.358566
C	1.510992	-0.445279	-1.742610
C	2.675288	-1.109982	-1.013200
C	3.959570	-0.593751	-1.213887
C	5.074467	-1.165972	-0.590200
C	4.903388	-2.280132	0.242201
C	3.628833	-2.787330	0.453265
C	2.498825	-2.223355	-0.161380
C	6.560664	0.447347	-1.531728
C	1.163148	-2.751848	0.198650
C	0.165070	-3.315366	-0.648140
C	-1.014659	-3.596174	0.171503
C	-0.752501	-3.214490	1.503065
C	0.570294	-2.618529	1.531157
C	0.312785	-3.685900	-2.090924
C	-2.263534	-4.223223	-0.359042
C	-1.639783	-3.394656	2.693010
C	1.267244	-2.086809	2.740215
H	-1.258124	2.069944	-0.054703
H	1.483872	0.984983	0.621392
H	0.285466	1.027642	3.178678
H	0.977722	2.634141	4.948063
H	0.799718	5.082134	4.540194
H	-0.065488	5.906874	2.357409
H	-0.733380	4.291766	0.598397
H	-0.546023	2.375169	-2.247847
H	0.240784	4.280923	-3.628196
H	2.427247	5.358298	-3.132614
H	3.817973	4.517371	-1.248977
H	3.018333	2.611279	0.126601
H	-3.602092	-1.688088	-0.470422
H	-4.391229	-1.613894	-2.822506
H	-4.349897	2.680271	-2.693814
H	-3.593131	2.600534	-0.327964
H	-4.784210	1.578715	-4.793642
H	-4.361925	-0.133738	-4.986722
H	-5.967744	0.319962	-4.413994
H	1.094122	-1.145622	-2.473024
H	1.908441	0.400223	-2.314993
H	4.070580	0.275011	-1.851542
H	5.772045	-2.719463	0.721210
H	3.499653	-3.640791	1.112776
H	6.007626	1.311685	-1.144419
H	7.631346	0.649485	-1.486564

H	6.266341	0.265388	-2.572310
H	0.207576	-4.770599	-2.215888
H	-0.450617	-3.207422	-2.714000
H	1.297799	-3.399510	-2.466723
H	-3.113645	-4.047597	0.304052
H	-2.510360	-3.834135	-1.351750
H	-2.131526	-5.308870	-0.457544
H	-1.348641	-4.297976	3.245305
H	-1.571151	-2.541570	3.370529
H	-2.686077	-3.489666	2.399806
H	2.018483	-1.344497	2.458533
H	0.556639	-1.624148	3.429368
H	1.779171	-2.897955	3.274862

HCOOH

Gsol = -189.647161524 Hartree

H	2.540647	-5.341793	4.252660
C	2.257270	-5.894890	6.031866
O	2.898801	-6.020123	4.859823
O	1.394997	-5.083087	6.271783
H	2.636000	-6.644159	6.740493

----

cat0-1

Gsol = -2502.78699310 Hartree

Rh	0.775860	-1.749010	-0.413469
S	-2.222090	-1.672412	0.826080
O	-2.186265	-3.013967	0.199881
O	-2.289984	-1.681424	2.311531
O	5.502255	3.252249	0.998229
N	-0.967864	-0.801055	0.281458
N	0.970725	0.170769	-1.243288
C	-1.109872	0.617739	-0.063228
C	0.320543	1.137962	-0.318956
C	-1.787831	1.494676	0.972551
C	-1.452508	1.407675	2.326796
C	-2.060147	2.248693	3.258960
C	-3.007052	3.188955	2.844313
C	-3.341056	3.283745	1.491511
C	-2.732720	2.439927	0.563223
C	0.343815	2.552391	-0.850807
C	-0.203421	2.858371	-2.103965
C	-0.175383	4.168495	-2.582606

C	0.397519	5.185477	-1.814282
C	0.942989	4.886230	-0.564136
C	0.917487	3.574221	-0.088564
C	-3.691022	-0.824574	0.249555
C	-3.876788	-0.632599	-1.121590
C	-4.995052	0.070453	-1.562042
C	-5.935126	0.579612	-0.653226
C	-5.731631	0.357183	0.715012
C	-4.612614	-0.334810	1.172074
C	-7.119645	1.378521	-1.137378
C	2.360781	0.514447	-1.623165
C	3.289088	0.652422	-0.435832
C	3.974569	1.864379	-0.297190
C	4.825196	2.093640	0.788986
C	4.998541	1.089298	1.751239
C	4.311611	-0.108550	1.621903
C	3.451829	-0.354518	0.538000
C	5.294802	4.323412	0.080439
C	2.705244	-1.630742	0.518927
C	2.755975	-2.660714	-0.496280
C	1.829814	-3.695841	-0.096659
C	1.208629	-3.320846	1.129717
C	1.739300	-2.023876	1.512596
C	3.701701	-2.736194	-1.654739
C	1.570259	-4.934577	-0.890004
C	0.251880	-4.131225	1.944282
C	1.369280	-1.277450	2.753247
H	-1.660155	0.728067	-1.007068
H	0.872815	1.099159	0.623352
H	-0.736775	0.660527	2.647622
H	-1.799326	2.166021	4.310594
H	-3.484669	3.839508	3.571734
H	-4.081159	4.007119	1.160776
H	-3.005040	2.498133	-0.486509
H	-0.650207	2.072085	-2.706518
H	-0.602148	4.395663	-3.555282
H	0.417567	6.205060	-2.188157
H	1.386356	5.671855	0.041106
H	1.344252	3.338398	0.882087
H	-3.144926	-1.001989	-1.831774
H	-5.140315	0.229808	-2.627300
H	-6.451510	0.742741	1.432077
H	-4.432962	-0.479677	2.230690
H	-7.969936	1.286039	-0.454382

H	-6.868606	2.445336	-1.204241
H	-7.440053	1.056259	-2.133219
H	2.691927	-0.266197	-2.312468
H	2.354641	1.458983	-2.175952
H	3.810043	2.636916	-1.037266
H	5.657945	1.272725	2.592891
H	4.436494	-0.878187	2.377723
H	4.238887	4.617696	0.046746
H	5.895009	5.154159	0.452510
H	5.629332	4.053336	-0.928250
H	4.434762	-3.537273	-1.497192
H	3.177003	-2.948596	-2.591634
H	4.255884	-1.801403	-1.769029
H	0.695273	-5.471480	-0.519192
H	1.398552	-4.686784	-1.941630
H	2.436891	-5.605072	-0.835494
H	0.803566	-4.710733	2.696739
H	-0.467086	-3.491967	2.459691
H	-0.315286	-4.823208	1.320511
H	1.644442	-0.223197	2.671975
H	0.293804	-1.348287	2.932600
H	1.893057	-1.696538	3.621855
C	-0.770075	-1.781552	-3.078410
O	-0.182994	-2.462905	-2.172507
O	-0.810907	-0.541660	-3.215855
H	-1.294262	-2.410284	-3.824816
H	0.385335	0.083862	-2.096130

----

cat0-2

Gsol = -2502.76203646 Hartree

Rh	-0.468879	-1.469708	0.165413
S	-2.536117	0.529715	1.686778
O	-2.984732	-0.752677	2.261429
O	-2.614536	1.738372	2.532166
O	6.419995	-0.593119	-0.701438
N	-0.997713	0.319982	1.151074
N	0.519475	-0.076665	-1.031234
C	-0.376282	1.526509	0.567546
C	0.900886	1.069393	-0.166362
C	0.006266	2.595661	1.574267
C	0.639315	2.250088	2.772095
C	1.072280	3.240318	3.652320
C	0.878238	4.589653	3.342072

C	0.247425	4.939661	2.147213
C	-0.184568	3.944716	1.268261
C	1.574792	2.179682	-0.939643
C	0.942578	2.777944	-2.037034
C	1.579788	3.794046	-2.749084
C	2.854041	4.223751	-2.369190
C	3.488193	3.632456	-1.274740
C	2.850512	2.612530	-0.566901
C	-3.572055	0.855030	0.263206
C	-4.256671	-0.197138	-0.347771
C	-4.988846	0.044568	-1.506467
C	-5.038645	1.323805	-2.078074
C	-4.362868	2.369213	-1.434759
C	-3.634744	2.146233	-0.266875
C	-5.790522	1.560018	-3.363814
C	1.632097	-0.652937	-1.831987
C	2.777383	-1.181166	-0.998892
C	4.048461	-0.655469	-1.254379
C	5.164475	-1.075458	-0.522917
C	5.006365	-2.044349	0.477952
C	3.745314	-2.556308	0.742524
C	2.613565	-2.144679	0.017773
C	6.610410	0.440733	-1.665409
C	1.297549	-2.676462	0.428509
C	0.355367	-3.424552	-0.362347
C	-0.808302	-3.664174	0.470127
C	-0.593044	-3.078025	1.750417
C	0.703211	-2.433900	1.726972
C	0.567273	-3.993821	-1.731315
C	-2.018916	-4.407451	0.009862
C	-1.469462	-3.196754	2.953755
C	1.329181	-1.710498	2.873082
H	-1.030947	1.980130	-0.191965
H	1.600246	0.677322	0.576566
H	0.766998	1.201173	3.017815
H	1.557266	2.960632	4.583678
H	1.213325	5.361430	4.029283
H	0.089531	5.985466	1.898813
H	-0.666668	4.217815	0.333214
H	-0.049430	2.452437	-2.340486
H	1.081267	4.250463	-3.599064
H	3.348778	5.015743	-2.923635
H	4.477108	3.963978	-0.971484
H	3.342623	2.148949	0.283245

H	-4.212698	-1.189385	0.085834
H	-5.515615	-0.776640	-1.984337
H	-4.412648	3.372605	-1.849182
H	-3.144585	2.967406	0.244688
H	-6.729259	0.996463	-3.386394
H	-6.021236	2.620342	-3.504246
H	-5.194994	1.232623	-4.225640
H	1.183157	-1.432165	-2.451497
H	2.010927	0.120361	-2.506122
H	4.148318	0.104571	-2.018454
H	5.874748	-2.366685	1.042457
H	3.625028	-3.293595	1.530550
H	6.005074	1.322951	-1.424969
H	7.668662	0.698635	-1.619007
H	6.362010	0.092836	-2.674922
H	0.635740	-5.087423	-1.676445
H	-0.263509	-3.746005	-2.398422
H	1.498473	-3.627418	-2.170013
H	-2.821501	-4.359445	0.748507
H	-2.384762	-3.989026	-0.934241
H	-1.767269	-5.462146	-0.159600
H	-1.135757	-4.046833	3.563922
H	-1.429573	-2.293419	3.562520
H	-2.511920	-3.355913	2.677098
H	2.126874	-1.047222	2.531067
H	0.581872	-1.116531	3.404742
H	1.764468	-2.423648	3.584740
C	-2.028080	-1.284978	-2.197362
O	-2.606329	-2.317420	-2.509835
O	-1.610981	-0.271324	-2.772321
H	-1.829744	-1.233260	-0.968333
H	-0.214393	0.202650	-1.706959

----

cat0-TS1

Gsol = -2502.76365273 Hartree

Rh	-0.746448	-1.713188	0.147632
S	2.357752	-1.620864	0.650718
O	2.424621	-1.588835	2.134404
O	2.305492	-2.953591	0.013628
O	-5.252611	3.386516	-1.583018
N	1.127122	-0.718919	0.029590
N	-1.206209	0.033189	1.193293
C	1.077306	0.656438	0.592239

C	-0.391202	1.120858	0.588070
C	1.914800	1.653105	-0.191866
C	1.697638	1.837898	-1.562011
C	2.454072	2.759109	-2.283242
C	3.446315	3.505657	-1.641562
C	3.666942	3.328169	-0.275278
C	2.898180	2.411864	0.444468
C	-0.590604	2.458168	1.265652
C	-0.349263	2.616248	2.636044
C	-0.552301	3.851815	3.250892
C	-0.993425	4.944424	2.499968
C	-1.230101	4.795381	1.131919
C	-1.032459	3.556174	0.521122
C	3.835977	-0.790921	0.074372
C	4.792806	-0.370107	0.993401
C	5.908173	0.335373	0.541010
C	6.070221	0.633879	-0.815888
C	5.104157	0.173220	-1.724578
C	3.992873	-0.537386	-1.290068
C	7.228886	1.469545	-1.297183
C	-2.666443	0.316535	1.255431
C	-3.300987	0.613701	-0.087287
C	-3.981677	1.832304	-0.201141
C	-4.597750	2.210509	-1.397940
C	-4.541118	1.348717	-2.501173
C	-3.860675	0.144900	-2.394204
C	-3.230758	-0.250495	-1.201476
C	-5.276539	4.315789	-0.502351
C	-2.450155	-1.507436	-1.213694
C	-2.639888	-2.668603	-0.364597
C	-1.587917	-3.606564	-0.683947
C	-0.746912	-3.023754	-1.678846
C	-1.291032	-1.724838	-2.024586
C	-3.818582	-2.949804	0.517352
C	-1.453625	-4.969683	-0.083070
C	0.429164	-3.655359	-2.351660
C	-0.714619	-0.801877	-3.048880
H	1.417364	0.660561	1.636732
H	-0.725964	1.199407	-0.448627
H	0.947697	1.239396	-2.067487
H	2.276406	2.889545	-3.347377
H	4.043502	4.218353	-2.203497
H	4.440172	3.898502	0.231623
H	3.076252	2.269777	1.506494

H	0.000059	1.775448	3.230299
H	-0.363460	3.961714	4.314660
H	-1.149290	5.906595	2.978939
H	-1.567234	5.641654	0.540205
H	-1.221005	3.436758	-0.541718
H	4.647857	-0.576307	2.047400
H	6.652068	0.675927	1.256390
H	5.217300	0.395821	-2.782155
H	3.232966	-0.860968	-1.992353
H	8.013560	1.544364	-0.538456
H	7.670830	1.056286	-2.210462
H	6.892745	2.488025	-1.532096
H	-3.118706	-0.553666	1.735688
H	-2.826494	1.174885	1.914424
H	-4.001291	2.493552	0.655533
H	-5.021150	1.644394	-3.428058
H	-3.807461	-0.514219	-3.255510
H	-4.261321	4.605894	-0.205884
H	-5.813639	5.189342	-0.872900
H	-5.804122	3.903082	0.365752
H	-4.496007	-3.664106	0.031587
H	-3.510987	-3.383122	1.473428
H	-4.392037	-2.040407	0.713901
H	-0.457714	-5.382794	-0.256521
H	-1.625254	-4.941969	0.995331
H	-2.189112	-5.650794	-0.531040
H	0.129247	-4.068495	-3.324122
H	1.222573	-2.923939	-2.518991
H	0.851347	-4.458243	-1.746729
H	-1.111820	0.209979	-2.935621
H	0.374606	-0.761798	-2.958776
H	-0.954279	-1.144845	-4.063654
C	-0.848631	-2.605999	3.036746
O	-0.991732	-3.790499	3.065160
O	-0.900797	-1.528668	3.567417
H	-0.273608	-2.338706	1.590435
H	-0.899716	-0.148610	2.154417

----

cat1

Gsol = -2314.30181616 Hartree

Rh	-0.433652	-1.672517	-0.194876
S	-2.833726	-0.091239	1.342138
O	-3.229664	-1.476687	1.659445

O	-3.110149	0.951736	2.355400
O	6.285536	0.387479	-0.607128
N	-1.244200	-0.087301	0.947813
N	0.400123	-0.034249	-1.207247
C	-0.734815	1.238747	0.550370
C	0.610009	1.020497	-0.173495
C	-0.512391	2.207773	1.698230
C	0.105355	1.781593	2.877891
C	0.386897	2.691244	3.896016
C	0.053366	4.040513	3.745130
C	-0.563883	4.471308	2.569671
C	-0.842674	3.556927	1.552201
C	1.191536	2.295791	-0.741658
C	0.547883	2.984343	-1.777246
C	1.096631	4.157022	-2.296683
C	2.294148	4.657859	-1.780017
C	2.938128	3.980456	-0.742438
C	2.389804	2.803541	-0.230874
C	-3.759480	0.406435	-0.113843
C	-4.191931	-0.555027	-1.030716
C	-4.835381	-0.148916	-2.197126
C	-5.050764	1.210340	-2.472539
C	-4.613289	2.156829	-1.537168
C	-3.976189	1.764317	-0.359395
C	-5.771453	1.635360	-3.728125
C	1.630293	-0.384621	-1.976889
C	2.805925	-0.792181	-1.111379
C	3.981225	-0.046870	-1.275443
C	5.122700	-0.312074	-0.514121
C	5.093339	-1.350911	0.424901
C	3.931287	-2.089901	0.591256
C	2.770172	-1.836605	-0.160098
C	6.329290	1.492218	-1.505795
C	1.555823	-2.625082	0.141099
C	0.766098	-3.390364	-0.819690
C	-0.377593	-3.902371	-0.115420
C	-0.330448	-3.419794	1.229360
C	0.895525	-2.658458	1.397022
C	1.213349	-3.812552	-2.188418
C	-1.387740	-4.853980	-0.680638
C	-1.268434	-3.778927	2.338052
C	1.308915	-1.983487	2.666666
H	-1.406480	1.721101	-0.177887
H	1.315791	0.595976	0.543710

H	0.341914	0.730003	2.997604
H	0.862456	2.347598	4.810772
H	0.269877	4.748881	4.539905
H	-0.830182	5.517209	2.444164
H	-1.314765	3.894690	0.633276
H	-0.392429	2.613093	-2.178325
H	0.587531	4.681172	-3.100158
H	2.720138	5.572369	-2.182026
H	3.865422	4.367752	-0.330067
H	2.892002	2.272981	0.572547
H	-4.031230	-1.604823	-0.818672
H	-5.179744	-0.897892	-2.905945
H	-4.781228	3.213768	-1.727316
H	-3.666598	2.499575	0.375229
H	-6.859005	1.558382	-3.600090
H	-5.544339	2.674038	-3.987322
H	-5.500340	1.000486	-4.578254
H	1.333472	-1.176421	-2.667055
H	1.926899	0.482923	-2.573883
H	3.980051	0.765822	-1.990324
H	5.980753	-1.555417	1.014743
H	3.910917	-2.890973	1.323837
H	5.571177	2.242545	-1.250554
H	7.324101	1.925511	-1.397436
H	6.183179	1.167967	-2.543242
H	1.546109	-4.858854	-2.176343
H	0.409071	-3.730042	-2.925763
H	2.059610	-3.211732	-2.531249
H	-2.366216	-4.721198	-0.212649
H	-1.504411	-4.713800	-1.758435
H	-1.071216	-5.891707	-0.509633
H	-0.848431	-4.600406	2.934878
H	-1.446196	-2.929095	2.999321
H	-2.238231	-4.096390	1.951652
H	2.065053	-1.216560	2.479017
H	0.446615	-1.508725	3.144804
H	1.730142	-2.702695	3.381040
H	-1.661418	-1.575877	-1.185507
H	-0.290519	0.296517	-1.880776

CO_2

Gsol = -188.487839652 Hartree

C	0.348076	0.012395	-0.054062

O	1.517490	0.012395	-0.054062
O	-0.821338	0.012395	-0.054062

1g

Gsol = -665.127079267 Hartree

C	0.046863	-5.244465	3.320222
C	1.108511	-4.231622	3.616867
C	0.712548	-3.386311	4.604689
O	2.240108	-4.288163	2.912411
N	-0.986278	-4.955358	4.175300
H	-1.863515	-5.455045	4.233492
C	-0.666125	-3.845102	4.988454
O	0.144195	-6.120847	2.477475
O	-1.426596	-3.401989	5.826290
C	1.424677	-2.259221	5.207688
C	0.842263	-1.481121	6.227379
C	2.724743	-1.928888	4.771156
C	1.538782	-0.412069	6.787102
H	-0.154036	-1.721695	6.573822
C	3.412864	-0.859114	5.336467
H	3.193775	-2.512627	3.988549
C	2.825062	-0.094153	6.347237
H	1.071366	0.175312	7.572358
H	4.413222	-0.622296	4.985456
H	3.364391	0.740029	6.786455
H	2.148071	-5.040625	2.290102

1g-R

Gsol = -665.115820543 Hartree

C	0.391937	-5.366598	3.160894
C	0.902807	-4.881042	4.537568
C	0.213969	-3.548518	4.840997
O	1.707601	-5.477318	5.206450
N	-0.444024	-4.357552	2.712374
H	-0.918141	-4.395699	1.815083
C	-0.584736	-3.260862	3.566785
O	0.667084	-6.401017	2.596695
O	-1.238911	-2.275581	3.311513
C	1.164639	-2.451124	5.262939
C	1.944927	-1.777758	4.314813
C	1.312119	-2.141720	6.618262
C	2.853829	-0.800910	4.719479

H	1.840697	-2.007366	3.257794
C	2.223931	-1.165941	7.023060
H	0.710832	-2.663657	7.357326
C	2.995906	-0.493040	6.074517
H	3.450315	-0.280877	3.975900
H	2.327167	-0.930542	8.078111
H	3.703001	0.269013	6.388361
H	-0.514792	-3.732719	5.641909

----

int1

Gsol = -2979.43308139 Hartree

Rh	0.610814	-0.772405	-1.056411
S	1.109466	2.285948	-0.365109
O	1.826274	2.204020	0.957457
O	1.977159	2.245582	-1.557912
O	-5.424597	-4.291472	-0.528167
N	-0.072787	1.180143	-0.533602
N	-0.496136	-1.235187	0.663732
C	-1.038185	1.166740	0.597877
C	-1.615863	-0.257805	0.717036
C	-2.189937	2.139422	0.405489
C	-2.961311	2.092005	-0.761649
C	-4.034165	2.962810	-0.938787
C	-4.346249	3.899676	0.050727
C	-3.582415	3.950793	1.217275
C	-2.514806	3.068585	1.394214
C	-2.487061	-0.421593	1.943514
C	-1.925976	-0.446698	3.226650
C	-2.742047	-0.601098	4.347803
C	-4.125663	-0.724621	4.198635
C	-4.690719	-0.695491	2.921779
C	-3.872367	-0.548375	1.801054
C	0.285516	3.870238	-0.316647
C	0.430747	4.704243	0.788435
C	-0.285670	5.900249	0.834132
C	-1.150817	6.263197	-0.203639
C	-1.255774	5.416211	-1.318278
C	-0.544518	4.225454	-1.382526
C	-1.992088	7.510668	-0.115981
C	-0.954816	-2.654803	0.687694
C	-1.945897	-3.010401	-0.402513
C	-3.187825	-3.495654	0.028186
C	-4.189986	-3.842523	-0.882205

C	-3.940762	-3.719204	-2.254779
C	-2.713790	-3.235909	-2.686302
C	-1.697759	-2.866766	-1.787441
C	-5.734329	-4.363046	0.860857
C	-0.463036	-2.260017	-2.331474
C	0.907407	-2.685769	-2.044546
C	1.788860	-1.735589	-2.666575
C	0.991182	-0.710603	-3.264112
C	-0.410616	-1.066569	-3.096118
C	1.319286	-4.006071	-1.459747
C	3.283333	-1.841114	-2.710724
C	1.491727	0.442037	-4.076507
C	-1.559299	-0.238226	-3.580340
H	-0.535623	1.397219	1.546393
H	-2.218507	-0.452406	-0.172724
H	-2.701261	1.384545	-1.541909
H	-4.622423	2.917445	-1.851372
H	-5.177358	4.585359	-0.088533
H	-3.813297	4.679179	1.989488
H	-1.918120	3.112634	2.300838
H	-0.849935	-0.364002	3.355147
H	-2.296418	-0.625274	5.337982
H	-4.759180	-0.843330	5.072870
H	-5.765972	-0.787819	2.797355
H	-4.308315	-0.528333	0.806501
H	1.079681	4.406282	1.603225
H	-0.185041	6.550380	1.699003
H	-1.922162	5.685638	-2.132936
H	-0.655468	3.559187	-2.230572
H	-2.015849	8.047385	-1.070557
H	-3.028847	7.252198	0.136634
H	-1.621459	8.193096	0.654594
H	-0.050558	-3.260440	0.635034
H	-1.418438	-2.850908	1.657780
H	-3.367835	-3.571895	1.092774
H	-4.717588	-3.995168	-2.960263
H	-2.527432	-3.134297	-3.751182
H	-5.086873	-5.082125	1.377032
H	-5.639474	-3.381286	1.340715
H	-6.769790	-4.700365	0.918329
H	2.063476	-3.889889	-0.665333
H	0.457943	-4.539959	-1.050247
H	1.759208	-4.647003	-2.234499
H	3.666210	-2.377177	-1.838382

H	3.603673	-2.385056	-3.609362
H	3.752191	-0.854233	-2.728567
H	0.849920	1.317703	-3.958129
H	2.497817	0.733361	-3.770601
H	1.514350	0.175973	-5.142351
H	-2.491502	-0.534559	-3.092300
H	-1.383496	0.822255	-3.372781
H	-1.699208	-0.344891	-4.663853
H	0.101477	-1.103495	1.482365
C	2.169131	-2.339115	2.671180
C	2.708808	-1.005750	2.211845
C	3.977355	-1.165105	1.746058
O	1.870836	0.011700	2.354788
H	1.862252	-0.583034	-0.140810
N	3.199052	-3.236386	2.506859
H	3.142740	-4.228056	2.696491
C	4.320338	-2.607659	1.928512
O	1.038242	-2.565134	3.068604
O	5.348241	-3.188920	1.639256
C	4.924270	-0.221801	1.138180
C	6.278451	-0.239168	1.518065
C	4.508460	0.688419	0.153968
C	7.185100	0.646150	0.938676
H	6.613911	-0.946779	2.268147
C	5.418078	1.574422	-0.420939
H	3.481464	0.693712	-0.181757
C	6.758354	1.558693	-0.030706
H	8.226927	0.625015	1.245971
H	5.068608	2.271926	-1.176333
H	7.467724	2.248274	-0.479698
H	2.043677	0.809062	1.767645

S-TS1

Gsol = -2979.40169627 Hartree

Rh	0.796111	-0.905423	-0.469422
S	0.589666	2.222923	-1.313198
O	2.052095	2.207706	-0.968443
O	0.342440	2.250244	-2.774796
O	-4.993025	-4.853361	-0.642216
N	-0.129072	0.989442	-0.570378
N	-0.724500	-1.096031	0.976306
C	-1.346120	1.160575	0.235545
C	-1.872376	-0.259529	0.527287

C	-2.465737	1.957280	-0.410610
C	-2.804717	1.754298	-1.752134
C	-3.855745	2.467744	-2.327962
C	-4.582943	3.384481	-1.564569
C	-4.256543	3.578484	-0.220543
C	-3.204678	2.865073	0.352166
C	-3.019816	-0.260596	1.511626
C	-2.820393	0.096310	2.851550
C	-3.891916	0.099100	3.745407
C	-5.172324	-0.249204	3.308284
C	-5.377243	-0.601495	1.972471
C	-4.303567	-0.609619	1.081301
C	-0.086923	3.724857	-0.621243
C	0.112148	4.010109	0.731093
C	-0.476757	5.151301	1.270394
C	-1.253241	6.009914	0.479424
C	-1.409971	5.710767	-0.881176
C	-0.836083	4.571760	-1.436278
C	-1.934317	7.212626	1.081940
C	-1.100230	-2.513430	1.243711
C	-1.752999	-3.199729	0.061093
C	-3.031978	-3.729174	0.269028
C	-3.741381	-4.341168	-0.769285
C	-3.157008	-4.432910	-2.039558
C	-1.891575	-3.906285	-2.250833
C	-1.164696	-3.284764	-1.220685
C	-5.644827	-4.722657	0.618958
C	0.150266	-2.696475	-1.542942
C	1.404413	-2.972612	-0.862026
C	2.408742	-2.135919	-1.455534
C	1.790134	-1.299211	-2.437232
C	0.389572	-1.677796	-2.524074
C	1.680730	-4.083213	0.106947
C	3.857772	-2.215101	-1.112434
C	2.485173	-0.323428	-3.335009
C	-0.608032	-1.069907	-3.456359
H	-1.104197	1.627711	1.200759
H	-2.210106	-0.689425	-0.418195
H	-2.221019	1.062329	-2.348414
H	-4.103410	2.312226	-3.374404
H	-5.396603	3.945766	-2.015223
H	-4.814949	4.291429	0.379586
H	-2.940050	3.026354	1.392652
H	-1.828655	0.369415	3.202270

H	-3.726643	0.375754	4.782572
H	-6.005534	-0.244347	4.004920
H	-6.370967	-0.867400	1.623569
H	-4.459905	-0.884452	0.042291
H	0.703247	3.349139	1.356337
H	-0.333350	5.377699	2.323350
H	-2.002269	6.370436	-1.509322
H	-0.981064	4.319647	-2.479502
H	-1.913014	8.067961	0.398619
H	-2.988972	6.992553	1.293375
H	-1.463862	7.510718	2.023609
H	-0.184690	-3.014819	1.561319
H	-1.797054	-2.536569	2.086554
H	-3.478505	-3.627951	1.249770
H	-3.710438	-4.908520	-2.842312
H	-1.444425	-3.975933	-3.237643
H	-5.102437	-5.261970	1.404593
H	-5.746946	-3.669068	0.905447
H	-6.633180	-5.164685	0.490096
H	2.165604	-3.718094	1.018260
H	0.762042	-4.606034	0.382993
H	2.353924	-4.820183	-0.348600
H	4.013396	-2.225770	-0.030697
H	4.273572	-3.150096	-1.511586
H	4.424115	-1.379357	-1.522790
H	1.842495	0.530008	-3.559774
H	3.395025	0.060111	-2.867444
H	2.763353	-0.808712	-4.280148
H	-1.628688	-1.250196	-3.109463
H	-0.446570	0.008070	-3.537545
H	-0.512405	-1.506251	-4.459349
H	-0.358309	-0.707777	1.854201
C	1.845128	-0.481180	2.841236
C	2.634631	0.256682	1.758129
C	3.940940	-0.305695	1.745682
O	2.333207	1.581063	1.619566
H	1.886718	-0.317182	0.701553
N	2.610899	-1.551622	3.183521
H	2.360667	-2.238475	3.883396
C	3.909237	-1.495312	2.578654
O	0.729529	-0.198878	3.270596
O	4.749029	-2.361617	2.803097
C	5.084395	0.112109	0.950403
C	6.383474	-0.366845	1.231437

C	4.930229	0.987099	-0.147111
C	7.470712	0.013239	0.449368
H	6.524196	-1.049434	2.059857
C	6.024245	1.361869	-0.924077
H	3.950841	1.350565	-0.424348
C	7.303006	0.880279	-0.634245
H	8.458608	-0.371670	0.689192
H	5.870260	2.030715	-1.766936
H	8.153922	1.173046	-1.242726
H	2.294217	1.829989	0.658588

S-int2

Gsol = -2979.41584582 Hartree

Rh	-1.151051	-1.462656	-0.575116
S	1.956751	-2.175659	-0.792806
O	1.890172	-2.851229	0.552886
O	1.734138	-3.074007	-1.941639
O	-4.277383	4.715727	-1.891861
N	0.915532	-0.919027	-0.917707
N	-1.047281	0.334438	0.513623
C	1.280515	0.342139	-0.225122
C	-0.014712	1.175457	-0.152485
C	2.319047	1.175657	-0.956712
C	2.188051	1.430923	-2.325700
C	3.094416	2.263292	-2.979902
C	4.145429	2.849247	-2.269383
C	4.277486	2.600763	-0.902154
C	3.364442	1.771899	-0.249230
C	0.172738	2.532771	0.485979
C	0.645727	2.655748	1.795702
C	0.811062	3.915057	2.370435
C	0.515708	5.066187	1.636389
C	0.054314	4.950341	0.322790
C	-0.117870	3.688039	-0.246882
C	3.614873	-1.533127	-0.888543
C	4.376346	-1.418330	0.271989
C	5.645394	-0.847550	0.193973
C	6.153396	-0.385240	-1.025449
C	5.377323	-0.547352	-2.183974
C	4.113357	-1.119122	-2.125617
C	7.487378	0.310858	-1.097936
C	-2.361048	1.005393	0.709887
C	-2.986425	1.509777	-0.568361

C	-3.355007	2.858123	-0.614731
C	-3.916064	3.413624	-1.769269
C	-4.113184	2.604887	-2.897736
C	-3.741510	1.269773	-2.856617
C	-3.182891	0.696076	-1.701566
C	-4.042255	5.583465	-0.784040
C	-2.778922	-0.722700	-1.759553
C	-3.283915	-1.812491	-0.955403
C	-2.589007	-3.009515	-1.378891
C	-1.669823	-2.676085	-2.411694
C	-1.760310	-1.245992	-2.636448
C	-4.469828	-1.772954	-0.044727
C	-2.827178	-4.373692	-0.822562
C	-0.872678	-3.632006	-3.237280
C	-0.980569	-0.486399	-3.657930
H	1.624422	0.161562	0.802232
H	-0.364120	1.324716	-1.177049
H	1.384974	0.955944	-2.878826
H	2.985213	2.450539	-4.044708
H	4.856408	3.493948	-2.778315
H	5.093104	3.049657	-0.342259
H	3.465608	1.579734	0.814742
H	0.884209	1.774171	2.376359
H	1.174056	3.988706	3.391245
H	0.647638	6.047614	2.082861
H	-0.168552	5.840416	-0.258967
H	-0.480507	3.596971	-1.266525
H	3.976638	-1.770886	1.214123
H	6.242556	-0.747144	1.096048
H	5.764627	-0.203108	-3.138504
H	3.510652	-1.227141	-3.019795
H	8.063188	-0.014289	-1.970943
H	7.341894	1.395128	-1.190437
H	8.086274	0.129485	-0.200780
H	-2.996976	0.277502	1.215383
H	-2.220257	1.841486	1.399382
H	-3.169345	3.473168	0.256042
H	-4.544799	3.042323	-3.791618
H	-3.887729	0.648536	-3.735180
H	-4.618809	5.270457	0.094223
H	-2.976958	5.617697	-0.527093
H	-4.374275	6.571045	-1.105126
H	-4.352562	-2.463560	0.792600
H	-4.648484	-0.766715	0.340130

H	-5.366382	-2.071149	-0.604439
H	-2.908092	-4.343021	0.266433
H	-3.767309	-4.771907	-1.226868
H	-2.022616	-5.059004	-1.097479
H	0.018593	-3.156156	-3.643301
H	-0.545800	-4.494937	-2.655119
H	-1.494006	-3.992151	-4.068586
H	-1.024974	0.588092	-3.466926
H	0.064713	-0.803605	-3.649541
H	-1.389390	-0.669752	-4.659336
H	-0.741907	0.044858	1.459942
C	-2.476483	-2.664657	2.730615
C	-0.967370	-2.349149	2.650874
C	-0.836875	-0.980484	3.236014
O	-0.607909	-2.558018	1.237313
H	-0.397657	-3.121414	3.183418
N	-3.070381	-1.562933	3.246702
H	-4.060239	-1.488141	3.442649
C	-2.125062	-0.529183	3.626932
O	-3.027142	-3.695113	2.357134
O	-2.541942	0.501529	4.173024
C	0.410057	-0.381275	3.678521
C	0.433525	0.727723	4.564201
C	1.662437	-0.858823	3.223446
C	1.631125	1.321888	4.948900
H	-0.510556	1.117667	4.923528
C	2.857481	-0.251131	3.610873
H	1.706005	-1.700527	2.542201
C	2.858520	0.849671	4.469616
H	1.605763	2.170316	5.629184
H	3.798666	-0.642584	3.232966
H	3.790269	1.324517	4.762728
H	0.364582	-2.746571	1.143827

SS-TS2

Gsol = -2979.39947520 Hartree

Rh	-1.179281	-1.454206	-0.597833
S	1.922263	-2.107431	-0.979253
O	1.908644	-2.868862	0.326821
O	1.686816	-2.946753	-2.170498
O	-4.357574	4.733802	-1.607346
N	0.861202	-0.871079	-0.992896
N	-1.058271	0.264018	0.564061

C	1.230711	0.367487	-0.263546
C	-0.087849	1.150783	-0.103531
C	2.219669	1.248274	-1.006062
C	2.032474	1.540494	-2.361418
C	2.892229	2.415724	-3.022745
C	3.953614	3.009034	-2.333978
C	4.142554	2.723887	-0.980619
C	3.275912	1.851561	-0.320956
C	0.102076	2.492995	0.575222
C	0.584646	2.586157	1.883732
C	0.760386	3.829390	2.489907
C	0.462023	5.000534	1.789230
C	-0.010512	4.917310	0.477269
C	-0.189708	3.669656	-0.122595
C	3.568207	-1.433570	-1.082817
C	4.351498	-1.341731	0.064837
C	5.605506	-0.739558	-0.017938
C	6.077922	-0.223598	-1.230051
C	5.281361	-0.362533	-2.377471
C	4.031438	-0.964035	-2.313754
C	7.394224	0.505390	-1.304133
C	-2.362077	0.914153	0.796742
C	-3.041191	1.477305	-0.434615
C	-3.411598	2.825819	-0.421558
C	-3.995923	3.425193	-1.541993
C	-4.220044	2.661276	-2.696054
C	-3.849584	1.324460	-2.713629
C	-3.263300	0.709553	-1.594760
C	-4.088276	5.558000	-0.475562
C	-2.858596	-0.705272	-1.701176
C	-3.324602	-1.809409	-0.897880
C	-2.653196	-3.001946	-1.378535
C	-1.786416	-2.654011	-2.444880
C	-1.872609	-1.215425	-2.624760
C	-4.472078	-1.793932	0.063108
C	-2.870446	-4.368815	-0.817606
C	-1.023058	-3.588701	-3.326227
C	-1.129846	-0.434908	-3.658452
H	1.621664	0.153118	0.739704
H	-0.450652	1.353290	-1.120351
H	1.221633	1.059882	-2.898057
H	2.738628	2.631107	-4.076835
H	4.628415	3.687242	-2.848783
H	4.966617	3.177925	-0.437219

H	3.422720	1.629887	0.731936
H	0.827696	1.688336	2.435587
H	1.133779	3.877188	3.509130
H	0.600534	5.970124	2.259264
H	-0.235677	5.822080	-0.080871
H	-0.560018	3.605853	-1.141562
H	3.980162	-1.734151	1.002238
H	6.218081	-0.656255	0.875542
H	5.640506	0.023994	-3.326817
H	3.412117	-1.053587	-3.198489
H	7.957022	0.228569	-2.201947
H	7.222545	1.588821	-1.349685
H	8.017518	0.302613	-0.428356
H	-2.997409	0.173274	1.288159
H	-2.218254	1.728797	1.515188
H	-3.203984	3.407419	0.467346
H	-4.670377	3.133470	-3.562756
H	-4.017236	0.736514	-3.611348
H	-4.644525	5.217271	0.405806
H	-3.016784	5.575793	-0.243523
H	-4.420790	6.559354	-0.750991
H	-4.282788	-2.434921	0.926900
H	-4.690599	-0.780801	0.407362
H	-5.374312	-2.169586	-0.437833
H	-2.865059	-4.344650	0.275090
H	-3.846133	-4.751392	-1.145057
H	-2.101086	-5.065752	-1.156864
H	-0.093318	-3.136584	-3.668687
H	-0.764546	-4.512707	-2.804931
H	-1.635712	-3.849958	-4.199891
H	-1.149138	0.633007	-3.429782
H	-0.089797	-0.765654	-3.708463
H	-1.587211	-0.579846	-4.645340
H	-0.743416	-0.225018	1.797659
C	-2.268161	-2.665560	2.791648
C	-0.787657	-2.342112	2.536014
C	-0.619031	-0.889023	2.982931
O	-0.535781	-2.636546	1.153321
H	-0.172871	-3.033400	3.127069
N	-2.788261	-1.600236	3.468476
H	-3.751694	-1.549075	3.778877
C	-1.864782	-0.544879	3.687154
O	-2.874116	-3.665108	2.438232
O	-2.185224	0.453681	4.320801

C	0.683673	-0.476712	3.580285
C	0.778198	0.514541	4.578309
C	1.884835	-1.032248	3.100911
C	2.016612	0.933091	5.061990
H	-0.130480	0.967439	4.953274
C	3.123122	-0.610395	3.589772
H	1.863427	-1.789884	2.326311
C	3.201056	0.379370	4.569432
H	2.053987	1.700908	5.830670
H	4.032543	-1.060836	3.201154
H	4.164992	0.711824	4.943597
H	0.435452	-2.801838	0.983823

SS-pro

Gsol = -666.310129707 Hartree

C	-2.631687	-0.172293	0.359222
C	-1.302210	-0.929032	0.320172
C	-0.397983	-0.051769	-0.565671
H	-0.531669	-0.407201	-1.595801
N	-2.367661	1.129442	-0.021821
H	-3.062255	1.869174	-0.004637
C	-1.075043	1.326120	-0.511197
O	-3.708672	-0.640247	0.672087
O	-0.619723	2.395368	-0.851565
C	1.068241	-0.046488	-0.216511
C	1.984235	-0.733587	-1.018863
C	1.528531	0.607485	0.933933
C	3.337806	-0.771235	-0.678861
H	1.634990	-1.243417	-1.912715
C	2.879930	0.573088	1.274713
H	0.830198	1.153792	1.562705
C	3.788737	-0.117804	0.468937
H	4.037687	-1.309296	-1.311576
H	3.223863	1.088037	2.167016
H	4.841741	-0.143518	0.733736
O	-1.454694	-2.229567	-0.193524
H	-2.266794	-2.591512	0.203669
H	-0.906312	-0.944001	1.348877

----

a-R-int1

Gsol = -2979.41405900 Hartree

Rh	-0.701317	-1.545182	-0.656258

S	1.547159	0.299848	-2.054624
O	2.378558	-0.922107	-1.846794
O	1.158802	0.526049	-3.470648
O	-7.005557	1.009438	1.024901
N	0.232073	0.289341	-1.099922
N	-1.256700	-0.415746	1.028948
C	0.004774	1.356047	-0.116634
C	-1.320996	1.009186	0.593386
C	-0.111798	2.749826	-0.709809
C	-0.780983	2.954211	-1.920157
C	-0.905312	4.239692	-2.447602
C	-0.364328	5.333198	-1.766564
C	0.296405	5.133547	-0.552160
C	0.418271	3.847859	-0.027212
C	-1.641539	1.953093	1.730044
C	-0.851185	1.983570	2.886470
C	-1.158495	2.862111	3.926065
C	-2.254941	3.721397	3.819208
C	-3.043572	3.698373	2.666934
C	-2.737786	2.815251	1.630564
C	2.556798	1.693144	-1.555044
C	2.682563	2.787588	-2.409006
C	3.442813	3.880594	-2.003970
C	4.075054	3.898975	-0.752711
C	3.945445	2.778654	0.079565
C	3.190629	1.675747	-0.312113
C	4.848279	5.112702	-0.301680
C	-2.519365	-0.899416	1.659175
C	-3.747629	-0.745978	0.783548
C	-4.793508	0.023445	1.309444
C	-5.965283	0.253079	0.582283
C	-6.101510	-0.307803	-0.693757
C	-5.067436	-1.068242	-1.220147
C	-3.878160	-1.304065	-0.509055
C	-6.873933	1.649962	2.290722
C	-2.794104	-2.052982	-1.179342
C	-2.099576	-3.221815	-0.644021
C	-1.040096	-3.543492	-1.557871
C	-1.036355	-2.572682	-2.610195
C	-2.155692	-1.667868	-2.391243
C	-2.541451	-4.068815	0.514144
C	-0.133319	-4.729399	-1.436353
C	-0.153120	-2.571629	-3.819067
C	-2.476307	-0.495356	-3.263706

H	0.787108	1.373488	0.652554
H	-2.119544	1.058317	-0.150507
H	-1.169831	2.095924	-2.457093
H	-1.417896	4.387327	-3.394395
H	-0.453233	6.333283	-2.181789
H	0.724623	5.977613	-0.018459
H	0.945223	3.689262	0.908698
H	0.007634	1.323826	2.976404
H	-0.539581	2.877293	4.818541
H	-2.491645	4.405763	4.628720
H	-3.894594	4.367067	2.573525
H	-3.351277	2.793706	0.734572
H	2.172938	2.779466	-3.364725
H	3.538542	4.738720	-2.663805
H	4.441213	2.766917	1.046520
H	3.088494	0.825905	0.350879
H	5.389285	5.574815	-1.134019
H	4.170015	5.873541	0.106584
H	5.569620	4.860557	0.481610
H	-2.332168	-1.935432	1.939262
H	-2.681976	-0.335867	2.582895
H	-4.664585	0.464669	2.289422
H	-7.012802	-0.129829	-1.255072
H	-5.173687	-1.496108	-2.212382
H	-6.010661	2.326401	2.306366
H	-7.790687	2.223540	2.431250
H	-6.774127	0.916707	3.100079
H	-2.790459	-5.081866	0.175093
H	-1.765370	-4.153713	1.282126
H	-3.437451	-3.650667	0.980403
H	-0.008875	-5.021737	-0.390524
H	-0.552269	-5.588312	-1.976995
H	0.856083	-4.520626	-1.850415
H	0.753319	-3.156330	-3.644956
H	-0.681122	-3.012062	-4.675874
H	0.152300	-1.557121	-4.084764
H	-2.998887	-0.813552	-4.175211
H	-3.117414	0.219343	-2.741181
H	-1.555648	0.014487	-3.562724
H	-0.517156	-0.514097	1.728768
C	0.995094	-2.793385	2.228794
C	1.844796	-1.598043	1.755262
C	3.006684	-2.144803	0.938136
O	1.640743	-0.453809	2.113155

H	0.613077	-1.982471	0.097419
H	2.789789	-1.897787	-0.112989
N	1.690993	-3.913767	1.822001
H	1.371897	-4.859859	2.004064
C	2.858592	-3.658383	1.088809
O	-0.036549	-2.755192	2.871909
O	3.588032	-4.523640	0.659377
C	4.366293	-1.595840	1.302790
C	5.147220	-0.978479	0.320715
C	4.847268	-1.674863	2.615963
C	6.392567	-0.439611	0.648430
H	4.765244	-0.906203	-0.692536
C	6.092177	-1.138945	2.942403
H	4.249274	-2.153740	3.387509
C	6.867033	-0.518010	1.958714
H	6.987698	0.044015	-0.120585
H	6.456236	-1.204036	3.963605
H	7.835386	-0.097699	2.214426

----

a-RS-TS1

Gsol = -2979.40911678 Hartree

Rh	-0.677682	-1.473496	-0.604275
S	1.542445	0.355655	-2.094216
O	2.214429	-0.972215	-2.071447
O	1.152725	0.816610	-3.450937
O	-7.047781	0.908441	1.010986
N	0.291813	0.320120	-1.066289
N	-1.245431	-0.268669	1.002946
C	-0.014105	1.466189	-0.198695
C	-1.346246	1.132970	0.501948
C	-0.126281	2.808039	-0.896982
C	-0.874965	2.949734	-2.069260
C	-0.999639	4.197211	-2.679835
C	-0.375085	5.316455	-2.122829
C	0.373500	5.180241	-0.951760
C	0.495215	3.930937	-0.343844
C	-1.687510	2.117232	1.596453
C	-0.888814	2.219123	2.743365
C	-1.215039	3.130467	3.747939
C	-2.338269	3.951026	3.614748
C	-3.135275	3.855960	2.472104
C	-2.810902	2.939692	1.470331
C	2.690339	1.563319	-1.438750

C	3.110874	2.621351	-2.241233
C	4.012762	3.549225	-1.723404
C	4.498167	3.434114	-0.413712
C	4.050008	2.364226	0.375356
C	3.150510	1.429177	-0.126871
C	5.494753	4.424963	0.134218
C	-2.470072	-0.760449	1.688626
C	-3.714623	-0.699072	0.825301
C	-4.791664	0.041992	1.325159
C	-5.976612	0.183775	0.595570
C	-6.091491	-0.435417	-0.656006
C	-5.023828	-1.164293	-1.158691
C	-3.824525	-1.314284	-0.441832
C	-6.942332	1.609495	2.247834
C	-2.711647	-2.042044	-1.088587
C	-2.019023	-3.204266	-0.570298
C	-0.945983	-3.503744	-1.488654
C	-0.960085	-2.544883	-2.547323
C	-2.067067	-1.637443	-2.309292
C	-2.442275	-4.042345	0.598321
C	-0.007388	-4.661259	-1.359493
C	-0.079863	-2.535913	-3.756987
C	-2.439344	-0.489940	-3.191081
H	0.741536	1.559987	0.589749
H	-2.142013	1.135061	-0.246714
H	-1.335032	2.074173	-2.512551
H	-1.577607	4.295143	-3.594740
H	-0.466646	6.287034	-2.602398
H	0.869291	6.043482	-0.516656
H	1.092456	3.817846	0.556271
H	-0.012883	1.585086	2.853174
H	-0.590810	3.201250	4.633951
H	-2.589571	4.661113	4.397246
H	-4.007342	4.493815	2.359367
H	-3.431572	2.860737	0.582332
H	2.717280	2.717933	-3.246308
H	4.339933	4.379641	-2.343368
H	4.411876	2.255568	1.394165
H	2.792438	0.629062	0.508811
H	6.514367	4.021222	0.083713
H	5.482245	5.360728	-0.432865
H	5.290721	4.655430	1.185263
H	-2.239802	-1.774220	2.019658
H	-2.636641	-0.153226	2.583428

H	-4.678418	0.530985	2.284124
H	-7.011934	-0.322689	-1.218990
H	-5.112228	-1.632761	-2.134311
H	-6.110835	2.324305	2.228924
H	-7.884023	2.146113	2.366726
H	-6.807213	0.917192	3.087275
H	-2.679122	-5.061290	0.269215
H	-1.665885	-4.106587	1.367070
H	-3.341605	-3.626166	1.060114
H	0.097695	-4.969850	-0.316988
H	-0.390254	-5.520322	-1.925574
H	0.984294	-4.418225	-1.748787
H	0.873674	-3.027148	-3.556069
H	-0.574476	-3.064529	-4.583007
H	0.137826	-1.516511	-4.081748
H	-3.009177	-0.841110	-4.061100
H	-3.059325	0.232158	-2.654054
H	-1.542405	0.017570	-3.555609
H	-0.449287	-0.322416	1.662659
C	0.902603	-2.950306	2.141634
C	1.521814	-1.681619	1.513855
C	2.823764	-2.140048	0.800578
O	1.354002	-0.561068	2.057887
H	0.669650	-1.825513	0.328099
H	2.747978	-1.919733	-0.268779
N	1.701013	-4.007904	1.744651
H	1.503637	-4.972450	1.991953
C	2.803261	-3.660127	0.958926
O	-0.090206	-3.026187	2.842155
O	3.584890	-4.464524	0.497563
C	4.104312	-1.528269	1.327491
C	5.119735	-1.207415	0.419469
C	4.302646	-1.275154	2.689986
C	6.314051	-0.639300	0.861813
H	4.956248	-1.380114	-0.639979
C	5.495506	-0.702352	3.133267
H	3.519063	-1.503895	3.405021
C	6.504389	-0.383010	2.221045
H	7.088786	-0.386489	0.143758
H	5.635272	-0.503835	4.192134
H	7.430622	0.066239	2.567913

----

a-RS-int2

Gsol = -2979.43206735 Hartree

Rh	0.186557	-1.326491	-0.352076
S	1.153531	1.285895	-2.110136
O	2.240994	0.302294	-2.295114
O	0.596771	1.872985	-3.352184
O	-6.688198	-2.421670	0.552703
N	-0.019382	0.597754	-1.202933
N	-1.130698	-0.440044	1.038921
C	-0.850076	1.512111	-0.406885
C	-1.877644	0.635688	0.332341
C	-1.592063	2.570963	-1.202568
C	-2.384285	2.213246	-2.298849
C	-3.123874	3.177690	-2.979457
C	-3.075222	4.515286	-2.573626
C	-2.282253	4.878992	-1.484620
C	-1.545680	3.907754	-0.803081
C	-2.784396	1.426505	1.249063
C	-2.287157	2.056781	2.396947
C	-3.141130	2.788331	3.223288
C	-4.497946	2.901105	2.910451
C	-4.998820	2.276481	1.766300
C	-4.144527	1.541357	0.943635
C	1.824117	2.665132	-1.172412
C	1.716546	3.963338	-1.664471
C	2.264252	5.015876	-0.929544
C	2.922003	4.785822	0.285595
C	3.005716	3.467929	0.762921
C	2.455594	2.409114	0.046699
C	3.545569	5.921859	1.058085
C	-2.018246	-1.410688	1.734221
C	-3.009241	-2.085626	0.812685
C	-4.365885	-1.963353	1.133272
C	-5.354061	-2.521996	0.316272
C	-4.977342	-3.223702	-0.836918
C	-3.633314	-3.339497	-1.160171
C	-2.627654	-2.779012	-0.354687
C	-7.112121	-1.650378	1.674049
C	-1.223202	-2.890071	-0.800182
C	-0.122124	-3.479596	-0.053553
C	1.055848	-3.345703	-0.862933
C	0.717202	-2.675580	-2.076613
C	-0.705626	-2.394091	-2.042901
C	-0.223688	-4.275598	1.212680
C	2.404810	-3.863571	-0.489932

C	1.622748	-2.456739	-3.245074
C	-1.470515	-1.701868	-3.125209
H	-0.247098	2.025278	0.358723
H	-2.488575	0.134523	-0.421792
H	-2.399607	1.178639	-2.625909
H	-3.733655	2.889819	-3.831546
H	-3.648942	5.268034	-3.107144
H	-2.234309	5.916472	-1.165540
H	-0.925323	4.188627	0.043189
H	-1.236857	1.967033	2.658580
H	-2.745727	3.271005	4.112478
H	-5.160145	3.472325	3.554635
H	-6.051851	2.361595	1.513228
H	-4.531790	1.055182	0.053012
H	1.204340	4.138471	-2.603067
H	2.181078	6.031535	-1.307662
H	3.522293	3.269740	1.698709
H	2.472408	1.390068	0.420773
H	3.133573	6.888142	0.751461
H	3.387185	5.806312	2.135801
H	4.630271	5.957288	0.893562
H	-1.359850	-2.127342	2.228176
H	-2.569092	-0.880841	2.517045
H	-4.638673	-1.399720	2.016261
H	-5.747646	-3.655412	-1.467153
H	-3.346101	-3.874707	-2.060182
H	-6.759004	-0.614723	1.599363
H	-8.202127	-1.668242	1.652476
H	-6.757802	-2.087887	2.615020
H	0.035896	-5.324524	1.023820
H	0.455804	-3.907416	1.989314
H	-1.242052	-4.255838	1.608141
H	2.555582	-3.840298	0.591356
H	2.502560	-4.906943	-0.818469
H	3.202914	-3.282594	-0.952689
H	2.653985	-2.305869	-2.922283
H	1.589289	-3.332982	-3.907616
H	1.327380	-1.576677	-3.816526
H	-1.631804	-2.376729	-3.975210
H	-2.447798	-1.374318	-2.762987
H	-0.924944	-0.823815	-3.480254
H	-0.534553	-0.030278	1.768111
C	1.634368	0.088952	2.982930
C	2.088974	-1.015121	2.008775

C	3.636545	-1.053441	2.251639
O	1.769836	-0.551923	0.752122
H	1.627212	-1.979334	2.268085
H	3.807550	-1.688174	3.133120
N	2.738396	0.852001	3.286815
H	2.678071	1.752775	3.749703
C	3.933856	0.364132	2.743566
O	0.487124	0.357202	3.325922
O	4.974867	0.984431	2.726838
C	4.491638	-1.550879	1.120821
C	5.247670	-2.717229	1.289434
C	4.522710	-0.898961	-0.119880
C	6.011927	-3.235117	0.242286
H	5.232587	-3.229286	2.248837
C	5.283981	-1.416329	-1.167948
H	3.925900	-0.014007	-0.285759
C	6.029620	-2.585263	-0.993372
H	6.590062	-4.142580	0.392520
H	5.282590	-0.903954	-2.125576
H	6.620286	-2.985303	-1.812862

----

a-RS-TS2

Gsol = -2979.41159059 Hartree

Rh	-0.909345	-1.334414	-0.773670
S	1.443350	0.195277	-2.272979
O	2.045302	-1.162136	-2.327044
O	0.840342	0.659773	-3.548498
O	-6.800605	1.626416	1.291774
N	0.400921	0.221977	-1.023795
N	-1.188406	-0.280064	0.947967
C	0.266614	1.368846	-0.101575
C	-1.075608	1.161214	0.632760
C	0.290842	2.750172	-0.722946
C	-0.471677	3.046386	-1.857469
C	-0.458210	4.331115	-2.399853
C	0.310805	5.335572	-1.806442
C	1.061779	5.048329	-0.664454
C	1.048661	3.762005	-0.126901
C	-1.200845	2.048891	1.852552
C	-0.343855	1.892525	2.950824
C	-0.475816	2.719230	4.066739
C	-1.461151	3.710099	4.098096
C	-2.316390	3.870805	3.006364

C	-2.185282	3.041043	1.891219
C	2.720076	1.362675	-1.830502
C	2.981594	2.448846	-2.662978
C	3.957949	3.367397	-2.287303
C	4.670726	3.219169	-1.088929
C	4.395700	2.109043	-0.277239
C	3.424272	1.181462	-0.640353
C	5.686782	4.250495	-0.666804
C	-2.433848	-0.641203	1.649374
C	-3.709115	-0.368756	0.868653
C	-4.659304	0.473443	1.454490
C	-5.849195	0.795921	0.791985
C	-6.099492	0.257817	-0.477680
C	-5.155193	-0.569622	-1.067672
C	-3.954318	-0.899809	-0.417641
C	-6.555934	2.242257	2.554091
C	-2.961434	-1.716055	-1.147345
C	-2.391454	-2.967704	-0.748801
C	-1.383068	-3.319799	-1.733827
C	-1.346466	-2.328022	-2.751813
C	-2.296026	-1.297767	-2.381399
C	-2.826565	-3.825038	0.398795
C	-0.539890	-4.552179	-1.669715
C	-0.540502	-2.361343	-4.011267
C	-2.598232	-0.071266	-3.176488
H	1.065418	1.316418	0.646810
H	-1.882513	1.419546	-0.064197
H	-1.048264	2.260143	-2.330750
H	-1.044132	4.547353	-3.289047
H	0.325989	6.334652	-2.232993
H	1.664166	5.822720	-0.197769
H	1.645922	3.531429	0.750185
H	0.417929	1.118495	2.934914
H	0.191934	2.589535	4.913797
H	-1.560863	4.352247	4.968567
H	-3.083583	4.639964	3.020962
H	-2.851455	3.162222	1.041459
H	2.407349	2.575545	-3.572718
H	4.162524	4.220636	-2.928302
H	4.946169	1.963744	0.648214
H	3.209913	0.340206	0.005711
H	6.212192	4.671461	-1.530108
H	5.198342	5.083788	-0.144787
H	6.428473	3.825315	0.016253

H	-2.344825	-1.698576	1.910784
H	-2.481761	-0.084810	2.591736
H	-4.440720	0.896033	2.426970
H	-7.023590	0.511173	-0.986220
H	-5.346234	-0.972018	-2.058186
H	-5.644501	2.851651	2.528338
H	-7.418370	2.881480	2.745139
H	-6.470430	1.494687	3.351500
H	-3.178031	-4.795112	0.026899
H	-2.014561	-4.009807	1.108653
H	-3.653553	-3.353372	0.935356
H	-0.376543	-4.867612	-0.636172
H	-1.042953	-5.375691	-2.193158
H	0.431777	-4.395319	-2.143696
H	0.367915	-2.953127	-3.883895
H	-1.133103	-2.808861	-4.820180
H	-0.239378	-1.356781	-4.312761
H	-3.303017	-0.312946	-3.983099
H	-3.057801	0.696395	-2.549281
H	-1.684983	0.328215	-3.623396
H	-0.182413	-0.654439	1.623888
C	0.795838	-3.530383	1.867153
C	1.175255	-2.144988	1.313561
C	2.642799	-2.311465	0.815546
O	0.845560	-1.102360	2.101715
H	0.574684	-2.131551	0.333383
H	2.736592	-1.940361	-0.209330
N	1.768695	-4.419939	1.438988
H	1.712899	-5.421548	1.593062
C	2.838518	-3.830532	0.768471
O	-0.194775	-3.827386	2.510485
O	3.754953	-4.446614	0.264910
C	3.729093	-1.694601	1.675247
C	4.934970	-1.322641	1.065710
C	3.593203	-1.534173	3.060147
C	5.977327	-0.778687	1.816894
H	5.050501	-1.451784	-0.006433
C	4.636213	-0.991078	3.812226
H	2.662272	-1.803015	3.544236
C	5.829154	-0.608574	3.195072
H	6.901326	-0.488660	1.324764
H	4.513141	-0.863538	4.884130
H	6.636558	-0.181728	3.783287

RS-pro

Gsol = -666.306190030 Hartree

H	-1.528016	-2.359684	-1.049227
C	-2.684087	-0.393072	0.030952
C	-1.337535	-1.052454	0.371651
C	-0.419672	0.133194	0.748819
O	-0.861971	-1.697011	-0.798797
H	-1.458156	-1.761666	1.198390
H	-0.469420	0.242137	1.839671
N	-2.461773	0.966988	-0.077392
H	-3.169865	1.617752	-0.400564
C	-1.155544	1.364001	0.201715
O	-3.732186	-0.968081	-0.187588
O	-0.730179	2.490350	0.065875
C	1.028608	0.033159	0.340937
C	2.002450	-0.264322	1.299465
C	1.421011	0.221230	-0.990469
C	3.346246	-0.377836	0.938105
H	1.707623	-0.407187	2.336083
C	2.762240	0.112756	-1.353268
H	0.673935	0.449426	-1.743570
C	3.729177	-0.188580	-0.390281
H	4.090815	-0.608578	1.694545
H	3.053314	0.263324	-2.388879
H	4.774290	-0.271516	-0.674389

RS-TS2

Gsol = -2979.38471867 Hartree

Rh	0.550937	-1.759316	-0.444077
S	-2.620302	-1.768184	-0.379277
O	-2.610491	-2.140550	-1.820062
O	-2.760995	-2.896759	0.570691
O	5.024881	0.565296	4.297083
N	-1.247917	-0.910838	-0.080186
N	1.075349	0.211003	-0.193716
C	-1.256255	0.422935	0.561590
C	0.218674	0.716811	0.913774
C	-2.088634	0.550146	1.822367
C	-2.058308	-0.441147	2.808619
C	-2.808494	-0.299184	3.975470
C	-3.589400	0.842687	4.171775
C	-3.610584	1.842060	3.196496

C	-2.861841	1.695325	2.029271
C	0.458248	2.169037	1.268799
C	0.103531	3.203083	0.395379
C	0.375006	4.530590	0.724848
C	0.988067	4.842536	1.940600
C	1.322850	3.818423	2.829166
C	1.059095	2.490103	2.491367
C	-4.001968	-0.670638	-0.104689
C	-4.203725	0.422507	-0.946579
C	-5.262274	1.287725	-0.676435
C	-6.121144	1.070625	0.408877
C	-5.908070	-0.054699	1.218250
C	-4.852444	-0.924934	0.972170
C	-7.229333	2.041781	0.727866
C	2.516642	0.412841	0.053736
C	3.103051	-0.312069	1.254240
C	3.795407	0.457475	2.195259
C	4.359136	-0.125086	3.335940
C	4.234310	-1.506475	3.538496
C	3.547066	-2.273392	2.609804
C	2.978020	-1.703893	1.457886
C	5.142406	1.978341	4.148920
C	2.220888	-2.582532	0.544474
C	2.458025	-2.818731	-0.846866
C	1.425622	-3.715623	-1.323795
C	0.545427	-4.035306	-0.254883
C	1.008068	-3.318894	0.911175
C	3.649093	-2.391536	-1.644990
C	1.369723	-4.247954	-2.713886
C	-0.580328	-5.019224	-0.288281
C	0.402982	-3.398778	2.272369
H	-1.605187	1.162642	-0.164144
H	0.460052	0.117980	1.800769
H	-1.472654	-1.337607	2.641654
H	-2.788697	-1.082127	4.728731
H	-4.179432	0.951168	5.077498
H	-4.217297	2.731862	3.339939
H	-2.890321	2.463401	1.262621
H	-0.385919	2.975026	-0.543085
H	0.112683	5.317254	0.024398
H	1.198113	5.877268	2.196365
H	1.786899	4.051867	3.783567
H	1.325178	1.692322	3.178817
H	-3.525143	0.632645	-1.765607

H	-5.417667	2.151463	-1.317248
H	-6.567818	-0.240938	2.061215
H	-4.667151	-1.778377	1.611865
H	-8.144225	1.520271	1.028750
H	-6.939907	2.695028	1.561467
H	-7.461947	2.681175	-0.128773
H	3.029537	0.108147	-0.862248
H	2.702075	1.484963	0.174434
H	3.863140	1.525967	2.035493
H	4.671220	-1.952168	4.425725
H	3.449131	-3.342760	2.772069
H	5.701212	2.237639	3.242026
H	4.156848	2.457987	4.117891
H	5.689702	2.324606	5.026038
H	3.362505	-1.960420	-2.608482
H	4.256063	-1.668014	-1.097106
H	4.282423	-3.262734	-1.854684
H	1.518281	-3.454710	-3.449390
H	2.167615	-4.992247	-2.842351
H	0.414228	-4.731740	-2.923060
H	-1.409933	-4.687059	0.335974
H	-0.963103	-5.144895	-1.303604
H	-0.232745	-5.997755	0.068176
H	0.673044	-2.527717	2.873791
H	-0.684988	-3.468838	2.203125
H	0.772514	-4.293419	2.790967
H	0.829557	0.820381	-1.352168
C	0.272652	-0.707681	-3.841389
C	1.366705	0.332872	-3.556938
C	0.661655	1.360463	-2.667639
O	1.804601	0.831496	-4.821892
H	2.204605	-0.173362	-3.055721
N	-0.908701	-0.188862	-3.381107
H	-1.737705	-0.763774	-3.233172
C	-0.763204	1.090083	-2.794893
O	0.423854	-1.793402	-4.380740
O	-1.735262	1.739723	-2.397859
C	1.204170	2.741901	-2.682923
C	0.404276	3.884552	-2.873688
C	2.588651	2.937165	-2.506583
C	0.962192	5.161329	-2.856611
H	-0.662944	3.759671	-3.013001
C	3.146818	4.216064	-2.496186
H	3.234645	2.074998	-2.360877

C	2.335377	5.338612	-2.663711
H	0.318997	6.025599	-3.001341
H	4.217579	4.332067	-2.351388
H	2.765463	6.335785	-2.649362
H	2.153341	1.724341	-4.655446

SR-TS2

Gsol = -2979.37922372 Hartree

Rh	-0.999982	-1.498863	-0.458644
S	1.946824	-1.534351	-1.630230
O	1.927850	-2.880039	-0.975248
O	1.756122	-1.551415	-3.100101
O	-5.806480	3.501276	-0.732225
N	0.841888	-0.637572	-0.851710
N	-1.049138	0.181317	0.691930
C	0.972215	0.801695	-0.548852
C	-0.445815	1.259966	-0.135761
C	1.483475	1.702371	-1.652259
C	1.019153	1.586734	-2.965802
C	1.495000	2.442395	-3.959764
C	2.429431	3.432677	-3.644586
C	2.881190	3.565120	-2.329370
C	2.410121	2.701993	-1.340736
C	-0.466999	2.636821	0.493935
C	0.212130	2.915511	1.685002
C	0.170338	4.193793	2.241069
C	-0.545051	5.213502	1.608731
C	-1.218312	4.946155	0.414498
C	-1.179412	3.663924	-0.135544
C	3.566747	-0.832582	-1.333016
C	4.308933	-1.253606	-0.227017
C	5.569447	-0.705478	-0.004867
C	6.105683	0.254379	-0.876034
C	5.354645	0.631973	-1.997070
C	4.092888	0.093550	-2.235042
C	7.451595	0.875402	-0.600239
C	-2.396392	0.510686	1.198829
C	-3.453234	0.778141	0.140967
C	-4.141675	1.993473	0.213044
C	-5.121773	2.328586	-0.727600
C	-5.429122	1.425695	-1.754563
C	-4.744662	0.221850	-1.833185
C	-3.755445	-0.127658	-0.898653

C	-5.483842	4.469096	0.264269
C	-3.018319	-1.391797	-1.107510
C	-2.929720	-2.523918	-0.235042
C	-2.046781	-3.497890	-0.862722
C	-1.579724	-2.975081	-2.093695
C	-2.149317	-1.649667	-2.250092
C	-3.720628	-2.758841	1.013037
C	-1.737599	-4.839788	-0.287848
C	-0.723182	-3.671127	-3.102623
C	-1.968702	-0.765482	-3.437578
H	1.632173	0.925278	0.315238
H	-1.040846	1.320090	-1.055542
H	0.314800	0.802696	-3.212943
H	1.140247	2.333283	-4.980860
H	2.803090	4.095944	-4.419486
H	3.607327	4.332346	-2.076228
H	2.772780	2.789633	-0.320309
H	0.772851	2.138207	2.185649
H	0.702161	4.387232	3.168257
H	-0.575103	6.209674	2.040926
H	-1.770525	5.734038	-0.090291
H	-1.708463	3.455129	-1.061074
H	3.914269	-2.003855	0.446432
H	6.146714	-1.032573	0.855358
H	5.759691	1.360502	-2.693546
H	3.522703	0.389044	-3.106594
H	7.352341	1.721948	0.091692
H	8.135909	0.156865	-0.137958
H	7.913278	1.253271	-1.517414
H	-2.694272	-0.321694	1.842170
H	-2.319344	1.392586	1.842703
H	-3.877878	2.687418	1.000607
H	-6.189278	1.690648	-2.481691
H	-4.976520	-0.469621	-2.637927
H	-5.698542	4.089877	1.270289
H	-4.429430	4.763866	0.204464
H	-6.118023	5.331482	0.057018
H	-3.091855	-3.118786	1.831767
H	-4.229110	-1.847216	1.334186
H	-4.487917	-3.521118	0.828312
H	-1.530857	-4.783726	0.783952
H	-2.601493	-5.503299	-0.428756
H	-0.875104	-5.295263	-0.777951
H	-0.011153	-2.981496	-3.558974

H	-0.148737	-4.478056	-2.643873
H	-1.352605	-4.102381	-3.891968
H	-2.130934	0.283100	-3.176523
H	-0.966535	-0.883289	-3.855471
H	-2.695136	-1.031683	-4.216793
H	-0.350061	-0.159486	1.785115
C	-0.413060	-3.163692	2.893796
C	0.485756	-2.183317	2.121268
C	0.311143	-0.838924	2.815858
O	1.760062	-2.681688	1.850971
H	-0.065897	-2.148273	1.126152
N	-1.180944	-2.395190	3.731027
H	-1.906225	-2.768710	4.332523
C	-0.763939	-1.045356	3.808862
O	-0.490518	-4.370846	2.741013
O	-1.308599	-0.256225	4.571333
C	1.459309	0.039361	3.169773
C	1.421343	0.904992	4.282213
C	2.588894	0.112501	2.334635
C	2.456244	1.807140	4.527048
H	0.559496	0.886799	4.936483
C	3.620327	1.017905	2.578642
H	2.653698	-0.549870	1.487454
C	3.560786	1.879971	3.674400
H	2.392038	2.463995	5.390927
H	4.467603	1.045050	1.898786
H	4.357823	2.593847	3.861629
H	1.816152	-2.862314	0.885251

RR-TS2

Gsol = -2979.39534219 Hartree

Rh	-0.809695	-1.194178	-1.086038
S	2.349282	-1.495370	-1.432234
O	2.027397	-2.910596	-1.043424
O	2.569431	-1.315430	-2.887682
O	-3.649001	5.165979	-1.300316
N	1.179885	-0.501010	-0.903529
N	-1.018325	0.174139	0.471854
C	1.383381	0.491295	0.163431
C	0.016087	1.204215	0.267908
C	2.451877	1.526252	-0.129279
C	2.546987	2.107848	-1.398038
C	3.485571	3.108371	-1.646503

C	4.338687	3.538645	-0.626644
C	4.242039	2.965127	0.642672
C	3.299805	1.966583	0.888338
C	-0.006413	2.334116	1.278984
C	-0.086649	2.103667	2.655149
C	-0.099682	3.171760	3.554342
C	-0.028354	4.485859	3.088367
C	0.054218	4.725266	1.714083
C	0.059357	3.655195	0.819530
C	3.876736	-1.054449	-0.618482
C	4.123190	-1.482908	0.684968
C	5.278599	-1.041314	1.328445
C	6.186109	-0.188143	0.688212
C	5.932298	0.186836	-0.639443
C	4.783859	-0.237484	-1.296147
C	7.395523	0.344760	1.412672
C	-2.374747	0.760077	0.526554
C	-2.731262	1.635393	-0.661743
C	-3.070651	2.969160	-0.415695
C	-3.342194	3.852004	-1.466382
C	-3.285008	3.390742	-2.788926
C	-2.941674	2.069662	-3.038312
C	-2.659600	1.174531	-1.993590
C	-3.639311	5.690856	0.025354
C	-2.243316	-0.201884	-2.319554
C	-2.872203	-1.418327	-1.859084
C	-2.097854	-2.516301	-2.373601
C	-1.027458	-2.015365	-3.175197
C	-1.100924	-0.569288	-3.130084
C	-4.194744	-1.524059	-1.165660
C	-2.391208	-3.954780	-2.096187
C	-0.079897	-2.826946	-4.000767
C	-0.185051	0.371204	-3.842618
H	1.602272	0.019687	1.133551
H	-0.146371	1.661333	-0.717512
H	1.900730	1.749353	-2.192461
H	3.556461	3.548395	-2.637570
H	5.075302	4.313101	-0.821243
H	4.903814	3.290778	1.440493
H	3.228373	1.515462	1.873576
H	-0.156438	1.095152	3.040057
H	-0.167680	2.970891	4.619911
H	-0.038206	5.316522	3.788438
H	0.113524	5.743197	1.338672

H	0.119542	3.842084	-0.248616
H	3.442971	-2.165303	1.180433
H	5.477778	-1.366535	2.346160
H	6.634278	0.834880	-1.156418
H	4.573787	0.071416	-2.312098
H	7.222187	1.380542	1.733251
H	7.623155	-0.245619	2.305144
H	8.279808	0.350480	0.766580
H	-3.074817	-0.067075	0.643202
H	-2.458053	1.369040	1.433275
H	-3.083538	3.315251	0.609863
H	-3.497204	4.081002	-3.598578
H	-2.888536	1.717406	-4.064238
H	-4.403900	5.211532	0.648367
H	-2.656542	5.564170	0.494794
H	-3.865213	6.753120	-0.073880
H	-4.128434	-2.052442	-0.212332
H	-4.609568	-0.531182	-0.976427
H	-4.905385	-2.066802	-1.801087
H	-2.904664	-4.071434	-1.138643
H	-3.041296	-4.363010	-2.880680
H	-1.475985	-4.551082	-2.072225
H	0.909435	-2.367993	-4.032362
H	0.030099	-3.835746	-3.594595
H	-0.459149	-2.917842	-5.027534
H	-0.223405	1.364795	-3.390257
H	0.842266	0.003793	-3.809337
H	-0.493053	0.465559	-4.892149
H	-0.881312	-0.682436	1.541945
C	1.124109	-2.910053	2.344791
C	-0.290889	-2.886635	1.760655
C	-0.975108	-1.706315	2.432958
O	-0.251516	-2.839500	0.322381
H	-0.763416	-3.840493	2.031603
N	1.140044	-2.004653	3.367534
H	1.935257	-1.855911	3.978150
C	-0.100798	-1.348222	3.562148
O	2.050639	-3.605754	1.961342
O	-0.283611	-0.601120	4.516418
C	-2.441966	-1.802017	2.686793
C	-3.102050	-0.866641	3.507438
C	-3.222623	-2.789276	2.061082
C	-4.480605	-0.928955	3.698516
H	-2.519225	-0.097934	3.999403

C	-4.603859	-2.853124	2.259412
H	-2.754078	-3.511551	1.399512
C	-5.243925	-1.921754	3.076709
H	-4.963103	-0.194756	4.338538
H	-5.178413	-3.632080	1.764771
H	-6.318634	-1.966814	3.226910
H	0.663910	-3.040056	-0.025136

----

a-SS-TS1

Gsol = -2979.39931081 Hartree

Rh	-0.447132	-1.417845	-0.452072
S	1.645418	0.631585	-1.934361
O	2.276446	-0.674195	-2.220842
O	1.121562	1.330419	-3.138228
O	-6.937735	1.055335	0.391326
N	0.538836	0.410193	-0.771504
N	-1.263037	-0.250056	1.098474
C	0.165899	1.502927	0.145017
C	-1.244310	1.176428	0.672598
C	0.139934	2.912150	-0.420131
C	-0.622365	3.227622	-1.549969
C	-0.685995	4.540696	-2.013533
C	0.008630	5.554964	-1.348386
C	0.765879	5.246929	-0.216728
C	0.827405	3.931742	0.242795
C	-1.679185	2.111917	1.778343
C	-1.012474	2.123500	3.011025
C	-1.415745	2.999563	4.018912
C	-2.485319	3.872982	3.805475
C	-3.152739	3.864768	2.579186
C	-2.751276	2.985262	1.572816
C	2.863538	1.727475	-1.211622
C	3.235138	2.889051	-1.885833
C	4.157182	3.751452	-1.297826
C	4.708896	3.470964	-0.040198
C	4.317825	2.294267	0.614824
C	3.404365	1.417795	0.036583
C	5.717957	4.400861	0.586731
C	-2.605480	-0.701723	1.560043
C	-3.691649	-0.575145	0.516018
C	-4.827511	0.157912	0.846063
C	-5.889780	0.316249	-0.056284
C	-5.807545	-0.266245	-1.324899

C	-4.664140	-0.989427	-1.662907
C	-3.602272	-1.164962	-0.769477
C	-8.033774	1.266450	-0.494168
C	-2.412829	-1.906651	-1.232358
C	-1.853228	-3.105056	-0.643195
C	-0.678815	-3.440523	-1.408626
C	-0.508798	-2.477994	-2.449391
C	-1.587568	-1.517806	-2.343376
C	-2.512518	-3.959817	0.397474
C	0.164949	-4.657453	-1.204394
C	0.469083	-2.564866	-3.576329
C	-1.815873	-0.359339	-3.259108
H	0.849037	1.499901	1.003962
H	-1.943668	1.254925	-0.163085
H	-1.140876	2.437616	-2.081759
H	-1.274061	4.772785	-2.897276
H	-0.038473	6.577484	-1.712737
H	1.313805	6.027212	0.304016
H	1.429995	3.686314	1.112205
H	-0.182035	1.444946	3.184844
H	-0.893360	3.000890	4.971345
H	-2.797045	4.554887	4.591421
H	-3.985905	4.539763	2.405883
H	-3.269161	2.976836	0.618061
H	2.775735	3.121019	-2.839309
H	4.441093	4.664408	-1.814737
H	4.736677	2.056165	1.588431
H	3.108395	0.513858	0.554845
H	5.637220	4.399440	1.678650
H	6.742057	4.094449	0.335830
H	5.588698	5.428547	0.232762
H	-2.478438	-1.728996	1.896891
H	-2.887195	-0.105980	2.433338
H	-4.903853	0.641282	1.814392
H	-6.606713	-0.162038	-2.048007
H	-4.597780	-1.435909	-2.650560
H	-8.505308	0.317495	-0.776073
H	-8.748301	1.878517	0.056947
H	-7.716288	1.798717	-1.398756
H	-3.495126	-3.552781	0.651347
H	-2.661034	-4.975135	0.011552
H	-1.936240	-4.030657	1.323159
H	-0.194134	-5.469406	-1.850544
H	1.210909	-4.468828	-1.457003

H	0.117989	-5.007528	-0.172257
H	0.694709	-1.580414	-3.984822
H	1.411727	-3.010590	-3.255183
H	0.044969	-3.189975	-4.374693
H	-2.497722	0.363957	-2.805195
H	-0.877462	0.147938	-3.493973
H	-2.269072	-0.704372	-4.197838
H	-0.595569	-0.355678	1.882354
C	0.236058	-2.945410	2.528715
C	1.218127	-1.807953	2.145050
C	2.590918	-2.547985	2.064479
O	1.078524	-0.653798	2.613064
H	0.806962	-1.828088	0.691823
N	0.869885	-4.135820	2.220950
H	0.417765	-5.041322	2.295418
C	2.193626	-3.996131	1.795928
O	-0.869377	-2.839178	3.026716
O	2.863028	-4.899698	1.342974
H	2.906800	-2.543294	3.120054
C	3.720551	-2.002320	1.244531
C	4.768281	-1.327652	1.887157
C	3.766922	-2.168205	-0.141620
C	5.845735	-0.829553	1.156747
H	4.737365	-1.197482	2.965916
C	4.844946	-1.671326	-0.873223
H	2.950650	-2.661687	-0.654407
C	5.886665	-1.005039	-0.228272
H	6.649145	-0.305676	1.666584
H	4.852698	-1.785536	-1.951608
H	6.721377	-0.613675	-0.802666

----

a-SR-TS1

Gsol = -2979.39714990 Hartree

Rh	0.345112	-1.340277	-0.351269
S	1.106266	1.508497	-1.682006
O	2.485634	0.968861	-1.537940
O	0.603457	1.511729	-3.080712
O	-6.384479	-3.249785	-0.069531
N	0.144491	0.730916	-0.646294
N	-1.092415	-0.854337	1.106768
C	-0.935060	1.387819	0.103384
C	-1.901298	0.272012	0.562754
C	-1.742954	2.440306	-0.633160

C	-2.028055	3.657780	-0.010078
C	-2.799215	4.621964	-0.657404
C	-3.297787	4.372294	-1.937693
C	-3.025610	3.151487	-2.560864
C	-2.255582	2.188244	-1.909840
C	-2.945700	0.772401	1.534546
C	-2.589333	1.209287	2.817204
C	-3.566887	1.671492	3.699188
C	-4.907609	1.705961	3.307871
C	-5.267794	1.274808	2.029342
C	-4.289788	0.808335	1.150119
C	1.123812	3.211349	-1.133555
C	0.678096	4.218501	-1.986771
C	0.653006	5.532885	-1.529584
C	1.059902	5.855119	-0.227388
C	1.520236	4.824638	0.605132
C	1.557285	3.506243	0.159230
C	0.974366	7.274893	0.274160
C	-1.906539	-2.020236	1.547983
C	-2.771895	-2.618210	0.459628
C	-4.148318	-2.673336	0.709779
C	-5.037450	-3.189269	-0.238003
C	-4.539427	-3.670152	-1.456229
C	-3.177381	-3.608977	-1.710831
C	-2.268936	-3.086955	-0.773752
C	-6.932464	-2.710254	1.130636
C	-0.847306	-2.977002	-1.162381
C	0.297905	-3.513607	-0.444795
C	1.479905	-3.089254	-1.146759
C	1.088265	-2.267978	-2.250657
C	-0.363779	-2.217656	-2.277916
C	0.291064	-4.478171	0.701639
C	2.865217	-3.483347	-0.749817
C	1.992346	-1.691349	-3.294255
C	-1.178260	-1.476612	-3.290242
H	-0.524231	1.859340	1.009041
H	-2.399549	-0.120842	-0.326572
H	-1.624677	3.856844	0.978344
H	-3.003387	5.569463	-0.166693
H	-3.892383	5.124559	-2.448367
H	-3.409458	2.951254	-3.557392
H	-2.020901	1.251679	-2.402734
H	-1.550095	1.182530	3.134113
H	-3.280976	2.005518	4.692410

H	-5.666591	2.067124	3.995712
H	-6.307504	1.301761	1.715848
H	-4.567394	0.470832	0.155813
H	0.332007	3.960243	-2.980124
H	0.298071	6.320628	-2.188601
H	1.853560	5.058863	1.612589
H	1.905032	2.714300	0.811997
H	1.244317	7.991551	-0.508411
H	-0.048988	7.512819	0.592767
H	1.633233	7.437357	1.132461
H	-1.196503	-2.743438	1.952084
H	-2.552627	-1.701062	2.371185
H	-4.517061	-2.281789	1.649109
H	-5.231840	-4.072532	-2.188103
H	-2.797191	-3.972148	-2.660769
H	-6.575591	-3.256680	2.011812
H	-6.684328	-1.647265	1.237623
H	-8.013040	-2.827050	1.043264
H	-0.725775	-4.672823	1.051177
H	0.721463	-5.438573	0.391250
H	0.891555	-4.101703	1.537303
H	3.024830	-3.319376	0.321521
H	3.018553	-4.551175	-0.954984
H	3.620953	-2.917165	-1.295760
H	1.605522	-0.743404	-3.671883
H	2.989491	-1.503586	-2.892297
H	2.084036	-2.390354	-4.136710
H	-1.248103	-2.049152	-4.224553
H	-2.194936	-1.307583	-2.926243
H	-0.719406	-0.510294	-3.515682
H	-0.568947	-0.538179	1.931461
C	1.770823	0.087374	2.712102
C	2.522467	-0.976409	1.866753
C	3.645350	-0.165378	1.149554
O	2.649315	-2.133117	2.309239
H	1.494360	-0.963412	0.769504
N	2.622506	1.163309	2.808845
H	2.443225	1.957928	3.415469
C	3.810456	1.045151	2.061334
O	0.672538	0.001386	3.244006
O	4.725455	1.833842	2.128747
H	3.207059	0.261602	0.235911
C	4.899147	-0.901002	0.785809
C	5.353941	-0.854367	-0.537726

C	5.613770	-1.649676	1.728619
C	6.503136	-1.549563	-0.917151
H	4.795436	-0.273618	-1.266621
C	6.761644	-2.345931	1.349950
H	5.261576	-1.697690	2.753709
C	7.208068	-2.299727	0.026423
H	6.845630	-1.505935	-1.947249
H	7.307594	-2.926817	2.088089
H	8.101578	-2.844147	-0.266037

----

a-RR-TS1

Gsol = -2979.39196779 Hartree

Rh	-1.099326	-0.337705	-0.734543
S	1.578892	-2.100883	-1.311205
O	0.728201	-3.300231	-1.076732
O	1.806354	-1.803831	-2.752349
O	-0.558017	6.637863	-0.831069
N	0.958567	-0.808461	-0.546167
N	-0.545972	0.941034	0.816625
C	1.709401	-0.008373	0.431657
C	0.892772	1.286264	0.661306
C	3.104390	0.429474	0.015226
C	3.333063	0.977252	-1.251514
C	4.602831	1.430747	-1.605910
C	5.657596	1.346149	-0.693205
C	5.431363	0.809611	0.575803
C	4.159388	0.358351	0.927265
C	1.413647	2.095115	1.827162
C	1.325918	1.599177	3.134786
C	1.809191	2.353260	4.204493
C	2.386583	3.605518	3.978072
C	2.478717	4.102228	2.676181
C	1.991372	3.349073	1.607083
C	3.207262	-2.449920	-0.638989
C	3.347696	-3.027698	0.621353
C	4.629820	-3.254437	1.120377
C	5.769012	-2.921670	0.377275
C	5.595530	-2.379626	-0.904329
C	4.325484	-2.142279	-1.416555
C	7.152277	-3.107478	0.947771
C	-1.434441	2.129793	0.938305
C	-1.366836	3.071352	-0.243983
C	-1.019018	4.400949	0.020310

C	-0.911915	5.339202	-1.011746
C	-1.165834	4.941748	-2.331404
C	-1.500597	3.622237	-2.597094
C	-1.608099	2.665466	-1.573632
C	-0.231277	7.065433	0.488969
C	-1.895289	1.265464	-1.950417
C	-3.005118	0.454638	-1.501195
C	-2.840691	-0.852358	-2.088771
C	-1.646953	-0.865761	-2.864435
C	-1.054822	0.456805	-2.794150
C	-4.224718	0.941434	-0.784810
C	-3.821178	-1.975962	-1.999791
C	-1.198366	-1.990352	-3.741159
C	0.172432	0.901201	-3.518987
H	1.779172	-0.541201	1.387653
H	0.958243	1.884788	-0.250423
H	2.519450	1.014230	-1.967080
H	4.771310	1.844316	-2.596621
H	6.649136	1.692661	-0.970906
H	6.246435	0.735615	1.290417
H	3.984802	-0.068529	1.910147
H	0.874749	0.627241	3.317809
H	1.736362	1.961914	5.215072
H	2.763242	4.190069	4.812461
H	2.929614	5.073272	2.492034
H	2.062580	3.731600	0.592947
H	2.472453	-3.268310	1.210758
H	4.745960	-3.693082	2.108158
H	6.467581	-2.125931	-1.501035
H	4.189820	-1.703724	-2.396442
H	7.847566	-3.495501	0.195545
H	7.556469	-2.148106	1.297048
H	7.147174	-3.794235	1.799547
H	-2.440495	1.735418	1.094668
H	-1.154576	2.679233	1.842006
H	-0.808691	4.685019	1.043542
H	-1.082827	5.673221	-3.128216
H	-1.684345	3.316403	-3.622564
H	0.609550	6.490575	0.895635
H	0.051725	8.114632	0.400242
H	-1.092747	6.974337	1.161032
H	-5.065314	1.008080	-1.487509
H	-4.518751	0.272936	0.024370
H	-4.066586	1.940374	-0.371204

H	-3.327480	-2.933691	-1.822231
H	-4.540873	-1.813531	-1.198580
H	-4.371643	-2.050613	-2.947321
H	-1.634126	-1.871674	-4.742905
H	-0.112117	-2.014379	-3.827614
H	-1.529049	-2.952004	-3.341631
H	0.582025	1.810035	-3.071417
H	0.929679	0.115062	-3.498816
H	-0.071751	1.125485	-4.566119
H	-0.686362	0.394738	1.688144
C	-0.418292	-2.693291	1.809510
C	-1.564757	-1.711933	2.068373
C	-2.830017	-2.615495	2.002026
O	-1.416079	-0.716331	2.821970
H	-1.549940	-1.310433	0.625941
H	-2.838425	-3.099129	2.994797
N	-0.973781	-3.786498	1.160924
H	-0.410669	-4.323187	0.505994
C	-2.357605	-3.708489	1.034338
O	0.739945	-2.553155	2.144350
O	-3.009970	-4.422310	0.294852
C	-4.175852	-1.955422	1.809244
C	-5.221796	-2.570226	1.107824
C	-4.420784	-0.728692	2.447739
C	-6.472230	-1.953817	1.018490
H	-5.049168	-3.524824	0.628168
C	-5.674105	-0.121168	2.364133
H	-3.619157	-0.256760	3.002885
C	-6.704753	-0.727653	1.643353
H	-7.267865	-2.440900	0.461600
H	-5.842051	0.830298	2.861211
H	-7.680270	-0.254582	1.576553

1a”

Gsol = -704.353786409 Hartree

C	-2.551847	-0.376843	-0.013917
C	-1.277258	0.439325	0.015709
C	-0.192393	-0.389914	-0.022672
O	-1.232256	1.768264	0.072708
N	-2.128212	-1.688738	-0.065232
H	-2.747393	-2.486934	-0.110970
C	-0.728650	-1.785282	-0.084072
O	-3.705082	0.012300	0.003026

O	-0.133857	-2.845746	-0.145841
C	1.232907	-0.053119	-0.014676
C	2.208105	-1.034940	0.254387
C	1.665754	1.264816	-0.270395
C	3.561614	-0.706227	0.270127
H	1.896203	-2.053326	0.443951
C	3.021085	1.583876	-0.254934
H	0.936365	2.034812	-0.485055
C	3.977205	0.602528	0.016744
H	4.295122	-1.478900	0.482690
H	3.331058	2.605137	-0.457941
H	5.033529	0.855088	0.030116
C	-2.437737	2.553116	0.183656
H	-2.993924	2.282223	1.082609
H	-2.087496	3.582910	0.247985
H	-3.069139	2.417595	-0.695647

----

b-int1

Gsol = -3018.64811909 Hartree

Rh	-0.420216	-1.384115	-1.111887
S	1.688143	0.539698	-2.783072
O	1.826754	-0.765781	-3.456274
O	1.561815	1.730065	-3.654805
O	-6.423872	1.410159	1.261669
N	0.440281	0.450379	-1.737636
N	-0.781363	-0.205278	0.597209
C	0.343321	1.523562	-0.733842
C	-0.902136	1.201874	0.121397
C	0.182268	2.934251	-1.271664
C	-0.760039	3.217321	-2.265432
C	-0.983488	4.530829	-2.671581
C	-0.264160	5.579158	-2.088309
C	0.677927	5.302865	-1.096990
C	0.895993	3.984322	-0.690500
C	-1.151211	2.194000	1.236283
C	-0.356713	2.216290	2.387326
C	-0.599584	3.147019	3.397870
C	-1.638375	4.070528	3.266091
C	-2.434788	4.056093	2.117962
C	-2.194043	3.118752	1.113464
C	3.216799	0.798703	-1.868760
C	3.944510	-0.303182	-1.413232
C	5.151114	-0.096096	-0.746915

C	5.640674	1.197194	-0.513239
C	4.897949	2.286180	-0.989421
C	3.692502	2.095354	-1.664443
C	6.914596	1.409641	0.264273
C	-1.970330	-0.664181	1.376656
C	-3.285625	-0.467776	0.654396
C	-4.234199	0.337692	1.297154
C	-5.470185	0.614478	0.706279
C	-5.769938	0.062281	-0.545529
C	-4.831347	-0.734592	-1.185488
C	-3.577925	-1.015722	-0.614553
C	-6.118954	2.051872	2.497001
C	-2.602032	-1.798632	-1.402347
C	-1.905605	-3.003822	-0.956525
C	-0.980370	-3.368916	-1.992423
C	-1.065483	-2.395380	-3.033964
C	-2.097821	-1.436178	-2.675891
C	-2.352836	-3.900636	0.158045
C	-0.144872	-4.612790	-2.014714
C	-0.405920	-2.473970	-4.373998
C	-2.457044	-0.237172	-3.495692
H	1.214626	1.516908	-0.061679
H	-1.762019	1.210547	-0.551562
H	-1.299919	2.398405	-2.728651
H	-1.715061	4.739383	-3.447510
H	-0.437069	6.603153	-2.407410
H	1.240811	6.110591	-0.637403
H	1.616535	3.768980	0.094204
H	0.455639	1.510279	2.507002
H	0.027684	3.146474	4.284335
H	-1.826649	4.796702	4.051595
H	-3.242658	4.773381	2.004158
H	-2.813233	3.106386	0.221541
H	3.576406	-1.305704	-1.602359
H	5.731184	-0.951897	-0.412234
H	5.268928	3.295824	-0.833077
H	3.131456	2.938709	-2.049336
H	6.701371	1.473601	1.339714
H	7.615774	0.581773	0.119212
H	7.412170	2.339825	-0.026903
H	-1.787810	-1.711371	1.611551
H	-2.002178	-0.116837	2.321829
H	-3.977139	0.770847	2.255140
H	-6.731584	0.275778	-1.000491

H	-5.065326	-1.154850	-2.158892
H	-5.959865	1.319689	3.297940
H	-5.230073	2.687680	2.405436
H	-6.987215	2.667252	2.735256
H	-1.530906	-4.501165	0.551337
H	-2.812208	-3.343442	0.977997
H	-3.117158	-4.595387	-0.217196
H	0.152349	-4.912446	-1.007206
H	-0.709085	-5.441675	-2.463639
H	0.761706	-4.465647	-2.607276
H	-0.229318	-1.482063	-4.790487
H	0.558445	-2.980910	-4.318162
H	-1.050142	-3.033327	-5.067513
H	-3.068714	0.463198	-2.921241
H	-1.551798	0.285004	-3.821785
H	-3.022203	-0.523609	-4.391647
H	0.030157	-0.276724	1.210830
C	0.679977	-4.088001	2.074348
C	1.786903	-3.056963	2.044800
C	1.439509	-1.988794	2.815101
O	2.864621	-3.403615	1.356795
H	0.961964	-1.832855	-0.474557
N	-0.274912	-3.566034	2.928130
H	-1.134094	-4.033803	3.185206
C	0.113619	-2.313373	3.423408
O	0.634855	-5.146669	1.480618
O	-0.555387	-1.635051	4.180769
C	2.051865	-0.666790	2.986240
C	2.069930	-0.034374	4.242641
C	2.566753	0.020485	1.873105
C	2.619766	1.238266	4.381191
H	1.653016	-0.545142	5.102598
C	3.121460	1.292441	2.017763
H	2.499252	-0.428594	0.888164
C	3.152994	1.902735	3.272024
H	2.633141	1.712801	5.357898
H	3.511539	1.804452	1.146541
H	3.577325	2.895890	3.383829
C	4.121865	-2.771939	1.658274
H	4.245894	-2.688335	2.741478
H	4.881519	-3.429078	1.236322
H	4.182012	-1.784397	1.201265

----

b-R-TS1

Gsol = -3018.62225158 Hartree

Rh	-0.745649	-1.561626	-0.607942
S	1.895725	-0.710214	-2.352614
O	1.924427	-2.187798	-2.338500
O	1.795588	-0.068355	-3.686079
O	-6.330022	2.613615	0.328160
N	0.705203	-0.231567	-1.358515
N	-0.968796	0.059456	0.692326
C	0.698168	1.111729	-0.762444
C	-0.698030	1.286440	-0.118090
C	0.936692	2.298878	-1.678055
C	0.263343	2.432658	-2.895328
C	0.434467	3.576302	-3.674228
C	1.278775	4.602381	-3.240861
C	1.949327	4.477922	-2.022471
C	1.775979	3.330997	-1.247016
C	-0.850525	2.568592	0.665950
C	-0.066822	2.823927	1.796229
C	-0.222789	4.011083	2.510623
C	-1.160376	4.960466	2.097258
C	-1.941012	4.715252	0.964973
C	-1.787718	3.522571	0.256703
C	3.464620	-0.167282	-1.666951
C	4.136992	-1.011810	-0.779754
C	5.350797	-0.602765	-0.235550
C	5.907930	0.643835	-0.561406
C	5.224152	1.462755	-1.469270
C	4.010111	1.063429	-2.029079
C	7.199475	1.093742	0.074546
C	-2.272022	0.062067	1.416866
C	-3.475870	0.260148	0.524255
C	-4.332695	1.318312	0.849786
C	-5.470844	1.589641	0.084632
C	-5.762873	0.780922	-1.021830
C	-4.910791	-0.263015	-1.350329
C	-3.760748	-0.549195	-0.594529
C	-6.026862	3.498829	1.403862
C	-2.867620	-1.623715	-1.081304
C	-2.504388	-2.852639	-0.406921
C	-1.585740	-3.558601	-1.276035
C	-1.364931	-2.767842	-2.438946
C	-2.167711	-1.568718	-2.332295
C	-3.165517	-3.423412	0.808694

C	-1.045144	-4.929765	-1.031727
C	-0.587166	-3.156754	-3.655101
C	-2.245401	-0.495087	-3.368145
H	1.441408	1.168935	0.044697
H	-1.436912	1.277003	-0.921944
H	-0.375524	1.628702	-3.238568
H	-0.087201	3.665305	-4.623161
H	1.413609	5.491670	-3.850169
H	2.607005	5.270460	-1.676565
H	2.296010	3.230992	-0.298413
H	0.670598	2.102500	2.123777
H	0.393990	4.190199	3.386279
H	-1.280231	5.886763	2.651693
H	-2.667209	5.451365	0.631883
H	-2.398165	3.328699	-0.620417
H	3.700393	-1.969156	-0.520524
H	5.873670	-1.256664	0.457635
H	5.648139	2.424078	-1.746973
H	3.497433	1.692758	-2.745341
H	7.032762	1.404862	1.114164
H	7.937290	0.284422	0.095115
H	7.635023	1.942848	-0.460322
H	-2.319489	-0.889961	1.947916
H	-2.250847	0.855882	2.168234
H	-4.080035	1.943980	1.695922
H	-6.646205	0.994846	-1.614210
H	-5.131613	-0.877298	-2.218111
H	-6.035996	2.973210	2.366153
H	-5.050665	3.977473	1.260522
H	-6.811205	4.256244	1.395747
H	-2.490158	-4.086905	1.352950
H	-3.513456	-2.640006	1.486522
H	-4.044267	-4.012650	0.513819
H	-0.803213	-5.083830	0.021448
H	-1.792427	-5.680086	-1.324727
H	-0.141353	-5.106406	-1.619288
H	-0.107967	-2.291566	-4.116327
H	0.195737	-3.877198	-3.419303
H	-1.267904	-3.606315	-4.391407
H	-2.639243	0.433543	-2.947725
H	-1.256231	-0.299035	-3.790250
H	-2.907331	-0.796091	-4.190737
H	-0.238780	-0.028788	1.405711
C	0.051336	-3.449020	2.359601

C	1.050238	-2.366689	1.958391
C	0.907644	-1.311529	2.880634
O	2.214212	-2.757007	1.348598
H	0.320102	-2.084894	0.607947
N	-0.808469	-2.830106	3.240036
H	-1.557625	-3.304730	3.727829
C	-0.313845	-1.568975	3.652819
O	0.022564	-4.611667	2.000343
O	-0.888612	-0.883436	4.490426
C	1.730793	-0.116463	3.022681
C	1.610346	0.744056	4.137108
C	2.672277	0.218595	2.027680
C	2.395103	1.890471	4.234545
H	0.887563	0.511143	4.907953
C	3.447141	1.372004	2.127527
H	2.785568	-0.427456	1.169198
C	3.311813	2.218580	3.230053
H	2.284412	2.537561	5.100366
H	4.149632	1.604461	1.333530
H	3.911860	3.120286	3.307798
C	3.259899	-3.127054	2.264522
H	2.933237	-3.951889	2.906479
H	4.093273	-3.454778	1.642078
H	3.564492	-2.272692	2.876210

----

b-R-int2

Gsol = -3018.63602771 Hartree

Rh	-0.799206	-1.578514	-0.470904
S	2.268712	-1.586059	-1.506776
O	2.163550	-2.988022	-1.035702
O	2.307780	-1.433643	-2.985866
O	-5.125630	3.783241	-2.011886
N	1.095005	-0.731725	-0.793910
N	-0.996145	0.288460	0.470886
C	1.242638	0.689942	-0.416657
C	-0.189528	1.258497	-0.330770
C	2.074708	1.579478	-1.319032
C	1.862146	1.637285	-2.699835
C	2.621228	2.495345	-3.495166
C	3.594541	3.313727	-2.916380
C	3.800377	3.271830	-1.535878
C	3.042245	2.409716	-0.745486
C	-0.265702	2.676027	0.191612

C	0.149381	2.999076	1.488929
C	0.028450	4.303686	1.966481
C	-0.494497	5.305638	1.145236
C	-0.892687	4.994636	-0.157243
C	-0.783753	3.684755	-0.627280
C	3.806438	-0.915134	-0.885192
C	4.081620	-0.996398	0.480845
C	5.271670	-0.459468	0.964772
C	6.191625	0.157556	0.103205
C	5.891032	0.218036	-1.263691
C	4.705955	-0.315635	-1.763699
C	7.483257	0.724127	0.637387
C	-2.408322	0.706821	0.704925
C	-3.176214	0.991467	-0.561559
C	-3.807357	2.234918	-0.670158
C	-4.508678	2.588113	-1.827249
C	-4.581022	1.680642	-2.893981
C	-3.950206	0.450301	-2.790409
C	-3.247313	0.079978	-1.630932
C	-5.022721	4.756621	-0.974485
C	-2.578006	-1.234388	-1.615049
C	-2.860586	-2.349774	-0.749928
C	-2.007901	-3.436203	-1.170022
C	-1.154682	-2.989701	-2.215797
C	-1.506521	-1.616958	-2.510912
C	-3.972289	-2.446213	0.246746
C	-2.118324	-4.837224	-0.682273
C	-0.196904	-3.829639	-2.998479
C	-0.951676	-0.799394	-3.629464
H	1.670729	0.742114	0.588906
H	-0.615638	1.240796	-1.336518
H	1.124641	0.988424	-3.154317
H	2.454782	2.522673	-4.568536
H	4.188277	3.978438	-3.537568
H	4.555561	3.902960	-1.076086
H	3.210701	2.367334	0.326536
H	0.580353	2.232072	2.120165
H	0.343482	4.526831	2.981693
H	-0.587849	6.322653	1.515352
H	-1.290144	5.769450	-0.806932
H	-1.109097	3.441058	-1.634481
H	3.371843	-1.463204	1.155766
H	5.490490	-0.515871	2.027622
H	6.587157	0.701576	-1.943489

H	4.455859	-0.243708	-2.815037
H	7.356248	1.120963	1.649784
H	8.259663	-0.050452	0.685949
H	7.861194	1.526925	-0.003211
H	-2.862568	-0.080645	1.308431
H	-2.397581	1.609655	1.321231
H	-3.714382	2.933590	0.150991
H	-5.122219	1.962819	-3.790773
H	-4.001710	-0.246739	-3.621443
H	-5.492395	4.401936	-0.049509
H	-3.975639	5.014026	-0.775702
H	-5.554119	5.636212	-1.338827
H	-3.612628	-2.820943	1.208844
H	-4.438171	-1.471127	0.406296
H	-4.748887	-3.131312	-0.116185
H	-2.487124	-4.881348	0.342230
H	-2.834769	-5.367894	-1.324787
H	-1.164724	-5.365482	-0.734612
H	0.617138	-3.227021	-3.403079
H	0.245682	-4.610007	-2.376902
H	-0.725238	-4.310221	-3.833277
H	-1.146185	0.264133	-3.472263
H	0.123979	-0.959111	-3.727305
H	-1.433292	-1.089397	-4.572702
H	-0.600137	0.187164	1.423562
C	0.021600	1.351010	4.561917
C	0.549294	0.083199	3.841780
C	-0.703399	-0.570717	3.323796
O	1.494805	0.422416	2.824266
H	1.072278	-0.547397	4.582833
N	-1.333731	1.222400	4.623984
H	-1.947699	1.942815	4.984041
C	-1.829971	0.085185	3.897243
O	0.686397	2.298082	4.965183
O	-3.045665	-0.142266	3.831714
C	-0.744518	-1.889401	2.751046
C	-1.736814	-2.839901	3.113640
C	0.263388	-2.323618	1.830624
C	-1.597807	-4.173223	2.765193
H	-2.551623	-2.518967	3.751596
C	0.386963	-3.690185	1.488550
H	1.073365	-1.645522	1.591135
C	-0.514934	-4.619139	1.981014
H	-2.327034	-4.893675	3.129001

H	1.201696	-3.980301	0.835026
H	-0.413106	-5.672086	1.738835
C	2.795405	0.733589	3.322165
H	2.763695	1.587369	4.002645
H	3.223806	-0.135388	3.842707
H	3.410638	0.968179	2.452461

----

b-SR-TS2

Gsol = -3018.61785454 Hartree

Rh	-0.978240	-1.568519	-0.417608
S	1.789798	-1.623637	-1.964247
O	1.702281	-2.997488	-1.423530
O	1.602656	-1.477463	-3.426079
O	-6.322562	2.883972	-0.292282
N	0.734029	-0.722557	-1.084675
N	-0.985246	0.215595	0.588533
C	0.858993	0.744421	-0.907709
C	-0.540159	1.203342	-0.436560
C	1.281568	1.560774	-2.109677
C	0.654794	1.416355	-3.351239
C	1.032406	2.216484	-4.429206
C	2.034745	3.177816	-4.272817
C	2.655083	3.336042	-3.031632
C	2.278915	2.529440	-1.957831
C	-0.618744	2.657772	-0.020537
C	0.290867	3.239772	0.868036
C	0.169339	4.581081	1.231156
C	-0.858217	5.364467	0.701990
C	-1.761196	4.797941	-0.200544
C	-1.637479	3.455023	-0.558135
C	3.408698	-0.987988	-1.559343
C	4.008061	-1.387095	-0.360847
C	5.254564	-0.868967	-0.024109
C	5.913593	0.040788	-0.866918
C	5.294966	0.410284	-2.067860
C	4.046911	-0.099966	-2.422807
C	7.264243	0.592368	-0.484401
C	-2.271344	0.531863	1.238653
C	-3.514180	0.557918	0.365715
C	-4.324807	1.696538	0.441784
C	-5.504159	1.799717	-0.302737
C	-5.889388	0.739571	-1.135048
C	-5.087375	-0.388560	-1.216834

C	-3.900265	-0.507567	-0.473724
C	-5.943277	4.001625	0.507409
C	-3.071519	-1.712011	-0.693534
C	-2.725417	-2.751200	0.222286
C	-1.848338	-3.683928	-0.479491
C	-1.653080	-3.232311	-1.807920
C	-2.370834	-1.981885	-1.947555
C	-3.276385	-2.955000	1.596928
C	-1.271814	-4.914831	0.134269
C	-0.903982	-3.917374	-2.905792
C	-2.467073	-1.166867	-3.193623
H	1.577363	0.935384	-0.104038
H	-1.208743	1.094012	-1.299553
H	-0.108125	0.657294	-3.478484
H	0.547812	2.087172	-5.392949
H	2.329894	3.798901	-5.113854
H	3.434432	4.081469	-2.900928
H	2.768154	2.640763	-0.994337
H	1.098991	2.653539	1.278856
H	0.885277	5.007801	1.927312
H	-0.950378	6.409599	0.983738
H	-2.556208	5.400670	-0.631129
H	-2.342479	3.016136	-1.258082
H	3.503033	-2.081924	0.301732
H	5.718990	-1.166177	0.912138
H	5.791057	1.112468	-2.731943
H	3.567907	0.191106	-3.349581
H	8.037576	-0.183004	-0.550950
H	7.559522	1.416562	-1.139980
H	7.263842	0.958113	0.548653
H	-2.397064	-0.212835	2.029302
H	-2.178546	1.502379	1.736858
H	-4.004130	2.512762	1.076185
H	-6.804021	0.825211	-1.712002
H	-5.380910	-1.203593	-1.872133
H	-5.902969	3.734824	1.570077
H	-4.970410	4.400711	0.196456
H	-6.715255	4.755398	0.349956
H	-2.523275	-3.370014	2.270427
H	-3.657970	-2.020218	2.014179
H	-4.113905	-3.663874	1.556247
H	-0.861732	-4.702525	1.125302
H	-2.059590	-5.671245	0.250756
H	-0.480889	-5.335499	-0.489114

H	-0.371666	-3.197262	-3.530829
H	-0.163000	-4.610800	-2.508026
H	-1.604343	-4.475410	-3.540986
H	-2.692999	-0.122886	-2.963642
H	-1.537494	-1.216708	-3.765261
H	-3.276504	-1.551499	-3.828180
H	-0.145843	0.113497	1.642949
C	-0.037283	-2.488542	3.077679
C	1.045650	-1.617086	2.403124
C	0.646010	-0.183265	2.776441
O	2.349030	-2.062399	2.751386
H	0.999559	-1.794929	1.320768
N	-0.809472	-1.652430	3.833721
H	-1.573566	-1.967699	4.420077
C	-0.391097	-0.291938	3.805557
O	-0.176191	-3.699485	2.976395
O	-0.938932	0.549943	4.509588
C	1.683452	0.871666	2.859054
C	1.569617	1.991214	3.707324
C	2.805543	0.809565	2.011005
C	2.526001	3.006173	3.684115
H	0.712463	2.065987	4.364171
C	3.753374	1.831342	1.980468
H	2.939144	-0.049318	1.370380
C	3.618187	2.943879	2.813817
H	2.408645	3.860290	4.346392
H	4.595823	1.749346	1.298642
H	4.351225	3.745101	2.789706
C	2.655748	-1.985936	4.138528
H	2.006709	-2.644989	4.731440
H	3.689578	-2.321401	4.241790
H	2.570121	-0.959550	4.513465

----

b-RR-TS2

Gsol = -3018.62503100 Hartree

Rh	-0.818006	-1.515622	-0.533818
S	2.194010	-1.466293	-1.648304
O	2.141676	-2.880722	-1.204730
O	2.149714	-1.280235	-3.123405
O	-5.362552	3.668893	-1.820283
N	1.044367	-0.653006	-0.850112
N	-1.018396	0.249951	0.505732
C	1.187328	0.753238	-0.412096

C	-0.253063	1.277795	-0.244300
C	1.962685	1.688063	-1.316790
C	1.682972	1.788095	-2.683665
C	2.392313	2.681813	-3.485706
C	3.382476	3.494299	-2.927140
C	3.655447	3.409894	-1.560148
C	2.947187	2.511880	-0.763200
C	-0.326759	2.661662	0.368813
C	0.206725	2.931751	1.634058
C	0.084950	4.199633	2.201886
C	-0.559922	5.222734	1.502242
C	-1.073340	4.969536	0.228134
C	-0.958684	3.695368	-0.331153
C	3.750567	-0.780780	-1.091696
C	4.108525	-0.901068	0.252268
C	5.319213	-0.362711	0.680458
C	6.176941	0.296247	-0.213671
C	5.793002	0.396828	-1.556891
C	4.587439	-0.139622	-2.002017
C	7.491604	0.863504	0.260311
C	-2.426401	0.640704	0.745880
C	-3.262205	0.929395	-0.480894
C	-3.927462	2.157525	-0.555834
C	-4.707530	2.487488	-1.668716
C	-4.830568	1.571071	-2.722916
C	-4.168774	0.354129	-2.651887
C	-3.384378	0.009585	-1.538193
C	-5.225215	4.645227	-0.791085
C	-2.671175	-1.281329	-1.554634
C	-2.842166	-2.401708	-0.676604
C	-1.953192	-3.449734	-1.141320
C	-1.204103	-2.980074	-2.247964
C	-1.618830	-1.615621	-2.504023
C	-3.862944	-2.546952	0.409021
C	-1.933896	-4.835896	-0.596964
C	-0.256858	-3.773773	-3.089862
C	-1.156855	-0.760745	-3.637122
H	1.661852	0.761704	0.573456
H	-0.690185	1.343659	-1.249427
H	0.932319	1.142449	-3.122823
H	2.174521	2.741778	-4.548625
H	3.937616	4.186958	-3.553618
H	4.424832	4.035872	-1.116825
H	3.168958	2.433163	0.297163

H	0.739448	2.153576	2.164225
H	0.493995	4.378030	3.192164
H	-0.655585	6.211243	1.942687
H	-1.563273	5.762236	-0.330956
H	-1.370929	3.497216	-1.316206
H	3.447575	-1.398934	0.953647
H	5.606387	-0.454911	1.724363
H	6.439872	0.912867	-2.261009
H	4.274031	-0.039001	-3.033765
H	7.421383	1.225673	1.291097
H	8.279158	0.099066	0.236325
H	7.819960	1.692004	-0.374875
H	-2.868804	-0.160325	1.340701
H	-2.428733	1.534472	1.379531
H	-3.801349	2.861444	0.256617
H	-5.434975	1.835275	-3.584193
H	-4.259256	-0.350426	-3.473513
H	-5.631177	4.280221	0.159864
H	-4.175654	4.930434	-0.651176
H	-5.798705	5.511050	-1.123583
H	-3.398088	-2.811313	1.364111
H	-4.421369	-1.617338	0.541560
H	-4.581511	-3.335941	0.154647
H	-2.190737	-4.855397	0.462531
H	-2.678271	-5.436524	-1.137876
H	-0.959437	-5.310658	-0.726106
H	0.520713	-3.137636	-3.514931
H	0.235947	-4.552832	-2.505396
H	-0.804444	-4.253283	-3.912770
H	-1.364935	0.293231	-3.437289
H	-0.085657	-0.889544	-3.804804
H	-1.691570	-1.039044	-4.555024
H	-0.636768	0.014083	1.766277
C	0.217913	1.229934	4.510255
C	0.741320	0.013468	3.716459
C	-0.511020	-0.542824	3.041553
O	1.765651	0.353637	2.802006
H	1.145120	-0.708137	4.448404
N	-1.142909	1.073302	4.587931
H	-1.754213	1.721719	5.070914
C	-1.636892	-0.001349	3.812005
O	0.865908	2.154291	4.975822
O	-2.825504	-0.308995	3.812705
C	-0.493319	-1.978502	2.680184

C	-1.366574	-2.939021	3.227693
C	0.490538	-2.421125	1.764451
C	-1.191579	-4.291584	2.950995
H	-2.151989	-2.617216	3.901098
C	0.657948	-3.787824	1.487895
H	1.200651	-1.705829	1.370264
C	-0.167543	-4.726162	2.095940
H	-1.849754	-5.020244	3.417628
H	1.433284	-4.082718	0.789680
H	-0.038185	-5.785470	1.894917
C	3.042556	0.555731	3.404656
H	3.009681	1.361801	4.141476
H	3.390631	-0.369599	3.886423
H	3.722730	0.818978	2.593893

SR-pro1

Gsol = -705.539462843 Hartree

C	-2.765636	-0.253005	-0.112080
C	-1.413795	-0.954979	-0.341698
C	-0.460382	0.202797	-0.718600
O	-1.005944	-1.528678	0.883715
H	-1.493820	-1.716337	-1.126824
H	-0.522708	0.320747	-1.808370
N	-2.489389	1.093538	0.063656
H	-3.190714	1.766214	0.354528
C	-1.159808	1.446139	-0.153480
O	-3.861422	-0.769843	-0.016468
O	-0.694083	2.549169	0.033943
C	0.986782	0.046067	-0.330051
C	1.934476	-0.286180	-1.303405
C	1.405216	0.209551	0.996883
C	3.277183	-0.458701	-0.961643
H	1.619820	-0.410112	-2.336671
C	2.745577	0.042478	1.340118
H	0.678788	0.464905	1.760982
C	3.685774	-0.294079	0.362326
H	4.000833	-0.716214	-1.729667
H	3.056957	0.174439	2.372380
H	4.730146	-0.423249	0.631584
C	-1.649482	-2.762720	1.188192
H	-2.728419	-2.634671	1.328276
H	-1.198444	-3.120880	2.115715
H	-1.477112	-3.497920	0.389608

RR-pro1

Gsol = -705.540330403 Hartree

C	-2.707194	-0.001765	-0.443568
C	-1.394087	-0.802679	-0.347314
C	-0.478255	0.087233	0.538460
H	-0.634535	-0.197089	1.586709
N	-2.393349	1.302003	-0.086791
H	-3.052404	2.069179	-0.170688
C	-1.103221	1.478932	0.406019
O	-3.799511	-0.400931	-0.788107
O	-0.612149	2.549796	0.692594
C	0.991304	0.007225	0.219791
C	1.836056	-0.771629	1.017251
C	1.523822	0.655730	-0.902382
C	3.189743	-0.902562	0.702644
H	1.431035	-1.279251	1.888859
C	2.876114	0.528425	-1.217233
H	0.883459	1.271691	-1.528073
C	3.713219	-0.251995	-0.415676
H	3.833323	-1.510142	1.332211
H	3.276777	1.041114	-2.086897
H	4.766790	-0.349482	-0.660711
O	-1.543570	-2.142750	0.022786
H	-0.974704	-0.827637	-1.360406
C	-2.208112	-2.376796	1.260963
H	-1.637120	-1.989062	2.114768
H	-3.212496	-1.938228	1.267639
H	-2.289079	-3.460669	1.359262

----

b-S-TS1

Gsol = -3018.62653369 Hartree

Rh	0.672410	-0.986629	-0.469770
S	0.654349	2.105400	-1.494135
O	2.122594	1.940618	-1.367743
O	0.171319	2.159411	-2.900504
O	-5.425725	-4.466147	-0.534604
N	-0.084043	0.967371	-0.613487
N	-0.862250	-1.024787	0.972955
C	-1.288964	1.251972	0.176985
C	-1.936334	-0.110756	0.497787
C	-2.342904	2.135941	-0.466405

C	-2.767764	1.910016	-1.779347
C	-3.770344	2.700513	-2.340616
C	-4.363276	3.720042	-1.591494
C	-3.949893	3.941303	-0.275977
C	-2.946723	3.150205	0.281512
C	-3.083719	0.003290	1.475490
C	-2.861055	0.357714	2.812221
C	-3.932091	0.465633	3.700164
C	-5.235935	0.225011	3.260121
C	-5.464385	-0.124974	1.927422
C	-4.391346	-0.237820	1.042726
C	0.210782	3.665206	-0.735425
C	0.482798	3.860297	0.619390
C	0.104599	5.057235	1.221193
C	-0.533717	6.066621	0.484964
C	-0.785593	5.845460	-0.875925
C	-0.419502	4.650827	-1.491004
C	-0.927331	7.367190	1.139862
C	-1.346872	-2.400735	1.280260
C	-2.061394	-3.061087	0.121232
C	-3.377313	-3.481719	0.345713
C	-4.137199	-4.059007	-0.676372
C	-3.566900	-4.226998	-1.945382
C	-2.264880	-3.806526	-2.172389
C	-1.487588	-3.220058	-1.158626
C	-6.062041	-4.245454	0.721818
C	-0.133174	-2.741104	-1.497825
C	1.104035	-3.111787	-0.833432
C	2.164046	-2.376886	-1.462776
C	1.600644	-1.507616	-2.448663
C	0.171869	-1.762100	-2.500113
C	1.300019	-4.234268	0.141148
C	3.611597	-2.605369	-1.180443
C	2.358437	-0.625600	-3.390975
C	-0.788439	-1.095614	-3.431666
H	-1.009336	1.712369	1.136433
H	-2.304888	-0.533834	-0.439111
H	-2.288101	1.137049	-2.367700
H	-4.085342	2.523897	-3.365434
H	-5.139743	4.339581	-2.031537
H	-4.402228	4.734262	0.312986
H	-2.612776	3.331514	1.298582
H	-1.850812	0.544659	3.167242
H	-3.747852	0.738592	4.735143

H	-6.068720	0.310661	3.952039
H	-6.475721	-0.308950	1.576171
H	-4.566072	-0.511645	0.006413
H	0.948569	3.068218	1.193175
H	0.299484	5.209337	2.279731
H	-1.290248	6.612759	-1.456937
H	-0.645379	4.460778	-2.533369
H	-1.121529	7.233385	2.208764
H	-0.127077	8.112588	1.043422
H	-1.824206	7.790889	0.676671
H	-0.470461	-2.964387	1.603663
H	-2.034270	-2.346339	2.129423
H	-3.809639	-3.321947	1.324974
H	-4.159783	-4.675584	-2.735469
H	-1.829272	-3.932379	-3.158857
H	-5.566454	-4.806832	1.522892
H	-6.073764	-3.179337	0.978838
H	-7.084579	-4.605448	0.604608
H	1.850912	-3.911435	1.029882
H	0.342926	-4.656892	0.456243
H	1.877278	-5.041860	-0.326718
H	3.812858	-2.665216	-0.109117
H	3.911210	-3.565715	-1.622807
H	4.240324	-1.820216	-1.598483
H	1.773337	0.255133	-3.661503
H	3.290043	-0.278958	-2.939885
H	2.602291	-1.176441	-4.309685
H	-1.816579	-1.206070	-3.077871
H	-0.558476	-0.030872	-3.521771
H	-0.727675	-1.546524	-4.431032
H	-0.467990	-0.657781	1.847010
C	1.737372	-0.913696	2.851182
C	2.550169	-0.055710	1.877225
C	3.829962	-0.662211	1.755670
O	2.252925	1.271900	1.984630
H	1.776161	-0.408076	0.699659
N	2.476341	-2.035285	3.051357
H	2.212974	-2.792711	3.668979
C	3.771409	-1.938106	2.441773
O	0.641515	-0.646924	3.336549
O	4.587399	-2.849483	2.537582
C	4.915021	-0.294411	0.853485
C	6.229810	-0.761282	1.056579
C	4.656308	0.492546	-0.287180

C	7.242750	-0.446443	0.153420
H	6.443417	-1.382953	1.917606
C	5.676951	0.808560	-1.181877
H	3.648294	0.828659	-0.497599
C	6.976950	0.342577	-0.970072
H	8.249040	-0.817389	0.330403
H	5.446789	1.415160	-2.054098
H	7.771419	0.587634	-1.669380
C	3.317835	2.229901	1.960197
H	3.575806	2.492592	0.933040
H	2.931735	3.107315	2.481795
H	4.195371	1.840247	2.482013

----

b-S-int2

Gsol = -3018.64426396 Hartree

Rh	-0.886552	-1.569505	-0.423706
S	1.969093	-1.343788	-2.043277
O	1.812060	-2.805842	-1.860338
O	1.954468	-0.885281	-3.454064
O	-5.587598	3.659910	-1.043579
N	0.856250	-0.607929	-1.122667
N	-0.987950	0.269329	0.608673
C	1.056421	0.764558	-0.617873
C	-0.350173	1.291775	-0.270103
C	1.729550	1.769858	-1.531872
C	1.295148	1.980008	-2.843846
C	1.893224	2.960595	-3.634944
C	2.924240	3.749226	-3.116928
C	3.351159	3.553911	-1.801787
C	2.755068	2.568190	-1.016156
C	-0.354661	2.685018	0.314230
C	0.341398	2.980311	1.490360
C	0.327578	4.272878	2.012649
C	-0.373689	5.287865	1.357750
C	-1.060538	5.002528	0.175102
C	-1.051299	3.705968	-0.340978
C	3.574418	-0.908320	-1.378255
C	4.008167	-1.537001	-0.208361
C	5.246433	-1.199822	0.327795
C	6.061454	-0.234859	-0.286892
C	5.610382	0.365768	-1.468225
C	4.373002	0.034792	-2.019943
C	7.382881	0.151367	0.328957

C	-2.348204	0.630936	1.092376
C	-3.329714	0.914932	-0.016741
C	-4.007918	2.137283	0.014128
C	-4.911241	2.484759	-0.995628
C	-5.140855	1.592182	-2.052882
C	-4.462527	0.383592	-2.088983
C	-3.556975	0.018762	-1.077872
C	-5.332727	4.621791	-0.021131
C	-2.848990	-1.268657	-1.216345
C	-2.926695	-2.413667	-0.344022
C	-2.089054	-3.441142	-0.921696
C	-1.493707	-2.949198	-2.117251
C	-1.938447	-1.582196	-2.296140
C	-3.882733	-2.600727	0.791036
C	-1.942926	-4.822380	-0.375882
C	-0.702519	-3.735537	-3.110881
C	-1.602776	-0.706140	-3.458524
H	1.636833	0.724488	0.313003
H	-0.934252	1.303179	-1.193270
H	0.509314	1.356433	-3.253003
H	1.556344	3.107841	-4.657311
H	3.390528	4.511283	-3.735112
H	4.150987	4.162938	-1.390142
H	3.091226	2.406192	0.004062
H	0.894529	2.207095	2.006556
H	0.870211	4.479978	2.930230
H	-0.381920	6.295647	1.763091
H	-1.600127	5.787816	-0.346961
H	-1.588558	3.482799	-1.258121
H	3.388334	-2.290661	0.262343
H	5.587150	-1.689121	1.236414
H	6.231372	1.108101	-1.961850
H	4.022440	0.507239	-2.929525
H	7.993257	0.734151	-0.366930
H	7.229984	0.760119	1.229407
H	7.954812	-0.732604	0.631713
H	-2.675914	-0.197313	1.721460
H	-2.261938	1.508443	1.737947
H	-3.795851	2.824068	0.823054
H	-5.839028	1.869808	-2.835255
H	-4.634789	-0.300061	-2.914860
H	-5.621586	4.238952	0.964617
H	-4.275140	4.910284	-0.004161
H	-5.945766	5.488410	-0.269700

H	-3.446331	-3.209882	1.584773
H	-4.201188	-1.642328	1.206832
H	-4.783380	-3.113245	0.427598
H	-1.931130	-4.815213	0.715364
H	-2.797451	-5.427944	-0.706723
H	-1.032183	-5.301978	-0.740555
H	-0.020722	-3.097297	-3.672568
H	-0.105986	-4.510982	-2.629396
H	-1.393439	-4.216820	-3.816687
H	-1.822458	0.340184	-3.235083
H	-0.547481	-0.796160	-3.724720
H	-2.206621	-0.997267	-4.327698
H	-0.426754	0.112273	1.463529
C	-1.230681	-2.849744	3.073841
C	0.136504	-2.212477	2.747336
C	0.030298	-0.813273	3.236306
O	0.405443	-2.368951	1.303584
H	0.941761	-2.782801	3.230731
N	-1.957428	-1.891103	3.693630
H	-2.900913	-2.020232	4.036478
C	-1.227067	-0.645330	3.869775
O	-1.594604	-3.988525	2.787299
O	-1.761289	0.294176	4.473244
C	1.169049	0.074870	3.389221
C	1.144657	1.191879	4.261296
C	2.348727	-0.125933	2.634543
C	2.233609	2.053344	4.357462
H	0.247430	1.374710	4.839995
C	3.431193	0.748082	2.729622
H	2.395285	-0.956545	1.939173
C	3.384961	1.849323	3.587798
H	2.180223	2.900510	5.037553
H	4.308122	0.569231	2.113712
H	4.225214	2.534301	3.655172
C	1.022867	-3.644384	1.020372
H	1.950576	-3.721262	1.600756
H	0.353886	-4.459696	1.298343
H	1.248866	-3.648081	-0.042582

----

b-RS-TS2

Gsol = -3018.62363485 Hartree

Rh	-0.910485	-1.438148	-0.647552
S	2.011199	-1.260517	-2.013074

O	1.983870	-2.705678	-1.681539
O	1.873104	-0.950445	-3.459830
O	-5.616460	3.787688	-0.756464
N	0.911141	-0.518301	-1.083738
N	-0.982818	0.217268	0.574332
C	1.099495	0.832516	-0.530513
C	-0.312577	1.323719	-0.151288
C	1.784542	1.862874	-1.406539
C	1.384587	2.082127	-2.728337
C	2.009360	3.060926	-3.500943
C	3.035056	3.837900	-2.956602
C	3.429763	3.631714	-1.633045
C	2.805755	2.649599	-0.865683
C	-0.306058	2.635570	0.606459
C	0.424488	2.794336	1.787762
C	0.394296	3.992332	2.498611
C	-0.365997	5.063744	2.023726
C	-1.082524	4.926303	0.832611
C	-1.052276	3.719248	0.131461
C	3.588769	-0.600024	-1.487542
C	4.003890	-0.792451	-0.168252
C	5.204841	-0.228898	0.254077
C	6.001256	0.520115	-0.625116
C	5.572633	0.673538	-1.950288
C	4.370145	0.123487	-2.385579
C	7.275284	1.170437	-0.147537
C	-2.344957	0.557988	1.040149
C	-3.333220	0.963642	-0.031825
C	-4.021212	2.171149	0.129072
C	-4.930070	2.617332	-0.835709
C	-5.158685	1.841055	-1.980858
C	-4.476247	0.644518	-2.143022
C	-3.564878	0.182397	-1.178557
C	-5.374990	4.628864	0.368486
C	-2.855498	-1.084326	-1.440204
C	-2.943062	-2.312682	-0.704697
C	-2.120383	-3.287532	-1.397545
C	-1.476462	-2.666831	-2.493671
C	-1.906711	-1.281725	-2.524438
C	-3.849491	-2.605751	0.449402
C	-2.078608	-4.739299	-1.067446
C	-0.617262	-3.333246	-3.520550
C	-1.555621	-0.285843	-3.579206
H	1.687152	0.759455	0.395551

H	-0.858183	1.488291	-1.089033
H	0.606282	1.464863	-3.158983
H	1.698017	3.214072	-4.530616
H	3.524330	4.596612	-3.560948
H	4.229191	4.227451	-1.201572
H	3.128023	2.473592	0.156432
H	1.042327	1.985371	2.148618
H	0.966490	4.079744	3.417614
H	-0.393219	6.000616	2.572566
H	-1.663931	5.759136	0.446749
H	-1.618417	3.612572	-0.788910
H	3.400511	-1.379036	0.516999
H	5.533837	-0.373166	1.279626
H	6.180585	1.245478	-2.645883
H	4.018098	0.271193	-3.399226
H	8.031072	1.196864	-0.938994
H	7.088882	2.207799	0.159819
H	7.695631	0.643660	0.714613
H	-2.700655	-0.309906	1.601058
H	-2.266023	1.379767	1.760168
H	-3.812323	2.769755	1.006362
H	-5.860915	2.195918	-2.727724
H	-4.650352	0.047623	-3.033543
H	-5.658298	4.132673	1.304297
H	-4.321370	4.928256	0.421413
H	-5.998919	5.510774	0.220272
H	-3.299086	-3.047812	1.285735
H	-4.338673	-1.693827	0.799911
H	-4.633535	-3.312753	0.150515
H	-2.250900	-4.916178	-0.005688
H	-2.874069	-5.247912	-1.629529
H	-1.126175	-5.194621	-1.346100
H	0.125109	-2.643067	-3.924270
H	-0.081843	-4.184842	-3.096531
H	-1.241607	-3.696394	-4.348261
H	-1.724631	0.733658	-3.223855
H	-0.510775	-0.393968	-3.877885
H	-2.188287	-0.440079	-4.463315
H	-0.417497	-0.126739	1.739770
C	1.182408	0.795556	4.243138
C	1.384528	-0.334888	3.210460
C	-0.035181	-0.781296	2.884970
O	2.116745	-1.395218	3.817920
H	1.958142	0.043302	2.351304

N	-0.123224	0.718486	4.654886
H	-0.524905	1.324541	5.360415
C	-0.889035	-0.240169	3.951369
O	2.022900	1.586452	4.643426
O	-2.067737	-0.452084	4.214896
C	-0.216039	-2.166948	2.412205
C	-1.099305	-3.090547	3.005254
C	0.563854	-2.606925	1.314816
C	-1.107936	-4.419918	2.596271
H	-1.736366	-2.764309	3.818109
C	0.550008	-3.952474	0.910787
H	1.297021	-1.941193	0.878789
C	-0.267075	-4.865931	1.564368
H	-1.762596	-5.126391	3.100304
H	1.190071	-4.248982	0.087316
H	-0.270456	-5.910994	1.270360
C	3.511244	-1.138522	3.929423
H	3.942464	-0.892612	2.948048
H	3.722179	-0.313791	4.619151
H	3.967097	-2.057962	4.303081

----

b-SS-TS2

Gsol = -3018.62676574 Hartree

Rh	-0.889318	-1.591199	-0.375930
S	1.906199	-1.346588	-2.049825
O	1.796160	-2.806673	-1.814141
O	1.832221	-0.938232	-3.475348
O	-5.706461	3.496487	-0.971920
N	0.814573	-0.610299	-1.111154
N	-0.999990	0.211048	0.652953
C	0.976553	0.781411	-0.647958
C	-0.441627	1.239090	-0.249039
C	1.564839	1.788795	-1.614763
C	1.082155	1.929888	-2.919282
C	1.601402	2.913436	-3.761104
C	2.601906	3.775010	-3.302870
C	3.077039	3.649251	-1.995611
C	2.559580	2.660282	-1.159739
C	-0.478085	2.646970	0.309349
C	0.193794	2.985653	1.487654
C	0.156114	4.290623	1.977607
C	-0.550308	5.279559	1.288894
C	-1.216364	4.952644	0.105269

C	-1.179994	3.643466	-0.377543
C	3.531753	-0.858482	-1.469830
C	4.077978	-1.518554	-0.366649
C	5.339497	-1.151977	0.092843
C	6.064969	-0.123841	-0.530036
C	5.501966	0.508890	-1.645274
C	4.243280	0.146049	-2.122634
C	7.408738	0.300024	0.008009
C	-2.354955	0.544594	1.132348
C	-3.384869	0.801779	0.054446
C	-4.081862	2.013692	0.078689
C	-5.012217	2.329250	-0.916742
C	-5.255842	1.413502	-1.950359
C	-4.560279	0.213569	-1.979534
C	-3.622976	-0.115308	-0.986027
C	-5.433551	4.482556	0.020977
C	-2.885350	-1.386921	-1.109773
C	-2.897582	-2.501936	-0.201760
C	-2.052511	-3.529316	-0.778720
C	-1.520211	-3.068606	-2.009616
C	-1.994079	-1.712195	-2.205357
C	-3.807722	-2.680928	0.973062
C	-1.842897	-4.878537	-0.173488
C	-0.738373	-3.860692	-3.007199
C	-1.718217	-0.861819	-3.402425
H	1.596226	0.789991	0.256893
H	-1.036387	1.242706	-1.172415
H	0.320940	1.249998	-3.281807
H	1.227062	3.005957	-4.776983
H	3.007054	4.539045	-3.960578
H	3.853340	4.315340	-1.629442
H	2.934433	2.552317	-0.145773
H	0.750849	2.232390	2.027144
H	0.683820	4.529826	2.896763
H	-0.578716	6.297139	1.668392
H	-1.760912	5.715826	-0.444190
H	-1.701644	3.389520	-1.295746
H	3.528300	-2.322708	0.108140
H	5.768951	-1.669076	0.946896
H	6.054003	1.298241	-2.148022
H	3.813757	0.635074	-2.988238
H	7.995256	0.826863	-0.750590
H	7.288964	0.978789	0.862744
H	7.988858	-0.560437	0.357507

H	-2.669552	-0.278248	1.779035
H	-2.286798	1.434758	1.767617
H	-3.859955	2.719133	0.869057
H	-5.976673	1.666508	-2.720635
H	-4.742648	-0.488710	-2.787721
H	-5.694457	4.121058	1.022761
H	-4.378013	4.778994	0.005922
H	-6.059176	5.339451	-0.230950
H	-3.302299	-3.174797	1.805543
H	-4.205776	-1.722290	1.313518
H	-4.663914	-3.305160	0.684406
H	-1.780484	-4.814303	0.914827
H	-2.692840	-5.526447	-0.426380
H	-0.935841	-5.352854	-0.554617
H	-0.056699	-3.225816	-3.573342
H	-0.140988	-4.636782	-2.526422
H	-1.430039	-4.344316	-3.710761
H	-1.937412	0.187367	-3.191609
H	-0.674283	-0.948121	-3.711606
H	-2.356051	-1.177518	-4.238362
H	-0.348558	-0.066860	1.824057
C	-0.997262	-2.658362	3.220751
C	0.307787	-2.022073	2.716338
C	0.153722	-0.535830	2.997067
O	0.524937	-2.319343	1.328689
H	1.150147	-2.456332	3.273988
N	-1.645304	-1.688330	3.929870
H	-2.534066	-1.830131	4.396645
C	-0.984213	-0.427789	3.920322
O	-1.393489	-3.797489	3.014085
O	-1.418406	0.521746	4.559934
C	1.396339	0.246614	3.245922
C	1.473910	1.284728	4.193191
C	2.527808	-0.000621	2.446790
C	2.632656	2.050606	4.319901
H	0.608299	1.502802	4.805984
C	3.683144	0.771285	2.571859
H	2.488221	-0.784846	1.698657
C	3.742221	1.806460	3.506440
H	2.664423	2.848629	5.057375
H	4.529342	0.565632	1.923719
H	4.637549	2.414688	3.598425
C	1.150581	-3.602531	1.125889
H	2.095002	-3.622743	1.683723

H	0.500573	-4.404359	1.478138
H	1.343957	-3.674870	0.058584

RS-pro1

Gsol = -705.539462843 Hartree

C	-2.765636	-0.253005	0.112080
C	-1.413795	-0.954979	0.341698
C	-0.460382	0.202797	0.718600
O	-1.005944	-1.528678	-0.883715
H	-1.493820	-1.716337	1.126824
H	-0.522708	0.320747	1.808370
N	-2.489389	1.093538	-0.063656
H	-3.190714	1.766214	-0.354528
C	-1.159808	1.446139	0.153480
O	-3.861422	-0.769843	0.016468
O	-0.694083	2.549169	-0.033943
C	0.986782	0.046067	0.330051
C	1.934476	-0.286180	1.303405
C	1.405216	0.209551	-0.996883
C	3.277183	-0.458701	0.961643
H	1.619820	-0.410112	2.336671
C	2.745577	0.042478	-1.340118
H	0.678788	0.464905	-1.760982
C	3.685774	-0.294079	-0.362326
H	4.000833	-0.716214	1.729667
H	3.056957	0.174439	-2.372380
H	4.730146	-0.423249	-0.631584
C	-1.649482	-2.762720	-1.188192
H	-2.728419	-2.634671	-1.328276
H	-1.198444	-3.120880	-2.115715
H	-1.477112	-3.497920	-0.389608

SS-pro1

Gsol = -705.540330403 Hartree

C	-2.707194	-0.001765	0.443568
C	-1.394087	-0.802679	0.347314
C	-0.478255	0.087233	-0.538460
H	-0.634535	-0.197089	-1.586709
N	-2.393349	1.302003	0.086791
H	-3.052404	2.069179	0.170688
C	-1.103221	1.478932	-0.406019
O	-3.799511	-0.400931	0.788107

O	-0.612149	2.549796	-0.692594
C	0.991304	0.007225	-0.219791
C	1.836056	-0.771629	-1.017251
C	1.523822	0.655730	0.902382
C	3.189743	-0.902562	-0.702644
H	1.431035	-1.279251	-1.888859
C	2.876114	0.528425	1.217233
H	0.883459	1.271691	1.528073
C	3.713219	-0.251995	0.415676
H	3.833323	-1.510142	-1.332211
H	3.276777	1.041114	2.086897
H	4.766790	-0.349482	0.660711
O	-1.543570	-2.142750	-0.022786
H	-0.974704	-0.827637	1.360406
C	-2.208112	-2.376796	-1.260963
H	-1.637120	-1.989062	-2.114768
H	-3.212496	-1.938228	-1.267639
H	-2.289079	-3.460669	-1.359262
